# Supplementary material for: Exploring the narrative landscape: The discursive construction of identity for Chinese enterprises in Africa
Source: PLoS One. 2025 Feb 28;20(2):e0314285. doi: 10.1371/journal.pone.0314285 (PMC11870363; doi:10.1371/journal.pone.0314285)
Supplement: S1_File — This file contains a comprehensive collection of data on 200 Chinese enterprises operating in Africa, including sections such as “About Us,” “Corporate Responsibility,” and “Culture.” (PDF) [file pone.0314285.s001.pdf]

1.CMOC

CMOC Group Limited (“CMOC” or the “Company”) was founded in 1969 and has since completed two respective mixed ownership reforms in 2004 and 2014. CMOC now is a joint stock company with a private company as its controlling shareholder. It was listed on Hong Kong Exchanges (HKEX: 03993) in 2007 and Shanghai Stock Exchange (SSE: 603993) in 2012.

The Company engages in the non-ferrous metal industry, mainly the mining and processing business, which includes mining, beneficiation, smelting and refining of base and rare metals, and mineral trading business. With its main business located over five continents: Asia, Africa, South America, Oceania and Europe, the Company is the world’s leading producer of tungsten, cobalt, niobium and molybdenum and a major copper producer. It is also a leading producer of phosphate fertilizer in Brazil. In terms of trading business, the Company is among the top three base metal traders in the world. The Company ranks 1,046 among the top 2,000 global listed companies by Forbes 2021 and 15 among top 40 global mining companies (by market value) in 2021.

The Company’s vision is to become a “highly respected, modern and world-class resource company”. In the context of global strategy of carbon neutrality, the Company has formulated a “three-step” development path, of which the first step is to “lay a foundation of low cost and high efficiency” that focuses on system and team building via organization upgrade and a corporate management system that covers global operations. The second step is to “ramp up with multiplying production capacity” as the two world-class projects, TFM mixed ore project and KFM project, complete construction and start production. This step aims at a modern and effective corporate governance structure with clear division of responsibilities and a stronger team that is tested through involvement in world-class projects that are launched to expand our production capacity. The third step is to achieve “stellar growth” towards a world-class company and our vision when new milestones are achieved in business size and cash flows, when teams and project pipelines are more robust, and when we are ready to seek greater growth around our strategic goals in key regions and key products.

Our approach

As a company with a growing international presence, CMOC fully recognizes the importance of sustainable development in its access to resources, markets, and financing. In recent years, international and Chinese stakeholders' expectations of sustainability-related issues have also been evolving, and we face a combination of increasingly high expectations and strict regulatory requirements on matters including the environment, tailings, health and safety, and contractor management. In 2022, we updated our ESG strategy to take account of these changing expectations and regulatory requirements.

Following the completion of the first phase of our three-step growth strategy, we have established a three-tier ESG management framework with clear divisions of responsibility for Board members, senior executives, and our individual operating sites. Over the coming years, as part of the second phase of our growth strategy, we will be introducing a range of measures to improve ESG performance, with a focus on four key areas.

First, we will place ESG at the core of our management by developing a systematic ESG management framework, aligning with leading international benchmarks, and prioritizing ESG

risks at all stages of our operations, from the planning of new sites through to production.

Second, we will also incorporate ESG into our organizational culture by expanding our ESG talent pool, improving training for management-level staff, and introducing ESG KPIs to gauge performance on environmental, social, and governance topics.

Third, we will encourage management practices that balance short-term performance with long-term value, continue to improve ESG performance, attach greater importance to medium and long-term forecasts, and take proactive steps to prevent and mitigate material risks.

Fourth, we will improve our ESG communications strategy to raise awareness of our ESG-related initiatives, both within the company and among stakeholders.

CMOC's compliance and sustainability policies are applicable to the entire Group, as well as all of its directly or indirectly controlled or majority-owned subsidiaries. As part of our commitment to align with international benchmarks, these policies reference international best practice frameworks, including the sustainable development principles of the International Council on Mining and Metals (ICMM), the International Finance Corporation's (IFC) Environmental and Social Performance Standards, the International Labor Organization (ILO) Conventions, the International Bill of Human Rights, the UN Guiding Principles on Business and Human Rights (UNGPs), the Voluntary Principles on Security and Human Rights (VPSHR), and the OECD Due Diligence Guidance for Responsible Supply Chains of Minerals from Conflict-Affected and High-Risk Areas (“OECD Guidance”). Guided by these policies, each of our sites has formulated and revised its own measures and procedures to reflect actual circumstances and material risks.

Responsible mining for a better world – this aspiration lies at the heart of CMOC’s founding principles and mission. In 2022, we once again demonstrated our unwavering commitment to sustainable development by joining the United Nations Global Compact, under which we will implement the Ten Principles on human rights, labor standards, the environment, and anti-corruption. We will also work with all our stakeholders to address global challenges such as poverty, inequality, climate change, environmental degradation, and other issues concerning prosperity, peace, and justice. In addition to encouraging us to uphold our fundamental responsibilities to people and the planet, the sustainable business practices envisaged in the Ten Principles will also help CMOC to secure its long-term success.

Social responsibility

CMOC reviewed in 2019 and updated in February 2020 its Community Policy applicable to the entire group. We are committed to conducting activities in a manner that promotes positive and open relationships with local communities. We continue our engagement with and investment in communities affected by our mining operations, using multi-channel grievance systems to collect their feedback and concerns. We develop infrastructure, support health, safety and education efforts, and provide local employment and business development opportunities. Our goal is to build such partnerships at all levels of community, from local to national, by making long-term positive contributions and effectively mitigating adverse impacts from our operations. Stakeholder relations across the international portfolio are guided by a risk-based approach to evaluating community expectations and needs, which informs community development plans. At our NPM, Brazilian and DRC operations, stakeholder communications, community risk and impact management and community investment plans guide our external engagement efforts. At our Chinese site, the national priorities of the government also play a role in driving community

relations and associated investments. In all cases, stakeholder engagement is based on mutual trust, communications based on transparent dialogue, and the effective mitigation of impact coupled with the enhancement of positive effects from mine operations. CMOC understands that these aspects are material to the success of our mining business.

Our community relations programs are guided by local laws and regulations and seek alignment with international best practice frameworks such as the International Finance Corporation and the ICMM sustainable development principles. Social engagement and community investment activities may also be governed by commitments made through the process of Environmental and Social Impact Assessment or their equivalents under national regulations.

Our operation in China closely aligns community spending with government development priorities and makes pertinent community investments to enhance performance in achieving those priorities. The Chinese operation is located in Luanchuan, a small mountain county in Henan province rich in minerals but with scarce agricultural resources along with frequent public emergencies due to floods and landslides. As an important company in Luanchuan County, we have long contributed to local development. In 2019 CMOC multi-year investment programs contributed to the host county close on a total of RMB130 million in donations for social development, poverty alleviation, education, health and other domains. After years of effort, in 2019 Luanchuan County was officially taken off China’s list of poorest areas. CMOC’s contributions have been widely recognized by society, and we were awarded the “2019 Henan Provincial Award for Poverty Alleviation and Dedication” by the Henan Provincial Government.

In the DRC our operation is near rural villages, agriculture and two towns which have steadily undergone significant population influx and urbanization in recent years. In recognition of TFM’s responsibility to maximize local opportunities, 93% of employees at the TFM mine are DRC nationals. The sustainability programs at this site continued to address acute community development priorities. Therefore, we have strengthened communication and cooperation with local governments at all levels. In addition, TFM has strengthened control over social projects to maximize benefits for local people. TFM’s community programs prioritize investments in five areas: education, infrastructure, community health, agriculture and capacity building.

Our mines in Australia and Brazil are sited in areas that are largely rural, with surrounding land uses dominated by agriculture. NPM and Brazil continue to strengthen the effectiveness of community plans through transparent identification of impact, communication through stakeholder forums and the maintenance of systems to register and respond to major community concerns. NPM's community programs have four key focus areas: economic development, education and training, health and safety, environment and cultural heritage. In our Brazilian operations, based on social diagnosis of community expectations and international good practices, we have prioritized investment in education and training, income generation, agriculture and livestock, infrastructure and environment. This approach allows us to maintain good neighborly relations, because communities feel benefits from our operations in ways that are important to them.

Corporate culture

Meritocracy

We reject mediocrity; thus, we pursue excellence. We appreciate change; thus, we embrace competition. We have a keen sense of crisis and the drive to innovate, and we dare refute

ourselves and transcend our limits.
We are steadfast in our mission, and with clarity of purpose and thought, we stay the course and take charge. We strive with an entrepreneurial spirit, and we speak with results.
We give the mandate to share, we respect and accommodate, and we hire by merit. We believe that only growth can bring value to the organisation.
Cost-Control
Competing effectively in the mining industry requires natural resource and cost advantages. But finding natural resources is a matter of chance than choice, and having a cost advantage is about competent management. We must therefore instil cost consciousness and make every cent count.
Cost advantages are derived from technological innovation, management optimisation, and effective supervision mechanisms. We are receptive and responsive, and we measure ourselves against the best companies in the sector, try to do better and become the industry pacesetter.
Focusing on professional strength is key. Staying focused and leveraging our area of specialisation is the only way to establish influence and appreciate the workings of cost control.
Continuous improvement
We must maintain the ability to self-examine, to defy convention, and to constantly adopt best practices, no matter who or where from.
Good goals are never easy to achieve, but they push an entity to excel and reward individuals with a sense of accomplishment.
We must be able to adapt, self-critique, and keep an open mind.
Achievement-sharing
We are willing to share our outcomes with everyone, outcomes that are created with our proprietary efforts, that add value, and that embody the values of equity and equality.
Our sharing of outcomes hinges on sustainable development, and sustainability is determined by our successfully entrenching meritocracy, strengthening cost control and achieving continued improvement.
We do not only share material benefits; we also strive to help our people reach their personal goals and actualise their professional self-worth.
We will continue to share our development outcomes with the world at large.
BUSINESS OPERATIONS
Copper and cobalt mine in the DRC
CMOC is the second largest cobalt producer and a leading copper producer in the world. Covering an area of over 1,500 square kilometers, our Tenke Fungurume Mine (TFM) in the DRC specializes in exploration, mining, extraction, processing, and sales of copper and cobalt, boasting a complete set of techniques and processes for all stages from mining to processing. It mainly produces copper cathode and cobalt hydroxide. In 2019, 177,956 tonnes of copper and 16,098 tonnes of cobalt were produced.
Recent highlights:
Covering a mining concession of over 1,500 square kilometers, TFM is one of the highest grade copper and cobalt mines globally with great prospects for resource exploration and development;
The integration of the new management revitalizes the business by bringing "Chinese efficiency and Chinese elements". TFM has been steadily improving its operations through management optimization and technological innovation;

Benefits from cost and efficiency programs start to show: the total cash cost decreased by US $ 130 million, of which the unit cost of mining was down 29%, the plant unit cost down 7%, and the administrative cost down 11%.
Advancing steadily, the 10K expansion project is expected to commission in 4Q2020. The project will allow the copper output to increase to more than 200,000 tons after a period of ramping up.

Message from Chairman
Dear Shareholders,

Thank you for your continued care, support, and trust in CMOC.
2021 marks an extraordinary year, as the pandemic brought great uncertainty to the world economy and no individual nor company could survive this global disaster alone. Despite demanding challenges, we managed to deliver satisfactory business results while protecting health of our employees and maintaining stable operations based on our strengths in global logistics network and production automation and our defenses built against COVID-19 in both pandemic prevention and control and in provision of supplies.
Financial highlights – In 2021, CMOC achieved an operating revenue of RMB173.863 billion, representing a year-on-year increase of 53.89%; the net profit attributable to the parent company was RMB5.106 billion, representing a year-on-year increase of 119.26%; the net profit attributable to the Company after non-recurring items amounted to RMB4.103 billion, representing a year-on-year increase of 276.24%. The total revenue and net profit reached a record high. All core business units maintained stable operation amid the pandemic, recording a significant drop in frequency of safety incident and new highs in output of key products, while IXM realized best-ever performance, a demonstration of the success that we will continue to achieve via the path of the new “mining + trading” business model.
More importantly, we also consolidated the groundwork to look ahead to the future. The “5233” management concept was practiced, the organizational upgrading and cultural reshaping were basically completed, and headquarter functions were further improved. We rebuilt the management processes and established a global management framework. Digitalization programs were rolled out to support the global business. All these helped lay a solid foundation for our growth going forward.
The pandemic itself is a trigger for people to rethink the relationship between humanity and the world, as well as for us to consider more broadly about the nature of the mining industry and our core competitiveness. Facing the new business environment and technology solutions, the traditional mining industry has been endowed with new connotations. Based on the history of the Company’s development and our understanding of the industry, we have officially updated the Company’s vision to “highly respected, modern, and world-class resources company” in accordance with the standards of international first-class mining companies.
Being “respected” represents our aspiration and pursuit that could be interpreted from three dimensions:
First is commercial success. This is the reason and purpose of the incorporation of any business entity like CMOC. To ride the tide of the new energy revolution, we will increase our production capacity and increase mineral resources and ore reserves to maintain industry-leading profitability. Sustained commercial success is the cornerstone to enhance our influence in the

industry and consolidate our leading position in the global supply of battery metals and EV raw materials, enabling us to play an important role in the global energy transition.

Second is all-round people development. We are committed to becoming an international company of excellence that upholds a corporate culture which makes our employees happy and proud and empowers them to realize their value and a successful and exciting career at CMOC.

Third is best practice in sustainability. We exercise the strictest safety, environmental, and social standards and cherish the natural resources to achieve sustainable development and maximum value for all stakeholders.

Being “modern” indicates our methodology and approach to business operations. This is a key trait that distinguishes us from traditional mining companies and requires outperformance in three aspects:

First is modern mine production. In line with the latest round of industrial revolution, we vigorously promote digital and intelligent mine development to modernize mining, beneficiation, and smelting processes. In addition, we refine mine production and improve resource utilization efficiency. Our ambition is to achieve harmonised development of the natural resources, by protecting natural environment and the communities that host our business.

Second is leverage the financial market and financial tools to avoid risks and gain profits based on a healthy balance sheet. The mining industry is quasi-financial, thus the ability to employ financial tools is a core competence of mining companies and is an advantage that we have already demonstrated in our track record. We will continue to leverage this advantage to support growth of the mining business. We will focus on balance sheet and prioritize liquidity management with a clear mind in the context of the cyclical nature of the industry.

Third is greater synergy of mining and trading. We will further leverage the synergy between upstream mines and IXM’s metal trading by taking advantage of IXM’s global network and persistently explore the potential of the “mining + trading” business model. Being “world-class” is our goal and will be the natural course of development should we keep on the right track. This necessitates a strong presence in the international mining arena and commercial success in a free and open economic system as a mature and confident player. In addition to world-class resources, industry-leading profitability, and pricing power over important resources, our teams, organizational structure, operational efficiency, corporate culture, and branding also need to be competitive by international standard. We are also dedicated to playing a leading role in new energy metals such as copper, cobalt and nickel and specialty metals such as molybdenum, tungsten, and niobium. We are keenly aware that to realize our vision, we need to be down-to-earth and take one step at a time. Therefore, we have formulated a “three-step” development path, of which the first step is to “lay a foundation” of low cost and high efficiency. Through the organization upgrade and the establishment of global control system, we will improve mechanism, attract mining elites for business operation and future expansion. The second step is to “ramp up” with multiplying production capacity. With the improvement of production capacity, the staff team has been tempered in the construction of world-class projects; with modern governance methods, our subsidiaries have been effectively controlled; with clear responsibilities and rights, and clear boundaries, the level of global governance has been raised in an all-round way. The third step is to achieve “stellar growth” towards being a world-class company, to improve our business size and cash flows to a new level and to build our talent team and increase our project reserves to meet new requirements; focusing on key regions and

categories and seeking great improvement to achieve our vision in accordance with our strategic goals. Today, we are at the crunch time to transit from step 1 to step 2. As 2022 is featured as a year of construction, we will speed up the construction of two world-class mines in the DRC to maximize resources value and lay a solid foundation for the Company to achieve leapfrog development. We are also aware that culture can be a driving force for productivity and a flexible value network bonding individuals with each other and with organizations. A healthy corporate culture can spark productive cooperation among excellent talents. After more than a year of discussion, the framework of our corporate culture has taken shape. Based on our history, this framework is built as an active response to the ever-changing environment and challenges ahead; it is an important guideline for business units worldwide to practice in their operations, in establishing rules and regulations, setting up code of conduct, fulfilling social responsibilities, and promoting brand image. It is a guidance for all employees to embrace and follow and a banner to unify thoughts, reach consensus, and motive and inspire employees. We believe that supported by such corporate culture, we will be ushered in a promising future to build our strongest moat. The world is undergoing profound changes and we are confident that we will become a respected, modern, and world[1]class resources company amid the global industrial and energy revolutions. The worldwide economy is gaining momentum to recover from the subsiding COVID-19 impacts. We believe that we could weather any crisis and challenges as long as we stay true to our aspiration, follow the rules, adapt ourselves to changes, and continue to create value for all stakeholders. The best way to predict the future is to create it! In this era of greatness, we rise to the challenges and remain resolute in pursuing our goals and vision by keeping the path of high-quality growth. We will live up to our mission and will continue to deliver results to maximize returns for shareholders!

Sun Ruiwen
CEO

SUSTAINABLE DEVELOPMENT GOALS

1.no poverty
2.zero hunger
3.good health and well-being
4.quality education
5.gendere quality
6.clean water and sanitation
7.affordable and clean energy
8.decent work and economic growth
9.industry, innovation and infrastructure
10.reduced inequalities
11.sustainable cities and communities
12.responsible consumption and production
13.climate action
14.life below water
15.life on land
16.peace, justice and strong institutions
17.partnerships for the goals

In 2022, CMOC continued to implement its Environmental Policy, which is applicable to the entire group. The environment is one of the most prominent ESG issues influencing CMOC, covering climate change, greenhouse gas emissions, biodiversity, tailings management, energy, water management, air quality, and mine closure planning and rehabilitation. As a global mining company, CMOC must manage these issues while meeting the challenges of the different climatic, geographical, ecological, and human environments where we operate, as well as the changing and evolving expectations of stakeholders.

CMOC is committed to complying with environmental laws and regulations applicable to our operations. We manage the extent of our environmental impact through risk-based approaches to material issues and adherence to standards.

We also invest in a range of projects to raise production efficiency and reduce our environmental impact. In 2022, CMOC spent around RMB 379 million on environmental protection.

All of our operations maintain mature Environmental Management Systems (EMS) certified to ISO 14001 standards. These management systems are independently audited at each operating site for recertification and include required training of all employees and contractors on environmental objectives and procedures.

CMOC is committed to gender equality. We treat male and female employees equally, safeguard the health, safety, and wellbeing of all employees, promote women's career advancement by enhancing their education and training, and raise community awareness through community programs and campaigns. Across all our global operations, we support and encourage innovations and efforts to promote equality and diversity, and believe that role models are a positive force for change.

In the DRC, our TFM operation supports the Women in Mining – DRC (WIM DRC) initiative, which aims to end all forms of discrimination against women in the DRC's mining sector and promotes the inclusion of gender factor in the DRC's national development strategy. Since 2019, TFM employee Dorothée Masele has served as President of the WIM DRC.

In accordance with the Employment Policy, to broaden firm-wide internal exchanges, we operate a differentiated talent development program that provides our employees with in-house training, rotation across mines, incentive-based development plans, and internal mobility channels based on recruiting, internal competitive selection, and referral. Guided by people-oriented principles and the strategy of the Group, we offer multiple paths for career development with space, tools, and opportunities for growth.

In 2022, 90.3% of CMOC's workforce received training, with each employee receiving an average of 22.7 hours of training.

Thriving Communities

CMOC's Community Policy is applicable to all of the Group's operations. We are committed to conducting activities in a manner that promotes positive and open relationships with local communities and supports the achievement of the United Nations Sustainable Development Goals (SDGs). We continue our engagement with and investment in communities affected by our mining operations, using multiple channels to collect their feedback and concerns. We develop infrastructure, support health, safety, and education efforts, and provide local employment and business development opportunities. Our goal is to build such relationships at all levels of community, from local to national, by making long-term positive contributions and effectively mitigating adverse impacts from our operations. Stakeholder relations across the international

portfolio are guided by community development plans, which are formulated after evaluating the expectations and needs of communities. At our NPM, Brazilian, and DRC operations, stakeholder communications, social risk and impact management, community priorities, and community investment plans guide our external engagement efforts. At our Chinese site, the national priorities of the government also play a role in driving community relations and associated investments. In all cases, stakeholder engagement is based on mutual trust, transparent dialog, and the effective mitigation of impact coupled with the enhancement of positive effects from mine operations. CMOC understands that these aspects are material to the success of our mining business.

Community relations programs at CMOC's international sites are guided by local laws and regulations and seek alignment with international good practice frameworks such as the Performance Standard 5 of the International Finance Corporation and Risk Readiness Assessment (RRA) of the Responsible Minerals Initiative. Social engagement and community investment activities may also be governed by commitments made through the process of environmental and social impact assessments or their equivalents under national regulations.

CMOC's investment approach is based on priority community needs and includes direct investment, contributions to community foundations, and participation in government as well as private sector-led initiatives. Our community investments are also centered around the United Nations Sustainable Development Goals (SDGs), with a particular focus on goals 1 (No poverty), 2 (Zero hunger), 3 (Good health and well-being), 4 (Quality education), 5 (Gender equality), 6 (Clean water and sanitation), 7 (Affordable and clean energy), 8 (Decent work and economic growth), and 9 (Industry, innovation, and infrastructure). Investments are typically made in the broad categories of education, healthcare, economic development, infrastructure, the environment, and resettlement.

In 2022, CMOC invested a total of RMB 290.4 million (eq. US$43.5 million) in community projects.

2.SINOHYDRO Corporation Limited

Founded in the early 1950s, SINOHYDRO is originally well known as China’s first brand in hydropower construction, responsible for 65% of the large- and medium-scale hydropower stations in the country. However, after 60 years of expansion and development, SINOHYDRO has become a global enterprise, running diversified businesses from water conservancy and hydropower construction to project financing, design, implementation and operation in almost all kinds of infrastructures such as power, transportation, civil work, mining and real estate.

SINOHYDRO is today a key brand of POWERCHINA — ranked No 253 among the Fortune Global 500. ENR ranks POWERCHINA No 11 in the Top International Contractors (2015)

SINOHYDRO Corporation limited the international flagship of POWERCHINA has, 5 regional offices abroad in Asia/Pacific, Africa, America, Eurasia and West Asia/North Africa to supervise the market development of 116 overseas offices in over 87 countries.

The dynamic system ensures that SINOHYDRO can provide clients with best-quality one-stop service from project consultancy, financing, survey, design and engineering to construction, fabrication, installation and operation.

The recent decade has witnessed SINOHYDRO' s rapid growth in business revenue, asset volume and asset structure diversification. Currently, SINOHYDRO has 524 international projects in more than 74 countries, with total contract value of nearly USD 42.50 billion.

SINOHYDRO has a strong corporate cultural identity based on harmony, cohesion and trust, with particular emphases on the global values of good governance, quality, safety, environment protection and social responsibility. Their projects promote local economies and improve people’s lives.

3.Electric Power Construction Corporation

SEPCO Electric Power Construction Corporation (hereinafter referred to as SEPCO) was established in 1952, and is a wholly-owned subsidiary of Power Construction Corporation of China (Power China). SEPCO has total assets of 1.7 billion USD. And it has more than 20 qualifications in China, including Level I general construction contracting for power, architecture, petrochemical (the only in the whole Power China group) and mechanical & electrical projects, etc. SEPCO has the integrated service capabilities of the entire industrial chain of power, oil & gas and petrochemical, renewable energy, energy conservation and environmental protection, and infrastructure sector, including project planning, investment and financing, survey and design, complete equipment supply, construction and installation, commissioning, operation and maintenance.

The company has more than 4000 professional employees and construction resources covering the world. It has 24 subsidiary companies including Power China Sichuan and Power China Ningxia, as well as specialized companies in renewable energy, energy conservation and environmental protection, infrastructure. In recent years, SEPCO has deeply implemented the overall development strategy of "global development, concentric diversification, innovative and upgraded, and international first-class", and focused on building a world-class energy investment and construction company with "quality and benefit type, innovative and ecological, global layout, professional operation, and integrated development". After years of development and practice, a series of SEPCO's corporate cultures have been formed, with the mission of "make customers successful, make employees happy, and contribute to the society", the core values of "integrity, responsibility, openness, and win-win", and the vision of "world leading and world renowned". SEPCO will continue to create value-added benefits for customers and realize their good wishes.

Honesty wins customers, service creates value, and capability creates the future. SEPCO is willing to work with partners all over the world to scientifically grasp the new development stage, firmly implement the new development concept, actively build a new development pattern, inject more energy into the recovery and development of the world economy, and create a better future!
SEPCO：Originated from the East, dedicate light to the world.

Enterprise strategy

During the 14th Five-Year Plan period, by analyzing the internal and external development situation and combining the current situation of the company's resource capacity, the company has formed the "1244" development strategy. The outline of the strategic plan is described as

follows:
Guiding Ideology：
Guided by Xi Jinping's socialism ideology with Chinese characteristics in the new era, we should comprehensively grasp the new development stage, conscientiously implement the new development concept, actively integrate into the new development pattern, adhere to the "1244" overall strategy, anchor the "1234567" planning objectives, strive for the overall realization of the economic indicators at the end of 14th Five-Year plan, and create a new SEPCO with high quality development. Strive to build a world-class, "two types and three modernizations "energy construction investment company.
Strategic positioning "Two types and Three modernizations"：
Build an energy construction investment company with "quality and efficiency, innovation and ecology, global layout, professional operation and integrated development" “ 1244 ” General strategy"1244"：
One vision Two major industries Four major businesses Four principles
World-class and World-famous.
Take the initiative to benchmark with first-class enterprises in domestic and abroad, reach the world-class level in business development, investment and financing, project performance, operation management, risk control, scientific and technological innovation, team management, brand image and other aspects, and strive to become a world-class energy construction and investment company.
Planning Goals：
Achieve the goal of "1234567" and create a new SEPCO. With the goal of establishing a world-class energy construction investment company, we will strive to achieve an annual operating income of 20 billion yuan, a total profit of 300 million yuan, a newly signed contract amount of 40 billion yuan, a labor productivity of no less than 500,000 yuan / person, an international business accounting for no less than 60% and non-traditional business accounting for more than 70% by the end of the 14th five year plan, Strive to develop into a comprehensive double super grade enterprise in the power sector of the group company, a leading enterprise in the international operation of the group company, and the backbone of the the Belt and Road Initiative. It will stimulate new momentum, renew vitality, and create new SEPCO.
Qualifications
SEPCO has more than 20 qualifications in China, including Level I general construction contracting for power, architecture, petrochemical (the only in the whole Power China group) and mechanical & electrical projects, and Level II general construction contracting for municipal public works, water resource and hydro power projects, etc.
Construction Industry Enterprise Qualification Certificate
Construction Industry Enterprise Qualification Certificate
Construction Industry Enterprise Qualification Certificate
Enterprise Honor
National Level Award
National Excellent Construction Enterprise for Electricity Construction
Awarded "Chinese National High Quality Engineering Prize" for 18 trophies
Awarded "Luban Prize" for 16 trophies
International Award

ENR Top 250 Global Contractors" for 71 consecutive years with the highest ranked 67;

♦ Saudi Aramco MGS Project Phase I was awa ｢ded "CII Implementation Award" by Construction Industry Institute from U.S., MGS Project Phase II was awarded "Saudi Excellent Chinese Enterprise Project" by the Embassy of the People's Republic of China in the Kingdom of Saudi Arabia.

♦ India KMPCL 6 x 600MW Coal Fired Power Plant project was awarded "Remarkable Projects" prize

"Best Construction Projects" prize, and "Outstanding Concrete Structure" prize in India.

China Daya Bay 2x900MW Nuclear Power Plant project was awarded "Plant of the Year" by POWER magazine from U.S.

Listed in ENR 11 consecutive years

Listed in ENR 11 consecutive years

President address

For many years, SEPCO Electric Power Construction Corporation has always adhered to the development concept of openness, integrity, innovation and pragmatism, and its mission is to make customers succeed, make employees happy, and contribute to the society. With the care and support of partners around the world, it has now developed into an international engineering group integrating the entire industrial chain of investment and financing, EPC, operation and maintenance, in the fields of power, oil&gas and petrochemicals, renewable energy, energy conservation and environmental protection, and infrastructure.

The initial heart is like a rock, and it will grow stronger over time. During the great changes in the world unseen in a century, SEPCO is willing to work with partners from all circles around the world to scientifically grasp the new development opportunities, firmly implement the new development concept, and actively build a new development pattern. With a global perspective, national height, industrial perspective, and corporate position, we create opportunities in opening up, we solve problems through cooperation, we inject more positive energy into the recovery and development of the world economy, build a community with a shared future for mankind, and create a better future!

Development history

In 1952, the Electric Power Bureau of central Shandong area established a local electric power engineering department to be responsible for the demolition, installation and maintenance of power generator sets in Shandong Province. SEPCO originated from then.

In 1958, the Shandong Electric Power Bureau established a capital construction company called Shandong Electric Power Bureau Capital Construction Company, and set up the No.2 and No.3 construction department, design institutes, manufacturing and repair factory, and transmission line equipment factory under the company to be responsible for the construction of power projects in Shandong Province.

In 1985, various construction department under Shandong Electric Power Bureau Capital Construction Company were restructured into legal entities and expanded in scale, successively establishing SEPCO1, SEPCO2, SEPCO3, transmission & distribution engineering company, and nuclear power engineering company.

In 1992,Shandong Electric Power Bureau Capital Construction Company changed its name to SEPCO Electric Power Construction Corporation (SEPCO for short).

In 1998, SEPCO Nuclear Power Construction Group was established with SEPCO as the core, and it has seven wholly-owned subsidiaries including Shandong Electric Power Engineering Consulting Institute (now SDEPCI), SEPCO1, Shandong Electric Power Transmission and Distribution Engineering Company, Shenzhen Shandong Nuclear Power Engineering Company, Shandong Electric Power Pipeline Engineering Company, etc.

In 2011, the State-owned Assets Supervision and Administration Commission of the State Council established Power Construction Corporation of China (Power China), and SEPCO and its affiliated companies were incorporated under Power China.

In 2016,Power China successively completed the reorganization of SEPCO's asset relationship, and designated Ningxia Electric Power Engineering Company, Sichuan Electric Power Engineering No.2 Company, and Sichuan Electric Power Engineering No.3 Company as wholly-owned subsidiaries of SEPCO, and all the SEPCO's original subsidiaries were transferred out from SEPCO.

In 2017, SEPCO and its affiliated companies successively completed the corporate restructuring. The affiliated company Ningxia Electric Power Engineering Company was renamed Power China Ningxia Engineering Corporation Limited, and Sichuan Electric Power Engineering No.2 and No.3 Company were further integrated and renamed Power China Sichuan Engineering Corporation Limited.

4.SEPCOⅢ

SEPCOⅢ Electric Power Construction Co, Ltd. affiliated to POWERCHINA, is a global engineering corporation that specializes in the construction of various types of power plants, including thermal power, saline water conversion, infrastructure, concentrating solar power, photovoltaic, wind power, nuclear power, biomass power and transformer substation. SEPCOIII's main operating models are EPCO, EPC, BOT, BOO and PMC and can provide clients with a full range of services including project investment and financing, project planning, design consultation, equipment procurement and supervision, equipment shipping, construction and installation, commissioning, operation and maintenance. SEPCOⅢ has the state top grade qualification of the power general contracting, the state A grade qualification of power engineering.

Since started business in 1985, SEPCOIll adhere to be “A Chinese Global Partner for the World", go abroad and grow stronger overseas. SEPCOIll's customer base has spread throughout the world to 28 countries in Middle East,South Asia, Southeast Asia and Europe, Africa, as constructed a variety of power plants with a single unit ranging from 12MW to 1000MW for a total installed capacity of 107700MW. SEPCOII has Constructed a number of large-scale saw water desalination projects, total daily water volume of the project is 4 million cubic meters. ranking first in the world. SEPCOIII has been awarded the title of "Advanced Collective of Central Enterprises" and "National Labor Award", and awarded National High Quality Engineering-Gold Award for 7 times and China's Luban Prize for 10 times.

SEPCOIll is a national-level high-tech enterprise, has provincial-level enterprise technology center. SEPCOIll bases on the international EPC projects, strives to develop engineering technology, lucubrates sea water desalination & solar power generation technology and the exploitation & execution of renewable energy projects, and masters the core technology of sea water treatment & low-carbon energy in field of high-tech &environmental protection.

SEPCOⅢ customers spread throughout the world, such as Saudi Arabia, Oman, Jordan, Iraq, Kuwait, Bahrain, UAE, Morocco, Egypt, Nigeria, Ghana, South Africa, India,Pakistan,

Uzbekistan,Bangladesh,Turkey, Myanmar, Indonesia and Singapore, etc. Presently the total contract value of overseas executing projects exceeds 35.86 billion US dollars.

SEPCO Ⅲ maintains a good cooperation relationship with many electric power equipment manufacturers, design institutes, and financial institutions both in China and abroad. Many foreign ambassadors in China regard SEPCOIII as the most reliable Chinese electric power construction company.

SEPCO Ⅲ is continuously pursuing its mission of "To build excellent projects, fulfill clients' expectations. highlight enterprise value and promote the welfare of society", adhere to the enterprise vision of "To be an incorporate enterprise group of investment, construction and operation, to play the leading role in global engineering EPC field" and its core value that is "Integrity, Innovation, Efficiency and Responsibility". In pursuit of our philosophy of cooperation based on "Success Through Cooperation", SEPCOIII sincerely welcomes partners from all over the world to work together to achieve our shared mission and goals.

Adhering to the concept of "Success through Cooperation' SEPCOⅢ devote itself to buiding the project with the best quality and the economic and technical indicators within the shortest period. Promoting international production capacity cooperation. SEPCO Ⅲ has held Management Seminar on international EPC power Plant Projects for five year and enjoys the fullest respect.

SEPCO Ⅲ engaged in fulfilling social responsibilities in terms of energy-saving and emission-reduction. engineering design optimization and the pursuit of an integrated value maximization of economic,social and environment. Keen on localization strategy,SEPCO Ⅲ provides training for local staff and actively applies new technologies and new crafts to promote the efficiency and prosperiy of local society. SEPCOⅢ also pays great attention to contributing public welfare activities at home and abroad, participating in charities and supporting cultural and educational and health services. In 2009, SEPCOⅢ received the Social Responsibility Award for Chinese international Contractors.

5.Harbin Electric Corporation

Harbin Electric Corporation (hereinafter referred to as HE) is evolved from six projects of China's 156 key construction projects aided by former USSR during the period of China's first Five-Year Plan. Established by reorganizing Harbin's Three Power Factories (Harbin Boiler Co., Ltd, Harbin Electric Machinery Co., Ltd, Harbin Turbine Co, Ltd), it is China's earliest R&D and manufacturing base of power generation equipment. Now HE has become one of the 52 state-owned backbone enterprises that concern national security and the lifelines of the national economy.

As the 'eldest son' of China's equipment manufacturing industry, HE has established numerous monuments throughout the national development history of power generation equipment. In the past 60 years, HE has been dedicating itself to the prosperity and development of China's equipment manufacturing industry. With great efforts, it has developed a series of leading products that ranges from nuclear power, hydropower, coal power, gas power, ships power plants and electric-driven devices to turnkey power plant projects, in which the core technical competences have reached the world's advanced level. HE now boasts a national enterprise technical center, 3 national engineering research centers (National Engineering Research Center of Hydropower Equipment, National Engineering Research Center of Power Generation Equipment, and National Engineering Research Center of Explosion-Proof Electric

Machines), 2 national key laboratories (National Key Laboratory of Hydro-Electric Power, National Key Laboratory of High-Efficiency and Clean Coal-Fired Power Plant Boiler), 4 postdoctoral workstations and 2 academician workstations. By the end of 2016, HE has obtained 1,934 patents in force, including 386 patents for invention.

By the end of 2016, HE’s registered capital was RMB 1.99 billion and its total assets was RMB 72 billion. It has over 25.5 thousand employees, of which over 10,000 were professional technicians (including 1 academician, 4 talents listed in Thousand Talent Program and 122 talents enjoying preferential treatments of governmental allowances). HE has manufactured power generation equipment with the installed capacity up to 390 million KW, provided equipment for over 500 power plants nationwide and worldwide, and exported its products to over 40 countries and regions in Asia, Africa, European and South America.

Social Responsibility

Our outlook on social responsibility: Dedicating to environmental protection and creating a big warm family.

Explanation

Building HE into an environment-friendly enterprise and a big harmonious and sweet family; pursuing amicable co-existence among people, between man and nature, and between man and the society; building a good atmosphere of mutual understanding, trust and care internally and close cooperation between the natural environment and the human environment externally.

HE continuously carries out management of and publicity to social responsibility, and sticks to the “whole-process, all-round and total-member principle” to advance social responsibility drive. In responsibility practice, HE continuously summarizes and extracts excellent management cases and typical experiences of corporate social responsibility, and establishes and perfects relevant responsibility systems, to in-depth promote whole-process coverage, all-round integration and total-member participation in management of corporate social responsibility, and make active contributions to sustained and stable development of the enterprise and to social harmony and stability.

Whole-process coverage:

Integrating the requirements for management of social responsibility into the production and operation system.

Integrating the requirements for management of social responsibility into assets and economic operation management.

Integrating the requirements for management of social responsibility into assets and economic operation management.

All-round integration:

Reshaping enterprise mission and enterprise values.

Formulating and implementing sustainable development strategy.

Optimizing governance mechanism and establishing social responsibility advancement system.

Establishing and perfecting stakeholder participation mechanism.

Building comprehensive value creation performance appraisal system.

All employee participation:

Leaders firstly promise to be models in advancing social responsibility management.

Conducting social responsibility training of all members.

Creating good institutional environment and cultural atmosphere in the system, institution and resources aspects.

Culture

Vision of HE

To build a power carrier of equipment manufacturing industry in the world.

Interpretation:

HE people aim high and never rest on the achievements made. They make up their minds to keep making great achievements, aim to be a world leading equipment manufacturing enterprise and build HE into a power carrier influential and competitive internationally.

Mission of HE

Bearing hopes of the national industry, highlighting glamour of China power.

Interpretation:

Bearing hopes of the national industry: HE people are brave in shouldering burdens and dedicated, not only to completing various major national tasks, but also to inspiring themselves, keeping advancing and creating miracles one after another, so as to make its contributions to the revitalization of national industry and achieve the great rejuvenation of the Chinese nation.

Purpose of HE

To provide power for the world and bring brightness to human being.

Interpretation:

To provide power for the world: HE, farsighted, aims to “ build power carrier of equipment manufacturing industry in the world" and pursues leading technologies to provide the world with quality power products and services and create optimal economic and social benefits, to boost national economic development and make its contributions to world economic growth.

Objectives of HE

Best in China, leading in the world.

Interpretation:

Best in China: HE, with the mission of “bearing hopes of the national industry, highlighting glamour of China power”, always makes efforts to be leading domestically and pilot in China’s equipment manufacturing sector. With these efforts, it has become a flagship in the sector.

Leading in the world: HE never rests on what it has made, but always keeps pioneering, to constantly lift its strength and comprehensive competitiveness, so as to spread the brand of HE around the world and become a leading enterprise in equipment manufacturing industry in the world.

Core philosophy of HE

People-oriented to develop, strength-based to win.

Interpretation:

People-oriented to develop: HE practices people-oriented management and focuses on employees’ livelihood, enhancing cohesion and teamwork spirit. It is user-oriented to meet users’ needs, winning users’ trust and loyalty. It is the public-oriented and loves the society, winning praises and support of the public. Only concerted efforts can generate great power and create an evergreen cause.

Strength-based to win: The market follows the law of survival of the fittest, and only the winner wins. HE people, struggling, pioneering, help HE develop its strength, build its brand, win its way in the market and create bright future with the first-rate employees, technologies, quality and

management.

Core values of HE

Concerted efforts, dedicated services.

Interpretation:

Concerted efforts: Unity makes power. HE members work and act together and in a concerted manner, to jointly develop HE and boost the great cause of HE.

Dedicated services: Cohesion is the source of hope. HE members, with dedicated services and teamwork, make constant efforts for common development and brilliance.

Spirits of HE

Innovative endlessly, powerful forever.

Interpretation:

Innovative endlessly: Innovation is the soul in and the power source for enterprise development. HE members, struggling, full of passion and dream, inspire them with innovation, integrate innovation into their character, and sharpen their vision through innovation, to keep advancing and creating miracles.

Powerful forever: Lofty sense of mission gives birth to inexhaustible power, and the power triggers a stream of vitality. HE members follow the mission of rendering service to the motherland and revitalizing HE, to give play to their potentials, pioneer with passion and make excellence.

Business Philosophy of HE

Market oriented, brand leading, honesty boosting.

Interpretation:

Market oriented: Enhancing market idea, sticking to be market-oriented, and aiming at meeting users’ demands, to seize potential market opportunity and keep right business direction.

Brand leading: Strengthening brand awareness to win trust and support of customers with good brand image and keep advantageous in competition.

Honesty boosting: Following the way of honesty operation to serve users sincerely and win market share with credit, so as to keep sustained, healthy development of the enterprise.

R&D

HE boasts a national enterprise technical center, 3 national engineering research centers (National Engineering Research Center of Hydropower Equipment, National Engineering Research Center of Power Generation Equipment, and National Engineering Research Center of Explosion-Proof Electric Machines), 2 national key laboratories (National Key Laboratory of Hydro-Electric Power, National Key Laboratory of High-Efficiency and Clean Coal-Fired Power Plant Boiler), 4 postdoctoral workstations and 2 academician workstations. In March 2011, it was listed as one of the 3rd batch of innovative enterprises.

Career

HE practices the “45411” talent strategy, namely: “4”:

Deeply advancing professional, occupational, market-oriented and internationally-based development of talents, and striving to make skill level professional, work accomplishment occupational, system and mechanism market-oriented and idea and goal internationally-based.

“5”:

Doing its best to build 5 teams: 1. Investor representative team, 2. Operation and management

talent team, 3. Party-masses worker team, 4. Sci-tech talent team, and 5. High-skill talent team.
“4”:
Focusing on innovation in 4 mechanisms, namely talent training and development mechanism, talent selection and appointment mechanism, talent appraisal and evaluation mechanism, talent incentive and guarantee mechanism.
“1”:
Closely centering on 1 center, to greatly build a good environment favorable for development of talents; closely centering on HE’s strategic development goals to establish strategy-oriented talent work system, actively construct a good environment favorable for talent development and spare no efforts to build a high-quality talent team with first-rate professional accomplishment, business skill, work style and position performance, so as to prepare powerful talent support and intelligence guarantee for achieving HE’s strategic development goals.
“1”:
Comprehensively implementing a talent matching project. The Corporate will strive to cultivate more than 1 academician of the Chinese Academy of Engineering or industrial topnotch technical experts equivalent to academician level in 5-10 years; train more than 10 industrial excellent entrepreneurs able to lead the enterprise into leading groups in the sector domestically and internationally; cultivate more than 10 chief technical experts of HE with comprehensive and profound attainments, remarkable performance and achievements and prestigious in the industry, introduce more than 10 overseas leading talents, and foster about 50 excellent leaders and 155 corporation-level experts as well as thousands of backbone talents in management, science and technology and international vision.
6.China National Machinery Import & Export Corporation
China National Machinery Import & Export Corporation (hereinafter referred to as "CMC") was established in 1950, and it has been holding a high reputation for its international operation which is also considered as its core competitiveness and company philosophy. So far, its accumulated international business volume has exceeded 130 billion US dollars.

Since 1950, CMC has been continuously importing high-end mechanical and electrical equipment, technology and services overseas into China, and made important contributions to the development of Chinese economy, the establishment and improvement of national industrial system, eradication of poverty and amelioration of people's livelihood.

Since 1978, CMC has been exporting domestic sophisticated mechanical and electrical products, industrial equipment as well as “turn-key” infrastructure projects overseas, contributing to the economic and social development of developing countries and emerging markets.

Since 2008, CMC has entered the low-carbon environmental protection field focusing on green energy. At present, specializing in the green and low-carbon industry has become the main development direction of the company. And CMC would spare no effort to achieve “emission peak” and “carbon neutrality”, trying to make more contributions to the entire humankind.

CMC's Responsibilities: Quality, Safety, Health, Environment, Society and Corporate Governance. CMC has set benchmarks against global corporate governance standards, proactively assumed

and fulfilled its corporate social responsibilities, and built a global connective partnership around quality, safety, health, environment, society, and corporate governance to promote local industrial development, as well as inclusive, strong and sustainable growth of where the projects are located, so as to achieve economical and social accomplishments. CMC strives to put green concepts into practice, vigorously develop green and low-carbon industries, promote clean energy construction, resource conservation and recycling, provide communities with a safe and healthy living environment, and ensure sustainable development. Moreover, CMC is always people-oriented. It respects the local culture and lifestyles, helps to achieve poverty alleviation, and promote local employment. What ' s more, it contributes to the development of local voluntary services and charities, and actively supports the development of local education, culture, medical and health care, etc., hoping to bring more benefits to the local people.

As time goes by, CMC continues to optimize and adjust its governance structure, comprehensively manage and control operational risks, continuously reform and innovate, steadily improve its strategy-execution capabilities, and gradually improve its corporate governance system.

Employees of CMC. Employees have always been the most valuable resource for CMC ' s development since its development. The concept of "sticking to people-oriented strategy to achieve a harmonious and win-win outcome" has been engraved into the company's development strategies, CMC strives to gather outstanding talents at home and abroad in order to build a market-oriented and international career development platform with its own characteristics. Besides, CMC respects different personalities, cultures, religions and safeguards the rights and interests of its employees. It provides excellent training opportunities for employees, spares no efforts to promote their all-round development as well as realize the simultaneous improvement of employees' value and corporate value. Up to now, CMC has more than 4,000 employees, of which 28% are female employees and 48% are from overseas.

CMC is currently a member of General Technology Group International Holdings Co., Ltd. It has specialized and international operating units and subsidiaries, and holds strategic investments in various industries. Up to now, CMC has established more than 40 subordinate branches at home and abroad.

7.China Machine- Building International Corporation

China National Machinery Foreign Economic and Technical Cooperation Co., Ltd. (CMIC) was established in 1980. It is a multi-functional professional and well-known international economic and technical cooperation company. With the aim of "combining industry and trade, combining technology and trade, promoting international economic and technological cooperation in the mechanical and electrical industry, and expanding my country ' s mechanical and electrical products exports", the company takes advantage of my country's mechanical industry's superior equipment, mature technology, and strong design capabilities to actively explore the

international market. Develop economic and technological cooperation with countries around the world, and have established stable economic cooperation relations with hundreds of enterprises in dozens of countries around the world, and have won a good reputation. Since its establishment, the company has been committed to contracting projects, complete equipment export and cooperative production and other businesses. It has successively provided complete equipment, electromechanical products and stand-alone equipment to nearly 100 countries in the world for more than one billion US dollars. In 1981, the company successfully obtained Pakistan The 210,000-kilowatt thermal power generating unit project contract for Gudu Power Station was the first to enter the international market for large-scale thermal power generation equipment in China, which was praised by users. The Wangqiao Sugar Plant, which is contracted by our company to squeeze 8,000 tons of sugarcane per day in Thailand, is the first large-scale sugar production plant in the world. The company has also contracted heavy machinery plants, casting and forging plants, water pump plants, agricultural machinery plants, cement plants, glass plants, etc. in Pakistan, Peru, Sri Lanka, Romania, Algeria, Sudan, Angola, Vietnam, Laos, Indonesia and Myanmar. Hydropower stations, power transmission and transformation projects, water supply projects and other projects. At the same time, the company has undertaken more than 1,000 technology import projects in my country's electromechanical industry, and imported various types of machinery and equipment worth billions of dollars. Including the introduction of 300,000-kilowatt and 600,000-kilowatt thermal power generating unit manufacturing technology projects from the United States. Cooperated with German and Japanese companies to contract the joint design and production project of 2,050mm hot strip mill, 1,900mm large slab caster, and Qinhuangdao Coal Terminal Phase II engineering equipment of my country's Baosteel Phase II project.

China National Machinery Foreign Economic and Technical Cooperation Co., Ltd. is a subsidiary of China National Machinery Industry Corporation. In 2014, it was reorganized with China Erzhong Group to become a new export platform for China Erzhong's high-end heavy equipment. The company will devote itself to international trade, international engineering contracting and international complete project services, and strive to become an economic and technical cooperation company with diverse trade cooperation channels and extensive business fields.

8.China Civil Engineering Construction Corporation (CCECC)

China Civil Engineering Construction Corporation (CCECC), formerly the Foreign Aid Bureau of the Ministry of Railways, was established in 1979 under the approval of the State Council of the People’s Republic of China and was incorporated into China Railway Construction Corporation (CRCC) in 2003 for strategic regrouping under the approval of the State-owned Assets Supervision and Administration Commission. As one of the pioneering Chinese companies entering the international market, CCECC is now developed into a large-scale state-owned enterprise with Chinese National Super Grade Qualification for railway project engineering, and has been listed among the world’s top 225 International contractors for many years in a row. CCECC has established resident offices or project management headquarters in 89 countries and regions by the end of 2016, spreading across Asia, Europe, Africa, America and Oceania with its business covering project contracting, civil engineering design & consultancy, industrial park construction,

development and operation, real estate development and property management, investment, railway operation, industrial mining, import & export, hotel management and travel services etc.

This Chinese company providing construction and engineering services is one of Nigeria's most successful Chinese-owned companies. It has been responsible for railway construction and real estate developments, among other major engineering services in the country.

Since the 1960s, CCECC has started to build China ’ s largest foreign aid project - the Tanzania-Zambia Railway. The continuous development over near 40 years enables the Group to operate in Asia, Europe, Africa, the Americas and Oceania. Up to now, the Group has established regional offices in 104 countries and regions around the world. During the implementation of the Belt and Road Initiative, CCECC has implemented or tracked projects in 79 countries that have signed cooperation documents with China on jointly building the Belt and Road.

We have been deeply involved in overseas infrastructure construction and undertaken many landmark projects setting records for Chinese contractors: the Turkey Ankara-Istanbul High-Speed Railway Project is the first overseas high-speed railway constructed by Chinese enterprises; the Abuja-Kaduna Railway in Nigeria marks the export of Chinese railway standards for the first time; the Abuja Rail Mass Transit is the first urban light rail project in West Africa; the Addis Ababa-Djibouti Railway is the first cross-border modern electrified railway in Africa.

We have expanded our business from engineering and contracting to project development and operation, transforming from a pure contractor to multiple roles such as an investor, an operator and a developer. We strive to capture valuable business opportunities and allocate production factors worldwide, and make every effort to building a multinational company with a great corporate image and strong business competitiveness.

Incorporated in 1996, CCECC Nigeria Ltd. (CCECC Nigeria), is a subsidiary of CCECC headquartered in Abuja, Nigeria. As the largest construction contractor in Nigeria and even in West Africa, CCECC Nigeria has obtained Grade-A qualifications for constructing projects of the Nigerian government. The Company has launched businesses in 29 out of 36 states across Nigeria, covering general contracting, project contracting, project management, and consulting services for railways, highways, bridges, municipal projects, housing projects, water projects, and so on.

CCECC Nigeria has successively undertaken or secured key projects in Nigeria, including repair and renovation projects of existing railways, highway projects in four towns of Cross River State, the Nigerian Railway Modernization Project, the Damaturu-Maiduguri Road Project, Abuja Rail Mass Transit (ARMT) Project (Phase I), LRMT Project (Blue Line), the Four New Terminal Buildings Project, the Nigerian Coastal Rail Project, and the Ibadan Light Rail Project (Section 1 of Phase 1), forming a business structure centering on transportation infrastructure and engineering contracting. Apart from improving the engineering contracting business, CCECC Nigeria has continuously expanded the upstream and downstream sectors of the industry chain to facilitate diversified development. The Company has contracted multiple projects, including the Athletes’ Village Project, the Nigerian Communications Commission (NCC) Building Project, the Abuja Rail Mass Transit Project (Phase I), the EMU and Traction Maintenance Depot Equipment Procurement Project, Procurement of Locomotives for Abuja-Kaduna Railway and Lagos-Ibadan Railway Project, forming the 1+N business structure covering diverse industries.

Adhering to the mission of “providing Nigeria with quality infrastructure and services,” CCECC Nigeria has pursued common development with Nigeria, winning the recognition of relevant industry associations, government departments, and private organizations in Nigeria. The

Company has also won many honors and awards, including the Outstanding Contribution Award of the Construction & Civil Engineering Senior Staff Association (CCESSA), the Best Infrastructure Construction Company of Nigeria, the Nigeria Outstanding Highway Contractor Award of the Nigerian Quality Products Council, the Best Infrastructure Contractor in Nigeria of the Nigerian Transport Association, and so on.

CCECC Nigeria Limited Corporate Social Responsibility Report(1981-2020)

Message from the Corporation

Thanks for the attention and support from all walks of life.

Since its inception in 1979, CCECC has been dedicated to providing integrated infrastructure solutions for its customers.

Presently, CCECC has developed into a large-scale group focusing on transportation infrastructure while covering design consulting, investment, railway operation and maintenance, industrial park, trade and logistics, and many other sectors.

As a multinational corporation, CCECC has established regional offices or launched businesses in 104 countries and regions around the world. While meeting local needs in infrastructure development, we have gained a deep understanding of the significance of responsible and sustainable development. We make all-out efforts to undertake high-quality and sustainable infrastructure projects, so as to create economic, social, and environmental values for local areas, facilitate high-quality

construction of the Belt and Road, and contribute to the realization of the Sustainable Development Goals (SDGs) set in the United Nations' 2030 Agenda for Sustainable Development.

Nigeria is the first overseas market that CCECC entered and is a model of the international development of CCECC. For the past near 40 years of development in Nigeria, we have embraced "sincerity, solidarity, amity and integrity" and established a favorable mechanism for interacting with local governments, employers, employees, partners, communities, and other sectors of society. We listen and respond to voices of stakeholders while developing corporate development strategies and carrying out production and operation activities. We have formed a benign development cycle featuring shared responsibilities with stakeholders, win-win cooperation, cultural prosperity, and harmonious coexistence, striving to set an exemplar of cooperation with Nigerian partners.

In the future, CCECC will stick to the principle of high standards, livelihood improvement and sustainable development, seize the tremendous opportunities created by the Belt and Road Initiative, and implement the requirements of "Quality of CRCC" and "giving priority to overseas markets." Leveraging CCECC's advanced technologies, businesses and resources, we will serve Nigeria's infrastructure development and industry upgrading, and contribute to Nigeria's economic development and social progress, as well as the well-being of Nigerians.

Zhao Dianlong

Chairman

China Civil Engineering Construction Corporation

Chen Sichang

President

China Civil Engineering Construction Corporation

Thank you for reading the first Corporate Social Responsibility Report of CCECC Nigeria Ltd.
Since entering the Nigerian market in 1981, we have actively responded to local market demands and fully deployed our resources and business strength in providing local areas with high-quality services relating to infrastructure construction, winning the trust and extensive support of our partners from all walks of life. At the moment, the Company has launched businesses in 29 states across Nigeria, covering general contracting, project contracting, project management, and consulting services for railways, highways, bridges, municipal projects, housing projects, water projects, and so on.
Following the principle of “sincerity, solidarity, amity and integrity” and adhering to the mission of “providing Nigeria with quality infrastructure and services,” CCECC Nigeria has pursued common development and growth with Nigeria and established harmonious and win-win relationships with local stakeholders.
We earnestly devote to local development. Through near 40 years of development featuring win-win cooperation, CCECC Nigeria has proven to be a truly reliable partner willing and able to support the prosperity and development of Nigeria. Sticking to pragmatic cooperation and relying on CCECC’s strong competences,
CCECC Nigeria has kept innovating in development visions and expanded areas of cooperation and development. We help Nigeria create high-quality infrastructure projects and provide quality services to local areas, and continue to deepen China-Nigeria cooperation under the Belt and Road Initiative. Insisting on localized development, CCECC Nigeria employs and trains local staff with great effort. We have established sound talent echelons, realized win-win cooperation with local suppliers and subcontractors, supported local communities in improving livelihoods, created diversified values for local areas, and established close and deep ties with local people. Upholding honest operations, we strictly abide by local laws and regulations, act in line with business ethics, and protect the ecological environment, which lays a solid foundation for the Company’s long-term and sustainable development in Nigeria.
In the future, we will remain a true friend and good partner of Nigeria to help her develop and thrive, strike a balance between the expectations and the demands of stakeholders, strive to create higher economic, social, and environmental values, and promote the high-quality development of the Belt and Road, contributing to the
sustainable economic and social development of Nigeria.
Jiang Yigao
Chairman, CCECC Nigeria Ltd
Zhang Zhichen
Managing Director, CCECC Nigeria Ltd

Outlook
At present, global economic development is facing greater uncertainties, and impacts of the COVID-19 pandemic are still spreading. Nigeria’s economic and social development is facing great challenges. Under such circumstances, CCECC Nigeria is more determined and confident to develop together with Nigeria. We will carefully analyze the impacts of these uncertainties, take earnest fulfillment of social responsibility as the foundation for solving problems and realizing steady growth, and strive to create development models that are more sustainable. We will work with government departments, employers, partners, employees, communities, and other local

stakeholders to ensure project quality and safety. We will provide technical support for the operation of projects, enhance local employment and training of local talents, and promote the transfer of advanced technologies. Through such efforts, we aim to forge ahead together with local partners, promote the upgrading and development of industries, and facilitate the improvement of local livelihood, so as to jointly build a bright future for Nigeria

Promoting Common Development with Sincere Actions

At CCECC Nigeria, we honor sincerity, friendship, equality, and mutual benefits. During the near 40 years of development in Nigeria, we have always sought solutions to leverage our business and resource strength to meet local development needs in line with the principle of sincerity, friendship, equality, and mutual benefits, so as to

promote local infrastructure development. CCECC Nigeria supports the implementation of the National Integrated Infrastructure Master Plan (NIIMP) and the Economic Recovery and Growth Plan (ERGP) for 2017-2020, and helps to create engines for local economic and social development, providing strong transportation and industrial guarantees for Nigeria to realize modern development.

Striding with Nigeria for Four Decades

Since entering Nigeria in 1981, CCECC has always adhered to the mission of “providing Nigeria with quality infrastructure and services”. Following the guidance of the Belt and Road Initiative, we responded to the needs of local governments and people in a timely manner and introduced advanced technologies to Nigeria. With these efforts, we have constructed a series of highquality projects for Nigeria and brought much convenience to local people’s transportation, life, and production, winning the trust and recognition of local governments and people.

Building a Multidimensional and Sustainable Transportation System

CCECC Nigeria engages in the construction and related services of railways, highways, urban light rails, bridges, ports, and other infrastructure projects. In the design phase, we strengthen overall planning to help Nigeria build integrated and interconnected transportation infrastructure projects and create a multidimensional transportation system, aiming to provide brand-new, punctual, safe, convenient, and economical transportation services for local citizens. We are committed to using sustainable transportation infrastructure solutions to benefit people’s work and life, laying a foundation for sustainable economic and social development in Nigeria.

Making Diversified Investments and Boosting Economic Development

With the continuous development of the market demand, economy, and society of Nigeria, we continue to upgrade our businesses.

On the basis of strengthening our main business - rail transit infrastructure construction, we continue to expand towards planning, design, operation and maintenance, and other upstream and downstream sectors, aiming to provide better integrated infrastructure services for the local area. At the same time, we have improved our development capabilities, providing engineering services for

the local area in terms of water-related projects, agricultural development, and power infrastructure, and launched and deepened the investment business, striving to make greater contributions to local economic and social development.

The Lekki Free Zone jointly invested and constructed by the Chinese Consortium in the name of CCECC-CALI (China-Africa Lekki Investment Limited), the Lagos State Government and Lekki Worldwide Investments Limited, has been highly valued

by Nigerian governments at all levels since its launch in 2006, and it is regarded as a model for free trade zones developed by China and Africa as well as a pilot project in Nigeria. Providing one-stop services for enterprises, the free trade zone has
attracted investments from a large number of companies engaging in manufacturing, trade, and logistics, creating a lot of jobs for local areas and driving Nigeria's industrialization.

Promoting Local Economy

Giving priority to manufacturing, warehousing & logistics as the leading industries in the Zone, and taking urban services and real estate development as the supporting industries, the Lekki Free Zone has attracted investments from a large number of companies, which not only fill up Nigeria's gaps in related industries but also improves local people's lives. Meanwhile, the zone has trained a multitude of local
technical talents, and promoted the development of local industries and the urbanization of the surrounding areas.

Eventually, the Lekki Free Zone will be turned into a satellite city of Lagos, a dynamic city with successful businesses, a modern coastal industrial city, and a livable city with a beautiful environment.

Boosting Infrastructure, Facilities, and Services Along the Whole Industry Chain

CCECC Nigeria extends business to the upstream and the downstream of the industry chain of infrastructure, including rolling stock supply and rail line operation, making the industry chain more comprehensive. By offering diversified business services, we facilitate the transformation and upgrading of local infrastructure. We have cooperated closely with the Nigerian government in technology transfer and training of locals to enhance the country's capacity for developing transportation infrastructure.

Strengthening Practical Cooperation and Holding Together for Shared Prosperity Building on Integrity, Putting Compliance First Ensuring High Quality

CCECC Nigeria keeps moving forward step by step, and embraces practical cooperation and high efficiency. We put quality and safety first while carrying out R&D and innovation in technology and processes. At the same time, we explore efficient and intensive management models to continuously improve our business capabilities, broaden the space for development, and enhance project quality. We strive to satisfy our customers, employers, and local people, and build high-quality projects that can withstand the test of time, advancing the cooperation between
China and Nigeria under the Belt and Road Initiative.

Building Quality Projects

As a key participant in and builder of the Belt and Road, CCECC Nigeria strictly abides by local laws and regulations, attaches great importance to project quality and safety, and upholds strict quality standards by implementing various quality control measures. Our advantages in technology, expertise, and management have enabled us to deliver a range of high-quality projects to Nigeria.

High-quality Construction

We fulfill our responsibility for quality by carrying out quality management throughout the lifecycle of projects, and strive to guarantee high quality from the source by implementing management measures, such as standard verification, realtime supervision, and quality inspection, aiming to build renowned projects. Upholding high standards in quality management,

CCECC Nigeria has won many honors for its infrastructure projects in Nigeria, and created multiple excellent projects that meet local people’s needs in daily life and production.

High-quality Operation

CCECC Nigeria has been providing operational technology support for projects including the Abuja-Kaduna Railway and the ARMT Project (Phase I). In the course of our operations, we improve the integrity and standardization of our projects, enhance local operation and maintenance capabilities, and ensure the efficiency and quality of teamwork. We guarantee the safety of operations to better meet the needs of employers, and provide safe, comfortable, and convenient passenger and cargo transportation services.

Ensuring Employee Health and Safety

We adhere to the people-oriented principle and regard safety as the prerequisite for all work. By enhancing the work safety management system and systematically carrying out safety training, we have enhanced employees’ safety capabilities, pursued the safety management goal of zero injuries and zero accidents, and obtained the certification for Occupational Health and Safety Management Systems. During the COVID-19 pandemic, we have vigorously carried out pandemic prevention and control measures, and resumed work in an orderly manner on the premise of ensuring the well-being of employees.

Strengthening Safety Management

CCECC Nigeria has formulated safety management policies and systems according to the actual conditions of projects, and strictly implemented various safety measures. In addition, the Company has hired safety inspectors to ensure that construction safety is always under control and employees’ safety and health are fully protected

Enhancing Safety Capabilities

Employees’ safety awareness and capabilities are the foundation of work safety. In order to improve such awareness and capabilities, we actively organize safety training for employees and explore different training models.

Guaranteeing Public Security

In light of potential terrorist attacks and violent threats, we actively communicate with the local military and police departments, and always monitor the construction and operation of the project in an effort to control and eliminate safety hazards.

Tightening COVID-19 Pandemic Containment

In the face of the COVID-19 pandemic, we have made every effort to fight the disease and resume work in an orderly manner. We conduct timely and comprehensive investigations and disinfection, publicize the knowledge and practice towards COVID-19 pandemic prevention, and distribute anti-pandemic supplies to our employees. Apart from providing a safe working and living environment, we also make rational arrangement for resumption of work and production

Insisting on Innovation-driven Development

Insisting on innovation-driven development, CCECC Nigeria advocates the spirit of innovation, and has introduced advanced infrastructure technologies to Nigeria. We conduct technological and process innovations based on local conditions and actively explore new models for localized management, upgrading the quality of local infrastructure and contributing to better economic development.

Technological Innovation

Based on the geographic conditions, resource endowment, and climatic and environmental

conditions of places where our projects are located, we optimize technologies and processes to solve challenges encountered during project implementation. By doing so, we can also deliver technological innovation results, thus providing strong guarantees for high-quality project construction and contributing to the upgrade of transportation infrastructure technologies in the local area.

Innovative Localized Operation

We strictly abide by local laws and regulations, and innovate in localized management models in accordance with local business practices, customs, cultures, and employees' working styles. By refining the division of labor within project teams, we have been cultivating local teams capable of independent construction, thereby increasing the proportion of local employees, improving their technical and management capabilities in a short period, and developing them into highcaliber talents in the infrastructure sector of Nigeria.

Holding Together for Shared Prosperity

We undertake responsibilities and enhance people-to-people bonds. To constantly deepen localized operation, we hire and train local staff to form teams needed for the development of local infrastructure, and expand local procurement and subcontracting through deepened cooperation with local suppliers and subcontractors. We also support livelihood improvement of local communities. While creating values and contributing to local development, we have established close ties with local people and formed a community of shared future with Nigeria

Enhancing Local Employment

During the past near 40 years of development in Nigeria, CCECC Nigeria has vigorously carried out local employment and protected the legitimate rights and interests of employees. We create a workplace of equal opportunities, respect and diversity. While offering a large number of local jobs, the effort helps promote economic development and improve local livelihood.

Hiring Local People

Attaching much importance to local employment, CCECC Nigeria continues to increase the proportion of local employees. Besides, we boost local employment to the hilt and strive to improve the skills and income of residents around the project sites. Every year, we sign employment contracts with fresh graduates from Nigerian universities, carry out induction training and follow-up appraisals for them, and train local staff with great efforts.

Guaranteeing the Rights and Interests of Employees

In strict compliance with local labor laws and regulations, we implement labor and employment systems properly, sign employment contracts with employees in accordance with the law, pay for employees' social insurances and related charges in full, and protect employees' rights on working hours and paid leaves. With a great emphasis on diversity, we work to enhance exchanges between employees from different countries and cultures, create an inclusive workplace featuring mutual assistance, and build a warm and harmonious big family of CCECC.

Conducting Systematic Talent Training

Conducting Systematic Talent Training

CCECC Nigeria puts an emphasis on the development of employees' vocational skills. We improve local engineering construction level and strengthen the transfer of operation technologies through various means, including providing vocational skills training, supporting employees in getting access to academic education, and assisting local areas in building higher

education disciplines, etc. This helps build a talent pool for construction, operation, maintenance of engineering projects in Nigeria and lays a solid foundation for future development.

Deepening Local Cooperation

CCECC Nigeria actively deepens cooperation with local companies. Working closely with local suppliers, we purchase materials and services needed by project construction locally as much as possible, and earnestly cooperate with local stakeholders from the supply chain, industry chain, and value chain, striving to stimulate the development of local businesses, commerce, and related industries, provided related training for locals, and contribute to sustainable economic and social development

Driving the Growth of Suppliers

We have established a list of qualified suppliers and subcontractors, and adjust and update the list based on the results of the annual evaluation, so as to encourage and support the development of responsible suppliers. While cooperating with local subcontractors, we continue to optimize the allocation of resources, provide training for locals, increase the proportion of projects subcontracted to local companies, and support the development of small and medium-sized partners. Since 2017, we have signed 288 subcontracting contracts with 170 Nigerian companies with a total contract value of approximately 25 billion naira (around USD 82 million)

Enhancing Local Procurement

According to the reality of the Nigerian market, we try our best to source raw materials and other products and services needed in our projects locally. In this way, we can improve the efficiency of project construction while creating greater added value for local areas. By now, the Company has established long-term partnerships with many local suppliers of bulk commodities and realized local procurement of rebar, asphalt, pyrotechnics, cement, diesel oil, auxiliary oil, and tires, etc.

Boosting Industry Upgrade

We have established a convenient and efficient transportation system by building roads, railways, terminal buildings, and other infrastructure projects, improving local transportation infrastructure. Also, we help improve local logistics services to benefit the production and life of local residents, logistics parks, and industrial development, thus accelerating the development of local industries and the service sector and drive the local economy to prosper.

Improving Local People’s Livelihood

CCECC Nigeria values communication with local communities. While developing our projects, we pay visits to local communities and tribes, and communicate the value of the projects to them. We try our best to know their needs, and actively respond to those needs. By developing community infrastructure projects and projects aimed at improving the local science, educational, cultural and health conditions, participating in local poverty alleviation, education, emergency rescues, and disaster relief, and organizing photography exhibitions, singing competitions, and other activities, we have built harmonious relationships with local communities

Enhancing Communication with Local Communities

In the process of project development, we try our best to meet the expectations and needs of local communities and pay regular visits to local residents and tribal elders living near the projects to enhance mutual trust, deepen friendship, and promote mutual development together with them.

Caring about the Development of Local Education

We care about and actively support education in the places where we carry out projects. By donating funds to school construction as well as donating stationery and sports supplies, we are helping more local children receive better education. In addition, we publicize safety knowledge to local communities based on our own business characteristics, creating a good atmosphere for safety education.

Improving Local Infrastructure

We attach importance to the urgent needs of local people for community development and improvement of livelihood. Therefore, we give full play to our expertise and resources in infrastructure construction, and spare no effort to help local villages and tribes build and repair roads, houses, street lights, drainage facilities, etc., so as to create convenience for local residents.

Building on Integrity, Putting Compliance First

Acting with honesty and being consistent is the foundation of our business. Based on the actual needs of Nigeria for infrastructure development, we operate in compliance with laws and regulations, improve the management structure and system, and integrate social responsibility concepts and requirements into the corporate management system. We uphold business ethics, protect the environment, and prevent business risks to lay the foundation for the Company’s overseas sustainable development. We strive to become a trusted partner of local stakeholders, including local governments, employers, business partners, employees, communities, etc., and create a better business and natural environment with joined hands.

Improving Corporate Governance

CCECC Nigeria strictly abides by local laws and regulations, and respects local business practices, cultures and customs. We have continuously improved the Company’s management system and obtained the three major management system certifications on quality, environment, and occupational health and safety. At the same time, we attach great importance to the expectations and needs of internal and external stakeholders in decision-making and management processes. These endeavors have laid a solid foundation for the Company’s sustainable development in Nigeria

Upholding Business Ethics

We strictly abide by the laws and regulations of Nigeria, follow international conventions, standards and guidelines while operating our business, and strongly oppose commercial bribery. On this basis, we have formulated internal compliance management rules. When cooperating with our partners, we act in accordance with contracts and protect their interests. We also sign integrity and compliance agreements with the partners to safeguard business integrity and ethics

Protecting Legitimate Rights and Interests of Customers

We adhere to open, fair and just competition, strictly abide by Nigeria’s procurement laws and regulations, and oppose commercial bribery; we sign fair and reasonable contracts with customers in accordance with industry practices, and construct and deliver the project as stipulated in contracts.

Protecting Rights of Suppliers

We have formulated the Measures for the Management of Equipment and Materials Procurement and the Measures for the Management of Equipment and Material Suppliers, as well as other measures to protect the legitimate rights and interests of suppliers and

subcontractors. We strictly implement the principle of selecting suppliers in an open, fair, competitive and merits-based way, and manage subcontractors in accordance with contracts to ensure the overall progress, safety, efficiency and quality of all projects.

Protecting the Environment

Adhering to the principle of “prioritizing prevention, integrating prevention and protection, and comprehensively addressing environmental problems”, CCECC Nigeria implements the concept of green construction, and lays emphasis on energy conservation and environmental protection. In order to promote sustainable development, we are committed to minimizing the negative impact of the projects on the local environment and protecting the green homeland of local people in the process of building eco-friendly transportation infrastructure projects.

9.China State Construction Engineering Corporation

Founded in 1982, China State Construction Engineering Corporation (hereinafter "China State Construction" and "CSCEC") is now a global investment and construction group featuring professional development and market-oriented operation.

China State Construction carries out business management through its public company - China State Construction Engineering Corporation Ltd. (stock code 601668.SH), and has eight listed companies and more than 100 subsidiaries.

In 2021, China State Construction saw its new contract value hit 3.53 trillion yuan, with a year-on-year increase of 10.3%. China State Construction's total operating revenue was 1.89 trillion yuan, with a year-on-year increase of 17.1%. The net profit attributable to the shareholders of the listed company was 51.41 billion yuan, with a year-on-year increase of 14.4%. Earnings per share was 1.25 yuan, with a year-on-year increase of 16.8%. China State Construction is an enterprise in the global construction industry with newly signed contract value and operating revenue both reaching 1 trillion yuan. China State Construction has moved up to No. 9 on the 2022 Fortune Global 500 list, No. 3 on the list of China's top 500 companies, and topped Engineering News Record's (ENR) Top 250 Global Contractors list. The company also received the highest credit rating in the global construction industry. China State Construction's market competitiveness and brand influence have continued to improve, and its leading position in the industry has been further consolidated.

China State Construction has been doing business in more than 100 countries and regions around the world, covering investment and development (real estate, construction financing and operation), construction engineering (housing and infrastructure) as well as survey and design and new business (green construction, energy conservation and e-commerce). In China, China State Construction has built more than 90% of skyscrapers above 300 meters, three-quarters of key airports, three-quarters of satellite launch bases, one-third of urban utility tunnels and half of nuclear power plants. One out of every 25 Chinese people lives in a house built by China State Construction.

Taking "expanding a happy living environment" as its mission, China State Construction adheres to core values of "quality assurance and value creation" and corporate spirits of "honesty, innovation, transcendence and win-win" to build a world-class enterprise with global competitiveness. It pursues to become the top brand in global investment and construction arena and a banner of China's reform, development and urbanization aimed to realize the Chinese Dream of national rejuvenation.

As the largest engineering contractor in the world,China State Construction represents the highest level in the field of housing construction in China.
With its business covering all the fields of urban construction and every aspect of project construction, China State Construction has completed many classic landmark projects at home and abroad featuring the changes of the times and commemorating economic and cultural development. It has also built many major infrastructure projects of rail transit, bridges and urban utility tunnels to serve the national economy and people's livelihood. By continuous optimization of business structure, China State Construction has contracted a large number of high-end projects of large-span factory buildings, exhibition centers and people's livelihood projects, and provided engineering services for high-end manufacturing industry.
As one of the most powerful investors in China, China State Construction makes investment in real estate development, financing, investment and construction projects, and urban comprehensive construction projects.
China Overseas Land & Investment Ltd., a subsidiary of China State Construction, is a leading real estate enterprise in China, with its brand value ranking 1st for 14 consecutive years. By strengthening internal resources integration and business cooperation, it specializes in integration of investment, development, design, construction, operation and services, and established a mature system for investment operation and risk management, with rich experience in investment and financing management and a large number of professional experts. It also provides financial services to promote integration of industry and finance. Fangcheng Investment & Development Group Co., Ltd., another subsidiary of China State Construction, makes investment in new type of urbanization for land plot development and land value improvement with unique capacity of integrated urban construction.
China State Construction is the largest architectural design enterprise in China, with its business covering many fields including architectural design,urban planning, engineering survey and municipal public works design, especially in planning and design of high-end airports, landscape gardens, sewage treatment, utility tunnels and sponge city. It develops fast in construction of new towns and infrastructure and has completed a large number of outstanding architectural design works with national characteristics; in addition, it leads the industry in original design, technological innovation and standards.
China State Construction is the representative and leader of the "Belt and Road" Initiative and the organizer and leader of integrated service value chain of investment, construction and operation of overseas projects.
China State Construction is one of the first groups of Chinese companies to go global. Its overseas business can be traced back to the 1950s'. So far, it has nearly 10,000 managers and engineering technicians abroad. Since the "Belt and Road" initiative was proposed in 2013, China State Construction made full use of its advantages in the brand "CSCEC", implemented the golden principle of "consultation, contribution and shared benefits" to integrate internal resources to explore international market, and help the countries along the "Belt and Road" to improve infrastructure and improve local people's livelihood.
Social Responsibility
Philosopy
Concept of responsibility
We take "developing happiness-filled space for stakeholders" as our mission, and take mission,

vision, core values and enterprise spirit as the most basic principles and concepts that the company believes in and upholds when dealing with the relationship with clients, employees, shareholders, communities and other stakeholders.

Responsibility culture

Culture and brand are c-existing, because a global famous brand must be supported by culture. Focusing on the mission of "expanding a happy living environment " and the core values of "quality assurance and value creation", we construct the corporate responsibility culture from the perspective of "culture and culture leading responsibility" with the carrier of "China Construction Doctrine" and "Ten Classics and Nine chapters", and promote the integration of responsibility into management, daily operation and employee work, and form the cultural leading social responsibility model.

Responsibility System

We set up the Social Responsibility Committee composed of corporate executives and department heads. The committee is responsible for drafting the overall CSR plans within the Company and establishing a social responsibility working system that covers all levels and fields of the Company. The social responsibility committee has a general office responsible for drafting social responsibility working plans and management system; organizing and implementing social responsibility practice; conducting external CSR communication and compiling sustainability reports.

Culture

Mission & Vision

EXPANDING A HAPPY LIVING ENVIRONMENT

We expand a happy living environment and create value for our customers. CSCEC works to “expand a happy living environment” and creates value for our customers. All CSCEC values stem from the recognition and trust of our customers, as we believe that they are best placed to offer a final judgment on our corporate value. We will continue to focus on the demands of our customers in order to exceed their expectations and provide systematic solutions.

To be the Investment and Construction Group with the Most International Competitiveness

Our vision is to become the investment and construction group with the most international competitiveness. We will realize it by utilizing our competitive advantages of the whole industrial chain of investment and construction and become a respectable group company and public company with the most international competitiveness. We uphold our own advantages, carry on the glorious tradition and enhance competitiveness through continuous innovation and system marketing, providing high quality and value-added products and services for the customers (government, enterprises and institutions, and individuals, etc.), creating value for stakeholders like employees, shareholders, partners, and making our due contribution to the economic development of China and even the world.

Corporate Values

QUALITY ASSURANCE AND VALUE CREATION

We focus on providing high-quality services and advocate strong management performance. We have always stressed a customer-oriented concept, firmly honor contracts with integrity, ensure the optimization of our engineering quality, and build service quality. We value the development of our “performance culture” and believe it is the best way to maximize value and achieve win-win outcomes with stakeholders.

INTEGRITY INNOVATION TRANSCENDENCE WIN-WIN

Integrity is an essential part of the development of our business; innovation is our magic weapon for success; transcendence is the driving force for our development; and a win-win is our value pursuit.

Codes of Conduct

Knowing is the beginning of action, and action is the result of knowing. Firm ideal faith and cultural concept should be turned into action and be tested through practice. Actions speak louder than words. Actions are more important when implementing the Credo of CSCEC. Much attention is to be internalized in the heart of the staff. The culture requirement of CSCEC can only be met when the core culture ideology is turned into staff behavior and custom in their daily work, both internally and externally.

The Code of Conduct and Protocol is the extension of the Credo of CSCEC, and it is the guide for implementing the Credo of CSCEC.

The Code of Conduct and Protocol is composed of two parts.

'Ten Codes of Conduct' advocates and requires managerial behavior and custom of staff. Among which, 'organizational behavior' attaches importance to the business ethics,and raise clear requirements as how to create sound organizational environment as well as improve the corporation cultural capacity.'Individual behavior' is concerned with detailed instructions, guiding and advocating the daily behavior of all CSCEC staff. 'We are against' makes standards of bottom line, and alerts staff from doing things against CSCEC culture.

‘Nine Aspects of Protocol’ sets up etiquette rules for CSCEC staff. Following standards of an internationalized corporation, the protocol explains the etiquette rules and detailed behavior that staff should pay attention to at the business and social circumstances. It helps staff to improve their professional quality, communication skills and set up good manner of both individual and the corporation.

Ten Codes of Conduct

Reaching the Best Quality

Organizational Behavior

Reaching the Best Quality. We attach great importance to process and the details, the result and efficiency. We establish our brand name with good quality.

Individual Behavior

We will follow high standard and strictly follow rules when doing things. We will behave ourselves when doing things. We will improve ourselves by taking account of the details.

We are Against

High speed of development with low quality. Producing under-standard products to count as good ones.

Being Professional

Organizational Behavior

We develop with our own character and advantage in the competition. We win trust from our partners with professionalism. We aim at being professional, sophisticated and strong. We create access to professionalism and bright prospect.

Individual Behavior

We are accountable, and work with ethics. We cherish every day and do well everything. We

should devote ourselves to our work.
We are Against
Speaking without careful studying. Working without professional ability. Muddle along, nothing to pursue.
Stick to the Rules
Organizational Behavior
There are no buts in front of rules and regulations. We let the standards to become our custom. We make implementation aggressively, speedily and completely. We make ourselves adhere to business ethics and keep business secrete.
Individual Behavior
Make the standard a habit. We follow principles and disciplines. We help each other and maintain the rules.
We are Against
Disobeying orders and doing things that are forbidden. Ignoring the rules. Always having excuse for not following the rules.
Keep on Innovation
Organizational Behavior
We always keep a mood of startup and passion for innovation. We make plan five years ahead, and we implement it three years ahead. We encourage innovation and tolerate failure during the process.
Individual Behavior
We appreciate good ideas. We are not afraid of challenging the professionals. We update ourselves every day. We keep thinking what can be improved.
We are Against
Being afraid of being exceptional. Rather caring less in the work. Being inactive towards work. Being over conservative and narrow minded.
Maintain High Efficiency
Organizational Behavior
We believe that consensus comes from communication and trust will bring about win-win situation. We should respect people’s dignity and treat everyone equally. We should take result based and efficient approach and get rid of the worthless details.
Individual Behavior
We should try to stand in other’s boots. We should try to listen, understand, appreciate and support other colleagues. We should learn the best from others and make the best of ourselves. We should try to do work immediately and do it well.
We are Against
Showing tender feeling without principle. Blocking communications. Making excuses at negligence.
Collaborative and Cooperative
Organizational Behavior
We believe the overall interest is our ultimate goal. We should make competition without defamation and cooperation instead of conflict. We should follow the common goal, establish the platform jointly and share the benefit.
Individual Behavior

We should look at the big picture and work from the details. We should help each other and often think in others’ boots. We should support others and accomplish ourselves.

We are Against

Lording it over and loot the resources to attract attention of others. Benefiting ourselves at the cost of others. Only thinking of our own benefit.

Clean and Broad-minded

Organizational Behavior

We should create an active and positive atmosphere for our staff. We should do good things and do not do bad things no matter how small they are. We should bear with different people and tolerate their differences. We should know staff are equal and their opportunities should be equal. We should treat staff equally with same standards.

Individual Behavior

We should establish ourselves and achieve our goals while helping others to do so. We should do good things and do not do bad things no matter how small they are. We stand upright and firm, and nothing will harm us. We should be simple and frank in our work.

We are Against

Working only on the surface. Only caring about individual gains and losses. Covering up one’s mistake and blaming others.

Hard Working

Organizational Behavior

We should keep going until our goal is realized. We should face the competition and go on fearlessly. We should take each of our accomplishment as the starting point and the difficulties as our steps. The whole team needs to study and we should keep it continuously.

Individual Behavior

We should have dream, passion and capacity. We should take step after step and the ladder is ascended. We should turn 1% possibility into 100% reality.

We are Against

Being lack of tenacity. Escaping in front of difficulty. Being lazy.

Green Development

Organizational Behavior

We should make most of the limited resources. We should turn green development into our competitiveness. We should embody ourselves into the nature and live with it.

Individual Behavior

We should start from our own behavior to save every bit possible. We should treasure our lives and live a green life. We should be full of public spirit.

We are Against

Being extravagant and wasteful. Being short sighted. Being eager for quick success and seek short term benefit.

Honest and Accountable

Organizational Behavior

We’d rather loose benefit than the creditworthiness. We honor contract with strong capacity and high quality. We speak with data and truth.

Individual Behavior

We honor our words, without compromise. We will work on it before requiring others. We speak

the truth and accountable.

We are Against

Making false or concealed report. Discrediting the opponent. Making tricks.

Nine Aspects of Protocol

General Provisions

Mutual Respect

Showing respect and treating people equal is our core principle communicating with outside world.

Showing respect and treating people equal means respecting ourselves while respecting others. We deal with people in equal base. That requires us treating people warmly while showing neither humble nor pushy.

We should respect the custom and taboo subject of people from around the world.

Sincerity and Tolerance

Sincere and tolerance reflects our manner at social communication occasions.

During the personal communication, we should be true to other people. We should understand other people to have their own actions and their own judgment. On one hand, we should be consistent with our own words and deeds; on the other hand, we should also be generous and understanding with others.

Moderate and Proper

It requires us to give moderate and proper treatment during social communication, according to different person, different things, at different time and locations.

Application of etiquette requires us to maintain the right balance. It is of a high skill of application of etiquette. Too much or not enough application of etiquette can hardly express our respect to other people.

Self Disciplined

Self discipline means that during social communication, we should be disciplined and keep appointment and observe the time. By following the rules and standards, our competence will be improved as time goes by.

Decent dressing and manner is resulted from well accomplished self discipline. Each employee represents the Chinese architecture in the external communication, not only reflects the personal insight and education, but also reflects the depth of thinking and cultural thickness of the enterprise.

Detailed Rules

Matching

1.Outfit Matching with the Situation

Serious and graceful in the office; Fashionable and particular during social communication; and comfortable and natural at casual occasion.

In the important occasions or international communication occasion, we should follow the requirement of the event. Ladies should better wear formal business suits and gentlemen should mainly wear western suits.

2.Color of Outfit

There should not be more than three colors for the outfit of a gentleman at business communication occasions. Gentlemen should choose darker color outfit if possible, with their shoes, belt and bag in the same color series.

There is no need to wear a tie in case of wearing jacket or T shirt. White socks and nylon socks are not matching with the suits.

It is suggested that ladies choose single color or simple and elegant color shirts to match their business outfits. The color of their shoes should match the color of their handbags and outfits.

3.Matching Jewels

When wearing jewels, it is suggested to accommodate the local custom. There should not be more than three kinds of them and should be no more than two pieces with each kind. The texture and color should be the same and the style should match with the outfit and other jewels.

Order

1.Order of Shaking Hands

Introducing senior person first. Between higher and lower level, only when the higher level person stretches out his or her hand, should the lower level person stretch out his or hers. Between older generation and younger generation, only when the older generation person stretches out his or her hand, should the younger generation person stretch out his or her hand. Between gentleman and lady, only when the lady stretches out her hand, should the gentleman stretch out his hand. Upon arrival of guests, the host should stretch out his or her hand first. At the departure, the guest should stretch out his or her hands.

2.Order of Making Introduction

Introducing senior person later. When introducing two persons, first introduce lower level person, and then introduce person of high level.Introducing younger person first and then the older person.Introducing men first and then women.

The host should make introduction first when meeting with guests. When receiving VIP guests, the highest level person of the host organization should make introduction.

During business communications, we must not forget introducing information of four aspects, namely organization, department, title and name.

3.When Taking Elevator

When taking elevator or escalator we should stand on the right side, avoiding standing side by side with others.

When we accompany guests moving in and out automatic elevator, we should hold the door and make sure the guests move in first. When we accompany the guests to an elevator that is controlled by the administrator, we should let our guests move in and out first. We should stand at the end or sides of the elevator, facing the elevator door, without making noise.

4.Guiding the Guests

At a strange place for guests, we should guide the guests 1 or 1.5 meters ahead of them, keeping guests at right hand. If the area is left hand traffic, we should keep our guests at left hand side. In case the guests are familiar with the direction, we should give the right to choose road to our guests.

5.Sitting Order

The senior person, the guests and the leaders should be seated first.
Sitting arrangement for the banquets: Usually the seat facing the door or the far-end seat from the main door is the host seat. International practice shows the right side is more respectable seat than the left side seat. The guest of honor will sit on the right side of the host, other hosts and guests should take cross seats.
We can only leave our seats when the host or the higher level person announces the banquet is over.
Rostrum Sitting Order: The front row is senior than the back row. The center seats are senior than the side seats. n China’s government communication occasions, the left side is senior than the right one. But in the international practice, the right side seat is senior than the left side seat.
Car Sitting Order: VIP guest should be seated at the seat behind the pilot seat. In case the host is driving, the VIP guest should sit at the pilot’s seat.

Timing
1.Giving and Receiving Gift in Diplomatic Communication Occasion
Generally, we should choose to give gifts face to face to our guests, attached with greetings and name cards. When receiving gifts, we should open it on spot and express thanks. In case we have to refuse the gift, we should explain the reason immediately. And we should respond with thanks in time upon receipt of gifts through mailing.
The gift to foreign guests should be packed well. Being treated equally is to be noted.
2.Receiving and Hanging up Phone Calls
When receiving phone calls, please pick up before it rings for three times. We should first give our titles, saying “Hello, This is CSCEC”, or “Hello, This is XXX Department”… It is polite to let our client or the other speaker to hang up the phone first.
3.Use Facial Expressions Properly
When greeting others or saying goodbye, we should show our respect and politeness with smile while looking at faces of our guests.
During conversation, we should keep eye contact properly, not to avoid eye contact, nor look at elsewhere.

Standard
1.Standard for Using Mobile Phones
Switch off mobile phone or turn it to mute mode, and try not to use mobile phone at the meeting, negotiations or at the meals.
In such circumstances, it is suggested to put the mobile in the briefcase, bags or pocket. Do not hold it in hand or put it on the table.
2.Standard for Title
During official communication, we should call others by their major titles. To the specialists or academicians, we should call their academic titles. In other occasions, we can generally call them Mr., Mrs., or Ms. and etc.
3.Standard for Using Name Cards
When receiving business cards, we should stretch out both hands to take it. We should read it carefully upon receiving. We should keep it properly and never forget taking with us.
4.Standard for Meetings

Follow the dress and discipline requirements of the meeting. We should follow discipline of the meetings and dress properly for the meeting occasions. We should avoid doing things like making noise, speaking privately, or chatting and laughing. Mobile phones should be turned into mute. We should try not to make any phone calls at the meeting.

We should get familiarized with the agenda and content before the meeting. We should arrive at the meeting on time and follow strictly the time limit for our speeches.

Do as Romans Do

1.Following the Custom of Guests When Giving Banquet

When inviting the guests to banquet, we should ask the guests if they have any dietary restrictions. Attentions are also needed when placing the dinnerware. It is not polite, for instance, to put the teapot mouth pointing to someone or place the chopsticks crossed.

2.Body Language

Every religion, every country, every nation and every region has its own body language. We should pay attention to the usage of head, hand, and other parts of our body’s language. We should not offend others with their taboo subjects.

3.Talking Topics

Personal privacy is an area to be avoided. During conversation, we should pick up the topics based on the extent of familiarization. We should avoid talking about religion, personal income, age and so on.

Career

“Human oriented" human resource management

We firmly believe that human resources are one of the core competencies of our company

For China Construction, one of the most competitive aspects is to have a large number of talented people who are loyal to the company and the cause, who believe that team interests outweigh personal interests and who are self-disciplined, self-motivated and self-developed to be able to manage and be good at marketing.

We will always follow the basic human resource management concepts of retaining talented employees by "career, emotion and treatment" and "individual concern"

and put this concept into our various human resources management policies. Through occupational planning, education and training and performance appraisal, we will establish individual value creation and ability combined with the sharing of enterprise development results by means of incentive mechanism, and strengthen the attractiveness and development of talent to provide stronger talent support for enterprise development.

We will always adhere to the principle of combining morality with ability and taking morality as the first

No one is perfect, but we will make the best of every employee. We always adhere to the principle of performance appraisal, and evaluate every employee according to his/her achievements. The only thing that can deny a person is his "conduct", and we will never employ a person with "misbehavior".

10.Transtech Engineering Corporation

Established in 1979, Transtech Engineering Corporation (TRANSTECH) is an independent legal entity and a key subsidiary of China Railway Group Limited (China Railways or CREC), a company being listed in Fortune Global Top 500 Corporations and in the World’s 500 Most Influential Brands. Transtech is a comprehensive international company, proficient in contracting

international engineering and construction projects, import & export activities, International investment and overseas labor cooperation services, who enjoys high reputation both at home and abroad .The works contracted by Transtech covers civil & industrial buildings, highways, railways, hydraulic works, ports, urban traffic systems, airports, tunnels, bridges and architectural decoration works, etc.; the projects operated by Transtech are in various nature, i.e. ICB (International Competitive Bidding), EPC (Engineering, Procurement and Construction/Turnkey) and PMC (Project Management Construction), BOT (Build-Owned-Transfer), BT(Build-Transfer) and BOOT(Build-Owned-Operate-Transfer) projects from general contracting to financing and/or investment projects.

11.China Road and Bridge Corporation

Introduction

China Road and Bridge Corporation (CRBC) is one of the four large State-owned companies in China that earliest entered into the international engineering contracting market. CRBC mainly undertakes contracting, investment, development and operation of projects such as road, bridge, port, railway, airport, tunnel, real estate and industrial park. With branches and offices in nearly 60 countries and regions in Asia, Africa, Europe and America, CRBC has established an efficient and rapid global market development network. CRBC is an important carrier, window and platform of overseas businesses of cCCC, a Global Fortune 500 company.CRBC has established relevant branches in nearly 60 countries and regions spreading from Asia, Africa to Europe and America.

Guided by the “go global" strategy and the “belt and road” initiative of the Chinese Government, CRBC has seized the opportunity to expand business and raise core competitiveness. It has adopted EPC and PPP modes and has undertaken a number of well-known projects, such as Tajikistan-Uzbekistan Highway, Pakistan Karakoram Highway Improvement Project, Mauritania Nouakchott Friendship Harbour Reconstruction and Expansion Project, Serbia Zemun-Borca Bridge, Mombasa-Nairobi SGR Project and Naivasha-Kisumu-Malaba SGR Project in Kenya, Hungary-Serbia Railway Project, and Pointe Noire New Port Project in Congo-Brazzaville. Along with these popular and high-quality "Connect-Heart Bridge", “To Wealth Road", "Development PortTand “Livable City", CRBC has made due contributions to the national welfare and community wellbeing of the host countries. With respect to local cultures and business compliance, CRBC is a welcome friend.

CRBC will adhere to the vision of “We Build a Better Connected Word; We Make Cities More Livable; We Create Better Life for People.", and try its best efforts to become “a vital responsibility bearer in economic and social development, a profound participant of coordinated development of regional economy, and a major provider of quality services in government purchase contracts” both in China and the host countries. CRBC will stick to the spirit of "Transportation integrates the world, the builder's Field is boundless", and devote to five business fields - infrastructure contracting; railway whole value chain; infrastructure and other industries investment; comprehensive urban and industrial park investment, development and operation; and overseas distinctive real estate. CRBC is committed to develop into a globalized enterprise group, outstanding with its five company-colours, following business ethics, pursuing operational excellence, respected by the public and loved by its employees, and popular in the capital market.

CRBC expands its business scope, ranging from road, bridge, port, tunnel, railway, municipal works, dredging, airport to investment and trade.
CRBC has established relevant branches in nearly 60 countries and regions spreading from Asia, Africa to Europe and America.
With the important contributions it makes to the infrastructure construction of China and other projects in host countries, CRBC constantly attracts the attention and affirmation of leaders of China and other relevant countries.
CRBC actively fulfills its social responsibility, and seeks for a benign interaction and integrative development of the enterprise, society and environment.
Vision and Values
Corporate Spirit
Building up roads and bridges,contributing to communities;Putting people first and searching for excellence.
Corporate Service Concept
Best service, high quality,efficient management,and great economic benefit.
Corporate Core Value
Credibility-oriented, fulfilling social responsibility; Quality first, delivering satisfactory service.
Corporate Mission
Be a responsibility partaker of socio-economic development an in-depth participant of coordinated development of regional economy and a premium provider of public service for governments.
Qualification&Honors
Honors for Projects
China Civil Engineering Zhan Tianyou Prize
International Arab Trophy Award
China National Engineering Luban Prize (Overseas Project)
National Excellent Construction Enterprise
National High Quality Engineering Award
Honors for Science & Technology
National Award for Science and Technology Progress
High and New Technology Enterprise Certificate
CHES' Yu-the-Great Hydraulic Engineering Science & Technology Award
Honors for Management
Tax-Paying Credit Class-A Enterprise
Advanced Collective of Central Enterprise
Advanced Unit of Central Enterprises in Ideological and Political Work
AAA Credit Enterprise Rated by International Project Contracting Enterprise Credit
National Advanced Enterprise in Construction Industry
Honors for Corporate Social Responsibilities
“Leading Enterprise” in 2012 Performance Evaluation on Social Responsibilities of Chinese International Contractors
China-Africa Friendship Award-the Top 10 Chinese Enterprises in Africa
Gold Prize for Corporate Social Responsibilities of Chinese International Contractors
Social Responsibility Management

Building Excellent Projects

Dedicated to building excellent quality projects in the international engineering market, CRBC undertook a number of iconic projects with international influence, including Karakoram Highway in Pakistan, Friendship Port of Nouakchott in Mauritania, Zemun-Borča Bridge in Serbia, Mombasa-Nairobi Railway in Kenya, etc.

Emphasizing Green Technology

CRBC has established cooperation with Tsinghua University, Tongji University, China Academy of Railway Sciences, etc. to conduct specific subject research, the results of which have been successfully applied to the construction projects as part of the effort to realize green environment-friendly construction.

Protecting Local Ecology

Adhering to the idea of environmental protection of “love, respect, conformity and protection” in the host countries, CRBC strictly abides by the local laws and regulations on environment protection, and integrates the idea of environment protection into all links of project planning, management and construction.

Protecting Employees’ Interests

One of the important measures of CRBC’s globalized development is the localization of its human resources. CRBC sticks to the principle of “people first” in the host countries, upholds the ideas of respect, communication, and cultivation, and protects employees’ interests. Meanwhile, it is CRBC’s commitment to the host countries’ government and people to do a good job in employee training and technology transfer.

Fulfilling Social Responsibility

Adhering to the idea of “full cooperation, active service, reciprocating society”, CRBC is actively engaged in social public welfare and charity activities, and provides timely aids to the host countries’ government and people when disasters occur.

Supporting Culture & Education

CRBC has always been concerned with the educational development in the host countries and gives priority to reciprocating the local education by assisting host countries’ students to come to study in China, establishing road and bridge scholarships with domestic key universities as well as conducting professional training during project construction to do a good job in local employee training and technology transfer.

CAREER

CRBC insists on the localized management, lays stress on recruiting and cultivating local employees.

Training of Employees

Technology transfer and training of local employees are not only CRBC's commitment to the local government and people, but also an important part of proving Local capability of building infrastructure. CRBC organizes all kinds of training such as on-the-job training, safety training, professional courses and so on.

Communication Mechanism

We full safeguard employee' rights to know, supervise and participate in decision-making, build channels for good communication between Chinese and local employees, and give full play to the role of employees in democratic management.

Remuneration and Benefits
In strict accordance with local laws, CRBC has paid social welfare expenses for all local employees. In addition to various insurances required by local law, CRBC offers extra benefits to employees, striving to create a healthy, secure working environment.
Establishing Awards to Encourage
The selection of “CRBC Excellent Local Employees” is carried out aiming to commend and promote the excellent local employees with outstanding performance in the reform and development practice of CRBC, set up advanced role models, guide and encourage the employees to strive for excellence.
Caring for Employees' Life
CRBC is always been caring for staff health and devoting to providing healthy, safe and humane working and living environments. CRBC has adopted various ways to help out needy employees and strengthen local employees' sense of identity and pride.
Cultural Exchange
Various cultural exchanges enable both Local and Chinese staff to relax in their spare time, develop healthy living and working habits, and deepen friendship.
Address:
As an international company engaged in large infrastructure construction and investment business worldwide, CRBC always adheres to the philosophy of “building excellent projects and creating first class brand”, proactively creates greater value for clients, and contributes to the development of the company and the society. While continuously seeking higher value standard, CRBC operates in line with compliance, sticks to its commitment, endeavors to innovate, and strives to establish mutually beneficial and win-win cooperation with its stakeholders.

Growing out of the Foreign Aid Office of the Ministry of Transport of China, CRBC has been undertaking overseas projects aided and financed by the Chinese government since 1958. In 1979, the company was formally established and entered the international project contracting market. It became a wholly-owned subsidiary of China Communications Construction Company Limited (CCCC) after a major corporate restructuring in 2005.

In response to the “Going Global” Strategy and the “Belt and Road Initiative” in recent years, CRBC has formed a market network connecting about 100 countries, including almost all the countries and regions along the Belt and Road, and has undertaken a great number of key projects with far-reaching impact on the economic development of the host countries, turning CRBC into a world renowned brand. In addition, CRBC has contributed significantly to the construction of the core road sections of China Pakistan Economic Corridor, accounting for 15% of the total length of the Corridor, creating favorable conditions to leverage the strategic role of Gwadar Port. CRBC has completed the China-Tajikistan Highway, Tajikistan-Uzbekistan Highway, and other hub projects in Central Asia, Central and Eastern Europe regions with total mileage of 2,400km. The Mombasa-Nairobi Standard Gauge Railway, completed open to operation in 2017, extends from the “Maritime Silk Road” to the African continent, and continues with the maritime voyages of the Chinese navigator Zheng He for development in the new era... Zemun-Bolza Bridge over the Danube River in Serbia, one of the key projects of between China and Central and Eastern European Countries (“16+1”), is hailed as “Friendship Bridge of China

and Serbia”.

Meanwhile, CRBC keeps pace with the times, implements deeply the reform on the supply side, and leverages fully its advantages to provide integrated solutions for the host counties by accurately observing the demands and requirements of local government, project owners and socio-economic development. Moreover, CRBC effectively promotes the quality improvement of supply system by exploring such new business modes as “Integrated Development of Industry, Financing and Construction”, and “Synergized Development of Road, Railway and Port”, cultivating infrastructure operation, economic development zone, and industrial zone in new fields with new forms , and conducting win-win cooperation with all partners of the industrial chain.

While committing itself to realize the “hard connectivity” of infrastructures, CRBC has attached great importance to the “soft connectivity” with people of the host countries, by fulfilling the social responsibilities and contributing to the local people and society. CRBC has delivered numerous high-quality and eco-friendly “green projects”, achieving the economic benefits and environmental protection, and promoting the harmonious development between man and nature. CRBC has proactively organized a variety of social welfare and charity activities, and rendered generous support to local government and people to deal with natural disasters or dangerous circumstances, leaving the people with the impression of “Turning to CRBC in case of difficulties”. CRBC has also promoted the localization management with local content in the project construction and operation so as to share the development achievements with local companies and people, and build a mutually beneficial business ecosystem.

In the future, CRBC will work ambitiously to provide more excellent products and services to clients with international vision and strong sense of social responsibility CRBC is looking forward to establishing win-win cooperation with all the partners for a brighter future.
12.ZTE south africa
Global Leading Integrated Communication Information Solution Provider
With innovative technologies and product solutions, ZTE serves global telecom operators, government and enterprise customers, and consumers. Covering more than 160 countries and regions, ZTE serves over 1/4 people worldwide, and is committed to achieving a bright future of connectivity and trust everywhere. ZTE, founded in 1985 is the world's leading provider of integrated communications and information solutions.
Through providing innovative technology and product solutions to telecom operators and government and enterprise customers in more than 160 countries and regions, the company enables users all over the world to enjoy all-round communication such as voice, data, multimedia and wireless broadband.
ZTE serves over 1/4 people worldwide, and is committed to achieving a bright future of connectivity and trust everywhere.
Our vision
To enable connectivity and trust everywhere
Our mission
To connect the world with continuous innovation for a better future

Our Culture
Customer Success, Value Contribution, Pursuit of Excellence, Simplicity Prevails
Corporate Culture
Core Values
Respecting each other and being faithful to the ZTE Cause
Serving with dedication and being committed to our customers
Endeavoring with creativity to establish a famous ZTE brand
Operating with scientific management to increase corporate performance
Behavior Guidelines
Cooperative Take the initiative
Responsible Dare to assume responsibility
Professional Win first place
Pragmatic Tell complete truth
Harmony.
ZTE adheres to the concept of sustainable development globally to achieve harmonious coexistence of society, environment, and stakeholders.
CSR Report
ZTE member of the UN global Compact and GeSl, implements Corporate Social Responsibility and contributes to global sustainable development.
Environmental protection
Adhering to the idea of "innovation, integration, and green", ZTE will promote "peak carbon" and "carbon neutrality".
Conflict Minerals
ZTE abides by the United Nations Global Compact and other universally accepted international conventions and practices respects human rights and the environment.

13.PetroChina Company
PetroChina Company Limited is a joint stock limited company incorporated on November 5, 1999, upon the restructuring of China National Petroleum Corporation (CNPC). The American Depositary Shares ("ADS"), H shares and A shares of the Company were listed on the New York Stock Exchange, the Stock Exchange of Hong Kong Limited ("HKSE" or "Hong Kong Stock Exchange") and Shanghai Stock Exchange on April 6, 2000, April 7, 2000 and November 5, 2007, respectively.

PetroChina is one of the major oil and gas producers and distributors in China, as well as a significant player in the global oil and gas industry. We are engaged in a wide range of activities related to oil, gas and new energy and new materials, and sustainably provide energy and oil products for economic and social development.
It implements the strategies of innovation, resource, market, internationalization, green and low-carbon. We are also committed to promote green development and energy contribution, to boost the growth momentum of customers, and to empower people to enjoy a better life
PetroChina’s logo symbolises our commitment to ensuring harmony between the development of energy and the environment. The flower-shaped logo’s colors are those of China’s national

flag, with its ten petals representing our core businesses. The solid red base illustrates PetroChina's strength and cohesion, while the rising sun highlights our brilliant future.

Health, Safety and Environment

We strongly acknowledge that a good ecological environment is fundamental for human beings to survive and remain healthy, and it is the most important factor in terms of the well-being of the people and productivity. Caring for life and protecting the environment to build a beautiful world with harmony between human beings and nature have been integrated into our work philosophy. We are always committed to the QHSE principle of "people-centered, quality foremost, safety first, environment prioritized" to achieve "zero defects, zero injuries and zero pollution", to promote economical production, cleaner production and safe production, and endeavor to build PetroChina into a resource-conserving, environmentally friendly and safety-conscious business.

Business Strategy

PetroChina will shoulder greater responsibilities and face more challenges as well as more opportunities. We will implement our strategies with respect to innovation, resources, market, internationalization and green and low-carbon, towards the goal of building a top-class international energy company on a quality-based path. While focusing on core business, reform and innovation, quality and profitability, and corporate governance, we will also seize the opportunities for low-carbon transformation, accelerate the process of building up new advantages in green development and contribute to the UN Sustainable Development Goals 2030, creating and sharing a bright future with all of our stakeholders.

Strategies

Innovation: We regard innovation as the primary driving force for development, and pursue all-around innovation centering on technology. We promote innovation in industrial chains to ensure they are more value-added. We improve our independent innovation capability in key technologies to provide the Company with the maximum level of competitiveness and contribute to China's overall strength in strategic technology.

Resources: We focus more on acquiring and supplying resources sustainably at low cost and from diverse sources. We develop domestic hydrocarbon resources efficiently, import oil and gas via multiple channels, make active planning for non-fossil energy, ensure talents remain our most important resource, and mobilize resources such as land, capital, technology and data in a coordinated way. We endeavor to increase the value of resources and strengthen the material basis for development.

Market: The market plays a decisive role in resource allocation. Being market-oriented and customer-based, we engage in proactive analysis of market situations and take active measures to adapt to, expand and guide the market. In both the domestic and international markets, we have rapidly established a modern marketing system, in order to ensure we always maintain a decisive competitive edge.

Internationalization: We optimize the strategic pattern of international cooperation on oil and gas, and enhance international business operations, capital management and global resource allocation. We internationalize our philosophy, management, technology, criteria and personnel, and engage in global energy governance. We improve our international business capability and industry influence, and build an energy community of shared interests.

Green and low-carbon development: We take continuous steps to increase the proportion of

low-carbon and carbon-free energy resources in our energy mix, promote clean production and eco-friendly industries, and build the low-carbon energy supply system. With energy conservation as the priority, we pursue economical and efficient utilization of energy resources, and explore a new business model based on low costs. We endeavor to be competitive for green and low-carbon development.

Development Direction

We will vigorously carry out oil and gas and new energy business, which includes domestic and overseas oil/gas exploration and development, natural gas sales, and new energy. These businesses represent PetroChina's core competitiveness. We will highlight efficient exploration and profitable development. We will maintain oil production and increase gas production to ensure improved upstream benefits domestically. We will optimize the composition, business structure and regional layout of our overseas assets. We will enhance the integration of natural gas production, supply, storage and marketing. We will industrialize new energy projects in organic coordination with oil and gas. We will build a clean energy supply system with the orderly replacement of multiple energy resources, and develop PetroChina into a world-leading clean energy producer.

We will efficiently expand our refining and chemicals, marketing and new materials businesses, which include refining and chemicals, marketing of refined products, new materials and international trade. These businesses are critical bridging and value-added aspects of PetroChina's business chain and major channels for realizing revenue and enhancing brand value. We will strengthen the integration of refining, marketing and trading in order to coordinate output and sales and improve our marketing capabilities. We will implement acceleration programs for new materials. With these efforts, we strive to improve the efficiency and profitability of industrial chains.

Society and Environment

We strongly acknowledge that a good ecological environment is fundamental for human beings to survive and remain healthy, and it is the most important factor in terms of the well-being of the people and productivity. Caring for life and protecting the environment to build a beautiful world with harmony between human beings and nature have been integrated into our work philosophy. We are always committed to the QHSE principle of "people-centered, quality foremost, safety first, environment prioritized" to achieve "zero defects, zero injuries and zero pollution", to promote economical production, cleaner production and safe production, and endeavor to build PetroChina into a resource-conserving, environmentally friendly and safety-conscious business.

HSE Management

We attach great importance to health, safety and environment (HSE), and always give top priority to HSE work in our business development. We adhere to the concept of "integration and coordination between resource development activities and environmental protection". We follow a uniform HSE management system, and maintain globally integrated HSE management supervision. We continuously strengthen supervision and HSE training, in order to enhance our HSE management performance. Our safety and environmental protection efforts are making steady progress.

We have established an environmental protection incentive mechanism to commend units and individuals for their outstanding achievements. The environmental protection performance as a

KPI index is included in the performance appraisal of senior management of the Company and senior managers of regional companies, and the accountability system for environmental protection performance and the lifelong accountability system for ecological damage have been put in place.

The Company, for the purpose of eradicating environmental pollution and ecological disruption events of a relatively large or more serious level, conducts deep investigation and assessment of ecological environment risks, constantly improves the "three-grade prevention and control" system, strengthens prevention and control measures against ecological environment risks, and highlights the management of hidden environmental risks in key fields. The Special Emergency Response Plan for Environmental Emergencies was revised to further define the responsibilities of all departments and levels of the headquarters in more detail and improve the operability of the plan. All enterprises regularly carried out emergency response drills for handling environmental emergencies.

Sustainable Utilization of Resources

We attach importance to the protection and rational use of resources. We strengthen the protection of water resources, conserve fresh water resources, rationally utilize land resources, and strive to improve the utilization efficiency of energies and materials in order to minimize the consumption of resources. In 2021, we saved 0.7 million tons of standard coal and 9.69 million cubic meters of water. Fresh water consumption decreased by 2.59% year on year. In 2021, we saved 1,120 hectares of construction land.

Clean Energy

Natural Gas： Natural gas is the Company's strategic, growing and value-added business, which plays a key supporting role in the process of energy transition, and it is also a bridge for the transition from fossil energy to clean energy. The vigorous development and utilization of natural gas is a basic project that runs through the process of green and low-carbon transition and the development of the Company. The Company insists on setting natural gas as the strategic focus, and promotes the development of conventional gas and unconventional gases such as tight gas, shale gas and coalbed methane. In addition, the Company imports natural gas through multiple channels in order to form a diverse energy supply system.

In 2021, we achieved domestic natural gas production of 137.8 billion cubic meters, representing an increase of 5.5% year-on-year, of which saleable natural gas accounted for 119.56 billion cubic meters, up 5.7% year-on-year. We increased imports of pipeline gas and LNG, and continuously improved the natural gas marketing network, so that the gas supply covers 31 provinces (municipalities and autonomous regions) and the Hong Kong Special Administrative Region. We sold 194.591 billion cubic meters of natural gas domestically in the year, which, according to the equivalent calorific value, is equivalent to a substitution of 259 million tons of standard coal and a reduction of 260 million tons of carbon dioxide emissions, which contributed to the optimization of China's energy mix and the construction of a beautiful China.

We promote the comprehensive utilization of natural gas in city gas, industrial fuels, natural gas power generation, chemical feedstock and vehicle fuels. We sped up the renovation project of replacing coal burning with natural gas, built a zero-coal demonstration zone and increased the utilization ratio of natural gas and fuel gas. We continued to carry out "coal to gas" and "gas to replace coal" projects and continuously increased the preference for resources in key areas. Projects such as "Gasification of Xiong'an", "Gasification of Hebei" and "Gasification of Yangtze

River" have been promoted continuously, in order to bring clean energy to large numbers of households.

New Energies and New Businesses: We take the development of new energy as new driver for the development of green and low carbon transition. We set up a new energy and new material business development leading group, strengthen strategic planning for the development of new energies and new businesses, and the construction of the business management system, and accelerate the expansion of new energy businesses such as geothermal energy, wind and solar power, hydrogen energy, and charging (battery exchange) stations. The research institute for new materials in Shanghai and the research institute for new energies in Shenzhen were set up to enhance investment in technical R&D and provide technical support for the development of new energies and new materials business.

In 2021, PetroChina's development of new energies and new businesses was accelerated comprehensively, especially with great progress in the fields of geothermal energy and hydrogen energy. A total of 39 new energy projects were completed and put into operation, and the new energy development and utilization capacity increased by 3.50 million tons of standard coal per year, with the total amount developed and utilized reaching 7 million tons of standard coal per year.

Response to Climate Change

We agree with the temperature goal set out in the Paris Agreement to hold the global average temperature increase, and implement the "carbon emission peak and carbon neutrality" goal proposed by the Chinese government. We strive to be the supplier of clean energy and the promoter of low-carbon transition of society, and share the practices of greenhouse gas control with industry peers and all sectors of society. Greenhouse gas reduction programs and initiatives supported and initiated by the company: Paris Agreement, China's National Program for Addressing Climate Change, China's Policies and Actions for Addressing Climate Change, China Technology Strategic Alliance for Carbon Dioxide Capture, Utilization and Storage (CTSA-CCUS), Oil & Gas Climate Initiative (OGCI), Carbon Emission Peak and Carbon Neutrality Declaration of China's Petroleum and Chemical Industry, and Alliance of Chinese Oil and Gas Enterprises for Promoting Methane Emission Control.

The Board attaches great importance to climate-change-related risks and opportunities. We pay close attention to policies, laws, technology and market changes related to the global and Chinese low-carbon economic transition, and have continuously conducted thematic studies for many years to identify and assess the real challenges and potential impacts of climate change on the Company, and incorporated them into our strategic planning and management practices. At the Company's management level, we have continued to strengthen climate-related risk management, carbon emission management and carbon risk management, improve the carbon emission control system, develop low-carbon industries, improve clean energy supply, and actively participate in cooperation among global oil and gas industry in tackling climate change. See the Environmental, Social and Governance Report for details of the governance actions of the Board of Directors and the management of climate risks.

In order to curb carbon emissions, PetroChina made continuous efforts to strengthen carbon emission management and carbon risk handling, and improved the carbon emission control system of the Company. We paid great attention to carbon emissions and carbon footprints in production and operation, and endeavored to reduce the consumption of fossil energy and

reduced the energy consumption intensity. We actively participated in the construction of the carbon market and carried out in-depth cooperation with the oil and gas industry globally to address climate change. We also strengthened methane emission reduction management and control, formulated the Action Plan for Methane Emissions Control to plan for methane emission targets and actively participated in the activities of the Alliance of Chinese Oil and Gas Enterprises for Promoting Methane Emission Control to encourage the oil and gas industry to accelerate the green and low-carbon transition.

PETROCHINA TAX POLICY

PetroChina tax policy is consistent with its corporate strategies. PetroChina is committed to paying taxes in countries where business activities take place and value is created in compliance with applicable tax laws and regulations, and to contributing to local economic growth and sustainable development of the company.

PetroChina carries out tax planning in support of its business strategy and in compliance with international tax agreements and principles, as well as tax laws of host countries. PetroChina opposes to aggressive tax planning to avoid potential risks and negative impacts on the company’s reputation and sustainability.

PetroChina pays taxes in compliance with applicable tax laws of host countries, its business principles and code of conduct, and adheres to high professional standards to ensure the legitimacy and accuracy of tax payment in a timely manner.

PetroChina advocates tax transparency, and ensures the accuracy and completeness of tax information disclosure in line with requirements of tax authorities.

PetroChina manages tax risks through its effective internal control system and engages with tax authorities and external advisors in case of different interpretation of tax laws and regulations for professional judgment.

PetroChina carries out intercompany transactions based on arm’s length principle and not for the purposes of eroding tax base and shifting profits.

PetroChina takes part in promoting a fair, consistent and stable taxation environment globally and strives to establish a fair, transparent and effective dialogue with tax authorities of host countries.

Work and Career

Employees Rights

We remain committed to a people-first philosophy in treating our employees with respect and equality while protecting their legal rights. We endeavor to resolve the most practical issues that our employees are concerned with and ensure that all employees equally share the fruits of our development.

Remuneration and Benefits

We have established a sound remuneration system, implemented a differentiated management and control system which classifies total payrolls by grade and category based on different enterprise functions, positioning and business characteristics, and improved a payroll decision and normal increase system that basically suits the labor market and is linked with the performance and labor productivity of the Company. We have stepped up targeted incentives for key groups, actively promoted mid- and long-term incentive policies, and given priority to remuneration distribution to key positions, backbone personnel, scientific and technological talents and frontline employees, so that employees can realize their full potential. We have paid all social insurance premiums for employees on time and in full in accordance with the Social

Insurance Law of the People's Republic of China, improved corporate annuity and supplementary medical care and insurance, and improved production and living conditions for employees, in order to ensure employees' interests.

Democratic Participation

We fully respect employees' democratic rights and lawful interests. We attach great importance to employees' role in democratic management, democratic participation and democratic oversight. Apart from establishing and improving trade unions, we have put in place a democratic style of management and a transparent system to deal with matters at plants, through the Employees' Congress system, to ensure employees are well-informed and have the means to participate in and supervise corporate management. We have further standardized the content, procedures and model of our open system for matters at our plants by clarifying duties and authorities, the organizational system and working processes for the Employees' Congress.

We have established multiple channels to communicate with employees and continued to implement democratic procedures. We communicate across different levels of the Company and conduct multi-level communication through meetings with staff representatives and online discussions, to encourage employees to participate in the management of production and operations.

Employees Health

Our health and safety management covers all staff, including staff in the entities in our supply chain. We give top priority to employee health and safety by emphasizing occupational health management and protection at field operations, continuously improving working conditions, abiding by the statutory working hours and holidays required by the places of business and regarding employees' mental health as a priority.

Occupational Health

We attach great importance to occupational health. We carefully implement the Occupational Disease Prevention Law in the prevention and management of occupational hazards by means of gas defense, dust removal and noise reduction. We deepen occupational healthcare and organize occupational health checks. We understand and analyze the check results and take active measures to prevent and eliminate any negative impact of occupational diseases on employees' health. In 2020, the establishment rate of employee occupational health monitoring archives was 100%, the detection rate of workplace occupational hazards was 99.33%, and the occupational health examination rate for employees facing occupational hazards was 99.25%.

Mental Health

We care about the employees' mental health. We improve the employee vacation system and regularly organize employee health examinations. We set up a hotline and a website for psychological counseling, and perform mental health education and training in various forms, in order to encourage employees to adopt a positive and healthy attitude. We provide training sessions on mental health management, extend the Employee Assistance Program (EAP), and invite psychologists to provide mental health counselling to our employees. In 2020, our overseas mental health team provided psychological assessment services to approximately 3,000 employees, and "one-on-one" online psychological consultation to more than 400 overseas employees in key overseas regions and projects during the COVID-19 outbreak period.

Localization and Diversity

At PetroChina, we embrace a respectful, open and inclusive culture and we are committed to

"selecting more local talents who live close to our overseas operations, upgrading their professionalism and making them more responsive to market forces". We abide by all laws and regulations of the host countries. We established an optimal system for recruitment, deployment, performance appraisal, incentives and penalties. We work hard to attract and train top local talents and to provide them with a working environment conducive to their career development.

Local Hiring in Overseas Operations

We actively recruit and train local employees and do not discriminate against local candidates for managerial positions, providing local people with job opportunities. Professionals employed in our overseas projects involve multiple disciplines, such as exploration and development, refining and chemicals, pipeline operation, international trade, finance, accounting, and human resources management.

Cultural Integration

We respect cultural diversity and local customs. We promote communication and exchange between employees with different cultural backgrounds, and advocate respect for different values and traditions, in order to foster cross-cultural cooperation in a culturally diverse workplace.

14.China National Petroleum Corporation (CNPC)

China National Petroleum Corporation (CNPC) is an integrated international energy company with businesses covering oil and gas E&P, new energies, refining & chemicals and marketing, new materials, support & services, as well as capital & finance etc.

Based in China, we have oil and gas assets and interests in more than 30 countries in Africa, Central Asia-Russia, America, the Middle East, Asia-Pacific, and other regions.

CNPC is China's largest oil and gas producer and supplier, as well as one of the world's major oilfield service providers and a globally reputed contractor in engineering construction, with businesses covering oil and gas E&P, new energies, refining & chemicals and marketing, new materials, support & services, as well as capital & finance etc.

Our Vision

To become a world-class integrated international energy company built to last.

Our Strategies

Innovation. Resources. Market. Internationalization. Green & Low Carbon.

CNPC implements actively the strategies of "innovation, resources, market, internationalization and green & low Carbon", and adheres to the mission of "pursuing green development and supplying reliable energy to fuel our customers' growth and power people's happy life" to facilitate the energy transition, achieve the goals for carbon peak and carbon neutrality, and become a world-class integrated international energy company built to last.

Our Values

Pursuing green development and supplying reliable energy to fuel our customers' growth and power people's happy life.

CNPC's Logo and Brand

CNPC's logo embodies our commitment to ensuring harmony between the development of energy and the environment. The flower-shaped logo's colors are those of China's national flag, with its ten petals representing our core businesses. The solid red base illustrates CNPC's strength and cohesion as a state-owned enterprise, while the rising sun highlights our brilliant future.

CNPC is our main global brand, which exists on our oil production platforms, refineries,

equipment manufacturing products and in the offices of the company, as well as on our lubricating oil products and service stations.

The acronym CNPC first appeared in public in 1998 and, thanks to our quality products and good service, the brand has become well known to millions of customers.

Harmony is part of our brand values, as well as the way in which we manage our company's relationships with society, our employees and our customers. We strive to realize harmonious development in all respects through our hard work, broad vision and creativity. In fact, this value has already been integrated into the company's business operations. In addition to harmony, our brand values also include honesty, innovation, performance and safety.

Localization & Diversity

With the rapid development of international oil and gas operations, CNPC keeps promoting local hiring and HR management in its overseas projects in host countries, and has trained a great number of technical and management personnel for the local petroleum and petrochemical industries. We advocate a respectful, open and inclusive culture, and ensure the equal rights, equal opportunities and fair treatment of all employees regardless of their nationality, race, gender, religion or cultural background. By integrating diverse cultures, we strive to promote better understanding and trust among employees, in order to build a team of great cohesion and creativity.

Management of Local Employees

CNPC values the contributions of local employees and attaches great importance to local talent training. We promote local hiring and encourage our overseas projects and oilfield service companies to offer more jobs to surrounding communities. We have Protocols on Local Employee Management in Overseas Operation in line with the laws and regulations in host countries, covering hiring, the monitoring of attendance, vacation, performance evaluation, incentives and penalties for each management process and position, to attract and retain outstanding local talents.

Our overseas operations are hiring professional talents in E&P, engineering and construction, international trade, finance, accounting and human resources management in more than 80 countries and regions. In 2021, international employees and local employees accounted for 86.67% of the Company's overseas employees.

Fostering Multicultural Environment

CNPC embraces a respectful, open and inclusive culture. We promote understanding and respect for different values and traditions, and foster cross-culture cooperation in a culturally diverse workplace. Chinese employees are encouraged to learn about the laws, history, culture and religious customs of local communities. On the other hand, foreign employees are invited to visit our headquarters in China and experience Chinese culture.

Technology and Innovation

Technological innovation is an important support and powerful driving force for the Company to promote high-quality development and build a world-class comprehensive international energy enterprise. With innovation as our top strategy, we continue to invest in science and technology and enhance our competency in research and development. We have built innovation capabilities along the industrial chain to create value for now and the future.

We have made new breakthroughs in independent innovation, and obtained and promoted a batch of new important technology achievements, which further enhanced the capability of independent innovation and core competitiveness, and strongly supported and led the development of our main business.

Innovation Platforms

We have 84 research institutes, 21 national R&D institutions and 54 Company-level key laboratories and experimental bases, covering the upstream, midstream and downstream sectors, and supporting and leading the Company's sustainable development.

Maintaining a two-level (national-level and provincial/industry-level) skills cultivation platform, we foster a talent pool mainly including 24 academicians and more than 30,000 researchers. We own a total of approximately 20,000 patents obtained in China and overseas.

Technological Achievements

We regard innovation as our top priority and a critical step, and we have encouraged the building of an open ecological environment for innovation. We led and participated in the "4Mt/a coal indirect liquefaction technology innovation, development and industrialization" project, which won the first prize of the National Science and Technology Progress Award. Our other two R&D projects, "Key technology and its application for efficient development of coalbed methane reserves in complicated geological conditions" and "Packaged technology for catalytic cracked gasoline super-depth hydrodesulfurization-alkene phased controlled transformation", won second prizes of the National Science and Technology Progress Award. "Key technology for new polymer flooding to significantly enhance recovery" won the second prize of National Technology Invention.

Technological Cooperation

We continue to deepen the construction of strategic partnerships, consolidate our strategic alliances with international companies to promote the sharing of superior technical resources and the exchange of cutting-edge technologies, and promote cooperative research and talent training with the Chinese Academy of Sciences, China University of Petroleum and other research institutes and colleges and universities in the frontier of oil and gas exploration and development, new materials, new energy and other fields.

Digital Transformation and Intelligent Operation

PetroChina regards digital transformation as a major strategic measure to modernize its corporate governance system and governance capabilities, and vigorously promotes digital, networked and intelligent development. The use of digital technologies represented by cloud computing, the Internet of Things, 5G, big data, artificial intelligence, etc., drives business model reconstruction, management model transformation, business model innovation and core

capability improvement, realizing industrial transformation and upgrading and value growth.

PetroChina makes efforts in such key orientations as intelligent technology and product innovation, integrates digital technology into the products, services and processes of the oil and gas industry chain, reconstructs the value system, adjusts production relations, and transforms from a capacity-driven development model to an innovation-driven development model. PetroChina will focus on forming new industries, new business forms, and new models that conform to the characteristics of “Digital PetroChina”, based on new elements, new driving forces and new capabilities. Adhering to the general principles of “value orientation, strategic leadership, innovation drive and platform support”, PetroChina implements digital transformation in accordance with the three main lines of business development, management reform and technology empowerment and, through the construction of industrial Internet technology systems and an application ecosystem that takes cloud platforms as its core, creates a digital transformation strategic framework.

15.Datang

Founded on December 29th, 2002, China Datang Corporation Limited(“CDT”) is a large scale state-owned power generation enterprise,with main businesses covering electric power, coal &coal chemical industry, finance, environmental protection, trade&logistics and emerging industries. CDT owns 5 listed companies, and 36 regional subsidiaries and specialized companies.

Guided by the vision of building CDT into a first class international energy enterprise, CDT fully develops 6 business sectors, including power, coal, finance, overseas business, coal chemical and energy service.

By the end of 2022, CDT’s power generation assets in operation and under construction are widely distributed in 32 provinces all over China, including HongKong and foreign countries and regions such as Myanmar, Cambodia, Laos, Indonesia etc., with assets amounted to 860 billion RMB, total installed capacity 172 GW, with clean energy units accounting for 42%. CDT has been listed in Fortune Global 500 companies for 13 consecutive years.

CDT currently boasts of the largest thermal power plant in commercial operation, Inner Mongolia Datang International Togtoh Power Company (capacity of 6,720 MW). Chifeng Saihanba Wind Farm(capacity of 1,708 MW), one of the largest operational onshore wind farm in the world. Datang Dongying Power Generation Company, the world's first six-cylinder six-exhaust 1,000MW Power Plant. CDT is responsible for supplying more than half of the capital city's electricity, as well as heating area of 800 million square meters in the "Three Norths" region of China.

Facing the 14th Five-Year Plan and its future development, China Datang is striving to build a "China's best and world class" energy supplier, guided by the new development philosophy.
Superlarge power corporation with installed capacity of 172 GW,

Owns 5 listed companies,

Total assets reached RMB 860 billion,

Named to Fortune Global 500 list for 13th consecutive year since 2010,

Assets distributed in 31 provinces, autonomous regions and municipalities, Hong Kong SAR and overseas

CSR

Devotion to Public Welfare and Charity Since its establishment, apart from continuous development, CDT has also devoted itself to public welfare and charity activities. We have called upon our employees as volunteers to do their best to be devoted to pandemic prevention and control, emergency rescue and relief, respect for the elderly, caring for children, cultural assistance, public welfare, environmental protection, etc. In this way, we aim to brighten the road of love with our effort in public welfare

Leadership Statement

The year 2022 crystalized memorable achievement and great pride of CDT. As a super-large energy conglomerate, CDT worked hard and moved forward with determination, and exerted unremitting efforts to build a community of all life on Earth and a clean and beautiful world for us all.

We furthered energy cooperation and enjoyed prosperity and development opportunities together with the countries along the “Belt and Road”. Energy constitutes an important material basis for the development of human society. CDT actively echoed with the “Belt and Road” Initiative and pushed energy cooperation evolution from “free sketch” to “elaborate painting”. Currently, seven overseas projects have been put into operation, including two hydropower stations and four clean coal power plants. Gross installed capacity of 1.5 million kW has been under construction or in service. Power grid projects have been launched for three substations and 294-kilometer lines. All ongoing projects maintained safe and stable operation. CDT reaped plentiful social and economic benefits, promoted international production capacity cooperation in the win-win situation, and contributed to boosting the economic development and people’s livelihood development of the countries.

We firmly shouldered responsibility, stayed true to grand mission, tided over difficulties and successfully fulfilled the task of guaranteeing energy supply. Confronted with severe and complicated situation of energy supply guarantee and the daunting operating pressure from overspending power generation cost, CDT placed top priority over guaranteeing supply and raising efficiency, spared no effort to produce electric power and heating power, and gained ground in stable production and output increase for coal and gas. As an enterprise committed to the output of more than 50% of power supply in Beijing and residential thermal energy of 800 million square meters in the northern China, CDT succeeded in fulfilling supply guarantee tasks. The Datang Power Grid, built in Cambodia, transmitted more than 21.4 billion kWh of electricity, propelling the development of Cambodian power industry. Since Dapein I Hydropower Project in Myanmar was put into operation in 2010, it has generated more than 10.2 billion kWh of electricity, lifted the long-standing electricity shortage bottleneck in Bhamo and boosted the rapid development of local economy, which was highly praised by the Myanmar Government and people. We followed the national strategy of “carbon peaking and carbon neutrality” and quickened the pace of green and low-carbon energy transition all over the world. Advancing the Green “Belt and Road” Initiative in a coordinated manner is of great strategic significance to

opening wider to the world and going further in green and low-carbon development. We took the lead in the establishment of Hainan International Carbon Emissions Exchange, China's first carbon market labeled with "internationalization". In this way, CDT deserved to be a example for exploring the introduction of foreign investors and the connectivity and trade with the international carbon market, pursuing a new round of high-level opening-up and high-profile international cooperation, and realizing goals of "carbon peaking and carbon neutrality". With promoting the realization of the goals of "carbon peaking and carbon neutrality" as the vision and ensuring the safe supply of energy and electricity as the front burner, we deepen the cooperation in the field of global energy transition, guided the way for international cooperation on climate governance, infused more certainties into the uncertain energy market, and pushed forward the joint construction of the "Belt and Road" Initiative to make progress in both depth and breadth in the energy field. We unveiled sound science and technology innovation system and stepped up the efforts to break through in key core technologies. CDT launched science and technology innovation project, made technological innovation system more well-organized, set up Science and Technology Advisory Committee, bolstered talent pool, appraised "Chief Expert/ Top-notch Young Science and Technology Talent" (the First Batch), implemented "Excellent Engineer Cultivation Program", improved the "methodology" of science and technology innovation assessment indicator system, and rolled out more original achievements from scratch. We performed social responsibilities and set a positive example for building a community with a shared future for mankind. We innovated and established characteristic assistance system of "Three-Support and Three-Assistance, and FiveSphere Integrated Plan", blazed a path for enabling rural revitalization with industries, diversified channels for construction of "Beautiful Countryside" and consumption assistance, broadened the employment channels for people lifted out of poverty, and made every effort for rural revitalization with down-to-eart and effective measures. We strived to let the majority of the public along the "Belt and Road" share cooperation benefits. Cambodia Stung Atay Hydropower Project changed six-kilometer open diversion channels into underground culverts, effectively protecting the local primary forest ecosystem. Datang Indonesian Power Plant launched "Longterm Ecological Protection Plan for Coral Reef Restoration" in nearby waters and initiated a fund to help the locals develop an awareness of sustainable development and maintain marine ecological health. We explored CSR Practice Road for medical assistance, cultural exchanges, education donation, skills training and infrastructure construction, vigorously improved the well-being of the people in the host countries, and joined hands with countries and regions along the "Belt and Road" to build "a Community of All Life on Earth".

"From the perspective of overall situation, we should know which direction we should go; From the perspective of general trend, we should make our mark". With outlook on bright future, CDT will endeavor to grow into "China's best and world class", seize good opportunities, follow the trend to take action, seek development while maintaining stable progress, improve quality in the development trajectory, write a new brilliant chaper on "the Second Startup", comprehensively promote green energy cooperation under the "Belt and Road" Initiative, and make contributions to global sustainable development.

Zou Lei Chairman of China Datang Corporation Ltd.

Caring for Employee Development

Cultivating and nurturing talents is the foundation of a nation. CDT is committed to fully

implementing the strategy for invigorating China through education in the new era, paying full attention to the realization of employees' self-worth. At the same time, by adhering to the fundamental requirements of "enterprise's development by the employees", we are continuously advancing the construction of "Top Six" livelihood projects, striving to build the "Happiness Datang", and constantly enhancing employees' sense of gain, happiness, and security. These efforts provide robust intellectual and talent support for the high-quality and sustainable development of the Group.

16.Longrich

President's word:

Welcome to Longrich,

China's FMCG market is growing fast in parallel with its economic boom. The complexity of today's buying behavior of the global consumer has increased the need for firms to implement effective and stronger marketing strategies that meets customers' expectations both in terms of quality and added value.

The past 30-year growth history of Longrich has left us cherished traditions that features a spirit of dedication, diligence and perseverance in everything we do.

In 2014, we took a small step forward towards differentiated competition and have made breakthrough in stock keeping units. Today, we are so proud to know that our products are gaining the trust and recognition from millions of people not only in China but also abroad. Today we continue to invest many efforts into the development of more innovative and high quality products so to meet that even higher expectation from all our clients.

With the guidance of our Chairman, we will develop this year a "new innovative-driven growth strategy". To be able to do so, we will focus on value management and identify our differentiated strengths in products and services in an effort to realize our unique advantage. We will diversify and improve our information and communication system, streamline internal process, budget control so to upgrade our management efficiency.

We firmly believe that the rigorous, efficient and effective internal management system that we will implement will enable us to stand and position ourselves into this fierce competition of the beauty industry.

Furthermore, we will keep improving customer's experience by combining experiential marketing and internet marketing together, thus providing a more convenient online and offline shopping environment for all our customers so as to maintain and increase our market share, size and growth opportunity.

I wish Longrich customers, partners and employees fulfilled dreams, good health, great happiness and well-being in 2016. We will strive relentlessly to fulfill our mission of being committed to a healthy and beautiful lifestyle.

Thank You.

Company Profile

Longrich is a leading Chinese cosmetic company that holds a wide variety of household cleaning products, personal care products, health care products and pet care products.

Longrich Bio-Science Co. Ltd, (Longrich), which is located in the Longrich Bio-Industrial Park, is one of the leading companies that dominates in the production of Health Care, Cosmetic/Skin Care, Household Cleaning, Furniture, Real Estate business and many more products.

Our trademarks have been registered in 183 countries and our products are sold in more than 50

countries and regions around the world including Malaysia, Taiwan, Hong Kong, Singapore, Ghana, South Africa, Cameroon, Russia, Ukraine, South Korea, Thailand, Philippines, United Arab Emiratesa and in USA.

We have 8 research and development centers in three continents and over 20,000 employees worldwide.

Jiangsu Longrich Bioscience Co., Ltd, known also as ‘’ Longrich (Chinese: 隆力奇， pinyin: Lónglìqí) ’’ is so far China’s largest and the most technologically advanced firm specialized in the development and production of daily chemicals as well as healthcare products and a company that legally practices the direct selling in mainland China and abroad since 2009. With our OEM/ODM capabilities, we export products to more than 60 countries and regions around the world.

We employ nearly 10 thousand people, including undergraduates students, postgraduates, and PhD students which account for more than 35% of the total staff. Every year, just about one thousand foreign talents are introduced to the company. Meanwhile entrepreneurship platforms are constantly built. Today, 3500 people already begun to give shape to the creative business incubator established.

Since our foundation in 1986, Longrich has been gone through a great and steady development so to become today a world famous company of cosmetic brands and health care industry. An investment of 600 Million RMB was made to build China’s most advanced and world class intelligent factory.

At the end of 2015, Longrich had established overseas branches and have representatives in more than 30 countries and regions. Following the One Belt, One Road strategy, the company plans to link and expand in the near future its business in 65 countries and hopes to complete full market coverage of 100 countries by 2020.

Longrich actively promotes the "Made in China 2025" project, an initiative to comprehensively upgrade Chinese industry. The initiative draws direct inspiration from Germany's "Industry 4.0" plan. The Ministry of Industry and Information Technology of the People’s Republic of China recently launched the China 4.0 pilot company project which consists of identifying exceptional manufacturer.

In the next 20 years, Longrich will explore industrial 4.0 firms in Southwest and North China, Guangdong, South-East Asia, Africa, as well as the Americas. Through output-supply chain management experience and technology, the firm plans to invest and build 10 smart factories in China but also abroad. As for the development of custom made brands, Longrich will put great effort to build alliances with the industry’s experienced and outstanding firms and together form a joint-stock company, strive and get listed within 3 years.

In order to have access to a wider international market clients, Longrich has successfully passed

the IS09001, IS014001, IS022716: 2007/G-MPC (American Standard), BRC and has set up 8 R&D institution including Longrich Postdoctoral Research Center, Jiangnan University Longrich Joint Research Center for Functional Food, Longrich (USA) Health and Cosmetics Research Institute, Longrich Postdoctoral Research Laboratory, Longrich (Japan) Beauty and Health Innovation Center Longrich Academician Laboratory, Tsinghua University （College of Life Science) Longrich Biology Institute as well as Longrich French R&D Center.

During the French cultural activities, Longrich products were the only cosmetics gifts designated for the French guests. Longrich has also been for many years one of the top ten and leading cosmetics enterprises in China with products such as " Longliqi snake oil, Evergreen, hand cream, etc… and 17 series of products were given the price of ‘’ outstanding products Award ‘’ and were presented at the achievement exhibition of the 60th anniversary of the foundation of the People’s Republic of China.

Our trademarks have been registered in 138 countries and have branches in over 30 countries and regions around the world including Malaysia, Taiwan, Hong Kong, Singapore, Nigeria, Ghana, Gabon, South Africa, Cameroon, Russia, Ukraine, South Korea, Thailand, Philippines, United Arab Emirates as well as the United States.

Longrich will continue to seize opportunities and keep going with its mission to providing a healthy and beautiful lifestyle to all the families around the world, constantly improve the technological content, the quality and added value of products dedicated for the global consumers, speed up the comprehensive promotion of its brands, furthermore, accelerate and spread the corporate culture ‘’ Better life, Better future ’’, its brand visibility and reputation across all countries.

Mission & Vision

To be World's Best Health and Beauty Solution Provider

Committed to a Healthy and Beautiful Lifestyle

Value

Diligence

Perseverance

Passion

Continual Improvement

Collaboration

Change

Innovation

NEW HIGH-TECH PRODUCTS IN JIANGSU PROVINCE

090581G0150N

Nanotechnology Coenzyme Q10 Capsule Emulsion

090581G0151N

Resveratrol Phosphatide Complex Nano-emulsion

100581G0332N
Ceramide Nano Liposome Emulsion

100581G0333N
Eyes Anti Dark Circles Medicine

110581G0022N
Emulsifier LCE

120581G0347N
Highly Active Antioxidant Enzyme Preparation

120581G0803N
Natural Herbal Antibacterial Against Mites

130581G0061N
Complex Active Enzyme Inhibitor of Plant Compound Amino Acid
Involved in Municipal, Provincial, and National projects
Jiangsu Science and Technology Support Project
Research and Development of New type of Phospholipid Glutathione Peroxydase

National Spark Project
Research of Key Technology Of Industrial Processing of Raw Nutritious Vegefruit Instant Drink, and the Development of its Product

Suzhou Science and Technology Agricultural Support Project
Research of Key Technology Of Industrial Processing of Raw Food

Jiangsu ‘’ Yinzhi ‘’ Project
Research and Development of Natural Organic Cosmetics

China 863 National Project
Research and Development of New type of Phospholipid Glutathione Peroxydase

Jiangsu Scientific and Technological Achievements
Application of Nanotechnology in Skin Care Products

National Torch Project
Industrialization of Nanotechnology Coenzyme Q10 Capsule Emulsion

National Torch Project
Development and Application of Crystal Liquid Emulsifier and Industrialization
Awards
Research and Application of Lipid Nanocapsule Technology

Science and Technology of Progress Award of China National Light Industry Council

Research of Key Technology Of Industrial Processing of Raw Food
Science and Technology Award of Chinese Cereal and Oil Association

Research of Key Technology Of Industrial Processing of Raw Nutritious Vegefruit Instant Drink
Science and Technology of Progress Award of China National Light Industry Council

New Bio-enzymes Toothpaste
Suzhou Science and Technology Progress Award

Research of Key Technology Of Industrial Processing of Raw Vegefruit Instant Drink
Changshu Science and Technology Progress Award

New Bio-enzymes Toothpaste
Changshu Science and Technology Progress Award

Nano Snake Oil Emulsion
Changshu Science and Technology Progress Award

A Kind of Chinese Medicine Composition with Whitening Function and its Application
Changshu Outstanding Patent Award

A kind of Red Special Diet Instant Drink for Women and its Processing Method
Changshu Outstanding Patent Award
PATENTS
ZL200410065250.X
Easy Cleaning Moisturizing Body Wash

ZL200410065249.7
Hair Conditioner

ZL200410065697.7
Anti-Bacterial Hand Protecting Detergent

ZL200510094951.0
Nanometers snake oil skin care

ZL200510095229.9
Neutral Moisturizing Soap

ZL200510094699.3
A Kind of Anti- bacterial Anti- itch Liquid Chinese Medecine

ZL200710131826.1
A Kind of Lip Balm

ZL200810024411.9
A Chinese medicine composition with whitening function and its application

ZL200710131827.6
A Kind of Soap

ZL201010200068.6
A Kind of Water Purifier Machine

ZL201110160185.9
Preparation Method of an Antibacterial Hand Washing Products Containing High Level of Alcohol and no Rinse Bubble

ZL200610012008.5
Multifunctional Enzyme Adding Striped Toothpaste and its Preparation

ZL200610097166.5
A Kind of Natural Plants Talcum Powder

ZL200610114598.2
A Kind of Snake Oil Nanocapsules Lotion and it’s Preparation

ZL200710063828.1
A Kind of Shampoo Contained with Snake Gale and Herbal Medicine

ZL200710304525.4
A Kind of Lanolin Nano-Emulsion and its Preparation Method and Application

ZL200710304523.5
A Kind of Coenzyme Q10 Nanocapsules Lotion and its Preparation Method and Application

ZL200710304524.X
A Kind of Ceramide Nano-liposomes Preparations and its Preparation Method and Application

ZL200710063829.6
Linseed Oil Microencapsulated Powder and its Preparation Method

ZL200710063485.9
A Kind of Concentrated Emulsion of Nano Emu oil

ZL200910084272.3

A Kind of Nano Emulsion of Resveratrol Compound and its Preparation Method and Application

ZL200910088890.5
A Kind of Multifunctional Natural Mouthwash and its Preparation Method

ZL200810106147.3
A Kind of Self-Emulsifying Vitamin C Preparation Method and its Application

ZL200810114872.5
A Kind of Anticarious Two-Component Toothpaste with Chinese Herbal Medicine and its Preparation Method

ZL201010282285.4
A Kind of cold Raw Nutritious Vegefruit Instant Drink and its Production Process

ZL201110098833.2
A woman surnamed people special dietary red salt drinks and processing method

ZL201010599432.0
A Kind of Antioxidant Combination

US008465757B2
Resveratrol Phospholipid Nano-emulsion Preparation and its Application
Published papers
Preparation and Characterization of a Lecithin Nanoemulsion as a Topical Delivery System，Nanoscale Research Letter，2010，5：224－230（SCI 2.894)

The advantages of a novel CoQ10 delivery system in skin photo-protection[J]， International Journal of Pharmaceutics， 2010，392：57-63（SCI 2.962）

Characterisation and skin distribution of lecithin based coenzyme Q10-loaded lipid nanocapsules，Nanoscale Research Letter，2010，5：1561－1569（SCI 2.894)
Social Responsibilities
Ensuring Health for All:
We bring and share wellness with thousands of families around the world. We are focused on providing to our consumers a high standard and healthier life condition.

Positive Impact:
Every partner we have and our clients all are actively involved in our sustainability plan. We are proud to assist organizations that develop projects which reflect mission and vision of our company.
Longliqi were certified ISO9001 in 1998 and ISO14001 in 2000 respectively. In 2005, we passed through HACCP certification.
Quality & Safety

100000 grade purification workshop, effectively guarantee the product quality
Engineering Quality Laboratory
Charity
17.Lenovo
Lenovo's story has always been about shaping computing intelligence to create a better world. With the world's widest portfolio of technology products, we deliver our vision of Smarter Technology for All through products, solutions, software, and services that individuals, communities, businesses, and entire populations need to fulfill their potential.
Today, industries are reshaping and reimaging what they make and how they do it. Virtual healthcare. Remote education. Smarter shopping. Smarter technology must be made for all. As a Fortune Global 500 technology company and one of Fortune Magazine's World's Most Admired Companies, we think and act globally. Breakthroughs aren't born in a bubble, and it takes all kinds of people and points of view to make technology smart. We're proud to leverage the diversity of our more than 63,000 employees to serve customers in 180 markets to redefine and stretch the limits of what technology can do.

We believe we can impact society most when we work together. That's why we have committed to the GSMA Digital Declaration that identifies shared values across the technology industry for an ethical digital future.

And that starts with making sure all voices are heard within and outside or our company. We've signed on to the Valuable 500, which aims to:

Increase representation of diverse voices in the design of products and solutions
Use our scale and global presence to raise awareness and champion inclusivity
Drive ambitious goals and targets for the business around Diversity and Inclusion in all its forms

Our Company
What makes us capable of leading Intelligent Transformation? Our credentials.
Full Portfolio
We deliver a full portfolio of PCs and tablets, monitors, accessories, smartphones, smart home and smart collaboration solutions, augmented and virtual reality (AR/VR), commercial Internet of Things (IoT), software, services, and smart infrastructure data center solutions around the world. Since 1995 we have shipped more than half a billion PCs, and we make three devices every second. Our Lenovo Neptune™ water-cooling technology for high performance computing (HPC) environments has made us the #1 provider of systems on the global TOP 500 supercomputer list. Lenovo has been included as one of the initial members of the new Hang Seng TECH Index, making us one of the 30 largest qualifying tech companies currently on the Hong Kong Stock Exchange.

We understand transformation
Just as we've transformed our business over more than three decades we have helped countless organizations, from healthcare, education, retail, manufacturing, logistics, professional services, and more, re-think the way they use technology to revitalize their business through smarter

solutions that leverage hardware, software, and services.

Global scale and manufacturing

We serve more than 180 markets, and we own the majority of our facilities, giving us unrivaled scale, efficiency, and control of our supply chain. Our global manufacturing allows tailored offerings to regional markets and includes more than 30 manufacturing facilities, including in-house, joint venture, original design manufacturer, and contract manufacturer sites in Argentina, Brazil, China, Germany, Hungary, India, Japan, Mexico, and USA.

We manufacture the majority of our products in our own facilities — more than most other hardware suppliers. This hybrid model helps us bring new innovations to market efficiently while having greater control over product development and supply chain for advantages in quality, security, and time-to-market. Recently, Gartner ranked us #15 on their list of Top 25 World Class Supply Chains.

Our Culture

When you come to work at Lenovo, you step into an environment built on respect for the people. We are exceedingly proud to be a truly global citizen. Our diverse team of people and offices means greater collaboration and sharing across borders, so we are exposed to the best practices in every sector.

Employee Engagement

Employee engagement surveys are a good way for us to better understand our workforce – we know that engagement is a measure of productivity, inclusion, and a predictor for retention. In our annual Lenovo Listens employee engagement survey, employees share their perspective on how we’re doing with inclusion measures at the company.Employee Resource Groups (ERGs) play an instrumental role in advancing the employee experience and create communities of shared identity.

Women in Lenovo Leadership (WILL)

More than a decade ago, Lenovo recognized the need to support women in theworkplace, and a small group of female executives created Women in LenovoLeadership (WILL) as its first ERG. WILL has physical chapters located in all five of ourgeographies: China, Asia-Pacifc (AP), Europe, Middle East & Africa (EMEA), LatinAmerica (LATAM), and North America (NA). Countries with chapters include Mexico.Brazil, Argentina, the U.S., Canada, the UK, France, Norway, Sweden, Germany,Australia, and China, among others. WILL focuses on supporting and cultivating women's careers at Lenovo.

Diversitas

Diversitas was launched in Bratislava by employees who are passionate about making Lenovo more inclusive. At the 2017 European Diversity Awards hosted in London.Diversitas won Outstanding Employee Network, beating out thousands of other nominations. lts members raise awareness about cutting-edge topics around LGBTQ+disability inclusion, and immigration. Diversitas hosts 15 events annually, including celebrating Diversity Month with an in-office Pride Parade and Diversity Fair, collecting charitable donations, and delivering Human Resources training.

New and Expectant Mothers Outreach (NEMO)

To support working moms, the New and Expectant Mothers Outreach (NEMO) ERG in North

America helps new and expectant mothers through community support and mentorship. This group focuses on creating a supportive network of moms who help foster a smooth transition into the world of balancing work and motherhood.Additionally, the team helps improve workplace conditions in collaboration with local facilities teams, by securing additional parking space for new and expectant mothers, aswell as dedicated space for private nursing rooms.

Black Leaders Achieving Success in Technology (BLAST)

Launched in 2014, BLAST serves more than 300 African American employees across North America. The group strives to inspire, support, and empower its leaders through enlightenment, exposure, mentorship, and outreach. lt provides regular career development opportunities, including its 6-month “Empower" mentor program. BLAST also regularly hosts speaker panels for best practice insights, recognizes member achievements and promotions, and partners with other ERGs to give back to the community through the Lenovo Foundation.

Hispanics of Lenovo Association (HOLA)

Launched in October 2017, HOLA focuses on empowering, developing, and advancingthe next generation of Lenovo leaders of Hispanic/ Latino descent. lt facilitatesroundtable discussions led by executive sponsors, giving employees an opportunity toshare their experiences around networking, professional development and work/lifeintegration. HOLA also hosts an annual diaspora multicultural event celebratingHispanic Heritage Month in September.

Pride

With the 2014 acquisition of Motorola, Lenovo became more involved in the LGBTO community. Our Motorola Chicago office supported the LGBTQ ERG and participated in Pride Month in Chicago and San Francisco. The Lenovo Foundation helped fun support of StartOut in Chicago, a non-profit that promotes LGBTQ equality. Lenovo launched its LGBTQ group for North America with a panel discussion in June 2018. The conversation brought together allies and LGBTQ leaders in the organization to talk about their experiences and their role in attracting top talent.

Lenovo Employees of Asian Descent (LEAD)

Established in 2019, LEAD celebrates and promotes the heritage of each and everyAsian ethnicity within Lenovo, assisting in developing the full potential of Asian Lenovoemployees professionally while engaging and giving back to the local Asiancommunities. LEAD also serves as a resource in assisting Lenovo to achieve its goal of adiverse and inclusive working environment.

Rising Employees at Lenovo (REAL)

Established in 2020 REAL is dedicated to building a community of tomorrow's leaders by accelerating the growth of Gen Z early career professionals and connecting them with the leaders of today.

A Better Lenovo for Everyone (ABLE)

Lenovo's newest ERG was launched in April 2020. ABLE's mission is to offer a community of support for Lenovo employees with disabilities, or colleagues who support family or friends with disabilities at home or are passionate about being allies for the disability community. That support comes in the form of guidance for resources,navigating benefits, and supporting community organizations with shared values.

Earlier this year, Lenovo EMEA launched its own regional ERG for disabilities called PwD (People with Disabilities), with 7 countries represented among its leadership committee. An AP chapter of the disability ERG is set to launch in 2021.

Lenovo Interfaith

Launched in Brazil in 2020, Lenovo lnterfaith employee resource group focuses on employee communities across religions and promotes religious tolerance in the workplace.

Committed to Social Responsibility

We strive to do things differently so that we can consistently become better—for our customers, our employees, the communities where we do business, and the environment.

Environmental

Social

Human rights

LABOR PRACTICES The Company’s Human Rights policy communicates its respect for human rights and how it extends those rights to employees and business partners. As a signatory of the UNGC, the Company’s practice is to uphold and support the protection of internationally proclaimed human rights. The Company does not permit the use of child labor, forced labor or coercion, including physical punishment, in any of its operations. The Company’s Human Rights policy is committed to the following practices: • Conduct business in accordance with the U.N. Declaration of Human Rights and the principles of the UNGC and extend those requirements to all suppliers doing business with the Company. • Perform due diligence across the value chain to identify risks and avoid complicity in human rights violations. • Provide access to grievance mechanisms, investigate allegations, and escalate known cases of human rights abuse to senior leadership. • Integrate training and accountability for respecting human rights across the business. • Engage internal and external stakeholders to address common challenges and advance human rights practices through continuous improvement. • Operate legally and ethically in each country where it does business.

All of the Company’s corporate strategies, practices, and guidelines as well as supplier requirements must support this commitment to human rights. The Company is not aware of any cases of child labor or forced labor at its facilities. Concerns about possible human rights violations must be reported to the Company’s management who shall take prompt corrective action. More information is available in the Company’s Human Rights Policy. The Company is determined to ensure that the working conditions at its locations are safe; workers are treated with respect and dignity; operations are environmentally sound; and business operations are conducted responsibly and ethically. The Company aims to raise awareness by engaging with the Responsible Business Alliance (RBA). As of FY 2021/22, all Lenovo owned and joint venture manufacturing sites (not including new locations with less than one year of labor data) have undergone audits by independent auditors using the latest version of the RBA Standards, which are based on the International Labor Organization (ILO) Standards and include a review of child labor and forced labor processes at each facility that is audited. The auditors also review employee files and conduct individual and group interviews. Labor practices are also evaluated as part of the scope of two main processes within the Company’s business management system. These include the Company’s global risk registration process as part of its Enterprise Risk Management (ERM) and the ESG reporting materiality assessment. The detailed processes may vary by market and are based on local laws.

DIVERSITY, EQUITY AND INCLUSION (DEI)

A Message from our Chief Diversity Officer

As a global technology leader, Lenovo touches the lives of millions of people from all walks of life,

from our global workforce to the customers we serve with experiences and solutions. We are ushering in a bold new era of Intelligent Transformation, and big data, 5G, and AI will deliver new models for how we live, work and play. In an ever-changing and increasingly diversified world, technology has the potential to serve as the great equalizer. It can provide more opportunity, greater connectivity, and the ability for populations to transcend historic, cultural and geographic limitations. We believe that technology companies have the capability and the profound responsibility to champion diversity and inclusion. Not only in the products and services we provide, but also in the values we foster and our internal practices. Innovating for a diverse world requires a diversity of perspective. As such, diversity is a business imperative at Lenovo. It ensures that we not only embrace the best, most disruptive ideas, but it also allows us to better understand and address our customers' needs. Lenovo has always pursued a path of inclusion and we've built a culture where all can belong – but our work is not done. With new research, data, and insights, we continue to evolve our approach of building inclusive leadership behaviors and fostering diverse and inclusive systems through increased accountability and training. Our goal is simple: People should no longer have to change themselves to fit the world. Instead, the world will change to fit its people – all of its people. Technology can and should be a positive catalyst for change and evolution. Lenovo is channeling this capability to build a smarter future, where everyone thrives, together.

DEI Culture

Maintaining a diverse culture and achieving its full potential is fundamental to the Company's competitive success. A key element in the Company's workforce diversity programs is the commitment to equal employment opportunity and to prohibit discrimination, harassment, and similar inappropriate behavior in the workplace. The Company's policy and Code of Conduct commits to providing a work environment free of discrimination and harassment based on race, color, gender, religion, age, nationality, social or ethnic origin, sexual orientation, gender identity or expression, marital status, pregnancy, disability, or veteran status. Company policy prohibits management from making employment decisions based on such characteristics. These business activities and the design and administration of the Company's benefit plans must comply with all applicable laws. For qualified employees with disabilities, the Company will make reasonable accommodations needed for effective job performance in a manner that complies with applicable laws.

"Inclusive Leadership is an intentional everyday act of empathy and creating a sense of safety by engaging in active listening and allyship that ensures individuals are respected, valued, and represented." Definition of Inclusive Leadership at Lenovo

Diversity and inclusion have been the building blocks of the Company's history and are among its greatest strengths. Its diverse team of people and locations enables collaboration and sharing across borders and encourages the Company to adopt the best practices in the markets it serves. The Company is bringing awareness about inclusion to all its leaders and employees in a variety of ways, including Global Anti-Harassment training to ensure a workplace free of harassment. The Company is conducting a global campaign with 100 percent target completion rate for Global Anti-Harassment Training.

A diverse business model starts at the top. The Company's leaders throughout the world hold a deep commitment to these values that fuel long-term growth. The Company believes that a global workforce should reflect the global customers that it serves, and this begins with

leadership that is representative of the various cultures and ethnicities that comprise its internal talent.

The Company has also taken a step forward in advocacy for women by endorsing the United Nation's Women's Empowerment Principles (WEPs). These principles offer guidance for businesses to promote gender equality and women's empowerment in the workplace, marketplace, and community. While the Company works toward gender equity, it understands that reducing bias and increasing gender equality is an effort that will require its total commitment. The Company is proud to join its fellow signatories and UN Global Compact members in this initiative. The Company's DEI strategy also prioritizes inclusive behaviors amongst its employees. In 2021, the DEI team launched the CARE Model for Inclusive Behaviors, defining and encouraging four behaviors to foster inclusion in the community: communicating across differences, acting in allyship, recognizing and mitigating bias, and ensuring psychological safety.

Inclusive and Compassionate

The Company continues to drive positive, meaningful change by embedding disability inclusion in the business leadership agenda. Since becoming a member of the Valuable 500 in 2020, the Company continues to partner with disability rights advocates to help ensure its products and solutions are inclusive and accessible. The Valuable 500 is a global business collective of 500 CEOs and their companies who have pledged to work together as a collective to drive systemic change. Since launching the Product Diversity Office (PDO) in 2020, the Company continues to scale up capacity to ensure its products are empowering its vision of delivering smarter technology for all – regardless of a user's physical attributes or abilities. The PDO's mission is to ensure usability for a diverse customer base and minimize any inherent bias in the Company's technology or products. It was founded with the support of LEC members and governed by the PDO Task Force, a group of business leaders who help to drive the PDO's strategy and influence across the business.

In addition to ensuring accessibility of the Company's products, the Company launched a People with Disabilities internal awareness campaign on Global Accessibility Awareness Day in 2021. The program works to create a stronger understanding of disability in its workforce through internal surveys. While the program began in the United States, the DEI team seeks to expand it to eight markets around the world by 2025. Much of the understanding and awareness for people with disabilities is driven with the help of the Company's ERGs, People with Disabilities and ABLE (A Better Lenovo for Everyone).

'Lenovo Listens' Employee Engagement

As the Company works to build a culture of inclusion, employee feedback is one of the best indicators of success. The Company seeks the insights of its employees worldwide through its annual 'Lenovo Listens' employee engagement survey. The survey is designed to measure employee sentiment and capture feedback on the Company's performance as it works to create an equitable and inclusive work environment. The 2021 survey results revealed that overall employee participation reached an all-time high of 94 percent.

Results also indicated confidence in the Company's future rose to 90 percent, representing an incredible 10-point increase since 2020. While the Company's greatest strengths continue to be its ethical business practices, incredible teamwork and its continued commitment to diversity and inclusion, the key takeaways from the 2021 Lenovo Listens survey are included in the graphic

below*

Health and Safety Awareness and Communication The Company fosters a philosophy that values a health and safety culture. Employee participation is essential to the success of health and safety management. Employee awareness is carried out through online tools that provide appropriate training and site-specific safety information. Training programs are also conducted during monthly team meetings and some manufacturing sites conduct annual Health and Safety Week or Safety Month to further encourage awareness.

WORK FOR HUMANKIND

So, in early 2022, Lenovo sent volunteers from around the world with a range of skills to support vital community and conservation projects on the island. After the volunteering phase was completed, a study revealed progress against the three objectives of the project:

1.Connecting an isolated community to the global economy Bridging the ‘digital divide,’ Work for Humankind aimed to bring high-speed connectivity to the remote island community, helping advance its education, healthcare and ability to protect its unique ecosystem. Lenovo’s Work for Humankind is a bold initiative in partnership with nonprofit organization Island Conservation and the Robinson Crusoe Island community that took volunteers from around the world to experience first-hand how to make a long-lasting difference with an island community, while working from Lenovo’s high speed technology hub. Born out of Lenovo’s research which showed a global desire for remote work, while supporting local communities and giving back, Lenovo was inspired to leverage its technology and partner with Robinson Crusoe Island. As one of the most ecologically rich places on the planet, Robinson Crusoe faces many challenges in a changing world, including access to education, healthcare, the impact of habitat degradation, and invasive species. Local efforts have been made to tackle these issues, however, they have been hindered by telecommunications and technology challenges.

2. Transforming local conservation projects that protect rare species and ecosystems The conservation results from Work for Humankind have helped Island Conservation and local conservationists achieve years’ worth of work in only weeks: • Increased endangered species protection for seven critically endangered species, including the Pink Footed Sheerwater, in the Juan Fernandez Archipelago • Lenovo’s AI server enables field staff to detect invasive species more efficiently. A process to analyze hundreds of thousands of images from camera traps that previously took months now takes days.

3. Bringing new skills and knowledge to the Robinson Crusoe community Nearly 1,000 hours of professional volunteer expertise advanced the community’s vision of a sustainable future. This resulted in: • Self-determined initiatives to increase the resilience of local food supplies and improve plastics and waste management • The island community now being empowered with digital tools and strategies to effectively engage with the global economy, enabling remote and hybrid work opportunities that previously did not exist.

Leaving a positive lasting legacy Looking ahead, Lenovo is committed to leaving a positive legacy on the island: • The tech hub will migrate to the local library to provide residents with access to new educational and digital tools. • Lenovo is also donating a US$100,000 grant for tech hub maintenance, ongoing project support and internet access for vital services to improve healthcare access and education.

Ethics and Integrity

The Company is committed to conducting business legally, ethically, and with integrity. Its Ethics

and Compliance Office (ECO) oversees the ethics and compliance function across the organization and strives to promote a culture that is committed to ethical business conduct. The ECO works in partnership with business units across the globe to promote legal and ethical operations. The ECO is committed to raising awareness about the importance of ethical and compliant business practices to the Company and serves a critical role in providing employees with the information, resources, and training they need to make informed ethical decisions. The ECO oversees Lenovo's Code of Conduct (Code), which establishes clear expectations for employee compliance with its policies related to lawful and ethical business conduct. The Code reflects the Company's culture of trust and integrity and holds employees accountable for their behavior and helps employees determine when and where to seek advice. The Code, policies, and related awareness and training materials are provided electronically and through periodic communications. The ECO is supported by various committees. The Executive Ethics Committee provides executive-level oversight and guidance to the ECO. The Investigation Oversight Committee works closely with the ECO to oversee the Company's internal investigation process. The Regional Ethics and Compliance Committee provides the ECO with global support, perspective, and insight. The Audit Committee also oversees the ECO and receives regular updates about the program priorities and initiatives.

BUSINESS PRACTICES

The Company's Code of Conduct (Code) mandates compliance with applicable laws in markets where it conducts business. Its policies strongly support ethical and responsible business practices, including but not limited to:

Anti-Bribery and Anti-Corruption

The Company complies with the anti-corruption laws of the countries in which we do business. Its policy on anti-bribery and anti-corruption and the Company's policy on gifts, entertainment, corporate hospitality, and travel reinforce provisions in the Code and provide additional guidance regarding compliance with global anti-bribery and anti-corruption rules and laws. Its policy stresses that the Company will not directly or indirectly offer or give anything of value to any person, including government officials, to influence actions or to secure an improper advantage as defined by applicable laws.

Anti-Competitive Practices and Fair Competition The Company competes for business ethically and lawfully. The Code and policy on anti-competitive practices and fair competition forbid employees from engaging in anti-competitive practices, such as entering into an agreement or discussion that would result in setting prices, limiting the availability of goods or services on the market, or agreeing to boycott a customer or supplier.

Intellectual Property The Company values intellectual property as it innovates for the future. The Company expects employees to protect intellectual property and to respect the intellectual property rights of other companies and individuals. It secures its intellectual property by using patents, copyrights, trademarks, confidential information, related contract rights, and other applicable forms of legal protection. Employees are required to abide by their agreement with the Company regarding confidential information and intellectual property. Additionally, all employees are expected to contribute to the Company's innovation leadership. This includes submitting inventions and ideas to the Company's Patent Review Board for review and protection with the support of the Intellectual Property Legal team. Employees must also consult with the Company's Legal Department as appropriate to ensure the required rights and licenses

are obtained before utilizing any third-party proprietary materials. Employees are expected to obtain and abide by licenses or other permissions as appropriate, as described in the Employee Code of Conduct.

Privacy & Data Protection

The Company maintains a Global Privacy Program, which leads the organization’s commitment to responsibly using and protecting customer, consumer, employee and partner identifiable information. The Lenovo Global Privacy Program develops and maintains policies, processes, training, and other mechanisms and resources to ensure that the Company is in compliance with global privacy and related data protection laws and regulations. These policies and the Company’s commitments in this area are communicated to all employees via the Lenovo Privacy Basics course which new employees are required to take within 30 days of their employment with the Company, and on a recurring basis thereafter. It is the individual and collective responsibility of the Company’s employees and contractors to act in accordance with the requirements of the Company’s privacy and security policies and standards and to report privacy and security incidents/vulnerabilities in a timely manner. The Lenovo Global Privacy Program, Chief Security Office, Chief Information Security Office, and the Company’s product security teams maintain incident reporting mechanisms and work together to investigate, mitigate, and prevent privacy and security incidents that could impact the Company, its customers, users, or employees. Individuals may learn more about the Company’s product and website privacy practices by visiting https://www.lenovo.com/us/en/privacy/. The Lenovo Privacy Program may be reached at privacy@lenovo. com (or privacy@motorola.com). The Company recognizes the great importance of privacy to individuals everywhere – customers, website visitors, product users, employees – everyone. The responsible use and protection of personal and other information under the Company’s care is a core value. To ensure adherence to its privacy policies, principles, and processes, the Company maintains a global Privacy & Data Protection Program led by the Legal Department. The Privacy & Data Protection Program reports its progress regularly to the Company’s Chief Legal Officer and Chief Security Officer. In addition, the Privacy & Data Protection Program coordinates a cross-functional Privacy Working Group (PWG) comprised of key partners drawn from Information Security, Product Security, Product Development, Marketing, E-Commerce, Service and Repair, Human Resources, and other groups. The PWG meets several times per year and discusses the Company’s privacy policies, processes, legal developments, industry developments, and more. Key elements of the Company’s approach to ensuring meaningful privacy and data protection include:

•Monitoring global privacy and data protection legal developments and regulatory trends, and improving the Company’s privacy practices and processes • Harmonizing global privacy and data protection requirements into an organization-wide set of guiding privacy principles intended to drive how the Company handles personal information and certain other types of data, including developing and updating its privacy policies and procedures • Providing contractual support to ensure that risks associated with supplier and partner agreements include appropriate privacy and security terms; including assistance to the Lenovo Legal Center of Excellence (COE) in its efforts to update contract templates and improve privacy and security-focused contract addenda • Providing early input to product and service development teams by incorporating privacy checkpoints into formal product development plans, including privacy impact assessments, and conducting pre-launch privacy compliance reviews of products, software,

services, websites, marketing programs, internal systems, and supplier relationships • Responding to requests from individuals to review, correct, amend and/or delete their personal information • Coordinating the Company’s response to law enforcement and other government requests for applicable personal and user information • Developing and delivering privacy and data protection-focused training programs and working closely with the Chief Security Office (CSO), Corporate Information Security Office (CISO), and product security teams to timely identify and respond to privacy and data protection incidents • Maintaining an internal Privacy Program portal and other resources for employees to provide guidance, documents, contract templates, compliance checklists, and additional privacy and data protection resources for the Company’s community

INNOVATION

Innovation Through Research and Development

Innovation is one of the pillars for the Company’s long-term success and the most effective game changer in its industry. From smartphones to servers and everything in between, the Company creates technology capable of transforming the way we live, work, and play. The Company recognizes how much potential the future holds for technology, and it is motivated to build smarter solutions for the things that matter the most to its customers.

The Company accelerated its innovation over the past two years to provide immediate solutions and shape a more equitable and empowered future through smarter technology. The intensified commitment to innovation, underpinned by ESG commitments, is manifesting the Company’s vision to transform from a devices company to a global technology powerhouse that also includes services and solutions.

Innovation through Research and Development (R&D) has enabled the Company to focus on new and emerging IT architecture and drive its service-led intelligent transformation. In FY2021/22, the Company strategically increased its R&D professionals by approximately 5,000 and announced plans to hire an additional 12,000 while doubling its R&D investments over the next three years.

Definition of Inclusive Leadership at Lenovo

Health and safety

Diversity and inclusion

Labor practices

Philanthropy/community engagement

Governance

Ethics and integrity

Cyber security and data privacy Regulatory/compliance

Product quality

Innovation

18.TCL

TCL is a leading global tech brand in display panels, TVs, home comfort and mobile devices. Founded in 1981, TCL operates its manufacturing and R&D centers worldwide and has products and services in 160+ countries.

At TCL, we are dedicated to improving people's lives and experiences with our innovative

technology. Our products and services aim to solve problems, bring joy to families and friends, and create a safer and healthier world. We aspire to inspire and empower people to pursue greatness in their lives.

At TCL, we continuously strive to make a difference in society and the environment as responsible corporate citizens. By leveraging our innovative technology, TCL aims to facilitate the transition towards a more sustainable future for local and global communities.

Over the past 40 years, TCL has been redefining what’s possible with display technologies through constant innovations.At TCL, we believe in giving excitement and happiness to people through gaming, sports, entertainment and technology that they love.

Brand Attributes

Proactive

A brave and fearless change-maker who believes in exploring the higher stages of life.

Innovative

Think outside the box , approach problems by considering a range of different perspectives.

Intelligent

A profound understanding of people and the world we live in.

Fashionable

Not afraid of change and always keeping the freshest state， is an innovator standing at the forefront of the times.

Innovations

TCL has over 1000 R&D technicians and Smart Screen R&D centers worldwide in countries including China, the United States, Japan, France, and Poland.

TCL boasts state-of-the-art labs, including optical design simulation, testing, Pangu (Mini-LED), screen technology, picture quality algorithms, and automatic tuning. Accredited by CNAS, TCL also has energy efficiency, safety, and wireless performance labs, as well as internationally recognized TÜV Rheinland and Intertek testing labs.

For years, TCL has heavily invested in independent innovation, establishing a comprehensive layout, and becoming a leading innovator in domestic R&D. With a main focus on smart displays, Mini-LED backlight technology, audio and video quality, perception and interaction, product design and appearance, OS, AIoT, and audio-video communication security, TCL leads the way in technological breakthroughs.

EXTERIOR CRAFTSMANSHIP

Construction & Materials

TCL has always been committed to innovation in structural design and technology, and adheres to the design concept of "low carbon, environmental protection, and green". They develop and apply new green materials to enhance user comfort through technology.

In 2014, TCL launched the H9600, the world's thinnest curved TV with a European design, high-end materials, and craftsmanship, making it not only a technological product but also a piece of art in the home.

In 2016, the XESS X1 curved TV was launched as the world's first ultra-thin partition backlight control. The product's back panel uses magnetron sputtering aluminium foil brushed metal film material and 3D curved surface moulding, along with a built-in shielding plate, making it a naturally seamless piece of work.

In 2018, the X5 featured a 4.9mm ultra-thin bezel design with a comfortable viewing experience

thanks to its broad viewing angle and humanized curvature. The middle frame adopts a polished stainless-steel appearance through chemical electrolysis, while the chrome-plated steel rod base gives users a luxurious metal technology experience.
In 2020, the XESS A200Pro was launched as the world's first 55-inch rotating TV, breaking the limitations of traditional TV products.
In 2022, TCL launched the world's thinnest stainless-steel backplate mini led-OD0 backlight TV at CES, with a thickness of only 3.9mm, leading the ultra-thin ultimate experience, and winning the "2021-2022 Display Technology Innovation Award" at CES.

19.CNOOC International
Who We Are
Delivering energy for all
CNOOC International, the international division of CNOOC Limited, is a global energy company with operations in Asia, Africa, the Americas, the Middle East and Europe. Our successful growth as an upstream oil and gas producer has been achieved by consistently finding and developing world-class assets.

Our business is focused on safely exploring and producing from conventional offshore, unconventional and oil sands assets. As part of the CNOOC Group of companies, which span the entire energy supply chain, our size and capability create a competitive advantage that ensures we deliver long-term value. Our growth strategy is to safely and sustainably develop our high-quality portfolio to deliver energy for all.
Sustainability
Working Safely: A Core Value
Safety is a core value at CNOOC International and the success of every activity we undertake is measured on our ability to execute our work safely every day. Our employees and contractors strive to ensure our oil and gas production occurs without incident or harm to our people, communities and the environment every day. Safety is just simply a part of the way we work.
Our Executive Leadership Team is fully committed to supporting a safe workplace by providing employees and contractors with clear expectations about:
Safety awareness
Consistent work practices
Proactive hazard identification
Rigorous risk mitigation, especially for non-routine work
Recognition and support for safety performance
Ongoing safety training and resources
Occupational Health & Industrial Hygiene
The health and safety of our employees and contractors is our top priority.

At CNOOC International, we're committed to anticipating, recognizing, evaluating and controlling workplace environmental health hazards that may cause illness or impair the well-being of our employees, contractors or the community members who live or work near our operations.
Promoting Healthy Work Environments

We've developed an occupational health management program that works to promote healthy work environments, protect the health and well-being of employees and prevent occupational injuries and illnesses. This system assesses and manages individual and work environment health risks, and organizational health issues that may pose a risk to our business (i.e. transmittable diseases).

We've also established an industrial hygiene management program that anticipates, recognizes, evaluates and controls workplace environmental health hazards or stressors. The components of this program include:

- Hazard identification and monitoring
- Risk evaluation
- Control plan development and implementation
- Employee training
- Evaluation of controls

Environment

Responsibly developing resources around the world

Addressing Climate Change

CNOOC International responsibly develops oil and gas resources that provide the energy we need today and in the future. This responsibility also includes acting on climate change and reducing our carbon emissions. Global energy demand is expected to rise as incomes grow and millions of people around the world are lifted out of energy poverty. Given this, there is no easy solution to delivering energy for all while reducing Greenhouse Gas (GHG) emissions.

We recognize the management of GHG emissions is an important policy and business issue and we support a range of actions to improve our emissions performance. These include using energy efficient technologies in the construction of our facilities, pursuing energy efficiency and conservation measures across our existing operations, and making investments in new technologies and innovation to improve emissions performance for the longer term. For operating jurisdictions with carbon pricing like the UK and Canada, we also participate in carbon markets and invest in carbon offsets as part of meeting our compliance obligations.

CNOOC International believes that establishing the right policy framework is also an essential part of effective climate policy. We work with governments and other stakeholders encourage energy and climate change policies that are effective, encourage investment and innovation, and provide clarity for companies and the public alike.

Habitat Management

Building Healthy Ecosystems that Support Biodiversity

CNOOC International operates in areas of rich and sensitive ecosystems. We recognize that earning the social license to operate and grow our business is dependent on our ability to explore for, and develop, energy reserves without adversely affecting natural ecosystems and wildlife. As a result, we integrate ecosystem considerations into our business practices and operations. This helps us minimize risks and maximize opportunities as we work to make a positive contribution to protect ecosystems in all the areas where we operate.

Managing Water Use

Environmental technician using a Heron H. Oil interface metre that measures the thickness of floating or sinking hydrocarbon products in ground water at one of the many metering stations at CNOOC International's Long Lake SAGD oil facility

Water is a key ingredient that enables the development of hydrocarbon resources. We continuously look for opportunities to minimize our water use throughout our operations as we seek to deliver sustainable energy for all. We aim to do this through the use of innovative technologies, continuously monitoring our work, recycling water where practical and collaborating with industry partners and stakeholders.

Partnering on air quality monitoring

CNOOC International supports the work of the Wood Buffalo Environmental Association (WBEA), which monitors air quality throughout the Regional Municipality of Wood Buffalo – one of the largest municipalities in North America. WBEA maintains 25 fixed and three mobile ambient air quality monitoring stations throughout the region and provides ambient air quality data and an air quality index that are updated hourly.

In addition to the air quality monitoring station that is located in the community of Anzac – the closest community to our Long Lake oil sands facility in Alberta, Canada, we also collect air quality monitoring data through 15 passive air monitors. Air quality data from the Anzac station is fed on a real-time basis to Alberta Environment and WBEA.

In 2013, a Joint Canada-Alberta Implementation Plan for Oil Sands Monitoring was put in place and is funded by industry, including CNOOC International. The plan aims to provide world class monitoring of air, water and biodiversity for the oil sands region of Alberta. The intent is not to replace the air monitoring programs currently managed by WBEA, but rather to enhance and improve the scope and coverage of the programs.

Social Responsibility

Earning our social license to operate

At CNOOC International, how we work is as important as the results we deliver. We’re committed to partnering with community members and other stakeholders in the areas where we operate. We build long-term trust by sharing information, consulting with stakeholders about business decisions and working collaboratively to understand their needs and expectations.

We believe our approach to responsible development provides us with a competitive advantage by allowing us to maintain our social license to operate while also fostering support for our future growth.

Anticipating and managing the impacts our operations can have on our neighbouring communities and the broader society is at the heart of our approach to social responsibility. Our ultimate goal is to generate a net benefit for society as we pursue our vision: to deliver sustainable energy for all.

Taking the time to understand the issues important to our neighbouring communities and other stakeholders is the first step in social responsibility. We do this by engaging and consulting with

local community stakeholders, including First Nation, Aboriginal and Indigenous groups. Our operations benefit from local suppliers who know the community and environment best. We also work with local governments, businesses and residents to enhance their capacity to provide goods and services. We strongly believe in giving back to the communities where we operate to ensure our presence results in mutual benefits.

Building Mutually Beneficial Relationships

CNOOC International demonstrates respect for stakeholders through early, ongoing engagement and open dialogue. We align the interests of stakeholders with our values and business principles.

Our approach is based on the belief that all stakeholders have a legitimate right to know about our planned and ongoing activities, and to be consulted on issues that affect them.

We define stakeholders as any individuals or groups who could be impacted by our operations or who could, through their own actions, affect our business. Many people have a stake in CNOOC International:

Employees

Governments and regulators

Customers

Community residents and landowners

First Nations, Aboriginal peoples and Indigenous peoples

Special interest groups and non-government organizations

Investors

When determining the level of engagement required with stakeholders, we consider the size of the project or our operations, impact scope and the degree to which stakeholders themselves wish to be involved

Engaging with Integrity

Operating with integrity is a cornerstone of our culture and a key component of our engagement. Effective engagement plays an essential role in earning and maintaining our social license to operate by building trust-based relationships and establishing an open dialogue. In turn, this helps identify concerns, create opportunities and provide benefits to CNOOC International and the stakeholders affected by our business activities.

Respecting Universal Rights

We believe respecting human rights is a fundamental aspect of responsible energy development. Wherever we operate, CNOOC International’s commitment to respecting human rights remains the same. We strive to ensure business decisions are examined for their potential impact on human rights and by taking steps to ensure employees and contractors are never complicit in human rights violations. One of the ways we assess and manage human rights risk is through a process known as Above Ground Reviews (AGRs). These reviews assess a country’s political, security, regulatory and social issues through interviews with government officials, industry operators, journalists, non-governmental organizations and security specialists. In addition to conducting AGRs prior to considering an initial investment in a new location, updates are conducted on a regular basis.

Community Investment Benefiting the communities where we operate
Giving
CNOOC International ’ s Community Investment program helps strengthen the communities where we live and work. Through meaningful corporate gifts including donations and sponsorships, we prioritize funding for projects that provide long-term and sustainable results that have a positive impact on our communities.
Our funding priorities are:
Supporting Thriving & Safe Communities
We focus on helping individuals and families in need to access resources and opportunities to help them reach their full potential. In addition, we support initiatives that contribute to advancing safety and emergency response in the communities where we operate.
Advancing Education
Through scholarships and bursaries, we are helping to advance the educational attainment of young people in our communities. In particular, we focus on funding programs that are related to Science, Technology, Engineering and Math (STEM), to help build capacity and to positively impact our business and industry.
Capacity Building for Indigenous Communities
Supporting our Indigenous stakeholders in a key pillar in our Community Investment program. We focus on providing funding that enables capacity building, educational advancement and thriving communities.
Green and Low-Carbon Initiatives
We are committed to doing our part to protect the environment in which we operate. By implementing programs and technology focused on reducing carbon emissions, we are proactively responding to climate change challenges.
Employee Programs
Through our ReachOut program, we support the organizations and initiatives that our important to our employees. We match employee donations to charitable and non-profit organizations. We also support employee volunteerism in the community through volunteer days, volunteer rewards and sports grants.
Scholarships
CNOOC International believes in sharing the economic benefits of development with the people closest to our operations. We provide employment opportunities and source the goods and services we need from local suppliers when possible. In addition, we give back to communities by investing in programs and projects that enhance the quality of life for people in those communities. This includes providing post-secondary scholarships to support the educational attainment of people in areas where we operate.

20.Hisense
Aspiring to become the most reliable brand in the world with more than a century of brand heritage, we aim to continually pursue scientific and technological innovation in hopes to improve overall quality of life and bring happiness to millions of families.
Hisense started its import and export business in 1988, and formally participated in the Canton Fair in 1993 to open up the international market, Hisense Confirmed, that all resources should be spent priority to overseas markets, and that it should expand through OEM business to gain

channel resources and operational experience. while developing Hisense brand in key markets at the same time.
In July 2018, Hisense integrated product companies with similar industries, technologies or business modes to realise resource and management sharing and improve operation efficeiency. Three industrial groups included Hisense Electronic Information Group, Hisense Home Appliances Group, Hisense Intelligent Technology Group.
With the maturity of markets in South Africa. Australia, Europe, North America. Hisense established more branches in Germany, UK, ltaly, Spain, Canada, Mexico, Japan, Dubai. It established sound overseas marketing system,and realized marketing localization.
Pushing the boundaries of innovation since 1969.
Hisense built R&D Centres and factories around the world to encourage its designer and researchers to go abroad to hear local demands, to improve product quality , to upgrade technical level , to realize the localization of production,and support the optimization of local product quality, service and response.
Acquisition of TVS & Gorenje in 2018, form Multi-Brand Strategy and become official partner of EURO,FWC,PSG and Fnatic since 2016.

Here at Hisense, we believe that everything we do should make your life simpler, better and more entertaining. That’s why, it is our aim to make the latest technologies accessible to everyone through thoughtfully designed and expertly built products. For almost 5 decades Hisense have been committed to developing innovations in consumer electronics. Today, we are the No.1 TV brand in China, Australia & South Africa, employing over 75,000 people across 18 global companies. We also utilize 3 high-end international production facilities in Europe, Central America and South Africa, as well as boasting 12 research & development centres worldwide, including a strategic partnership with MIT Media Lab. All of which have the sole aim of delivering high quality, cutting-edge and affordable products that improve the life of our consumers.

Mission & Vision
Vision
To be a century-old company, and to become the most reliable brand in the world.
Mission
To pursue scientific and technological innovation, take the lead in the advanced manufacturing with intelligence as the core, and bring happiness to millions of families with high-quality products and services.
Our Core Value
We’ve always adhered to the core values of Integrity, Innovation, Customer Focus and Sustainability: Attributing our sustainable and healthy development to upright cadres and good corporate culture, attaching importance to R&D investment, prioritizing customer-centricity, and most importantly promoting precise urban governance and contributing to sustainable city development.
Corporate Social Responsibility
Hisense actively decorates to social public welfare and takes the initiative to assume social responsibility, which have been deeply rooted in Hisense culture and planted all over the world with the global development at Hisense brand.

Hisense's social welfare activities in South Africa

On Mandela Day, Hisense South Africa sent support to more than 700 lonely elderly people in local pension institutions, and donated televisions and daily necessities to the non-profit child care center in Johannesburg. Hisense South Africa also sponsored the underfunded Ethiopian Youth Summer Football Tournament to help 440 young people realize their dreams, and donated the South African Red Cross Children's Hospital and other projects to actively help solve education and employment problems in South Africa, North Africa, Middle East and other countries and regions. Moreover, this company has become the only enterprise partner of the United Nations Green Innovation SEED Awards and has provided support to more than 100 small and medium-sized enterprises in developing countries over the past five years.

Behind every great company, there is a great story.

Hisense S.A. (PTY) Ltd. entered the South African market in 1996. The company is based in Bedfordview, Johannesburg, and in Century City, Cape Town. Hisense has a national footprint with offices in the country’s 4 major centres, allowing the company to distribute our products to over 3,000 chain stores and 500 home appliance franchise stores. In addition, our products are exported to more than 10 countries in the neighbouring regions, including Namibia, Mozambique, Zimbabwe, Malawi, Zambia, Botswana and Lesotho. In June 2013, Hisense launched our R350 million, state of the art consumer electronics and home appliance manufacturing facility in Atlantis industrial park, Cape Town, which is capable to produce 400 000 refrigerators and televisions per annum. Our continuous efforts over the last 23 years have helped grow Hisense’s LED TV and refrigeration market share by 25% and 14% respectively. This signifies Hisense’s presence in the region, being rated third in the market place for refrigeration. Moreover, to compliment Hisense S.A.’s existing product range, new categories were launched during 2012 that include the production of washing machines and mobile communication devices. With our eco-friendly and energy saving product range, Hisense will continue to offer our ‘reimagine your life’ products to the South African consumer.

Our commitment to quality

We want you to love your Hisense products. Not just when you first buy them, but for years and years to come – that’s why quality is so important to us. Whether it’s our stringent Quality Improvement Process, our commitment to excellent aftersales customer service or the reassurance of our comprehensive warranties, it’s all done to make sure you feel confident that a Hisense product is the right choice for your home.

Innovation through experience

Since 1969, we have been striving to push the boundaries of innovation, working tirelessly on the research and development of electrical products for the home. It’s this dedication to developing and producing cutting edge technology that has seen us become one of the world’s leading electronic manufacturers and the No.1 TV brand in China today.

Education

We encouraged children to face life bravely and change their own fate by learning.

21.Xiaomi

OUR LOGO

The "MI" in our logo stands for “Mobile Internet”. It also has other meanings, including "Mission Impossible", because Xiaomi faced many challenges that had seemed impossible to defy in our early days.Xiaomi Corporation (“Xiaomi ”) was founded in April 2010 and listed on the Main Board of the Hong Kong Stock Exchange on July 9, 2018(1810.HK). Xiaomi is a consumer electronics and smart manufacturing company with smartphones and smart hardware connected by an IoT platform at its core.

Embracing our vision of “Make friends with users and be the coolest company in the users’ hearts”, Xiaomi continuously pursues innovations, high-quality user experience and operational efficiency. The company relentlessly builds amazing products with honest prices to let everyone in the world enjoy a better life through innovative technology.

According to Canalys, the company’s market share in terms of smartphone shipments ranked No. 3 globally in the second quarter of 2022. The company has also established the world’ s leading consumer AIoT (AI+IoT) platform, with 526.9 million smart devices connected to its platform as of June 30, 2022, excluding smartphones, tablets and laptops. Xiaomi products are available in more than 100 countries and regions around the world. In August 2022, the company made the Fortune Global 500 list for the fourth time, ranking 266th, up 72 places compared to 2021.

Xiaomi is a constituent of the Hang Seng Index, Hang Seng China Enterprises Index, Hang Seng TECH Index and Hang Seng China 50 Index.

OUR CULTURE

"Just for fans" – that's our belief. Our hardcore Mi fans lead every step of the way. In fact, many Xiaomi employees were first Mi fans before joining the team. As a team, we share the same relentless pursuit of perfection, constantly refining and enhancing our products to create the best user experience possible. We are also fearless in testing new ideas and pushing our own boundaries. Our dedication and belief in innovation, together with the support of Mi fans, are the driving forces behind our unique Mi products.

OFFICE ENVIRONMENT

We are incredibly flat, open, and innovative. No never-ending meetings. No lengthy processes. We provide a friendly and collaborative environment where creativity is encouraged to flourish.

EVENTS

Xiaomi’ s got talent! Friendly competitions let the Mi team show off their athletic prowess in bask- etball, swimming, badminton and more. Our annual "Mi Idol" has also uncovered plenty of star potential.

CAREERS

Xiaomi is focused on being the most user-centric mobile internet company, and we aim to constantly exceed expectations through innovations in software, hardware and services. Many of our employees were initially fans of Mi products, before they decided to join us. Our team is not only passionate about technology, but also relentlessly pursues perfection to break tradition and push boundaries, all just to ensure that our products remain unique and offer an unparalleled user experience.

Sustainability

Climate Change

Xiaomi recognizes the importance of climate change and environmental protection, and strives to play our part in driving the sustainable development of our society.

With each year, the consequences of climate change are becoming more profound and direr in many parts of the world. Guided by our mission to bring the benefits of technology to everyone for a better life, we believe it is our responsibility to help avert the rapidly changing climate with solutions built upon our products and technological innovations. As a global technology innovator, Xiaomi has unique strengths to leverage our know-hows in technological innovation and operational efficiency to provide solutions. We have infused climate-conscious elements into the design-to-delivery process of our "coolest product", exploring every possible way to integrate low-carbon with Xiaomi's business strategy and brand features, and translating these principles into environmentally-friendly technologies and products. We are proud of the encouraging progress we have made that helps accelerate the global transition to a net zero emission economy.

Climate Resilience

As climate change exacerbates, it has become an issue of urgency for businesses across sectors to build resilience against extreme weather events and climate catastrophes through climate adaptation and mitigation measures. As a global technology company, Xiaomi is also exploring more innovative ways to build climate resilience through its smart devices and technologies.

To this end, Xiaomi has developed the "Natural Disaster Warning" system on its MIUI operating system of smartphone, which receives warning information from the China National Warning Center that includes meteorological, geological, oceanic, forest, and biological disasters. Disaster prevention guidelines are also disseminated to provide holistic information technology support on disaster emergency response measures.

Water Stewardship

Water is an elemental resource that flows through various aspects of sustainable development, and a thriving society and natural environment depend vitally on a well-functioning water system.

Furthering our water stewardship practice to safeguard water security and conserve aquatic ecosystems in the watersheds where we operate, and using technology as a force to enhance access to clean and affordable water resources.

Ensuring that the wastewater generated from our operation is properly treated and safe to discharge in full compliance with local laws and requirements.

Establishing its water management system with reference to the Alliance for Water Stewardship (AWS) standard to conduct water risk assessment, setting water management targets and action plans, and evaluating progress on an annual basis.

Energy

Operational Energy Management

Xiaomi has established an Energy Management System in accordance with ISO 50001 Standard.

Through more extensive application of solar energy facilities, energy grading management, use of sensory lighting, smarter management of air-conditioning systems, optimizing heat exchange station for chillers, and elevator plant room temperature control measures, we have made notable progress in reducing energy consumption and GHG emissions from our operation.

Together with our other energy-saving measures such as the use of variable frequency control and waste heat recovery technology, we delivered approximately 2,630,000 kWh in energy savings and 3,086 GJ in heating savings in 2022, which amounted to 1,839 tons of CO2e emission reduction *.

Improving building energy efficiency

Building energy management is a top priority for Xiaomi, as we strive to ensure sustainable and eco-friendly operations. We thoroughly explore and evaluate opportunities for energy savings in our existing buildings and office campuses. Moreover, we incorporate energy efficiency principles early on in the design process of new buildings, and adopt a green construction approach that considers local conditions and building functions.

Biodiversity

Ensure that our business activities are compliant with applicable local biodiversity laws and regulations in all the markets we operate.

Ensure that our site selection and construction activities avoid and do not cause negative impacts to the habitats listed on the International Union for Conservation of Nature (IUCN) Red List, and the natural and cultural heritage sites listed in the World Heritage List.

Encourage suppliers to conduct biodiversity-related risk assessments associated with their operating sites, and to take necessary measures (such as avoidance, reduction, restoration, and offsetting) in the event that their operating boundaries are in the vicinity of key biodiversity ecosystems and habitats, in order to minimize negative impacts and enhance ecological well-being.

Collaborate with partners to explore possible ways to alleviate biodiversity degradation.

Xiaomi Cares About Biodiversity Protection.

Xiaomi launched a charity program on Xiaomi Fundraising Platform for Charities with a focus on ecological conservation, covering topics on wildlife protection, stray animal rescue, and environmental protection. One of our projects - "Protecting Habitat of Migratory Birds", has an aim to foster the protection of local migratory birds and their habitats through education, scientific research, and policy advocacy. Another highlight is the "Three Rivers Water Conservation and Species Preservation" project - through which we wish to preserve the most pristine and authentic nature of the Three River Sources (or Sanjiangyuan) ecosystems, and to demonstrate the harmony of people co-existing with nature.

Mobile in Africa Group is passionate about empowering South Africans by making innovative, cutting edge technology accessible to all. Our customers are at the heart of everything we do, from choosing the right products to customer after-care. MIA was established in 2015 as a proudly South African distributor of Xiaomi’s world-class, affordable smartphones and smart home products.

MIA’s mission is to bring amazing technology within reach of everyday South Africans, because we know affordable mobile technology makes people’s lives better. We make life easier, simpler, and more fun through our eco-system of automated technology. With this purpose in mind, MIA has built relationships with major retailers and telecoms partners in South Africa, as well as launched our own shoppable website to bring Xiaomi directly to the public we love to serve.

Talent Growth

We recognize that human rights are universal values and an essential part of corporate social

responsibility. We are committed to complying with relevant laws, regulations, and international conventions, and to respecting and protecting human rights. In our business management and operations, we always put people at the center, fully respect and safeguard the rights and interests of our employees, provide a safe and comfortable working environment, create an inclusive and open culture, and support and monitor our suppliers in safeguarding labor rights.

Respect for human rights: We respect and safeguard the human rights of all Xiaomi employees, including those in our supply chain, including but not limited to freedom of speech, equal treatment, and non-discrimination. We adhere to the principles of equality, fairness, and justice, treating all employees equally, regardless of gender, race, religion, nationality, age, sexual orientation, disability, and other factors. We prohibit any form of discrimination, oppression, and abuse.

Support for employee growth: We comply with Chinese and international laws and regulations on labor rights protection, and have formulated and implemented the "Xiaomi Group Employee Handbook". We design training systems that align with various talent development needs to cultivate, motivate, and retain professional tale

nts that meet our corporate development needs. We provide equal employment opportunities and good career development paths for our employees, and develop competitive recruitment, employment, welfare, and incentive policies worldwide, ensuring that our employees receive fair treatment, work in a safe, healthy, and excellent environment, and have a sound compensation system.

Engineer Culture

At Xiaomi, we are relentless to forge an "engineer culture" that focuses on innovation and optimal efficiency. We embrace differences, encourage free-minded creativity and expression, and harness the power of our diverse people to drive innovation at scale.

Business Ethics

Xiaomi is committed to conducting business ethically and in full compliance with applicable laws and regulations.

Training

We organized a series of ethical training that covered the topics of anti-bribery, corruption news, legal requirements, ethical standards, case studies, whistleblowing, and conflict of interest. Every year, the entire Board of Directors was updated on the progress of integrity management and the outcome of anti-corruption training. We rolled out extensive online and offline training sessions to all levels of our employees (management, frontline staff, fresh graduates, interns, and part-time employees), and achieved 100% employee coverage. We also provided training on the aforementioned topics to our business partners (e.g. suppliers, contractors).

Anti-bribery and corruption

At Xiaomi, we uphold the principles of openness, fairness, transparency, and integrity, with a "zero tolerance" policy against bribery and corruption in order to achieve "full coverage and no restricted area" in anti-corruption management.

Continually optimize our governance structure and management policy, and upscale anti-corruption training to nurture a positive and corruption-free work environment;

Continually update our anti-bribery and corruption policies for employees, suppliers, and other business partners;

Released a new version of the Business Integrity Agreement in 2022, which has been appended

to our service agreements with suppliers and business partners as part of their contractual obligations.

Anti-monopoly

Xiaomi puts a high emphasis on anti-monopoly and anti-unfair competition compliance.

Xiaomi has included anti-monopoly and anti-competitive behaviors in our Code of Conduct for Employees, and published the Anti-monopoly Compliance Handbook.

This handbook specifies the definitions of monopoly agreements, abuse of dominant market position, compliance with concentration of undertakings, and anti-monopoly investigation procedures to guide a responsible business development.

Intellectual Property

Xiaomi established robust intellectual property (IP) management system to protect the fruits of intellectual endeavor and respect those from the partners in our ecosystem.

Established an IP management system that entails a multi-disciplinary IP management framework that covers patent planning, trademark and brand identity, copyright, open-source, data protection and privacy. Each business unit designates an IP specialist to take responsibility for implementation and action.

Advocate and explore diverse and sustainable collaboration in IP with our industry peers and business partners to create shared values.

Officially released our inaugural Xiaomi Intellectual Property White Paper in 2022, which sums up our years of practices in IP protection.

22.OPPO

OPPO is a leading innovator in smart device.

Technology as an art form

The world's leading smart device manufacturers and innovators.

Step by step, OPPO has built a global innovation system that bolsters the exploration of cutting-edge technologies. Since it was established in 2004, OPPO's businesses has expanded to more than 60 countries and regions worldwide. More than 40,000 OPPO employees dedicate themselves to technological innovations that create a better life for all. ColorOS now includes a full spectrum of system applications to create a free and frictionless user experience for 500 million users around the world.

Awards and Honours

OPPO has deepened its competencies in various fields, earning the recognition of users and professional institutions all over the world OPPO has been cultivating its capabilities for nearly 20 years and gone through four transformations spanning four different eras. OPPO has deepened its competencies in various fields, earning the recognition of users and professional institutions all over the world.

Brand Values

The core of OPPO's corporate culture has always been BenFen, and it is committed to creating great products that would delight the users. In the face of pressure and temptations from the external environment, we firmly believe that maintaining a calm posture and focusing on the fundamentals is the only way to make correct judgements and choices that will pave the way to health and longevity for the enterprise.

Brand Mission

“Technology for Mankind, Kindness for the World” is the driving force behind OPPO's

innovation and technology development. In other words, OPPO is committed to helping every individual with innovative spirit to grow, as well as helping the society as a whole to develop, while practicing the BenFen culture, helping humankind to build a mutually beneficial and inclusive society.

Sustainability Strategy

Aligned with our mission of "Technology for Mankind, Kindness for the World", we are committed to integrating social responsibility into business practices, to contribute to the sustainable development.

Operation and Compliance

Build a compliant and harmonious operational environment to facilitate the healthy and sustainable development of the business.

Environment Protection

Build a green and low-carbon business operation model to create an environmentally-friendly value chain.

Caring for Employees

Establish and implement labor standards to build a healthy and harmonious work environment.

Virtuous Innovation

Provide more users with digital convenience through technological innovation.

Ecosystem Engagement

Carry out interactions among core stakeholders to promote the eco-integration and common development.

OPPO Supplier CSR Code of Conduct

OPPO is committed to building a healthy, safe, responsible and sustainable supply chain worldwide, strictly complying with laws and regulations and upholding the highest ethical standards to provide customers with decent products, services and experience. In accordance with the valid International Labor Organization (ILO) Conventions, the Universal Declaration of Human Rights and the United Nation Convention on the Rights of the Child, and with reference to the Social Accountability 8000 Standard (SA8000) and ISO 26000 - Guidance on Social Responsibility, OPPO formulates its Corporate Social Responsibility (CSR) work principles here, requiring business partners, collaborators, and service providers at all levels in supply chain (hereinafter collectively referred to as "suppliers") to operate in accordance with the requirements set out in this OPPO Supplier CSR Code of Conduct (hereinafter referred to as "Code") and in compliance with applicable laws and regulations of the country and/or region where it locates, respect religious ethics and local folklore, and keep in line with social ethics when processing all its business activities.

This Code applies to suppliers who provide products and/or services to OPPO. OPPO will assess its suppliers' compliance with this Code, and any violations of the Code may jeopardize a supplier's business relationship with OPPO, based on which, OPPO reserves the right to terminate cooperation with suppliers.

The requirements listed below in this Code are the basic principles to guide suppliers towards business ethics and compliance. OPPO encourages suppliers to apply higher standards than those of laws, regulations and general ethics, when engaging in production and business activities, so as to benefit employees and the community, and fulfill corporate social responsibilities.

Laws and Regulations

Suppliers shall strictly comply with the requirements of national/regional laws and regulations in production and business activities. In case of any conflicts between legal/regulatory requirements and industry regulations or the requirements of this Code, the stricter standards shall apply.

Ethics and Human Rights

Suppliers shall conform to social ethics during production and business activities, protect the basic rights and needs of the survival and development of employees, and ensure that their dignity, rights and interests are protected from any infringement to the greatest extent possible.

Employment Relationship

Suppliers shall establish a fair and just employment relationship with workers, and guarantee the basic rights and benefits of workers are protected in accordance with applicable laws and regulations.

Child Labor and Juvenile Workers

Any form of child labor is strictly prohibited. If juvenile workers are employed, suppliers shall undertake their responsibilities and obligations for the protection of juvenile workers in accordance with applicable laws and regulations.

Working Hours

Suppliers shall follow the requirements of applicable laws and regulations on working hours. The regular working hours per week shall not exceed 48 hours and the weekly total working hours shall not exceed 60 hours, meanwhile, employees shall be guaranteed to have at least one day off every seven days except in emergencies or unusual situations.

Remunerations and Benefits

Suppliers shall comply with the local minimum wage standards, accurately calculate and compensate overtime work, pay employees in monetary way fully and timely, provide and guarantee the basic rights and benefits of employees in accordance with the applicable local laws and regulations. Wages deduction or withholding to cut employees' deserved work income in any form should not be permitted.

Anti-Discrimination

Any form of discrimination is strictly prohibited. Suppliers shall not unfairly treat or judge employees based on their ethnicity, race, region, accent, gender, age, political affiliation, marital status, religious belief, disability, sexual orientation or any other form of personal characteristics.

Humane Treatment

Any form of forced labor, slavery, labor exploitation or use of bonded, indentured or prison labor is strictly prohibited. Suppliers shall not directly or indirectly use involuntary labor or get labor support from human trafficking. No corporal punishment, mental coercion, verbal or physical bully or harassment is allowed.

Freedom of Association and Collective Bargaining

Suppliers shall respect the legitimate rights and interests of employees to join employee organizations and express appeals or different opinions under the terms set forth in applicable regulations, set up effective channels for collecting and processing employee complaints, opinions and suggestions, listen to their voices, follow up on appeals in a timely manner, and establish a positive, harmonious employee communication mechanism that enables win-win situations.

Health and Safety

Suppliers shall build healthy, safe and reliable workplaces and facilities for employees, and provide necessary training to employees for improving their awareness of health and safety. If catering and accommodation are arranged, the health, safety, and comfort of those facilities should be guaranteed as well.

Environment

Suppliers shall ensure that the impact of their production and business activities complies with national and/or regional environmental protection regulations, and discharge or disposal of waste generated from the production and business activities in compliance with the related laws and regulations. Suppliers shall reasonably use the energy and water resources, advocate the use of clean energy and environment-friendly materials in production and operations, and actively cooperate with the local community in sustainability to support the construction of a transparent and positive cultural environment.

Conflict Minerals

Suppliers shall not directly or indirectly purchase minerals (i.e., 3TG - Tungsten, Tantalum, Tin and Gold) that come from mines controlled by anti-human armed forces in the Democratic Republic of the Congo and its adjoint countries or regions, which may convert into finance the serious violations of human rights and the destruction of the cultural and ecological environment there. Suppliers shall conduct source due diligence in supply chain if any of the above-mentioned metals used in production or operation, and maintain the relevant records for traceability verification to demonstrate a compliance to responsible mineral management.

Trade Security

Suppliers shall strictly follow international trade regulations and regulatory requirements, fulfill obligations in global anti-terrorism cooperation, and shall not support or disguise any form of terrorist activities, including but not limited to hiring, harboring, shielding terrorists or suspects of committing atrocities against humanity, carrying or concealing lethal weapons or viruses in the product packaging or transportation; and publishing or spreading panic-arousing messages in network systems or intelligent groups at all levels.

Information Security

Suppliers shall be committed to constructing a rigorous and reliable information security system, reasonably classifying and protecting all types of business information and employee personal information, and shall not disclose sensitive and classified information such as business secrets, user confidential data, employee privacy in any form without appropriate authorization. Suppliers shall respect intellectual property rights and shall not access professional technical information through unofficial or other illegal means, or misappropriate or disseminate such information.

Business Integrity

Suppliers shall uphold the highest standards of business ethics, abide by the law and operate with integrity. Engagement in corruption, bribery, extortion and misappropriation is strictly prohibited, and it's not allowed to gain benefits or get improper advantage in business through unfair competition.

Documentation and Records

Suppliers shall document accurate records of activities in production and operation in accordance with the law, and maintain the consistency and traceability of the documentation. It is not allowed to forge or alter documents or provide false information. All records shall be kept for at least six years for possible verification or review.

Supervision and Audit

Suppliers shall strictly comply with this Code and conduct periodic evaluations of their facilities and operations to ensure compliance with this Code and the law, and drive continuous improvement. OPPO reserves the rights to appoint dedicated employees or authorized third-party representatives to perform supervisory audit in the plants of suppliers.

Company Culture

Vision

We strive to be a sustainable company that contributes to a better world.

Company Culture

We strive to be a sustainable company that contributes to a better world.

Mission

We exist to elevate life through technological artistry.

Values

Benfen | User-led | Aim for perfection | Goal-oriented.

23.Hikvision

Hikvision is committed to serving various industries through its cutting-edge technologies of machine perception, artificial intelligence, and big data, leading the future of AIoT:

Through comprehensive machine perception technologies, we aim to help people better connect with the world around them;

With a wealth of intelligent products, we strive to identify diverse demands by delivering intelligence at your fingertips;

Through innovative AIoT applications, we are dedicated to empowering every individual to enjoy a better future by building an intelligent world that is more convenient, efficient and secure.

Hikvision provides a broad range of physical security products, covering video security, access control, and alarm systems. We also provide integrated security solutions powered by AI technology to support end-users with new applications and possibilities for safety management and business intelligence. Over the past several years, we have deepened our knowledge and experience in meeting customer needs in various vertical markets with professional and intelligent solutions, including smart city, transportation, retail, logistics, energy, and education. In addition, Hikvision is extending its business to smart home, robotics, automotive electronics, intelligent storage, fire security, infrared sensing, X-ray detection, and medical imaging to explore new channels for sustaining long-term development.

Hikvision has established one of the most extensive marketing networks in the industry, comprising 66 subsidiaries and branch offices globally, to ensure quick responses to the needs of customers, users and partners. Hikvision products serve a diverse set of vertical markets covering more than 150 countries.

Hikvision went public in May, 2010, and is listed on SMEs Board at Shenzhen Stock Exchange.

Our Vision

Empower vision for the security and sustainable growth of the world.

Our Mission

To explore innovative ways to better perceive and understand the world, to empower vision for decision-makers and practitioners, and work together to enhance safety and advance sustainable development around the world.

Core Businesses

Intelligent security products

Integrated security solutions

Intelligent vertical solutions

Corporate Sustainability

Overview

Hikvision is committed to integrating corporate social responsibility and sustainability development philosophy into our business, while also being driven by technological innovation. We are dedicated to fulfill our global responsibilities to create lasting value for customers, improve social well-being, and work together with partners to ensure harmonious co-existence and sustainable development globally.

Our commitment

Technology for Good

Based on innovative and intelligent products and technologies, we will serve and give back to society with kindness in mind. We are dedicated to empowering every individual to enjoy a better future by building an intelligent world that is more convenient, efficient and secure.

Integrity and Compliance

We will conduct our business in a manner consistent with high standards of business ethics and pursue a business philosophy of honesty, integrity and legal compliance as a cornerstone of our sound, long-term global growth.

Green and Low-Carbon Development

Adhering to the idea of green, sustainable development, we will continue to enhance environmental management, optimize our use of resources, reduce pollutant emissions and explore low-carbon technologies in our business operation and technological innovation.

Harmonious Co-existence

We are committed to creating value for customers, improving social well-being, providing a solid platform for employees and working together with partners to ensure harmonious co-existence and sustainable development.

Our Solid Foundation

Always focused on quality

Reliability and quality management have been fundamental pillars in the success of Hikvision. We are very proud of our full implementation of product lifecycle quality management that meets leading global standards and with many authoritative certifications. The full lifecycle quality

management process covers material quality, innovation for design quality, manufacturing quality, and end-to-end quality services.

We have established a strict quality management regime based on compliance with the following key systems and protocols.

In-house manufacturing

Hikvision runs extensive in-house manufacturing operations with more than 1 million square meters of combined manufacturing facilities in Hangzhou, Tonglu, Chongqing and Wuhan, China, and international manufacturing locations in India, Brazil and the UK. Fully-automated SMT production lines and machine vision products are incorporated to ensure premium quality products, flexible production, and punctual delivery to meet the unique development needs of our various business units.

Our Innovations

With significant R&D investment, Hikvision is advancing several core technologies, covering multi-dimensional perception, artificial intelligence, and big data, as well as imaging technologies, cloud computing, and video codec.

Imaging and video related technologies remain very important for Hikvision, and we have been leading the industry in numerous areas, including high definition, and low-light imaging, as well as image defogging, smart rapid focus, and image stabilization among others.

Besides visible light imaging, Hikvision is extending its machine perception technologies to the full electromagnetic spectrum. Varying in wavelength and frequency, electromagnetic waves can extend from visible light to both ends of the known spectrum. Hikvision has developed innovative products and applications powered by thermal imaging, multispectral imagery, radar sensing, and so on. We are also exploring multiple machine perception technologies that pick up X-rays, infrared rays, millimeter waves, sound waves, and temperature variations, etc.

In addition, our extensive supply chain ecosystem covers more than 1,000 long-term suppliers globally, enabling us to maintain robust supply chain stocks to ensure reliable product delivery.

Corporate Sustainability

Hikvision is committed to integrating corporate social responsibility and sustainability development philosophy into our business, while also being driven by technological innovation. We are dedicated to fulfill our global responsibilities to create lasting value for customers, improve social well-being, and work together with partners to ensure harmonious co-existence and sustainable development globally.

By integrating corporate social responsibility and sustainable development philosophy into our business, while also being driven by technological innovation, Hikvision is committed to being known as a respected global technology company. In 2022, we continued our efforts for the improvement of public safety, traffic management, business efficiency, environmental protection, etc.

Upholding our aspiration to serve society and create a brighter world for all, we have continuously taken actions towards fostering sustainable development. We not only encourage and organize employees to engage in social endeavors to make meaningful contributions to public welfare, but also harness Hikvision's expertise, fueled by innovative technologies and professional know-how, to construct a smart ecosystem and foster a harmonious society.

Tech for an inclusive society

Hikvision is dedicated to leveraging AIoT and other advanced technologies to offer greater

support to communities. We have developed a diverse range of products and solutions tailored to the specific needs of vulnerable groups, creating a smarter and more convenient environment.
Tech for thriving industries
Driving digital transformation with leading AIoT technologies, Hikvision empowers enterprises and other organizations in enhancing production security and improving operational efficiency, while supporting improved working conditions for employees.
Tech for livable cities
At Hikvision, we are dedicated to expanding intelligent applications to improve city operations and management. We help achieve high-quality public services, smooth traffic flow, and livable environments, thus bringing more possibilities for healthy and joyful lives.
Tech for sustainable environment
Hikvision utilizes the power of AIoT to protect the environment, enable low-carbon development, and foster the harmonious coexistence of humans and nature. Together with our partners, we will continue to explore innovative ways to a sustainable future.
STAR Program for Social Good
Main focus areas
Biodiversity Monitoring and Conservation
Let the world see more hope of life
Biodiversity is essential for all life on earth and critical for sustainable economic and social development. Hikvision would like to provide technologies, products, and solutions for biodiversity projects to support them better observe and protect wildlife, focusing on areas such as:
Wildlife monitoring
Digitalization of biodiversity conservation
And more⋯
Environmental Monitoring and Protection
Let the world see more sustainable environment
Environmental harm and climate change pose significant risks to the earth, and are a concern for people everywhere. Hikvision has explored multi-dimensional perception technologies and accumulated advanced application practices in intelligent environmental monitoring and protection, in a number of fields such as:
Air quality monitoring
Water quality monitoring
And more⋯
Cultural Heritage Preservation
Let the world see more cultural heritage
Cultural heritage is an invaluable resource from the past, it connects societies today, and should be passed on to future generations. Hikvision is committed to making contributions to protection of our heritages with innovative technologies, which includes:
Historical heritage security protection
Virtual access to museums
And more⋯
24.CLOUDWALK
The National Team of AI: A More Efficient Provider of Man-Machi

Incubated from the Chinese Academy of Sciences, Cloudwalk is a leading enterprise of AI in China to undertake the construction of three major national platforms and participate in the formulation of national and industry standards. Focusing on every scene of people ’ s life, Cloudwalk, with the world ’ s leading man-machine coordination platform, provides users with cross-scene, cross-industry and personalized AI services, in the hope of building a more harmonious and warmer intelligent future.

After years of profound cultivation in the industry, Cloudwalk has successively explored four major business areas, i.e. AI finance, AI governance, AI transportation and AI business. Cloudwalk has become the largest AI supplier in the financial industry, with a market share of 82.8%. Formulating the integrated solutions based on industry-leading AI, cognitive computing and big data technology, Cloudwalk provides intelligent, convenient and user-friendly AI experience for 300 million users around the world every day.

As China ’ s development of new infrastructure deepens, Cloudwalk will always practice its original intention, devote itself to innovatively exploring man-machine coordination, defining AI life and strengthening the potential of mankind, and build a harmonious and warm AI-based future for the world.

What does Cloudwalk adhere to

We insist that AI, which is always people-oriented, should focus on people ’ s challenges and pressure and respect people ’ s emotions and demands. We constantly improve our products and services through the in-depth innovation of AI engineering, so that AI can develop towards a positive direction to truly understand, help and promote people.

What does Cloudwalk advocate

Advocating the spirit of persistent exploration, pragmatism and daring to do things, we persist in doing one thing till we succeed. We endure the loneliness, seek extraordinariness out of ordinariness, do warm things with a dedicated heart, and persevere to the end till we achieve the intelligent future featuring man-machine coordination.

How does Cloudwalk make the world smarter

After years of profound cultivation in the world ’ s cutting-edge industries, Cloudwalk has gained a deep understanding of the demands of users and integrated more forward-looking AI technology with big data technology to provide high-quality and rich integrated solutions in various fields such as finance, governance, transportation, commerce, health care, education and new infrastructure. As the largest AI supplier in the Chinese financial and civil aviation industries, Cloudwalk provides more intelligent, convenient and humanized AI experience for 300 million users around the world every day.

What does Cloudwalk adhere to

Sticking to practice the original intention of founding Cloudwalk, we focus on the innovative exploration of AI engineering and the development of a more powerful super brain. Relying on the world ’ s leading man-machine coordination platform, we strive to build a brand-new world featuring man-machine harmonious symbiosis and integrated co-creation, in the hope of making life warmer and more intelligent for every industry, every family and every person, and filling the future world with ubiquitous intelligence.

WORLD-LEADING AUTONOMOUS AND CONTROLLABLE

Man-machine coordination to construct a future city with ambient intelligence

Original and intelligent creation by the world’s leading technical team
The world’s top three-tier R & D architecture
One forefront
Grasp the development trend of cutting-edge AI science and technology at the forefront of the world
Three fulcrums
We’ve mastered the research ability with regard to basic core technologies
Shanghai Center for Brain Science and Brain-Inspired Technology
Joint Laboratory of Cloudwalk and the Chinese Academy of Sciences
Joint Laboratory of Cloudwalk and Shanghai Jiaotong University
6
SIX R & D centers
With more than 1000 R & D personnel, we provide the capability of researching and developing core technological products
We cultivate the reserve force of AI by establishing a university with AI as the orientation
We have set up R & D centers in Guangzhou, Chongqing, Shanghai, Suzhou, Chengdu and Wuhu
Breaking world records more than once
Cloudwalk has made a breakthrough in terms of 3D human body reconstruction technology, reducing the accuracy error by 30% and increasing the computational speed of the algorithm by ten times to 5 milliseconds.
25.China Telecom Global

China Telecom Corporation Limited (“China Telecom”), one of the world’s largest providers of integrated telecommunication services, has been unwaveringly strived to enhance its capabilities in maintaining its global footprints while addressing changing demands. In 2000, China Telecom established its first overseas office. In order to further enhance its global service quality and accelerating overseas business expansion, China Telecom established China Telecom Global Limited (“CTG”) in 2012, which is headquartered in Hong Kong, China.

Leveraging its vast network resources of 47 submarine cables with 74T in intercontinental capacity and 218 Points-of-Presence (PoPs) around the world, China Telecom offers a high-performing global network for international carriers, multinational enterprises and overseas Chinese customers. China Telecom delivers a wide portfolio of high quality, integrated communications solutions, including internet direct access, internet transit, data services, broadband, unified communications, internet data centres, cloud computing, ICT services, fixed-line and mobile services, multi-domestic MVNO and global IoT connectivity service, professional services, industry solutions, telecom operation consultancy and service outsourcing.

With an agile and forward-looking spirit, innovative products and business models, and industry-leading technologies, China Telecom has become a world-class integrated telecommunication services provider with presence in 41 countries and regions. China Telecom is dedicated to creating value for its customers in their business transformation, enabling them to achieve business growth, enhance global footprints and maintain competitive edges by digitalization.

China Telecom Corporation Limited ("China Telecom" or the "Company", a joint stock limited company incorporated in the People's Republic of China with limited liability, together with its subsidiaries, collectively the "Group") is a leading large-scale integrated intelligent information services operator in the world whose principal business is the provision of fundamental telecommunications businesses including wireline and mobile communications services, value-added telecommunications businesses such as Internet access services, information services and other related businesses. As at the end of 2022, the Company had mobile subscribers of about 391 million and wireline broadband subscribers of about 181 million. The Company's A Shares and H Shares are listed on the Shanghai Stock Exchange and the Main Board of The Stock Exchange of Hong Kong Limited, respectively.

The Company actively embraces the opportunities of digital transformation by cultivating customer needs and use cases, fully implementing the “Cloudification and Digital Transformation” strategy, building new information infrastructure based on cloud-network integration, operation support system and sci-tech innovation core strength with 5G and cloud as the core, deepening system and mechanism reform, and creating differentiated advantages through innovation and convergence. China Telecom is committed to providing flexible and diversified, converged and convenient, secure and reliable integrated intelligent information services with quality experience for individual (To C), household (To H), and government/enterprise (To B/G) customers.

CORPORATE CULTURE

Strategic Goal

Be a world-class integrated
information services provider

Service Philosophy

Customer First
Service Foremost

Core Value

Comprehensive innovation
pursuing truth and pragmatism,
Respecting people and creating value all together

Operation Philosophy

Pursue mutual growth of corporate value and customer value

Corporate Mission

Let the customers fully enjoy a new information life

Corporate Slogan

Connecting the World

Code of Corporate Practice

Keep promise and provide excellent service for customers Cooperate honestly and seek win-win result in joint innovatiorOperate prudently and enhance corporate value continuously Manage precisely and allocate resources scientifically Care the staff and tap their potential to the full Reward the society and be a responsible corporate citizen

COMPANY STRATEGY

Adhering to the mission and vision of “as the main force for building Cyberpower, Digital China and maintaining network and information security” and “becoming a leading integrated

intelligent information service operator", China Telecom comprehensively and thoroughly implements the "Cloudification and Digital Transformation" strategy. With a customer-oriented mindset, China Telecom strengthens the core capabilities of sci-tech innovation, speeds up the construction of new information infrastructure based on cloud-network integration, greenness and security. It consolidates the foundation of green development and network and information security, builds a digitalised platform hub, creates a win-win cooperation ecology, deepens the system and mechanism reform, accelerates development with higher quality, promotes sci-tech innovation to a higher level, deepens the reform more comprehensively, steps up efforts on open cooperation, places more emphasis on risk prevention and works together in more practical ways, makes every effort to build a service-oriented, technology-oriented and secured enterprise and earnestly enhances the enterprise's core competitiveness, to accelerate to become a world-class enterprise.

BUILDING HARMONY THROUGH OPENNESS AND INCLUSIVENESS

Insisting on the "people-oriented" principle, China Telecom cared for employees and strove to achieve the common growth of employees and the Company. It supported rural revitalisation, narrowed the digital divide and enthusiastically participated in social welfare activities to share the accomplishments in digital development with people. At the same time, the Company adhered to open cooperation, proactively built an open ecology with partners and facilitated interconnection of global networks, proactively fulfilled overseas social responsibilities, supported the development of local communities, and supported the creation of a bright future for the mankind as a whole.

CARING FOR EMPLOYEES

China Telecom protected the rights and interests of employees in accordance with the law, focused on establishing harmonious labour relations, cared for employees' well-being, supported the labour union to perform its functions, encouraged employees to participate in management, and proactively helped employees to enhance their capabilities, thus striving to achieve the common growth of employees and the Company.

Protecting employees' rights and interests

The Company protected the labour rights, democracy rights and spiritual and cultural rights of employees in accordance with the law, enhanced labour management, and conducted workforce employment in accordance with laws and regulations. The Company adhered to the principles of equality, voluntariness and consensus, and implemented the Notice on Issuing of the Labour Contract of China Telecommunications Corporation (Template) and other documents
in accordance with relevant laws and regulations regarding labour and the protection of the employees' rights and interests including the Civil Code of the People's Republic of China, the Law of the People's Republic of China on Labour Contracts and the Trade Union Law of the People's Republic of China. The Company entered into written labour contracts with employees, specified in detail the circumstances under which employees may terminate labour contracts and implemented the contracts in accordance with laws and regulations, to protect their basic rights and perform the obligations of both parties.

The Company valued the labour of employees, attached great importance to employees' health, full paid remuneration and social security insurance in a timely manner, implemented the paid leave system, specified working hours, rest and vacation, to protect the legal rights of employees.

The Company continually enhanced the business operation models and job role classification for

labour dispatch, clearly determined the employment form of each role, standardised the designated agreements signed with labour dispatch units. The Company checked and supervised these dispatch units and the signing of employment contracts by dispatch workers, and paid remuneration and social security insurance in a timely manner.

The Company recruited talents from the whole society with full compliance with the Employment Promotion Law of the People’s Republic of China, making job opportunity information available on the Company’s website, official account, third-party recruitment websites and such other channels with due respect to fairness, openness and impartiality, to solicit various outstanding talents through a number of channels and diversified recruiting approaches. The Company offered equal opportunities to all applicants in its recruitments without discrimination against ethnicity, race, gender, age, region, marital status or physical condition, and offered suitable jobs to the disabled according to their characteristics. The Company adhered to equal pay for equal work, provided employees with promotion in their positions and smooth career development paths. The Company handled and used its employees’ personal information in strict compliance with the applicable laws and regulations, showed high respect to their privacy and ensured security of such information.

The Company attached great importance to occupational health management of its employees, established and optimised the Interim Measures for Labour Protection of Female Employees of China Telecommunications Corporation and other internal occupational health and labour protection policies in accordance with the Occupational Disease Protection Law of the People’s Republic of China and such other laws and regulations related to occupational safety and health. The Company launched the Employee Assistance Program (EAP) to protect the occupational safety and physical and mental health of its employees.

The Company strictly implemented the relevant requirements of the Regulations on the Prohibition of Child Labour, prohibited child labour and forced labour in accordance with laws, and specified the age requirements of candidates in accordance with the recruitment management measures to avoid child labour. In 2022, no instances of child labour or forced labour was found.

Caring for employees’ well-being

The Company continued to strengthen communications with its employees, understand their needs and increase its care for them to enhance their sense of gain, happiness and safety.

Supporting employees’ development

The Company continued to promote the development of the cadre and talent team, strengthened employees’ training, promoted the spirit of model workers, encouraged employees to participate in management, further enhanced the skills and values of the workforce.

The Company solidly pushed forward the establishment of a high-quality cadre team and set clear talent allocation orientated to hard work and entrepreneurship. The Company selected candidates and established teams with a focus on corporate high-quality development, while continuously optimising the age and professional structure of the management team, making greater efforts to select and train young and outstanding cadre as well as sci-tech cadre, aiming to establish a reserve of sci-tech talents. The Company strove to foster correct ideals and beliefs among its managers, encouraging them to take necessary responsibilities, aiming to lay a solid foundation for the successful implementation of its “Cloudification and Digital Transformation”

strategy, as well as the acceleration of the building of a world-class enterprise with high-quality development.

Adhering to the principle that science and technology constitute the primary productive force, talent is the primary resource, and innovation is the primary driver, the Company constantly advanced its "Outstanding Enterprise Powered by Talent" project by holding sci-tech talent work conferences, releasing the "14th Five-year" sci-tech talent development outline, aiming to raise four sci-tech talent teams, i.e. a team of strategic scientists, a team of technological leaders and innovators, a team of outstanding engineers, and a team of young sci-tech talents. The Company further advanced the reform of its talent development system and mechanism, while promoting the establishment of talent cloud, talent work station, special talent pools and such other mechanisms, so as to ignite the team's vitality.

The Company continued to strengthen employees' training. The Company kept abreast of the development trend of the digital economy, comprehensively promoted its "Cloudification and Digital Transformation" strategy, held 27 sessions of "Cloudification and Digital Transformation Seminar" series and four sessions of "the Way of Transformation" lecture series throughout the year, with more than 2 million person-times. The Company further strengthened the empowerment of its cloud expert team, and promoted its cloud empowerment system through the integration of "learning zone of China Telecom Cloud empowerment + key topic learning + China Telecom Cloud certification + train the trainers + labour contest + training camp", covering nearly 70,000 cloud professionals. The Company also organised large-scale talent trainings at different layers and levels, aiming to extensively foster three teams of engineers specialised in Industrial Digitalisation, R&D, and cloud-network through "Practice + Certification", which are open to the front-line workforce, with skill certification exams covering 40 majors in various professional lines and 370,000 person-times, promoting the transformation of skilled talents into outstanding engineers.

The Company encouraged employees to participate in management. During the year, the parent company revised and optimised the terms of reference of its employee representative congress by formulating a plan on the re-election of the employee representatives as a step to organise and prepare for election of the second employee representative congress. The Company has been unwaveringly soliciting suggestions from its employee representatives, and has received over 100 suggestions, covering cloud-network integration, sci-tech innovation, green development, network and information security, customer service, enterprise operation and management, workforce development, and care for its employees, etc. Since the first employee representative congress held in 2017, the Company's democratic management, democratic participation, and democratic supervision mechanisms have been further enhanced, and employees' rights to know, participate, express, and supervise have been effectively guaranteed, indicating that their involvement in the Company's democratic management has reached a new level.

SHARING DEVELOPMENT ACHIEVEMENTS

China Telecom took prudent steps to consolidate and expand its achievements in poverty alleviation and effectively link it with rural revitalisation, and persisted in giving full play to the role of the digital information infrastructure in empowering economic and social development, with the aim of narrowing the digital divide and enhancing digital inclusion, so that all people may enjoy the achievements of digital development. Serving rural revitalisation 2022 is the year

for the deepening of the consolidation and expansion of achievements in poverty alleviation and its effective linkage with rural revitalisation. The Company maintained its supporting efforts and gave full play to its corporate strengths to advance its key tasks such as targeted assistance and industrial assistance in a solid and orderly manner.

Throughout the year, the senior management of the Company carried out all-round supervision, inspection and study of the four targeted poverty alleviation counties and two targeted support counties (hereinafter referred to as "4+2" poverty alleviation counties), facilitated the introduction of free assistance funds of RMB18.99 million, and the training for 53,441 cadre-times and talents in the "4+2" poverty alleviation counties. The Company proactively carried out poverty alleviation through consumption, helping the characteristic industries of the "4+2" poverty alleviation counties and over 1,400 poverty alleviation sites of enterprises at all levels to develop and grow, effectively consolidating and expanding the achievements of poverty alleviation, supporting comprehensive rural revitalisation.

26.China Unicom Global

China United Network Communications Group Corporation Limited ("China Unicom") was established on January 6, 2009 based on the merger between the original China Netcom and China Unicom. In addition to over 130 overseas business access points (PoPs), it has subsidiaries in 31 provinces (autonomous regions and municipalities) across China and many other countries and regions around the world. It owns a modern communication network with nationwide coverage, global reach, and worldwide customer service system. Currently, it has over 850 million subscribers to its "Big Connectivity" portfolio.

In 2022, the company ranked 267th in the Fortune Global 500. As a provider of basic communication services to support the Party, government bodies, military organizations, various sectors and the general public, China Unicom plays a fundamental, pillar, strategic and leading role in the national economy with technology-intensive offerings, integration across its entire network, economies of scale, and commitment to serving the economy, the society and people's livelihood.

In recent years, China Unicom has adhered to political building to lead its efforts in thorough implementation of the decisions and deployments of the CPC Central Committee. Starting from 2021, China Unicom has fully undertake the new mission entrusted by the new era, with a clearly defined development orientation during the "14th Five-Year Plan" period to grow the company into "a national team in the operation and service of digital information infrastructure, a key force in the development of cyber strength, Digital China and smart society, and a frontline troop in the integration and innovation of digital technologies". The company's strategy has been upgraded to "strengthen & solidify, preserve & innovate, and integrate & open", placing more emphasis on strengthening the network and solidifying service-related efforts as the underlying foundation; preserving network-based advantages complemented by innovation toward digitalized intelligence; integrating resources and factors while opening markets to prosper hand in hand with partner players. Under such new positioning and strategy, China Unicom will make a full endeavor on the track toward the digital economy, with "Big Connectivity, Big Computing, Big Data, Big Application, Big Security" as its main responsibilities and businesses, to transform and upgrade comprehensively in terms of development power, path and approaches to create new

room for development and integrate into the new development pattern.
With a unswerving determination to build up, optimize and enhance state-owned capital and to grow into a world-class enterprise with global competitiveness, China Unicom resolutely fulfills national missions to establish unblocked information artery and new digital base for economic and social development, through comprehensive approaches to building industry-leading intelligent and comprehensive digital information infrastructure in greater breadth and depth; it resolutely implements the strategy of strengthening national cyber strength and supports the development of Digital China and smart society, by empowering thousands of industries to ” migrate to the cloud and apply digital intelligence ” with its technically-advanced, highly integrated digital services featuring "full coverage, online connectivity, cloud accessibility, greenness and one-stop-shop offering" for digitalized, networked and intelligence-based development; it is also determined to realize innovation-driven development with focuses on core technologies and key applications while making converged and innovative efforts to transform China Unicom into a technology- and innovation-based enterprise with high-level of self-reliance and self-improvement of digital technologies so as to become an important part of the nation’s strategic technological strength.
Committed to its social responsibility as a central SOE, China Unicom takes every possible action to be the "new engine" that empowers social and economic transformation. It gives full play to the technology advantages of big data and communication network to promote the digital economy and the upgrading of information consumption, supports the shifting of old drivers of growth to new ones in economic development, and practically improves the satisfaction and sense of gain among the majority of users regarding information and communication services, further benefiting the whole society with new achievements in ICT development.
27.China Mobile International Limited (CMI)
Overview
China Mobile International Limited (CMI) is a wholly-owned subsidiary of China Mobile. In order to provide better services to meet the growing demand in the international telecommunications market, China Mobile established CMI in December 2010, headquartered in Hong Kong, China. CMI has expanded its footprint in 36 countries and regions.
Leveraging the strong support by China Mobile, CMI is a trusted partner that provides comprehensive international telecom services and solutions to international enterprises, carriers and mobile users.
CMI was established in December 2010, and is responsible for China Mobile's international business operations. Headquartered in Hong Kong, China, CMI has expanded its footprint in 38 countries and regions.

Corporate culture
TRUST Build Mutual Trust
RESPONSIBILITY Undertake Our Responsibility
CARE Care For You
China Mobile firmly believes that “lucid waters and lush mountains are invaluable assets”. We therefore prioritize the harmonious co-existence between human and nature in our development plans, which guide us to make significant strides in cutting energy consumption and carbon emissions. We also actively drive the establishment of green standards along our industry chain

and empower society to save energy and reduce carbon footprints with information technology. By doing so, we aim to increase our ecosystem’s diversity, stability and sustainability, and make a contribution to the "Beautiful China" initiative.

Conducting Green and Low-Carbon Operations

China Mobile incorporated carbon peaking and carbon neutrality goals (the "30-60 Decarbonization Goals") into its overall development plan. While meeting the needs of society in information services with high quality and continuously promoting 5G and data center construction, we placed a heightened focus on the management of the 30-60 Decarbonization Goals, continued to advance all energy conservation and carbon reduction efforts, and worked to make our carbon emission stable and controllable, thereby supporting the country in achieving the 30-60 Decarbonization Goals on schedule.

Building Green Networks

With setting up a green network architecture and developing energy-saving network technologies as our goals, China Mobile continued to build green networks by advancing green and low-carbon development of base stations and data centers throughout their lifecycles, and retiring and renovating equipment.

Promoting Green Energy Consumption

With the goals of electrified energy consumption and application of green power at scale, China Mobile increased the use of green energy, steadily reduced the use of traditional energy and promoted the integrated utilization of energy to achieve green energy consumption

Fostering a Green Culture

China Mobile actively promoted the vision of green development, fostered a green culture, and carried out extensive public welfare activities on environmental protection as we continuously endorsed a green and environment-friendly lifestyle.

Supporting Social Initiatives in Energy Conservation and Environmental Protection

China Mobile actively builds a green supply chain and sets green standards in the industry. We actively leveraged information technology to empower carbon emission reduction and drive the green transformation of the society. Consistently attaching high importance to protection of the ecological environment, we optimized and improved our environmental management system and continually explored new methods and paths of environmental protection, thereby promoting harmony between humanity and nature and contributing to the conservation of our beautiful planet Earth.

Empowering Carbon Emission Reduction in Society

We actively leveraged information technology to drive carbon emission reduction, accelerated the low-carbon and clean supply of energy, promoted the low-carbon transformation of energy-intensive industries, and helped enterprises save energy and reduce emissions. We worked to reduce "carbon footprints" (i.e., greenhouse gas emissions from human activities) while creating more "carbon fingerprints" (i.e., human initiatives to reduce greenhouse gas emissions). We actively promoted information-based applications in pollution prevention and control and developed "smart environmental protection" solutions.

Protecting the Ecological Environment

In strict compliance with the Environmental Protection Law of the People’s Republic of China and relevant laws and regulations at places where it operates, China Mobile managed the full process of production and operation in a standardized fashion, adhered to economical and clean

development, and built itself into a resource-saving and environmentalfriendly enterprise. We attached great importance to environmental protection and remained committed to respecting, adapting to and protecting nature. We continued to optimize and improve our environmental management systems, made sustained efforts to protect biodiversity, and helped foster a new relationship where humanity and nature could live and prosper in harmony.

CHINA MOBILE (HONG KONG) LIMITED

promote honest and ethical conduct, including the ethical handling of conflicts of interest; • promote full, fair, accurate, timely and understandable disclosure; • promote compliance with applicable laws and governmental rules and regulations; and • deter wrongdoing.

Each Covered Officer must: • act with integrity, including being honest and candid while still maintaining the confidentiality of information where required or consistent with the Company’s policies; • observe both the form and spirit of laws and governmental rules and regulations, accounting standards and Company policies; and adhere to a high standard of business ethics.

Recognition

Telecom Review Excellence Awards 2022 “Best Asian Operator”

Telecom Review Excellence Awards 2022 “Best Global Carrier Enterprise Service”

Telecom Review Excellence Awards 2022 “Best Global Green Technology”

Global Telecom Awards 2022 “Most Innovative Cloud Offering”

CC Global Awards 2022 “Best Innovative MVNO Operator of The Year”

CC Global Awards 2022 “The 5G Deployment Award”

Global resources

Submarine Cables Resources

9 self-built submarine cables

54 submarine cable resources

Invested Terrestrial Cables Resources

8 invested terrestrial cable resources

PoPs

225 PoPs worldwide

Data Centers

4 self-owned data centers

Global Footprint

36 Global Business Support

28.Henan Rebecca

Company profile

Henan Rebecca Hair Products Inc. is the biggest hair product making company and the only listed company of hair product trade in China. Our company is located at 666 Rebecca Avenue, Xuchang City, Henan Province, China. Our company has 10300 employees, including over 900 technicians and over 500 senior level technicians. Our company has total assets of US$0.28 billion and covers an area of 500,000 square meters including a construction area of 20,000 square meters and a greenbelt area of 87,000 square meters. Our company integrates scientific research, development, production and sale. We have 5 series of products, such as 100% human hair weaving, 100% synthetic hair weaving, wigs and other hairpieces and Men's Toupee and mannequins, totally more than 2000 kinds of products. Our products sell well in more than 30 countries and regions, such as North America, Europe, Africa and Asia. Our annual export volume

reaches over US$180 million. Our company is advancing with the times. The multiple economic indexes of our company are in the first place in the field in China. Our export value was US$90.62 million in 2004, US$100.9 million in 2005, US$143 million in 2006, US$168 million in 2007 and US$180 million in 2008. Our yearly sales income is US$171 million, and yearly tax revenue is US$ 8 million, which made a great contribution to the economic and social development of Xuchang City. Our company persists in the policy of "Quality first, clients first" and strictly implements quality management and control systems according to the national and international standards. Our company is the only one in the hair product trade of China. We have attained ISO9001:2000 International quality management system certification, ISO14001:1996 International environment system certification, OHSAS 18001:2001 professional health and security system certification and IQNET (International attestation league) quality system certification.

Henan Rebecca Hair Products Co., Ltd. rises in the hinterland of the Central Plains, is located in Xuchang Rebecca Avenue, Xuchang City, which is develop from its predecessor, Xuchang County Hair Products Factory, built in 1990 and Sino-US joint venture Henan Rebecca Hair Products Co., Ltd. ,formed on March 20, 1993. The overall restructuring of the joint-stock companies of it happened on October 24, 1999. The joint-stock companies officially launched in the same year on November 11. Rebecca came into the market on the Shanghai Stock Exchange on July 10, 2003, which became the hair products industry's first stock. Currently, the company has total assets of 3.7 billion yuan, more than 11,000 employees, covers an area of more than 700,000 square meters, including construction area of more than 500,000 square meters. The company integrates scientific research, development, production, selling of hair products and hair fiber raw materials into one body. And her fashionable products satisfies many domestic and international consumers. There are six series of hair products such as 100% human hair wvg, lady’s wigs and hairpiece,100% synthetic hair wvg, Lesson wig, Men Toupee fiber hair and more than thousands kinds of products made in the company, which sell well in more than 40 countries in North America, Western Europe, Asia, Africa, and more than 100 domestic cities.

During two decades, Rebecca strictly obeys state laws and regulations, and earnestly implements the scientific concept of development, adheres to people-oriented management concept, vigorously carries forward the enterprise spirit of the "good faith, innovation, development", actively implements the "talent, technology, brand "grand strategy, and makes full use of the trade advantages. The company sets market as direct, economic benefits as core, to create and achieve customer dream as its mission, to technological progress and improve the overall quality as the driving force, to adjust products structure and upgrade as the main aim. The company continuously increases technological innovation and products creation, makes efforts to continuously improve the independent innovation and R & D capabilities, and makes efforts to build international enterprise group that owns the intellectual property rights and well-known brand

The company persists on the quality policy of "Quality first, and start all over again from beginning," took the lead to pass ISO9001: 2000 international standard quality system attestation , ISO 14001: 2004 international standard Environment System attestation, OHSAS18001: 2001 standard occupational health and security system attestation, IQNet (International Attestation League) quality system attestation. In 2005, 2006, 2007, and

2008"Rebecca brand ” hair products, were respectively appraised to be Henan Famous Brand product" and "Henan Famous Export Brand." In 2009, Rebecca trademark was accepted as Chinese famous Trademark by SAIC Trademark Office.

Over years, the company adheres to science and technology, promote technological progress, had set up a hair products Engineering Center, Corporate Technology Center, National Model Productivity Promotion Center, post-doctoral science research station, assumed independent innovation demonstration projects, drafted standards preparation of the national hair products industry standards and Henan province, completed a number of scientific research and technology patents, effectively promote the company's product structure adjustment and industrial upgrading, which has played a positive role in promoting an international enterprise group that owns the intellectual property rights and well-known brand.

Rebecca Company keeps making progress, has acquired many honor titles such as "Industry Profits Companies of 20 Strong in Henan Province, the" Outstanding Private Technology Enterprises in Henan Province, Henan Province Private Enterprises Export Champion" "Private Enterprises in Henan Province Tax-paying of one hundred Strong." Private Enterprises Star in Henan Province "Top 100" of Private Enterprises in Henan Province, Henan Advanced Management Enterprise "," Henan Province Credible Enterprise "," Henan Credit and Faith Taxpaying Large Family ” , "China Light Industry 500 strong" and was identified as "National High-tech enterprise", "National Sparkle Extroversion-type Enterprises," "National Industrial Tourism Demonstration Sites "

Research strength

A Brief Introduction to Henan Engineering Research Center of Hair Products

In November, 2009, technology center (hereinafter refers to as the center) of Henan Rebecca hair products, Inc. was identified as a state-level enterprise technology center by the National Development and Reform Committee, Ministry of Science and Technology, Ministry of Finance, the General Administration of Customs, and the National Taxation Bureau. It is the sole state-level enterprise technology center in domestic hair products industry.

The center is an integrated entity which includes technology and product research, development, communication, decision-making, service as. Its development direction, target, main task and other relevant information are as follows:

Direction: Aiming at the international research progress of hair products range, product fashion trend, actively carry out the scientific research of the hair industry, which will make the center becoming the technical distribution center and radiation center as well as the cradle for talent.

Target: The center will be established into national hair products industry technology innovation center, the hair products industry standard center, the hair products industry testing center, the hair products industry information center, the hair products industry training center for talent, and the comprehensive technology platform integrated many kinds of functions.

Task: The research, promotion and application of new materials, new equipments, new products, new technology, new agents and new procedures; the research and design of high value-added and functional and new product; the transformation and industrialization of scientific and technological achievements; Carrying out the research for the tested programs and methods for the hair industry by using the high and new developed technology.

The Center possess its own complete laboratory facilities and professional experimenter,

and which has established laboratories of hair dyeing, human hair weaving, mannequin, synthetic wig, synthetic hair weaving, additives, humanoid hair fiber materials, physical and chemical rooms, pilots and other advanced production lines. In 2011, the center built two test lines for wet spinning and melt spun, equipped with 3 D color laser scanning microscope, intelligent moisture inspecting instruments, and other test facilities. Furthermore, its modern information processing system has extended to foreign R&D platform, and filled the need of product research and development, and quality testing, etc.

In the future development, the center will continue to work hard to conduct extensive exchanges with related domestic research institutions and academic organizations, and strengthen the ability of independent innovation. Surrounding the goal of the "two have", it will implement technology leading strategy, develop humanoid hair fiber materials, new technology of hair products, and new process, pursue original innovation, emphasize on integrated innovation, and promote its dominant products to reach international first-class level. It will also adhere to "innovation" and combination of "production, study, research ” , build first-class technical team, track worldwide advanced technology, actively preempt the summit of industry technology, develop core technology with independent intellectual property rights to promote progress of industrial technology and also to promote the national economy to realize sustained, rapid and healthy development, and contribute to the rising of central China!

Honor

Advanced Group of the National Light Industry

Innovative Enterprise

Advanced Enterprise

Excellent Group of National Business System

Key High-tech Enterprise of National Torch Plan

State-Certified Enterprise Technical Center

President Speech

Experienced more than 20 years ups and downs in the international market, Rebecca grown up from a hand-workshop to the world’ s biggest professional hair products company and the first listed company of hair products, from specializing hair raw material trading and processing to a company managed six series of thousands kinds of hair products, export to more than 40 countries. We’ ve strived hard! Thinking the past, I sincerely thank the leader at all levels and the community friends who have given your utmost concern and support for the development of Rebecca, my sincere thanks also to the friends both at home and abroad which have been cooperated with Rebecca, to the Rebecca people who made this miracle happen – the people I affectionately known as the Most Lovable People consisting the unity and hard work team.

We’ve tried hard to achieve it and we should try harder to promote. Rebecca's vision is the construction of intellectual property rights and famous brands of the internationalized group company. To achieve this objective is not easily move, we must continue to uphold the spirit of enterprise of "sincerity, innovation, development", uphold and carry forward the work style "said then try, try till done", conscientiously follow the corporate mission ‘ innovation, create, complete dreams " and strive to practice values of "strengthen Rebecca, perfect individual, to serve the country and the citizen ", Use our wisdom and sweat to create a Rebecca company a more brilliant tomorrow!

Brand information

Rebecca is multinational enterprise where produce hair goods, the drafter of the industry standard, Rebecca company high-end brand. Rebecca identity elegant, noble, core consumer groups for fashion, independent of the modern women's. After years of development, now Rebecca in the country has set up nearly 200 stores, cover nationwide first-tier cities and second-tier cities, firmly occupied domestic high-end wig market.

Sleek brand was born in 1989 and headquartered in London; Europe has branches in many nations. Since the establishment, since, Sleek brand is to lead the hairstyle tide for the target, has now become popular European fashion wig brand of one of first choice.

In 2010, Sleek landed in China market. Sleek (China) uphold the British taste, in order to realize the dream of women in pursuit of perfect for mission, and constantly create amazing fashion wig, obtained the a great many hobby beautiful, reveal personality customers the praise and favour.

JOEDIR 、BLACK PEARL and STYLE ICON , is Rebecca Fashion Company in West Africa 、South Africa and EastAfrica region as the pursuit of high quality life of women who launched the high-grade hair brand. Since the establishment, brand, with its high quality products and diversified styles, quickly won a majority of female beauty the trust and support. JOEDIR、 BLACK PEARL & STYLE ICON has been constantly try to use the new craft and technology, creating better products to help do women beautiful and confidence interpretation to blossom the most delicate character charm, dazzle gives the most colorful life. At the same time in stable supply of goods, good quality as the backing, for each consumer provide quality enjoy, and exalted experience, let you find a more beautiful you. Its product series: ALL IN ONE, INDIAN HAIR, SIYA and NATURE.

NOBLE 、MAGIC and FASHION IDOL is my company in Africa, Nigeria, Ghana, Kenya,Tanzania, Congo (gold) and South Africa and other countries of the mass chemical fiber products brand. Under the covers brand GOLD series mid-range synthetic hair weaves, human hair wigs products; CLASSIC series top-grade HT fiber weaves, high temperature wire spring silk dress put (LACE wig) products. Brand in early 2000 the African market so far, already from an unknown brand, development up to now in short supply situation, with the vogue, changeful style lead hairstyle agitation, attracted numerous customer's loyalty to follow. And by its stable supply of goods, changeful style, functional sex is strong of the original silk unique advantages of the millions of African women love become the first choice. Its brand localization is: the fashion, parity, tide, changeful.

Enterprise Culture

First, Guiding ideology of corporate culture

Rebecca Group Company is not only the unity of the assets, personnel and management, and has the uniform requirements of corporate culture. To adapt to competition in the marketplace, building and nurturing a good corporate culture, the formation of common values , the integration of a variety of internal relations, and this "link" unify thinking and pace to achieve the company's continued rapid development.

Second, Two system of corporate culture

Rebecca corporate culture includes the soul system and the carrier system. The soul system includes the corporate historical mission, business philosophy, the speculative target vision and leadership, business philosophy and strategic point of view. Carrier system includes the business environment, corporate image, the system model, corporate identity, staff, the overall image, work standards and work clothing norms, cultural activities, theme educational activities and so

on.

Third, Approach to business

The company Earnestly implements the scientific concept of development , in strict complies with state laws and regulations , establishes a standardized, improved corporate governance structure , enhances the management level ; adhere to market-oriented economic efficiency as the main line to the product structure adjustment and industrial upgrading in order to technological progress and improve the overall quality as a driving force to maximize profits ; increase capital operation , and increase the core competitiveness ; people-oriented enterprise orients people, people treat enterprise as home, build a harmonious company , to build a better home ; to seize the opportunity to accelerate development .

Fourth, Corporate strategy

1. Talent Strategy

Taking a combination of training and the introduction, the company creates a large number of the management of complex, technical professional staff intelligence personnel as soon as possible to ensure fast and sustainable development of the corporate health.

2. Science and technology strategy

The company increases investment in science and technology, improves the scientific research system, and promotes the construction work of the independent intellectual property rights, constantly enhances the independent innovation and R & D capabilities, and promotes structural adjustment and industrial upgrading.

3. Brand strategy

Establish high-quality consciousness and brand awareness. With "high quality" as the guarantee and the "high standards" as the goal, we strive to create the autonomous well-known brand, implement brand drive, and constantly expand the domestic and foreign market.

Fifth, Enterprise spirit

Absolute sincerity, innovation, development

Sixth, Enterprise mission

Innovation, creating and realize customer dream

Seventh, Enterprise vision

Construction the international enterprise group of "with independent intellectual property rights, with independent famous brand"

Eighth, staff values

Grow Rebecca, perfect oneself, serve the country and human

Ninth, Enterprise ideal

1: Four mechanisms: The competition mechanism, the supervision mechanism, the incentive mechanism and the constraint mechanism

2: Four innovations: Technology innovation, management innovation, system mechanism innovation and enterprise culture innovation

3: Four management modes: Institutionalization, standardization, routinization and elaborate

4: Quality concept: Quality first, completely from scratch; Keep improving, conscientious and meticulous.

5: Service concept: To do everything possible to create satisfaction

6: Outlook: Never satisfied and pursuing forever

7: TALENT PHILOSOPHY: Having both ability and political integrity, abnormality reuse;

With virtue but having no ability, cultivate use; Have no ability and virtue, resolute disuse!

8: THE HARMONY CONCEPT: PEOPLE FIRST, CARE STAFF, EMPLOEE BENEFIT IS NO SAMLL MATTER. Constructing the harmonious company and making their homes better.

9: WORK GUDIELINES: Streamlined work procedures, improved executive capability and enhanced handle affairs efficiency

Tenth: Enterprise logo

1. Depends on Rebecca English initials "R" as the design element

2. Two elegant hairs are its main business symbol.

3. Black head type and lady decoration means main products with guidance

4. In the types implicates flexible management strategy and broad mind of Rebecca.

Eleventh: Staff overall images

a refined style of conversation and good appearance; standard words and deeds and conduct himself nobly; Be polite and enthusiasm obey; rigorous attitude and noble behavior; Pay attention to "SanDe" and observe discipline; Sincere innovation and Brave In Exploitation; Be loyal to their duties and do well the collaboration; Love their work and advocate science; Against waste and to pay attention to the economy; Work venture and dedicated for joy.

Remark: "SanDe" Refers to the social morality, professional morality and ethics.

Twelfth: Staff rules

Observe law and discipline, loyal to their duties and self-esteem and improvement.

Maintain company reputation and protect the interests of the company

Obey management, care colleagues and unity friendly

Took good care of public property, save money and put an end to waste

Study hard, improving quality and proficient in business

Positive enterprising, brave in exploitation and innovation contribution

Thirteenth： Employees work rules

1. In business management activities, it is not allowed to ask for or receive bribery from business association unit.

2. In communication with the business association unit, staff should stick to legal and proper vocational moral code

3. Staff shall not use internal information for private gain and damage the interests of the company

4. To strengthen team concept and the collective cooperation; Active communication and mutual learning; don’t suspect each other and fiddling within to build harmonious work environment.

5. Obey the company decision and supervisor's arrangement and complete work with the high quality; actively contribute their ideas to promote the development of the company

6. Keep improving, and constantly improve the work performance

7. Observe labour discipline; don't be late, leave early, absenteeism, take off and the string work

8. Staff shall not be the same business with company or as the position of other enterprises

29.China State Farms Agribusiness (G) Corp. Tanzania Ltd

China State Farms Agribusiness (G) Corp. Tanzania Ltd, a large-scale commercial sisal farm, is located in Kilosa County, Morogoro Region, Tanzania, and consists of Rudewa Estate and

Kisangata Estate with a land size of 6,900 hectares. It’s engaged in sisal planting, processing and sales.
China State Farms Agribusiness (G) Corp. Tanzania Ltd is one of overseas agricultural subsidiaries of China-Africa Agriculture Investment Co., Ltd. China-Africa Agriculture Investment Co., Ltd, jointly contributed by China National Agricultural Development Group Corporation and China-Africa Development Fund.
Production of sisal fibre in 2011 is 2,630 metric tons, ranking Top 3 among the Tanzania sisal estates. Years 2012-2014 would witness the peak production to be estimated at around 3,000 metric tons per annual.
China State Farms Agribusiness (G) Corp. Tanzania Ltd is the only China Company that invests in sisal planting and processing in Africa. It will take full advantages of talents, technology, equipment and management to promote agricultural cooperation and development between China and Africa and realize the complementary advantages and mutual benefit.

China State Farms Agribusiness (G) Corp. Tanzania Ltd. (Hereinafter referred to as CSFACOT) is one of the offshore companies managed by China National Agricultural Development Group Corp (Hereinafter referred to as CNADC) which is the largest central enterprise under the direct governance of the State-owned Assets Supervision and Administration Commission of the State Council.CSFACOT starts to establish in 2002, and has RUDEWA ESTATES and KISANGATA ESTATE sisal plantations , covering a land size of 6, 900 hectares (equal to 103, 500 Mu). Since the year 2000, CSFACOT has engaged in reclaiming land and growing sisals. The 1st phase of sisal planting is nearly 20, 000 Mu. Right now, the plantations of CSFACOT is in the period of high production; meanwhile, CSFACOT sets up a supplementary processing factory to match up with the annual production of 5, 000 tons of sisal fibers.During the peak of production, the total amount of sisal fibers will reach the expected production.CSFACOT always adheres to the business concept of " to compete by means of high grade quality, to develop on the basis of striving, and to win by sincerity and honesty." And CSFACOT stresses the importance of product quality and good service.The sisal fibers produced by CSFACOT are very popular and in widespread use in European countries, America, Africa and Asia because our sisal fibers are highly tensile, tough and wearable, free of static electricity, no brittle failure and unlikely to go rotten and so on.CNADC will continue to increase the investment in the sisal project of CSFACOT. It is predicted that CSFACOT will develop into a leading sisal enterprise in Tanzania within a few years because of its up-to-date technology, best product quality, dynamic development and large-scale plantation .

30.Boomplay

Boomplay is the leading music streaming and download services in Africa, provided by Transsnet Music Limited that aims to empower the African music eco-system to unlock its full potential. As of July 2021, it has over 60 million monthly active users and an expansive music catalogue of 60million songs. The app is available globally on mobile through the Google Play Store and App Store and on web via www.boomplay.com.
Chinese music streaming company Transsnet, through its mobile app Boomplay, has become the largest streaming music service in Africa with an estimated 50 percent of the market. It has 46

million users in Africa with a catalog of around five million videos and songs for users to choose from, according to CNN. Transsnet Group is a joint venture between internet firm NetEase Group and mobile phone manufacturer Transsion.
Boomplay (formally known as Boom Player) is a music and video streaming & download service developed and owned by Transsnet Music Limited.
Boomplay’s vision is to build the largest and most reliable digital music ecosystem for artists and content creators in Africa. The Boomplay app which won the “Best African App” at the 2017 Apps Africa.
Awards has over 42 million users as of February 2019. It is currently the biggest and fastest growing music app in Africa with a catalog of 5 million songs and videos which is still growing day by day. Users can stream songs and videos online free of charge, subscribe to one of the daily, weekly or monthly plans to save songs and videos to play whilst offline or purchase content to download. The service is currently available on Android, iOS, and the Web.
31.Transsnet
With the continuous improvement of African infrastructure and the continuous growth of Internet consumer demand, the future development of African Internet business has shown great potential and space for growth. Focusing on creating the most favorite Internet products for African users, TRANSSNET combines NetEase’s strong technical strength and advanced Internet product operation concepts with TRANSSION’s mature channel resources and solid market foundation in Africa. Based on this, we will continue to achieve more powerful technological innovation and broader geographical coverage. Through unremitting efforts in the field of localized content, TRANSSNET is committed to bringing more convenient and diversified Internet experience to African users, and continues to grow into the most influential technology leader company in the African market.
TRANSSNET Group is a joint venture company established by China's leading Internet company NetEase Group (NASDAQ: NTES) and TRANSSION Holdings Group, a global provider of smart terminal products and mobile value-added services. From music, short videos, social media to payment services, TRANSSNET focuses on the mobile Internet field and is committed to providing diversified online services to the vast number of users in Africa. As the first Chinese Internet company to penetrate the African market, TRANSSNET has now become one of the Internet companies serving the most users in Africa with excellent online applications such as Boomplay, the largest online music platform in Africa, and Vskit, the No. one short video social platform in Africa.
Culture
Honest pragmatic efficient
"To become the most influential and popular integrated Internet service provider in Africa"
"To provide uses with high-quality Internet integrated services: Create fulfillment for employees; Create value for shareholders"
32.VSKIT
Vskit is the world's unique short video community product focusing on African users. Since its development in 2018, Vskit has fully covered all countries in Africa, with more than 30 million monthly active users, and over 2,000 exclusive online celebrities on the platform. It has become one of the most popular short video products in Africa.Funny social video creation and sharing platform designed for Africans!

33.PALMPAY

Palmpay is a mobile payment product, it has provided services to Nigeria and Ghana, serving millions of users and processing monthly payments of hundreds of millions of dollars. PalmPay plans to provide world-class finance service to hundreds of millions of Africans in the next 5 years. In addition to basic transfer and payment functions, PalmPay is committed to providing more user scenarios to meet users' all-round payment needs such as clothing, food, housing, transportation, education, and entertainment, bringing more innovative payment methods, and creating a service ecosystem of mutual prosperity and mutual benefit.

Building The Future Of Finance In Africa

PalmPay is redefining the payments experience for consumers and businesses in Africa by making financial services more accessible and affordable.

We Use The Power Of Technology To Accelerate Financial Inclusion

Since PalmPay first launched in Nigeria and Ghana in 2019, we've provided over 5,000,000 customers with convenient and affordable digital payments. For around 20% of them, their PalmPay app is their first formal financial account.

We are gearing up to reach more people, faster. Our goal over the next 3 years is to enter more markets and empower over 100 million consumers and 10 million businesses with access to relevant, reliable and affordable financial services.

To achieve this, we are building out an online and offline payments ecosystem that brings together the best digital products from across the marketplace along with our own innovative features, and makes them available to anyone with a smartphone.

Thanks to our exclusive partnership with TECNO， INFINIX and ITEL Mobile, three popular mobile phone brands on the continent, PalmPay will come baked into the core user experience of new smartphone owners. So consumers are empowered with a bank in their pocket, out of the box.

Our enterprise products allow our clients to leverage the reliable network and consumer-focused solutions we have built to grow their own business. We offer tools to help them make and receive payments, manage business processes and grow their revenues and customer base...

Building A Pan-African Financial Ecosystem

PalmPay makes it easy to send and receive money and pay for airtime, bills and shopping, all while saving money through discounts and cashback.

In our main market Nigeria, we are one of the fastest-growing fintech companies with over 5,000,000 users.

And our customers can depend on us: In a recent survey, 99% of our users have voted PalmPay as the most reliable payment network in the country.

Our Values

Putting the customer first

We seek to understand our customers and put their needs and goals at the center of our product innovation.

We are driven by a service mindset and strive to deliver consistently positive customer experiences.

Team work makes the dream work

We support and trust each other and collaborate effectively with people of diverse cultures and backgrounds.

We are open and transparent in our communication and sincere and pragmatic in our approach.

Keep innovating
We are ambitious in our vision, draw inspiration from the world around us, and make decisions using local insights.
We continuously iterate and improve on our product and approach
Passion for excellence
We are ambitious with our goals and take ownership of driving results
We continuously strive to do our best and pursue growth and learning
Open and Transparent
We are open and transparent in our communication and sincere and pragmatic with our approach
Entrepreneurial accountability
We treat PalmPay as if it was our business and make decisions in the best interests of our customers, company and brand.
34.More
MORE is the first social media based on the local market and dedicated to high-quality content sharing platform in Africa. In addition to using artificial intelligence technology to create a personalized content information flow for users, MORE also provides an interest group function, allowing users to meet like-minded friends from all over Africa.
35.NetEase
NetEase is a leading Internet technology company in China. It was founded by Mr. Ding Lei in June 1997 and was publicly listed on the Nasdaq Stock Market (NASDAQ: NTES) in June 2000. At present, NetEase has covered more than 900 million users in China, providing diversified Internet services such as mailboxes, e-commerce, games, live video, news media, content communities, and online education.
Adhering to the people-oriented talent strategy, NetEase is eager to attract, retain and motivate the best talents, advocates providing employees with a relaxed office atmosphere and good living conditions, and provides a platform for rapid growth and sufficient development space for outstanding talents.
NetEase, Inc. (NASDAQ: NTES and HKEX: 9999, "NetEase") is a leading internet and game services provider centered around premium content. With extensive offerings across its expanding gaming ecosystem, the Company develops and operates some of the most popular and longest running mobile and PC games available in China and globally.
Powered by one of the largest in-house game R&D teams focused on mobile, PC and console, NetEase creates superior gaming experiences, inspires players, and passionately delivers value for its thriving community worldwide. By infusing play with culture, and education with technology, NetEase transforms gaming into a meaningful vehicle to build a more entertaining and enlightened world.
Beyond games, NetEase service offerings include its majority-controlled subsidiaries Youdao (NYSE: DAO), an intelligent learning company with industry-leading technology, and Cloud Music (HKEX: 9899), a well-known online music platform featuring a vibrant content community, as well as Yanxuan, NetEase's private label consumer lifestyle brand.
NetEase's market-leading ESG initiatives are among the most recognized in the global media and entertainment industry, earning it inclusion in 2022 Dow Jones Sustainability World Index, 2022 Dow Jones Sustainability Emerging Markets Index, and 2023 Bloomberg Gender-Equality Index, as

well as receiving an "A" rating from MSCI.

Games and Related Value-added Services

Our game products and services are comprised of in-house developed mobile and PC games (including certain games co-developed with our collaboration partners) as well as games licensed from renowned global developers. As a global early mover that anticipated and captured the trend toward mobile games, we have significantly expanded our portfolio of mobile game offerings in recent years. At the same time, our flagship titles continue to provide solid support for our online games business with persistent longevity and user loyalty. In addition, while solidifying our leadership position in the Chinese domestic market, we have also expanded globally with launches in Japan, Southeast Asia, the United States and other international markets.

Youdao

Youdao, Inc. (NYSE: DAO) is a leading technology-focused intelligent learning company in China dedicated to developing and using technologies to provide learning content, applications and solutions to users of all ages. Building on the popularity of its online knowledge tools such as Youdao Dictionary and Youdao Translation, Youdao now offers smart devices, STEAM courses, adult and vocational courses, and education digitalization solutions.

NetEase Cloud Music

Cloud Music (HKEX: 9899) is one of the leading online music platforms, featuring an interactive content community for music enthusiasts in terms of user scale and engagement.

Innovative Businesses and Others

We also offer a wide range of other innovative services, including Yanxuan, our private label consumer lifestyle brand, www.163.com portal and related mobile app (Wangyi Xinwen) which deliver quality information to our users, NetEase Pay, an online payment platform, NetEase Mail, China’s leading email service provider since 1997 and other value-added services.

ESG

Product Quality Control

At NetEase, we stepped up our quality assurance to reinforce our management of product quality. At Yanxuan, we prioritize quality assurance and have established a comprehensive quality management system to promote the creation of products that are safe and reliable.Since 2021, Yanxuan has been operating a number of Quality Control Circle (QCC) programs, using various quality management tools to continuously improve product quality and reduce the rate of returns. A project by one of our quality control teams to reduce the return rate of irons due to product defects was awarded the "Second Prize for Quality Control Achievement in Zhejiang Province" in the 2022 Achievement Presentation by the Zhejiang Excellent Quality Management Team.At NetEase, we seek to increase our product quality while raising quality awareness among employees.

Innovation Development

Our Commitment: We are committed to the principle that "From 0 to 1 is innovation, so does 1 to 1.1 ", and we promise to keep inspiring and accelerating technology innovation to enable value creation in society. Moreover, we remain focused on maintaining a robust intellectual property rights management system to respect and protect the R&D achievements of our employees, which we believe will continue to invigorate innovation in the workplace.

Focus Areas: Innovating cutting-edge technologies and establishing systematic intellectual

property rights management are not only important to achieving our business objectives, but also to satisfying the fundamental requirements of the external market environment. We fully understand the importance of technological innovation and intellectual property rights management and also take active steps to promote inventions and creations. With continuous capacity-building in innovation and exploration in cutting-edge technologies, we can leverage the power of technology to benefit society.

Cutting-Edge Technology

Intellectual Property Rights Protection

Environmental Protection

Our Commitment: NetEase is committed to incorporating energy efficiency, clean and low carbon strategies, water use conservation and climate change risks into our policy-making and decision-making process. We will continue to identify and track carbon emissions from our own operations and our value chain and take targeted measures to reduce our carbon footprint. We will keep leveraging our influence to drive emission reduction across the value chain and embracing the opportunities and challenges presented by climate change.

Focus Areas: Climate change presents potential risks to our business and the communities in which we live and work. As global temperatures and the frequency of extreme weather events continue to increase, climate change has the potential to cause damage to our data centers, office campuses and other facilities. Therefore, NetEase must work with the community to address the worldwide environmental challenges.

Awards & Recognition

Hosts/Organizers	Awards and Recognition
S&P Global	Dow Jones Sustainability World Index
S&P Global	Dow Jones Sustainability Emerging Markets Index
Sustainalytics	2023 Top-Rated ESG Companies List
Bloomberg	2023 Bloomberg Gender-Equality Index (GEI)
Bloomberg	2022 Bloomberg Green ESG Pioneers-Projects
Forbes	The World's Best Employers
Association for Talent Development (ATD)	ATD Excellence in Practice Award; ATD Best Award
UNESCO HK Association Global Peace Centre, Rotary Action Group for Peace, Society Next Foundation	ESG Care label
World Benchmarking Alliance	World Benchmarking Alliance’s 2020 Digital Inclusion Benchmark
Bloomberg	2020 Bloomberg Gender-Equality Index (GEI)

Employee Welfare

Our Commitment: Promoting employee wellbeing and safety has always been our commitment, and we endeavor to create channels to empower employee voices. We are committed to building a healthy and pleasant workplace where our employees feel NetEase's devotion to them.

Focus Areas: We focus on preventing accidents and reducing occupational hazards in the workplace to maintain a safe and comfortable working environment.

Employee Wellbeing

Employee health and safety is a top priority in NetEase. We abide by all laws and regulations on workplace safety, such as the Law of the People's Republic of China on Production Safety and the Fire Control Law of the

People's Republic of China. For better health and safety, we organize awareness-raising seminars, improve our infrastructure and provide mental support to employees through our EAP (Employee Assistance Program). In
addition, NetEase has developed and implemented an Injury and Illness Prevention Program (IIPP) in the U.S. to protect the health and safety of our employees.
Creating Social Value
Our Commitment: NetEase is committed to creating greater value for society through various philanthropic activities to fulfill its social responsibility.
We also seek out partnerships and collaborations with all sectors of society to elevate together the industries in which we operate.
Focus Areas: Promoting community welfare by devoting resources to philanthropy is a top priority for sustainable development. We are committed
to multiple programs, such as education empowerment, caring for the vulnerable and rural revitalization.
Contributing to Social Welfare
NetEase selects areas of focus in social welfare that are compatible with deeper societal needs and our advantages to live up to our commitment
to society and public welfare.
For example, NetEase Games established the NetEase Games Corporate Social Responsibility Promotion Center in 2022, and sets up the official website (https://csr.163.com/), official WeChat account, WeChat Channel and other platforms to spread the vision of practicing social responsibility. The NetEase Games Corporate Social Responsibility Promotion Center is committed to exploring the positive social value of games through the promotion of diversified cultures, learning through fun, youth education and the development of interdisciplinary exchanges and cooperation between games and academia, science and technology, education, charity, and other fields.
36.Transsion
Shenzhen Transsion Holdings is committed to becoming the favorite smart terminal product and mobile internet service provider for consumers in emerging markets. TRANSSION owns well-known mobile phone brands TECNO, itel and Infinix in emerging markets, as well as digital accessories brand Oraimo, home appliance brand Syinix, and after-sales service brand Carlcare. In 2019, TRANSSION's mobile phone shipments were 137 million units. According to IDC statistics, the global market share was 8.1%, ranking fourth; the African market share was 52.5%, ranking first.
In 2019, TRANSSION won the "Top 500 Private Enterprises in China", "Top 500 Private Enterprises in Manufacturing in China", "Top 500 Manufacturing Enterprises in Guangdong Province", "Top 100 Private Enterprises in Guangdong Province", "Top 500 Enterprises in Shenzhen", "Shenzhen Private Leading Key Enterprise" and other awards. On September 30, 2019, Transsion Holdings was successfully listed on the Science and Technology Innovation Board of the Shanghai Stock Exchange. Currently, Transsion's global sales network has covered more than 70 countries and regions, including Nigeria, Kenya, Tanzania, Ethiopia, Egypt, UAE (Dubai), India, Pakistan, Indonesia, Vietnam, Bangladesh, etc.
37.StarTimes

Founded in 1988, StarTimes is one of the most influential system integrators, technology providers, network operators, and content providers in China's TV broadcasting industry, and is on its way to becoming a globally influential media group.
With a global vision, StarTimes began to expand its business to Africa in 2002, and has been working closely with African governments to jointly promote digitalization and informatization. To date, StarTimes has established subsidiaries in more than 30 African countries, including Rwanda, Nigeria, Kenya, Tanzania, Uganda, Mozambique, Guinea, D. R. Congo, South Africa, etc, and has begun digital TV and online streaming operation in most of them. With more than 13 million digital TV subscribers and 20 million mobile subscribers, StarTimes has now become the fastest-growing and the most influential digital TV operator in Africa.
While growth is our objective, it is imperative for this growth to be entrenched in a strong value system: innovation, integrity, diligence and devotion. StarTimes always seeks harmony and win-win outcomes, and regards fulfilling social responsibilities as an obligation from time to time. It is thus widely recognized and respected both at home and abroad.
In the near future, StarTimes will continue to plough deep into the African market, establish its own business ecosystem, and strive to materialize its media coverage impact to make affordable business convenience available, and ensure the high integration of humanity and business for African friends.
Chinese broadcasting firm StarTimes has grown into one of Africa’s top pay-television providers, with around 10 million subscribers and established subsidiaries in more than 30 African countries. StarTimes has invested in low-cost, digital satellite television, according to CNN. In some countries, such as Kenya, StarTimes has introduced digital satellite television to rural areas that previously had limited access to a television signal.
To become a media group with international influence
Our Team
With a workforce of 5,000, StarTimes is an internationalized, specialized and localized enterprise.
Internationalization - Staff from dozens of countries, including China, African countries, Britain, France, the U. S., Portugal, Canada, etc.
Specialization: Bringing together professionals in radio & TV, communication, IT and media
Localization - Over 95% local staff in African operating countries
Core Values
Customer-centric, Fighter-oriented
Innovation, Integrity, Diligence & Devotion

38.CCIC AFRICA
China Certification & Inspection (Group) Co, Ltd (CCIC) is the one and only state-owned inspection organization in China.China Certification & Inspection Group(CCIC) is the first independent body dedicated to provide commodity certification and inspection services in China which now owns approximately 300 offices,200 cooperative labs, over 16,000 employees, with its business network covering major ports, cities and trade centers in over 20 countries and regions.
CCIC Africa was established in 2012, which is an accredited ISO/IEC17020 enterprise. CCIC Africa head office is based in Woodmead, with one Laboratory Company, 6 domestic branches in Richards Bay, City Deep, Durban, Steelpoort, Postmasburg, Port Elizabeth and 6 branch companies in Mozambique, Guinea, Nigeria, Kenya, Zambia, Sierra Leone. By the end of 2022,

CCIC Africa plans to establish more branch companies, thus expanding our inspection business into more African countries.
Currently, our business is mainly related to mineral product inspection and testing (Sampling, Supervision, Weighing, Sealing, Analysis, Draft Survey, Hold Inspection and TML/FMP), Pre-shipment Inspection (waste materials and used machinery) appointed by the Chinese government, PVOC/SONCAP authorized by SON & KEBS, agriculture and food inspection, customs return claims and certification ect.
Our reports which are accurate and traceable are compiled by professional experts with state-of-the-art testing facilities and available 24 hours, thus providing customer satisfaction and convenience with respect to supply chain management.

39.Twyford
Twyford is one of the best-known tile brands and manufacturers in Africa and South America. It is universally recognized as a benchmark for quality tiles, a symbol of Made in Africa around the world. Twyford brand was founded by SUNDA Int'l Group in 2000 and has successively established 5 factories with KEDA INDUSTRIAL GROUP CO., LTD in Kenya, Tanzania, Senegal, Zambia, and Ghana since 2016. Nowadays, Twyford and its tiles span more than 100 cities across over 20 countries worldwide, reaching over 1 billion people. Due to the great production capacity and Sales Network, Twyford is regarded as a global key player in the industry.

Our concept
People and their homes are central to the focus of Twyford. For Twyford, the meaning of home is much more than just a place to live. From your little one's first step through to their youthful years, the subtle emotions and value connotation conveyed by the family are shaping the soul of your children. Twyford tiles are always committed to creating a comfortable environment for you and your children, allowing you and your child to grow up together and giving your child a happy childhood , A home with order, beauty and love!
Our mission & responsibility
We grow together with Africa in opportunities and challenges. We combine the accessibility and affordability of our tiles with responsible business growth. The slogan "Better tiles, Better life" has always been regarded as Twyford's mission and creed.
Since decades ago, Twyford has been striving to make better tiles for everyone, everywhere in an affordable way. We are committed to providing our customers with affordable and better tiles,creating better life and communities for people, as well as better world we live in.

40.Sunda International Group
Established in 2000, Sunda International Group is one of the international trade enterprises which penetrate into overseas markets such as Africa and South America at the earliest; The Group insists on the operational concept of appointing people with merits and advancing with the times. After over ten years of efforts, the Group now has become a comprehensive transnational industrial group integrated with overseas industry manufacturing, international trade, industrial investment, consultation service, information technology service. The Group

dedicates itself to development and production of ceramics, sanitary ware, hardware accessories, daily cleaning products and personal care products. Moreover, the Group also has a marketing network covering more than 20 countries and regions.
Sunda International Group has an operational scale of more than RMB three billion. Since many years, the Group ranks in the front among the top 100 enterprises which have export business in Africa. The Group has set up subsidiaries in Ghana of Africa, Tanzania, Kenya, Cote d'Ivoire, Senegal, Uganda, Zambia, Nigeria and Peru of South Africa. Depending on its market opportunities and the resource advantages, the Group closely follows the development strategy of "One Belt One Road", develops the localized manufacturing industry in Africa. Now it has set up eight production bases including Ghana, Kenya, Tanzania and Senegal, establishing the complete production chain integrated with industrial manufacturing, network marketing, strategic purchasing, marine and land transportation and warehouse storage management.
For many years, Sunda has been insisting on human orientation, integrity operation and excellent working environment, normalized enterprise management system and complete staff training system, paying attention to the potential capacity, responsibility sense and challenge spirits of each staff and establishing a high-quality international commerce team with thousands of staff. While adapting to the changing of international operation market rapidly, the Group grasps the business opportunities constantly, wins the trust of customers with constant profits and assumes social responsibilities with sustainable operation and integrity.

"Making great achievements on the broad world platform". Sunda International Group will keep making efforts to write a new chapter. Under the "walking out" policy of China, the Group implements the "One Belt One Road" development strategy, cooperates with people from all nations, continuously reforms to improve the core competition and the operational management capacity to provide better working opportunities and development spaces for all the staff and make progress towards the goal of becoming a famous international comprehensive industrial group!

41.Bank of China

Bank of China is the bank with the longest continuous operation among Chinese banks. Formally established in February 1912, the Bank served consecutively as the country’s central bank, international exchange bank and specialised international trade bank. After 1949, drawing on its long history as the state-designated specialised foreign exchange and trade bank, the Bank became responsible for managing China’s foreign exchange operations and offering international trade settlement, overseas fund transfer and other non-trade foreign exchange services. Restructured into a wholly state-owned commercial bank in 1994, the Bank provides various financial services, and has developed into a large commercial bank delivering services in local and foreign currencies and featuring complete business varieties and strong strength. The Bank was listed on the Hong Kong Stock Exchange and the Shanghai Stock Exchange in 2006, becoming the

first Chinese bank to launch an A-Share and H-Share initial public offering and achieve a dual listing in both markets. The Bank is the official banking partner of the Beijing 2008 Summer Olympics and the Beijing 2022 Winter Olympics, thus making it the only bank in China to serve two Olympic Games. In 2011, Bank of China became the first financial institution from an emerging economy to be designated as a Global Systemically Important Bank, a designation it has now maintained for 12 consecutive years. With its growing international status, competitiveness and comprehensive strengths, the Bank has marched forward into the ranks of the world’s large banks. In 2021, the Bank prepared the Group’s 14th Five-Year Plan, clarified its positioning and comparative advantages in the domestic economy and in the domestic and international economic flows, and focused on the development of the “Eight Priority Areas” of technology finance, green finance, inclusive finance, cross-border finance, consumer finance, wealth finance, supply chain finance and county-level finance. It moved faster in forming a strategic development landscape with its domestic commercial banking services as the business mainstay, its globalised operations and diversified business platforms as the two growth engines, and worked hard to write a new chapter of building a first-class global banking group.

As China’s most globalised and integrated bank, Bank of China has institutions across the Chinese mainland as well as 62 countries and regions, and BOCHK and the Macau Branch serve as local note-issuing banks in their respective markets. The Bank has a well-established global service network and an integrated service platform based on the pillars of its corporate banking, personal banking, financial markets and other commercial banking business, which covers investment banking, direct investment, securities, insurance, funds, aircraft leasing, asset management, financial technology, financing leasing and other areas, thus providing its customers with financial solutions featuring global expertise and all-round services accessible at any point of contact.

Bank of China embodies a noble sense of duty and commitment. Over its 111 years of development, the Bank constantly cared about the nation, dedicated to contribute to the country, and continued to strive for prosperity of the country and rejuvenation of the nation. Being deeply rooted in people and committed to providing excellent services, it has fulfilled its responsibilities of delivering financial services for the people, bringing benefits and convenience to the people, and improving the living standards of the people. Keeping the common good in mind and advocating openness and inclusiveness, the Bank has mobilised domestic and overseas resources to serve the two-way interactions between China and the world. It has always followed the law, innovated with prudence, and upheld the spirit of integrity and innovation to strengthen the Bank. As a large state-owned commercial bank on a new journey towards fully building a modern socialist country, the Bank, under the guidance of Xi Jinping Thought on Socialism with Chinese Characteristics for a New Era, will carry forward the Bank’s century-old tradition of pursuing excellence, take full, accurate and comprehensive implementation of the new development philosophy, contribute to the new development pattern; bolster the Chinese modernisation, promote high-quality economic and social development, and make an even greater contribution to realising the Chinese Dream of national rejuvenation in all respects.

Bank of China, as the oldest bank in China and the first Chinese bank to have a presence in South

Africa, opened its Johannesburg Branch in early October 2000. The branch is located in Sandton, Johannesburg, the financial hub of South and Southern Africa...

Bank of China Africa Branches:

BANK OF CHINA (ZAMBIA) LIMITED;JOHANNESBURG BRANCH; Bank of China Limited Nairobi Representative Office

CSR

The Bank carries out due diligence on the environmental and social risks of customers (projects), with a focus on their compliance and compliance risks, including their pollution prevention and control measures, the characteristics of their industries and localities, as well as their policies in climate change and energy management, cultural heritage protection, labor conditions and community health and safety management, and biodiversity and sustainable resource protection. The Bank makes reasonable and objective judgments on the environmental and social risks of the customers, and provides relevant evaluation and analysis in business initiation reports.

We prioritised the regional industry development and brand building in the fruit industry and animal husbandry, focused on improving water supply capability, disaster relief, and pandemic containment to consolidate the achievements in poverty alleviation, paid attention to sewage treatment, garbage disposal, and other actions to improve the rural environment, and attached importance to developing education, health care, and other public services in rural areas to promote balanced development between urban and rural areas. In the year, we invested and channeled a total of RMB107 million funds in those four counties in Xianyang, and carried out almost 100 projects concerning people’s livelihood and industrial assistance, directly benefiting more than 30,000 people. Through those efforts, we contributed to the construction of beautiful villages.

Message from Chairman

Sustained actions will lead to a sustainable future. Bank of China adhered to the original aspiration, pursued excellence and fulfilled the mission of “Bridge China and the World for the Common Good” with actions in the significant year 2021. From serving the front line of micro and small businesses to supporting cross-border cooperation of green development, from the farm fields of Yongshou County, Xunyi County, Chunhua County and Changwu County (“four North-Xianyang counties” hereinafter) in Xianyang City, Shaanxi Province, to the ice and snow tracks of Beijing 2022 Winter Olympics, we contributed to the all-round development of economy, society and the environment with high-quality financial services, played an active role in serving and integrating into the new development pattern, and continued to create value for shareholders, customers, employees and society. We adhered to our distinctive advantages, laid out the pattern featuring “One Mainstay, Two Engines”, thus to contribute our shares to the new development pattern. In the face of a confusing and changing international landscape, we always upheld openness, inclusiveness and win-win cooperation, and built a strategic development pattern where domestic commercial banking serves as the main body flanked by the two wings of globalised and integrated operations. We also set up an operation system featuring accessing the Bank’s global resources and services at any point of contact. In addition to steady promotion of overseas institution layout, we constantly enriched cross-border products and services, fully participated in the implementation of dual listing of H-Share and piloting southbound trading under the Bond Connect programme. With these efforts, we supported high-quality “going global” and “bringing in” by acting as a financial bridge and bond to

connect China and the world, and put into practice the concept of a human community with a shared future with concrete actions. Moreover, we leveraged the full-licensed operation advantage in stocks, loans, bonds, leasing and insurance, deepened the cooperation between commercial and investment banks, offshore and onshore institutions, and provided market entities with whole lifecycle financial services covering the entire industry chain, thus to boost the modernisation of industrial and supply chains. We kept in mind the original aspiration and mission, and focused on the eight priority areas for enhancing its financial services capabilities to empower a high-quality development. Centring on the new business forms and new trends of economic and social development, we improved the quality and efficiency of financial services starting with the eight priority areas of serving technology, green finance, inclusive finance, cross-border business, wealth management, consumption, supply chain and county development, to cultivate new drivers and new modes and serve new business forms. We focused on supporting key areas and weak links of economic and social development. The balance of financing for core enterprises of the supply chain amounted to RMB4,732.1 billion, that for upstream and downstream enterprises of the industrial chain stood at RMB921 billion and new inclusive finance lending to micro and small enterprises notched a record high. In promoting a comprehensive and sustainable development, we also designed green planning and action programmes, launched various innovative products in green funds, wealth management, leasing, insurance etc., and greatly supported the carbon control and emissions reduction, clean energy promotion as well as transformation and upgrading of traditional industries. We stood solidly on the people’s position, cultivated human-based culture, and contributed to a better life. We promoted corporate culture in a thorough manner, centring on customers outside the Bank and on employees within the Bank. We advocated values of “providing excellent service, innovating with prudence, upholding openness and inclusiveness, and collaborating for mutual growth”, and acted on the mission of providing finance services for the people’s good to help them prosper and bring them benefits and conveniences. We devoted ourselves in the rural revitalisation cause by searching for programmes, introducing resources and building platforms for the development of agriculture and rural areas, and unblocking the channel of poverty alleviation through consumption, thus helping agricultural products from the mountainous areas debut on the international exhibitions. Relying on the BOC Charity Foundation and Bank of China Philanthropy, we explored the mutual promotion between finance and public welfare. In the past year, we supported 188 public warfare programmes initiated by 73 institutions, raising charity funds of RMB17.3631 million (matching gifts inclusive) in total. And we participated in the Olympic Games again after the 2008 Beijing Olympics by provided quality financial services for the “simple, safe and splendid” Winter Olympic Games in a comprehensive manner, making the dream of “Inspire 300 Million People to Participate in Ice or Snow Sports” come true. We embraced the wave of technology and moved forward with digital transformation to deliver excellent service to the society. Grasping the opportunity of another round of technological changes, we carried out enterprise-level architecture building, data governance, building of a scenario-based financial services ecosystem, product innovation, the reform of technological system and other key projects in a coordinated manner, and accelerated the digital reconstruction of our mode of thinking, business philosophy, customer service, product innovation and risk control, in a bid to empower our employees and serve the society. We also made breakthroughs in the digital business model, launching products such as “BOC Cross-border E-commerce Connect” and

“BOC E-cooperation”, and released mobile banking version 7.0 to better digital services in key areas. Focusing on crossborder, education, sports and silver-haired scenarios, we continued to strengthen basic capacity building and be more community-focused. To eliminate the digital divide, we provided exclusive and convenient services to the senior people by launching a specific version of mobile banking for the elderly and smart counters for seniors, aiming to make technology more humane and services more considerate.

We upheld long-termism, strengthened comprehensive risk management, and created value with a sound development. We continuously improved the corporate governance system, promoted the integration of ESG concepts into corporate governance with focuses on ESG issues such as green finance, inclusive finance, consumer rights protection and targeted poverty alleviation, and clarified the responsibilities of the Board of Directors and Board of Supervisors. Facing the complex and serious challenges at home and abroad, we improved the building of a comprehensive risk management system, re-examined the Group’s risk appetite with appropriate adjustments, and assessed global macro policies, debt structure and capital flow trend in a forward-looking manner so as to ensure the liquidity safety, stable asset quality and continuous compliant operation of overseas institutions. Besides, we strengthened the building of a digital fraud risk prevention system and improved the real-time anti-fraud capability in online finance. As a result, the number of suspicious transactions intercepted by the “Network Defence” system reached 879,000 in 2021, well safeguarding the security of clients’ funds and property.

Bank of China will celebrate its 110th anniversary in 2022. Over the 110 years, Bank of China has always adhered to and passed on “serving society, and contributing to national prosperity” through generations in an unyielding struggle, be it from the time when fulfilling the duties as a central bank to consolidate the results of the Revolution of 1911 to the time when raising foreign exchange funds to support the anti-Japanese war, or from supporting major equipment imports and developing foreign trade in the period of reform and opening up to serving the real economy and supporting poverty alleviation in the new era. Bank of China has made important contributions in different historical periods. From this new starting point, Bank of China will continue to draw on wisdom from the century-long history, and promote the fine tradition in pursuing excellence as a century-old enterprise. We will stay resolved to serve the people and the country, enhance our capabilities to achieve the intended goals as planned, cultivate the drivers to innovate with future-oriented views, and strive to build a first-class global banking group. We will also strive to yield more good returns while serving the new development pattern, make greater contributions to the continuous and healthy development of economy and society, and advance hand in hand with stakeholders towards a better future with greater prosperity and mutual growth.

42.EL-ALAN Construction Company (Nigeria) Limited

EL-ALAN Construction Company (Nigeria) Limited (EL-ALAN or the Company) was founded in April 1982 as a private limited liability company and today is one of the fastest growing and most dynamic construction and civil engineering companies in Nigeria. For over three decades El-ALAN has delivered high quality integrated construction solutions.This rapidly expanding Chinese construction company, which is based in Ikoyi Lagos, El-Alan, is renowned for providing its clients in the government and the general public with high-quality integrated building solutions.

Our initial focus was renovations and sub-contract work through which we quickly gained a name for ourselves. Our success and reputation today are built on the consistent delivery of quality,

speed, efficiency and overall client satisfaction.
We have expanded our operational and service offering to cater to a wide spectrum of construction needs in both the public and private sectors. Our portfolio of work comprises successful projects in the residential, commercial and industrial spaces, working with clients across all sectors and with differing budgets..
We are an associated member of a group of companies that provide services across all areas of the construction industry. Through our associated companies we have access to world-class ideas, skills and innovative technology which puts us in a unique position where we are able to offer integrated and value-added services to our clients.
Unlike so many of its competitors, EL-ALAN has weathered more than one economic recession and has come out the other side stronger than before. “We have survived all this time in part due to perseverance,” says Geday. “We tighten our belts and focus on delivering a quality product, no matter how tough the economy. This has paid dividend on a number of occasions, because when the market picks up again, we have less competition.”
EL-ALAN has also been incredibly smart when it comes to diversifying its product and service offering. “We have developed new complementary arms of the business including MEP building services, Property Development, Facility Management and Logistics,” says Geday.
“We provide complete construction solutions. The majority of our contracts are turnkey projects. We are involved in each stage of the project, from architectural design, planning approval and construction. We also provide building maintenance and facility management solutions. This holistic approach delivers cost effectiveness, streamlines project management and shortens construction timelines.
“We have truly developed a one-stop-shop service whereby we can take a building from inception to completion, even down to details such as bathroom design,” says Geday.
Not only does the company offer a complete turnkey solution, but it also has all the required core competencies in-house. EL-ALAN has a small database of trusted contractors, sub-contractors and suppliers with whom it has worked with for a long time. “This is extremely attractive to our clients,” says Geday. “Everything is managed by us and us alone, which means there is nobody else to hide behind, no excuses. Every element of the project is controlled by us, which makes completion much easier.”

OUR VISION
Vision
Our vision is to be the undisputed best in our industry, providing our customers with exceptional products and services that set the standard for quality and innovation. We strive to be the best in everything we do, from concept development and construction to customer support and beyond. By constantly pushing the boundaries of what's possible and setting the bar higher for ourselves, we aim to create value for our customers and employees. At our core, we are a company that is passionate about excellence and committed to delivering results that exceed expectations.
OUR CORE VALUES
QUALITY
We always adhere to principles of best practice and service excellence. In so doing, we have built a name for ourselves that has undoubtedly become a by-word for quality.
INTEGRITY

We remain true to our founding values of honesty and integrity and these tenets have been instrumental in our growth and success. High ethical standards and a professional culture characterise all our activities and our clients have come to expect nothing less.

FLEXIBILITY

We go the extra mile to satisfy our clients’ needs. The flexibility of our approach means that we are able to quickly adapt to changing situations and can maintain our high level of service quality at all times. We offer our clients the accessibility and flexibility of a small company with the expertise and resources of a much larger company.

COMMITMENT

We believe that the best results are achieved by the early and committed collaboration of all project-stakeholders from the design and construction process through to the entire performance life-cycle of the structure.

43.Chinaville Chinese restaurant

Chinaville Chinese Restaurant is located in Victoria Island, Lagos. With a reputation for offering world-class authentic and oriental Cuisine, this Chinese-owned restaurant in Victoria Island has become a hot spot for lovers of deliciously made Chinese Cuisine. Along with serving delicious meals, this restaurant is also known for its fine dining and private atmosphere.

This Chinese-owned restaurant in Victoria Island has earned a reputation for serving top-notch authentic Chinese cuisine and has become a popular destination for those who enjoy deliciously prepared Chinese cuisine. In addition to delivering excellent meals, this restaurant is renowned for its fine dining and elegant setting.

44.North China Construction Nigeria Limited (NCC)

As a division of the renowned North China Construction Group Co. Limited of Beijing, the North China Construction Nigeria limited is known for undertaking complex engineering designs and construction projects in the country. Operations carried out by this company include; roads, seaports, airports, harbors, stadiums, and a lot more.

North China Construction Nigeria Limited (NCC) is the Nigerian Subsidiary of North China Construction Group Co. Limited of Beijing, China. The North China Construction Group Co. Limited is a Chinese Government owned company with strong Asset and Financial base and having an AAA Credit Rating.

The major reason for entry into Nigeria is to implement the Group’s Global Strategy of “going abroad” having applied for and passed the National Contracting Foreign Engineering “Business Qualification Certificate” and having pooled Excellent talents to develop a strong International Engineering Contracting Business. We envision that Nigeria will be the hub of our larger African Business as the country thrives to rank among the 20 largest economies of the world and the biggest economy in Africa in the not too distant future.

Our Understanding Of The Nigerian Opportunities And Infrastructural Challenges

The greatest challenge facing the country and which may constrain Nigeria’s ability to realise her vision of becoming one of the top 20 Economies of the world by 2020 is the serious deficit in infrastructures, including roads, Rails, Sea Ports, Airports, Power, Housing etc. This deficit has two dimensions – availability and quality with the later posing a bigger challenge. In this regard, Nigerian governments at the Federal and State levels should actively seek out world-class

construction and infrastructure companies that will partner with Nigerians and Nigerian Governments to solve the infrastructural challenges qualitatively.

OUR STRATEGIC THRUST

North China Construction Group Co. Limited is a Chinese Government owned integrated Design, Engineering and Technology Company that has the mandate of the People’s Republic of China to further the Sino-African Strategic Partnership. Consequently, our proposed engagement with Nigerian public and private sectors is far beyond mere business to business relationship. We are not just contractors because our mandate for Nigeria goes far beyond that. North China Construction Group is a “POLICY” company with a mandate to pursue the Chinese Government Policy in Africa with the twin objectives of delivering both social benefits and business value.

North China Construction Group Co. Limited is an “AAA” rated firm with Equity capital or Shareholders’ fund of about US$300 million. We are therefore able to leverage our balance sheet and financial strength to attract the right level of funding from Chinese Banks and Government backed Financial Institutions for our strategic projects in Africa. The government of China is also willing and able to support our activities because we are a Policy company.

The Chinese Government had set aside about US$30 billion under the China African Development Foundation (CADFUND) to promote economic Co-operation between Africa and China. Nigeria as the largest country in Africa, should utilise part of this fund to drive her infrastructural development. We intend to use our engagement in Nigeria to raise the awareness and encourage Nigerian Governments and Institutions to leverage this opportunity to create jobs and wealth for the people of Nigeria.

Core Values/Objectives

The core value of North China Construction Nigeria Limited is to build a better society by employing the latest Engineering Technology, Innovation and Managerial Know-how. Our company seeks to build a better society by equipping young Nigerians with the latest Engineering & technological skills and to foster collaboration and friendship between Africa and China in the spirit of South-South co-operation.

The choice of Nigeria for Group’s first African footprint is borne out of the fact that Nigeria is the most important country in Africa given her population and resources. North China Construction Nigeria Limited will operate within the highest standard of Engineering quality, environmental protection, and occupational health safety system authentications in line with global best practices. The company shall pursue the aggressive training and transfer of skills to Nigerians as well as the pursuit of Research and Development, in collaboration with higher institutions and Research Institutes in Nigeria, which is consistent with our current strategic thrust in China.

North China Construction Nigeria Limited takes seriously the Nigeria Government’s local content policy and shall thrive to train and promote Nigerians into strategic positions. Our current Board and Senior Management composition reflect this aspiration and strategic thrust. Our company encourages diversity as a source of strength and innovation.

45.Vitex China Nigeria Limited- Tractor Dealer in Lagos, Nigeria

lncorporated in Nigeria in 2002, Vitex China Nigeria limited is a Nigerian/Chinese owned Agent Company with a long history in sales, assembling, manufacturing, and marketing of VT-D12 Tractors in the Ecowas regions. As a sole agent of an award-winning Chinese company named Chang Chi lndustry limited, Vitex has continued to live up to the company's reputation for quality

and versatility.
Vitex China Nigeria Limited - Tractor dealer in Lagos, Nigeria. is a Tractor dealer located at 47 Oduduwa Cres, GRA, Ikeja, NG.
The business is listed under tractor dealer, agricultural machinery manufacturer, agricultural service, agricultural service supply agency, farm equipment supplier category. Their services include Delivery, In-store shopping, Same-day delivery.

46.China Harbour Engineering Company Nigeria Limited (CHEC)
CHEC Nigerian limited is a subsidiary of the famous China Communication Construction Company Limited (CCCC) specializing in marine engineering, airports, dredging,environmental protection, roads and bridges, railways, and municipal works, and complete plants.
China Harbour Engineering Company Ltd. (CHEC) was established in the 1980s. As a subsidiary of China Communications Construction Company Ltd. (CCCC) - a Global Fortune 500 company, it explores the overseas market on behalf of the CCCC. CHEC's more than 90 branches (subsidiaries) and representative offices around the world presently conduct business in over 100 countries and regions with more than 20,000 employees undertaking hundreds of international projects whose total contract amount is over USD 40 billion.
CHEC, adhering to the development direction of “platform company and industry leading”, actively promotes the overseas practice of the strategy “new CCCC in Three Cores and Five Main Fields" and strives to build itself into an internationally first-class engineering contractor,industrial investment operator, urban developer and ecological manager.In the fields of ports, construction, roads and bridges, rails,comprehensive urban development, ecological and environmental protection, pipe networks, green electricity and so on, CHEC, with advantage in integrated services for the whole industrial chain of investment-management-construction-operation-exit”,can provide high-quality products and services for global customers and continually create value for stakeholders. CHEC has constructed a lot of landmark projects with international influence, which have won a series of important awards, including“China Construction Engineering LubanPrize",“Tien-yow Jeme Civil Engineering Prize"“National Quality Engineering Gold Award,“Brunel Award", and “ENR Global Best Project". These accomplishments have transformed CHEC into a well-known brand in the international engineering industry.
Corporate Mission
Creating more value for the stakeholders
Corporate vision
Becoming an excellent organizer and leader of integrated service in international marine engineering and relevant building fields
Core value
Realizing the responsibilities, returning to the society with a high quality and establishing win-win cooperation
Corporate spirit
Inclusiveness, Integrity, Innovation and Dedication
We are building a closer world, We are sharing a better life.
Together with you, we are turning dreams into reality!
47.Jade Palace Chinese Cuisine

For decades now, Jade Palace has been the St. Matthews’ go-to for authentic Chinese cuisine. True to tradition, we serve some of our Hong Kong favorites as well as dim sum-style meals. Our family took over ownership in 2016, and we’ve been working hard to improve every aspect of our restaurant ever since. You can expect prompt service and authentic flavor every time. Next time you’re in the mood for homemade Chinese food, we’d love to welcome you in to try our menu, buffet, or Chinese brunch.
This exquisite Chinese-owned Cuisine which has stood the test of time in the competitive city of Lagos, is best known for its courteous customer services, reasonable price, variety in vegetarian options, and delicious meals. Located at 61, Adeola Odeku Street, Victoria lsland Lagos, with a branch in lkeja, Jade Palace Cuisine can be said to be presenting Chinese Cuisine excellently.
China Development Bank
Established in 1994, the China Development Bank is a state-funded and state-owned development finance institution. As an independent legal entity directly overseen by the State Council, it is dedicated to supporting China’s economic development in key industries and under-developed sectors.

With a mission to support national development and deliver a better life for the people, CDB aligns its business focus with China’s major medium- and long-term economic development strategies. Leveraging its strength as a leading bank for medium- and long-term financing and comprehensive financial services, it mobilizes economic resources and channels them towards eight key areas: (1) Socioeconomic development, such as infrastructure, basic industries, pillar industries, public services and management; (2) Urbanization, urban-rural integration and balanced regional development; (3) Programs essential for national competitiveness, including the transformation, upgrading, and restructuring of traditional industries, energy conservation, environmental protection, and advanced equipment manufacturing; (4) Public welfare, including affordable housing, poverty alleviation, student loans, and inclusive finance; (5) Areas of strategic importance, including science and technology, and people-to-people exchange; (6) The Belt and Road Initiative (BRI), international cooperation in industrial capacity, equipment manufacturing, infrastructure connectivity, energy and resources, and Chinese enterprises “going global”; (7) Initiatives in support of development and economic and financial reforms; (8) Other areas encouraged by national development strategies and policies.

Placing a strong premium on pioneering and innovation and tapping into its strengths as a development finance institution, CDB keeps closely to national development strategies, operates on market principles with the backing of sovereign credit, and ensures principal safety and modest profit. Its vigor, influence and risk resilience has been growing continuously, putting it on track to be a world-class, modern development finance institution and a steady force of support for China’s economic and social development. At the end of 2022, CDB had the total assets of RMB 18.2 trillion.

CDB has 37 primary and 4 secondary branches on the Chinese mainland,one branch in Hong Kong, and 11overseas representative offices in foreign cities including Cairo, Moscow, Rio de Janeiro, Caracas, London, Vientiane, Astana, Minsk, Jakarta, Sydney and Budapest. CDB has more than 10,000 employees in total.Its subsidiaries include CDB Capital, CDB Securities,CDB Leasing,

China-Africa Development Fund, CDB Development Fund and CDB Infrastructure Fund.
Enhancing national competitiveness and improving people's livelihood

Mission is what CDB believes in. It represents the purpose and objectives of all CDB's undertakings. Since its inception, the bank's own development has been closely linked with the national development strategy and the interests of the people.

[Enhancing national competitiveness]

As a development finance institution, CDB is duty bound to facilitate national strategies and build on the country's competitiveness. Since its foundation, CDB has made active efforts to push forward the implementation of national strategies through financial support. The bank dedicates itself to key projects of infrastructure and basic/pillar industries, urbanization development, improving people's livelihood, overseas investment of Chinese enterprises, alleviating development constraints and supporting macro regulation. The bank has helped mitigate the impact of the global financial crisis and promote stable economic growth and restructuring, contributing greatly to China's economic and social development and overall competitiveness.

Sustainable development of the Chinese economy requires development finance to continue to play its role. CDB will continue to support major national projects, increase the provision of public goods, and continuously build up economic development momentum; create new scope for regional development, and tap into economic growth potential; drive industrial restructuring and facilitate the development of new technology, new industries, and new business formats; inject new vitality into economic development; support the "Beautiful China" strategy, develop green finance, and boost sustainable economic and social development; support internationalization initiatives of Chinese enterprises, actively participate in global governance, increase China's overall competitiveness and international influence.

[Improving people's livelihood]

As an ancient Chinese saying goes, "The people are the root of a country, and a country can develop steadily only if it is firmly rooted." CDB aligns its operations with the goal of finishing building a moderately prosperous society, applying successful experiences gained in infrastructure construction to projects closely related to the people's livelihood, e.g. affordable housing for low-income groups, poverty relief, "agriculture, rural areas, and farmers" development, education and healthcare, SMEs and microbusiness projects. In addition, inclusive finance will be further developed to stimulate social progress and facilitate the building of a harmonious society.

Efforts should be made to improve people's livelihood by effectively addressing fundamental production and development issues affecting the general public. CDB commits itself to strongly supporting "shared development", pushing forward key projects related to people's livelihood, promoting social equity and justice, improving people's well-being, so that more people can benefit equally from the country's development. To this end, we will continue to innovate our

products, services and business models, and mobilize various resources to channel private capital toward projects closely related to people's livelihood; we will work to strengthen "weak links" in China's modernization drive, and make new contributions to finish building a moderately prosperous society.

Our Vision

Building a world-class DFI to provide sustained support for economic and social development

Our vision reflects our confidence in the future development of CDB. It is the goal we keep working to realize.

To become a world-class DFI, CDB continues to grow with renewed vitality and keeps building on its influence and risk-resistance capability.

[Renewed vitality for sustained innovation]

CDB needs to acquire renewed vitality to push through reform-driven development. We will rise to challenges on our way to success through reform and innovation, and keep optimizing the bank's corporate governance, operating mechanisms and procedures, service standard and operational efficiency. We will adhere to the philosophy of "people first" in driving business growth, and cultivate high quality, professional and creative talents to add impetus to CDB's development.

[Acquire strong global influence]

Growing influence is a major aspect of the role played by development finance. Domestically, CDB will maintain its position at the forefront of reform-driven development, give full support to improve people's livelihood, and guide private capital to become an indispensable economic and social growth driver in the financial institution system, thereby making even greater contributions to national development strategies. Internationally, we need to promote international exchange and cooperation, actively take part in international economic and financial governance, and promote win-win cooperation and common development, showcasing the strengths of Chinese financial institutions on the international stage.

[Strong risk-resistance capability to ensure sustained development]

Strong risk-resistance capabilities provide the foundation for CDB to support national strategies and achieve sustainable development. Adhering to the "macro risk management" approach, CDB implements effective risk prevention and control measures amid business operations while prioritizing business development as the overriding objective; keeps developing the comprehensive risk management system, and tightens up risk control; improves the internal control system and operational management, and guards against irregularities and illegal activities. We are dedicated to building the bank into a DFI with adequate capital, standardized corporate governance, strict internal controls, operational security, quality services, and safe assets.

Providing sustained support for economic and social development means that CDB should fulfill its role as a DFI in key sectors and “weak links” during critical transitional periods.

[A major role in key sectors]

It involves optimizing business coverage, focusing support on key industries and projects related to national security and economic lifelines, balancing domestic and international business development, promoting international cooperation, and playing a leading role in the implementation of China’s national development strategies.

[Remedying leading role in strengthening “weak links”]

CDB is fully committed to its social responsibilities by providing needed assistance for national development. As the “financial pioneer and coordinator”, we continuously innovate our products and business models to guide private capital to solve bottlenecks in economic development.

[Support counter-cyclical development at critical times]

CDB devotes itself to facilitating the implementation of macroeconomic control policies through medium- and long-term investment and financing activities, supporting the national economy at times of economic difficulty without interfering with the market during boom periods. Therefore, it has become the financial platform for macroeconomic regulation.
Core Values
Responsibility, innovation, green growth, prudence and win-win development

Core values are the cultural guidelines for CDB, and the shared goals uniting people at CDB in its pursuit of excellence. These core values have been at play throughout the bank’s development history and are now part of the thinking of our staff.

[Responsibility]

Responsibility gives CDB a sense of mission to support national strategies. For many years, CDB has committed itself to serving national strategies, contributing greatly to economic and social development in China by supporting the infrastructure and basic/pillar industries, people’s livelihood projects and “Go Global” efforts of Chinese enterprises.

Standing at a new starting point, DFIs have honorable lofty mission and immense responsibilities. CDB must continue to develop despite the many challenges ahead, fulfill its mission with an enterprising spirit, and live up to the high expectations placed on it by the state and the government.

[Innovation]

Innovation is what drives CDB’ s development and reforms. We have always remained at the forefront of China’ s financial reforms and managed to maintain momentum for business development through bold, open-minded innovations, actively exploring new models and methods for effective financial service development.

Under the new normal, CDB will stick to the innovation-driven development approach. It allows us to resolve problems and create new growth engines through innovation, thereby ensuring sustained and dynamic business development at the bank.

[Green growth]

Green growth is an important aspect of our social responsibilities. As one of the members of the United Nations Global Compact, CDB places great emphasis on energy and resource conservation and environmental protection. We support green, circular and low-carbon development, incorporating the notion of “green credit” into all aspects of its business operations. Over the years, we dedicated ourselves to promoting harmonious development between man and nature.

CDB will adhere to the green development principle, reduce waste of resources in business operations, and prioritize green finance in line with our commitment to combating pollution in China. In international cooperation activities, we guide enterprises to develop and manage construction projects according to international ecological protection principles and regulations. We encourage efforts to strike a balance between economic, social and environmental benefits.

[Prudence]

Prudence is the foundation for the survival and development of CDB. The bank adopts the scientific development perspective to support market-oriented operations, and continuously improves project planning, development, review, credit authorization, post-loan management, etc. We have developed risk management and internal control systems unique to CDB to ensure prudent business growth.

CDB will continue to guide its business operations with a prudential approach balancing the relationship between development, risk, and profit. We will further refine and rationalize the bank’ s management, improving the adaptability, stability, coordination of organic growth as a means of ensuring high quality and sustainable growth.

[Win-win development]

Win-win development is the prerequisite for inclusive growth. CDB gives high priority to the most critical issues in domestic and international projects alike. We endeavor to tackle the most pressing concerns and challenges facing government departments, clients and partners, and ensure enterprises and the real economy can benefit, and have therefore won the respect and trust across various sectors in the Chinese society.

On the national level, CDB always gives top priority to the facilitation of national strategies, promotes economic growth and social progress in China and safeguards the interests of investors, ensuring value preservation and appreciation for state-owned assets while promoting common growth and prosperity through cooperation with other countries; in terms of social responsibilities, we increased support for undertakings concerning people ’ s livelihood to enhance the well-being of the general public, in line with the bank ’ s commitment to inclusive development; for clients, we strive to create mutual benefit, win-win development and shared value by aligning the interests of both sides; a staff development platform has been introduced to allow employees to fulfill their potential and achieve personal development through development-oriented financial endeavors.

48.China Construction Bank Corporation Johannesburg Branch (CCB JHB)

China Construction Bank Corporation Johannesburg Branch (CCB JHB) was established in October, 2000. It is CCB ’ s advance base for development of the African market. The Johannesburg branch consists of a diversified, international and professional team. Its aim is building a business focusing growing both the domestic and foreign enterprises in support of the one belt one road initiative by providing bilateral loans (including CCB domestic guaranteed loan), syndication loans, trade finance, project finance, Renminbi services, FX trade and settlement in support of customer business requirements. The business targets clients in the integrated energy, communication, mining, financial services, trading, logistics, manufacturing and media industries, in 47 countries in the sub Saharan Africa region by providing multi facet services to our clients.

The Johannesburg branch concentrates on localization, with the majority of the clients being African corporations, including more than 20 of South African listed companies, state owned companies and international companies. The branch has also established a strategic alliance with one of the leading financial groups in South Africa. CCB JHB ’ s assets and profits are ranked high among the foreign banks in the African region, which makes it very competitive. CCB group holds the African market in high regard.

Relying on the group ’ s strong financial background and overseas networks, the Johannesburg branch has used these advantages to provide financial services and funding to Chinese companies entering the Africa markets, and simultaneously enhancing the development of Sino-African trade.

In the future, CCB Johannesburg branch aims to become a key bank in the Southern African region by actively focusing on Sino- African trade by increasing its reach and contribution as a regional organization, and providing our clients with robust and efficient financial services.

49.Jiu Hua Nigeria Co. Ltd

Based in different parts of the country, Jiu Hua Nigeria Ltd is a popular Nigerian-based company partnering with reputable Chinese companies to offer services related to material investments and developments. The company is best known for its production of doors, glass, aluminium, stainless steel, wrought iron, furniture, and sofa.

50.Tugrow1688 logistics

Tugrow1688. is an English Tugrow1688 based in China. Our mission is to make buying items in Taobao more safe and convenient by offering buying and shipping solutions to our valued clients.
We offer highly professional service rooted in our core values: Competence, Convenience, and Reliability. We are results-oriented, so you can be assured we deliver quality service. Wherever you are, we want to offer you only the best shopping experience.
Our whole team at Tugrow1688. is committed to serve your needs and be there with you all throughout your shopping experience. We are dedicated to constantly improve our services in offering you only the best possible options because we love making you happy. This Chinese-owned but Nigeria-based company is committed to procuring goods from china and shipping them down to Nigerian clients. Located in Ikeja, this Chinese logistics company offers services ranging from shopping in china, payments of suppliers, order tracking, and shipping from China.We offer highly professional service rooted in our core values: Competence, Convenience, and Reliability. We are results-oriented, so you can be assured we deliver quality service. Wherever you are, we want to offer you only the best importation experience.
51.Huawei
Founded in 1987, Huawei is a leading global provider of information and communications technology (ICT) infrastructure and smart devices. We have 207,000 employees and operate in over 170 countries and regions, serving more than three billion people around the world.With integrated solutions across four key domains – telecom networks, IT, smart devices, and cloud services – we are committed to bringing digital to every person, home and organisation for a fully connected, intelligent world.
Huawei Consumer BG is the leader of the all-scenario AI life. It covers smartphones, PC and tablets, wearables, mobile broadband devices, family devices and device cloud services. Huawei Consumer BG is dedicated to delivering the latest technologies to consumers and sharing the happiness of technological advances with more people around the world. Walk the walk and make dreams come true.
Huawei, a Chinese telecommunication company with a branch in Lagos, is one of Nigeria's providers of information and communication technology (ICT) infrastructure and smart devices. With the vision to bring digital to individuals of the world, Huawei has succeeded in providing superior computing power to the people of Nigeria.
Our vision and mission is to bring digital to every person, home and organization for a fully connected, intelligent world.
Research & Innovation
Scientific exploration and technological innovation drive civilization and society forward. Huawei understands the importance of research and innovation and how openness is critical for both. We are ready and willing to work with academia and industry to explore the frontiers of science and technology, push innovation forward, create value for industry and society as a whole, and build a better intelligent world.
Openness, Collaboration, and Shared Success

TECH4ALL's education programs have benefited over 600 schools and more than 220,000 people, including K – 12 students and teachers, unemployed young people, and senior citizens.we are committed to giving back and donating to the countries and regions in which we operate so that we grow hand in hand with local communities and drive local socioeconomic recovery.Worker

participation, consultation, and communication on occupational health and safety. Huawei works hard to create a culture of integrity and enhance its compliance capabilities.

52.Chai Tang Restaurant

As one of the best Chinese restaurants in Nigeria offering authentic Asian style fine dining, Chai Tang Restaurant is best known for its unique menu that has been put together to fit the Nigerian palette. Their unique environment and interior give it a plus to customers looking to explore Chinese Cuisine. Chai Tang Restaurant is situated Off Remi Olowude road Lekki second roundabout (by Lekki leisure/ark event centre).

53.Jereh Group

Innovation Driven Multi-industry Corporation

Yantai Jereh Oilfield Service Group Co., Ltd. was founded in 1999 and was transformed to a company limited by shares on 22 November 2007, with its headquarters located in Yantai City, Shandong Province. And in February 2010, Jereh (stock code 002353) was listed on the SME board in Shenzhen Stock Exchange.

Founded in 1999, Jereh Group is an innovation driven multi-industry corporation specializing in high-end equipment manufacturing, oil and gas engineering and technical services, environmental management, new energy, collaborative innovation of the industrial internet, code-less digital twin designer, massive health and elderly care, etc. We provide customers with products, services and integrated solutions with great competitiveness, safety and reliability.

Sincerity has been the cornerstone of Jereh’s culture since its founding. While we work to actively and continuously create value for customers, we aim to contribute honesty, harmony and respect to the society as well. Today, nearly 7,000 outstanding Jereh employees are working diligently in over 70 countries and regions. Together with our global partners, we are striving to meet people's expectations for a better life.

As a leading manufacturer of oil and gas field, Jereh provides customers with integrated solutions for the exploitation of oilfields and introduce cutting-edge products based on the development of unconventional energy, including more than 160 types of products: cementing equipment, fracturing equipment, coiled tubing, nitrogen generation and pumping equipment, gas turbine gensets, etc.

Honors

National Manufacturing Individual Champion

National Industrial Design Center

National Enterprise Technology Center

LexisNexis Innovation: The Global Top100

Deloitte Best Managed Companies

Top 50 China’s Most Innovative Enterprises for Out-going

Forbes China's Top Up-and-Comers Listed Company

Forbes China’s Sustainable Development Industrial Enterprises Top50

China's Top 10 Energy Equipment Enterprises

China's Most Influential Enterprise in Oil&Gas Industry

National Quality Benchmark Enterprise

Shandong Top 1 High-end Equipment Manufacturing Private Enterprise

Top 10 Industrial Clusters in Shandong

Industry Leading Entrepreneur in Shandong

Top100 Taxpayers of Shandong Province
China Charity Outstanding Contribution Award
Social responsibility
Jereh is dedicated to creating long-lasting value for local societies and our customers, and to be an enterprise respected by people all around the world. We provide our time, skills and gifts in kind for mutual benefit. We concentrate on stimulating local economic development and supporting educational opportunities in our areas of expertise. Below you will find some examples of what we do in local communities in our home markets.
Protection of the Ecological Environment
While pushing forward with the development of its various businesses, Jereh is also committed to protecting the natural environment and natural resources, and to ensuring sustainable development for future generations. Jereh has been disseminating basic knowledge of energy and environmental protection to the younger generation through innovative brand popularization activities, fulfilling its corporate social responsibilities with practical action.
Educational Support
Education is the cornerstone of cultivating innovative talents as well as an important foundation for the rapid and sustainable development of the industry. Jereh actively promotes the training and development of industrial talents, and works towards enhancing society's knowledge of oil & gas, new energy, environmental protection and other industries.
Energy Equipment Innovation Design Competition
The "Jereh Cup" China Postgraduate Energy Equipment Innovation Design Competition, founded in 2014, is specifically for postgraduate students and is one of the 11 themed competitions of the "China Postgraduate Innovation Practice Series". Jereh has sponsored the competition for 8 consecutive years, attracting a total of 170 universities and 1,261 works on themes of marine energy equipment, green and efficient oil & gas equipment, wind energy equipment, and solar energy equipment, etc.
Entrepreneurship Competition
The HKUST Entrepreneurship Competition originated in 2011 and the famous DJI drones and Yunzhou unmanned ships were its first competition projects. Jereh has exclusively sponsored the 2022 "HKUST - Jereh Group Entrepreneurship Competition". Focusing on artificial intelligence, the competition has attracted and selected high-quality artificial intelligence entrepreneurial projects from around the world! Through the cooperation, Jereh has comprehensively expanded in-depth exchanges and resource sharing with HKUST in various fields, such as production, education and research.
Social Relief
We actively participates in social welfare undertakings, and has initiated the establishment of Jereh Charity Fund of Yantai Charity Federation and Boji Relief Fund in Laishan, which is mainly used for the relief of individuals and families in distress, the incentive of righteous and courageous, the targeted relief for the elderly, children, students, disabled people and patients, etc., and social welfare projects such as natural disasters. Since 2010, Jereh has donated a total amount of more than $3 million, helped nearly 500 families and implemented more than 400 welfare projects.
Our Culture
Focus On Customer Needs

Jereh exists only to serve customers. To survive, we must create value for customers and satisfy their needs.

Prompt Response and Sincerity Remove the Worry

During a Spring Festival holiday, Jereh engineers got an American customer's urgent demand, they gave up their vacation and took the first flight to solve the problem. After 3 days and nights, they did it. The customer, who once was sceptical of the Chinese company, commended in the letter of appreciation "Jereh's engineers are as intelligent as the NASA engineers who put men on the moon".

Understand Customers' True Demands Through Onsite Investigation

Since 2021, Jereh has carried out "Quality Crusade" activity, leading by senior management to learn the voice of customers. They formulate improvement plans, and regularly report the progress to ensure the closed-loop management and continuous improvement of product and service quality. Up to now, Jereh has collected 607 suggestions from customers, with a response rate of 100%.

Value Employee Dedication

Only dedicated employees ensure the possibility of survival in the fierce competition. They are the backbone of Jereh. We value their contributions and reward them accordingly.

Never Let the Hardworking Man Down

We believe we can achieve best with full dedication. Great efforts bring more chances and rewards. Since 2008, "Golden Key" Car Award has been Jereh's most solemn commitment to motivate outstanding employees, covering 445 people with a total value of over $9.7 million.

The Highest Authorization of Incentives

At Jereh, the managers at all levels should pay special attention to dedicated employees and put forward incentives in time. When there is no ready-made regulations, inspiring dedicated employees is the highest authorization. Now, there are types of employee recognition and rewards, such as spot bonuses, instant incentive, Jereh Oasis defender, project award and some personalized rewards.

Continuously Improving To Be The Best

As change occurs, so should our ideas. Usually what defeats a company is an internal, rather than an external force. Numbness, complacency and isolation will only lead to failure. Self-criticism in an open manner is to keep Jereh more risk-conscious and innovative.

Suggestion for A Better Jereh

"Suggestion for A Better Jereh" has been our annually-organized event since 2003. Every colleague is welcomed to make suggestions to the company or department. And at the annual meeting, the founders will reward those whose advice is the most accurate and reasonable. We expect to form an honest and transparent environment where people are willing to share their true thoughts. Besides, there are many employees who would actively expose their "Faults", to remind others not to make similar mistakes .

Continuously Improving To Be The Best

Be fearless in every task, work hard and strive to be the industry's best.

Good, Better, Best. Never Let it Rest

At the beginning of the business, the first thing Mr. Sun Weijie did whenever he came back from exhibitions abroad was to share the products of the international companies. Though everyone felt that those high-end products were out of reach, Mr. Sun firmly believed that: We can make

the best oil equipment in the world! Nowadays, Jereh not only fills gaps of oilfield equipment at home and abroad, but also makes strategic layout in the fields of natural gas, environmental protection, municipal cleaning and new energy, etc.

Create an Oasis of Sincerity

Jereh is building a platform with sincerity, friendliness, purity, magnanimity, trust, openness, inclusiveness, equality, justice and enthusiasm. This will help people from all backgrounds work together and achieve positive results. Sincerity is fundamental to Jereh’s core values. We firmly believe that there will be more people to join us, and build a better future.

Our Vision

To be an enterprise respected by people all around the world.

Our Mission

Focus on customers’ challenges, provide competitive solutions and create long-term value for customers.

Our Spirit

Always look forward. Never give up. Nothing is impossible.

Our Purpose

Create value for our customers, create wealth for the society, provide opportunities for our staff, provide a return for our investors and secure growth for Jereh's future.

Our Goal

Build a worldwide brand. Make Jereh go from success to success.

Our Credo

We can work well only with dedication. There are always opportunities for those who work hard. No pains, no gains.

Service Goal

Focus on customer needs, exceed customers expectations.

Our Orientation

Be a responsible person. Shoulder the responsibility of citizenship, corporate, team and family.

54.SINOSURE

China Export & Credit Insurance Corporation (hereinafter referred to as "SINOSURE") is a state-funded and policy-oriented insurance company established and supported by the state to promote China's foreign economic and trade development and cooperation. As an independent legal person, SINOSURE was officially launched and put into operation on December 18, 2001, whose service network now covers the whole country.

By means of export credit insurance against non-payment risks for China's foreign trade and investment cooperation, SINOSURE promotes Chinese exports of goods, technologies and services, especially the exporting of high-tech and high-value-added capital products such as mechanical and electrical products. In this way, the company further supports economic growth, employment, and international balance of payments. SINOSURE's main products and services include: medium and long-term export credit insurance, overseas investment insurance, short-term export credit insurance, domestic trade credit insurance, bonds & guarantees and reinsurance related to export credit insurance, accounts receivable management, and information consultation services.

Taking "performing policy functions and serving open economy" as its own responsibility, SINOSURE effectively serves for national strategies, accurately supports the development of enterprises and ensures financial sustainability. The company actively expands its coverage of export credit insurance and plays an irreplaceable role in supporting the construction of the Belt and Road Initiative (BRI), promoting a steady and quality growth of foreign trade, cultivating new edges for international economic cooperation and competition, and accelerating the optimization of economic structure.

Focusing on the area of credit risk management, SINOSURE has established the Country Risk Research Center and the Credit Rating Center. Its credit information database covers more than 360 million global enterprises and banks. SINOSURE also has more than 400 information channels home and abroad, whose credit reporting and recovery business cover all countries and regions and major sectors across the globe. As of the end of 2020, SINOSURE had accumulatively supported more than USD 7.06 trillion of domestic and foreign trade and investment, provided credit insurance-related services for over 280,000 enterprises, paid a total of USD 19.38 billion claims and facilitated nearly 300 banks offering more than RMB 4 trillion of financing for exporters. According to the statistics of Berne Union, since ever 2015, the total insured amount of SINOSURE has been continuously ranking top among ECA members.

SINOSURE, a policy-oriented financial institution established to meet the demands of economic globalization and the development of China’s foreign economy and trade, will closely focus on the goal of serving the national strategies. Guided by the policy and centered on customers, SINOSURE will strive to build the company into a more responsible and reliable policy-oriented export credit insurance institution that enjoys global impacts, making greater and new contributions to China’s new round of high-level opening up.

Mission and responsibility

Supporting the Belt and Road Initiative

Since President Xi Jinping proposed the Belt and Road Initiative in 2013, SINOSURE has vigorously supported the development of the Initaive. By the end of 2022,the total insured amount of SINOSURE on export and investment to the The Belt and Road countries was over $1.3 trillion, covering over 3800 projects, mainly in such industrial sectors as power, transportation, petroleum equipment, housing construction, communications equipment, shipping, infrastructure and telecommunications, with claims paid amounting to over USD 4.3 billion.

Supporting “Made in China”

As a national policy-oriented insurance agency, SINOSURE has taken an active role in fullfilling the "Made in China 2025" Initiative, guiding enterprises to use national credit resources, carrying out scientific and technological innovation and technological upgrading, and striving to help "going out" enterprises become more competitive in the global market. SINOSURE has set up special underwriter teams on industries such as information technology, advanced rail transportation equipment, energy-saving and new energy automotive, in order to strengthen the risk study and propose tailor-maded underwritting policies for different industries.

In recent years, the world economy has been experiencing a period of deep adjustment. Faced with the continued downturn in the international demand and severe situation of foreign trade, SINOSURE has increased support for high-tech industry, mechanical and electrical products, textiles, light industry, automobile,etc., to help stablize the development of the nation's foreign trade.

Supporting Small Business

For a long time, SINOSURE has always taken supporting the export of small- and medium-sized enterprises as a priority work, not only for their important role in stabilizing social employment, but also because they are an important group of technological innovation and intelligent manufacturing. Since 2005, SINOSURE has launched two products for SMEs, “Comprehensive Credit Insurance for Small- and Medium-Sized Enterprises” and “Easy Credit Insurance for Small- and Micro-Sized Enterprises”.

In 2022, SINOSURE provided service to over 150,000 small- and medium-sized enterprises; supported USD 226.8 billion for the export of small- and medium-sized enterprises, a year-on-year growth of 15.7%.

Overseas office

Johannesburg Representative Office

Address: Unit 58, The Nicol, Hobart Road, Bryanston, Johannesburg, South Africa

55.China Jiangxi International Economic and Technical Cooperation

China Jiangxi International Economic and Technical Cooperation Co.,Ltd (CJIC), subordinated to State-Owned Assets Supervision and Administration Commission of Jiangxi Province(SASAC-Jiangxi), was established in 1983 with the approval of the State Council of the People's Republic of China. CJIC is an international conglomerate corporation focused on international contracting and domestic and foreign investment, it is also engaged in real estate development, domestic construction, architectural design, mineral exploitation, foreign labor service, international trade and China's foreign aid projects.

In more than 50 countries and regions in Asia, Africa, Oceania and Latin America, CJIC has delivered over 600 international contracting projects and China's foreign aid projects concerning buildings, roads and bridges, water conservancy, airports, stadiums and power supply infrastructure. The total contract value of CJIC's projects has reached 8 billion USD. In respect of foreign labour service, CJIC has sent about 100,000 skilled workers to Japan, Singapore, Jordan and Saipan (US).

CJIC has gained the following endorsements by virtue of its outstanding performance:

Vice-Chairman Member of China International Contractors Association (CHINCA);

Chairman Member of Chinese International Corporations' Working Committee of CHINCA;

Chairman Member of Jiangxi Enterprise Association of Outward Investment and Economic Cooperation;

Standing Vice-Chairman Member of Jiangxi International Chamber of Commerce;

No.67 in ENR's 2022 Top 250 International Contractors List

No.22 in 2020 Top 50 Chinese International Contractors List for Highest New Contract Value

No.25 in 2020 Top 50 Chinese International Contractors List for Highest Turnover

AAA Credit Rated International Contractor

AAA Credit Rated Foreign Labour Service Provider

AAA Credit Rated Chinese Construction Company

Core Values

Opening up, Pioneering,Innovating, Creating

Our Mission

Advance Construction Engineering
Bridge International Cooperation
Our Strategic Goals
Become a leading international contractor
Become a major international conglomerate
Become a key resource optimizer
International contracting
International contracting is the core business of CJIC, involving building construction, roads and bridges engineering, water conservancy engineering, Borehole drilling, water supply and sewage treatment, power supply and airport infrastructure. With over 130 projects in progress, the total contract value of CJIC's projects has reached 8 billion USD. CJIC has been delivering projects mainly by the models of construction general contracting, Design-Build, Engineering, Procurement and Construction(EPC), EPC+F and Turnkey contracting.
Corporate Philanthropy
CJIC has donated teaching and learning materials to Likasa Boys Boarding Secondary School in Lusaka Province, Zambia.
CJIC has sponsored a number of African students to pursue education in China
CJIC has funded and constructed water boreholes for the locals to secure clean and safe drinking water
CJIC's employees volunteered at Mother Teresa Children's Home (Kenya) and donated food and necessities to the children
56.HUAJIAN GROUP
Huajian Group was established in 1996 with its headquarter located in Dongguan of Guangdong Province, mainly specializes in the production of high and middle-grade women’s leather shoes. It owns 4 major production bases, namely Dongguan in Guangdong Province, Ganzhou in Jiangxi Province, Zhaotong in Yunnan Province and Ethiopia in Africa, as well as 7 shoes vamp factories and more than 10 subsidiaries such as Huajian International Light Industry City(Ethiopia)PLC, Dongguan Huarui World Footwear Headquarter Base,ect., and has developed into an integrated international enterprise including research and development, trade, production processing, leather and shoe material manufacturing, shoes machine supporting, logistics distribution, education and training, footwear headquarter base, industrial park, and so on. With more than 15000 employees and 40 modern shoe-making production lines, it has an annual output of more than 20 million. By virtue of a strong production capacity, advanced technology, and excellent quality and service, Huajian has won the favor of top 30 high and middle-grade women’s shoes brand of US. Many famous brands have established a stable cooperative relationship with Huajian.
Huajian has always been adhering to the enterprise principle of “People-oriented,Serve the people”,and the enterprise mission of “Exist for society,Strive for industry”,devoted to happy life and efficient work of all its employees, and has turned into one of the most dynamic modern enterprises in the footwear industry.
Huajian International Shoe City(Ethiopia) PLC was established in November 2011. It’s a wholly owned subsidiary of Huajian Group in Ethiopia, the latter is one of the world's largest women's shoes manufacturer. It’s located in Dukem town, the outskirts of the Ethiopia’s capital Addis Ababa, with 9 modern shoe-making production lines and shoes material factories and more than

6000 employees. It mainly produces shoes of GUESS and other world famous brands, with an annual output of more than 2.4 million. Now it has become an important women's shoes OEM manufacturing base in Africa of Huajian Group, known as "the most successful case of China's production capacity going overseas", has also become a model project of economic and trade cooperation between China and Ethiopia.

Introduction to China-Ethiopia Huajian Light Industrial Town

Invested and constructed by Huajian Group, Huajian International Light Industry City(Ethiopia)PLC is located in the Labu Lafto District of Addis Ababa, the capital of Ethiopia. It includes such functions as export processing, trade and service,etc., this integrated industry park focuses on light industry and takes science and technology, high efficiency, civilization, harmony and environmental-friendly as its theme. It is the pioneering and demonstration area of China’s advantageous industry going to Africa and of Guangdong Province’s African industrial park through “One Belt, One Road” strategy. The China-Ethiopia Huajian Light Industrial Town is an industrial park for manufacturers and exporters of light industrial products, such as clothing, footwear, headwear and electronics, located in Labu Lafto district in Addis Ababa, Ethiopia. The construction was started in April, 2015, and is expected to be completed by 2025. The construction of the factory area, covering 500,000 m2s, is expected to be finished by February, 2018.

The Huajian Industrial Town will become a dynamic, glamorous and environmentally-friendly community, a combination of both industrial and residential areas. It involves 7 sub-areas, including the comprehensive industrial area, the industrial park, apartment area, Forest Inn area, office building area, commercial area and shopping centre. When completed, the Industrial Town will become a new driver of the development of light industry in Ethiopia and Africa, generating over 2 billion dollars of revenues and 30 to 50 thousand jobs each year.

The construction of this project is in line with Chinese government’s “One Belt, One Road” strategy, as well as Huajian Group’s ambition to further expand and update its business internationally. It will become a great demonstration of the Group’s motto of serving more people and contributing to the industry and society, and a perfect example of Chinese company successfully settled in Africa.

58.Sinoma Overseas

Sinoma International Engineering Co., Ltd, Overseas Development Branch (Sinoma Overseas For Short) established in 1988, the branch of the state-owned publicly held company, Sinoma International Engineering Co., Ltd (Sinoma International For short, stock code: 600970). As the largest integrated service provider in cement industry in the world, Sinoma International belongs to Sinoma Group, who is directly under the management of State-owned Assets Supervision and Administration Commission (SASAC for short).

Three core businesses of Sinoma Overseas are project general contracting, overseas investment and production & operation management. Over 30 years’ development, Sinoma Overseas has spread its business to Southeast, Middle-east, Africa and South America and other districts all over the world. Sinoma Overseas contracted projects including cement, glass, refractory, power facilities, etc. under the EPC turn-key basis and provided production & operation services for a large number of industrial plants, which makes Sinoma Overseas a highly influential company in

international engineering market. Based on the company's advantages in technology, management, and capital, overseas investment business achieves the company's globalization strategy through professional teamwork, integration of local resources, EPC financing, equity investment, and BT, BOT, PPP, and other modes. The purpose is to actively contribute to the economic development and prosperity of the host country.

Under the guidance of China Building Materials Group's "Innovation Drive, Green Development, and International Cooperation" strategy, Sinoma Oveseas insisting on the development concept of erials Group's "Innovation Drive High coordination” and the ethics of “Satisfy needs of customers, Create values of the company”. Sinoma Overseas have been endeavoring in pursuit of healthy & sustainable development future to meet the ever-changing challenges in building material engineering industry over the world.

Main Business

Cement Project

Sinoma Overseas has established a number of cement production lines of different scales in EPC contract general contracting mode and EPC+ financing mode in Southeast Asia, the Middle East, and Africa. All projects have been completed and put into operation. The technical indicators of the production line have reached the international advanced level. It was well received by the owners. Under the national policy and the strong support of CITIC Insurance, Sinoma Overseas has actively helped foreign owners and domestic financial institutions to build bridges, promoted the successful landing of a series of financing projects, and better achieved the “walking” of Chinese technology, equipment and engineering services. Go out." At present, it has successfully operated and implemented two 5,000-ton export buyer’s credit projects in Turkey, and the financing of subsequent projects is in progress.

Glass Project

Sinoma Overseas specializes in engineering design, complete sets of technologies and equipment supply for various glass factories, general contracting of glass projects, technological transformation of glass factories, technological development, technical consultation, technical services, and research and development of glass production technologies, materials and equipment, manufacturing and other services.

Power Engineering

EICO (Energy and Infrastructure Co., Ltd.) in Jeddah City in Saudi is operating the power engineering business. The scope of work covers power facilities construction, power equipment supply, EPC for power distribution stations, substations, hydropower stations, thermal power stations and wind power generation. EICO commenced operations in September 2012, has successfully obtained SEC (Saudi Electric Company) Al-Kharj-2 380kV substation civil engineering sub-project. EICO is gradually speeding up the electricity market development both in local and in the Middle East.

Sinoma Overseas has been operating in the power industry for many years and has extensive experience. Its business scope covers general contracting of power facilities, equipment supply, distribution stations, substations, hydropower stations, thermal power stations, and wind power generation.

Other Areas

In addition to the general contracting business in the cement, glass, and electric power fields, Sinoma Overseas has also diversified its operations and can undertake construction projects

including housing construction, road construction, bridge construction, power transmission and transformation, non-metallic grinding plants, and metal and non-metal processing. Various industrial and municipal construction projects such as factories, aerated concrete block plants and municipal waste incineration projects are currently underway in Oman, Myanmar and Vietnam.

Enterprise Culture

1.Vision

Leading in China Top-class in the World

2.Development Philosophy

Sustainable and Fast-paced Development

3.Operation Philosophy

Create Corporate Value by Decoding Customer Demand

59. CGCOC Group

CGCOC is a mixed-ownership enterprise jointly funded by state-owned enterprises, management teams, local state-owned assets, overseas key employees and external natural persons. By adhering the business philosophy “creating wealth through cooperation, seeking development through innovation", CGCOC shoulders the mission of “going out” and insists on focusing on overseas markets and long term rooted localized management. By having distinctive characteristics in its own system, mechanism and development model, CGCOC brings lucrative returns for shareholders and makes great contributions to the development of those countries and the implement of the China’s strategy of “going out”, thus receiving the praise from national leaders, foreign heads of state and people from all walks of life, becoming a new era Export-oriented enterprises with unique characteristics in China's external economic.

Since its establishment, the business field of CGCOC has developed from the single project construction into four business areas, which include consulting planning, investment operation, overseas infrastructure construction, logistics and trade. The employees of CGCOC have reached to nearly 25,000 at home and abroad, among them, over 90% are foreign nationals.

CGCOC has formed three regional centers in Africa, namely the eastern Africa, west-central Africa with English speaking regions and French speaking regions, covering 27 countries and accounting for 50% of the whole African countries, among which, CGCOC Nigeria Company has been established for 35 years. Now, the brand influence of CGCOC taking team works as its base and core is becoming increasingly remarkable.

By relying on the long-term overseas backbone team strength, the markets of host countries, CGCOC takes the "high-end consulting, comprehensive planning" as the means, providing the comprehensive development of host counties with synthesized solution which is accordant to Chinese overseas interests as well as the needs of host countries.

Following the important instruction President Xi Jinping and Premier Li have given to CGCOC during their visit to Africa, CGCOC is now working hard to build service platforms, namely "a partnership for Africa's development" and "a bridge for China-Africa cooperation".

CGC Nigeria Limited (China Geo-engineering Company), a Chinese company with its headquarter in Abuja is a subsidiary of CGCOC GROUP CO., LTD. in mainland China. Our company CGC Nigeria Limited is a reputable Engineering Management and construction company at the forefront of construction of roads, bridges and other civil infrastructures in Nigeria.

The predecessor of CGCOC Group was founded by the Ministry of Geology & Mineral Resources (zh) in 1983 as CGC Nigeria Ltd.. In 2002 other investors were introduced in the incorporation of CGC Overseas Construction Group Co., Ltd., which CGC Nigeria became part of the business group

Culture

We are committed to an excellent corporate culture, reflecting culture reflecting the modern enterprise development features. In the past few decades, we have formed a distinct corporate personality. Our employees come from over 20 countries around the world. The diverse cultural traits they represent are well integrated and respected in our company.

Cooperation Breeds Wealth

Based on brands and markets,

we proactively cooperate with all kinds of organizations, merging and synchronizing various resources.

We collaborate on the principle of equality, mutual benefits, sincere cooperation and common development.

Innovation Empowers Development

Innovation is the soul of a nation’s progress; innovation is also the inexhaustible source for a corporation to survive and thrive.

Over the years, we are committed to an overseas operation platform for China’s capital and technology.

We seek constant improvement; adjust strategies to adapt to the new demands, harnessing innovation for the sustainable development of our enterprise.

Visionary and down-to-earth

We are a team with dreams. We aspire to become a world-class enterprise. We wish for our country to become a top global country. Dreams are the constant source of our enterprising spirit. But we are also acutely aware that focusing on the present is the way to achieve our dreams. While looking forward to a distant future, we also need today's hard work. The only way to realize our dreams is diligence.

We want to be a cross-border investment holding group, able to get adapted to international conventions, converge converging international resources and harness the global value transfer.

Our market is mainly overseas. Since our inception, we have keenly followed the rules of the target markets. We not only reference our culture as a standard, but also fully accept the localized culture. Because we focus on oversea markets, we also place our resource targets overseas. All legitimate resources, if beneficial for business development, (capital resources, technical resources, human resources, or intellectual resources) are potential intakes for our company. We always strategize corporate development against the bigger picture of international economic orders. We closely follow changes in the international economic order and value-orientation. Our goal is to be able to stage and strengthen our foothold in different valued regions and to achieve high-level strategic decisions and operational objectives in the international commercial order.

A new-Chinese-styled multinational

We consciously strive for the national mission – to realize the great Chinese dream. With our roots in China, our vision casts to the world, far and wide.

CGCOC is a multinational corporation, focusing on the overseas operations. The English abbreviation of the company ‘CGCOC' bears special meanings as follows: the company logo uses two colors, namely orange and blue, with orange-yellow as the theme color. In the upper left cornerright corner, the blue color represents ‘the sky of dreams’. In the lower right left corner, the orange color represents "the harvest of the earth." The yellow letter C stands for yellow-skinned Chinese people, but also the initial letter of the word ‘China’. The sky and the earth blend harmoniously are respectively situated in the diagonal corners, indicating that our hard work will bring forth continuous harvest,we are open-minded, professional and devoted to the broad overseas market, and ultimately realize the dream with our down- to-earth action and determined spirit.

60. Foton Motor Group

Founded on August 28th, 1996, Foton Motor is headquartered in Beijing, China. With a business scope covering a full series of commercial vehicles including medium and heavy-duty trucks, light-duty truck, mini truck, vans, pickup, bus, and construction machinery vehicles, Foton Motor has become a leading commercial vehicle manufacturer in the world. Foton Motor's new energy technology has leading the world, especially in electricity and hydrogen fields.

Offering high value-added products and services to local users, and implementing globally leading automobile manufacturing and operation standards to other regions with its business.

Making a fast development in commercial vehicle business.

MISSION & VISSION

Since its foundation, Foton Motor has been focusing on building a future full of harmony of human, auto and nature.

EMBLEM

Image of a diamond has been cited as the logo prototype of FOTON, it signifies technology, quality, high value and permanence. Foton "Brilliant Diamond" is likened to a sparking diamond, which implies Foton's commitment to technological innovation, human care and the beauty of harmony.

VISION

FOTON will lead the way to the future of mobility, sustainably create perfect and outstanding eternal values for the welfare of customers, society and humankind.

MISSION

We FOTONER are always aiming to challenge high goals, seizing sustainable developing opportunities, raising our standard for dependability, reliability and customer satisfaction, driving a modern life through a commitment to integrated technology.

TECHNOLOGY LEADING INTO THE FUTURE

Driven by technology, we are vigorously developing new energy technologies and moving towards fossil fuel-free transportation.

MILESTONES

Leading the way to a global commercial vehicle manufacturer.

Innovations

Leading forward to the commercial vehicle mobility and intelligence.

Foton has made constant breakthroughs in automatic driving field from 2016. In 2016, it took the lead in releasing unmanned trucks in China. In 2018, Foton developed into the first enterprise that got automatic driving road test license plate in commercial vehicle sector of China. Till now,

it has put L3 automatic driving into use. Foton enabled the intelligent sharing of safe, comfort, energy-saving and efficient driving between vehicles and people, other vehicles & roads.

R & D CAPABILITIES

Advanced Testing Capabilities

FOTON has the industry's advanced virtual simulation testing capabilities for crash safety, aerodynamics, NVH simulation and optimization, which can effectively improve vehicle comfort, safety and reliability.

World-class Testing and Verification Capabilities

FOTON has built a domestic high-standard experimental center, and has the testing and experimental capabilities of complete vehicles and key components. It will further develop into an automobile enterprise with high-level testing and verification capabilities.

High Standard Digital Factory

In accordance with the concept of "zero emission, no contact and automation", through upgrading and introducing automation, digitalization and intelligent technology: the world's first flexible production line for 8 models, “6-Axis and Horizon Robotics” Technology, 40% reduction in welding energy consumption, 100% automation rate of mainline design, 100% auto paint spraying automation rate.

Sustainability

NEW ENERGY PRODUCTS

Under the dual trends of international "carbon reduction" and environmental protection, FOTON IBLUE has been at the forefront of China's new energy commercial vehicles, independently developed the core technologies of Battery, electric motor and Electric Control Units (ECU). New energy products cover medium and heavy trucks, light trucks, VANs, mini trucks and passenger vehicles, covering multi-route development of pure electric, hybrid, and hydrogen fuel.

NEW ENERGY TECHNOLOGY

FOTON has global leading hydrogen fuel technology, and we are the first enterprise in China to realize the commercial operation of the entire industrial chain of hydrogen energy production, hydrogen storage, hydrogen transportation, and hydrogen refueling. During the Beijing 2022 Winter Olympics, more than 1,300Foton buses escorted the Winter Olympics 2022, including 500+ hydrogen fuel buses which became the largest hydrogen bus fleet serving for Olympics. It makes a record of the largest hydrogen bus fleet serving for Olympics.

ZERO EMISSION

In accordance with “zero emission,contactless and automatic” concept, FOTON upgrades and introduces automation, digitization and intelligent technology to build world-class modern factory and automatic production line by investing more than USD 2.3 billion within 4 years. FOTON produces intelligent products in harmonious and symbiosis with environment.

61.Chifeng Gold

Chifeng Jilong Gold Mining Co., Ltd. (Chifeng Gold) is a fast-growing international gold producer, mainly engaged in the mining ,metallurgical processing, and sales of gold globally.

The company owns 7 operating mines and 1 comprehensive resource recycling company in China, Southeast Asia and West Africa, including Jilong Mining - Zhuanshanzi Gold Mine, Wulong Mining - Wulong Gold, Huatai Mining - Honghuagou Gold Mine, Jintai Mining - Xidengping Gold Mine, Hanfeng Mining - Tianbaoshan Zinc-Lead-Copper-Molybdenum Polymetallic Mine in China, Sepon Au-Cu Mine in Laos, Golden Star Wassa Gold Mine in Ghana. The comprehensive resource

recycling project is a disassembly business of waste electrical and electronic products conducted by Guangyuan Technology in Hefei, Anhui Province, China.
Adhering to the core value of "to benefit more people through the development of Chifeng Gold", we attach great importance to the environment, safety, humanistic care and social responsibility. We always adhere to high-quality sustainable development with high ESG standards and are committed to following the highest standards and best practices in the industry.
As a growing publicly listed gold company, Chifeng Gold is committed to "being a dedicated and professional gold mining company". Through continuous innovation of management mechanisms, maintaining cost competitiveness, increasing the acquisition and consolidation of gold resources, utilizing resources effectively, proper development, adhering to internationalization, we strive to become a globally welcomed major gold producer.
62.China Henan International Cooperation Group CHICO
China Henan International Cooperation Group Co., Ltd. (CHICO) is a large-scale parastatal comprehensive foreign economic and trade enterprise under the jurisdiction of Henan Province, PRC. CHICO’’ s main business operation covers contracting international engineering projects, consulting service for international projects, international human resource cooperation and exchange, mining development, agriculture investment, undertaking infrastructural projects aided financially by Chinese Government in oversea countries apart from the involvement in information industry. CHICO maintains and operates regional offices in over 20 countries and regions across Asia, Africa and Europe to service its vibrant business.

The Company was accredited with ISO 9001:2000 Quality System Certificate, ISO14001 Environmental Management System Certificate and OHSAS18001 Occupational Health and Safety Management System Certificate from 2001 and has been listing in “Top 225 International Contractors” consecutively for many years. The Company was also awarded the title of “Advanced Enterprise of Henan Province in Foreign Economic Cooperation”, “National Advanced Enterprise of Engineering Construction Quality Management”, “AAA Credit Level Enterprises of International Project Contracting and Human Resource Cooperation”, “New Century International Technology and Quality Gold Medal” etc. domestically and internationally.

Since establishment in 1983, CHICO has been adhering to the principle of “Honesty and Faithfulness, Quality First, Mutual Benefit and Common Progress” under which the Company has successfully completed more than 400 international engineering projects in such sectors as industrial and civil buildings, roads and bridges, power construction, and urban infrastructure development, which spans over 30 countries and regions in Asia and Africa; accomplished exploration of alumina, iron, copper and uranium in Guinea, Liberia and other countries, professionals provided to employers in more than 20 countries and regions, including Japan, Canada, Singapore in excess of 50,000 people qualified in different fields; the international trade business of the Company covered more than 30 countries and regions; the business volume and capacity of the automobile dealership and information service sector also rank high in the industries in China.
63.Mutual Commitment Company Ltd
Mutual Commitment Group (MCG)is a technical and execution arm in providing comprehensive services as an EPC and O&M service provider in Turnkey bases in the sectors of Energy Civil

Engineering，Agriculture, Water, Medical & Health Care Transportation，Aviation, Mining， ICT，Machinery and Complete Plant Supplies etc. And we are supported by the technical partners that are leading companies in those relative economic sectors. Our headquarter locates in Beijing, China and our services cover Asia.Africa, Middle East, America and Europe.

MCG develops like an Aircraft Carrier Base by organizing the member companies and different business areas complementary to each others. And also we develop the global market in a complementary way that we will not just look at a market independently or single lined, we bind our development closely to countries' development and Nations' mutual-benefits and try all our best to play a sustainable role in it. We tie up our growing closely on this mutual value adding way of development as our belief and commitment.

Right now, we are implementing our Support Nation's Development Plan (SNDP).SNDP comprises building the strongest one stop solution provide team for the infrastructure development to serve the developing nations and the developing or upgrading areas of developed regions for avoiding failure in the economic foundation;developing industry parks to transform the potential of the resources into development and welfare in the areas of agriculture, medical, oil& gas and mining; developing real estate in different regions for accommodating the people and efficient & economic logistics including marine; airline and marine services to reduce the cost for cargo transportation. We've started implementing this plan and will make out a model of development for supporting the developing nations' development and welfare of the people. We hope our plan will come true and we grow very well in this process MCG will be built up to be a platform that has Hundreds Billion Dollars capacity in serving the Nations and the system for sustainable operation and grow of the platform for long term. With our united, diligent and intelligent team that deals with concrete matters relating to work, we will make it and we will never give up,since our future is not just the beautiful future and dream of ourselves, but for all that are related.

Corporate culture

Life Is A Journey.

You Came With Nothing And Will Go With Nothing

But Can Leave Something.

Support The Nations' Development

Building A Platform For Changing Life Of The Staffs

Work Hard For The Better Life Of The Families

Contribute Good Lives To The People

Transfer Warm And Love To The One's In Need

CORPORATE SOCIAL RESPONSIBILITY

Mutual Commitment Group(MCG) always believe that while developing projects and being liable to the shareholders, we also need to dedicate to employees,clients, communities and the social environment of responsibility. “ Return to the Community,Public Feedback to Become a Good Corporate Citizen." is the goal that MCG always pursue and practice"

64.DEWE SECURITY SERVICE GROUP

DEWE SECURITY SERVICE GROUP（Abb. DWSS）is a leading and professional overseas security company in China that has successful management experience providing integrated overseas security services for Chinese-funded enterprises and institutions.

DWSS has established localized professional security service companies in China mainland, as well as in Kenya, Hong Kong and other countries and regions. The subsidiary Beijing DeWe

Security Service Co., Ltd. is a member of the China Security Association; an executive member and a standing committee member of the Overseas Security Service Professional Committee of the Beijing Security Association; Received the credit rating of AAA enterprise of China Export & Credit Insurance Corporation; In March 2016, Phoenix International Think Tank and Tsinghua University released the first comprehensive ranking of security companies. Beijing DeWe Security Services Co., Ltd. was ranked among the top three in China's security companies, and it has had a huge impact inside and outside the industry.

DWSS provides security services to overseas Chinese-funded enterprises and Chinese by means of “Integrated Security Service Solutions” featured by four core contents of advisory, training, overseas site security management and technology guarantee, with the core values of “patriotism, loyalty, dignity and devotion”. Performances and achievements of DWSS has been accepted and praised by Chinese government departments, overseas institutions and overseas Chinese enterprises and Chinese.

Our Clients mainly include the Ministry of Foreign Affairs, the Ministry of Commerce, the Ministry of Education, the Confucius Institute Headquarters and other government agencies; Sinopec, China Poly Group Corporation, China Communications Construction Company, China State Construction Engineering Corporation, Industrial and Commercial Bank, China Development Bank, etc. more than 50 large state-owned enterprises, and a large number of overseas Chinese business enterprises.

Up to the end of June. 2018, DWSS has provided high-quality and multi-field security services for over 200 overseas institutions, overseas Chinese enterprises and projects in 50 countries; Protected 20 billion $ assets and 70 thousand overseas Chinese; Dispatched More than 700 professional security personnel to overseas project sites with over 110 thousand working days; Organized More than3 thousand emergency drills on overseas sites; Carried out 3 thousand trainings on overseas sites with 70 thousand person-times; Found and proposed more than 4 thousand hidden dangers for safe rectification; Provided More than 500 case clues; Successfully handled more than 1 thousand various cases; Assisted in arresting more than 1,600 criminal suspects; Received more than 300 pieces of pennants, written praises, and thank -you letters from all units; Organized and implemented more than 1,600 period and nearly 170 thousand hours of pre-departure security training for more than 100 Chinese-funded enterprises, and trained more than 110 thousand person-times.

In the extreme events of coups and civil wars in some African countries since 2013, DeWe security officers assisted Chinese embassies and Chinese-funded enterprises in making correct decisions, properly disposing, and orderly evacuation. The relevant client units not only sent a thank-you letter, the main leaders also made a special trip to DeWe for their appreciation;In July 2015, DeWe successfully completed the security mission for the bidding delegation, the central and Beijing's main leaders in Kuala Lumpur during the bidding to host the 2022 Winter Olympics and was commended by the Central Government and Beijing Municipality.

With advanced concepts, professional elite team and strong comprehensive strength, DWSS is

playing an important role in safeguarding national economic interests and protecting the lives and property of Chinese citizens abroad. We will unremittingly strive to realize the "Chinese Dream" of the great rejuvenation of the Chinese nation!

65.Huaxin Zhong An Security Group
HXZA is the leading provider of security and risk management solutions in complex environments around the world. Our privately-held firm with over 21,000 employees provides specialized customer-centric services to protect client assets and personnel by implementing bespoke threat mitigation strategies which simultaneously simplify operations and harden security posture, all while reducing costs and building a responsible brand.

The company's extensive experiences underpin our capacity to coordinate and deploy tailored, multidisciplinary teams and technology globally. Our management works closely with our clients to develop individualized security and commercial programs intended to achieve all mission objectives by mitigating risk, ensuring compliance, enhancing quality, and meeting budgets.

Our operations are certified in compliance with relevant ISO standards and the International Code of Conduct (ICoC) for Private Security Providers. In fact, HXZA is the first Asian company to receive certification from the ICoC Association and is presently the only maritime security company to hold such certification.

The company's core management team is primarily composed of college-educated ex-servicemen and ex-police officers, who are dedicated to the company vision: Security through harmony. Although we hold substantial market share throughout mainland China, our overseas operations are rapidly expanding-- now supporing clients across four continents and more than 40 countries.

HXZA proudly serves clients across a range of industries including manufacturing, maritime, aviation, banking, retail, hospitality, government, academia, petrochemical, and extractives.
Social Responsibility
HXZA constantly strives to enhance the quality of our services, promote a responsible brand, and be a genuine force for good-- creating low-profile, but positive impacts in the sensitive regions where we operate.

Our company recognizes the constructive role businesses like ours can play in advancing a socially responsible agenda. We place significant importance on the promotion human rights and protection of the environment throughout our worldwide operations. This is reflected in our business philosophy: Security through harmony.

Our conceptualization of social responsibility is derived from three main international frameworks. First is the UN Guiding Principles on Business and Human Rights, which all business should comply. Second is the Voluntary Principles, which many of our clients must comply and demand our adherence. Third, and most specific to our work, is the International Code of Conduct for Private Security Service Providers.

To live what we preach, we also pursue socially responsible behaviors at home. We have established a labor union to represent our more than twenty-thousand employees, developed a suitable grievance mechanism to process complaints, adopted environmentally sound practices, and engage in several charitable projects aimed at the serving the underprivileged.

66.China Security Technology Group

China Security Technology Group Co., Ltd. (CSTG) is an integrated security solution provider skilled in using advanced technology approach and new decision-making ability to ensure professionalism and sustainability of security business. As the wholly-owned subsidiary of Beijing Huatai Zongheng Investment Management, CSTG was incorporated in Hongkong in June 2016. It mainly provides security service and support for the major China-invested projects, employees and assets in the countries along “Belt and Road”, ranging joint personnel, physical and technological protection at fixed or mobile places, VIP guarding, land or maritime transportation escort, risk assessment and consultation, safety training, public security technological precaution, and equipment rent and sale, etc. CSTG is committed to build its internationalized security strength with legal compliance through establishing cooperation and mutual trust relationship with host countries and integrating with the international security system and convention.

CSTG is possessing an elite group composed of advanced consulting organ, expertise and senior executives. Its new cooperative mode of bundling its core capability with localized supplier into strategic combination has stand the test. Furthermore, by signing cooperative agreement with some well-known domestic security product manufacturers for global promotion, it is committed to achieve mutual benefit and win-win between countries. By far, CSTG has set up several localized branches or subsidiaries in Pakistan, Sri Lanka, Kampuchea, Kenya, Algeria, Iraq and Angola, and the total number all round the world will be more than 30 by 2020, meeting the growing security demand for international market.

In line with “people oriented, safety first, prevention prioritized, and service foremost” principle and based on advanced technology and modern management concepts, CSTG will keep pace to deliver the customer with distinctive service and applicable solution featured by professionalism, localization, precision and customization, boosting the harmonious and win-win development of the countries along “Belt and Road”.

Developing Goal

- to become china’s largest overseas security service provider in three years
- to become Asia’s largest overseas security service provider in five years
- to strive to enter the industry's global top five in eight years

OUR CONCEPT AND CULTURE

Our Mission: We commit ourselves to the continuous provision of internationalized, standardized, and China-characterized overall, comprehensive and professional security protection service for China’s overseas investment interests in the countries along Belt and Road.

Our Strategic Orientation: Leading pioneer of China’s security protection service reshaping; Strong guardian the China-funded customers’ overseas interests; Dynamical participant of international security market competition

Our Service Tenet: Legal compliance, cooperation and win-win, service creates value

Our Principles: People oriented, safety first, prevention prioritized, service foremost.

Our Core Values: solidarity, persistence, vision, decisiveness
Our Entrepreneurship: Responsibility, dedication, integrity
Business Philosophy: Corporatized running, localized operation
Enterprise Culture: to pursue development through global market, to create brand through excellent performance, to gain customers through quality and reputation, and to obtain win-win through strategic cooperation.
Enterprise Features: localization, precision and customization featured by high-standard, high tech, professionalism and stability
Developing Strategy:Based on the "all-round security" sector centered by security protection business, CSTG is committed to build a new model of international cooperation characterized by technology leading, cultural integration, protection foundation, and industry combination. It is clustering the mobile Internet technology, intelligent protection technology, IOT situation awareness technology and advanced concept and service mode into a fully-integrated system that will cover operation service (personnel security, armed escort, safety training, etc.), equipment development and manufacturing, security assessment and consultation, and information release and warning. CSTG will always strive for the construction, integration and upgradation of trinitarian "all-round security" industry chain so that the customers' business, information and personnel are all protected safe and sound.
67.CIG Motors Company Limited
Guangzhou Automobile Group Co., Ltd. (abbreviated as GAC group, 6601238.SH， 02238.HK)， headquartered in Zhujiang New Town, is a large joint-stock automobile enterprise listed in both Hongkong and Shanghai Stock exchanges. At present, it has 119,000 employees. Fortunate Global 500 ranked No.165. The main business covers seven major sectors: research and development, vehicle, parts, energy and ecology, internationalization, commerce and transportation, investment and finance. Sticking to independent innovation and joint venture cooperation, GAC group is now transforming itself with all its strength to a technology-based enterprise. Adhering to the enterprise concept of 'Humanity', Credibility, Creativity', GAC is committed to building a world-class company which wins customers' trust, ensures staff's well-being,and meets social expectation.
We are committed to building a world-class company which wins customers' trust, ensures staff's well-being, meets social expectation.
Group Brand
What is the soul of an enterprise?

It is the unswerving persistence of creating high quality products and the ambition held in the business world.
GAC has long engraved "Detailing • Greatness" deeply in its brand genes.
GAC has a subtle insight into everyone's needs, adheres to the craftsmanship in every technological process, immerges in intensive study with a pragmatic attitude and explores every opportunity for innovation with an open mind.
All of these are for every one of you.
Creativity Defines Our Future.
GAC Philosophy is the general term for the core of GAC Culture. It shows the value and pursuit of GAC Group and the basic concepts and principles established to achieve its goals.

Taking GAC philosophy as the core, GAC Group will build an inspiring corporate culture with GAC characteristics to unite the value and strength of each employee to form a “business community”, so as to move toward the goal and vision of world-class enterprise and sustainable business.

Corporate Vision

We are committed to building a world-class company which wins customers’ trust, ensures staff's well-being, meets social expectation, and keeps creating value for a better mobile life of human beings.

Corporate Values

Humanity

Humanity: We put staff, customers, shareholders, cooperation partners and the public first. We care for staff’s development, devote to meeting customers’ needs, bring reasonable returns to shareholders, share profits with partners, and benefit the public.

Credibility

With integrity, we gain recognition from customers and all walks of life. With trust, we strengthen seamless cooperation among the staff, the company and partners for common good and development.

Creativity

Creativity: Taking innovation as the primary driving force for development, we are committed to continuous improvement and breakthrough. Encouraging creativity, we are devoted to creating products for customers, building platforms for the staff, earning wealth for shareholders and bringing value to the society.

Respect Humanity and Advocate Communication

We respect individual differences and respect different voices. We advocate perspective-taking and treat everyone equally. We value mutual communication and improve its mechanism to ensure timely and effective feedback.

Sincere Cooperation and Open Sharing

We conduct internal and external cooperation on the basis of integrity and commitment.We value inclusiveness and openness, share resources and benefits to achieve win-win situation and complement each other’s advantages.

Innovation Driven and Pragmatic Efficiency

Innovation is the driving force of development – we dare to challenge and explore.Practice makes perfect – we keep learning to maintain flexibility and improve efficiency.

Theme Activity

According to the corporate culture construction plan and in combination with the corporate culture and philosophy of GAC Group, three theme activities of "Happy GAC", "Responsible GAC" and "Innovate GAC" have been carried out by integrating the existing activity resources of GAC Group.

68.Zhejiang Dinson Holding Co., Ltd.

Zhejiang Dinson Holding Co., Ltd. is an enterprise integrating manufacturing, sales and service which specialized in Chrome Ore, High Carbon Ferrochrome, Stainless Steel Wire Rod, Stainless Steel Coils, Bars and Billets. With headquarter located in Wenzhou, Dinson owns very superior location that 6km east to Longwan International Airport, 7km west to Longwan Dock and 16km far from Wenzhou station.

On the basis of chromium resource superiority, Dinson has developed the stainless steel industrial chain beginning from the raw material R&D to Stainless Steel products and domestic and foreign trade. Its subsidiaries includes Afrochine Smelting (Pvt) Ltd, Zhejiang Stellar Global Co., Ltd. and Wenzhou Dinson Metal & Minerals Co., Ltd.

Dinson owns 5000 hectares large chromium claims in Zimbabwe. Since the operation construction started in 2012, Dinson people overcome all kinds of difficulties and build the modernization of beacon ferrochrome smelters from the wild shrub with boldness and hard working spirit. The total investment on the first phase in the region of USD 35 million. Afrochine Smelting has owned 3x 16.5 MVA smelters with a capacity to produce 100,000 metric tons of ferrochrome per year with a sintering plant. The annual output value of Afrochine Smelting (Pvt) Ltd is 100 millions.

Dinson always adhere to the "Quality First, Credit standing paramountcy" business philosophy. The products have been widely applied in industries like petroleum, chemistry, metallurgy, shipbuilding, machinery, power, automobile, aviation, food and pharmacy, etc. The marketing network is now all over the world, products are mainly sold to North America, South America, Europe, Africa, Southeast Asia, the Middle East, India, South Korea and other countries and regions.

Zhejiang Dinson Holding Co., Ltd in the operation of human-oriented management concept, recruiting more capable personnel to build a enterprising, creative team to provide first-class products and service for global clients. Dinson people will, as always, cling together and strive for the bright future with practical approach and huge enthusiasm.

69.Tsingshan Holdings

Tsingshan is focusing on stainless steel production while exploring in the new energy sector. Aiming to make a change and to improve the living quality through stainless steel, Tsingshan takes ideas into action for the green and sustainable future of mankind. Tsingshan Holding Group Company Limited was registered in June 2003.

Vision：To build a world-class and respectable enterprise

Business Scope

Manufacturing, sales, warehousing, investment, import and export trade etc.

Main products: stainless steel ingot, bar, rod, plate, wire, pipe and other products, which are widely used in petroleum, chemical industry, machinery, electric power, automobile, shipbuilding, food, pharmaceutical, decoration and other fields. At the same time, we produce raw materials, intermediate products and new energy batteries, which are mainly used in energy storage systems and electric vehicles.

Mission

“Making life-long stainless steel and creating a sustainable future”, Tsingshan is committed to producing the high-quality, low-cost, energy-saving and environmental-friendly stainless steel in the efforts for building a safer and more convenient living environment.

Philosophy

FOCUS • OPENNESS • INNOVATION • COOPERATION

Focused on its industrial chain, Tsingshan communicates with people from all walks of life about industry development in an open attitude; and it is committed to production, technology, business running, and management innovation. Tsingshan puts people first and values mutually beneficiary cooperation and is willing to join hands with like-minded friends in the world to make

our dreams come true, and thus contributing to the society!
Corporate Culture
"Brave to be the first, honest and dedicated". Tsingshan has advocated "positivity" as the core of its business ethics and developed a unique corporate culture. The connotation includes: the consciousness of being brave to take risks and to lead the world; the values of developing enterprises and contributing to the society; the spirit of making stainless steel business stronger, bigger and better; the vision of working at the present and preparing for the future; team work spirit with combat effectiveness; the strategic thought that company's success depends on talents; a simple and harmonious interpersonal relationship; a clean and honest team image; a broad mind for win-win cooperation; and a vigorous and resolute style stressing speed and efficiency, as well as the value of developing enterprises and contributing to society.
CORPORATE STATUS
In 2023, we ranked 257th in Fortune 500. Over the years, we have achieved honors and are continuously being included in Fortune 500,Top 500 Chinese Enterprises, Top 500 Chinese Private Enterprises, Top 500 Chinese Manufacturing Enterprises and awarded many other honorary titles.Rank 257st in FORTUNE GLOBAL 500. 14th in TOP 500 CHINESE ENTERPRISES. 10th in
TOP 500 CHINESE MANUFACTURING ENTERPRISES.

70.China Zhonghao Nigeria Limited.
Zhonghao Overseas Construction Engineering Co.,Ltd.(ZOCEC) was registered as a private shareholding enterprise with capital RMB 200 Million in Beijing in 0ctober, 2001. As a supervisory member of Beijing International Economic and Technical Cooperation Association and China International Contractors Association, ZOCEC is authorized overseas engineering contracting enterprise by Ministry of Commerce of the People's Republic of China and certified by Quality Standard ISO9001.
Over the last decade, rapid development has been realized by the team of 300 staffs under the management Board Chairman Tian Chao thanks his vast experience and career as a senior leader with a state-owned international contractor, increasing overseas assets to 1500 medium and large scale construction equipments. ZOCEC is renowned as a major international project contractor home and abroad,has completed major projects of Road and Bridge Construction; Municipal Works & Building Engineering Construction and Decoration; Ge-engineering Investigation and Evaluation Boreholes and Water Supply; Water Treatment and Sewage Treatment Plant Construction; Mineral Exploration and Exploitation: Manufacturing and related Import and Export; Overseas Real Estate Development located across Asian and African nations: established branch offices in Nigeria. Ghana,Mali, Uganda, Kenya, Tanzania, South Sudan, Algeria, Zimbabwe, Cambodia making great contributions to the local economy through employing 2700 employees.
Focusing on the vision of "Becoming a Centennial Corporation of China", ZOCEC shall continue to grow via stepping up competitiveness, diversification and globalization following the nation's "Go Global" investment initiative, strengthen its core operation management of Boleholes and Water Supply, Water Plant Construction to achieve outstanding results both in quality and efficiency.
CULTRUE
Vision: Becoming a Centennial Corporation of China

Mission: Parter of International Economic Cooperation
Spirit : Hardworking Self-improvement
Value: Driven by Good Business Result
A Passionate Team of Soul
Ethics as the key to success
71.Jiangsu Construction Engineering Group Co., LTD (JSCE)
Jiangsu Construction Engineering Group Co., LTD (JSCE) was founded in 1989 with the grant of Jiangsu Government. Approved by the Ministry of Construction of superior building construction qualification, JSCE has also been qualified with First Class Contractor for municipal public works, electrical installation construction, foundation and foundation engineering, building decoration engineering, steel structure engineering, electrical and mechanical equipment installation, and Second Class Contractor for fire facilities engineering. JSCE has also obtained the qualification and operation of foreign contracted projects overseas labor export by the Ministry of Commerce.
The company now has engineering and economic management of 2350, including 180 senior engineers, 726 intermediate level, and junior title of 1030; 162 first grade architects, 405 second grade architect; and registered capital of 341.68 million yuan. It has constructed the comprehensive capacity of high-rise buildings, large industrial plants, and public facilities with large capacity, first-class decoration, large-scale municipal engineering, environment landscape engineering, and electrical and mechanical equipment installation projects. Since establishment, JSCE has designed and applied various operation and contracting methods, such as general design and construction, contracting, general construction contracting, professional contraction. JSCE has undertaken many large-scale important projects, including the Olympic Games and World Expo, many of which have been awarded "Luban" Cup, "Yangzi" Cup, "the Great Wall" Cup, "the Mountain Tai" Cup, "Magnolia" Cup and "Golden-steel" Cup.

The company, for many years, has been awarded by the Jiangsu Construction Association the "Best Enterprise" "Good Quality Management Units" in the field of Jiangsu construction, "Credit Business" and won the prize of "the contract and trustworthy enterprises" by the People's Government of Jiangsu Province. Winning the "2008 Construction of Jiangsu 30 Top Comprehensive Ability Enterprises" and "2008 Outstanding Construction Enterprises" JSCE has been certified by ISO900:2000 international quality system, ISO14001:2004 environment management system and GB/T28001-2001 occupation health and security management system.
Becoming more vital and competitive after being restructuring, JSCE invested to establish eight branches and absorbed the Building Research and Designing Institute of Changzhou City as one member group. With the provincial technical center, the company formed a various structures of design, research, general contracting, professional and construction services, new materials and production, winning a number of new technology applying and demonstration projects, the national labor law and national patents. JSCE also edits and co-edits a number of national industry standards, creating more economic and social benefits for the general contractor and operation, creation of new situation of technological innovation and opening up a wider operation space.

72.Shanghai Construction Group (SCG)
Shanghai Construction Group (SCG), which has been undertaking many important tasks in the

building and modernization of China’s cities, is the flagship of China’s building industry.

SCG possesses core technologies in the construction of high rise buildings, large bridges, light railways, public culture & sport facilities, large industrial plants, major environment protection projects & etc. At the same time, SCG has also completed about 100 landmark projects in more than 30 overseas countries and regions.

Shanghai Construction, with its business covering nearly the entire building industry, is capable of integrating all related social resources in its business. In the field of investment, construction and operation of urban infrastructure, Shanghai Construction owns a competitive & leading professional work force which could provide comprehensive general contacting service to the society. As the trade mark of Shanghai Construction, “SCG” has obtained the national certification of “China’s Famous Brand”.

Possessing a state-level technical center, a post-doctoral working station and teams engaging in technical research & development work at different levels, SCG’s professional work force is composed of academician of Chinese Academy of Engineering, great masters in surveying & designing, experts enjoying State Council perquisites and a large number of specialists in various technical fields.

SCG will keep to the core conception of “With harmony as the fundamental principle, Always pursuing the best”, and continue to promote the corporate spirit of “Science, Cooperation & Gumption”, and will spare no effort to provide all round & best quality service to all walks of life, so as to create more architectural wonders which will be passed on from generation to generation.

73.China Poly Group Corporation Ltd.

Under the approval of the State Council and the Central Military Commission, the former General Armament Department (GAD) of the General Staff Headquarters and CITIC Group cofounded Poly Technologies, Inc. tasked with the mission of importing foreign military equipment and exporting domestic military equipment in 1984.

Established with the approval of the State Council and the Central Military Commission, China Poly Group Corporation Ltd. is a large state-owned enterprise under the supervision and management of the State-owned Assets Supervision and Administration Commission of the State Council (SASAC). Over the past three decades, Poly Group has formed a development pattern with extensive businesses in multiple fields, including international trade, real estate development, light industry R&D and engineering services, arts and crafts raw materials & products management services, culture & arts business, civil explosives materials & services, information and communication technology, and finance. With business operations in more than 100 cities across China, Poly Group has ranked 174th among Fortune Global 500 in 2021 and has been rated A-level by SASAC for ten times. In 2021, Poly Group achieved an operating revenue of 445.12 billion RMB and a total profit of 56.81 billion RMB. By the end of 2021, its total assets reached 1.74 trillion RMB, and total tax contribution has exceeded 400 billion RMB.

At present, Poly Group owns 11 major secondary subsidiaries, more than 2,000 wholly-owned or majority-owned enterprises, 110,000 employees and 6 publicly listed companies at home and abroad, i.e. Poly Developments and Holdings Group Co., Ltd. (Stock Code: S.H.600048), Poly Property Group Co., Ltd. (Stock Code: H.K.00119), China Haisum Engineering Technology Co., Ltd. (Stock Code: S.Z.002116), Poly Culture Group Co., Ltd. (Stock Code: H.K.03636), Poly Union Chemical Holding Group Co., Ltd. (Stock Code: S.Z.002037), and Poly Property Services Co., Ltd. (Stock Code: H.K.06049).

74.ZHONGMEI ENGINEERING GROUP LTD(Kenya)

Zhongmei Engineering Group Ltd (hereinafter referred to as Zhongmei Group) is affiliated to Coal Geological Bureau of Jiangxi Province, with its headquarters in Jiulong Lake International Expo Center in the historical and cultural city of Nanchang. As an internationalized municipal group of special grade which starts from Jiangxi Province and conducts trans-provincial, trans-regional and transnational business, the group has been shortlisted for the Top 250 Global Conductors (ranking 111th in 2016) for five successive years.

Zhongmei Engineering Group is a company providing construction and engineering services. It offers its services in the construction fields of roads, housing, urban rail, mines, and hydropower, as well as the installation of mechanical and electrical equipment and geological exploration.

Zhongmei Engineering Group Limited is a leading import company in Kenya. Zhongmei Engineering Group., Ltd. offers construction contractor services. The company offers supervision engineering, engineering construction, investment construction, real estate development, geological engineering, energy engineering, surveying mapping and aerial survey, foreign aid projects, and international engineering.

75.Zhejiang Kangle Pharmaceutical Co., Ltd.

Zhejiang Kangle Pharmaceutical Co., Ltd., formerly named as Wenzhou Pharmaceutical Factory, was established in 1945. The company headquarter is located in Wenzhou, Zhejiang Province. Currently, we have two manufacturing sites. One is located in Wenzhou, Zhejiang Province while the other is Lianyungang Kangle Pharmaceutical Co., Ltd, which is 100% controlled subsidiary company of Zhejiang Kangle Pharmaceutical Co.,Ltd and located in Lianyungang, Jiangsu Province. We are the largest and professional manufacturer of Paracetamol and Paracetamol DC.

The other main API products are Erdosteine, Hesperidine, Silymarin, HTH (DL-homocrsteinethiolactone), Glucosamine HCl and etc. All of our products are manufactured in accordance with international quality standards and are greatly appreciated in different markets throughout the world.

We also have finished product. The main products are Famotidine Tablets, Acetaminophen Tablets, Compound Paracetamol Tablets, Polygeline Injection, Erdosteine Capsules, Compound Paracetamol and Chlorphenamine Maleate Granules, Pediatric Paracetamol, Atificial Cow-bezoar and Chlorphenamine Maleate Granules and etc.

We zealously welcome everywhere person from all walks of life to discuss the business and cooperate in the long term .Let’s develop together.

76.TIENS GROUP

Tiens Group was founded in 1995 by Mr. Li Jinyuan in Tianjin China, began its march into the international market in 1997. Today Tiens Group has become a multinational conglomerate, boasting of industrial capital, trading capital and financial capital. Its businesses cover fields like biotechnology, health management, hotel and tourism industry, educational training, e-commerce, finance investment and real estate, etc. And with its business reaching over 190 countries, Tiens Group has set up branches in 110 countries & regions and has established strategic alliances with top-rank enterprises from many countries. The diversified products developed by Tiens Group, such as health food, health care appliances, skincare applications and household products, are creating a high quality life for more than 30 million families around the world and have helped them to enjoy health, happiness, beauty and affluence.

With the development strategy of great health industry, Tiens International Health Industrial Park and Tiens International University, sponsored by Tiens Group, are planed to cover an area of 4.2 square kilometers with total investment of RMB 17 billion Yuan. Tiens International Health Industrial Park is a comprehensive industrial park including product research & development, product pilot, product manufacture, biological health study, hospitality industry, exhibition economy and so on. There is a "great health innovative technology system" basing on its R&D centers and QC centers with international standard; a "great health production logistics system" relying on its modernized manufacturing shops and automatic warehouses; a "great health service system" depending on Tai Ji Sun Hospital International Health Management Center, hotels, conference centers; a "great health education system" being backed by Tiens International University, parts of which have been implemented in an orderly manner, so as to form a chain of health industry linked up with the flow of human resources, products, finance and IT; and it is believed that Tiens International Health Industrial Park will, on this basis, grow into a "great health industry cluster" in its true sense, and lay a more solid foundation and provide even more opportunities for the development of Tiens Group.

Tiens Group has been adhered to its charitable philosophy of "originating from society and therefore contributing to society" and has been actively taking on corporate social responsibility. Tiens Group has donated thus far more than RMB 1.5 billion to public welfare and social charities.

A high quality workforce of professional talents, together with a localized and well regulated management, has contributed to the internationally strategic goals of Tiens Group. In addition, Tiens Group has an unbeatable international team in terms of research, innovation and professionalism; 35% of which hold master's degree or higher.

Based on the theory of "Business-oriented Thinking, Refined Management, and Refined Service", Tiens Group is currently practicing the advanced theories of "New Swap and Alternative Theory", "Consumption Makes Wealth, Operation and Consumption Makes More Wealth" as a foundation, guaranteed with the perfect operation management system and well-designed regulations, to take big strides towards World Top 500 with great confidence for the development of Chinese national industry.

Mission Statement: To provide global consumers with quality products and opportunities of education & cause, improve their life quality, and make the society harmonious.
Tiens Brand Core: One World, One Family;
Tiens Brand Core Value: Extraordinary Innovation 、 Paramount Responsibility 、 Excellent Teamwork;
Tiens Brand Slogan: Harmony, Unity, Prosperity;
Business Philosophy: Contributing to society by restoring health to mankind;
Quality Guideline: Parable to build human health, Milestone to realize technical innovation, Method to make sustained improvements, and Heart to satisfy consumers.
77.Zonsen Group
ZONSEN INDUSTRIAL GROUP
Zonsen Industrial Group Co., Ltd (hereinafter referred to as “Zonsen Group”) was established in 1982. In recent years, under the leadership of the founder Mr. Zuo Zonsen, Zonsen Group is transforming from the traditional manufacturing industry to the integration of industry, finance, and internet business, and striving to build a leading industrial internet ecological platform in the world.
Industry
Industry is the solid foundation for Zonsen to develop the industrial internet. Zonsen is known for its engine core technology. Its products include motorcycle engines, special vehicle engines, general engines, boat engines and aero-engines, and its end power products include motorcycles, special vehicles, garden machinery, small and medium-sized agricultural machinery. It is among the top in the industry in terms of motorcycle engines, general engines and tricycles. Over the past 38 years, Zonsen has been engaging in manufacturing business, and has grown into a power system integration expert and an industry leader in excellent manufacturing.
Zonsen focuses on new energy and aerospace power in the transformation and upgrading of industrial sectors. In the field of new energy, Zonsen invests in hairpin motors, electric controllers, continuously variable transmissions (CVTs), integration of electric power systems, wireless charging, hydrogen fuel cells, Electra Meccanica Vehicles, super capacitors and other projects, which can provide integrated power systems and solutions for new energy vehicles. In the field of aerospace power, the aviation engines independently developed by Zonsen have been mounted to large drones and plateau type unmanned helicopters and made successful trial flights, which is the first invented in China. It has made major breakthroughs in a number of technologies and achieved mass production.
Zonsen upgrades manufacturing business with new internet technology, builds platforms, shares resources, cooperates with partners, empowers the industrial chain, and achieves information-based, digital and intelligent manufacturing.

Finance
Zonsen Finance focuses on providing services for the upper and lower industries in the industrial chain.
Zonsen engages in small loans (including online small loans), factoring, finance leasing, and asset management business, and it can provide comprehensive solutions. Zonsen has investments in Chongqing Fumin Bank, whose main business is internet finance. The Zhong Yi Fund and the Betrust Fund affiliated to Zonsen invest in high and new technology, new materials, and new

types of business, which innovatively boosts the development of the industrial chain.

Internet Business

Driven by big data and intelligence, Zonsen actively explores the "advanced manufacturing + internet" model, creates a product life cycle value chain of smart research and development, smart manufacturing, smart products, smart service, smart finance, and gradually builds an industrial internet ecological platform that integrates the three platforms " 360humi.com + Automobile and Motorcycle Exchange + Master Zuo ".

360humi.com focuses on new smart manufacturing. It is one of the first national industrial Internet platform pilot demonstration projects released by the Ministry of Industry and Information Technology. With the commercial layer for the resource coordination and transaction services of the entire industrial chain, the core technology layer for logo decoding, intelligent manufacturing, and industrial APP, etc., and the application scenario layer for industrial Internet industrial park, industrial intelligent empowerment center, and logo decoding innovation center, it provides comprehensive services for traditional manufacturing enterprises, such as intelligent upgrading and cloud service.

Automobile and Motorcycle Exchange focuses on new finance. It is the only licensed exchange in the factor markets of the automobile and motorcycle industries in China. Based on product spot transactions, through transaction settlements, and financial empowerment, it extensively connects internal and external resources of the industry to create a credible exchange for transaction settlement and supply chain finance public services in the automobile and motorcycle industry.

Master Zuo focuses on new retailing. It makes full use of Zonsen's industrial heritage and product advantages of more than 30 years, mobilizes ecological resources such as products, channels, and customers to provide retailers with solutions for a retail upgrade, such as digital procurement, smart stores, precision marketing, vehicle sharing, instant delivery, and credit systems. It facilitates the application of network channels and helps thousands of retailers to do better business.

In the past, Zonsen was a manufacturer of small- and medium-sized engines and end products; now Zonsen is an enterprise integrating industry, finance, and internet business; in the future, Zonsen will be a leading industrial internet ecological platform in the world!

Enterprise Spirit:Unique, Exquisite, Earnest, Creative

Mission:a stock resources optimization enabler, an incremental breakthrough leader

Development Goal:to be a leading worldwide provider of small and medium power systems and industrial internet solutions

Vision:global influence and vitality for one hundred years!

78.Chongqing RATO Holding Group Co. Ltd.

Chongqing RATO HOLDING(GROUP) Co., Ltd. was established and founded in 2007. With years of dedication and development, RATO has grown into a well-known manufacturing corporation within the industry, owning multiple companies in the business of general dynamics, motorcycles, gasoline engines, diesel engines, and intellectualized industry.

With the core value of Innovation, Focus, Responsibility and Sharing, RATO has always believed in the significance of talent driven innovation. We move forward by constantly renewing and refining ourselves to create values for our customers. We Strive To Be A Well-Respected Chinese Company Around The Globe.

As one of the best representatives among the greatest industrial enterprises in Chongqing, RATO has earned many awards within its area of expertise, including but not limited to The Top 500 Manufacturing Companies in China, The Top 50 Most Profitable Companies in Chongqing, The Top 100 Companies in Chongqing in 5 consecutive years, The Top 100 Manufacturing Companies in Chongqing, The Merit Industrial Enterprises of Chongqing (from the year of 2012 to 2014).

Corporate Culture

Become a Chinese enterprise respected in the world

To be a Globally Respected Chinese Enterprise

“Innovation, Concentration, Responsibility，Sharing”

79.Guangzhou Kusing Generator Co., Ltd

Guangzhou Kusing Generator Co., Ltd is a professional manufacturer of generator sets, which produces diesel generator sets range from 10KVA to 2000KVA. Kusing has a modern factory covering more than 12000 ㎡, located in Guangzhou(Canton), Guangdong province, China. The products of Kusing are winning more and more customers’ approval and favor, and are widely applied to hotel, highway construction, mining, oil exploration, disaster relief etc. all over the world.

‘Humanity, innovation, efficiency, perfection’ is the belief of Kusing. We have a distinguished R&D team, which is made up of designers, engineers and technicians. To ensure the quality of products, we manufacture all the generating sets according to the International Quality of Certification: ISO9001:2000. Our productions have been with CE Certification,SUN-CAP Certification & SOSA Certification. For each new order we receives, we will strictly apply the manufacturing steps as follows:

1. Making a production plan;
2.Technology designing,
3. Supporting the procurement,
4. Manufacturing,
5. Performance controlling,
6.Testing,
7.Packing and warehousing.

With convincing quality, Kusing products endue generators ‘Made in China’ with a new implication-‘European quality with competitive price’.

Kusing has steady and long-term cooperation with famous companies, such as engine: CUMMINS, DEUTZ, PERKINS, LOVOL, VOLVO, YTO, SDEC, YANGDONG, etc., alternator: STAMFORD, MARTHON, LEROY SOMER, EVO-TEC, etc. The above engines give the diesel generator sets we made a strong and stable heart. And the high quality brushless alternators ensure the generators to supply electricity safely and stably for a long time. Nowadays, with great enthusiasm and persistence efforts, all Kusing members are working toward the goal of being one of leading enterprise in the international lines. Warmly welcome people from all over the world to visit our company and cooperate with us!

80.UNIVAL Group

As the new section of UNIVAL Group, UNIVAL Health focuses on the household medical health, rehabilitation and elderly care; Raising the vision to the personal health protection of the whole world, aiming to bring effective product solutions for the elderly and the people who are in the

process of rehabilitation, both market demand are increasing year by year. Standing in the post-COVID-19 era, we are more aware of the significance of the health protection to the whole society, which is regardless of national boundaries.
UNIVAL Health is just one of the several sections for the medical and health services bred by UNIVAL Group. In the past 30 years, we have been committed to manufacturing, importing and exporting all kinds of chemical materials, active pharmaceutical ingredients, intermediates, food additives, pharmaceutical and food machinery and equipment, pharmaceutical and food packaging materials for the chemical, pharmaceutical and food industries. With this conscientious work for the pharmaceutical industry, we have gained high recognition from customers all over the world. Now, we will continue to integrate the sense of social responsibility for the human being’s health into the construction of UNIVAL Health.
UNIVAL Health's products focus on the personal and household medical health, elderly care, rehabilitation and various of nutritional supplements. Passed by our strict review, all products have gained various quality certifications and meet with the full demand of the international market. We sincerely believe that more and more individuals and families will awaken their attention to health, have the ability of self-healing and rehabilitation, and obtain the right to "live and grow old with dignity ".
We look forward to working with you from all over the world to create health and prosperity!
81.TBEA Sunoasis
Founded in 2000, TBEA Xinjiang Sunoasis Co., Ltd. ("TBEA Sunoasis") is a global leading provider of green and smart energy service, with business in more than 20 countries and regions around the world. As a global leader in the photovoltaic, wind power EPC, inverter and other fields, TBEA provides overall solutions to development, investment (financing), design, construction, commissioning, and O&M for clean

ects mainly in PV power, wind power, power electronics, energy Internet and other fields.
TBEA provides overall solutions to development, investment (financing), design, construction, commissioning, and O&M for clean energy projects mainly in PV power, wind power, power electronics, energy Internet and other fields.
The Company has been awarded the titles of "National High-tech Industrial Base for New Energy Power Generation Equipment", "National New Industrialization Demonstration Base", "National patent and Innovative Pilot Enterprise" and so on. To be the world-leading green smart energy service provider.
TBEA focuses on intelligent products such as PV inverters, energy storage, TSVG, flexible DC device, and power routers, and provides services such as TB-eCloud intelligent energy management platform and smart microgrid solutions, striving to be "the world-leading green intelligent energy service provider".
One concept:Reliable
Two goals: Equipment for China, Equipment for the World
Three purposes:Satisfy Clients, Comfort Employee，Reassure Shareholder
Four Spirits:Hard Work, Vitality,Dedication, Learning
Five Worldviews:Integrity Management, Flexible Operation, Sound Development
Simple Process, Harmonious Cooperation
A global platform - rapid response and intimate service

An industry leader - data record for powerful strength

82.HAINAN Airlines of China

Hainan Airlines Holding Co., Ltd. (hereafter referred to as "Hainan Airlines") was established in January, 1993 in Hainan Province, China's largest special economic zone and free trade port. As one of the fastest growing airlines in China, Hainan Airlines is committed to providing passengers with comprehensive, seamless and high-quality service experience.

Since 1993, Hainan Airlines has been operating safely for 30 consecutive years. As of 2022, Hainan Airlines and its subsidiaries operated nearly 1,900 domestic and international routes. We had around 1,700 domestic routes, including all mainland provinces, autonomous regions and municipalities directly under the Central Government. As well as more than 200 international routes, including scheduled passenger flights, specific charter flights for work and school, and passenger-to-cargo flights, covering Asia, Europe, Africa, North America and Oceania regions, flying to 44 cities overseas. Hainan Airlines has actively responded to The Belt and Road Initiative by focusing on building a world-class airline network. Hainan Airlines and its subsidiaries have established operational bases/branches in 24 cities including Beijing, Haikou, Shenzhen and Guangzhou.

Hainan Airlines is the first and only SKYTRAX 5-Star airline in mainland China, who has been awarded the SKYTRAX 5-Star Airline for 12 consecutive years since 2011. This honor is Hainan Airlines highest level of recognition for our outstanding service, and also represents praise from the majority of our passengers for our high quality services.

On December 8, 2021, the actual control rights for the operation and management of the main business were officially transferred to the strategic investor Liaoning Fangda Group. In the future, Hainan Airlines will follow the corporate values put forward by the controlling shareholder Liaoning Fangda Group that, “operating an enterprise must be beneficial to the country, the enterprise, its employees and its customers” and will continue to aspire to build a world-class airline focusing on safety, service and profits.

Fly Your Dreams, Hainan Airlines.

Social responsibility concepts

With our core brand value of ‘Caring Love - Integrity’, Hainan Airlines has always regarded CSR fulfillment as an important approach and foundation for building a leading world-class enterprise. It has persisted in fulfilling its basic responsibilities towards the government, shareholders, employees, clients, cooperation partners, etc., and expanded its responsibility fulfillment to cover communities, the environment, public welfare and charity.

Safety culture construction

Safety is related to all employees and positions. Our safety education and training system covers every employee. We are devoted to building a safety culture with the core elements of integrity, love, discipline, responsibility, fairness, communication and learning. Safety culture is deeply rooted in every employee's heart.

Promoting cultural transmission

We adhere to the corporate culture of thinking globally and bringing benefits to all humans, uphold the essence of oriental culture featuring ‘people first, harmony and unification’, give full play to our advantages in the aviation industry during internationalization, and link social benefits with corporate benefits. Taking the spirit of the new commercial civilization as a guide to improving service quality, we endeavor to shape an international service brand and further meet

the needs of our customers through the combination of Chinese culture with the unique service concept of ‘Oriental Hospitality’. With Hainan Airlines’ accelerated pace towards internationalization, more and more international routes have been opened up. These new routes have brought new opportunities for China’s exchange with foreign countries in politics, economy, culture and other aspects, and also have built new channels and bridges for contact and interaction between Chinese and Western cultures in the new era.

Educational training for special personnel

Safety training for all Safety knowledge popularization In-flight safety is the core of air transport safety, in which the pilots play a crucial role. Our training aims to improve the pilots’ core competence. They are trained in many operation scenarios. The instructors are responsible for designing training contents, methods, evaluation standards and so on. With scientific training for special personnel, the quality of flight training has been greatly improved. We stick to a zero tolerance policy towards violations against safety culture, and every violation will be disciplined.

Democratic management

We strengthen democratic management and improve the communication and feedback mechanism. We adopt different approaches to listening to and adopting the opinions and suggestions of our employees.

83.CHINESE Xinjiang Zhongtai Chemical

Xinjiang Zhongtai Chemical Co., Ltd.is Xinjiang Uygur Autonomous region key support of the advantages of resource conversion enterprises, established on December 18th,2001, listed on the Shenzhen Stock Exchange on December 8th,2006, The predecessor of the enterprise is the Xinjiang Caustic Soda Plant built in 1958.

At present, the company has 43 wholly-owned, holding subsidiaries and 38 participating companies, including Xinjiang Zhongtai Import and Export Trading Co., Ltd., Zhongtai International Development (Hong Kong) Co., Ltd., with nearly more than 20,000 employees. Company's main PVC resin (PVC), ionic membrane caustic soda, viscose fiber, viscose yarn BDO PTA and other products. Products are widely used in textile, building materials, national defense and other more than 20 applications, in addition to the supply of Xinjiang market, but also exported to inland provinces and regions and exported to Russia, Central Asia, South Asia, South America and Africa and other countries and regions, enjoy high market awareness and credibility.

In recent years, the company has made great achievements in scientific and technological innovation, economic benefits, product quality, energy saving and emission reduction, resource utilization, environmental protection, brand strategy, production safety, social responsibility, enterprise management, human resources, enterprise culture construction and so on.

The company has been awarded Urumqi economic development "outstanding contribution enterprises "," tax outstanding contribution enterprises ", "Western Development of Xinjiang's best enterprises "," Xinjiang Autonomous Region Circular Economy Pilot Unit "," Urumqi City Air Pollution Control Advanced Unit "," Autonomous Region Civilization Unit "," China's top 500 chemical enterprises "," China's most growth chemical enterprises" honorary title. Won the 2011" CCTV Finance 50 Top Company "Award. May 2014, China and Thailand Chemical won the "China Industry Award Nomination Award ". In 2018 won the "Fifth China Industrial Award" and other honorary titles.

The company takes "enriching the people, prospering Xinjiang, strengthening the country" as the enterprise mission ," achieving world-class energy and chemical enterprises with world-class

status "as the enterprise vision ," harmony to the middle, faith to the Tai" as the enterprise philosophy ," people-oriented, public, harmony for the core values "," down-to-earth, hard work dedication, perseverance, self-improvement "as the enterprise spirit, while pursuing development, actively assume the responsibility of stakeholders, better achieve the common economic, social and environmental development of enterprises and stakeholders.
In the future development, the company will continue to establish a harmonious, mutual trust, mutual benefit and win-win relationship with all stakeholders to achieve the transformation, upgrading and vigorous development of the entire industrial chain.
Enterprise Mission:
Enrich People Promote Xinjiang Construct Motherland
Enrich People:
Benefit employees, benefit people's livelihood, make employees rich in economy, spirit and happiness.
Promote Xinjiang:
Enterprise development, for local economic construction, feeding society, for the stability of Xinjiang.
Construct Motherland:
Strong and excellent enterprises, to continuously enhance the competitiveness of China's chemical industry and unremitting efforts.
Status of Energy Enterprise Group
New era, new weather, new action
Integrate "Belt and Road ", vigorously promote supply-side structural reform, continuously improve technology readiness, continuously improve management level, continuously promote innovation and development, constantly consolidate industrial status, continuously strengthen capital function, continuously optimize scale level, continuously upgrade brand image, continuously expand space scope, continuously extend time span, constantly highlight beautiful chemical connotation, and achieve world-class energy and chemical enterprise group with world-class status, be the leader of innovation development, coordinated development, green development, open development and shared development.
For the sake of the public, filial piety is the first and the most important
people - oriented
Employees are the first resource of the enterprise, development depends on employees, development for employees.
Public first
National interests, national interests, enterprise interests to. On the whole,
Local interests obey the overall interests.
Filial piety comes first:
Grateful society, grateful enterprises, grateful parents, filial piety, good friends, abide by social ethics,
Professional ethics, family virtue, personal morality.
Tolerance, virtue, unity and cooperation, work together, live in harmony, harmonious development.
Pepsi for the people, all want to do the public ,100 good filial piety first
84.China ComService International (CCSI)

The Group is a leading service provider in the informatization sector in the PRC. Leveraging its position as a “New Generation Integrated Smart Service Provider” and targeting to become “the Main Force in Digital Infrastructure Construction, the Vanguard in Smart City Services, the Leading Enterprise in Industrial Digitalization Services, and a Trusted Expert in Smart Operation” (“1 Positioning, 4 Roles”), the Group commits to “Building Smart Society, Boosting Digital Economy, Serving a Good Life”. The Group provides integrated comprehensive smart solutions for the informatization and digitalization sectors. We offer telecommunications infrastructure services (including design, construction and project supervision and management), business process outsourcing services (including management of infrastructure for information technology ("network maintenance"), general facilities management ("property management"), supply chain and products distribution), and applications, content and other services (including system integration, software development and system support, and value-added services). The Company is also the largest telecommunications infrastructure service group in China with over 100 specialized companies that offer a range of professional services along our customers' value chain.

All the major telecommunications operators in the PRC as well as China Tower Corporation Limited are our customers. We also provide services to domestic non-telecom operator customers like government agencies, industrial customers and small and medium enterprises as well as overseas customers. Our service coverage is spread across the nation and we have also extended our business to dozens of countries and regions globally.

On 8 December 2006, the H shares issued by the Company were successfully listed on the Main Board of the Stock Exchange of Hong Kong Limited. As of 30 June 2023, the total number of issued shares of the Company was 6,926,018,400 shares, including 2,391,420,240 H shares and 4,534,598,160 domestic shares.

The Group is a leading service provider in the informatization sector in the PRC. Leveraging its position as a “New Generation Integrated Smart Service Provider” and targeting to become “the Main Force in Digital Infrastructure Construction, the Vanguard in Smart City Services, the Leading Enterprise in Industrial Digitalization Services, and a Trusted Expert in Smart Operation” (“1 Positioning, 4 Roles”), the Group commits to “Building Smart Society, Boosting Digital Economy, Serving a Good Life”. The Group provides integrated comprehensive smart solutions for the informatization and digitalization sectors. China Comservice has equipped with advanced technology, comprehensive business lines, healthy financial condition, versatile qualifications, extensive and localized service network and unique and integrated service model. The management team has extensive experience and excellent execution capability. China Comservice will develop strong capabilities to grasp market opportunities, build outstanding core competences, enhance intensive high-efficiency operations and realize stable growth in enterprise value, with a goal of being a "world-class information network builder", thereby becoming an important force contributing to the informatization of the community and an excellent enterprise satisfying its shareholders and customers, being trusted by its staff and recognized by the society.

85.Shanxi Construction Engineering (Group) Corporation

Top International Engineering Corp. (in short TIEC), the official foreign name of Shaanxi Construction Engineering Group Corporation (in short SCEGC), was founded in 1950. It is a large state-owned group enterprise under provincial government administration. TIEC is the first class

corporation in Shaanxi Province certified with top-class qualification of general contracting for building construction and class A engineering qualification of construction industry, which has been authorized to run international business. TIEC has integrative abilities to undertake construction investment, engineering exploration, design, construction and management， etc. TIEC also involves into other industries like urban mass transit, ready-mixed concrete production & delivery, building decoration and finishing, horticulture works for ancient style building and garden, boiler research and manufacture, logistics, real estate development, medical & health education, tourism and hotel business running, etc.

With extraordinary strength, the corporation always ranks on the list of Top 500 Enterprises of China, and Top 100 Competitiveness Enterprises in China Construction Industry. In 2015, the corporation ranked No. 212 of “ Top 500 Chinese Enterprise ” and No. 4 of Top 100 Competitiveness Enterprises in China Construction Industry.

With nearly 10,000 of professionals and technician personnel, including 87 professor level senior engineers, 1,508 senior engineers, 4,674 Chartered Constructors and Chartered Associated Constructors, TIEC has great advantages of construction talents resources in western region of China and is the locomotive of the provincial construction corporations in China.

In recent years, TIEC has implemented hundreds of scientific research projects, and has been awarded 92 national and provincial science and technology prizes, 6 Huaxia construction science awards by Ministry of Construction, obtained 334 national and provincial level construction methods and 235 patents and participated in 90 national industry norms editing and compiling. 40 projects had been awarded the Lu-ban Prize, the highest architectural prize in Chinese construction field, 33 projects awarded national high quality engineering projects, 2 projects awarded China civil works “Zhan Tianyou” Prize, and 12 projects awarded China Construction Steel Structure Golden Prize.

Following the principle of equal development both in and outside the province, both at home and abroad, TIEC has completed a number of important projects and its overseas subsidiaries have spread over 23 countries. TIEC is now taking the big strides towards the strategy of dominating the Shaanxi market, the national market and even the international market.

86.XCMG

XCMG is a top heavy machinery manufacturer in China. XCMG has the most complete construction equipment for sale such as excavators, loaders, road machinery, mining machinery, etc. It ranks 4th among global construction manufacturers including Caterpillar, Sany, Volvo, Komatsu, Hitachi and Liebherr.

Xuzhou Construction Machinery Group Co, Ltd. (XCMG) was founded in 1943. Since then, XCMG has stood at the forefront of the Chinese construction machinery industry and developed into one of the domestic industry's largest,most influential, and most competitive enterprise groups with the most complete product varieties and series.

XCMG is the 3rd largest construction machinery company in the world. lt is ranked 65th in the list of China's Top 500 Companies, 44th in the list of China's Top 100 Manufacturing Enterprises, and 2nd in the list of China's Top 100 Machinery Manufacturers. XCMG is dedicated to its core value of “Taking Great Responsibilities, Acting With Great Morals, and Making Great Achievements and its corporate spirit of being“Rigorous, Practical, Progressive, and Creative in order to keep moving towards its ultimate goal of becoming a leading world-class enterprise capable of creating real

value.

87.Northern International Group (NIG)
Established on March 8th, 1998, Northern International Group is combined by the advanced enterprises among state-owned foreign trade companies in Tianjin and it is the first sizable foreign trade group approved by Tianjin Municipality, ranking No.2 in the 50 top export enterprises in Tianjin. Nowit has nine enterprise members of colose level,which all have established modern enterprise system,seven import and export companies of them are classified among the 50 top export enterprises in Tianjin and three of them rank with the 500 largest import and export and export enterprises in China,the other two service companies are leading enterprises in their own fields as well.The Group has 2 branches in Tianjin engaging import & export business and warehousing & transportation.The Group also has over 110 enterprises of semi-close level with investment relationship,more than 30 of them are overseas enterprises or representative offices.
Approved by the Ministry of Foreign Trade and Economic Cooperation and Tianjin People′s Municipality,the core enterprise of the Group,Northern International Holding Co.,Ltd.(herein after called NIC)is a holding company and solely-owned legal body with first lot of registered capital of RMB 118 million and total assets of about RMB 3 billion.Its import and export volume in 1999 is US$420 mill-ion and export volume is US$368 million which covers about 40% of total export volume of the state-owned foreign trade companies in Tianjin.It expects that its annual grows of import and export volume will be 10%. NIC aimes at turning itself into a multinational company backed up with trade, industry,finance and technology and engages in trade,financing,information,service and development.
The staff of NIC sincerely welcome friends from all walks of life both at home and abroad to establish and promote the business relationships,and enjoy a prosperous future together through further cooperation.
NIC business scope:Import and export of commodities and technology in modes of transit trade, processing with material supplied,barter and agency;Domestic trade inclusive of wholesale and retail of varieties of commodities and materials,business on commission or franchise basis;Trade services including warehouse and transportation,labouring and contracting of engineering projects,info-consulting ,advertisement and exhibition,forwarding and shipping,special delivery and distribution as well as all kinds of services to foreign buying offices in China;Real estates.
88.Jiangsu Yulong Group
Jiangsu Yulong Steel Pipe Technology Co., Ltd. is located in Yuqi Industrial Park, Wuxi City, on the shore of the beautiful Tai Lake, with convenient water and land transportation. The company was established in September 2016 and inherited the manufacturing equipment and personnel of Jiangsu Yulong Steel Pipe Co., Ltd. The company employs more than 1,000 people and sells more than 500,000 tons of welded pipes annually. Yulong Technology is a Tier 1 supplier of Sinopec, PetroChina and Shenhua, and a qualified supplier of China Resources Gas, China Gas, and ENN Gas. The company has the right to import and export, and the proportion of export sales reaches 20-30%. Oil and gas pipelines and dredging pipeline projects mainly in the Middle East, Central Asia, Africa and South America.
The company has 6 production lines of Φ219——Φ2850 double-sided submerged arc welded pipe (SAWH) with an annual production capacity of 500,000 tons; 3 Φ406——Φ1422 SAWL

production lines, the most domestic JCOE forming method production line. The production capacity is 400,000 tons; one Φ800——Φ3600 coil welded pipe (RBE) production line with an annual production capacity of 30,000 tons; two 250x250 and 400x400 square rectangular pipe production lines with an annual production capacity of 200,000 tons. Two Φ1422 and Φ2850 3PP/3PE/FBE external anticorrosion and epoxy powder/epoxy resin internal anticorrosion production lines, with an annual production capacity of 3 million square meters of internal and external anticorrosion. Products are widely used in petroleum, chemical industry, natural gas, mineral slurry, tap water, sewage, heat and large steel structures and other fields.
Yulong Technology whole heartedly serves customers at home and abroad with advanced technology, equipment, complete product varieties and specifications, and high-quality product quality and reputation.
CORPORATE PHILOSOPHY
Yulong Technology, Casting Dreams More than 30 years of hard work, experienced the baptism of the market wave, With the revival of national industry, On the way forward, we blew the loud horn, Compose a splendid chapter for the rise of the private economy.
89.China General Nuclear Power Company
The preliminary work of Daya Bay Nuclear Power Plant in Guangdong province began in 1979, and China General Nuclear Power Corporation (CGN) was founded in 1994. Focused on the development of clean energies such as nuclear power, nuclear fuel, wind power, and solar power. Over 30-years’ experience in R&D and operation of nuclear power projects. 39,000 employees worldwide. The largest nuclear power operator in China. The largest nuclear power constructor worldwide
OUR MISSION IS: Developing clean energy to benefit mankind
OUR VISION IS: To build a world-class clean energy group
OUR BASIC PRINCIPLES ARE: Safety First, Quality Foremost, Pursuing Excellence
OUR CORE VALUES ARE： Doing Things Right in One Go
OUR BRAND SLOGAN IS : Natural Energy Powering Nature
STRATEGY OF CGN: professionalization, autonomization, market-oriented and internalization
BY THE YEAR 2020, CGN plans for the nuclear power installed capacity to remain no.1 domestically, and among the top 3 globally.
BY THE YEAR 2020, CGN plans for the aggregate performance indicator for wind power and solar energy to rank among the top three domestically.
90.Hainan Zhongchen Biological Engineering Company Limited
Hainan Zhongchen Biologic Engineering Co.,Ltd was established in July 2001. It is a high-tech enterprise specializing in large-scale planting,processing and related application technology R&D of organic Aloe vera for nearly 20 years.

Our company selected the best Non-GMO aloe vera seeds and established a world-class organic aloe raw material planting base in Hainan. Relying on the unique climate and environment, it provides excellent natural conditions for cultivating high-quality organic aloe vera fresh leaves. In order to ensure the quality of organic aloe vera, the company started with organic cultivation and adopted a large-scale, standardized farm farming model with a farm area of several thousand acres. With the use of organic fertilizers that are allowed to use, without any chemical fertilizers and pesticides, so that the quality of fresh leaves reach the organic and high-yield quality planting objectives, our company has passed the EU organic food certificate in

2003.

Our company carries out the operational principles of “faithful operation and quality first”. We have set up a quality scientific team from high starting point and high-quality, persisting in taking biological engineering as the leading factor with high standards and strict requirements. We also have set up a scientific research laboratory equipped with advanced equipment that can undertake the fine test and analysis on products. The company has an advanced aloe vera R&D center conducting a series of researches on the affect of aloe vera growing environment to its quality, the high efficiency and energy saving concentration technology at room temperature, dried powder production technique retaining the full biological activities of aloe vera in its process. The company’s research department developed independently, and constitute a several management regulations together with domestic and foreign scientific and research institutes, the equipment of the production technology and industrialization technology of our company have already been put in basic form and we also is the first aloe vera supplier in China who has the large organic aloe freeze dried equipment according with the GMP standard, that have offered both advanced software and hardware for us producing series products of the aloe vera raw materials.

Under the premise of ensuring the quality of organic aloe vera leaves, the control of organic aloe processing is also the key to the quality of organic aloe products. GMP production plant with 4500 square meters, strictly complies with the international regulations on the five key quality control factors of macromolecular substance: time, temperature, health, biological enzyme activity.,and keep the active ingredients and biological activity to the maximum. We also have jointly developed the concentration technology equipment used at room temperature with some domestic scientific and research institutes that thoroughly solve the technical obstacle of temperature rising and high energy consumption in concentration. The technical equipment is very effective in key quality control section to protect the biological enzyme activity of the product and to prevent the product from oxidation and brown stain. The equipment plays important role in protection of the biological enzyme activity of the product and the structure of macromolecular. The biological function ingredients content of our organic aloe vera extraction product have reached to the high-class quality standard comparing with other manufacturers in aloe industry.

Our company will continuously improve the organic production management and regulate the organic production process with higher standards. Both our company’s aloe vera plantation and processing are in line with EU(EOS) organic、JAS（Japan）organic and USDA (US) organic production regulation requirements, and the regulations of HACCP concerning fruit and vegetable juice products for export to the United States of America and the regulations of FSSC22000 and PAS 220 and meeting requirements with China’s exportation food hygiene and the registration requirements of safety regulations and ISO9001: 2000 Quality Management System and ISO14001: 2004 Environment Management System standard requirements, according to the standards of organic products and processing requirements, and has achieved the registration certificate of above all standards regulations , and meanwhile, we also have already acquired the IASC certificate issued by the International Aloe Science Council. These certificates are also a real verification of the quality of our product.

The company already has a strict organic planting management system, organic processing

extraction management system, quality control management system, scientific research and experimental management system, each link can reflect all the high quality organic aloe raw materials from our company's standard, professional management and faithful operation, delivering health and beauty brought by organic aloe vera.

91.Shenzhen Power Solution Limited

Shenzhen Power-Solution Ind Co., Ltd, established in 2004, a company that take "Improve the global Off-Grid population's life quality" as its company mission while dedicating to help off-grid population with China made green energy for the last 19 years.

19 years' hard working, Power-Solution gained core competence in innovation, service and cost control. We have provided solar light to 48.99 million people from 7.07 million households, generated 64.36 million KWH electricity, saved $17.65 million electricity cost for the BOP population, decreased 5.87 million tons of carbon emission, protected over 37,940 children from respiratory diseases that caused by using candles and kerosene lamp.

So far, 1/10 of the products certified by VeraSol are designed and developed by POWER-SOLUTION, and 1/5 of GOGLA member products are manufactured by POWER-SOLUTION.

Everyone has the right to enjoy the light. When the darkness is dispelled, freedom and hope reach to people's heart. Light up the last unreachable mile is our eternal wish.

Shenzhen Power-Solution lnd Co., Ltd, is established in 2004, aiming to create lasting impact in the renewable sector and the "Bottom of the Pyramid" population. And we are committed to provide the affordable green and clean energy to achieve sustainable development solutions.With 50+ patents for our manufactural products, we already have a wide range of innovative products Featuring the solar reading light through international partnerships and linkages like UNICEF and UNDP amongst others. We are keen in integrating 10 SDGs in our company's version. Many international organization like lFC, World bank, and United Nation Foundation visited us to prove we are a great company with social missions. And we constantly visit remote areas in Africa and Asia, to identify potential challenges our devices face in real situation.

Mission

Innovative design to maximize consumers' value, supply of most competitive products and services, and affordable solar energy. Integrated solar lighting solution to global BOP

Vision

Solar energy creates new life

Values

Integrity, innovation, professionalism, pragmatism, win-win

Business Philosophy

Build an enterprise through honesty, obtain sustainability through credibility, and achieve a win-win situation through promise keeping.

92.Esavior

LED & Solar Lighting & Renewable Energy Systems Leading Manufacturer since 2008

TUV Certified Factory| Supply to UN & The World Bank| IoT Technology Pioneer

Operating Since 2008, Supply to 100+ Countries

Founded in 2008, ESAVIOR GREEN ENERGY has become the leading manufacturer of LED & Solar Street Lights and Renewable Energy System Solutions. After years of continually innovating and improving, we have been highly praised by 120+ customers from over 100 countries for our turnkey renewable energy solutions.

Focus on LED Solar Lighting and Solar Products Manufacturing

We have integrated our wealth of experience and spirit of craftsman to our reliable LED Street Lights, Solar Street Lights, Solar Lights and other Green Energy System Solutions.

Full Own 3000+㎡ TUV Certified Factory

Our 3000+㎡ led and solar lighting factory is equipped with the most advanced technologies and equipments with full inspection capacities for all our materials, components and the finished products. In 2018, ESAVIOR factory was audited by both SGS and TUV-SUD and awarded ISO 9001/14001 certificate.

<0.3% Complaints Secured by Full Supply Chain QA System

Under our unique Full Supply Chain Quality Assurance System created in 2013 and keep on improving, we managed to lower our defects complaints within warranty period to 0.3%.

Successful UN, The World Bank, Government & NGO Project Experience

Supported by our reliable product quality and outstanding performance, our distributors in different countries have managed to win projects from government, UNDP, UNHCR, the World Bank and other NGOs. In 2014, ESAVIOR was appointed by Hunan provincial government as recommended supplier for solar street light government projects in China.

Comprehensive Understanding and Strict Respecting to International Standards

Our comprehensive certifications on international standards create unique advantages for our partners in project tendering. Our strict respecting to international standards and export experiences since 2008 ensured our products fulfill most standards of different markets.

National High-tech Enterprise with R&D Center Cooperate with Famous Universities

Our R&D center cooperated with the most famous Chinese technology universities Tsinghua University and South China University of Technology. With innovative technical support from universities, we managed to keep our unique position on solar charger controller, solar inverter, solar power systems and other renewable energy system solution technologies.

Our Independent R&D captured over 20 patents for us on solar and energy saving field. ESAVIOR also bas been qualified as “National High-tech Enterprise” since 2016.

IoT Technologies Pioneer with Experience Since 2014

Start in 2014, ESAVIOR have applied the most advanced IoT remote monitoring technologies to our LED street lights and Solar Street Light. Over 6 years R&D and application experiences in different countries created our unique pioneer position on the IoT Smart lighting field.

ESAVIOR IoT Solar Street Lights and Smart Street Lights have been installed in over 20 countries including USA, China, Italy, Singapore, India, Thailand, Kenya, Botswana and etc.

Professional Green Energy System Solutions Supplier

Our comprehensive understanding of all green energy technologies allows us to work out the most appropriate technologies or multi technology solutions to meet the individual requirements of our clients. Our core technology advantages, reliable QA system and maturing supply chain enabled us to expand our business to a wide range of green energy business range including led street light, solar street light, solar garden lights, solar lights, solar charger controllers, solar inverters, solar batteries, solar/wind hybrid street lights, solar power systems, solar/wind hybrid power generation, solar pest killing lamps and etc.

Reliable Global Green Energy Supply Chain Provider

Growing our business together with our partners, we are sharing our maturing and reliable green energy supply chain with our global partners. Start in 2017, we set up our first joint venture solar street light assembly plant in Egypt, now we are exporting not only the products, also our technology and the full supply chain on supplying in CKD and SKD to support our partners' "local manufacturing" plan in different countries.

93.Divine New Energy

Divine New Energy is a leading company that develops portable solar lights and solar system solutions, which are designed to provide sustainable and affordable lighting solutions to many segments including: energy poverty regions, as well as many indoor and outdoor applications.

With its proprietary technologies, Divine New Energy is working on empowering lives through clean and reliable off-grid solar products; to make our planet a better place.

Divine New Energy aims to improve the quality of life for the people who are lack of reliable electrical power. We develop and distribute affordable solar products and solutions for households and commercial customers, and provide customized solar power system integration based on professional technology ability and projects experience.

Divine New Energy is one professional solar products and system solutions provider. We aim to improve the quality of life for the people who are lack of reliable electrical power.

We develop and distribute affordable solar products and solutions for households and commercial customers, and provide customized solar power system integration based on professional technology ability and projects experience.

We are deeply committed to building a world class enterprise by operating efficiently and ethically, while delivering the highest level of quality in our products.

Divinergy is implementing a strategy to expand its global presence.

Vision

To improve people's quality of life accessing to reliable, affordable, renewable energy

To be the leading provider of affordable, renewable energy solutions.
Mission
To be dedicated to provide clean energy solutions for households & commercial customers
To leverage our technologies in order to expand the horizons of people
Goals
To help transform the lives of at least 10 million people before the end of 2020
94.Qingdoo Sunflare New Energy Company Ltd
Qingdao Sunflare New Energy Co., Ltd. is located at Qingdao, Shandong Province, China,which has superior location and convenient traffic conditions. Sunflare is a professional company specializing in the design, production and sale of solar power products. Sunflare is one of the companies with more than 20 series of solar products. The product range includes solar panel, solar power system, LED solar lantern/light and other solar products. Due to the high efficiency, low-carbon and environment friendly, high quality, Sunflare ’ s products have won a good reputation in America, Africa, Middle East, India and other countries and regions.
Guaranteed by our precise and highly efficient quality control system, well-trained employees, and top class facilities, Sunflare has strong capability of designing and cost controlling, at the same time, any OEM and ODM orders are welcomed.
Sunflare closely follows the developing steps of the world to research the solar series products meeting environment protection requirements. We sincerely hope to become your long-term supplier and make a bright future together with you.

95.China Southern Airlines
China Southern Airlines Co., Ltd. is directly affiliated with China Southern Air Holding Company Ltd., specializing in air transportation services. With headquarters based in Guangzhou, China Southern Airlines has its company logo seen around the globe with a brilliant red kapok delicately adoring a blue vertical tail fin.
China Southern Airlines possesses the most developed route network, and offers the largest passenger capacity among any airline in the People's Republic of China.
Currently, China Southern Airlines operates more than 850 passenger and cargo transport aircraft, including Boeing B787, B777, B747 and B737, as well as Airbus A330, A321, A320, A319 and ARJ21.The airline’s fleet ranks 1st in Asia (Data source: IATA, in terms of fleet size) .
Build International Hub with Canton Route
With continued efforts to establish itself as a network-oriented world-class airline with Chinese characteristics, China Southern Airlines has formed an extensive network across China that links Europe and Oceania and radiates throughout North America, the Middle East, and Africa from Asia.
China Southern operates more than 3,000 daily flights to 224 destinations in 40 countries and regions across the world.
For years China Southern has been committed to building up the Canton Route to turn Guangzhou into an international air hub. Now Guangzhou is becoming the No.1 gateway from China mainland to Australasia and Southeast Asia.
China Southern Airlines' safety commitment is - and will continue to be - unwavering both throughout China and globally. On June 15th 2018, China Southern Airlines was honored with the

2-Star Flight Safety Diamond Award by the Civil Aviation Administration of China (CAAC) as the leading Chinese carrier to maintain the highest safety records in China.
SKYTRAX’ s Four Star Airline
In 2011, China Southern Airlines earned its Four-Star status from SKYTRAX, the world's most prestigious airline performance evaluation organization.
China Southern was recognized as the Top 50 Most Admired Chinese Companies by FORTUNE China in 2012 and 2013.
In 2014, China Southern won the Stars of China Award 2014 by Global Finance Magazine based in USA and the China’ s Most Admired Company Award by Fortune China.
In 2015, China Southern was awarded Excellent A330 Operator by Airbus, and received China's Top 100 Valuable Brand Enterprise Award & Golden Pegasus Award in the 15th China International Transportation & Logistics Expo.
In 2016, China Southern was listed among the Top 500 corporations in China by FORTUNE China with the highest ranking among companies in the transportation industry.
In 2017, China Southern was named among the most valuable airline brands by Brand Finance, a famous international consultancy, ranking 1st in China and 6th globally.

In 2018, China Southern won the “World’ s Most Improved Airline” by SKYTRAX.
96.JMET Corporation, Jiangsu Sainty International Group
Jmet Corp, Jiangsu Saity International Group was established in 1974. It was formerly known as China Metals and Minerals lmport and Export Co, Ltd. in Jiangsu Province and Jiangsu Province Metals and Mierals Import and Export(Group) Co, Ltd. In the year of 2010, Jiangsu GuoxirInvestment Group Ltd. merged with Jiangsu Sainty International Group which had incorporated Jmet Corp. In November 2004. Since then, JmetCorp. has become a member company of Jiangsu Guoxin Investment Group Ltd.
The company focuses on both international and domestic markets. Under the combinaton of import and export trade and domestic trade, the online and offline platform, it has transformed from a traditional foreign trade enterprise based on import and export business to an integrated trader provides comprehensive solutions for customers. The company engaged in many fields, including steel, non-ferrous metals, hardware products, construction materials, chemicals, automobiles, household appliances, and imported consumer goods. It has established long-term and stable cooperative partnerships with sellers and clients around the world. At present, it is general agent of Japan's Mitsubishi Heavy Industries Refrigeration and Air Conditioning Systems Co.. Ltd in China. general agency for Vega Robledo Crianza of Juan Ramon Lozano, S.A.U. and general importer of Munchkin Inc.in China.
After more than 40 years of development and accumulation, the company has built a highly qualified management team and established a process supply chain management system and a normative and scientific internal control system. In 2001, it has attained IS09001:2000 quality management system certification. In 2016, it successfully upgraded and attained the ISO 9001:2015 certification. We uphold the business philosophy of growing with customers, looking forward to working together with all partners around the world for mutual benefits and development.
Business Type: Manufacture,Trade,Agent
Product Scope: Steel materials.Non-ferrous chemicals Iron and steel products,Automobiles and

air conditioners
97.CHINT
Founded in 1984, CHINT Group Co., Ltd. (hereinafter referred to as "CHINT") is a global leading smart energy solutions provider. Over the past 39 years since its establishment, CHINT has always focused on industry and brand building, deeply implemented the strategy of "Industrialization, Technologization, Internationalization, Digitalization and Platformization", and formed three major segments of "Green Energy, Intelligent Electric and Smart Low-carbon" and two major platforms of "CHINT International Platform and Sci-tech Innovation Incubation Platform", with its business covering more than 140 countries and regions. CHINT has 4 global R&D centers and 6 international marketing regions, and its manufacturing bases are spread over 16 countries and regions, with employees of more than 40,000 worldwide. In 2022, CHINT's operating revenue reached 18 billion dollars, and CHINT has been listed among the Top 500 Chinese Enterprises for more than 20 consecutive years.

CHINT seizes the new development opportunities of digitalization and dual-carbon goals and continuously strengthens the "One Cloud & Two Nets" strategy. Creating "CHINT Cloud" as the carrier of intelligent technology and data application, leading in building the Energy Internet of Things (EIoT) and Industrial Internet of Things (IIoT) platforms, aspiring to be explorers, advocates, and practitioners in the world of low-carbon development. With the "green energy, smart network, load reduction, and new storage" service systems, CHINT set up a platform-based enterprise, building a regional smart energy industry ecosystem. It provides a total energy solutions package for public institutions, industrial, commercial, and end users to achieve energy conservation, carbon reduction, and accelerate the energy transition.
98.China Engineering Company Ltd (CAMCE)
China National Machinery Industry Corporation (Sinomach), formerly known as the First Ministry of Machinery Industry, is a major State-owned enterprise directly managed by the central government. Sinomach has built itself on around 70 research and design institutes, equipment manufacturing and industrial and trade enterprises it took over from its predecessor.

Sinomach has more than 130,000 employees, over 28 wholly-owned or majority-owned subsidiaries, and 14 listed companies. It is a Fortune Global 500 enterprise and has ranked first among the top 100 enterprises in China's machinery industry for many years.

Sinomach is an international, diversified, comprehensive industrial equipment company, committed to providing quality services globally and building a world-class enterprise driven by science and technology. It has participated in emerging business development such as energy conservation and environmental protection industry and new energy industry.

With a focus on scientific and technological research and service, advanced equipment manufacturing, engineering contracting and supply chain, the company's business covers industrial basic research and development, high-end heavy equipment, high-end agricultural and forestry geological equipment, high-end textile equipment, design consulting and engineering contracting, supply chain integration services, automobile and exhibition, and industrial-financial investment.

Its services cover such critical national economic fields as machinery, energy, transportation, automobiles, ship building, metallurgy, construction, electronics, environmental engineering, aeronautics and astronautics, and light industry. The company is present in five continents, with more than 300 overseas service agencies in over 100 countries and regions.

The company aspires to become a leader in the machinery industry, a driver of progress for humanity, and a world-class enterprise with global competitiveness, continuing to contribute to the national strategy, national economy and people's livelihood.

For years, Sinomach has promoted further optimization of its resources, rebuilt its core competencies, accelerated its pace to go global and extensively advanced its strategic transformation with continuous technological, managerial and business model innovations. It has become a large comprehensive group with extensive business coverage, research and development strengths, high degree of internationalization and outstanding industrial chain advantages.

Sinomach adheres to the business philosophy of working together and pursuing win-win innovation and promotes harmonious cultural construction. It also strives to boost the value construction with contribution, innovation, green development, responsibility, and happiness at the core.

The company is determined to pursue high-quality development, and continues as a front-runner of China's machinery industry, an important supporter for the safety of the national industrial chain of major technology and equipment, and a significant platform for supply chain integration services of electromechanical products in China, helping further explore the international market for Chinese equipment.

99.RAYAL INDUSTRIAL

RAYAL INDUSTRIAL is a South African company specializing in tile manufacturing and distributing porcelain floor and ceramic roof tiles. RAYAL's products are supplied to the building and construction industry, as well as to wholesalers operating within these industries.

Through a focused marketing strategy, high quality products, exceptional customer care as well as being the first South African manufacturing company that produces porcelain tiles, RAYAL has already managed to grow exponentially.

SOCIAL RESPONSIBILITY

RAYAL has embarked on skills transfer programs that would benefit the South African team and the small enterprises/suppliers that form part of the overall supply chain. The current and future expansion plans of the company will see RAYAL contributing meaningfully to the Gauteng and South African economy whilst creating permanent jobs for previously disadvantaged individuals from within the region.

RAYAL, a leading tile manufacturing company, has adopted the most advanced Chinese technology and technological procedures in its operations. The company is equipped with supported automatic mechanical equipment, enabling efficient production processes. As a result,

RAYAL provides permanent employment opportunities to more than 200 South African citizens. Additionally, the company's commitment to sourcing raw materials from local suppliers contributes to indirect employment in the region.

RAYAL aims to increase its production capacity with the introduction of a second Production Line. The expansion will not only benefit the province but will create jobs for at least another 200 employees, resulting in tremendous economic and social benefits and also huge profit and economic sources to investors.

The second production line will primarily be producing Polished Porcelain Floor Tiles, Polished Glaze Porcelain Tiles, at the same quantities and production timeframe as the current production line.

OUR VISION

Our vision is to develop a business enterprise in the tile manufacturing sector that would become a leading commercial entity in the country and in the medium term, develop into one of the leading suppliers of porcelain tiles and other building materials to the African market.

The company ’ s expansion would see it develop into a company that would function and contribute to the empowerment of the Small Medium Enterprises and Job Creation in South Africa.

100.Nanchang Foreign Engineering Corporation

Nanchang Foreign Engineering Co., Ltd. (short for NFEC) was established in 1981, formerly known as Foreign Economic Cooperation Office of Nanchang Municipal Construction and Administration Bureau (It is called Nanchang Urban and Rural Construction Committee for now). In order to adapt to the development of foreign business, Nanchang Municipal Construction and Administration Bureau subsequently established the Foreign Economic Cooperation Office as Nanchang Foreign Construction and Installation Engineering Corporation. In 1992, the company was renamed Nanchang Foreign Engineering Corporation, and was renamed Nanchang Foreign Engineering Co., Ltd. in 2018 with a registered capital of RMB 79,900,000 Yuan.

NFEC ’ s main business scope includes contracting various projects in the domestic and overseas, exporting labour service to foreign countries and developing cooperation in the field of foreign labor service. NFEC now has first-class qualification as general contractor of housing construction projects and of Municipal public works, and second-class qualification as contractor of steel structures of groundwork and foundation, and of building decoration. NFEC has passed 1809001:2000 quality, environment and occupational health certification systems. As of the beginning of 2020, our company has more than 170 professional and technical personnel and managerial personnel.

NFEC consists of General Affairs Department (Office of Board of Directors), Party-Masses Affairs Department (Human Resource Department), Overseas Business Department, Safety Supervision Department, Enterprise Planning and Management Department, Operation Department, Discipline Inspection and Supervision Department (Legal and Audit Department), Labour Union Office, Finance Department, Engineering Department and other functional departments. NFEC has two subsidiaries, namely Jiangxi Zewen Construction Engineering Co., Ltd. and Nanchang Zhengzi Construction Development Co., Ltd., and has also four branch companies, namely Nanchang Foreign Engineering Co., Ltd., Provincial Branch, Nanchang Foreign Engineering

(Beijing) Co., Ltd., Nanchang Foreign Engineering (Nanjing) Co., Ltd., and Nanchang Foreign Engineering (Ziyang) Co., Ltd.

NFEC has carried out foreign business activities for decades and has established economic cooperation relations with partners in more than 20 countries and regions in the world. It has executed hundreds of projects at home and abroad.

While following the business philosophy of technological innovation and "Strengthening the Enterprise by the Talents", emphasizing on strategic development and transformation, NFEC not only aims to carry out engineering construction and achieve a diversified industrial extension, but also to develop domestic and international markets simultaneously, and to improve its core competitiveness continuously, striving to become a first-class export-oriented modern enterprise group in Jiangxi Province.

101.Ekemp International

EKEMP Focuses on Digital Biometric Security Identification, Providing Customers and Partners with Reliable Products and Solutions that Drive the Process of Government and Social Digitalization.

Founded in 2006, EKEMP Electronics Ltd. is hi-tech enterprise and focusing on R&D, manufaturing of industrial mobile computing Terminal, EFT-POS terminal, automatic Identify products. EKEMP International Ltd is professional on research and development the Barcode Technical , Wireless RFID technical, electronics payment, Industrial mobile data collection terminal, applicated terminal collection together real-time message with enterprise backstage system, business processing, Logistics and Supply-Chain management system in all. These have brought outstanding mobile productivity to Logistics, manufacturing industry, Retail business and Medical & Healthy care.

In China, EKEMP has branch office and Factory in Beijing, Shenzhen, HongKong. We owned professional applicated consultancy and abundant technical force. Over 30 engineers and designers have bachelor and master degrees in telecom, electronics, and mechanical engineer. Our company devoted in created group which is study innovative. Cooperate inseparably, harmonious development. Our company are the top of this industry. EKEMP is searching for development and innovation as innovationist. Transfer the most advanced mobile data collection terminal and application solutions to all of our customers, Increase comprehensive competition. Common benefit with our customers and our partner Company Strategy: Shenzhen EKEMP Electronics Co., Ltd adhere to the direction of customer, Strategy of Application oriented, Devote in develop wireless modules, smart phone, and mobile date equipment, automatic identify equipment and the application. EKEMP electronics Ltd. win the honor beyond comparison basic in excellent enterprise reputation. Professional industry knowledge and IT Strength. And become long-term cooperation partner with the famous logistics and industrial groups in China and overseas.

102.Haishan Group International

China Hasan International Holdings Co. Ltd. (hereinafter referred as Hasan International) is an enterprises group incorporated at Hong Kong, with a registered capital of 2 billion HKD.

Hasan International is a multinational corporation which is engaged in transnational investment and global management. Being Headquartered in Beijing, Hasan International has established

dozens of branches and subsidiaries in Guangdong, Sichuan and Qinghai provinces of mainland China as well as in Angola and Ghana.

Its business scale covers real estate development at domestic and international level, investment in industrial parks and mineral resources, overseas projects contracting, international logistics, multi-national trading, financial investment, hotel management and new-resource development.

Being a forerunner and practitioner among the non-governmental private enterprises in China who adhering to the national development strategy of “Going Global” and expanding their businesses overseas, Hasan International has been successfully operating its business in Africa for nearly 6 years since it set foot on it in early 2006. Guided by the notion of "taking root in Africa to serve Africa", Hasan International is committed to bridging between China and Africa, hence achieving its rapid development and growth in the African countries.

While based in Angola and Ghana, Hasan International has been actively expanding and developing its business to more and more African countries. In Angola, Hasan International has successfully constructed and operated some of the most renowned national projects, including the municipal housing projects in CAMAMA and NOVA VIDA as well as the VILA AZUL, SAMBIZANGA and CUNENE projects. In addition, Hasan International invested and constructed the Rose Garden housing project, which is located in the capital city of Luanda and covers a total area of 140 hectares and a planned construction area of 800,000 square meters, with an overall investment of 1.5 billion USD. In Ghana, by virtue of its comprehensive strength, Hasan International has been appointed by the Ghana government as the sole developer of the well-known Secondi Bonded Industrial Park, holding the responsibilities of planning and designing, construction and maintenance, investment promotion operation administration, etc. The Phase one of the Secondi Bonded Industrial Park covers a planned area of 10 square kilometers, with an initial investment of about one billion USD on infrastructure constructions.

Being a successful private enterprise that accumulates in a wide range of project and social resources in the progress of international business expansion and development, Hasan International has been one of the most active explorers in the implementation of the national development strategy of "Going Global".

103.Kerui Company

KERUI Petroleum & Gas Company is an international integrated industrial group which is committed to making it more efficient in the extraction of oil and gas, providing customers with one-stop solutions including R&D of high-end oil and gas equipment, oil and gas energy engineering and oil field integration services etc.

KERUI’s marketing service network covers Asia - Pacific, Central Asia, Middle East, Europe, North America, South America and Africa areas which include more than 52 countries and zones. She owns 10 technology R&D centers and laboratories around the world and offers integrated high - end technical support to global customers. Meanwhile,12 regional service centers and parts

warehouses are separated around the world.

KERUI Petroleum & Gas company has seven large oil equipment manufacture bases which include 9 series and more than 400 categories on drilling and workover equipment, gas compression equipment, nitrogen generation and injection unit, oil & gas stimulation equipment, wellhead & well control equipment, artificial lifting equipment, off-shore oil & gas exploration equipment, natural gas processing equipment, carbon capture and application equipment.

KERUI has gained the API Q1 quality management system certification, and DNV QHSE system certification, possesses the right to use the logo of API 4F and 7K, and acquired US ASME certification, EU CE marking, Russian CU-TR certification, 3C certification and CNAS laboratory accreditation qualification， qualified by the Authorized Economic Operator (AEO) Program of the World Customs Organization (WCO), KERUI can provide whole life cycle of retrospective management for their products according to international mainstream quality control standards.

Currently, it provides high - quality services and practical products to customers in more than 80 countries worldwide.

KERUI devotes itself to make it more efficient in oil and gas extraction, firmly believes that technology leads the future and service creates value; adheres to ideas on customer - centered, requirement - oriented; adopts its sincere, professional, dedicated craftsmen spirit and joints our efforts to promote the development and progress of global oil and gas industry.
104.GEO Chinese Company
China Energy Conservation and Environmental Protection Group (CECEP) is a central enterprise founded in 2010 after the merger between China Energy Conservation Investment Corporation and China New Era Group Corporation with the approval of China’s State Council.

As a centrally-administered state-owned enterprise specializing in energy conservation and environmental protection, CECEP has stayed true to our mission of “conserving resources and protecting the environment” since our founding, making significant contributions to the emergence, development and growth of China’s energy conservation and environmental protection industries.

At present, CECEP boasts more than 700 subsidiaries, including seven listed companies. Our business has expanded across China and beyond, radiating to 110 countries and regions around the world. With a “3+3+1” business structure, namely three main segments (energy conservation and clean energy supply, ecological environmental protection, and life and health), three green businesses (green building, green new materials, and green engineering services), and strong strategic support capabilities, CECEP has emerged as a flagship enterprise in China’s energy saving and environmental protection field with the largest scale, the most complete range of solutions, the widest coverage, and the strongest competitiveness.

In recent years, CECEP has developed advantages across the whole industry chain of energy conservation and environmental protection from planning and consulting to R&D and design,

investment and development, equipment manufacturing, engineering construction, operation and management, and investment and financing services. Our business basically covers all segments of energy conservation and environmental protection, enabling us to provide energy and environmental solutions for the green development of a region or a river basin.

In the new era, CECEP has resolutely implemented the decision and deployment of the CPC Central Committee, actively participated in major national strategic missions such as Great Protection of the Yangtze River, coordinated development of Beijing, Tianjin and Hebei, construction of Xiong’an New Area, integrated development of the Yangtze River Delta, ecological protection of the Yellow River Basin, high-quality economic development, construction of the Guangdong-Hong Kong-Macao Greater Bay Area, and construction of the Hainan National Ecological Civilization Pilot Zone, and taken an active part in the Belt and Road construction. In May 2018, CECEP was designated by the leading group office for promoting the development of the Yangtze River Economic Belt as the main platform for pollution control in the Yangtze River Economic Belt.

Looking into the future, CECEP will continue to remain true to our original aspiration of saving resources and protecting the environment and keep our mission of meeting the growing needs of the people for a beautiful ecological environment firmly in mind. In accordance with the requirements of high-quality development, CECEP will accelerate the construction of a world-class conglomerate specializing in energy conservation, environmental protection and health industry while striving to make greater contributions to building a beautiful China and a clean, beautiful world.

105.China General Nuclear Power Group

CGN：A leader in Clean Energy

The preliminary work of Daya Bay Nuclear Power Plant in Guangdong province began in 1979, and China General Nuclear Power Corporation (CGN) was founded in 1994.

Focused on the development of clean energies such as nuclear power, nuclear fuel, wind power, and solar power.

Over 30-years’ experience in R&D and operation of nuclear power projects.

39,000 employees worldwide.

The largest nuclear power operator in China

The largest nuclear power constructor worldwide

OUR MISSION IS: Developing clean energy to benefit mankind

OUR VISION IS: To build a world-class clean energy group

OUR BASIC PRINCIPLES ARE: Safety First, Quality Foremost, Pursuing Excellence

OUR CORE VALUES ARE：Doing Things Right in One Go

OUR BRAND SLOGAN IS : Natural Energy Powering Nature

STRATEGY OF CGN: professionalization, autonomization, market-oriented and internalization

BY THE YEAR 2020

CGN plans for the nuclear power installed capacity to remain no.1 domestically, and among the top 3 globally.

BY THE YEAR 2020

CGN plans for the aggregate performance indicator for wind power and solar energy to rank

among the top three domestically.
106.China's Shenzhen Xinguodu Technology Co., Ltd
XGD focuses on innovation in the fields of electronic payment technologies and persists in “innovation” as the core concept of enterprise.
NEXGO has been dedicated to fintech for over 20 years. We are offering high-tech payment devices, solutions, and technologies to customers all over the world. Whether you're running a startup, scale-up, or global enterprise, NEXGO products perfectly meet your demands.
NEXGO was founded in Shenzhen,China.
Mission Create Better Lives
With the rapid development for over 20 years, NEXGO now is a worldwide influential fintech enterprise. We believe that technology is the foundation of vibrant lives, thriving communities and forward progress. Therefore, NEXGO is committed to enhancing our capability to bring your business to new height.
NEXGO Spirit
Innovation | Focusing Professionalism | Cooperation
Vision
Sustainable and Eternal NEXGO
We believe that only a healthy and long-lived company can fulfill its mission and make contributions to human society.
107.Xinlan Group
Xinlan Group Co.,Ltd is a national non-area-limit enterprise group which was founded in 1997. It is a modern large-scale integrated cable company, integrating scientific research, design, production, marketing and service as an organic whole.It has 3 companies, and total area reaches 210000 squares meters.General headquarters is located in Liushi, the electrical capital of China.

We specialized in producing rubber sheathed cables、mining cables、power cables、control cables、communication cables、special cables、cables for civil purpose、heat shrink and cold shrink cable accessories、 cable connector and so on. The products have more than 10 major categories and 2000 specifications. Over 450000KM wires and cables are manufactured and sold every year. We are one of the largest manufacturers of cables in China. Out sales market includes 30 states and regions from America、 Africa、 Southeast Asia and Middle East. And our products are deeply trusted and welcomed by domestic and international users.
OUR BELIEF
XINLAN GROUP CO., LTD. develop and grow up rapidly. The most important thing
is that they unswervingly adhere to the "three convictions":
1.respect employees' personal beliefs.
2.respect customer's beliefs Their resounding slogan is "integrity" . They take two measures to ensure the quality of service:
First, select and train excellent staff.
Second, choose and train engineers for customer service.
3.dream Perform all tasks for dreams.
THE CORE VALUE
XINLAN GROUP CO., LTD. has five core values:
1.believe and respect for all individuals;

2.pursue higher and better achievement;
3.do things honestly, no cheating users or employees and can not do things against the integrity of things;
4.the company's success is to rely on all employees, not by a single person;
5.believe in constant innovation and do things with creativity.
OUR CULTURE
What are the cultures of XINLAN GROUP CO., LTD. ?
1.production power;
The slogan of XINLAN GROUP is scientific and technological innovation, become the leading one in the world. This is also the significance of the existence of XINLAN.
2.customer;
As an enterprise, it is necessary to listen to and understand the needs of customers, and then meet these needs, and then exceed customer expectations. Our mission is to become an innovative enterprise, and has become highly respected enterprises in the market. The enterprise must make sure the high quality of products, and continue to develop new ones, creating a higher value for customers.
3.staff;
An enterprise,no matter from which country, ultimately need people to realize its value. No staff, no enterprise. Commonly companies regards customers as god. But in XINLAN GROUP, employees and customers are equally important for the company.
4. investors;
Companies must give investors a reasonable return. Of course, investors should not only look at corporate profitability, but also look at the development of enterprises in the face of risks and whether it has a more development prospects. So, how to let investors in favor of your business? Enterprises not only need to have courage, but also have the ability to better and long-termly survive, and has a good foresight for the future.
5.social responsibility;
To maximize the wealth of shareholders or the pursuit of maximum profit is not the main driving force or the primary objective of XINLAN GROUP. In addition to making money, we are still actively take up the responsibility of social citizens in the country and region where the enterprise is located. The tax is part of them. Equally important is environment friendly. Environmental protection is closely related to the health of all the people.
HONOR AND CONCEPT
Our corporation has obtained honorary titles of AA Enterprise Assessed by General Administration of Quality Inspection, “National Hi-tech Enterprise”、”National Machinery Industry 500”、”Science and Technical Department Spark Program” and ”Star enterprise and Key enterprise of Yueqing&Wenzhou”. The trademark ”新缆 XINLAN” has been awarded as a “Well-known Trademark in China”, “High&New technical product of Zhejiang” and a series of products were named as “Brand-name product of Wenzhou”. The Xinlan Company is a rising star of cable industry in China.
With the company concept of being United、Honest、Pragmatic and Innovative, we always provide more and more credible products and better service to requite customers and societies. The Xinlan Company will be sincerest cooperation and common development with customers at home and abroad.

108.China Railway Construction Corporation Limited (CRCC)
Formerly the Railway Engineering Corps, China Railway Construction Corporation Limited (CRCC) was solely established by China Railway Construction Corporation on November 5, 2007 in Beijing. Now we are a mega size construction enterprise under the administration of the State-owned Assets Supervision and Administration Commission of the State Council. We were listed in Shanghai (601186.SH) and Hong Kong (1186.HK) on March 10 and 13, 2008 respectively, with a registered capital of 13.58 billion yuan.

As one of the most powerful and largest general construction groups in the world, we were ranked 42nd among Fortune Global 500, 3rd among ENR's Top 250 Global Contractors and 12th among "China's Top 500 Enterprises" in 2021.

We have operations in 32 domestic provinces, autonomous regions and municipalities, as well as more than 130 countries and regions in the world, covering project contracting, planning and design consultation, investment operation, real estate development, industrial manufacturing, materials logistics, environmental protection, industrial finance and other emerging industries. We have developed from an enterprise mainly engaged in construction contracting into one with an integral industrial chain involving research, planning, survey, design, construction, supervision, operation, maintenance and investment & financing, which enables us to provide customers with one-stop integrated services. We play a leading role in terms of the engineering design and construction of plateau railways, high-speed railways, highways, bridges, tunnels and urban rail transit. We have a member of the Chinese Academy of Engineering, 11 National Survey & Design Masters, 11 experts included into the "National Talent Program" and 251 experts enjoying the special allowance from the State Council. We have 81 projects winning the National Science & Technology Award, 112 projects winning the Tien-yow Jeme Civil Engineering Prize, 444 projects winning the National Quality Engineering Award and 148 projects winning the China Construction Engineering Luban Prize, 3,182 provincial and ministerial construction methods, as well as a total of 19,072 patents.

We have upheld the new development philosophy, followed the policy of "pragmatism, integrity, innovation and sustainability", endeavor to serve national economic and social development and meet the people's yearning for a good life, focused on the government, the city and the people, spared no efforts to build a "quality-oriented enterprise", leveraged our advantage in the whole industry chain, provided customers with one-stop integrated services, and forged ahead with the vision of becoming the most trustworthy world-class general construction industry group.
Business
Project contracting is the core and traditional business of the company. It covers railway, highway, urban track, water conservancy and hydropower, housing, municipal, bridge, tunnel, airport and wharf construction and so on. It operates in 32 provinces, autonomous regions, municipalities directly under the Central Government and Hong Kong, Macao Special Administrative region, including Taiwan Province, and 116 countries in the world. Construction contract mode and financing contract mode are mainly used in project contracting business.

With the active implementation of "The Belt and Road" construction by the state, the

coordinated development of Beijing-Tianjin-Hebei region, the development of the Yangtze Economic Belt, the construction of the Xiong'an New Area and the Guangdong-Hong Kong-Macau Greater Bay Area, as well as the continued in-depth promotion of the new urbanization, the strategy of rural revitalization, and the transformation of shantytowns, To strengthen the improvement of transport facilities in the central and western regions, the Company's domestic railway, highway, house construction, urban rail, municipal and water conservancy, hydropower, airport and other fields will continue to maintain high operation, military and civilian integration, rural construction, Utility tunnel, Sponge city, greens environmental protection, pollution abatement and other emerging markets are expected to grow rapidly. Although the structure of the market will have further changes, the overall infrastructure market is in a steady and rapid development trend.

Overseas

China Railway Construction Corporation Limited (CRCC) has joined the United Nations Global Compact Organization, with overseas operations in nearly 100 countries and regions. Since participating in the Tanzam Railway and the Pyongyang Subway in the 1960s, CRCC has been involved in the construction of the second phase of the Ankara-Istanbul High-speed Railway in Turkey, the Benguela Railway in Angola, the Railway Modernization Project in Nigeria, the Algeria East-west Highway and other landmark projects with significant international influence. Refreshing the record of single contract amount of China's Foreign Contracting projects several times.

39 African countries, namely Algeria, Libya, Sudan, South Sudan, Madagascar, Nigeria, Botswana, Niger, Angola, Zambia, Tanzania, Benin, Guinea, Sierra Leone, Ethiopia, Chad, Mali, South Africa, Djibouti, Comoros, Rwanda, Côte d'Ivoire, Ghana, Uganda, Congo (Burundi), Namibia, Togo, Mozambique, Cameroon, Burundi, Cape Verde, Egypt, Zimbabwe, Kenya, Morocco, Equatorial Guinea, Senegal, Eritrea and Malawi.

CSR

Social Responsibility Concept

China Railway Construction Corporation Limited (CRCC) adheres to the concept of rewarding shareholders and society, insisting on taking development as the first priority, operating in good credibility, strengthening management, and continuing to grow with good economic benefits and company growth. Give back to investors and society.

CRCC sticks to the concept of balancing growth between enterprises and employees, striving to build a harmonious enterprise. Give full play to the enthusiasm of employees to participate in the management of enterprises, safeguard the legitimate rights and interests of employees, ensure the safety and health of employees and improve living conditions.

CRCC advocates the concept of "Green Engineering", committed to the construction of resource-saving and environment-friendly enterprises. Strengthen energy conservation, emission reduction and environmental protection to achieve coordinated development between enterprises and the environment.

-In the construction of large-scale projects, we have actively created employment opportunities and absorbed more than 1 million people in the social labor force each year, which has played a

positive role in alleviating the pressure on social employment and ensuring social stability.

-In the event of disasters, CRCC has always been in the lead, becoming the main force in rescue and disaster relief.

-CRCC and its employees actively participated in poverty alleviation, education assistance when the people in poor areas encounter difficulties.

Scientific Research Achievements

The company has implemented innovation-driven development, improved the scientific and technological innovation management system and mechanism, vigorously strengthened the work of scientific and technological innovation, accelerated industrial technological innovation, and used high and new technology to enhance the competitiveness of enterprises. The company has made a breakthrough in a series of major technologies, grasped numerous core technologies, obtained rich achievements in scientific and technological innovation, and cultivated a number of high-level scientific and technological innovation platforms, teams and talents. At present, CRCC has 19 national innovation platforms and 9 postdoctoral research workstations. In 2017, the investment in science and technology amounted to 10.3977 billion Yuan.

CRCC presided over and participated in the development of 12 national standards; 1719 authorized patents, of which 375 were invention patents; 363 provincial and ministerial level working methods; 10 Demonstration Projects of innovative Technology Application in National Construction Industry. Several honors are awarded to CRCC in 2017.

National Prize for Progress in Science and Technology

Provincial Award of Scientific Research Advancement

China Patent Awards

Consulting Award for Survey and Design at or above the Provincial level

Feddick Engineering Project Awards

China Civil Engineering Zhan Tianyou Awards

And More...

109.CRRC Corporation Limited (CRRC)

Headquartered in Beijing, CRRC Corporation Limited (CRRC) has 46 wholly-owned and majority-owned subsidiaries with over 170,000 employees.

CRRC is the world's largest supplier of rail transit equipment with the most complete product lines and leading technologies. Its main businesses cover the R&D, design, manufacture, repair, sale, lease and technical services for rolling stock, urban rail transit vehicles, engineering machinery, all types of electrical equipment, electronic equipment and parts, electric products

and environmental protection equipment, as well as consulting services, industrial investment and management, asset management, and import and export.

CRRC will improve its technological innovation system, and constantly upgrade its technological innovation capabilities. It has built the world ’ s leading rail transit equipment technology platform and manufacturing base. Its world-class products like high-speed trains, high-power locomotives, railway trucks, and urban rail transit vehicles can adapt to various complex geographical environments and meet diverse market needs. The high-speed trains manufactured by CRRC have become one of the jewels in China ’ s crown to showcase China's development achievements to the world.

In the future, our mission is to connect the world through better mobility, and our goal is to continue innovation, reform and internationalization. CRRC will vigorously implement the strategy for internationalization, diversification and collaborative development, and strive to be the world's leading provider of high-end equipment system solutions with rail transit equipment as the core, which is capable of multinational operations.

CRRC is committed to supplying products and services that offer energy-conservation, eco-friendliness, cost efficiency, intelligence, speed and comfort. Joining hands with you, our dear customers and friends, CRRC will find the most effective solutions for the sustainable development of railway transportation.
Chairman's Address
Welcome to CRRC website! Thank you for your attention and support to CRRC!

CRRC was merged and established by CNR and CSR. It inherited the excellent culture of CNR and CSR, inherited the Centennial accumulation of China's rail transit equipment manufacturing industry, and carried the dream of China's high-speed rail to the world. On June 8, 2015, CRRC was successfully listed on Shanghai Stock Exchange and Hong Kong stock exchange. The establishment of CRRC is an important measure for China's rail transit equipment industry to enhance its core competitiveness and build a world-class multinational enterprise. It is an important milestone in the development of rail transit in China and even the world, and opens a new journey of internationalization of China's rail transit equipment.

CRRC adheres to independent innovation, open innovation and collaborative innovation. It has a world leading R & D and manufacturing platform for rail transit equipment. Its products such as high-speed EMUs, high-power locomotives, railway freight cars and urban rail vehicles have fully reached the world advanced level, and can adapt to various complex climatic and environmental conditions such as high temperature, high humidity, high cold and sandstorm, as well as diversified individual needs, It is one of the enterprises in the global rail transit industry to achieve full coverage of product types. At present, CRRC's products have been exported to nearly 100 countries and regions around the world, and have gradually changed from product export to technology export, capital export and global operation. The global operation network has basically taken shape.

Starting point of China's China one belt, one road to the new opportunities and challenges, China's Middle East will continue to uphold the ideals of revitalizing the national industry and make the best of the central enterprises. We will take the opportunity of the "one belt and one road" and "China made 2025" to build global resources, integrate global resources and integrate international production capacity with global vision, industry development and open mind. Vigorously promote business model innovation, technological innovation and management innovation, improve business quality, comprehensively build a global leading and multinational high-end equipment system solution supplier, promote China's high-end equipment to the world, and repay shareholders and society with excellent performance.

Sincerely hope that friends from all walks of life will continue to pay attention to and support CRRC! We will sincerely cooperate with all sectors of society to move forward hand in hand and jointly create a better future!

Products Services

Rolling Stock

CRRC specializes in design, manufacture, testing, commissioning and maintenance of locomotives and rolling stock, including electric locomotives and diesel-electric and diesel-hydraulic locomotives from 280 kW to 10,000 kW for mainline and shunting duties, high-speed trains with speed over 350 km/h, DMUs and EMUs for urban, suburban and regional transport, trams and light rail vehicles, metro cars and passenger coaches, a full line of freight wagons, such as covered wagons, open-top wagons for coal, ore, steel and timber, hopper wagons for grain, ore, fertilizer, flat wagons, double-deck container flat wagons, tank wagons for all types of liquid and chemicals, tipper wagons, schnabel and depressed center wagons, and track machinery.

Components

CRRC serve rail component market through its activities in the fields of diesel engine, motor, locomotive, coach, wagon, forgings and castings. Our expertise lies in designing and manufacturing a comprehensive range of components, while we maintaining good quality , reasonable price and quick turnarounds. We can provide a cost-effective and timely solution for mechanical or electrical, moulded, forged or finished, and metal or non-metal. From design to delivery, we maintain a close working relationship with every customer to assure that their performance needs are met, no matter the crankshaft is slated for a high performance locomotive or a hard working wheel-set performing critical duties. We adopt professional assistance with CAD/CAM to process a variety components suited to customer ’ s needs and peculiarities. If customers provide us with drawings, samples or materials, our engineering and product development department will then enhance the work to maximize quality and effectiveness.

R&D

CRRC employs a great number of technological talents with expertise in locomotives, rolling stocks and other products. The soul of CRRC enterprise is innovation in technology in the course of its development. CRRC effectively digests, absorbs and recreates imported technology, commits itself to mastering critical know-how and elevating its self-determination and originality,and CRRC is rapidly and significantly speeding up the heavy-load freights and maximizing the speed of passenger cars in China. CRRC has many sophisticated tools for calculation analysis and engineering testing. Based on the market demand and using cutting edge

and cost-effective technology, CRRC aims not only at offering the best but also the most valuable products and service to its customers. CRRC has always been internationally reputed for its high-quality. All production enterprises are the ISO 9001 certified,.and some have acquired certificates of ISO 14001, OHSAS 18001 and AAR. CRRC has strong ability in machining, casting, forging, steel fabricating, welding and assembling as well as in developing motor and electrical equipments. Highly competent employees, sound technical product examination and efficient management system are the guarantee of high quality products. The focus of CRRC is quality, which is the way we do business. Our policy is to strengthen the management to serve the market by supplying products conforming to international standards and/or standards of customers. Every product receives strict scrutiny from the first operation step to the last one. Operators, inspectors, supervisors, and managers ensure that the products our customers receive meet quality standards.

Other Products

CRRC has long devoted itself to industrial extension and concentric diversification, and has expanded its business to a wider scope of green and smart city development by achieving remarkable success in new industries, including wind power equipment, polymer composites, new energy buses, environmental protection, ship and marine engineering equipment, PV power generation, intelligent machinery, heavy engineering and mining machinery.

China Machinery Engineering Corporation (CMEC), a core subsidiary of SINOMACH, is among the world’s top 500 companies.

110.China Machinery Engineering Corporation (CMEC)

Founded in 1978, CMEC is China’s first engineering & trade company. Through over 40 years of development, CMEC has become an international corporation with engineering contracting and industrial development as its core divisions. It has been underpinned by a full industry chain of trade, design, survey, logistics, research and development. It has offered “one-stop” customized solutions for integrated regional development and various types of engineering projects, covering pre-planning, design, investment, financing, construction, operation and maintenance.

Regarding engineering construction, trade and services, CMEC has found its presence in 160-odd countries and regions. It has built batches of high-quality projects involving energy, water, environmental protection, agricultural cooperation, infrastructure, transportation facilities, industrial engineering, and communications in nearly 60 countries and regions including China, in particular along the Belt and Road routes.

Since 2014, President Xi Jinping has witnessed the signing or commencement of multiple CMEC overseas projects, including the Maldivian housing project, Sri Lanka Puttalam Coal-fired Power Plant, Serbia’s waste-to-electricity and wastewater treatment projects, and Argentina Belgrano Railway Reconstruction.

Development Strategy

Vision

A global leading EPC and integrated solution provider with innovation and value creation

Strategy Goal

Maintain CMEC's business scale and profitability while accelerating Strategic Business Group collaboration and improvement. Expand the company's breadth through exploring and developing new business opportunities. Build meaningful, effective relationships with high-performing enterprise partners both within China and abroad. Leverage CMEC's core management mechanisms as the foundation for the firm's globalization and continued expansion.

Business Portfolio

Build a multi-dimensional business with EPC at its core; trading and investments as transformative businesses with high value; engineering and technical consulting, real-estate development & services, regional ecosystem development, and integrated agriculture development as innovative businesses.

Global Footprint

Establish five Core Business Centers in China: Beijing Headquarters serves as CMEC's center of operations, strategy, and governance. East China Center serves as center of regional business. Guangdong and Hong Kong Centers are dedicated for financial and investment. Midwest China Center houses research and development. In addition to China, several overseas regional business centers will be opened to enable global operations and footprint.

Core Competency

Reinforce CMEC's core competency of providing end-to-end expertise and innovative management in developing business in feasible markets. Accelerate enhancement of capability and resource integration through value chain and collaboration across CMEC's business groups.

Core Management Mechanisms

EPC business management, legal, finance, investment management, along with corporate culture, and Corporate Social Responsibility make up the key values of CMEC.

Inorganic Growth

Driven by CMEC's corporate growth strategy, desire to add and improve core competency along value chain, and to meet business development demand, the company will pursue opportunities for rapid and sustainable growth through cooperation and strategic alliances with leading enterprises and M&A activity.

Foundation for Strategic Success

Leverage CMEC's strong foundation in effective organizational governance, human resources, risk management, IT, corporate culture, and branding to facilitate successful implementation of business strategies.

Corporate Certificates

Qualification for engineering tendering agencies(Grade A) Specialties: thermal power and other(new energy)

Qualifications for Project Supervision Grade A qualification for building construction project supervision and municipal utilities project supervision

Class A Certification of Comprehensive Engineering Investigation (by MOHURD)

Qualifications for Engineering Design

Certificate of Project Tendering Agency of China, issued by the Ministry of Housing and

Urban-Rural Development of China to CMEC
Our business
Engineering Contracting

CMEC is an internationally renowned provider of integrated engineering contracting services.
1.overseas Market
Since the beginning of the 1980s, CMEC has been specialized in international engineering contracting, representing one of China's first teams "going global". Currently, CMEC has an impressive track record of projects in 60-odd countries and regions across the world, especially along the "Belt and Road" routes. Since 2014, President Xi Jinping has witnessed the signing or commencement of multiple important CMEC projects.
2.Domestic Market
CMEC boasts development, design and construction projects in the key areas of national coordinated regional development, including Beijing-Tianjin-Hebei, the Guangdong-Hong Kong-Macao Greater Bay Area, the Yangtze River Delta, the Yangtze River Economic Belt, the Yellow River Basin, the Chengdu-Chongqing region and the Hainan Free Trade Zone. It has continued to engage itself in the integrated regional development and park development and construction in domestic cities. The company has also built six CMEC plazas in Beijing, Shenzhen, Changsha, Shanghai and Xi' an. They are home to high-tech industrial parks in the cities.
3.Business Area
Focusing on energy engineering, CMEC has specialized in eight important industries of national economy and people' s livelihood in domestic and international markets.
4.One-Stop Service
CMEC has provided "one-stop" customized solutions for integrated regional development and various types of engineering projects, covering pre-planning, design, investment, financing, construction, operation and maintenance.
5.Luban Award
In 2013, 2015 and 2021, Turkey EREN Power Plant Project, Belarus Berezov Power Station and Angola SOYO Power Plant, undertaken by CMEC, won the Luban Award (Overseas Project), China' s highest award for construction projects.
Trade and Services
CMEC boasts a trade network covering 160-plus countries and regions across the world. By giving full play to its unique role of bridging two domestic and international markets and two types of resources, CMEC has big strides in technology manufacturing, international business corporation, system synergy, new energy, bonded processing park and bidding and tendering services. It has produced a series of internationally leading high-end castings and forgings. It has created multiple new models for international trade and supply chain, including Yinchuan Silk Road International Cooperation Park, the "Internet plus" and overseas warehouses.

1 Technology Manufacturing
CMIPC serves as CMEC' s core subsidiary specializing in high-end equipment manufacturing and R&D business. CMIPC, coupled with CMPM and CMHC, has operated technology manufacturing in an integrated manner. The division focuses on manufacturing high-end cast and forged parts,

copper pieces, welded structural components and finish machining parts. It has offered one-stop value-added services and complete solutions for the whole industrial chain ranging from design & development, production, quality control, logistics and after-sales services to well-known customers in China and beyond. The products have been widely applied in mining equipment, construction machinery, electric power, metallurgy, oil & gas, shipbuilding, and transportation.

2 System Synergy

CMEC Engineering Machinery Import & Export Co., Ltd. represents one of CMEC ’ s core subsidiaries in terms of system integration and coordination, with Suzhou Company and General Machinery Company brought under CMEC ’ s control. As such, CMEC has realized integrated operation. Focusing on import & export trade as its main business, it has established an integrated operation platform covering Southeast Asia, Central and South America, North America and European markets. The platform has combined a range of strengths including power equipment, machinery manufacturing, complete industrialized projects, new energy engineering, e-commerce operation, technical consulting services and capital operation.

3 International Business Corporation

CMEC has built an international business corporation with China-East Resources Import & Export Co. at its core, achieving its strategic transformation towards an international integrator of engineering, trade and services. Under the guidance of diversified development strategy, the international business corporation has developed four major business divisions, trade, engineering contracting, labour export and investment.

4 New Energy

With CMEC Wuxi as its core subsidiary for new energy and business innovation, CMEC, in conjunction with Shanghai Zhongjing managed by CMEC and Wuxi Shimaotong, Wuxi Taichang and Wuxi Taichen controlled by CMEC, has realized integrated operation. It has fully relied on the economic advantage of the hinterland in East China and the regional strength in the Central China, with its business involving new energy, traditional trade and supply chain services.

5 Bonded Processing Park Services

With CMEC Yinchuan as the core, CMEC has conducted integrated operation with CMEC Henan. It has developed the business model of “Park plus Trade” by building a variety of platforms for bonded processing, domestic engineering cooperation and international engineering cooperation.

6 Bidding & Tendering Services

CMEC has a specialized platform for bidding services, consisting of CMEC Bidding Division and the Tendering Company. The bidding business involves more than a dozen fields such as machinery and electric power. Relevant projects are funded by loans from the World Bank loans, the Asian Development Bank and foreign governments, and by national bonds, technological upgrading funds and government budget funds from China. Bidding methods include open bidding at home and internationally, invitation to bid, competitive negotiations, inquiry & procurement, and direct procurement. Bidding contents include goods, engineering, services, and BOT.

Design and Consultation
Design & consulting is also one of our main businesses. We possess more than 40 Class-A design qualification certificates, which include engineering consulting, urban and rural planning, machinery, building, municipal facility, military, metallurgy, textile, landscape design, environmental engineering, engineering investigation and mapping, geological hazard control, geotechnical design and construction, engineering contracting, etc.

Our two wholly-owned subsidiaries in this sector, China Machinery International Engineering Design & Research Institutes Co., Ltd.(CMIE) and China JIKAN Research Institute of Engineering Investigations and Design, Co., Ltd.(JK Institute) , are among the earliest large comprehensive design and consulting institutes established in China. We have over 2100 professionals in more than 30 professions. Among them, there are National Masters in Engineering Investigation and Design, APEC Architects, International Project Management Professional (IPMP) managers, professors of engineering, senior technicians and national registered engineers.

Our design and consulting team has provided services around more than 50 countries and regions globally and 31 provinces and regions nationally. Besides the conventional areas, our research and development achievements also enable us to engage as EPC contractor in new technique projects and markets, such as "Sponge City", Utility tunnel (City's underground pipelines), smart plant, environmental protection and new energy projects, etc.
Since established, We have undertaken more than 38,000 design and consulting projects. We have achieved nearly 500 scientific research achievements, as long as more than 400 national and provincial science and technology progress awards, and over 100 national patents and proprietary technologies. We are also chief editor or co-editor of more than 80 national technology standards and specifications.
Investment and Assets Operation
CMEC has upgraded and implemented the integrated model of “investment, financing, construction and operation”, and provided new underpinnings for its sustainable development through investment in and operation of high-quality business.
Since 2014, CMEC has developed a series of coal mines and power plants in Thar, Pakistan, with the Thar Programme launched. The total investment in the programme exceeds USD200 million, while the EPC contract amount is about USD2 billion. The programme has become a typical project of CMEC’s “investment-led EPC” and “integration of investment, financing, construction and operation”.
The Thar programme, a priority project for the China-Pakistan Economic Corridor along the Belt and Road, is of high political significance as the signing was witnessed top leaders of both countries. CMEC has creatively integrated the financial resources of China and Pakistan in advancing the project and completed its financing closure in less than a year.
Up to now, the Thar phase-I project has been up and running, while the phase-II is scheduled to be completed in 2022. The Thar Programme has been successfully implemented, setting an exemplar for local energy development in Pakistan. It has provided new ideas for Pakistan’s energy security, contributing to the successful construction of the China-Pakistan Economic Corridor.
Responsibility Management

Fulfilling social responsibility has become an important path for enterprises to contribute to global economy’s sustainable development and a significant strategy to adapt to the new normal of economic development and improve their corporate competitiveness. CMEC strengthens its social responsibility management, pays special attention to the construction of four major systems including social responsibility management system, social responsibility practice system, social responsibility communication system and social responsibility dissemination system. By these efforts, the company continues to integrate the concept and demand of social responsibility into its policies and daily business operation, hence constantly improving its sustainable capacity.

CMEC is devoted to promoting the realization of the sustainable development goals (SDGs) of the United Nations. With the correct idea of moral and profit at the core, leveraging the industry characteristics, its own advantages and responsibility practice, and joining hands with interested parties such as shareholders, investors, employees, government, customers, industries and partners, it pursues the maximization of the comprehensive value of the economic and social environments, cultivates the responsibility culture, improves the social responsibility work system, strengthens the responsibility communication, and provides innovative solutions for the sustainable development issues such as economy, society and environment, in order to achieve the mutual development and all-win harmony with all interested parties, and help the country and the world realize the sustainable development.

CSR Action

Value CMEC

As the competition in international contract project market is becoming increasingly intensified and the society is paying more attention to the comprehensive influence of enterprises’ operation, ...

Improve Value Creation

CMEC adheres to the corporate culture of “diversity, inclusiveness and innovation”, deepens the transformation and upgrading of core businesses of complete projects, trade and services, investment and financing. Efforts have also been made in building design and consulting segments, promoting resource integration capability, and creating more values for investors.

Build Responsible Value Chains Together

CMEC pays attention to its leading role in the supply chains, insists on carrying out responsible procurement, makes regulations and takes measures to do strict qualification examinations towards suppliers and sub-contractors, by which it tries to avoid liability risks at the maximum level and jointly builds a responsible value chain.

Pursue Win-win Cooperation

CMEC stick to the philosophy of achieving win-win cooperation, cherish the cooperation with our stakeholders, and aim to realize responsible growth and development with our partners. CMEC highly values its partnerships from the strategic height. CMEC actively expands its external cooperation mode, conducts mutually beneficial cooperation with governments, banks, enterprises and international agencies, and strengthens communication and mutual trust, especially its cooperation with global leaders and key strategic partners, to promote the industrial competence and broaden business development for common development.

Quality CMEC

Customers are the basis for the existence and development of enterprises, recognition and trust of customers are the source of our values. CMEC has always put clients interests first, and in pursui...

Building High-quality Projects

In CMEC, the quality of projects always comes first. CMEC provides high-class projects and services to its customers wholeheartedly, makes quality engineering projects, and strives to create excellent projects to create long-term and sustainable project values for its customers.

Customer Services Satisfaction

CMEC attaches great importance to the operation and maintenance of its projects, strives to transform from an engineering contractor into a comprehensive services provider and offers package solutions to its proprietors, including training for the operation and maintenance of the power plants and preservation of an operation and maintenance team to provide services and guidance for its proprietors. With these, it aims to maintain a stable operation of the power plants after project delivery and to create more values in each step.

We concern, tap and respond to customer demands, and provide services in the whole process from contract signing, plan formulation, designing, procurement, manufacturing, storage and transportation, construction, installment to training, test running and after-sales services, etc. to our customers. We work hard to provide high-quality services beyond their expectation to satisfy them.

Ping CMEC

CMEC has always been operating on the basis of work safety, sticking to the principle of "Safety first with precaution and comprehensive treatment", continuously improving the management system of ...

Enhance Work Safety

CMEC comply strictly with red line of work safety, tighten safety supervision of domestic and overseas projects, and have introduced the work safety responsibility system and organized related themed activities. In this way, we have been able to improve the safety ability and awareness of the Company and guarantee the work safety in the long run.

Protect Occupational Health

CMEC attaches great importance to the occupational health of overseas employees and constantly improves the occupational health management system and health safety service. CMEC offers employees with regular physical examination and disease prevention training, and dispatches medical teams to overseas projects in order to truly guarantee the health and safety of overseas employees.

Green CMEC

As an enterprise with businesses all around the world, we always pay attention to environmental protection whereever we go. We incorporate the concept of environmental protection into the entire production.

Implement Green Operation

In its global operations, CMEC has always been giving special attention to environmental protection during the projects construction and actively practicing green construction. It has established a systematic and informationized energy conservation and emission reduction

management system, and introduced new energy resources and materials to minimize the negative impact on the environment.

Promote Energy Saving and Emission Reduction

CMEC continues to promote green operations, energy saving and environmental protection, striving to raise the awareness of its employees. It also advocates green offices and low carbon lives and organizes environmental protection campaigns, thus creating a united atmosphere devoted to environmental protection.

Devoted to Clean Energy

According to the concept of green development, CMEC has long been focusing on and devoted to the development and application of clean energy. We actively build green projects such as solar power plant in order to contribute to the construction of a beautiful China and dealing with global climate change.

Happy CMEC

CMEC attaches importance to the leading role of talents towards corporate development, always sticks to the people-oriented philosophy. The Company builds good development platforms for its employee.

Build Harmonious Working Environment

CMEC strictly abides by national laws and regulations to conduct employment management. It provides a competitive salary and other welfare and receives employees’ expectations and appeals to effectively guarantee the legal rights and interests of them and build harmonious and stable labor relations.

Establish Developmental platforms for Employees

CMEC carries out the strategy of “reinvigorating enterprise through human resource development,” attaches great importance to the growth and development of employees, tries to build a scientific and reasonable talent cultivating mechanism and dedicates itself to build a solid and broad stage for the career development of employees.

Create Happy Life

CMEC concerns the happiness of employees. It cares for the life of overseas employees and their families as well as the retirees, helps employees with financial difficulties and attaches importance to the balance between work and life for employees. Besides, it also organizes various kinds of cultural and recreational activities to continuously enhance employees’ sense of happiness and strengthen corporate inclusiveness in order to build a happy CMEC family.

Harmonious CMEC

CMEC adheres to the concept of “Taking Root in Society and Rewarding Society”. We actively fulfills global corporate citizenship responsibility. By carrying out localized operation based on the e...

Implement Localized Operation

CMEC continuously implements localized operation, respects local traditions and customs and makes greater efforts to develop overseas organizations to create more job opportunities. We give priority to using local resources and are committed to building a “community of shared destiny" with the regions in which we operate so as to strengthen the tie of our mutual benefits.

Improve Community Livelihood

With its advantages in project contracting, CMEC actively supports local infrastructure construction such as medical service, housing, power and facilities in an effort to meet the most pressing need of local residents, and continuously improve the local community environment.

Engage in Public Welfare

For years, CMEC has been continuously contributing to public welfare. We innovate the way of public welfare, actively engage in public welfare and organize employee volunteer activities to offer care and support to those in need, and facilitate poverty alleviation as well as scientific and educational development.

Our Culture

Corporate Image

Vision

A leading international engineering contractor and integrated service provider for industrial development

Philosophy

Create Ideas, Achieve Dreams

Enterprise Spirit

Integrity, Pragmatism and Craftsmanship

Corporate Culture

Being diversified, inclusive and innovative

111.Shandong Weiqiao Pioneering Group.,Ltd

Shandong Weiqiao Pioneering Group Company Limited is located at the southern end of Lubei plain, closely adjoining Jinan airport, Qingdao port, Qingdao-Jinan Railway, Jinan-Qingdao Expressway, and on the brink of the Yellow River. With 12 production bases, the company is a super-large enterprise which integrates spinning and weaving, dyeing and finishing, garment and home textile and thermal power. Since 2012, the company has been listed in the World’s Top 500 for 10 years. The “Weiqiao” brand was selected into the ranking list of the 500 Most Valuable Brands in China for 18 consecutive years. It has been selected as one of the world's top 500 brands for two consecutive years.Hu Jintao, Wu Bangguo, Wen Jiabao, Yu Zhengsheng, Wu Guanzheng, Zhang Gaoli, Chi Haotian, Liu Yandong, Li Jianguo and other leaders respectively visited the group and fully affirmed and highly appraised the development performance of the enterprise.

Weiqiao Pioneering Group has been awarded ‘National May 1 labor medal’ in 1997, ‘National advanced unit in building of spiritual civilization’ in 1999, ‘National advanced Party organization at primary level’ in 2006, ‘Home for national working models’ in 2010, ‘National advanced group of textile industry system’ for many times, national advanced unit of textile harmonious enterprise construction in 2006, Chinese ten brand cultural textile enterprise in 2010, national textile industry industrialized & informationized fusion model enterprise in 2011, and national textile industry advanced party construction demonstration enterprise in 2013.

COMPANY CULTURE

Enterprise spirit

Fighting, striving, innovation and high efficiency.

Work creed

Think only for success, not for failure

Enterprise Production and sales policy

Sale is based on the market; Production is based on sale; Supply is based on production; Plans are based on benefits.

Production management policy

People-oriented, realistic and innovative, worsted weaving, customer satisfaction.

Development concept

We should not only do what we can, but also do our best; we should improve in development and develop in improvement

quality standard

One -vote veto, two prohibitions, and three never miss.

Enterprise management policy

Strict management and tight production. Good quality, flexible operation and care for life

Core values

Start a business for the country and benefit the people

ENTERPRISE HONOR

Energy saving and emission reduction innovative textile enterprise

National Textile Industry Quality Award

National textile industry implements excellent performance model

China's Top 500 Manufacturing Industries in 2006

DEVELOPMENT PATH

In 2019, the group became one of the world's top 500 brands. With an investment of 820 million Yuan, the green and intelligent textile integration project was completed and put into operation. Weiqiao-UCAS Research Academy was officially launched.

BRAND INTRODUCTION

Shandong Weiqiao Pioneering Group Company Limited is located at the southern end of Lubei plain, closely adjoining Jinan airport, Qingdao port, Qingdao-Jinan Railway, Jinan-Qingdao Expressway, and on the brink of the Yellow River. With 11 production bases, the company is a super-large enterprise which integrates spinning and weaving, dyeing and finishing, garment and home textile and thermal power. Since 2012, the company has been listed in the World’s Top 500 for 8 years. The “Weiqiao” brand was selected into the ranking list of the 500 Most Valuable Brands in China for 16 years in succession. Hu Jintao, Wu Bangguo, Wen Jiabao, Yu Zhengsheng, Wu Guanzheng, Zhang Gaoli, Chi Haotian, Liu Yandong, Li Jianguo and other leaders respectively visited the Group and fully affirmed and highly appraised the development performance of the enterprise.

Responsibility report

The group has always been firmly committed to the belief of "doing business for the country and benefiting the people", and has taken driving the local economic development and building a harmonious society as the driving force and self-conscious pursuit of the enterprise. It has played some roles in solving the problems of "agriculture, rural areas and farmers" and promoting urbanization construction. The first is to strive to increase national and local fiscal revenues. It has always paid taxes strictly in accordance with the law and has paid more than 90 billion Yuan in taxes for the country. The second is to expand the employment of farmers and promote urbanization. Currently, 95% of the group's 100,000 employees are from rural areas, and 95,000 rural surplus labors have achieved employment. The third is to promote the "Project of Living and

Working in Peace and Prosperity". Provide affordable housing for employees at cost prices. At present, more than 50,000 households have been built to live in, which has solved the problem of left-behind children and left-behind elderly after farmers turn into industrial workers. There are outpatient hospitals near each industrial park, allowing employees to enjoy simple medical services at a cost price nearby. It has built 10 provincial-level standardized high-standard kindergartens for the children of employees, and invested in the construction of local primary and secondary schools to facilitate the children of employees to enter the kindergartens nearby; in order to effectively solve the worries of employees, the group buys social insurance for the employees.

Environmental governance

The group attaches great importance to environmental protection work and consciously strengthens environmental protection governance. In recent years, it has invested 10 billion Yuan to strengthen environmental protection governance and has taken the lead in the industry to achieve ultra-low emissions of thermal power units, alumina, and electrolytic aluminum. After the ultra-low emission of thermal power generators, the pollutant sulfur dioxide, nitrogen oxides, and soot emission concentrations are respectively about 15 mg/m3, 25 mg/m3, and 1 mg/m3, which are far better than the emission standards of natural gas generator sets of 35 mg/m3 of sulfur dioxide, nitrogen oxides 50 mg/m3, and soot 5 mg/m3. After the ultra-low emission of electrolytic aluminum, the particulate matter is 2 mg/m3 and sulfur dioxide is 10 mg/m3, far better than the 20 mg/m3 particulate matter and 200 mg/m3 required by the "Aluminum Industry Pollutant Emission Standard" The requirements of the company are in the leading international level.

WELFARE

While driving local economic development, the group company is enthusiastic about public welfare and charity, actively donating to disaster-stricken areas, poverty-stricken areas, extremely poor employees, and disadvantaged groups across the country, and vigorously supporting local public welfare undertakings such as transportation, education, and health. An excellent corporate citizen image of the society.

After the Wenchuan earthquake in Sichuan, it donated 13.5 million Yuan of money and materials to the people in the disaster area.

The group is cooperated with Beijing Rainbow House Charity Organization to form 19 Weiqiao Rainbow Houses.

During the rampant period of the Ebola epidemic in 2015, we invested $1 million to build two medical stations.

For the COVID-19 epidemic, the company immediately donated 30 million Yuan for epidemic prevention and control.

Human resources

TALENT STRATEGY

Weiqiao Pioneering Group attaches great importance to the construction of talent team, and strives to build an outstanding talent team with "loyal enterprise, strong style, strong innovation and technical reform ability", centering on the realization of the vision goal of "keeping the leading edge and striving to be the industry pioneer". Speed up the introduction of high-end talents, do a good job in selection and retention, and enhance the core competitiveness of enterprises.

The group has always followed the people-oriented management philosophy, regarded the construction of the talent team as the first driving force of the group's development, highlighted the leadership, innovative talent, skilled talents and team building, worked hard to improve the level of talent team building, and made every effort to build a team that was in line with the strategic development of the group.

The Group has always paid attention to the growth of employees, and established management personnel selection and incentive mechanisms in accordance with the principle of “the capable ones are promoted, and the mediocre ones are eliminated” . We adopt many ways of training, such as teachers and apprentices, on-the-job training, vocational skills competition, special training, academic education promotion, independent evaluation of skilled talents, etc. to continuously unblock career development channels, to help employees achieve career development goals, provide employees with opportunities and platforms to realize their self-worth, and achieve harmonious development between employees and the company.

EDUCATION TRAINING

Skilled personnel training

The group attaches great importance to the training of skilled personnel, gives full play to the role of skilled personnel in "passing, helping and leading", carries out annual technical competition activities, grandly commends skilled personnel, and relies on the company's independent evaluation of qualifications to do a good job of skill level assessment, forming a relatively complete skilled talent evaluation system, plans to increase 3,000 skilled talents within 5 years, so that the skill level of skilled personnel will be improved as a whole.

Management training

By organizing high-level executives to participate in high-end entrepreneurial forums, summits, annual meetings, visits to successful companies, and attend high-end lectures, help them develop strategic thinking, improve business philosophy, improve scientific decision-making capabilities and management capabilities, and comprehensively improve management level.

New employee training

The group also focuses on training newly recruited employees on company culture, national laws and regulations, labor discipline, safety production, craftsmanship, quality awareness, political and ideological education, etc., and guide employees to integrate into the company as soon as possible and adapt to the company's work and living environment.

112.Wynca Group

A-share listed company under Transfar Group

Wynca Group was founded in 1965 and came on the market in 2001, possessing 76 holding subsidiaries and more than 6,300 employees.

Wynca is mainly engaged in three industries: crop protection, silicon-based new materials and new energy materials. The crop protection industry has formed an integrated development model of "intermediate - original drug - preparation", covering seeds and seedlings, herbicides, insecticides, fungicides, crop nutrition and other products, providing comprehensive solutions to ensure food security and help increase yields and incomes. Silicon-based new materials have a complete industrial chain from mining and smelting upstream silicon, organic silicon monomer synthesis and manufacturing downstream products, which are widely used in electric power communication, rail transportation and automobile, medical and health care, consumer electronics and other fields in more than 130 countries and regions worldwide. Based on the

strengths of the phosphorus-based and silicon-based industries, New Energy Materials has built comprehensive material solutions around new energy application scenarios, building a new "three-legged" development pattern with the other two industries.

Global top 20 agrochemical sales, China's leading enterprise in fluorosilicon industry, China's top 500 manufacturing industry, China's top 500 chemical industry National Innovative Enterprise, National High-tech Enterprise, National Intelligent Manufacturing Pilot Demonstration Enterprise, National Intellectual Property Demonstration Enterprise

National Advanced Enterprise in Comprehensive Utilization of Resources, China Green Factory in Petroleum and Chemical Industry, China Top 100 Private Enterprises in Social Responsibility, Advanced Private Enterprise in Combating Epidemic by the all-China Federation of Industry and Commerce

Chairman's Speech

Plodding its way from Baisha Bank, Wynca croup has endured more than half a century of baptism of reform a nomarket economy, witnessing the difficult years of development and growth of Chinese enterprises, and has made its own growth both in scale and strength.

A drop of water can see the wild waves of the sea, a period of years concentrates the struggle spirit, and the spirit can inspire a glorious future. We are delighted and proud of such a journey, which is the result of the wisdom and hard work of all Wynca people and our persistent pursuit of Wynca's cause.

We see hope in times of difficulty, knowing how to persevere in times of adversity, and being prepared for dangeiin times of prosperity. We do not move blindly, we do not retreat. We persevere our will and we act with determination. We will create a brighter future with contributions that are worthy of our forefathers, our mission and our times.

The power of perseverance is evident in every step of Wynca Group's development.

Because of our persistence, we are culture-led, pursuing excellence. creating value while adhering to customer.first, contribution-based, hard-working and sharing

Because of our persistence, we are proud and innovation-driven, committed to becoming a global leader in the field of silicon-based and phosphorus-based new materials and crop protection solutions.

Environmental friendly chemicals create a better life!

Wynca Culture

Our Mission

Environmental friendly chemicals create a better life.

Our Vision

Be a global leader in siliconphosphorus-based new materials and crop protection solutions.

Core Values

Customer prior, Contribution-oriented, Hard struggle, Cocreating and sharing together.

Our Spirit

Stay exploring,Keep innovating

Sustainable Development

Research and development team

Scientific and technological honors

scientific and technological talents1000+

Masters and PhDs200+
high-level talents of all kinds300+
1,000+ scientific and technological talents, 200+ masters and PhDs, 300+ high-level talents of all kinds. There are 1 national candidate of the New Century Talents Project, 4 people enjoying the special allowance of the State Council, 3 young and middle-aged experts with outstanding contribution in Zhejiang Province, 1 young top talent of the 10,000 people plan in Zhejiang Province, 13 candidates of the "151" talent project in Zhejiang Province... Zhejiang Provincial R&D Team

Nearly 20 famous research institutes and enterprises at home and abroad, such as Tsinghua University, Zhejiang University, Russian National Element Institute and Dow, have carried out various forms and rich technical cooperation and exchanges with Wynca.
Digital Wynca
Digital economy is the new driving force of enterprise, and digital transformation is the road one must take for high-quality enterprise development.
In 2016, Wynca drew a blueprint for digital development and invested over 2 billion yuan to build Mamu Intelligent Park with high standard, thus opening its own digital reform road, starting to take big data, industrial cloud, artificial intelligence and other new generation information technology as the driver, " Four Integration" concept as the core, to promote the high-quality development of Wynca, to create an efficient and intelligent production and operation mode integrating product development, manufacturing, sales and service.
The sword sharpens from sharpening. Wynca's digital transformation path is recognized by governments at all levels and all walks of life, becoming a typical representative of traditional manufacturing industries at provincial and municipal levels to successfully practice digital transformation.
In 2021, Hangzhou Chain Master Factory Cultivation Enterprise
"Provincial Industrial Internet Platform Project" in Zhejiang Province
"Smart Manufacturing Pilot Demonstration Project" by National Ministry of Industry and Information Technology
Win-win cooperation
Adhering to the "customer-first" idea, we take customers as the center of everything, making customers' needs as the starting point and end point of all value creation, continuously creating value and providing services for customers, and establish strategic partnership with common interests.
Social responsibility
All accidents can be prevented.

We have introduced DuPont SHE management system, established a three-level emergency rescue network of "company-park-factory" and professional emergency rescue team, realized 100% coverage of safety information in the park through SHE intelligent and visualized management, and carried out SHE system audit for all production units under our company every year to futher consolidate the main responsibility of safety production.

We have won the first prize of provincial and municipal emergency skills competition for many

times.
Never let a drop of sewage flow into the river.
Adhering to green development, resource-saving development, environment-friendly development and social beneficial development, Wynca Group makes full use of new technologies, new materials, new processes and new equipment to accelerate energy-saving and low-carbon transformation, form the synergistic effect of pollution reduction and carbon reduction, and continuously deepen the three-element circular economy model of chlorine, phosphorus and silicon, brightening the ecological background of high- quality development.
Spiritual civilization
Being responsibility-based, we endeavor to steer the carrier Wynca into the flood of outstanding enterprises of the times, to be responsible to shareholders and society, and to establish a mutually beneficial and win-win community.
Contribute to winning the battle against poverty by donating 20 million RMB in three years to build 100 "Xin'an - Anxin" health rooms in Guizhou and Yunnan to improve the medical service level of poor villages.
Wynca adheres to the mission of "Environmental friendly chemicals create a better life!"
Insisting on the development path of resource-efficient, environment-friendly and socially-efficient. Wynca has pioneered the recycling technology of chlorine, silicon and phosphorus, and become the pioneer of circular economy model within the industry.

113.COFCO
Founded in 1949, COFCO Corporation (COFCO) is the leader of the Chinese agricultural industry, and one of the world's leading agri-businesses with global footprints and a fully-integrated value chain.

COFCO’s takes agri-products as our core business, involving grain, oil, sugar, cotton, meat, dairy products, etc., and also features food, finance, and real estate as three major complementary business segments.

As of the end of 2023, COFCO boasted total assets of RMB 730.7 billion. In 2023, COFCO registered an overall operating income of RMB 692.1 billion and total profits of RMB 21.2 billion.

COFCO continuously improves our agri-business landscape, enhances our operations in agriculture commodities, and strengthens integration of origination, storage, processing, logistics, and trade across the industry chain, to safeguard the G & O supply efficiently in a market-oriented way.

COFCO has been actively going global with bigger strides, by continuously improving G & O logistics and storage capacity worldwide to ensure a stable global supply chain. COFCO has now established a global agriculture trade and logistics network covering major production and sales regions, where we engage in the origination, storage, processing, logistics, and trade of agricultural commodities, such as grains, oils & oilseeds, sugar, meat, and cotton. COFCO has set up a stable corridor between the major grain producing regions, such as South America and the Black Sea, and emerging Asian markets. Currently, over 50% of COFCO’s revenue comes from overseas, with our annual global overturn of agriculture commodity more than doubling China’s annual total imports.

In China, COFCO is the major importer and exporter of soybean, wheat, corn, sugar, and other agricultural products. COFCO meets Chinese consumers’ daily needs of agricultural products. In China, COFCO is also among the leaders of the oil crushing industry and in processing, trade, and selling of rice, flour, and ingredients of beer, one of the top cotton traders, a large-scale technological leader in deep processing of corn, as well as the leading meat and dairy product supplier with a fully-integrated value chain.

COFCO produces quality food and creates outstanding brands. COFCO’s well-known brands, including Fortune, Greatwall, Chinatea, Joycome, Jiugui, and Lohas, are available in more than 90% of China’s prefecture-level cities. COFCO is also the cradle of the first bottle of dry red wine and dry white wine, as well as the first stated-owned tea company of the People’s Republic of China. COFCO is the bottling and packaging partner with Coca-Cola and other world-renowned food and beverage brands. COFCO is the host of the enormous Food and Drinks Fair that attracts visitors from around China, and a professional E-Commerce platform for food products, enriching people’s life.

Based on the agriculture and food industry chain, COFCO provides financial support tailored for agricultural development, develops financial business chains such as trust, futures, insurance, and fund to integrate industry and finance, and serve the agriculture, rural areas and farmers.

COFCO facilitates urban upgrades and service improvement with its business covering shopping malls, residential buildings, industrial parks, hotels, office buildings, and other fields. Among them, the commercial real estate brand “Joy City” shopping mall leads China’s new department store formats in more than 10 first-tier cities.

COFCO has been steadily promoting the reform as a state-owned capital investment company and innovating market-oriented mechanisms, established 17 specialized subsidiaries based on their core products, namely: COFCO International, COFCO Trading, COFCO Grains & Cereals, COFCO Oils & Oilseeds, COFCO Biotechnology, COFCO Sugar, Chinatex, COFCO Technology & Industry, COFCO Wines & Spirits, COFCO Coca-Cola, COFCO Joycome, China Tea, Mengniu Dairy, Womai.COM, CPMC, COFCO Capital, and Grandjoy Holdings.

As an investment holding company, COFCO owns 15 listed companies, with 8 listed in Hong Kong, namely China Foods (00506.HK), CPMC Holdings (00906.HK), Mengniu Dairy (02319. HK), Joy City Property Ltd. (00207.HK), COFCO Joycome Foods Limited (01610.HK), Fountain Set (00420. HK),China Modern Dairy (01117.HK), China Shengmu Organic Milk(01432.HK); and with 7 listed in Mainland China: COFCO Sugar (600737.SH), Miao Ke Lan Duo(600882.SH), COFCO Biotechnology (000930.SZ), Grandjoy Holdings (000031.SZ), Jiugui Liquor Co. Ltd. (000799.SZ), COFCO Capital (002423.SZ) and COFCO ET (301058.SZ).

In the future, COFCO will continue to focus on our core businesses, promote quality development, boost agricultural and rural modernization, and speed up on our way to the world’s leading agri-business.

114.Honghua

As one of the leading land drilling equipment manufacturers in the world and the largest land drilling rig exporter in PRC, Honghua is primarily engaged in manufacturing conventional land drilling rigs, digital drilling rigs, accessories of drilling rigs, as well as the parts and components for the drilling rigs or for the maintenance of the drilling rigs in operation. Leveraging on the strong R&D strength, high-quality production facilities and mature international sales network, Honghua's 80% products have been sold to a large number of famous enterprises all over the world, including major oil-production regions such as North America, Middle East, and emerging markets including South America, India, Russia and Africa. In the future, Honghua will deeply engage in advanced energy equipment manufacturing and oil & gas field services field and achieve synergy in R&D, project execution and market expansion, to become an international leading combining equipment manufacturing provider in oil & gas industry.

CSR

Throughout the years, Honghua has always upheld its corporate social responsibility principles of “Gratitude, Love, Education and Enlightenment” as well as its value of “Contribution to the society.” We continue to actively take part in promoting workplace safety, environmental protection, talent cultivation, employee benefits, and social welfare. We take great consideration of these values during our corporate management and decision making processes. We aim to

promote a harmonious relationship between the Company and the society and between the economic development and the environment with a view to guarantee the interests of our Shareholders, partners and employees. And we also aim to contribute to the sustainable development of global energy.

Products & Services

Energy Equipments Manufacture

Energy Services

Marketing

HH Group has established subsidiaries and offices in the USA, Russia, Ukraine, Egypt, the UAE, Venezuela, Bolivia, Colombia, India, Indonesia, Pakistan, Kuwait, Iraq, Poland, Azerbaijan, Kazakhstan, Uzbekistan, Brazil, Mexico, Algeria and China.

In recent years, nearly 100 sets of HH drilling rigs were sold to all oil fields domestically and internationally every year, 80%were sold abroad.

Depending on the excellent quality, competitive price and satisfying after-service, self-made DBS series electric rig, arcticrig, fast-moving rig, HH was awarded acceptance and appreciation by global clients. HH rigs are widely used in more than thirty countries and regions such as United States, Russia, Middle East, Central Asia and Africa etc, which take up great sales share of global land rig market and promote the upgrading of global land rigs.

Currently, almost 900 sets of HH drilling rigs are serving around the world.

After Sale Service

HH owns a 200-staff professional service team for rig installation, commissioning and solution of problems arising during on-site operation. After-sale service offices have been set up in America, Egypt, U.A.E., Venezuela, Russia and China.

HH Commitment: Respond within 4 hours after receiving customer feedback and give an accurate and complete answer within 48 hours. For the problem needing to be solved on site, our technicians will arrive at the site as soon as possible.

Tech. Training

HH has DBS drilling rig training base including one set of advanced DBS training rig, hydraulic system teaching simulation operation platform, training classroom and other hardware facilities and professional training materials. It has provided training service for thousands of customer engineers from the Russia, the United States, Middle East and so on to make them capable in the fields of mechanical, electrical, drilling engineering and other aspects.

Qualifications

HSE certification, API certification, integrator authentication for ABB drive system and explosion proof certification forrelated electrical products, etc.

HH can provide clients with equipment that meet local special specifications, like GOST standard in Russia.

115.Le Bunna

Focus Makes Expertise

One-stop Solutions for Ethiopian Coffee International Supply Chain

Our company is dedicated in single coffee origin - Ethiopia, the birthplace of Coffee Arabica for since 2014.

During the 8 years, we deeply invested in the local coffee industry chain from variety research,

coffee processing, international trading as well as marketing, especially in the rising China market.
Focus makes expertise and Lebunna your reliable Ethiopian coffee Partner.
Social Responsibility
We do more than business
Farmers
Value-added working skills training
Women & children caring
At the forefront of coffee value chain, coffee farmers play key role in improve coffee qualities with better wefull red cherries and standardized processing methods.We would like to share our coffee picking skills and processing standards with local farmers for higher coffee qualities, so we could share higher added value from the market.Also, special caring is provided for women and children in our partners' farms and washing station
Researches
Coffee Varieties
New_processing methods
Science & Technology constitute the primary productive force.We conduct coffee variety study together with Ethiopian Commodity Exchange, ECX and Jimma Coffee Research Center, as we believe that different variety of coffee fits for different terroir, which matters a lot to coffee quality and value.
Meanwhile, experimental coffee processing is popular and gain big market success recently. We area mong the earliest companies to invest in this sector in Ethiopia.
New Coffee2022
China Beauty
Hambella, Guji Zonehiopia
One most popular Ethiopian coffee in China, even East Asia.
Star-product created by Lebunna Coffee since2014 and carefully being upgraded every year from version 1.0 to 6.0 in 2022.
Traditional natural processing with floral aroma and peach and berry-like tastes.
116.China Jushi
China Jushi Co., Ltd. (“China Jushi”) is a core enterprise in the fiberglass business division of China National Building Material Company Limited (HK3323; “CNBM”), and specializes in the manufacture and sales of fiberglass and its finished articles as the main business. China Jushi is one of the largest enterprises in the new material industry of China and in 1999, it got listed on the Shanghai Stock Exchange (Stock name: China Jushi, Stock Code: 600176).
JUSHI'S MISSION
INNOVATE TO DRIVE INTELLIGENT MANUFACTURING AND CONTRIBUTE TO THE DEVELOPMENT OF THE COMPOSITES INDUSTRY

Transform the industry through innovation and upgrade the industry through intelligent manufacturing. Lead the high quality growth of the fiberglass industry and contribute to the sustainable development of the composites industry and the society.
JUSHI'S VISION
MAINTAIN THE LEADERSHIP POSITION IN THE WORLD FIBERGLASS INDUSTRY

Always lead the fiberglass industry and continuously enhance our core competitiveness. Become an industry leader believed in by employees, trusted by shareholders, praised by customers, supported by suppliers, admired by society and respected by competitors. Grow from big to strong to great

Through many years of efforts, China Jushi has become the leading enterprise in the fiberglass industry with sound governance, distinct strategy, good assets, excellent culture, lean management, advanced technology and complete sales network.

China Jushi owns proprietary technologies on design and construction of large E-glass fiber furnaces and environment friendly waste fiber recycling furnaces. The company has developed globally innovative oxy-fuel combustion technology and put it into industrial application which significantly reduces energy consumption per unit of output. We have an advanced fiberglass R&D base including a National Enterprise Technology Center, a Zhejiang provincial key laboratory, and a post-doctoral research station. Our testing center has been certified by both China National Accreditation Board for Laboratories (CNAL) and Germanischer Lloyd (GL).

China Jushi has achieved lean management by following the “Five Targets” (Integration, Patternization, Systematization, Streamlining and Digitization) and the KPI advocated by CNBM. We stick to the overall development strategy and annual operation goals and emphasize on the enhancement of streamlined operation and management processes to strengthen the fundamental management and perfect corporate governance.

With a commitment to “Harmonious Progress, Prudent Governance, Standardized Operation, Lean Management and Innovative Development”, we strive to become an internationally competitive building materials corporation with “prominent main business, sound governance, standardized operation and outstanding operating results”.

JUSHI'S VALUES

Integrity

We conduct business and deal with all stakeholders in an ethical manner.

We act with honesty, integrity and trustworthiness in workplace.

Ethical behavior has become one of our core competitive advantages.

Our continued success depends on following our Code of Conduct.
Innovation
Innovation is the essence of Jushi culture.

Innovation drives our growth which enhances our strength.

We innovate our concept, mechanism, technology and management.

We are always ready to take up challenges and adapt ourselves to changes.
Responsibility
Responsibility is an integral part of our corporate culture.

Jushi shoulders the mission to develop the fiberglass industry.

Jushi is committed to social responsibilities.

We are responsible for our workforce, products, environment and society.

Learning
Learning empowers innovation.

Excellence is our benchmark.

Through learning we upgrade ourselves and enhance corporate value.

Jushi promotes an environment where employees share information, experience, technology and knowledge.

Passion
Passion permeates our corporate culture.

Passion drives the rapid growth of Jushi.

We relentlessly pursue perfection and aggressively seek growth.

We are always full of passion in all our endeavors.

China JUSHI is committed to becoming become the Leader of the World Fiberglass Industry!

Contribute to the Development of the Composites Industry!
Corporate Governance
China Jushi has achieved lean management by following the “Five Targets” (Integration, Patternization, Systematization, Streamlining and Digitization) and the KPI advocated by CNBM. We stick to the overall development strategy and annual operation goals and emphasize on the enhancement of streamlined operation and management processes to strengthen the fundamental management and perfect corporate governance.
PRODUCT&SERVICE
R&D Core
High strength and high modulus glass fiber
Product Description
More than 100 kinds of glass fibercovering the whole field
Quality Assurance
High strength and high modulusglass fiber
Building & Construction
Fiberglass offers the advantages of high strength, light weight, aging resistance, good flame resistance, acoustic and thermal insulation, and therefore is widely used in the building and construction field.Applications: reinforced concrete, composite walls, screen windows and decoration, FRP steel bars, bathroom and sanitaries, swimming pools headliners, daylighting panels, FRP tiles, door panels, etc.
Infrastructure
Fiberglass offers the advantages of dimensional stability, good reinforcing effect, light weight and corrosion resistance, and therefore is a material of choice for infrastructure materials.Applications: bridge bodies, docks, waterside building structures,highway pavement and pipelines.
Electrical & Electronic
Fiberglass offers the advantages of electrical insulation, corrosion resistance, heat insulation and light weight, and therefore is muchpreferred in the electrical & electronic fields.Applications: printed circuit boards, electric appliance hoodsswitchgear boxes, insulators, insulating tools, motor end caps and electronic components, etc.
117.Herocean Group
Herocean Supply Chain Management is a comprehensive enterprise integrating international supply chain services, international logistics trade, African industrial development and information technology. Its headquarters is located in Shanghai, China. It has over ten wholly-owned or controlled subsidiaries. It is also distributed in mainland China, Hong Kong(China),Singapore, Tanzania, Kenya, Rwanda, Uganda, Zambia, Congo (Kinshasa), Mozambique, Malawi, Ghana, Angola and other Asian and African countries and regions. As of the end of 2019, the Group's business grew at a compound annual rate that exceeded the industry average.

Adhering to the core corporate culture of “Openness, Transparency, Persistence, Collaboration and Sharing” and basic corporate culture of “Sincerity,Diligence,Responsibility, Profession, Creativity”, we are committed to “business and every employee learn and grow together, and together share it". With the tenet of “Customer Needs, Our Mission”, we design and implement

convenient, thoughtful and cost-effective third-party logistics solutions for China-Africa international trade customers, and rely on strong physical investment to provide trade commissions from procurement. , the whole international supply chain service of warehousing, shipping, customs clearance and land transportation. As the core second business segment, we are in Tanzania, Rwanda, Zambia, Congo (Kinshasa), Kenya, Mozambique, Malawi and other countries. We have established a professional building materials production base to meet the various needs of enterprises in Africa for investment and development. Based on the development and precipitation of Africa in the past ten years, we continue to invest more in information technology, and it will become the third pillar industry in our company .

All along, we rely on strong strength, efficient and sincere service, focusing on African market development and physical platform, and have built our own brand and influence in the Asia-African region. And relying on "diligence, professionalism, enthusiasm "team spirit, we believe that we will follow your responsibility and create success together.

Our hearts

GOING ABROAD BENEFIT THE WORLD

Our History

Group has experienced more than a decade of development. Many Heroceaner have dedicated their best youth to this "home", and many people continue to create their own glory in that African land. And we have always been adhering to the "stable development" thinking, and the development and development of Africa as a lifelong career.Under the united efforts of all Heroceaner, we have written many new chapters. This is the wisdom, sweat and crystallization of all Heroceaner, and fully demonstrates the entrepreneurial spirit and hard work style of Heroceaner.

Our efforts

We believe that through our own efforts with the team, we can become the "star" of Herocean! And hope that more "Leadership" talents will emerge in this big family.However, it is inevitable that difficulties will arise on the road of growth. All these problems and deficiencies are only tireless efforts and hard work. We will eventually create greater value and lead to a more ideal journey

Our sharing

The company has achieved some achievements and has been adhering to the efforts of Herocean, who has shared every effort. Until this year, we have already owned dozens of employee shareholders except the founder and obtained corresponding corporate dividends every year.

Our expectation

With the rapid development of the company, the company's future hopes are also placed on everyone. In the development of the enterprise, there have been many models of the younger generation, and they have served as the burden of the company's development. They are active, courageous and responsible. Especially for those young people who are going to Africa, they can work in Africa. It should be said that Everyone is dreaming, embarrassed, and constantly improving, will overcome all kinds of difficulties and get more than expected return.

We have always said that Hong Kong's richest man, Li Ka-shing, summed up his life's words as my motto: Never give up. We also always think that there is always a return, and there will always be a moment of light. Destiny is always in your own hands. Therefore, we need to have a more

positive sunshine attitude to deal with every moment, every setback. I believe that we can always go ahead and even go further and higher.

Our vision

Complete all the network arrangement in all African countries by 2025 and achieve outstanding coordination; bring our strengths into full play and provide the best products and services to our customers.

In order to achieve our goals, apart from the great efforts from our teams, we need to closely combine our human resources, financial resources and materials; each department must work closely together, we need to speed up fund operation efficiency, and prepare and provide necessary support for each component of the supply chain. Particularly in the Human Resources Department, we have recruited experts from various industries and engaged them in the improvement and promotion various component, from information technology to purchase service chain and from the marketing chain to supply chain. On the basis of perfecting existing supply chain and trade and industrial industry, we have introduced new information technology, forming a "tripod" structure in order to improve and upgrade the ecological chain and competitive strength of the Company. To truly achieve our "honesty, diligence, responsibility, profession, and innovation" policy, we need to base ourselves on "honesty, diligence, and responsibility" in order to achieve "profession and innovation". Let us work together to grow Herocean in the following four aspects: resource globalization, management localization, service networking, and ideological informatization.

Along with the national "One Belt and One Road" strategy and our "capital" and "talent" globalization, Herocean will start the advocacy of "globalizing outstanding business models and philosophies".

Conclusion

Never drop intention ,follow the trend,and make adaptations to achieve greater success! We are always on the way to share our The road ahead will be long climb will be steep.

Culture

Enterprise Culture

OUR MISSION

Continuous efforts to create a better life for the people of Africa

OUR WISH

Establish a physical network base of organic collaboration in each country in Africa

OUR BASIC CORPORATE CULTURE

Sincerity / Diligence / Responsibility / Profession / Creativity

OUR CORE CORPORATE CULTUREO

openness / Transparent / Persistence / Collaboration / Sharing

OUR INTERNAL VALUE PROPOSITION

Safty / Health / Family / Development / Sharing

OUR EXTERNAL VALUE PROPOSITION

Fulfill commitments to provide the best customer evaluation experience

FOUR MODERNIZATIONS
Resource a location globalization / Management localization / Service networking / Cogitation informatization
OUR EMPLOYMENT PHILOSOPHY
Both ability and virtue / Take virtue first
OUR BUSINESS PHILOSOPHY
One center, two basic points. That is to focus on overseas (African) market development and physical platform: to make the company stronger and bigger, let employees develop together with the company as the basic point.
OUR BUSINESS DEVELOPMENT FACTORS
Talent / Business / Cost Efficiency / Creativity

Social Donation

Public Welfare Action
Sep-27,2017
Herocean Group donated a donation to the poor
Mr. Song Senhua, President of the Group, went to Linfeng Township, Fenghuang County, Hunan Province, Hunan Province to carry out donation assistance and poverty alleviation activities.
Sep-22,2017
Re-take Mr. Xi’s General Secretary, Mr. Song Senhua, President of the Group, to go to Bujugou Village, Xiazhou, for donation and student activities.
Sep-30,2016

118.Chongqing Haifu Medical Technology Co. Ltd.

Founded in 1999, Chongqing Haifu Medical Technology Co. Ltd. is headquartered in Chongqing China and keeps being a world-leading manufacturer of non-invasive ultrasound therapeutic systems for both malignant and benign tumors. Her employee pool exceeds 400 persons including top-class experts in HIFU field.

Cooperating with Chongqing Medical University and its affiliated hospitals, Haifu has established an innovational operation mode integrated of manufacturing, marketing, R&D, and education. These would lead to effective treatment for fibroids and patients suffering from cancer, osteosarcoma and other kinds of tumors.

In October 2005, National Engineering Research Center of Ultrasound Medicine was set up by Haifu, playing an important role of transformation base of R&D, engineering and clinical medicine. Until now, it is still the only one National Engineering Research Center approved by National Development and Reform Commission of China in the field of digital medical device.

The Clinical Training Center, authorized by China Ministry of Health and situated in the 2nd Affiliated Hospital of Chongqing Medical University, has trained and qualified a large number of doctors for non-invasive Ultrasound Ablation from 13 countries and regions.

Furthermore, there are consistently academic relationships between Haifu and foreign organizations, such as University of Oxford (UK), European Institute of Oncology (Italy), Florence University (Italy), Bonn University (Germany), University of Hong Kong (China), etc.

Equipped with original innovation ability and independent intellectual property right, Haifu has established win-win cooperation with some multinational companies such as Siemens, Esaote, Olympus, and etc. Haifu and Siemens Medical Solutions have been jointly developing a MRI-guided Focused Ultrasound Tumor Therapeutic System. The primary consequences of this collaboration will be an important development in the treatment of malignant and benign tumors, such as uterine fibroids and osteosarcoma.

Moreover, from over 20 years’ HIFU R&D experience, Haifu has developed a series of products including the CE marked Haifu Model JC Focused Ultrasound Tumor Therapeutic System, the CE marked Haifu Model JC200 Focused Ultrasound Tumor Therapeutic System, the Seapostar Ultrasound Therapeutic Device for Gynecological Diseases and the Seapopinna Ultrasound Therapeutic Device for Rhinitis. Haifu products have been installed in United Kingdom, Russia, Spain, Hong Kong China, Italy, Japan, Korea, Mainland of China, Romania, Saudi Arabia, Ukraine and etc.

The Haifu Model JC Focused Ultrasound Tumor Therapeutic System offers non-invasive treatment of solid tumors including primary and metastatic liver cancer, breast cancer, primary and metastatic malignant bone tumors, soft tissue sarcomas, and benign tumors such as uterine fibroids and breast fibroadenomas; meanwhile it could be used for pain relief of patients with advanced-stage malignant tumors. The Haifu Model JC200 Focused Ultrasound Tumor Therapeutic System offers non-invasive treatment of uterine fibroids to reserve the patients’ uterus and ablate the fibroids extracoporeally.

President Messages

What we`ve been doing is to offer newer and better therapeutic opportunities to our patients. Every staff member in Haifu, therefore, faces a corporate undertaking rather than a simple job. Frankly speaking, we need time to reach the peak of our business, and first we have to set up a Haifu concept according well with the marketing law. 99% of a task has succeeded, for which much more efforts have been exerted, however, all of the painstaking efforts will probably fail in vain because of the rest 1%. Newer and higher should be the objective of Haifu.

New frequently emerging technologies suggest the speedy development of the society. An explorer must lay himself out to seek for innovation. Only innovation does help an enterprise survive and develop.

We are now working intently to open a new field, since a pause has ended the past success like a stop ends the sentence. We should always have level-headed understanding from the beginning to the end, that the achievement should be left behind and our only choice is to start from a new starting point, another zero point.

Send an email to Prof. Zhibiao Wang: wangzhibiao@haifu.com.cn

Technology

HIFU & Focused Ultrasound

Ultrasound is a form of vibrational wave. It can transmit harmlessly through living tissues, and this makes it possible to use an extracorporeal source of ultrasound for therapeutic purposes. If ultrasound beams are focused and sufficient ultrasonic energy is concentrated within volume while they propagate through tissues, the temperature in the focal region may be raised to levels at which the tumors are cooked, resulting in tissue ablation. This process occurs without any damage to surrounding or overlying tissues, and the tissue ablation technique that employs such beams is known interchangeably as high intensity focused ultrasound (HIFU).

HIFU is a different therapy from conventional hyperthermia, which has been used as an adjuvant to radiotherapy and chemotherapy for cancer treatment since the 1980s. The purpose of hyperthermia to raise the temperature of the tumor from 37℃ to 42-45℃, and to maintain uniform temperature distributions in a narrow therapeutic range for 60 minutes. However, the temperature distributions induced in vivo are usually non-uniform because of tissue cooling by blood flow, and it is extremely difficult to avoid local cold spots that do not reach the necessary therapeutic temperature level. With HIFU, the temperature within the focal zone is rapidly raised to temperatures between 56℃ to 90℃ and is held for one second. The rapid deposition of HIFU thermal energy causes a peak temperature rise that is unaffected by blood flow cooling. Therefore, it can avoid the problem of non-uniform temperature distribution in hyperthermia, and there is no need for inserting thermocouple probes into the targeted tumor to describe temperature distribution during HIFU procedures.

Ultrasound Ablation

Mechanism of Ultrasound Ablation

Several mechanisms are directly involved in the tissue damage induced by Ultrasound Ablation(UA). The first is a thermal effect from the conversion of mechanical energy into heat in the targeted tissue, the thermal effect depends on the temperature achieved during Ultrasound Ablation(UA). If the temperature rise is above a threshold of 56℃ and the exposure time is one second, irreversible cell death will be induced through coagulation necrosis. In fact, the temperature at a focal volume may rise rapidly above 80℃ during Ultrasound Ablation(UA).

The second is acoustic cavitation, the presence of small gaseous nuclei existing in subcellular organcelles and fluid in tissue are the source of cavitation, which can expand and contract under influence of the acoustic pressure is more than several thousand Pascals, and the temperatures reach several thousand degrees Celsius, resulting in tissue damage.

Damage to tumor vasculature, caused by Ultrasound Ablation(UA), may account for the third mechanism of the targeted tissue necrosis. It can directly cease the blood supply to the tumor through the destruction of the tumor-nourishing vessels, thus causing deprivation of nutrition and oxygen for the tumor cells, indirectly resulting in coagulation necrosis.

In fact, it seems impossible to distinguish the thermal effects from either acoustic cavitation or vessel damage in Ultrasound Ablation(UA), and they can occur simultaneously within the targeted tissue. Therefore, the coagulation necrosis induced by Ultrasound Ablation(UA) can be considered as the result of biological effects from a combination of heat, cavitation and vascular

destruction on tissue.
Treatment
Minimize harm to patients
119.XAG
XAG is dedicated to bringing drones, robots, autopilot, artificial intelligence, and Internet-of-things into the world of agricultural production. It creates a smart agriculture ecosystem that leads us into the era of Agriculture 4.0 characterised as automation, precision, and efficiency.
Mission
Advancing Agriculture
Vision
We aim to build the infrastructure of
agriculture for the next 100 years, that will
provide the world with sufficient, diversified,and safe food.

and safe food.
XAG Core Values

User First
XAG users are all residents of this planet, including billions of agricultural producers and consumers, even our planet itself. By transmitting our mission and vision into superb products and services, we pursue Users First as one of our core values, not only aiming to impress customers with our sincerity, but also letting people from all generations enjoy benefits brought from technology.
Keep Promises
XAG regard credit as our life. Treating each other with sincerity and being honest with ourselves are creeds carved in our mind. We know Keep Promises is not always easy since people usually give up holding their principles and surrender to enticements and challenges. Persist promises to our partners and clients, dare to overcome difficulties and say NO to great temptation, that’s how we do
Stay Hunger
Life just like a long trip on a moving train that never turns back. The more places you reach, the deeper understanding of the world you can get. Unless you get tired of exploring and find curiosity faded inside of you, Stay Hunger and keep advancing your life.
Global Vision
XAG is evolving from a technology company towards a Smart Agriculture Ecosystem. The most significant difference between them is not how big the scale but how heavy it shoulders. Global Vision is like a torch we hold meanwhile pursuing our dreams. Even if some of us might start a new career decades after, we’d still be proud of what we once did to improve people’s life.
Chasing Perfection
XAG is a company founded and formed by Geeks. Though our interest may diversify, our mutual character is always Chasing Perfection. Just as perfect person, perfected products is more likely “the carrot on a stick” to us, it is the capacity of providing our customers with better things that really matters, as well as the key to keeping ourselves trustworthy and excelsior

Global Collaborations for the Future of Sustainable Development

Technology is the greatest equaliser of this era. Advancing agriculture is XAG’s business objective as well as social responsibility as a corporate citizen. XAG has established research and development bases in over 20 countries and regions, in partnerships with international corporations such as Bayer, Alibaba, Huawei, and Ping An Insurance Group, to jointly promote smart agriculture and therefore to achieve sustainable development of mankind.

CSR

Technology is a Great Equaliser

Corporate social responsibility is XAG’ s major business as well as our commitment to society.

Extreme Innovation,Empower Next-gen Farmers

XAG dives deep into smart agriculture and keeps empowering farmers with digital technology that catalyses equal opportunities in the rural community.

Unlock new possibility for agriculture

Catering to user needs, we introduce autonomous drones, robot and AI to free farmers' hands and make farm management simpler, smarter and more efficient.

Close the digital divide

We engaged in building high-accuracy field navigation networks that cover 35,000 villages of China. Also, XAG Academy is established to train the next-gen agricultural talents.

Empower rural women

We leverage unmanned technology to break through the physical limit in agriculture, with modern skill trainings provided for rural women to increase their agritech know-how.

Gather Strength, Revitalise Our Planet

We help farmers reduce water and pesticide use through precision technology, while calling on actions to lower carbon emissions and promote biodiversity.

Less consumption, greater development

Precise and even sprays for crop protection are ensured to target where it is needed, growing more food with 30% fewer pesticides and 90% less water.

Reducing our carbon footprint

We turn to electric powered devices as alternative to oil-fired machinery. 760,000 tons of carbon emission was cut down, equivalent to planting 10.32 million trees.

Protecting biodiversity

Facing environmental crises such as bushfires and land desertification, we push the boundary of agritech to restore the degraded wetland.

Safety First, Promote Healthy Living

Improving food security for the table of tomorrow is what we strive for. The well-being of agricultural producers and consumers should be guaranteed.

Fight for zero hunger

In cases of pest disease and natural disaster, we quickly deploy autonomous devices to protect the healthy growth of crops and maintain desirable yields.

Make farming traceable

We work with international agrochemical companies to develop eco-friendly, traceable crop protection solution for the scientific use of pesticides.

Create safe working environment

We explore the innovative use of drone technology for infectious disease control, to make the

world a better, peaceful place.
Participate in Global Anti-epidemic Battle
Facing the COVID-19 crisis, we launched the Spring Thunder operation and set up an RMB 5 million special fund to help enhance the capability of pandemic control in China, while providing global partners with drone disinfection solutions.
Altogether We Grow, For the Future of Food
At XAG, we encourage diverse culture and create an inclusive network of every stakeholder. We strive to become an enterprise that cares for customers, respects partners, and supports its employees.
120. KEDA

INFINITE INNOVATION &TECHNICAL SOLUTION PROVIDER
KEDA Industrial Group was founded in 1992 and listed both on the Shanghai Stock Exchange in 2002 (stock code: 600499) and the SIX Swiss Exchange in 2022 (stock code “KEDA”). With innovative techniques and a global network, KEDA Industrial Group is committed to providing customized sustainable solutions and advanced technologies for customers to achieve a better performance.KEDA Industrial Group have been developed from a single-product manufacturer into a diversified company who is committed to becoming an advance-machinery provider and an innovative technical problem-solver for multiple business sectors. Starting from ceramic machinery, KEDA’s business has been explored into something different, including wall material machinery, stone machinery, building materials, lithium-ion battery materials, lithium-ion battery materials machinery, coal gasification, hydraulic pump, smart energy, etc.

KEDA Industrial Group has a rapid growth in multiple business sectors, especially in the Building Materials Machinery(Ceramic Machinery, Autoclaved Aerated Concrete Machinery, Stone Machinery), Building Materials, Lithium-ion Battery Materials and Machinery. Besides, more business were explored and cultivated, for example, Clean Energy and Environmental Protection, Hydraulic Technology, Smart Energy, etc. 70+ subsidiaries and 20+ research & production bases are currently operated across the world. Meanwhile, products have been sold to 60+ countries and regions.
Science & Technology Innovation
KEDA was named after its strong belief that a corporate will thrive and prosper when it attaches significance to the scientific and technological innovation. By integrating varied resources and platforms from related internal and external stakeholders, KEDA has now achieved a competitive capabilities on self-development researches on core technologies. 20+ research and production bases distributed in Asia, Europe and Africa. 2800+ patents including 470+ invention patents for distinguished industries were authorized by different countries and regions.
Customized Service
By being creative on business optimization, KEDA is no longer a single-product provider but a comprehensive problem-solver. Not limited to the former role of being a traditional manufacturer, KEDA has combined the manufacture with well-considered service. A full-range service package will be included to help customers maximize their production when the products were sold by KEDA, for example, factories design, production line management, staff training, etc.
Empowering People & Societies

As a global company, KEDA is devoted to creating a rapid-growth and friendly platform for talents with diversified culture backgrounds and skill sets to shine. KEDA cares about how to improve the employee engagement and satisfaction. Apart from that, KEDA has a strong responsibility on making a positive contribution to the local communities' sustainable development around the world.

Culture

Infinite Innovation

Bring prosperity to the enterprise by making scientific and technological progress

Human Resource Capitalization

10% of the key employees were granted equity -- the criteria for the export of securities

Global Talents Acquisition

Integrity and Trust; A cross-cultural and multi-industrial platform to grow together

GROUP

121.Wangkang Holding Group

GM MESSAGE

Dear friends,

Thank you for your concern and kindness to Wangkang Holding Group Co., LTD.

Our company was established in 2010, as a solid advocate of "The Belt and Road" concept, we are actively getting involved in overseas market, after years of hard work, Wangkang Group has found subsidiaries in Nigeria, Ghana, Tanzania, Uganda, Saudi Arabia, Iraq, six companies and five large ceramic tile factories, which account for 25% of African ceramic tile production capacity.Saudi Arabia's ceramic tile factory, which is under construction with an investment of $200 million, is also the key project of China-Saudi Arabia production capacity cooperation.

"Thousands of sails compete, the first must be the hardest." we are in the age of great change with each passing day, as "industry goes out" practitioner, we are shouldering the ideal, the responsibility and the innovation.Wangkang people will complete the magnificent turn of our group with the support and help of our friends! We believe that China's building materials manufacturing industry has the strength to fulfill grand plans in the vast overseas market with the help of "The Belt and Road" great decision and build our common entrepreneurial dream. We will firmly work with our partners to make contribution as Chinese private enterprises to the industrialization development of the countries along the "The Belt and Road" route.

Wangkang Holding Group Co. Ltd., hereinafter referred to Wangkang, is an outstanding Chinese private enterprises which engaged in overseas industrial investments.

Wangkang has established ceramic production bases in four African countries, in the land of around 1.5 million square meters. With the investment of around 200 million USD in Africa. Wangkang has occupied more than 30% market share in the African ceramics sales Twelve ceramic tile production lines have been successfully set up in individual plants of different countries including Nigeria, Ghana, Tanzania and Uganda, which achieve great economic and social benefits. Wangkang's tile brand GOODWILL has become a famous ceramic tile brand in Africa which is widely accepted by African people. Wangkang is gradually achieving its mission, collecting individual talent and promoting the spirit of Chinese manufacturing to the world.

Wangkang is making a great move with the strategy of overseas development by investing in the Middle East market in 2018 when the Saudi Arabia company and Iraq subsidies are established. Especially the Saudi Arabia production base which is as a key project of Sino-Saudi Arabia, with the cooperation of Chinese Development and Reform Commission. The infrastructure construction of Wangkang (Saudi Arabia) Ceramic Co., Ltd. Is launched in October 2018. There are four large-scale ceramic tile production lines have been set up with a daily capacity of nearly 200,000 square meters various types of ceramic tiles. Wangkang is making outstanding contributions to Saudi Arabia ’ s industrialization as a representative of Chinese private enterprises.

Co-prosperity coexistence

Sino-African friendship dates back a long Time.Wangkang Group hopes that our blueprint of development in Africa is not only for the sustainable development of Wangkang, but also for the local people's lives. Wangkang will improve the employee's ability of sustainable development through every aspect of enterprise development.

The Belt and Road

In response to the clarion call of “The Belt and Road" advocated by the state, Wangkang has transferred China advantage capacity to Africa and coordinated among the African infrastructure construction,which make the efforts for the economic revitalization of Africa and contribute our powder for the clarion call of “The Belt and Road”

Sustainable Development

Wangkang people attach importance to the harmonious coexistence with nature while enterprise development. We value the sustainable development of the people in Africa. Enterprises are part of society. Wangkang is a drop of water among the Chinese private enterprises. We will continue to make contribution to the economic development of the countries in Africa during the cause of the building material development,train and teach the employees,upgrade our products and equipment, protect and cherish the environment in our factory. This is the requirement and plan of Wangkang for its own development.

Social responsibility

Society helping

Wangkang (Uganda) Ceramics Co., Ltd donates educational funds and teaching facilities and new school uniforms to the local primary school of the Bekaa 2019-08-28

Cultural activity

Employee care

Human resource

Making the best use of people and common development

Talents are the foundation of enterprise development and competition. Wangkang holding group regards talents as the first resource of the enterprise, takes relying on talents as the fundamental premise of enterprise development and respecting talents as the fundamental criterion of enterprise development, and consider promoting the common development of the enterprise and employees as the fundamental task of talent strategy.

In the practice of human resources, wangkang holding group is good at providing a platform for all kinds of talents to fully display their talents and make the best use of them. At the same time, through various effective ways, we keep improving our human resource management skills and

talents’ comprehensive quality, promoting the mutual development of enterprises and employees, and letting employees share the development achievements of enterprises, fulfill common development and share common success.

Common development and Sharing success

Without the efforts of employees, there will be no success of the enterprise, whereas there will be no success of employees without the success of the enterprise. Letting employees and enterprises grow and develop together, Wangkang holding group has always stuck to the concept of talent development. So we encourage employees to combine their own career development with the company's long-term plan, providing employees with multiple growth channels and development models. Being a platform for employees to develop, improve and realize value, Wangkang holding group strives to create good growth conditions for every employee, provides a broad development space for every employee who is determined to become a talented and professional person, enabling employees to give full play to their potential and helping them realize the common development of employees and enterprises. Wangkang holding group insists on the concept of equality and caring for talents. There are no differences in positions between people, only differences in responsibilities. The company respects the personality and pursuit of employees, encourages employees to improve their abilities and recognizes their achievements. At the same time, we always focus on the concept of developing by employees, developing for employees, and sharing development achievements with them. Therefore,We pay attention to the interests of both enterprises and employees and advocate unity and cooperation between enterprises and employees to jointly create and share value in our work towards realizing the win-win situation of common development and success sharing between enterprises and employees.

122.Poly Sinolight

As a pillar of national economy and a fundamental livelihood industry, light industry involves all aspects of people’s life. It is closely related to people’s basic necessities and plays a major role in meeting their needs for a better life.

The national industry was backward overall in modern China. After the founding of new China, industrialization was set as the strategic objective of top priority. Since the first “Five-Year Plan” period, a number of institutes had been established to promote the development of light industry, including national scientific research institutes in the fields of daily chemicals, food and fermentation, pulp and paper, leather and shoemaking, etc., as well as light industry engineering design institutes in major cities across the country. These state-level research and engineering design institutes of light industry are the predecessors of present subsidiaries of Sinolight Corporation. By self-reliance and independent innovation, they have created records in research and engineering in new China with a lot of technologies and products. As a major participant in industrial development, technical support and project construction of China’s light industry, these institutes have made significant contributions in driving the industry to grow into big and strong from scratch and meet people’s needs in life.

Through seven decades of restructuring and development, with hard work and great effort of

several generations, Sinolight has developed into a large innovative enterprise group in China’s light industry. In science and technology R&D, by giving full play to its advantages in scientific research platforms and talents, Sinolight has made a number of major achievements of international level and widely applied them in people’s life. By playing a leading role in formulating many ISO standards, Sinolight has won a bigger say for China in international standards formulation and firmly supported domestic products’ entrance into global market. In engineering services, Sinolight is capable of providing services of entire process, high quality and full package, by integrating engineering design, consulting, general contracting, project management and construction supervision, etc. On the path of sustainable development, Sinolight leads in industrial energy conservation and emissions reduction by actively promoting new technologies, techniques and materials. In the field of light industrial equipment, especially pulping and papermaking equipment manufacturing, Sinolight takes intelligence as the direction and provides customized solutions for clients with both system integration and core equipment development. As a leader of scientific and technological progress, industrial transformation and upgrading in China’s light industry, Sinolight has always been demonstrating its influence and guidance of state-owned enterprise.

Since 2017, the year Sinolight was restructured into China Poly Group Corporation, World Top 500, Sinolight has come to a new stage of development. It closely follows the state strategy of building pro-innovation country, works towards enabling people to live a better life and abides by the high-quality development as a new concept for development. Sinolight takes the opportunity and achieves the consensus by laying a new strategic development planning. Sinolight proactively seizes the period of strategic opportunity that the state drives “Internet Plus”, vigorously promotes the building of Internet Data Information Platform of light industry and dedicates to providing customers with integrated, systematic and customized services. Besides, Sinolight integrates its best resources in industrial testing and commits itself to providing high-level services for entity enterprises.

The wind is strong enough to set sail. Now is the time to blaze the trail. Guided by the new strategic development planning, Sinolight pursues the vision of “For a better life”, upholds the values of “Pragmatic, Innovative, Open, Collaborative and Responsible”, and positively fulfills its mission of “To empower the industry and create a better life with science and technology”. Sinolight is striving to become a scientific and technological innovation industrial group in light industry, which is nationally leading and internationally renowned.

Sinolight Corporation (“Sinolight”), headquartered in Beijing, is a wholly-owned subsidiary of China Poly Group Corporation, a World Top 500 company.

After nearly seven decades of reform, development, reorganisation and integration, Sinolight has now developed into a large-scale innovative enterprise group in the domestic light industry, forming five major business landscapes including scientific research and achievements industrialisation, whole-process services in intelligent engineering, intelligent equipment and industrial internet, trade and asset management, overseas industrial investment and operation.

Sinolight has many state-level scientific research platforms and a well formed scientific research team and R&D system. It possesses comprehensive service capabilities for light industry-related fields through scientific research, design, intelligent manufacturing, trade and other complete industrial chains. Its business covers more than 60 countries and regions worldwide. As a leader of scientific and technological progress, industrial transformation and upgrading in China’s light industry, Sinolight has always been demonstrating its influence and guidance of state-owned enterprise.

Records of Sinilight

The inventor of the first ultra-thin capacitor paper in China

—— China National Pulp and Paper Research Institute

The technology inventor of the first bottle of dry red wine and dry white wine in China

——China National Research Institute of Food & Fermentation Industries

The leading research institute in baijiu production industrialization in three major pilot projects, namely, Yantai, Fenjiu and moutai, which are landmarks in the development of China's baijiu industry.

——China National Research Institute of Food & Fermentation Industries

The technology provider and manufacturer of the first synthetic detergent, enzyme detergent and detergent paste after the founding of China

——China Research Institute of Daily Chemicals Industry

The creator of the first industrialized pigskin leather- making technology in China

——China Leather & Footwear Research Institute

Published Paper and Biomaterials, the first English academic journal in China's paper industry

——China National Pulp and Paper Research Institute

Published China Detergent & Cosmetics, the first professional English journal in daily chemical industry

——China Research Institute of Daily Chemicals Industry

Established 2 of the first 14 "Belt and Road" joint laboratories

——China-Ethiopia Joint Laboratory of Leather Technology; China-Cambodia Joint Laboratory of Food Industry

The first listed company of engineering design service sector in China

——China Haisum Engineering Co., Ltd (SZ. 002116)

The first company supplying conveying and wrapping system in pulp and paper industry to replace imported equipment, and supplying the most complete equipment for finishing house in pulp and paper industry in China.

—— Chaint Corporation

One of the first enterprises authorized to engage in foreign trade and overseas engineering contracting projects

——Sinolight International Holdings Corporation
Culture
Vision

For a Better Life

Mission

To empower the industry and create a better life with science and technology

Values

Pragmatic, Innovative, Open, Collaborative and Responsible

Business Philosophy

Focusing on customers

Seeking win-win solutions

Aiming at solving problems

Following market demand

Strategic Positioning

Through scientific and technological guidance and innovative development, Sinolight will be the main force of research and development in the national light industry, the promoter of scientific and technological innovation, the practitioner of high-quality development of industrial chain. Sinolight relies on technological guidance and Innovation-driven development to become a

scientific and technological innovation industrial group in light industry, which is nationally leading and internationally renowned
Scientific Research and Achievements Industrialization
Systematic Scientific Research Team

Sinolight has nearly 7,000 employees, of which more than two- thirds have a bachelor’s degree or above and are professional technicians. It has more than 100 experts enjoying special government allowances from the State Council, 3 experts from the national “Ten Thousand Talents Program”, and 1 person from the “State Ten Million Talents Project”, being an important “think tank” in the light industry and the main source of relevant industry experts for ministries and commissions including the National Development and Reform Commission, the Ministry of Industry and Information Technology, and the Ministry of Science and Technology.

Substantial Scientific Research Achievements

Since the 13th “Five-Year Plan” Period, Sinolight has undertaken over 300 national projects. It has formulated and revised over 900 national and industrial standards, accounting for more than 80% of the total of relevant industries and has led the formulation and issuance of 15 international standards in the field of surfactant and footwear standardization. It has been granted more than 1,200 authorised patents, ranking at the forefront of central enterprises in terms of the number of authorised patents granted. It has won more than 1,300 provincial and ministerial level awards, and has established cooperative relations with over 100 foreign scientific research institutions and universities.

National R&D and Service Platform

4 National research institutes (pulp and paper, food and fermentation, daily chemistry, leather and footwear)

3 National Science and Technology Innovation Base

5 National Quality Inspection and Testing Center

28 National International Standardization Platform

5 State-level international science and technology cooperation bases

2 Two of the first 14 of “Belt and Road” joint laboratories

5 National Industry Alliance

1 Post-doctoral scientific research workstations (with four branches in four state-level research institutes)

Right to grant professional master’s degrees in 4 specialty directions
Responsibility
Targeted Poverty Alleviation
Popular Science Promotion
Foreign Aid Training
Foreign Assistance
Sinolight has made solid efforts to implement the decisions and arrangements of the Central Government on poverty alleviation and rural revitalization. On the principles of “accuracy, effectiveness and efficiency”, Sinolight has combined industry support with the support of education. It has continuously increased inputs in information, technology, capital, projects, intelligence and talents, striving to create the five-in-one (industry, education, public welfare, consumption and employment) targeted poverty alleviation model to promote the economic and social development of designated area. Poverty alleviation has been effectively integrated with rural revitalization.

In April 2019, Karijini Inner Mongolia, Sinolight’s designated poverty alleviation site, successfully removed the label of National Impoverished County. In July 2019, General Secretary Xi Jinping paid an inspection visit to Karijini, which was a great encouragement to the targeted poverty alleviation work of Sinolight.
Sinolight actively promotes scientific knowledge and carries forward the scientific spirit through practical work such as the Science and Technology Open Week and the Youth Popular Science Lectures.
Closely combining foreign economic and trade business with fulfilling overseas social responsibilities, Sinolight has been carrying out a variety of foreign economic assistance for years. Besides effectively promoting the expansion of the company’s overseas business, foreign assistance has also further strengthened the political and economic exchanges between China and the recipient countries, deepened bilateral friendship and cooperation, and greatly enhanced the local influence of Chinese enterprises.

Mali Sukala S.A. and N-Sukala S.A., as the two unique large-scale sugar manufacturing enterprises in the Republic of Mali in West Africa, have played an irreplaceable role in promoting the local national industry, developing social economy, creating a large amount of employment, and meeting the people’s sugar needs. The company had also funded the construction of China-Mali Friendship Embroidery Center and the teaching building in Women and Children’s Fund Training Center, provided a large number of medical facilities for local hospitals and clinics, built roads and deep wells for the residents free of charge, sponsored materials and equipment to schools, etc. Sinolight has established a reputed image of Chinese enterprise in Mali.
123.Mainland Group
Mainland Group was establishment in 2001. In terms of marketing and sales, as the market economic system becomes sound and perfect, the competitive situation is also changing constantly. After Mainland Group perceives such change, it carried out the corporation reorganization twice successively, realizes the external innovation of the enterprise The group is a global enterprise specializing in agricultural commodity and trading At present, the business types include the import & export business of natural rubber, mixed rubber, synthetic rubber,

cotton, palm oil, tapioca chips, starch, soybean, grain and oil, wheat, sugar and other products.

124.Tiantang Group
The business involves eight fields, including park construction, hotel catering and tourism, manufacturing, real estate development, mineral development, machinery trade, and security services.

While running the business, Mr. Zhang Zhigang never forgets to assume social responsibilities. He provides various forms of funding to local schools, hospitals, churches, wildlife protection organizations and government agencies every year. During the time of COVID-19, actively organize donations and donations;

Mr. Zhang Zhigang has been received many times by the leaders of China and Uganda, such as former Premier Wen Jiabao, former Chairman of the Standing Committee of the National People's Congress Zhang Dejiang, Chairman of the National Committee of the Chinese People's Political Consultative Conference Wang Yang, Chinese Foreign Minister Wang Yi, Ugandan President Museveni, Ugandan Deputy President Secandi, Prime Minister Rugunda of Uganda, etc.;

Mr. Zhang Zhigang has been invited to many important events, such as the military parade held to commemorate the 70th anniversary of the victory of the Chinese War of Resistance Against Japanese Aggression and the World Anti-Fascist War; Congress, the Beijing Summit of the Forum on China-Africa Cooperation, the 2nd Belt and Road Forum for International Cooperation, the 1st China-Africa Economic and Trade Expo, the Celebration of the 70th Anniversary of the Founding of the People's Republic of China, etc.;

Mr. Zhang Zhigang has been interviewed by well-known media in China and Uganda for many times, such as CCTV, Hunan Satellite TV, Hebei Satellite TV, Hebei Economic Daily, Party Magazine, Uganda "New View", NTV TV, etc.
Mr. Zhang Zhigang, born in Tangshan, Hebei in 1972, served in the Hunan Armed Police Force for three years from 1990 to 1993; in 2000, he went to Tanzania to start a business and engaged in luggage trade; in 2002, he opened a luggage processing factory in Uganda, and then established Uganda Tiantang Group in 2006.

At the beginning of its establishment, the group has implemented the development strategy of "localization" and "diversification". The group's business now covers 8 major areas: hotel and catering, tourism, manufacturing, real estate development, mineral development, machinery trade, security services and park construction. It has more than 20 subsidiaries. As a diversified and comprehensive large-scale Chinese enterprise group with more than 3,000 local employees, not only the local employment problem has been greatly improved, but the technical backbone and skilled workers trained by the group have improved the overall technical level of local employees;
Responsibility
On June 27, 2020, China-Uganda Mbale Industrial Park donated 150 pieces of COMFOAM foam

mattresses to the Ugandan government. These materials will be used in the anti-epidemic work of the Orungo-Constuency Regional Hospital to help the local build a better medical system.

On May 22, 2020, Tiantang Group, on behalf of the Mercer Entrepreneurs Federation, donated materials to the Mbale Catholic community and the Muslim community, and donated 20 tons of corn flour to the two societies respectively. Zhang Zhigang, Chairman of Tiantang Group

092022.05

On May 1, 2020, China-Uganda Mbale Industrial Park donated anti-epidemic materials worth about 100,000 yuan to the Mbale area. Zhang Zhigang (first from the right), chairman of the China-Uganda Mbale Industrial Park, was donating with the regional government and donors. Group photo at the ceremony.

125.CTG

A World-Class Clean Energy Group

To construct the Three Gorges Project and develop the Yangtze River, the China Three Gorges Project Corporation was founded on September 27, 1993, with the approval of the State Council. On September 27, 2009, it was renamed the China Three Gorges Corporation (CTG). CTG positions itself as a clean energy group focusing on large-scale hydropower development and operation. Its main businesses cover the construction, international investment and contracting, development of wind power and solar energy among other renewable energies, comprehensive development and utilization of water resources, as well as providing relevant professional technical services. After more than 20 years of rapid growth, CTG has become the largest hydro-power development enterprise worldwide and the biggest clean energy group in China.

An Integrated Business Model

By the end of 2016, CTG's consolidated installed capacity had reached approximately 70,000 MW, and the total installed capacity, including capacities that were commissioned, under construction, and on a minority-equity basis, had reached 118 GW. Specifically, renewable clean energy accounts for 97% of the total mix, while consolidated hydropower capacity represents 16% of the total installed capacity of hydro in China. By the end of 2016, the assets of the corporation had reached RMB 660 billion, and it ranked high among all SOEs in terms of total profit, net profit attributable to the parent company, ratio of profits to cost, overall labor productivity, per capita profit, and per capita profit and tax.

A Global Developer

CTG assumed overall responsibility for the construction and operation of the Three Gorges Project. After 2 decades of handwork, the preliminary design and construction work of the Project was completed as scheduled in 2009.The trial operation of the ship lift began in September 2016.Upon authorization of the State, CTG developed, constructed and operated cascade hydropower stations on the lower reaches of Jinsha River, composed of four world-class large hydropower stations (i.e. Xiluodu, Xiangjiaba, Wudongde and Baihetan). CTG also focused its efforts on wind power, solar power, and other new energy sources as its second principal business, and strived to lead the development of offshore wind farms. In addition, CTG kept in step with the "Belt and Road" initiative and actively upgraded the "going global" plan of the Chinese hydropower industry. As yet, CTG's overseas investment and contracting business has expanded to over 40 countries and regions in Europe, America, Africa and Southeast Asia, with a

total installed capacity of over 15 GW. Overseas business has offered a major impetus for the sustainable growth of CTG.

Promoting Sustainability

During the development and construction process of hydropower plants, CTG upheld the principle of "building a power station to stimulate the local economy, improve the local environment, and benefit the resettled residents" based on innovation, coordination, green development, opening-up, and sharing. As a central SOE, CTG fulfilled all due social responsibilities. In addition to flood control, water resource preservation, energy saving, emission reduction and other ecological benefits that can be expected out of a cascade development, CTG pursed social and economic benefits as well through engineering or technical measures and scientific regulation. Besides, CTG maintained an active presence in the fields of targeted poverty alleviation, and provided assistance to Xinjiang and Tibet, among others. CTG also coordinated hydropower development with efforts to provide material benefits to resettled local populations, protect the ecological environment, and promote local economic and social progress, so as to share the achievements of reform and development with a larger population.

Vision

To become a world-class multi-national clean energy group with international competitiveness

Mission

Build the Three Gorges Project and develop the Yangtze River resources;
provide clean energy and build beautiful communities together

Core Values

Dedication

Responsibility

Innovation

Harmony

Three major transitions

From a builder of major projects to a key market player

From a hydropower company to a leading clean energy group in the world

Three leading roles

A leading role in the global hydropower industry

Leading the world in the sustainable development of the hydropower industry

A leading role in the "going global" initiative of China's hydropower industry

Leading the whole industrial chain of China's hydropower industry in its going global endeavor

A leading role in offshore wind power development

Leading the innovative development of offshore wind power and other new energy sources

From a mainly domestically-oriented company to a transnational entity with a dual
focus on domestic and overseas markets

Our Sustainability Model

We manage our corporation through the principle of "localization with global thinking", by actively supporting local economic development, creating jobs and improving local people's livelihood. We proactively take on social responsibility initiatives, protect biodiversity and the ecological environment, and play a bigger role in boosting local prosperity and development.

Preserving Natural Resources and Biodiversity

In 2016, CTG assessed the environmental management system, compiled and released "CTG Management Approach for the Three Gorges Environment Fund Project".

The Yangtze River basin supports 36% of all freshwater fish species in China

In 2016, CTG invested RMB 930 million in environmental protection and released 2,020 large Chinese sturgeons. The wild Chinese sturgeon has resumed natural spawning. Environmental protection volunteers, environmental organizations, universities and media from six provinces in the middle and lower reaches of the Yangtze River conducted a series of public awareness campaigns in 9 riverside cities such as following the migratory route of sturgeon to forge a joint protection network in all reaches of the Yangtze River.

CTG has collaborated with WWF-China and research institutions to implement e-flows at the dam to mimic the Yangtze River's natural flood pulse and promote carp spawning. To restore eco-hydrological processes during the carp spawning period, CTG funded the necessary studies: field survey, analysis of hydrologic and fish biology data, numerical modelling. The effectiveness of e-flows implementation for Chinese carp is also helpful in conserving the biodiversity of freshwater fishes and has socio-economic benefits through its contribution to fresh water aquaculture.

Three Gorges Nursery Research Center

By the end of 2016, the Three Gorges Nursery Research Center had introduced more than 17,000 rare and precious plants of 256 types to the Three Gorges dam. Through traditional reproduction, 13,000 seedlings were cultivated, including Hibiscus hamabo, Aesculus, Magnolia officinalis, Winceltis and Chinese yew.

CTG has transplanted 21 ancient trees along the Shenyu River on the left bank of the construction area for the Wudongde Project and strengthened the tending to ensure the surviving rate.

Liquidambar is also called the "Pioneer of barren hills".

It is a precious multi-colored leaf species in China and an excellent ecological protection tree with very high ornamental value and ecological value. After more than two years of exploration and research, the scientific research team has achieved the objective of establishing a sterile system of liquidambar and obtained the patent licensing from the State Intellectual Property Office.

Training on Ecological Restoration of Rivers

CTG invited the experts of The Nature Conservancy (TNC) to give a two-day (January 25-26, 2016) training on ecological restoration and planning of rivers in Beijing. During the training, the experts introduced the principles, methods and technologies of ecological restoration of rivers. Apart from citing some international cases of river restoration, they introduced the hydropower development of the mainstream and the protection of tributaries in the upper reaches of the Yangtze River.

Our Sustainability Model
We manage our corporation through the principle of "localization with global thinking", by actively supporting local economic development, creating jobs and improving local people's livelihood. We proactively take on social responsibility initiatives, protect biodiversity and the ecological environment, and play a bigger role in boosting local prosperity and development.
People at the heart of our operations
Through their talent, our men and women make an enormous contribution to our leadership. In each of our structures, our priority is to ensure their safety, their development and their well-being.

Safety first and foremost
As far as health and safety initiatives are concerned, CTG is capitalizing on a demanding policy targeting zero accident. The keys are training, empowering and involving every employee.
Training and personal development
Invest in training to ensure the professional development of our employees. Internal mobility is encouraged at all levels, this allows each employee to make progress in his or her career and guarantees the spreading of knowhow.
Equal opportunities
The Group strives to ensure equal opportunities to its employees from recruitment and throughout their careers.
Support to local economies and supply chain
Our infrastructure should also contribute to the economic and social wellbeing of the communities in which we operate, create new employment and offer professional development and training opportunities.
Open dialogue and support to communities
We support local communities through direct interventions and social programmes: free health interventions in the villages and construction of local infrastructures.

Our best practices overseas
Guinea Souapiti hydropower station. In May, 2016, major construction works of Yabha new village was completed by CTG. Yabha new village is about 7KM from the former site and covers an area of 130,000 square meters. It is composed of 102 houses with auxiliary facilities, one school, one clinic, one mosque and one market.

Uganda, Isimba hydropower station. The project provides free medical services to the villagers of Kayunga and Kamili districts. About 1300 people benefited from this service.

Pakistan, Karot Hydropower project. ?Improvement of existing and construction of new access roads and bridges will facilitate the population of the area in their movement and transportation specially the population of Kahuta and Kotli districts. New business activities will bring social uplift in the life of people.

Brazil, Sao Manoel Hydropower Station. CTG helped with the radio communication project for surrounding Indian people’s residential areas, and constructed radio communication systems for

21 villages.

Our Commitment
Statement
CTG continued success and global growth can only be maintained and guided by our Core Values of Dedication, Responsibility, Innovation, and Harmony. When we individually and collectively conduct ourselves honestly, fairly, responsibly, and with integrity in all of our relations internally and externally, we honor these values and focus on people and harmony.

CTG has adopted the Ethic and Compliance Principles applied widely among CTG entities. The Principles sets out the rules which executive directors must be familiar and comply with, and which they must ensure compliance with in their entities.

CTG commits to contributing towards sustainable development by means of environmental protection, economic growth, social progress, professional training of personnel. Today, in response to the growing importance that these aspects are assuming in the economic and industrial worlds, CTG believes it is necessary to promote a Sustainable Development Policy that can serve as a guide and reference for all staff and all sectors where the group operates, both now and in the future.

The implementation of these policies is subject to the CTG s monitoring and audit system, and these policies apply to all CTG group entities.

126.SHEIN
SHEIN is a global online fashion and lifestyle retailer with a mission to make the beauty of fashion accessible to all.
Our mission is to make the beauty of fashion accessible to all, reimagining fashion by leveraging our small-batch on-demand production model. This innovative, customer-driven model allows for more choice at more affordable prices while minimizing waste, by measuring customer preferences more accurately and efficiently.
Our vision is to offer every customer exactly what they want. It’s a vision we’ve worked toward since 2012 by empowering customers, creators, independent suppliers and individual entrepreneurs.
Customer-first is SHEIN’s guiding principle. Since our beginnings in 2012, SHEIN has put our customers at the heart of our business. Through our small-batch, on-demand production model, we deliver exactly what each customer desires by accurately forecasting demand and responding quickly to demand signals.
SHEIN’s portfolio of brands provides something for every customer. Our brands strive to meet the diverse needs of our global customer base, from chic apparel, to cosmetics, to the cutest looks for our customers’ furry friends. In addition to SHEIN’s brands, the SHEIN Marketplace hosts other renowned global brands in various markets.
We are committed to operating responsibly across every area of our business and hold ourselves to leading international standards. The trust of our customers and partners is paramount to what

we do, so we implement leading industry practices and policies designed to protect those we work with.

127.Yuan Long Ping High-Tech Agriculture Co.,Ltd.

About Yuan Longping

Yuan Longping, born in Beijing in 1930, now lives in Changsha of Hunan Province, an Academician of Chinese Academy of Engineering, and the founder of hybrid rice, known as the "father of hybrid rice".

Academician Yuan took the lead to carry out rice heteros is application research in our country, published the famous paper Male Sterility in Rice in 1966, achieved the matching "three lines" of sterile lines, maintainer lines and restorer lines of hybrid rice in 1973, cultivated the world's first hybrid rice variety "Nan You No.2", obtained success in "two-line method" hybrid rice research under the charge of him in 1995, launched and led the research of "super hybrid rice" in 1999, and realized the research goal with yield per mu exceeding 1,000 kg in 2015.

" Develop hybrid rice to benefit people of the world" is a lifelong pursuit dream of Academician Yuan. Since the hybrid rice of China went abroad for the first time in 1979, it has been studied and promoted in more than 80 countries and regions in the world, and it has made a significant contribution to food security of the world.

Founded in 1999 and listed in 2000, Yuan Longping High-tech Agriculture Co., Ltd. ("Longping High-tech") is a high-tech modern seed group named after the academician Yuan Longping, the "Father of Hybrid Rice", who serves as Honorary Chairman of the company, with its largest shareholder being CITIC Group. Since its foundation, Longping High-tech always insists on being led by strategy and driven by innovation. It is determined to strive for the dream of developing national seed industry under the mission of "Progress the seeds, Benefit the world".

The company is the leading seed industry enterprise "integrating breeding, reproduction and promotion" in China. Its main business covers two major systems of seed operation and agricultural service. The hybrid rice seed business is leading globally, and the corn, pepper, cucumber, millet and edible sunflower seed business leads China. In 2018, the company gained revenue of 3.579 billion yuan and net profit of 791 million yuan attributable to shareholders of listed companies, and ranked among the top eight global seed enterprises.

Strong R&D strength is the core competitiveness to support the company's sustainable development. The company has established the domestic leading commercial breeding system and testing system and set up the international advanced biotechnology platform, with its R&D and innovation ability of main crop seeds ranking the leading level in China. The company has maintained a high level of scientific research input for many years in a row, with the R&D input accounting for about 10% of the operating income. In the past three years, it has invested nearly 1 billion yuan of R&D expenses, which greatly exceeds the level of the peers in China.

The company firmly promotes the internationalization strategy. In Southeast Asia and South Asia such as India and the Philippines, R&D of rice variety has entered the stage of concentrated output of results, and the breeds are highly competitive in local markets. In the South American market, corn varieties mainly focus on the high-end and medium-

RESEARCH,DEVELOPMENT&INNOVATION

The company(LPHT) has built a complete commercial breeding system that focuses on company as the mainstay, market-oriented, scientific design, rational division of labor, standard operation, streamlining, and resource sharing, and has established a world-class commercial breeding platform. There are 13 rice breeding stations, 13 corn breeding stations and 7 vegetable breeding stations in the main ecological areas of rice and maize in China and abroad. The total area of the base is nearly 7,000 mu. For rice, corn, cucumber, pepper, etc., a large-scale unified test evaluation platform has been established, which has become the largest and highest-level test system in the domestic seed industry.

LPHT has established a world-leading biotechnology platform, including the China Biotechnology R&D Center and the Brazilian Biotechnology R&D Center. China's biotechnology R&D centers include Tianjin Laboratories, Henan Jiyuan Laboratories, Hunan Changsha Laboratories and Huazhi Biotechnology Co., Ltd. LPHT has introduced several high-throughput automated large-scale liquefaction instruments, including automatic DNA extraction workstation and SNP genotyping instruments. LPHT R & D will focus on crop breeding, biotechnology, big data science, etc. to fully promote the collaborative innovation of various R & D platforms.

CSR

Foreign aid technology training

LPHT is one of the five ”Foreign Aid Project Implementation Enterprises” qualified enterprises awarded by Ministry of Commerce. At present, the company has trained more than 5,000 agricultural officials and agricultural technology experts for more than 60 countries .We provide the professional training courses;meeting and seminar arranging;organizing conferences in the field of agricultural.

Foreign Aid Cooperation Projects

Long Ping High-Tech is committed to helping other countries develop agricultural production and promoting them to solve food security problem with Chinese modern agricultural technology. It has successively undertaken more than 10 national foreign aid projects, mainly including “China-Philippines Agricultural Technology Center Project”, “China-East Timor Hybrid Rice Technology Cooperation Project”, “China-Liberia Agricultural Technology Demonstration Center Project”, “Brazil Hybrid Rice Research and Development”, “China-UNEP ‘Africa Water Action’ Water Saving Agriculture Demonstration Project”, “China - ESCAP Hybrid Rice Technology Promotion Project” etc.

Talent Concept

Respect, care for every employee;. all employees are equal in dignity and access to development opportunity.

Provide a good working environment to employees, and create a warm working atmosphere and simple and harmonious interpersonal relationship.

Young people are the company’s future; it is necessary to build mechanism and platform to encourage excellent young talents to undertake responsibility,

strive to work and act actively.

No idlers at work and no lazy man on the team.

Everybody has to start somewhere.

Competition tells more than selection.
Correct selection, fair use, diligent education and strict management of talents.
Trust People,Achievement People,Educating People
Seed Operation
Main business of seed industry operating company focuses on seed industry, taking hybrid rice, corn and vegetable seed industry as the core, and taking wheat, cotton, rape and other seed industries as the extension, so as to be a all round player in seed industry. At present, the market share of hybrid rice seed ranks the first in the world, hybrid corn seed ranks the first echelon nationwide, the promotion area of hybrid pepper seed ranks the first nationwide, and cucumber breeding scale is leading the world.
Agriculture Service
Agricultural services are based on the concept of making profit for cultivator and bringing benefit to eater. The company has continued to expand agricultural service value chain, its business covers precision planting technology,new professional peasant training,quality grain trading platform,cultivated land rehabilitation and development, brand agriculture and agricultural finance etc, and it creates an agricultural integrated service platform by including peasants, dealers and other means of agricultural production, finance and other partners into the integrated service ecological circle.
128.China National Agricultural Development Group Co., Ltd.

China National Agricultural Development Group Co., Ltd. (hereinafter referred to as "CNADC") was founded in October 2004 upon the restructuring and merging of China National Fisheries (Group) Corporation with the China Animal Husbandry (Group) Corporation and changed its governance structure and name in January 2011 according to the Company Law. It is the only central comprehensive agricultural enterprise under the direct governance of the State-owned Assets Supervision and Administration Commission (SASAC) of the State Council. It is a leading enterprise in national animal epidemic diseases and is irreplaceable in meeting China's agricultural needs. CNADC holds 17 wholly owned or share-controlled subsidiaries, and 3 publicly listed companies. CNADC's business can be found in all provinces, autonomous regions and municipalities in China. It has established branches or bases in more than 40 countries and regions around the world and maintains economic and trade ties with over 80 countries and regions.
As a wholly state-owned company, CNADC devotes itself to international cooperation and the exploitation of agricultural and fishery resources. Domestically CNADC keeps in mind its aim to serve "agriculture, rural areas and farmers" and actively promotes the development of agricultural industrialization. After years of development, CNADC has gradually built up three core businesses: development of strategic resources, with focus on pelagic fishing and agricultural resource exploitation; research and development, manufacturing and distribution of biological vaccines, vet-medicines and feed additives; and modern seed, agricultural insurance and agricultural international trade. Meanwhile, it develops supplementary services for core

businesses, such as diesel manufacturing and port construction.

CNADC adheres to the plans made by the Central Committee of the CPC and the State Council to deepen reforms in state-owned enterprises and follows the SASAC’s guiding principles “to grow stronger and larger” in a bid to implement the “Twelfth Five-year Plan”. CNADC will continue its efforts regarding enterprise reform, further adjust and optimize its business structure and make a more reasonable allotment of resources so as to enhance the enterprise’s core competition. CNADC will cater to the domestic and international markets and strive towards more ambitious goals.

129.China Shandong International Economic &Technical Cooperation Group Ltd (CSI)

China Shandong International Economic &Technical Cooperation Group Ltd (CSI), founded in 1984 under the ratification of Chinese State Council, is a large-scale foreign economic enterprise group with the most diversified qualifications and licenses for international trading and it is the earliest of its kind in Shandong province. In 2008, CSI became a wholly-owned subsidiary of Shandong Hi-Speed Group Co., Ltd(SDHS) and its platform and window for implementing the “Going Out” strategy. Relying on the overall advantage of SDHS, CSI established an international development structure in which CSI is the leading unit and the other specialized brother companies will be supporting units to develop more new projects. So far, CSI has the business scope of international contracted engineering, China-aid project, international human resource cooperation and exchange, etc. In five continents, CSI has branches and regional companies in over 30 countries and enjoys a high international reputation. In the past 30 years, with its rich experience in international economic cooperation and an outstanding international business, engineering, investment and management team, CSI has finished more than 200 housing construction, transportation, port, tunnel, sports stadium, airport runway, water supply, wastewater treatment and municipal engineering projects in over one hundred and six countries and regions. Transportation, real estate, Agricultural, energy, and mineral projects have been carried out in Serbia, Ghana,Sudan, Canada, Fiji and Guinea. More than 40,000 trainees have been sent to Japan, South Korea and Germany by CSI. CSI has won the honors of “The 41st Among China’s 500 Biggest Service Enterprises”, “Trustworthy Enterprise by China Customs”, “One of China’s Biggest 50 Foreign Contracting and Labor Service Enterprises”, and “The Best Overseas Contracting and Labor Service Enterprise in Shandong Province”. CSI being selected into ENR 250 world Largest international contractors list. As the chairing company of Shandong International Contractors Association and managing director of China International Contractors Association, CSI has made very important contribution for China’s international economic and technical cooperation.

Enterprise culture

Under the Road & Belt Initiative and relying on main businesses of Shandong Hi-Speed Group, we adopt a people-oriented concept to seek international cooperation at the guidance of Shandong’s policy to foster new growth drivers to replace old ones. As a platform to integrate quality resources of home and abroad, we take advantages of our global market experience to create a “CSI” brand of Shandong Hi-Speed Group with an influential impact on global business, international contracting, and investment areas under the environment of go-global and bring-in strategy. We are dedicated to create an atmosphere of happiness for our staff.

Global Engineering Service, Investment and Financing Sector： We shall mobilize resources in engineering service and investment and financing sector and do our best in internationalization of CSI s main business.

Multinational Enterprise Group： With international market-oriented strategy and global vision, we aim at effectively allocating resources around the world and cooperating with all the countries and regions to achieve a win-win situation and good reputation.

International Influence： Influence does not merely refer to the maximum economic returns but also highlight the respect shown by all clients and competitors in the same industry. With excellent development strategy, innovation capabilities outstanding talents, abundant capital, corporate culture and brand effect, we are engaged in strengthening our overall competitiveness to become an multinational corporation with global influence which enables us to participate in formulating industrial standards and contributing to the sound development of global engineering service and investment and financing sector.

To Deliver Value to Clients： We shall satisfy the demand of clients and provide them with quality and efficient service We shall update resources and technologies as well as serve clients with knowledge and sophisticated methods: We shall pursue benefits for clients and regard clients satisfaction as the ultimate aim.

To Produce Economic Benefits for Partners： We shall produce economic benefits for partners that stand together with us regardless of situation based on the win-win principle.

To Bring Happiness to Staff： We shall take employee benefits as the focal point and employee demands as central task. In addition, we shall attract outstanding talents with better salary, welfare, training system and good prospect and enable them to make success in career with pleasant feelings in spiritual and material worlds.

Sincerity： We re meticulous about every work and pursue perfection of each segment and procedure to ensure quality and safety. We treat our partner with sincerity and gratitude. We win trust and respect with genuine attitude. We undertake social responsibility to create a harmonious society

Integration： With international vision and open mind, we shall integrate the essence of ethnic characteristics and global marketing in corporate management and operation and promote corporate innovation and development following the human-oriented principle.

Unity：Carrying on the fine tradition of solidarity cooperation, respect and understanding. we are striving to forge a happy and harmonious team committed to mutual trust and support, learning and innovation.

Dedication： Carrying forward the good tradition of diligence and persistence, we shall always confront difficulties bravely and sincerely fulfill our duties, serving our country and customers heart and soul.

Enterprise qualifications and honors

130.Kilimall

Kilimall - Online Shopping in Kenya

Kilimall is Kenya's leading online shopping mall. It was launched in June 2014 with the mission of "Enriching Lives for Africa".

Kilimall serves a retail-customer base that continues to grow exponentially, offering products that span various categories including Electronics such as smart phones, laptops, subwoofer etc, Clothing, Home Appliances, Fashion bag, Baby Products, makeup and much more. Kilimall continues to expand the mall, with the scope of offerings that will increase in variety, simplicity and convenience.

The range of services are designed to ensure optimum levels of convenience and customer satisfaction with the retail process; order delivery-tracking, dedicated customer service support and many other premium services. The company is highly customer-centric and are committed towards finding innovative ways of improving the customers' shopping experience.

Now, shopping with Kilimall App will bring you millions of cash rewards, best customer services, and track your order at anytime. Download Kilimall App APK and enjoy best service online in Kenya.

Great Value

We offer competitive prices on our 10 million plus product range.

Worldwide Delivery

With sites in 3 languages, we ship to over 20 countries & regions.

Safe Payment

Pay with the world’s most popular and secure payment methods.

24/7 Help Center

Round-the-clock assistance for a smooth shopping experience.

Shop On-The-Go

Download the app and get the world of Kilimall at your fingertips.

131.Zonergy Corporation

Established in 2007, Zonergy Corporation (hereinafter as “Zonergy”) is a national level high-tech world-renowned enterprise specialized in smart microgrid integrated solutions. Zonergy is committed to providing first-class smart micro-grid solutions for global customers. After years of development, Zonergy has accumulated strong technical strength and engineering experience,

forming a first-class technology research and development, engineering implementation and market development team. This has enabled Zonergy to develop business in most regions across China and overseas countries, providing customers with premium quality microgrid solutions and services.
In recent years, relying on its own complete industrial chain, high-level R&D team and strong strategic alliance, Zonergy has ranked among top in the world in the construction scale of off-grid optical storage smart micro-grid projects, with industry leading technical solutions and implementation capabilities.
On this basis, the company has concentrated efforts in the field of optical storage smart micro-grid, successively established Zigong R&D and production bases and Shenzhen Research Institute, and established Chengdu Research Institute, focusing on the research and product development of sodium ion battery cells and BMS. Among them, Zigong R&D and Production Base covers an area of about 140 mu.

In 2020, it was awarded the "Sichuan Solar Energy Storage Smart Microgrid Engineering Technology Research Center" issued by the Sichuan Provincial Department of Science and Technology.

In 2021, the project was successfully selected as one of “Major Projects jointly-constructed by Sichuan and Chongqing”.
Zonergy has an existing product portfolio with four major categories including residential products, commercial products, large-scale power station products and photovoltaic module products. Products specifically include Residential Energy Storage Systems, Residential Off-grid Energy Storage, Integrated Solar Power Storage, On-grid Photovoltaic Inverters, Off-grid Photovoltaic Inverters, Residential Outdoor Off-grid Energy Storage, Portable Power Supply, Outdoor Commercial Energy Storage, Integrated Communication Power Supply, Utility Energy Storage System, PV modules, Carbon Crystal Wall Heater, etc.
Zonergy's products are designed in strict accordance with relevant international and domestic standards and have been certified by CQC, UN/MSDS, CE/CB, IEC, EN, VDE, CEI and other domestic and foreign authorities. Additionally, all products have passed the World Bank Lighting Global (Lighting Global) equipment partner certification. As of September 30, 2022, 182 intellectual property rights have been declared, including 39 invention patents, 100 utility model patents, 25 design patents, and 18 software copyrights.
Zonergy's industry status has seen rapid improvements. In 2021, it’s domestic shipments of energy storage system integrators ranked 8th; in 2022, it entered the top 30 innovative energy storage companies in China, ranking 24th, and ranking 17th in energy storage system integration companies.

Zonergy won the most influential enterprise award in China's energy storage industry in 2022, the new enterprise award of the 6th International Energy Storage Innovation Competition. Zonergy has a leading share in the market of nationally distributed photovoltaic demonstration projects, domestic off-grid photovoltaic storage projects, and large-scale photovoltaic ground power station projects. At present, Zonergy has invested in the construction of photovoltaic power stations with a total installed capacity of more than 1GW and an installed capacity of energy

storage exceeding 1GWh.

In the domestic solar power storage sector, Zonergy has invested in the construction of large-scale ground photovoltaic power stations in Inner Mongolia, Gansu and other western provinces rich in light resources.. Additionally, Zonergy also invested in the development of distributed solar power storage projects for industrial and commercial applications in Guangdong, Zhejiang, Jiangsu, Shandong, Tianjin, etc.

Among them, the Photovoltaic Power Generation Project in Zibo High-tech Zone, Shandong was listed in the first batch of “National Demonstration Zone for Large-scale Application of Distributed Photovoltaic Power Generation”. Additionally, the Photovoltaic Power Generation Project constructed in the High-tech Park Shenzhen was approved as a national “Demonstration Zone for Centralized Application of Solar Photovoltaic Power Generation”.

In the off-grid solar power storage sector, Zonergy took solving the people’s livelihood issues as its own responsibility and focused on participating in the “Three Year Action Plan of Comprehensive Power Supply Solutions for People Living in Areas with no Mains Supply” issued by the National Energy Administration, and became the leader in PV independent power supply project implementation, operation, and maintenance in China.

In practice, Zonergy deployed power supply projects in Sichuan, Gansu, Qinghai and other provinces and autonomous regions to solve the problem of no mains power availability for 453 thousand people living in 657 towns situated in 99 counties. In view of its outstanding performance in these projects, Zonergy was awarded the title of “Advanced Provider of Comprehensive Power Solutions for the People Living in Areas with no Mains Supply” by the National Energy Administration of the People’s Republic of China.

Among them, the “Power Project with Independent Photovoltaic Supply for Areas with no Mains Supply” in Sichuan Province was successfully selected into the “Demonstration List of Smart Photovoltaic Projects” jointly reviewed and released by six ministries and commissions in 2020.
In the international market for solar power storage and supply, the photovoltaic ground power station with a capacity of 9*100 MW in Punjabi, Pakistan was listed as one of the priority projects in China Pakistan Economic Corridor. The commencement ceremony of the project were jointly presented by XI Jinping, President of the People’s Republic of China and leaders of the Islamic Republic of Pakistan.

Among them, the on-grid project with the power of 3*100 MW commissioned in July 2016 with an annual power generation of more than 500 million kWh has become the largest Independent Power Producer (IPP) in the field of photovoltaic power station in Pakistan. This is the first on-grid power generation project and the first one yielding profits in the China Pakistan Economic Corridor. Due to the Zonergy’s outstanding performance, the project won the "Luban” China Construction Engineering Award.

In recent years, Zonergy furthered market exploration in Pakistan and customized integrated solar

power storage solutions for local residential, industrial and commercial customers. To present, Zonergy has opened nearly 73 flagship stores in Pakistan to provide one-stop services from equipment sales to project design and project implementation.

Several distributed solar power storage projects for industrial and commercial use constructed by Zonergy have been successfully connected to the grid. As a result, the market share of Zonergy in distributed solar power storage reached 30%. Meanwhile, Zonergy also developed a well-known new energy brand that is growing in popularity in Pakistan.

With a more efficient synergy of its complete industrial chain, continuous innovation and global market system, Zonergy will make further explorations in the field of smart micro grid, strive to be the first in green energy technology and the market, serve global customers with high-quality products and solutions, facilitate green energy transformation and build a greener and brighter future!

Zonergy Culture

Pursues balance and harmony between human and nature, and symbiosis between society and the environment, and is committed to becoming a responsible, environmentally-friendly corporate citizen helping reduce resource use.

While vigorously promoting R&D in new energy, energy conservation and environmental protection technologies, Zonergy earnestly implements the national strategic deployment, integrates the elements of green environmental protection into its strategic management, implements the concept of resource conservation into the main links of its daily production and operation, and vigorously carries forward the culture of environmental protection and conservation.

We hope to influence more people through our own advocacy and behavior, personally promote all industrial partners, maximize rational use of the earth's resources, minimize impact on the environment and ecosystems, and jointly create achievable and sustainable green development.

Qualifications and Honors

Social Responsibility

Zonergy has always advocated and pursued the development concept of “from society, to society.” While strengthening organizational capacity and improving management efficiency, we also pay attention to the environment and society, and actively fulfill our corporate social responsibility. We continue to participate in public philanthropy and transmit positive energy from enterprise development!

Production workshop

Strong R&D and Production Capacity

1 billion +The Output Value

1 GWh +The Storage Production Capacity

3 GWh Sodium-ion Battery Cell Planning Product Capacity

132.BGRIMM GROUP

Founded as a research institute in 1956 and transferred to be a technology enterprise in 1999, BGRIMM Technology Group (renamed on 29 December 2017 from former BGRIMM , hereafter

referred to as BGRIMM Group) is directly administered by the State-owned Assets Supervision and Administration Commission, the country's top SOE regulator. As one of the earliest technical innovative enterprises in China, BGRIMM Group has been playing an active role in scientific research and technology development in the field of comprehensive utilization of mineral resources in China.

Currently BGRIMM Group is China’s premier comprehensive research and design institute in mining and metallurgy, with three core businesses of technical and engineering services in mineral resource development and utilization, advanced material technologies and products, and mineral resources recycling and environmental protection, that is supported by its well-integrated strengths in R&D, engineering and product manufacture capabilities. BGRIMM Group represents the national level in such fields of mining, mineral processing, metallurgy, process mineralogy, industrial explosives, processing equipment, surface engineering technologies and related materials.To date, BGRIMM Group has completed more than 6700 research projects, won more than 1200 engineering design awards of China, including more than 100 national-level awards, and registered more than 1100 patents.

Utilizing its class-A qualifications in engineering design, engineering consulting, environmental impact assessment and environmental engineering consulting, as well as safety evaluation, BGRIMM Group has undertaken more than 600 engineering design, consulting and contracting projects, the achievements of which have won more than 70 provincial and ministerial level awards. Currently BGRIMM Group takes pride to be a top-ranking engineering company in China, growing from a conventional engineering services provider to a solution provider integrating process development, multi-disciplined engineering, equipment supply and EP/ EPCM /EPC etc tailored to verified requirements of mining companies at home and abroad.

Granted a license by the central government for direct import and export operation in 1993, BGRIMM Group has been actively promoting its technology and product exports to more than 40 countries and regions, including USA, Canada, Germany, South Africa, the democratic republic of Congo, Chile, Peru, Russia, Kazakhstan, Australia, Japan, South Korea, Mongolia and etc.

BGRIMM Group explores an innovative path for its transformation from traditional research institute to an enterprise with customer insight and market orientation through consistent efforts to commercialize its R&D achievements in mining machinery, instrumentation, civil explosives and blasting engineering, mine chemicals, metallic powder, battery materials, magnetic materials. guar gum and related equipment.

Establishing administration, R&D and production bases in Beijing and six other provinces, BGRIMM Group operates a network of 11 research institutes and 24 wholly-owned or holding companies, two of which are public listed companies. BGRIMM Group is staffed with more than 3400 employees, over half of them are technical professionals with master's degrees or doctorates, including three academicians of the Chinese Academy of Engineering, 86 senior experts receiving special government allowances, and 11 young experts with outstanding

contributions to the State.

With over-sixty-year’ s experience and capabilities of “Turn-rock-into Gold”, BGRIMM Group strives to inherit and promote China’ s long-heritage mining and metallurgy technology and cultivate its brand culture. Guided by a global perspective and high-end development strategy, BGRIMM Group is committed to making new contributions to the mining and metallurgy industry with the vision of “Being a global leading technology group in mining and metallurgy industries”.
Our Vision
To be the leading global technology group in the mineral and material industries
Our Mission
To promote sustainable development and utilization of mineral resources
Our Core Values
Turn Rock into Gold
Win-Win with Client
Our Core Business
Technical and engineering service in utilization of mineral resources
Advanced material technology and products
Mining, mineral processing, metallurgy and secondary metal recycling
133.Henan Guoji Industry Group Co., Ltd.

Henan Guoji Group was founded in October 1994, which was originally called No. 5 Construction and Installation Company in Jinshui District, Zhengzhou, then changed to Henan Guoji Construction and Installation Company. It is an international corporation mainly engaged in construction and installation, real estate development and overseas development, supplemented with large-scale property management, greening and landscaping, project decoration. There are many subsidiaries, such as Henan Guoji Property Co., Ltd.(founded in 1995), Henan Guoji Decoration Engineering Co., Ltd. (founded in 1995), Henan Kanglv Garden Greening Co., Ltd.(founded in 1996), Henan Guoji Construction Group Co., Ltd.(founded in 1997), Henan Guoji Property Management Co., Ltd. (founded in 2001),Henan Guoji Industry Group Co., Ltd. (founded in 2003). The group has 36 domestic branches and 13 overseas subsidiaries. Henan Guoji owns the National Premier Class Certification for House Construction and General ....
Relying on domestic and international market, innovating cooperative business mode
Assembling domestic and international resources, achieving collection of production factors.
ADVANTAGE
Brand Call
Henan Guoji is a professional company with more than 20-years experience in real estate development. We have outstanding management team and rich experience for the preliminary market research, project orientation, project planning and project management. At the same time, we enjoy nearly 15 years’ experience in overseas project operation.
Superior Quality
Henan Guoji owns the National Premier Class Certification for House Construction and General Contracting and the qualification for overseas projects contracting, and ranks the 29th among the Top 100 construction companies in China. We are awarded Luban Award for the Engineering Projects in China (for national projects with superior quality) for many times.

Outstanding Team

Henan Guoji is in charge of a Class A Engineering Design Institute, which is an institute with the highest qualification for designing in China, and a designers team which is stationed in different countries in Africa for long term as well as more than ten years' project operation experience in dozens of countries and the professional team for project construction and management.

Capital Policy

Henan Guoji has set up the good cooperation relationship with the local government in Africa countries when Going Out. Henan Guoji Group possesses strong capital strength and financing capability, with China-Africa Development fund being one of its shareholders. In addition, China Development Bank as well as Industrial and Commercial Bank of China are its strategic partners.

134.Jiangsu Overseas Group Co., Ltd.

Jiangsu Overseas Group Co., Ltd. (JOC) was established with the approval of Jiangsu Provincial People's Government. It is the first enterprise engaged in international trade in Jiangsu Province (since 1984) and the first enterprise to invest abroad (since the establishment of Zhongshan Company Ltd. in Hong Kong in 1985). Now JOC has developed into a comprehensive enterprise group with an annual turnover of over 21.7 billion yuan and a total import and export volume of nearly 2.7 billion US dollars.

JOC focuses on the two high positioning of building a leading enterprise in high-end commodity import and a high-level integrated 'go global' service platform in Jiangsu.It is devoted to trade, chemical warehousing logistics, overseas investment and financing services and it has three high-quality growth (namely food industry, freight trains platform, and financial services).

Trade

By taking advantage of its long-term overseas market layout and centering on the core function of building the supply chain system of scarce materials, JOC aims at the important raw materials of modern industry and national defense industry with high external dependence, such as chromium manganese ore and natural rubber, as well as high-end meat food, in order to meet the strategic reserves and people's livelihood consumption needs in Jiangsu. JOC shoulders the responibility of state-owned enterprises in ensuring the security of national and provincial energy, resources, food and other strategic materials supply chain. JOC's beef, mutton and Vietnam rubber imports volume ranks in the forefront of the country for many years and its chromium and manganese ore imports volume maintains on the top of Jiangsu.

Import Trade

The imported products of JOC mainly include industrial raw materials and energy products, equipment and food. Raw materials and energy products mainly include non-ferrous metals, iron and steel products, chemical raw materials, textile raw materials, wood, light industrial raw materials and chemical intermediates. Equipment include urban transportation, textile machinery, medical devices, marine equipment, municipal infrastructure, complete sets of equipment, etc. Imported food mainly includes meat products.

Export Trade

The export products of JOC mainly include textiles and clothing, light industrial products, biomedicine, chemical pesticides and new energy chemical raw materials, pet food and products, complete sets of equipment, etc.

Overseas Enterprise

JOC has established a number of overseas institutions in Hong Kong, Macao and Belarus, and 5 production bases in Myanmar, Cambodia and Tanzania. It led the establishment of a fully licensed China Dasheng Bank in Tanzania. JOC ’ s autonomous and controllable garment factories in Southeast Asia can produce more than 12 million pieces a year. JOC has successfully completed Belarusian beef’ s state, enterprise and product access and officially imported the beef into the Chinese market in 2018.

Corporate culture

Overseas Enterprise

JOC has established a number of overseas institutions in Hong Kong, Macao and Belarus, and 5 production bases in Myanmar, Cambodia and Tanzania. It led the establishment of a fully licensed China Dasheng Bank in Tanzania. JOC ’ s autonomous and controllable garment factories in Southeast Asia can produce more than 12 million pieces a year. JOC has successfully completed Belarusian beef’ s state, enterprise and product access and officially imported the beef into the Chinese market in 2018.

135.TBEA

TBEA: a service provider of systematic solutions for global energy industry

TBEA Group is committed to providing clean energy solutions for the global energy industry. It is a national high-tech enterprise and a large-scale energy equipment manufacturer in China, and has successfully built three listed companies——TBEA (600089SH), Xinjiang Joinworld (600888SH) and Xinte Energy (HK1799). It has cultivated three strategic emerging industries including high-end equipment manufacturing for power transmission and distribution, silicon-based new energy industry, and aluminum-based new materials industry. It boasts 21 manufacturing industrial parks in China and 3 bases overseas. Its annual output of transformers has ranked the top in the world for years running, and the silicon and aluminum based new materials have entered the first rank of the international supply chain. Its total installed capacity of PV EPC also takes a leading place worldwide. It has ranked among ENR's top 20 Chinese enterprises for six consecutive years.

1000kV Shanxi Southeast – Nanyang – Jingmen extra-high voltage model project.

Pakistan 100 MW photovoltaic power station constructed by TBEA

New materials such as electronic aluminum foil and electrode aluminum foil are used in the field of rail transit, electronic information technology and automotive lightweight fields etc.

2×660MW Zhundong Power Plant to transmit Xinjiang electricity outside.

Distribution of TBEA industry parks

The Company has 21 manufacturing industry parks in China and 3 bases abroad.

Road of innovation for the preparation and manufacture of transmission and transformation (equipment)

It records the history of manufacture that we inherit China’ Transformer; from the birth of the first transformer in TBEA Shenyang till now, China’ Transformer has a history more than 80 years; as a participant, TBEA runs all the way up to now from the first equipment in China to the first one in the world.

The development of TBEA has won high praises along the Belt and Road.

On December 30,2020, TBEA and the Republic of signing theGambia held an "online$ 23.05million ceremony for power Transmission and transformation project.

On Oct.14,2016, Mr Xi Jinping, the President of China and Sheikh Hasina the Prime Minister of Bangladesh together witnessed the contract signing between TBEA and Dacca Distribution Company in Bangladesh on smart grid and upgrading project for Dacca, the capital of Bangladesh,amounting to over111 biltion RMB.

On November 21， 2017, TBEA started the construction of the 115kV power transmission and transformation project in Vientiane,thecapital of Laos. The signing of the project is to further implement the important achievements of President Xi Jinping's visit to Laos and solidly promotes the construction of the Belt and Road Initiative”.

Honors and qualifications

Ranking No. 228 in the World's top 500mechanical companies

Ranking No.313 in China's top 500 enterprises

Ranking No.9 th in China Top 500 Machinery Manufacturers

Ranking No.109 in Top 250 International Contractors listed by ENR

The Company invests 4% of yearly sales revenue into technological breakthroughs and innovations, and has built China’s unique UHV Transformer Engineering Technology Research Center which has become the innovation platform for R&D and manufacture together with the existing State-level Engineering Laboratory in the areas of power transmission and distribution, new energy and new materials, Enterprise Technological Center, Post-Doctoral Scientific Research Workstation and Academician Workstation. By undertaking more than 100 research and innovation projects in such national programs as National 863 High-tech Plan, key research and development plans and major local special projects, the Company has made technological breakthroughs in as many as 126 independent researches. The Company has so far successfully applied for more than 2012 independent intellectual property rights, technological secrets, and software copyrights. The Company has also participated in formulating more than 210 domestic or world industrial standards.

The Company has won numerous prizes for its scientific and technological achievements, among which are 2 Grand Prize of the National Science and Technology Progress Award, 4 First Prize of the National Science and Technology Progress Award,3 Second Prize of the National Science and Technology Progress Award, 9 Grand Prize of Science and Technology Progress Award of China’s Machinery Industry and more than 200 relevant industry or provincial awards.

TBEA’s produces power transmission and transformation equipment whose scope covers transformers, cable&wires, converter valves, switches, secondary equipment and bushings which integrated whole industry chain in power transmission and transformation area. whose products are widespread in power grid, new energy, high-speed train, subway, petrochemical, big data center, intelligent manufacturing and other field. Meanwhile, in more than 30 countries, TBEA provided system solutions and turnkey projects which including survey, design, construction, installation, commissioning, and training to operation and maintenance.

TBEA owns opencast coal mine with reserves of 12.6 billion tons in Wucaiwan, Zhundong Area, Xinjiang， and constructed industry chain which covers coal resource, coal power, and railway logistics.Built a production system for smart and green mines. The mine has obtained an approved production capacity of 54 million tons a year. The coal mine lay a foundation for TBEA's renewable energy and polysilicon industrial chain and aluminum-based new material industrial

chain.
International Cooperation
TBEA is always commited to sharing China's power construction experience with the world, and actively carry out Electric energy science and technology coperation with neighboring countries.Since 20, it has provided high-end equipment for more than 60 countries such as USARussia,lndia, Brazil, etc. and provided intearated solutionstoverin survey and esion, enaineernd onstruction, eauioment supoy, insta lation and commissionino. operation and mantenance.and trainina serices for laikstan.rgyzstan, Pakistan, lran, Saudi Arabia in Asian, Zambia, Kenya and Togo in Africa.
136.SANY
Since 1989, SANY’s vision to build a first-class enterprise, to foster first-class employees, and to make first-class contributions to society has never wavered.

We have focused on delivering value to customers, employees, shareholders, and the public by offering high-quality products and services, providing a work environment full of opportunities, releasing financial reports to meet investor expectations, and exerting our own influence to help those in need.
What We Do
In the past three decades, we have expanded our business to a wide range of areas, including construction and mining equipment, port machinery, oil drilling machinery, and renewable wind energy systems. We keep exploring more possibilities to expand our reach and innovate our way of doing business as well as to raise the standard and answer the call for new market needs
SANY’s Footprints
In 1986, Liang Wengen, Tang Xiuguo, Mao Zhongwu, and Yuan Jinhua founded Hunan Lianyuan Welding Material Factory in Lianyuan, which was officially renamed SANY Group 5 years later.

In 1994, SANY independently developed China’s first high-pressure, large displacement, truck-mounted concrete pump.

In more than 30 years of innovation, SANY has become one of the largest construction equipment manufacturers in the world.

Now, we are diversifying our business as a corporate group by setting foot in new fields like energy, financial insurance, housing, industrial internet, military, fire protection, and environmental protection.
Working on the Future
SANY is a forward-looking company that was born in a time of change and now looks for any opportunity in those changes that will lead the way to the future.

We are unleashing the power of data in the connectivity of machines, realizing smart manufacturing and autonomous operation with some models.

The efforts to add a new electric product portfolio makes SANY stand out in today’s digital world and is also part of our sustainable solutions.

At SANY, we put research and development at the core of what we do to search for new ways to make our products better.

Intelligent manufacturing

In line with Industry 4.0, SANY is committed to developing intelligent manufacturing that applies the internet to the whole manufacturing process.

•Investing 5 – 7% of annual sales revenue in R&D each year
•More than 7000 SANY employees involved in R&D
•Intelligently linking people, technology, and adaptation
•Growing productivity and efficiency

Sustainability development

SANY embraces new technology for greater efficiency, increasing sustainable performance.

•Reducing our impact on the environment
•Building smarter machines
•Creating new energy production
•Delivering increased performance

New energy development

SANY has been proactively exploring the new energy industry for the sustainable development of human beings.

•A society where people can live and work with peace of mind
•A society where people can enjoy safe and plentiful food
•A society that offers an exciting life filled with rich and fulfilling experiences
•An energy-saving society

AR, VR, & holograms

SANY turns heads with new immersive technology.

•Embracing new technology to train, educate, and empower our teams
•Sharing knowledge with our teams worldwide
•Promoting our brand through new digital communication
•Celebrating our success through interactive learning

Sustainability at SANY

SANY contributes to society by both providing quality products and fulfilling our corporate social responsibility centered on achieving sustainable development of the industry, society and individuals.

Sustainability Strategy

We are at a critical moment in human history, where every one of us, by making choices today, can decide what the world we live in will be like tomorrow. As a responsible, visionary and aspiring global enterprise, SANY supports the UN 2030 Agenda for Sustainable Development. In all decision making processes, we always take into consideration global trends and challenges, international norms of responsible business behavior, and stakeholders’ expectations.

Sustainability Framework

Carbon Neutrality

Against the backdrop of a low-carbon future featuring a carbon emissions peak and carbon neutrality, SANY is now leading the wave of electrification in responding to the call for an electrical era that is widely echoed across the industry. SANY has launched more than 30 new energy products, covering three major energy supplement routes of pure electricity, electricity

exchange and hydrogen fuel
Renewable Energy
SANY Renewable Energy explores the low-carbon development of the manufacturing industry through developing intelligent, renewable-energy products including wind power products and photovoltaic power plants.
People
The Beijing SANY Foundation (SANY Foundation) is a funded foundation initiated by the SANY Group and was established on December 31, 2013 in the Beijing Municipal Civil Affairs Bureau. The SANY Foundation has a mission to promote science and public welfare by empowering the public to fully realize their potential, thus bringing real change to the world and building a vibrant, well-intentioned and trusted society.
137.BGI Genomics
BGI Genomics is the world's leading integrated solutions provider of precision medicine, now serving customers in more than 100 countries, involving over 2,300 medical institutions.
Relying on cutting-edge sequencing and bioinformatics technology, we provide our customers with expert and affordable clinical molecular diagnostic solutions and high-throughput sequencing (NGS) research services.
100+
Active in 100+ countries
1%
Sequenced 1% of the original Human Genome Project
150000+
150,000+ human whole genomes sequenced to date
1. Job Purpose & Principal Accountabilities:

Develops strategies for increasing sales revenues and marketing objectives.
Effectively promoting the company and its merchandise and targeting the correct and appropriate audience.
Manages and develops marketing programs and materials such as advertising, event support and online promotions.
Retain, extend, and acquire customers through effective identification of opportunities and specific needs for our industry
Build ongoing knowledge of current industry trends, competitive information and industry/technical knowledge and disseminate that information with company colleagues
Develop and maintain relationship with potential customers, accelerate follow-up opportunites
Maintain records with customer information, including sales
Organisation:

Report to the regional sales manager.
Work closely with the product and sales managers to promote product development through collaboration with key customers.
Understand BGI’s values and expand BGI’s vision in the markets.
Key Challenges:

Think outside of the box and implement alternative strategies to prospect business opportunities and stand out from the competition.
Market and business awareness: Prove the value of BGI’s products and services.
Offer value to the client: get a deep understanding of the client’s needs and pain points.
Time management: create a solid selling message and reach out.
Job Knowledge, Skills and Experience:

In-depth knowledge NGS, IVD and translational markets and of customer needs in this industry.
Knowledge of technical and marketing skills including professional, impactful presentations
Ability to demonstrate both clinical and sales aptitude
Strong marketing and business development skills
Excellent communication, analytical and organizational skills.
Willingness to travel.
Fluency in English and Mandarin languages
Competences:

Significant past experience in similar positions, at least 3 years of experience in customer management in the biotechnology industry.
Degree in Master’s or above in biology, biochemistry, molecular biology, bioinformatics or equivalent.
138.Hangzhou Chief Technology Co., Ltd.
Your Health, We Care
The icon of the Hangzhou chief Technology is derived from the national treasure "China's First Lonng" more than 6000 years ago. Its powerful galloping shape makes people feel the magical power of lonng traveling through space and omnipotent. The lonng is the soul of the Chinese nation's spirit, and the surging, fearless lonng is the spiritual symbol of the Hangzhou chief Technology. The logo visually integrates the first Chinese lonng and the first and last letters C and F of the chief. Like a giant lonng about to emerge from the water, it is vigorous and rising, showing Chief’s five thousand years of rooted Chinese culture, combined with high technology, and strive to become a modern multinational group.
Chief Culture
Our mission
Let every employee, customer, shareholder and business partner of Chief live a better life.
Our vision
Promote the industrialization process of developing countries with Chinese intelligence.

Our strategy
Localization,Platformization, branding,channelization.
Core value: Kindness, Mutualism, Self-discipline, Innovation, Integrity.

- Kindness: The public, the society, mutual development.
- Mutualism: Benefit customer, benefit ourselves, sharing together.
- Self-discipline: Self-discipline, self-contribution, complying with rules.
- Innovation: Never stop to learn and expand.
- Integrity: Treat people sincerely, keep commitment strongly.

About Chief Group

In 2003, chief Group 's predecessor, Mali CONFO Co., Ltd., was founded in Africa. It was a council member of the China-Africa Chamber of Commerce. Its business currently spreads to more than 30 countries and regions in the world. Besides, it has subsidiaries in more than ten countries in Africa and Southeast Asia.

Based on traditional Chinese culture, chief Group 's predecessor 's regards sustainable development as the premise and aims to bring cheap and fine products to consumers. It has R&D institutions and production bases in many parts of the world, introducing excellent technology and management experience of China into local areas and developing together with local people. At present, the BOXER and PAPOO series of household chemicals produced by its subsidiary Boxer Industrial, CONFO and PROPRE series of health products produced by CONFO, OOOLALA, SALIMA and CHEFOMA series of delicacies produced by Ooolala Food Industry have become well-known local brands.

Remaining true to the original aspiration while being filled with love, chief Group established the chief Group Charitable Funds and set up chief Group scholarships in some colleges and universities to give back to the society with love.

CONFO Group represents strength and courage, and it carries the spirit of never yielding and never giving up of the Chinese nation. We will inherit the spirit of “Kungfu” and devote ourselves to promoting the industrialization process of developing countries with Chinese culture and advanced productivity, and will work hard for the health and beauty of people all over the world.

Our Advantage

Professional management team

20 years’ experience in international brand operation and management.

Vast product group

More than 20 patents, 4 mature brands renowned in the international market, trademark and patent registration have been completed in more than 100 countries and regions.

Stable product quality

Advanced production technology, strict product inspection and professional supplier audit system provide a guarantee for production of high-quality products.

Perfect product service

It has 15 direct sales branch companies, over 100 agents and hundreds of thousands of retail terminals all over the world, conducting brand marketing and maintenance all over the world.

Qualification Certificate

Trademark registration certificate

Factory

Nigeria Mosquito Coil factory

Confo Healthcare Product Factory

Boxer Mosquito Coil Factory

Insecticide Aerosol And Airfreshner Factory

Products

Healthcare Product Series
Refreshing & Cool
Anti-Pain & Pain Relief
Anti-insect Product Series
Mosquito Coil
Insecticide Aerosol
Household Product Series
Papoo Detergent Liquid
The PAPOO Flame Gun
PAPOO MEN Shaving Foam
Grand Launch Of Our New Product: PAPOO MEN BODY SPRAY
Refreshing Home Car Washing Room Papoo Air Freshener Spray
Anti-Broken Papoo Home Use Adhesive Super Glue(Gel 3.5)
Anti-Broken Papoo Home Use Adhesive Super Glue(Liquid 3g)
Yummy Food Product Series
SPICY CRISPY
The CHEFOMA Spicy crispy adopts traditional Chinese food technology, 3 minutes constant temperature frying, fast deoiling and greasy, heat packaging, multi-process elaborate production, the process of production retains the flavor of rice, the taste is crisp and refreshing, long time eating is not greasy, restore the taste of childhood. The crispy taste is worth the aftertaste. Experience better mouthfeel and savor with endless aftertaste. Nowadays it is very popular in China, South Asia and Africa.
SPICY TWIST
CHEFOMA spicy Twist is a traditional dish in northern China. A crisp filling containing osmanthus, min ginger, melon and other special ingredients is sandwicked between white strips and forest strips, so that the fried twist flowers are soft and sweet and distinctive. The mixed stuffed hemp flowers are fragrant, crisp, crisp and sweet, and will not go stale, soft or bad when placed in a dry and ventilated place for several months.

The appearance of twist flowers is transparent and golden, rich as wax and clear as crystal. A variety of small materials of natural aroma ratio fusion, accurate temperature control after the activation of the compound fragrance, the traditional process to create crisp flavor, crisp but not hard, crisp and unremitting, endless aftertaste. Appearance uniform full, compact and orderly, loose and untiring, filling overflow and not scattered.
Nutrition Salima Egg Cake Bar
Company History
2003
Founded Mali CONFO Co., Ltd. to create a business base in Mali
2004-2008
Set up Mali CONFO Mosquito-Repellent Incense Factory and Mali Huafei Slipper Factory to create business bases in Burkina Faso and Cote d'Ivoire.
2009-2012
Defined strategic layout and business model of products, and created business bases in Guinea, Cameroon, Congo-Brazzaville, Congo, Togo, Nigeria, Senegal, etc.

2013
Founded Hangzhou Chief Technology Co., Ltd. to build a headquarters security system.
2016
Confirmed the company' s first five-year plan, further defined development strategy of the company, and started to prepare to build food factories and household chemicals factories in many places.
2017
Settled in Binjiang HuanYu Business Center in Hangzhou, starting a new journey
2019-2021
set up Tanzania branch, Ghana branch and Uganda branch,participate in the preparations of the ZheJiang-Africa Service Center.
Until to 2022
Chief group have more than 20 companies worldwide, now we are writing new African stories for enterprises.
Our Exhibitions
Distinguished Guests Visit
139.ZXAUTO
Hebei Zhongxing Automobile Co., Ltd.'s (ZXAUTO) predecessor was founded in 1949 and in Dec 1999 transformed into a joint venture company with an assets of CNY3.0 billion, integrating passenger vehicles, commercial vehicles and auto-parts production. It was the birthplace of the very first Chinese made pickup truck with its own intellectual property rights and is rated as China's the pillar enterprise on multi-purpose vehicles, the export base for national automobile, the national advanced technology corporation.

The corporation possess 3 main global production bases - Hebei Zhongxing, and overseas KD plant, and R&D centers.

Baoding production plant' s core production are pickup vehicles and has an annual production capacity of 50,000, fully supported by 4,000 personnel. The main production lines are fully computerized, starting from raw materials entering the workshop to finished built-up vehicles within the plant.

The Baoding base' s R&D product center comprise numerous scientific and technological elite which possess strong whole vehicle development ability and has successfully developed high-end pickup such as ZXAUTO GrandTiger TUV and ZXAUTO Admiral which has won the consumers recognition and the general public alike. These pickup models are regarded as the main pickup product for governmental and commercial uses.
ZXAUTO is the brand known for pioneering in automobile export, and the first corporation in the industry to realize large order export. It is the first national automotive products 3C certified, achieve ISO9001 Quality Management System certification, US RAB certification, Chilean certification, Iranian certification, GCC export certification and UNECE homologations. The China Ministry of Commerce and National Development And Reform Commission appraise ZXAUTO as "Designated Exemplary Enterprise on China Overseas Trade Credit System" and "China National Whole Vehicle Export Base Enterprise". ZXAUTO pickups is one of the most exported

units in the same pickup category, and it is also one of the earliest Chinese national pickup brand which is able to compete against other International brands in Libya, Iraq, Syria, Iran Ecuador, Chile, Peru， Bolivia.
ZXAUTO has nearly 200 suppliers from home and abroad and established a high-level platform for global auto-parts supply. ZXAUTO strictly controls every aspect of the value chain, the various process from auto-parts production, procurement and integration, adopting a 360-degree quality testing and control so as to ensure the quality of vehicle meets the design objectives and customer needs.

ZXAUTO has more than 300 dealers in China and with nearly 400 service stations.ZXAUTO products are sold in more than 90 countries and regions, facilitated with a 24 hours call center in providing personalized service to consumers. In servicing sector, the dealers, the service stations and ZXAUTO consultations comprising the 3 areas for customer feedback and care service, assisting customers in understanding and familiarize ZXAUTO, taking the initiative in eliminate any hidden issues for the customers, increase the product effective life and to assure and maximize customers interests.

Hebei Zhongxing Automobile Co., Ltd. R&D Center consist of more than 300 technical personnel composing the high-end quality development teams, of which 17 are Senior Research Fellow, 70 Senior Researcher. 50% of the team are equipped with more than 10 years in whole vehicle development and practical experiences.

The R&D Center is instituted with whole vehicle, chassis, electronics, car body, car body accessories, mold engineering, modeling design, product experiments, technical management and relevant project management. Possessing sound technology development program and complete management system. Implementing matrix management, establishing market-oriented product development system and fully utilize domestic and overseas resources to joint venture with well-known international design firm in product design and self-development.

The corporation is focus on new product development and technology enhancement and has gradually increase the investment into the technology development funds. On 2015, more than CNY100 million was invested into the technology development funds, and has since introduced and applied 3D design development software system graphics workstation.

The product modeling development workstation and 3 coordinate measuring system were available at the same time. Able to complete the entire whole vehicle development process independently starting from modeling graphic effect design, clay modeling, 3 coordinates measuring, engineering design, CAE engineering analysis, etc. An electronic information technology system was fully established, realizing the digitized of the entire design process.

ZXAUTO upholds the "Perseverance, Exploration" entrepreneurial spirit, idealizing"Industry ServingThe Nation" and the spirit of "Benevolence ", through close partnership with suppliers and distributors in providing customers with value, social responsibility, revitalizing the national automotive industry.

MANUFACTURING PLANT
The Press Shop
The Welding Shop
The Painting Shop
The Assembly Shop
Research and Development
Excellence In Construction
The research centre of Hebei Zhongxing Automobile Co.,Ltd includes 150 engineers, including 17 senior researchers and 70 researchers. About 50% of our researchers have more than 10 years of experience related with vehicles.

Our motor research centre is grouped into such departments as chassis, body, appendix, model engineering, design, technical management, program management, etc. Our Research and Development Group works closely with our Marketing Group to make full use of our domestic and foreign resources. We also co-operate with some famous foreign design companies.
Research And Development

ZXAUTO pays strict attention to new model vehicle research and technology. 1,000,000,000 CNY was invested in Research and Development (2005 figures) We also use three dimensional research software such as CATIA, UG, Alias as well as two dimensional software as AutoCAD, Project, Visio, Office. We posses our own vehicle model study office and coordination system. We also own our own Automated vehicle model development studio and three dimension measuring system. We are able to draw and design the vehicle model, make the full scale models. With our digitilized design process allows us to make three dimensional measurements within our engineering design process. We meet and / or exceed CAE engineering specs.

ZXAUTO's scientific, integrated development process, ensures the high quality of our product line. We have achieved an excellent union between art and engineering, with a continuously innovative conceptual design. Our research models are all finished with a powerful three-dimensional engineering system. We design all of our vehicles and vehicle components. Within our research facilities we build actual full size functional test model vehicles. This allows us to test design specifications in real world conditions, so that our product is of the highest quality that we can deliver to our customers all over the world.
Corporate Culture
Core Value
Value creator oriented
Corporate Vision
To be a respected century-old company
Corporate Mission
Industry serving the country as its mission
Corporate Spirit
Perseverance, Aspiration, Collaboration
Strategic Notion

Based on commercial vehicles, developing passenger vehicles, focusing on special vehicles, and deploying new energy vehicles

Management Philosophy

To be a century old brand

Sales Network

The world map

140.SDHS

Shandong Hi-Speed Group Co., Ltd. (SDHS), headquartered in Shandong Province, is a state-owned capital investment company with registered capital of CNY45.9 billion and total assets of over CNY1.4 trillion. Relying on its core strengths in infrastructure development, SDHS is committed to building a world-class comprehensive infrastructure service provider with global competitiveness and providing powerful support for making Shandong a province with strong transportation network.

Currently, SDHS is operating and managing 8,311-kilometer motorways in China, including 6,702 kilometers in Shandong, accounting for about 83% of the whole province. SDHS owns five listed companies, namely, Shandong Hi-Speed Limited (600350. SH), Shandong Hi-Speed Road & Bridge (000498. SZ), CHINA SDHS FIN (0412. HK), Qilu Expressway (1576. HK) and Weihai City Commercial Bank (9677. HK). It has obtained domestic AAA and international A credit rating, and has been ranked among the "China's Top 500 Enterprises" for 14 consecutive years.

SDHS shall fully implement the decisions and deployment of the Central Government and the Shandong Provincial Government, and fulfill its economic, political and social responsibilities as a state-owned enterprise diligently, striving to achieve high-quality transformation and development, industrial layout optimization and structural adjustment as well as deeper reform and innovation. Aimed at speeding up the construction of industrial chain, supply chain, value chain and innovation chain with outstanding advantages and distinctive characteristics, and making new and even greater contributions to a strong and modern province in the new era.

Concept of Social Responsibility

Shandong Hi-Speed Group, committed to the people-oriented principle, highlights the main responsibility and business and tries to achieve high-quality development in order to provide premium services for the public, create value for society, increase revenue for enterprises, bring welfare to employees and make new greater contributions to the construction of better human homelands.

Lay Emphasis On Quality And Build The Brand

When undertaking the construction of projects, Shandong Hi-Speed Group consistently regards the quality of the project as the foundation of business and establishes its own local corporate image through a series of quality projects. As a result, the company has won extensive praise among proprietors, supervisors and users and thus its brand reputation has been enhanced simultaneously.

Stick To Green Development And Ecological Protection

The company focuses on the harmony of nature and the community, holding the concept of low carbon and environment protection to take good care of lucid water and the blue sky. In addition,

the company strictly conforms to local regulations on environment protection and integrates the concept of environment protection into the links of the design, management and construction of the project.

Benefit Local Places As A Responsible Company

With regard to project construction management, staff management and local public welfare, the company aspires to benefit society through active conduct. Moreover, acting as the envoy of Chinese cultural transmission, the company tries to build friendly image of China and conduct project construction beneficial to local people in a down-to-earth manner so that it has gained extensive support.

Talent Concept

Human resources are the most crucial resources of the company. Each employee is as valuable as gold.

[Make Full Use of Talents]

Give employees opportunities and stages to develop their potential and promote their development.

[Employ Employees According to Their Abilities]

Build an open and transparent talent mechanism to encourage employees to give full play to their talent. Use the professionals reasonably, cultivate the all-round persons and perfectly appoint talented persons.

141.Hengtong Group

Hengtong Group is an international enterprise with a diverse range of expertise covering fibre optical communication, power transmission, EPC turnkey service and maintenance, as well as IoT, big data, e-commerce, new materials and new energy.

Hengtong is the largest Optical Fibre and Power Cable manufacturer in China, ranking among the world’s top 10 issued by Integer. The company is ranked among the world’s top 3 producers of optical fibre communications products, and supplies approximately 25% of the domestic market volume and 15% of the international market volume.

Hengtong has over 70 wholly-owned companies and holding companies (5 of which are listed on the Shanghai, Hong Kong, Shen Zhen and Indonesian stock exchanges respectively), with 12 manufacturing bases in Europe, South America, Africa, South Asia and Southeast Asia. Hengtong operates sales offices in over 40 countries and regions around the world, supplying products to over 150 countries and regions.

Committed to innovation and social responsibility, Hengtong is steadily transforming to intelligent manufacturing, with the aim of becoming the most advanced systematic integrator and internet service provider in the world.

Social responsibility is regarded as the first responsibility of Hengtong. It has established the Hengtong Charity Foundation and has donated over 700 million Yuan to a wide range of causes.

Global Operation

HENGTONG possesses 70 wholly-owned companies and holding companies (5 of which are listed on Shanghai Stock Exchange's main board, Singapore, Hong Kong, Shen Zhen, and Indonesia), establishes industrial bases in up to 16 provinces of China and in Europe, South America, South Africa, South Asia, and Southeast Asia, and marketing technological service branches in over 40 countries and regions around the world, supplying products in over 150 countries and regions.
Sustainability
Creating Excellence, Advocating Civilization

HENGTONG takes "creating excellence, advocating civilization" as the enterprise objective , and takes " integrity , gratitude , responsibility and equality" as the enterprise ethics. It owns law-abiding business and pays taxes in accordance with the law, and highlights green development. Besides, it adheres to the cyclic and low carbon development concept, and actively participates in social and charitable causes so as to realize the sustainable development of the company as well as the harmonious development of the society as a whole.

5. 67
Contribution value of 5.67 yuan per share
700
Charitable donation of more than 700 million RMB
7356
Saving 7356 tons of coal annually
20000
Arrange employment of 20000 people
HENGTONG Charity Foundation
Directly administrated by National Ministry of Civil Affairs, First in Jiangsu Province Non-public charity foundation initiated by private company.
CSR Concept

For HENGTONG, Corporate Social Responsibility (CSR) is not a temporary matter, nor a short-term behavior, but a need fora long-term system of regulations and management. To this end, Hengtong Group set up a department to manage social responsibility work which will also draw on international standards to build corporate responsibility management system.
For society
Contribution to society as our mission
For clients
Creating value and common development
For shareholders
Realization of maximized capital
For staff
Care, tolerance and growing together
Innovation is the Power House of HENGTONG

HENGTONG invests 5% of its sales volume annually in researching new products and technology of the future. Advanced production and testing equipment, together with strong, international R＆D capability, are crucial to HENGTONG’s strong position in the wires and cables industry. HENGTONG has partnerships with renowned players in R＆D globally.

Hengtong has advanced testing equipment, strong R＆D,a group of high-tech R＆D talent and a number of core technologies.

The company has developed more than 60 kinds of new products， of which 51 products have been identified by the State Intellectual Property Office and granted the inventing and designing utility model patent. The company established Postdoctoral Research Station and Postdoctoral Technology Innovation Center in 2002, which were identified as 'Jiangsu Engineering Research Center' by Jiangsu Office of Science and Technology and Ministry of Finance in 2004.

Research Platform

Research Achievement

Research Paper

Certifications

Solutions

HENGTONG, making the best of its advantages on the brand, marketing, technologies, equipment, management and the its team, proposes the first comprehensive application solution on wire & cables in the industry, thus realizing the transition from providing single product to offering technical solutions.EPC

The EPC business of communications, electric power and marine is one of HENGTONG’s important business segments. The company has an EPC project management team with leading technologies and more than 1,000 employees. It has long been committed to providing comprehensive, effective and reliable full life cycle services ranging from feasible research and investment/financing planning, survey/design, and construction, to operations and maintenance for global customers in EPC, BOT, BOO, PPP and other models in the fields of electric power and communications. Its professional manufacturing and services include optical cable transmission, FTTx, quantum secure communications, and various types of power distribution, generation, transmission and transformation. The company has developed itself into the famous provider of communications, electric power and marine EPC services and technologies in China. Its business covers 20 countries and regions in the world.

As a responsible citizen enterprise, HENGTONG always puts the protection of the environment and life’s safety and health first, and strictly implements an effective HSSE management system and guidelines with integration of Health, Safety, Security and Environment.

The system has been implemented in all project management and operational decision-making, achieving good results. The company implements strict HSSE protection for employees, customers, partners, and the public as well, and pursues better HSSE management through its continuous improvement.

Products

HENGTONG aims for high-end technology and products, conforms to the industrial trends of communication and electric power, and is dedicated to the needs of optical communication,

power distribution＆transmission and a variety of special transmission applications.
Power System
Telecom System
Submarine System
New Energy
142.Choice International

In 2015, CAD Fund invested Choice International, became its shareholder.

With CAD’s powerful support, Choice International upgraded its business model from traditional trade to international branding and marketing operator.

As the feedback, we contributed to helping the daring building branding products and services in Africa
Investment　Consulting
We connect Africa and the world
Since we managed to build branding and operate branding, we can do more with that.

- Introduce African investment programs

- Connect Nigeria with en at high level

- Introduce international capital

Branding
Branding and products enlighten
Africa
In 2000, Choice International built its own branding. The brands covered multiple domains.

Choices International focus on Africa Market’s need, contribute to helping the local people on better life.

Now, we eager to import more brands and capitals on improve Africa’s sphere. We are always hardworking on that.
Investor
CAD funded us focusing on Africa
In 2015, CAD Fund invested Choice International, became its shareholder.

With CAD’s powerful support, Choice International upgraded its business model from traditional trade to international branding and marketing operator.

As the feedback, we contributed to helping the daring building branding products and services in Africa
Market
Brands-operating
Brands became well-known in Nigeria

In 2013, Choice International introduced the GAC® brands to Nigeria.

As one of top five biggest vehicle brand in en, the GAC ® show its design and performance powered by the channel effect of Choice International.

More international leading brands are on the way of becoming leader brands in Africa boosted by Choice International.
We can find you these opportunities in Africa
Agricultural
Power
Extractive Sectors
Infrastructure
Social Services
Aviation
Services
Automotive Industry
143.Yewhing
Established in 1993, Yewhing (H.K.) Holdings Co., Ltd. has a registered capital of RMB 50 million .and the Vice-Chairman of Sino-African Civilian Chamber of Commerce, the Chairman of Qingdao Sino-African Chamber of Commerce, the Vice-Chairman of Angola-China Chamber of Commerce and the Chairman of Chinese Security Protection Association of Angola were from the company. After 20 years of development, the company has become a comprehensive transnational enterprise group in the fields of commerce and trade supply chain, cross-border e-commerce, mechanical and electrical engineering contract, international trade and investment in Africa. Now it has over 200 Chinese employees and over 500 foreign employees.

The company owns multiple commerce and trade businesses in Angola such as MDC home decoration one-stop shopping center, BAOBABAY cross-border e-commerce, ISP business, digital city, digital product wholesale and 3C-Global retail. In addition, it trades building materials and mechanical and electricla products and is the exclusive agent of Huawei, Gree and Hitachi commercial air conditioners. In the field of mechanical and electrical engineering, it offers a one package service including solution design, installation and repair of generators, generator station and central air-conditioning and it is also the exclusive contractor of Gree and Hitachi commercial air-conditioners. Its international steel trade covers the markets of Middle-East, South America and Africa. It also invested in Africa in the fields of felling and wood processing, manufacturing of fishing boats, fishing and fish processing, agricultural development projects, furniture and building materials and other light industry manufacturing fields.

The company persists in the business concept of “international thinking, localized operation and professional management”, the value guidance of “wining respect of customers and moving customers” and the enterprise spirit of “responsibility, high efficiency, excellence and win-win” to realize the enterprise mission of “becoming an internationalized enterprise respected by the society”. It initiates passionate work and happy life and expects to get mutual benefits with the

partners, the markets and the society.
Yewhing Culture

I. Our mission: Business benefiting all. Do business to connect resources, bring benefits back to people.

II. Our vision: To be a well-respected international group.

III. Our strategies: A global mindset, localized operations and professional management

IV. Our standards at work: Work ethics, professional knowledge, systematic thinking and respect for systems

V. Our core values: Responsible, efficient, transcending, win-win
1、Responsible

Responsibilities to society: The old model of business—profit as the sole purpose—is long past. At Yewhing, we prioritize social values, compliance with the law, integrity and credibility, and contributions to the local community, environment and its people over profits.

Responsibilities to employees: We provide a safe and beautiful workplace for our employees, respect contracts, deliver timely payrolls, share business operating results, and provide opportunities to encourage personal growth.

Responsibility to take on challenges:
First, for every employee at Yewhing, it’s a responsibility to work hard, abide by rules, and do one’s work well.
Second, by taking on challenges outside of your assigned job or expertise and succeeding at them, you take such responsibility one step forward.
Third, we appreciate our employees’ responsibility to initiate providing solutions for the company, see the bigger picture, subordinate individual interests to general interests and willingly take on bigger challenges and more demanding assignments and succeed at them.
2、Efficient

We encourage our staff to finish work on time or ahead of schedule while discouraging time-wasting. It is our hope to achieve the best result within the shortest time by encouraging our staff to be more focused, professional and get ahead.

Yewhing emphasizes the input efficiency where every kind of input is expected to bring higher output. We continuously improve the cycle of investment to make sure higher returns with limited resources.

Our concept of high efficiency also can be found in the enterprising spirit of our employees. We

stress the importance of personal development through learning so that our employees can grow to be more efficient at their work.

3、Transcending

Years of growth has seen Yewhing evolve into a stronger multi-faceted leader in industries while at the same time transcending itself to get involved in new fields of business and achieve greater developments under volatile economic conditions.

At Yewhing, we encourage each and every employee to have a sense of honor to work toward their best possible performance and to rise above themselves instead of resting on their laurels.

At Yewhing, we encourage our staff to take on new challenges, assignments in new fields or areas and to accumulate expertise in such areas so as to achieve new accomplishments.

4、Win-win

Always putting our clients first, we strive to be complementary to our clients by sharing resources and information and bring benefits to both sides.

We emphasize win-win results for employees and partners by encouraging them to perform their respective duties so as to achieve better returns. Through a fair and just distribution, a win-win outcome can be achieved by realizing greater personal interests for employees and partners.

We value the harmony of teams and advocate teamwork spirit. Mutual help and support, collaboration and information sharing can all be conducive to group cohesion and help achieve our goals and realize win-win results.

Yewhing Social Responsibility

In line with our vision to be a well-respected international group, Yewhing always keeps in mind its social responsibility to give back to the people and the society.

On the morning of Dec. 17, 2018 till on Dec. 21, 2018, Pan Jingjian, Secretary General of Security Protection Association, visited Belas Police Station, Viana Police Station, Talatona Police Station, Luanda Municipal Police Station, São Paulo Police Station and Catete Police Station and donated materials of 1,000,000 - 2,000,000 kwanza and a consolation prize of 500,000 kwanza to condole the families of the murdered policemen.

On 17 August, 2018, the delegation led by Huang Ping, deputy director of Yewhing Group, went to the Orphanage of Viana which is the designated organization supported by the group for visiting and donating the sympathy materials.

In December. 2017,Mr.Pan, the vice-president of Yewhing， secretary-general of Security & Defense Association,with other representatives of Security & Defense Association，visited 5 local police stations, and donated some life necessities， which were worth 2.5 million Kwanzaa, and one million Kwanzaa consolation money.

On December 21,2016, together with other Chinese companies in Angola， our company launched donation activities towards the orphanages here, which brought a lot of daily necessities，also our best wishes to them.

On March 2, 2016, a charity event “The Power of Love, Help to Leukemia Kids” was dedicated

to helping kids with leukemia.
On November 30, 2014, Yewhing Angola participated in Angola Christmas charity sale event and donated more than 4,000 dollars worth of items.
Yewhing Honors
In 2011, Qingdao Yewhing Import & Export Co., Ltd was named one of 100 District Leading Enterprises in Qingdao.

In February 2012, Yewhing (H.K.) Holdings Co., Ltd. was awarded ISO9001 certification.

In December 2013, Qingdao Yewhing Import & Export Co., Ltd was awarded "Most Credible Enterprise of the Year—Qingdao".
In January 2014, Yewhing Angolar was awarded "Outstanding Enterprise of the Year 2013" by the Chinese Chamber of Commerce in Angola (CCCA).
On December 20, 2015, Yewhing Angola was honored with "Outstanding Contribution Award" by the Chinese Chamber of Commerce in Angola.
On April 2, 2016, Yewhing Angola was awarded "Exemplary Enterprise" of 10-year development by the Chamber of Commerce of Chinese Companies in Angola.
144.Wanxin Doors & Windows Manufacturing
Doors & Windows Manufacturing
20 Years Longer Experience
GOOD QUALITY
COMPETITIVE PRICE &
PERFECT SERVICE FOR CLIENTS
Dear Clients
Wanxin Doors & Windows Manufacturing (Belongs to China WanXin Trade Co., Ltd.) was established in 1999, located in Zhejiang province, China, and have been with 20 years longer history of manufacturing and exporting.
Our company tenet is"Good quality,Competitive price & Perfect service for clients".
Our products are Steel door ,Wooden door,Door skin,Aluminum & PVC-U window and Kitchen cabinet ect.
Our products are exported to seven greatest continents, East-Europe, Africa, Middle-East, Asia, South America and Oceania ect.
Hope to be your long term partner in China.

Thanks & Best Regards Sunny Lee
Sales Director
Wanxin Doors & Windows Enterprise

Project
Hotel Project
Residential Project
CBD Project

Public Facilities Project

145.ZTT Group

ZTT Group(Full name: Jiangsu Zhongtian Technology Co., Ltd.) has a diversified industrial portfolio that includes marine equipment, renewable energy, new materials, smart grid, optical communications, and other diversified industrial products. With 80 global subsidiary companies and over 16,000 employees, ZTT is one of the largest advanced manufacturing enterprises in China and is listed among the Top 500 Chinese Enterprises.

At ZTT, we believe that sustainability is not only a moral imperative but also a business imperative. We are committed to reducing our environmental impact and promoting sustainable development in everything we do. From our manufacturing processes to our product design and supply chain management, we strive to minimize our carbon footprint and conserve natural resources. We are working towards net zero emissions and are taking concrete steps towards achieving our goals.

ZTT has a global presence, with 54 offices and 12 marketing centers set up overseas and 5 overseas plants operated in India, Brazil, Indonesia, Morocco, and Turkey. The company's products are exported to over 160 countries and regions, and in 2022, ZTT broke through $13.4 billion in sales revenue.

Company Culture

Precision Manufacturing

Jiangsu Excellent Enterprise

Top 100 Electronic Information Enterprises

Top 10 Competitiveness Enterprises in Global Optical Fiber and Cable Field

Top 100 Companies Listed on the Main Board by Market Value

Human resource

Human resource is our primary resource

ZTT adheres to the "university-industry collaboration" mechanism, and has established a long-term strategic partnership with University of Electronic Science and Technology, Nanjing University of Posts and Telecommunications and other colleges and universities for scientific problem tackling and talent training through the technological innovation platforms such as Zhongtian Technology Research Institute and Zhongtian Technology Institute, which has become the source of innovation for sustainable development. The Human Resources Department of the company arranges special personnel to explain the company's rules and regulations, corporate culture and other essential knowledge to new recruits. In addition, ZTT has hired external experts to train employees on careers, business etiquette, and invited industry technical experts to give professional lectures. ZTT has established a comprehensive training system to help each employee achieve their career aspirations during probation.

Innovative Entrepreneurship

Scientific and technological innovation is the symbol of the core competitiveness of enterprises and the foundation for long-term survival of enterprises. ZTT will hold a grand meeting of technicians in every year - Innovative Entrepreneurship Conference. Adhering to innovation and attaching importance to innovation has become the consensus of ZTT. The company has given great support and care to the technical personnel's bit efforts, and as long as there are

achievements, the company will not hesitate to reward. The Innovation and Entrepreneurship Conference has become a symbol and driving force for ZTT, which makes the technicians happy, refreshed and confident, and allows the professionals involved in technological innovation to be respected and given their status, and the innovation atmosphere of ZTT full participation is more intense.

At the same time, ZTT has provided a rich innovation platform for technicians, and built a state-level enterprise technology center, post-doctoral research station, enterprise field workstation, enterprise graduate workstation, ZTT Research Institute, Enterprise Key Laboratory and more than 10 at the provincial level or above innovative platforms such as engineering centers and engineering technology research centers.

Talent Concept

University-industry Collaboration

Let Everybody Fully and Appropriately Display Talents

Talent is Our First Resource

Products

ZTT is a world-class optical fiber manufacturer and supplier, which has independent core intellectual property rights of optical fiber. So far, ZTT fiber is reference in 138 countries globally. ZTT optical fiber can be applicable for transmission system rate up to100Gb/s, and also has supporting technologies for 400Gb/s system.

ZTT has her own research and development center, from fiber preform to optical fiber. ZTT have the unique fiber drawing technology, produce high quality products. ZTT optical fiber, which is made of high purity SiO2, Ge and coating acrylic resin, provides large capacity and fast speed for data transmission.

ZTT fiber meet the international standards of ITU and IEC series (such as ITU-T G.652, G.655, G.657, G.651, IEC 60793 etc.) and can be supplied according to customers’ requirements of standards.

Employee Care

Light the Frame of Life - ZTT Launched a "Voluntary Blood Donation Pass the Love" Activity

Recently, the subsidiaries of ZTT including ZTT RF, ZTT Submarine Cable, ZTT Cable Accessories, ZTT SRIM, ZTT Alloy and other brother units jointly launched the activity of"Voluntary Blood Donation, Pass the Love", which attracted the staff...

ESG

In the history of development, ZTT has always maintained a concern for social welfare and a high sense of social responsibility. ZTT has carried out many activities to perform social responsibility, such as charitable donations, hope primary schools building, and so on.In the future, ZTT will strengthen confidence and provide better products and services for the whole society to make contributions to social stability and economic development.

Charitable Donations

Practicing social responsibility is the mission for enterprises to perform responsibilities in society, morality, economy and environment, which is also necessary for enterprises to create high-end

Don’t forget the water-well digger when drinking water. ZTT Group does not forget to repay for

the society while development. It pays attention to the promotion of regional economy and actively carries out various charity activities.

146.Sansheng

Chongqing Sansheng Industrial Co., Ltd. is a diversified listed company in China. The stock code is 002742. The stock is abbreviated as "Sansheng Stock".

Sansheng shares pay close attention to the development trend of global building materials industry and pharmaceutical health field, pay attention to the introduction of scientific and technological personnel, Market Research and the development of high-tech products, and have formed industrial scale production in mining development, chemical industry, commercial concrete, concrete admixtures, pharmaceutical raw materials, pharmaceutical products and pharmaceuticals.

Sansheng Stock Co., Ltd. takes building the pharmaceutical health industry chain as the strategic direction, taking pharmaceutical intermediates and raw materials as the basis, taking preparations as the core, and extending to the comprehensive pharmaceutical health industry of medical treatment, rehabilitation, health preservation and old-age care.

Enterprise Vision: Connect the world with industry and build a hundred years with health.Creating History through Health Care.

Culture

Sansheng Shares, a platform for its staff to achieve self-realization, a pathway for its shareholders to gain interests and returns, a sincere party for its partners to gain win win development, a responsible and self-disciplined enterprise for the government to supervise without effort, has always practice the mission of"Creating Value and Rewarding the Society with its staff.

ETHIOPIAN ENTITY

SAN SHENG SHARES

Pharmaceutical

Sansheng Pharmaceutical Co., Ltd. adhering to the idea of "international advanced and domestic first class", according to the European Union as a whole. GMP standard design, supporting China's first-class pharmaceutical equipment, high degree of automation. There are three building buildings in the whole plant, which are complex buildings, production workshops and staff dormitories.Building. The whole production area is equipped with purified water, injection water, steam, air treatment, sewage treatment and so on.High standard public facilities.

Building Material

Sansheng Building Materials P.L.C. is located in Eastern Industrial Zone in Ethiopia. The company owns one set of 180-type automated concrete production line, 12 concrete mixing transport trucks, one 38-meter and one 48-meter arm-type concrete pump and two truck-mounted concrete pumps, and the designed annual production of cocail concrete is 300,000m^3 .

147. Sinotrans

Sinotrans Limited(referred to as "Sinotrans") was incorporated on 20 November 2002 and listed in Hong Kong Stock Exchange in 2003. As the second-tier subsidiary and single logistics platform for China Merchants Group (CMG), Sinotrans Limited aims to build a world-class business platform with intelligent logistics services. According to the latest Armstrong & Associates, Inc' s list, the third-party logistics and freight forwarding services in the world rank sixth and fifth respectively.

Sinotrans has formed its three main business segments including agency and related business, supply chain logistics and e-business, which can provide end-to-end supply chain solutions and one-stop services.

Vision：

Becoming a World-Class Intelligent Logistics Platform Enterprise

Mission：

Transport everything, connect the world, and create ecology

Promoting the progress of the industry with logistics success

Global Network

In China, our strong service network covering all of China's provinces and territories, overseas network covering Asia, Africa, the Americas, Europe and other regions and districts. Whenever, wherever, China Sinotrans will provide customers around the globe with high quality logistics services.

Sustainability

Sinotrans is actively involved in social events, environment protection, endeavoring for a better life of the people and a more harmonious community.

For years Sinotrans has taken environment protection as its own responsibility. By optimizing transport routes to improve fuel efficiency and taking advantage of new technologies to enhance operation efficiency, Sinotrans has built up a green logistics system.

Sinotrans is dedicated to help people and communities in China and other parts of the world affected by poverty and major natural disasters. Apart from donations, Sinotrans delivers relief supplies and makes every effort for post-disaster reconstruction and rehabilitation.

Sinotrans continuously makes contribution to people’s health and cultural life through servicing and supporting large sporting and cultural events, such as the 2008 Olympic Games, and the 11th Asian Games, etc.

With the joint efforts of all staff of Sinotrans, our quality logistics services facilitate the world trade and sustainable economic development.

Environment,social and governance reporting

Social welfare-Poverty Alleviation

In 2020, China secured full victory in building a moderately prosperous society in all respects, and attained a complete victory in the fight against poverty. In the face of the test of COVID-19, Sinotrans, according to the overall arrangement of the China Merchants, continuously focused on the overall objective of “making rural poor people free from worries over food and clothing and have access to compulsory education, basic medical services and safe housing” and spared no effort to work for targeted poverty alleviation by combining the task of poverty alleviation in poverty-stricken counties and taking China Merchants Charitable Foundation as a professional unified public welfare platform. Under the guidance of the corporate strategy, Sinotrans

effectively combined social responsibility with corporate development strategy, and integrated the concept of social responsibility management and commitment to stakeholders into corporate management, in a view to promoting the infusion of social responsibility into all aspects of corporate production and operation, and striving to build a respectable international company. The board of directors carried out a special review on the annual social responsibility report,and deliberated and made decisions on major issues related to social responsibility. The management actively implemented the concept of social responsibility. A social responsibility special group was established to publicize and advocate social responsibility, take actions to fulfill social responsibility, and regularly sort out and collect information on social responsibility fulfillment. Subsidiaries are also required to actively perform social responsibility and carry out various kinds of social responsibility activities in their respective regions.

In 2020, Sinotrans donated 12 million yuan as poverty alleviation funds through the China Merchants Charitable Foundation. The money was utilized under the overall plan of the Foundation. By the end of 2020, the actual expenditure of the China Merchants Charitable Foundation for targeted poverty alleviation work had reached 62.441 million yuan, and 23 poverty alleviation projects had been launched. 51.661 million yuan was allocated to national level poverty-stricken counties including Weining County of Guizhou Province, Qichun County of Hubei Province, and Yecheng County and Shache County of Xinjiang Province. 5.762 million yuan was spent in Zhenxiong County, Yongren County and Wuding County of Chuxiong Yi Autonomous Prefecture in Yunnan Province, and 5.018 million yuan was utilized in other poverty-stricken areas.

Under the overall guidance of the China Merchants Charitable Foundation, Sinotrans did a series of public welfare work for poverty alleviation, and bravely assumed corporate social responsibility.

I. “Whenever the country needs, China Merchants will respond and Sinotrans will be there”2020 was an extraordinary year. In the face of the COVID-19 pandemic, Sinotrans and the Chinese people went to the war and fought together against the virus. With the strategic performance of the “five channels and one platform”, we built a global lifeline for fighting against the pandemic and a transportation line for resuming work and production, which transported a total of 20,600 tons of supplies for the fight against the virus. We completed major tasks of logistics support for China’s foreign aid, and effectively fulfilled the role of “Whenever the country needs, China Merchants will respond and Sinotrans will be there” as a central SOE. Our “Disaster and Emergency Logistics Volunteer Service Team” was honored as the “National Advanced Group for Combating COVID-19”.

II. Sinotrans made solid progress in the poverty alleviation work in Weining
Sinotrans Party Committee made overall arrangements to continuously actively participate in the poverty alleviation work in Weining, Guizhou according to the poverty alleviation plan of "opening the cold chain logistics channel to help send out agricultural products in Weining" proposed by the Group. In 2020, a total of 266 tons or 1000 cubic meters of agricultural products were transported to Beijing, Hubei, Guangdong, Liaoning, Shandong and other places by full truckload cold chain, establishing a "green cold chain transportation channel" for transporting local agricultural products in Weining to developed areas. The company also launched the China Merchants characteristic poverty alleviation product "Plateau Vegetable Bags", and developed an overall scheme that integrates procurement, sorting, packaging, sales and transportation. About 20,000 vegetable bags were booked throughout the year, including 8799 bags or 1265 cubic meters transported by full truckload.

III. Sinotrans continuously carried out characteristic public welfare and volunteer projects for poverty alleviation.

As the COVID-19 swept across the globe in 2020, many public welfare and volunteer projects for poverty alleviation were canceled, while the staff of all levels at Sinotrans pulled together, kept doing volunteer work and continuously taking social responsibility as a central SOE without any slack in epidemic prevention. In the process, a number of great volunteer service events emerged, benefiting as many as 50,000 people.
For example, the videos selling goods made by Sinotrans in response to the 27° Farmers' youth host poverty alleviation plan were played on major video streaming platforms, which attracted 32,000 online views, helping the Group create the public welfare brand "27° Farmers" and opening a new path of poverty alleviation through consumption. It was selected among the 50 excellent examples of poverty alleviation through consumption.
In 2021, Sinotrans will continue to press ahead with work concerning public welfare poverty alleviation and social responsibility in accordance with the requirement of "keeping up aids and policy support even the aided have shaken off poverty".
Awards & Recognition
2020 Corporate Excellence of China Logistics
2020 National Advanced Logistics Anti-Epidemic Enterprises
2020 Science and Technology Award of China Federation of Logistics & Purchasing
2020 Top Graduate Employers
2019 Corporate Excellence of China Logistics
2019 The Asian Freight ,Logistics & Supply Chain Awards

148.Development reimagined
We are a pioneering, African-led, women-led and award-winning international development

consultancy

Our diverse and multilingual global teams combine deep, multisectoral expertise and cutting-edge analytics to help businesses, governments, and international organizations reimagine their approach to trade, aid, finance, climate action, and much mores.

Our advisors bring trusted expertise and new perspectives to:FINANCIAL TIMES，THE DIPLOMAT，African BUSINESS，the africa report.

Our flagship projects

Elevating Africa in global markets and dialogue.

Africa’s premier China market entry service platform and full-service consultancy

African perspectives on the continent’s key economic challenges and opportunities

What we can do for you

SOUND STRATEGIC ADVICE

DATA-DRIVEN ANALYSIS

Strong Partnerships & Events.

Enabling Dynamic Investments.

OUR WORK AREAS

AFRICA-CHINA

DECOLONISING DEVELOPMENT

CLIMATE ACTION

DEVELOPMENT FINANCE

GLOBAL TRADE

AFRICA-CHINA

Making from “China-Africa” to “Africa-China” a reality

At Development Reimagined, we firmly believe Africa’s own development plans and priorities should be highly valued by all development partners, including China. We know from experience that cooperation is most effective when governments and the private sector align their plans with African priorities and needs.

Development Reimagined has long-standing relationships among the African Diplomatic Corps in Beijing, the China Africa Business Council (CABC), and Chinese ministries including the Ministry of Foreign Affairs and the Ministry of Commerce. We draw on this deep network to provide unparalleled insights on China’s role as a key development partner for the African region in striving to achieve the UN Sustainable Development Goals (SDGs) and Agenda 2063: Africa’s strategic framework to deliver on the African Union’s goals for inclusive and sustainable development by implementing 6 continental frameworks and 15 flagship projects.

Our work – including our flagship Africa-wide “China Strategy” – draws on our unique network and DR’s trusted role in China, along with an in-house portfolio of unique databases. These rich resources enable us to publish a series of original, timely research as well as convene innovative capacity building and trade/finance facilitation activities to help African countries get the most out of existing and future relationships with China.

DECOLONISING DEVELOPMENT

Reimagining the aid and development sectors to become truly “sustainable”

At Development Reimagined, we take seriously the agency of low-income and middle-income countries to reshape aid and development – including the global health, agriculture and humanitarian sectors – into more equitable, sustainable and exit-able systems.

As a pioneering, African-led development consultancy, our team provides a unique perspective backed by evidence-based analyses of the challenges and opportunities for change in aid and development, along with tailored support to organisations and partners that wish to drive internal or external change and/or design new programmes that truly center recipient needs.

We do not shy away from difficult topics or conversations, but also focus on the practical activities, norms and outputs that might be needed. In all our work, we conduct and draw evidence and inspiration from a diverse set of organisations, other non-development sectors as well as a diverse range of countries, including emerging economies such as China.

Our work on Making Africa’s Pharmaceutical Needs a Reality: Part 1 and Part 2 is a key example, where we investigated the local medicines manufacturing deficit in Africa, and the learning opportunities from China and India’s pharmaceutical sectors.

149.Dongfeng Motor Corporation Limited
Dongfeng Motor Corporation Limited is a significant backbone of China's automotive industry. Its predecessor, the Second Automobile Manufacturing Plant, was established in 1969. The company is headquartered in Wuhan, Hubei Province. It currently has total assets of 499.3 billion yuan and employs 127,000 people, ranked 188nd in the world's top 500. It has sold over 1,200,000 vehicles overseas, with its products sold in over 100 countries.

Our main business covers commercial vehicles, passenger vehicles, NEVs, auto assemblies, parts and components, machines and equipment, and other automobile-related products and services.

After over 50 years of development, Dongfeng Motor has become an industrial R&D, manufacturing, and marketing leader. At the same time, Dongfeng Brand has become a well-known name for a long time and also enjoys a good awareness in the global market.

Its commercial vehicles (trucks and buses) cover a full range of series, including medium-duty, heavy-duty, light-duty, mini trucks, and conversion trucks for particular purposes. The passenger vehicles include cars, SUVs, MPVs, mini-CVs, and Special trucks. High-mobility special off-road vehicles include the Dongfeng Mengshi, desert off-road, and AWD off-road vehicles. Moreover, Dongfeng Motor also offers new-energy vehicles, including HEV, all-electric, and natural gas vehicles. Dongfeng Motor can provide customers with the most reliable and valuable product solutions with its full range of products.
BRAND STRATEGY
We Aim to Become a Prominent Tech Company Providing Superior Products and Services for Customers
Company Mission
Drive your dreams

Company Values
Quality | Intelligence | Harmony
Three Business Segments
Vehicle business | Scientific and technological development | Service ecology
Science and Technology Advancement Plan
Dongfeng Advance
CORPORATE CULTURE
As an excellent brand with more than 50 years of development history, Dongfeng is the China automobile industry's first well-known trademark and one of the World's Top 500 Famous Brands, winning such honorary titles as China Famous Brand Product and The Most Competitive Brand in the Market and enjoying high brand awareness and reputation both at home and abroad. Dongfeng Motor is one of the Chinese auto brands that entered the world's top 500.
The Dongfeng brand text logo is . Its graphic logo resembles a pair of revolving spring swallows in a circle, vividly representing the vision "Two Swallows Dancing with Dongfeng" and evoking the sense of the East Wind (Dongfeng) bringing warmth and enchanting sunny days; every living thing is full of vitality, intimating the company's prosperous future. The logo also resembles a double "人" (the Chinese character "ren," meaning "people"), implying that the company infuses its business with a people-oriented philosophy. The dancing, flying sparrows in the logo revolve like a wheel moving forward, representing Dongfeng Motor's products galloping throughout China and toward the global market.
DONGFENG GLOBAL STRATEGY:
BRAND PYRAMID
01 Mission Drive your dreams
02 POSITIONING
An excellent technology enterprise providing global users with quality automotive products and services
03 SLOGAN
DRIVE YOUR DREAMS
04 KEY VALUES
QUALITY INTELLIGENCE HARMONY
05 BRAND FOUNDATIONS
Advanced Quality
Intelligent car manufacture
World's top suppliers
Guaranteed safety
The choice for global customer
Tech Innovation
World-leading NEV tech
Mach power and Loong-Gine power
Patented technologies
Global R&D and design team
Global Operations
Fortune 500
World's top automakers as partners

Global intelligent KD factories
Global dealership + service network
GLOBAL COMMS: BRAND VALUES
Quality is the root of the Dongfeng brand of the new era, uniting the company, its employees and its products.
Intelligence is the source of Dongfeng brand in the new era, and we are committed to constantly improving our ability of providing intelligent products and customers' experience of using them.
Harmony is the soul of the Dongfeng brand in the new era, and we foster and uphold a positive and pleasant ecology for our customers, our employees and our society.
GLOBAL COMMS: BRAND FOUNDATION
Intelligent Car Manufacture
World's Top Suppliers
Guaranteed Safety
The Choice for Global Customers
World-leading NEV Tech
Mach Power and Loong-Gine Power
Patented Technologies
Global R&D and Design Team
Fortune 500
World's Top Automakers as Partners
Global Intelligent KD Factories
Global Dealership + Service Network
TECHNOLOGY & INNOVATION
Dongfeng Motor is a "National Innovative Pilot Enterprises" in China. The Company boasts a leading car research and development system with over 7000 R&D staff (accounting for 5% of its total number of employees) and the most significant automobile test base and self-assembly capability in China.Moreover, the Company has ranked first in several awards for scientific and technological progress in China's automotive industry for six consecutive years and boasts one of the most significant number of patents in the industry. As a state-level "enterprise technology center" in China, the Dongfeng Motor Technology Center will enable Dongfeng Motor to lead development by technical innovation with the support of its global research and development centers.
GLOBAL EXPANSION,CONSTANT INNOVATION
Dongfeng Motor has led a development path of continuous breakthroughs by adhering to independent R&D and innovation based on the formulation and implementation of its globalization strategy. Dongfeng Motor’s overseas business covers more than 30 countries and regions in Asia, Africa, South America, and Central and Eastern Europe. While expanding its overseas market, Dongfeng Motor has been shaping an internationally famous brand with its advanced management concepts, technology, and top design to support its brand globalization. Located in China, Sweden, and Germany, our domestic and overseas R&D bases are committed to technological innovation and application in the automotive industry through joint research and development.
ADVANCED TECHNOLOGY
Thanks to its global strategy and pursuit of independent R&D and innovation, Dongfeng has

embarked on a path of continuous breakthroughs. Dongfeng's R&D centers in China, Sweden, and Germany are working together in their commitment to technological innovation and application in the automotive industry.
TECHNOLOGY LEAP ACTION
The automotive industry is experiencing an unprecedented transformation, and Dongfeng's intelligent technology is becoming a key driving force for the industry to enter a new era.

The "Technological Leap" represents the flagship action in technological innovation, symbolizing the revolutionary development of clean energy and intelligent technology. It is the technical force that Dongfeng brings to the automotive industry.

"New Energy Leap" and "Intelligent Driving Leap" symbolize that two innovative technology fields will usher in leap-forward development. It also carried out technological upgrades in traditional power to help save energy and reduce emissions, and launched two major power brands, Mach power and Loong-Gine power.
HONOR & AWARDS
iF Design Award 2022 in the UI Discipline
VOYAH's HMI design, redefining the relationship between vehicle and screen, won the iF Design Award 2022in the Ul discipline with a score of 305 (the Award's bench-mark score was 270), highlighting VOYAH's modern luxurywarm technology and simple yet superior design concept.
China Automobile Festival 2022
VOYAH FREE won the jury award at the China Automobile Festival 2022, an automobile awards ceremony hosted by China's CCTV.
China EV Rally
At the 8th China EV Rally (CEVR) (Qinghai Lake), VOYAH FREE, a newcomer, won multiple awards as a premium NEV model.
Transform Awards Asia 2020
VOYAH'S brand logo won a gold award for best visual identity at Transform Awards Asia 2020.
C-NCAP Five-Star Rating
VOYAH FREE has won a C-NCAP (China New Car Assessment Programme) five-star safety rating.
China Car Body Conference 2020
VOYAH FREE (WHITE) won a medal at china car body conference 2020
2017 Fortune Global 500
Dongfeng Motor ranking 68th
Top 100 Chinese Brands 2017
2017 Chinese Brands Summit Forum. Dongfeng Motor was listed in the Top 100 Chinese Brands of 2017
China Enterprise Confederation and China Enterprise Directors Association
2017 Top 500 Manufacturers of China Dongfeng Motor ranks 3rd.
Interbrand Best China Brands 2017
Dongfeng Motor ranks 37th in all brands and 1st in the automobile industry.
Xinhuanet, Academic Division of Economics, Chinese Academy of Social Sciences and China Enterprise Reform and Development Society
“ 2017 China Social Responsibility Awards, Outstanding Chinese Company of 2017 for Precise

Poverty Alleviation.
State Administration for Industry and Commerce and World Intellectual Property Organization
China Trademark Awards Ceremony. Dongfeng Motor awarded "2017 China Trademark Award-Trademark Application"
CCID Research Institute, Ministry of Industry and Information Technology
2017 (3rd) China Summit of New-energy Vehicle Leaders. Dongfeng Motor E70, titled The Most Popular Model.
Society of Automotive Engineers of China (SAE-China)
2017 China Automotive Industry Awards for Science and Technology. Twelve projects of Dongfeng Motor granted awards; Dongfeng Motor titled Member of the Year for SAE-China from 2013 to 2017.
PSA Group
2017 "Golden Palm" Global Industrial Awards Ceremony. 3rd Plant of Dongfeng Peugeot-Citroën Automobile Co., Ltd. granted the "Golden Palm-Best Plant" award.
Special Events Gala Awards
Dongfeng Motor's "Vanucia-LeTV • Charity Tour China" cycling event is titled "The Best Event Marketing Campaign."
World Economic and Environmental Conference
2016 Annual Summit on Green Low-carbon Transformational Development and the 6th International Carbon Awards Ceremony. Dongfeng Motor Chenglong H7 and New Chenglong M3 titled "Recommended Models by the World Economic and Environmental Conference."
4th Annual Conference for Sustainable Competitiveness of Chinese Enterprises
Dongfeng Motor was granted the Award for Best Social Communication Innovation.
China Automotive News
2016 (9th) Consumer Models Ceremony and Forum on Competitiveness of Automobile Brands. The marketing campaign for the launch of Glory 580 of Dongfeng Xiaokang granted the award of "Annual Brand Marketing."
China Youth Daily
Youth Attitude • Annual Car Ceremony and the Automobile Forum of the First International Innovation & Entrepreneurship Expo. Dongfeng Motor titled "Automobile Group of 2016"
9th China Annual International Energy Conservation Competition or Trucks
Dongfeng Motor of driving trucks (500HP+). Dongfeng Motor Captain N300 won the championship for energy conservation of light-duty vans (2.8-3.0L). Dongfeng Motor Duolika D9 won a tournament for energy conservation of light-duty vans (3.0L+). Dongfeng Motor Duolika D6 titled "Most Satisfactory Model for Express Delivery."
2016 China Social Responsibility Awards
Dongfeng Motor granted "Outstanding Chinese Company of 2016 for Social Responsibility" and "Outstanding Chinese Company of 2016 for Precise Poverty Alleviation."
2016 China Car
Dongfeng Motor A9 titled Model of the Year. Dongfeng Motor AX5 is titled Fashionable Independent Brand SUV of the Year, Most-expected Independent Brand SUV, and Most-focused Compact SUV, among other titles.
Dongfeng Motor was granted the "China Charity Award
Dongfeng Nissan granted the "Global Nissan President's Award". Awarding ceremony of the 9th

“China Charity Award”. Dongfeng Motor was granted the “China Charity Award.”
China Automotive Technology & Research Center Co. Ltd
Dongfeng Motor AX7 was granted the Most Promising Chinese Model Award.
Fortune Global 500
Dongfeng Motor ranked 81st
2014 Most Satisfying Commercial Vehicle Brand amongst Chinese Users.
2014 Annual Grand Ceremony for Commercial Vehicles: Dongfeng Motor honored with ''2014 Most Satisfying Commercial Vehicle Brand amongst Chinese Users.''
2015 China Automotive TV Chart
Dongfeng Motor AX7 China City SUV of the Year - Dongfeng Motor AX7.
2015 Fortune
Dongfeng Motor ranked 109th amongst the World's Top 500 of 2014 by Fortune.
2014 China Automotive News Press
Captain N300 and Captain XLCaptain N300 and Captain XL respectively awarded 'Energy-saving Vehicle of the Year' and 'Heavy-duty Truck of the Year' at the 2014 China Truck Model Appraisal.'
2014 China Machinery Industry Federation and China Association of Automobile Manufacturers.
China's Top 30 Automakers of 2013
2014 China Automotive Media Award
China's Most Anticipated SUV-Dongfeng Motor AX7
2014 China Federation of Industrial Economics/United Nations Industrial Development Organization
Dongfeng Motor: China Five-star Industrial Enterprise in Fulfillment of Social Responsibilities.
2014 Fortune
Dongfeng Group ranked 113th amongst the World Top 500 of 2013 by Fortune
2014 Interbrand
2014 Best Chinese Brand: Dongfeng Motor with a brand value of RMB 4.473 billion.
China International Energy Conservation Competition.
Dongfeng Motor Ruiling was awarded Fuel Economy Champion at the China International Energy Conservation Competition.
2014 China Association of Automobile Manufacturers
Dongfeng Motor A30Best Intelligent Car Model in China Automotive Brand Tour-Dongfeng Motor A30.
2013 Chinese Association of Productivity Science (CAPS) and the Committee for Promotion of Innovation of CAPS
DFM was awarded ''2012 China Best Enterprise in Independent Innovation.''
2013 China Automotive News Press
DFM honored ''2012 Most Satisfying Commercial Vehicle Brand amongst Chinese Users.''
2013 China Machinery Industry Federation and China Association of Automobile Manufacturers
DFG awarded ''China Top 30 Automakers in 2012''
2013 Auto Magazine
DFM A60Most Popular Compact Vehicle - DFM A60.
2013 Fortune
DFG ranked 146th amongst the World's Top 500 of 2012 by Fortune.
Awarded Car Model of China Automotive Industry in 60 Years

DFM EQ240 2.5t Truck Awarded 'Car Model of China Automotive Industry in 60 Years.'
Outstanding Listed Company in Environmental Protection
Dongfeng Motor Group Co., Ltd. was named ''Outstanding Listed Company in Environmental Protection.''
2013 Organizing Committee for China Excellent Automotive Industry Design Award and China Industry News Press
DFM KINLAND has given ''The First China Excellent Automotive Industry Design Award.''
2012 Reliability Reports magazine
DFM H30 CROSSCompact Vehicle with Standout Quality in 2011 - DFM H30 CROSS
2012 China Machinery Industry Federation and China Association of Automobile Manufacturers
DFM: The World Top 500, China Top 30 Automakers.
2012 Fortune
DFG ranked 142nd amongst the World Top 500 of 2012 by Fortune.
2011 China’s Most Influential Brands Summit
SOCIAL RESPONSIBILITIES
During the "14th Five-year Plan", to adapt to the national economic and social development requirement and corporate development strategy, Dongfeng Motor formulated a new round of social responsibility action plan-"Nurturing" Plan 3.0, clarifying the strategic vision, target path, and practice system of Dongfeng Motor's new game of social responsibility work, and guiding Dongfeng Motor's social responsibility work to a new journey.
Strategic Vision
Committed to becoming a sustainable development of excellent technology enterprise.
Responsibility Value
Integrity, innovation, empowerment, and goodness.
Work Objective
Strive to build a high-quality CSR working system under sustainable development, construct the social responsibility "3+1" supporting framework, create Dongfeng Motor responsibility soft power, and promote the company to achieve high-quality development.
Science and Technology Advancement Plan
Conduct social responsibility "Nurturing" practice system of social responsibility, focus on the three actions of "Runfeng Action", "Runxing Action" and "Runmei Action", and promote 12 social responsibility practice issues.
SUPPORT ARCHITECTURE
Dongfeng Motor conducts social responsibility "3+1" support architecture and strives to create and improve "three systems" and "one platform" and consolidates a work foundation for social responsibility.
CORPORATE SOCIAL RESPONSIBILITY IDEA
Dongfeng Motor actively conducts the "Nurturing" practice system of social responsibility, focuses on the three actions of "Runfeng Action", "Runxing Action," and "Runmei Action," and promotes 12 social responsibility practice issues and facilitate the quality and efficiency of the responsibility performance.
Add new vitality to the national economy and people's livelihood and share common prosperity.
Implement the major decisions and plans of the CPC Central Committee and the State Council.
Make state-owned capital and state-owned enterprises stronger,better and bigger.

Strengthen scientific and technological innovation capabilities.
Adhere to compliance and legal operation.
Grow and develop with the industrial economy
Accelerate the research, development, and application of emerging products.
Provide high-quality services for customers.
Improve the quality of products and services in the international market.
Strengthen the responsibility management of the industrial chains and value chains.

Develop a beautiful and harmonious social environment.
Continue to fight against comprehensive poverty alleviation and rural revitalization.
Achieve clean, low-carbon and green development goals.
Guarantee the legitimate rights and interests of employees and their physical and mental health.
Promote the development of social welfare undertakings.
IMPLEMENTATION PATH
The social responsibility work of Dongfeng Motor during the "14th Five-year Plan" will adopt the "three-step" path to improve the mechanism, optimize the layout, deepen the practice, polish the results, and help the high-quality development of social responsibility work.
Improve the mechanism and optimize the layout (2021-2022). We will deepen the configuration of the "3+1" support framework and improve the support capacity of social responsibility management. Secondary units shall promote the implementation of the "Nurturing" Plan 3.0 and consolidate the foundation of social responsibility management and practice.
We fully implement the practice system of "Nurturing" Plan 3.0, coordinate and promote social responsibility, realize resource integration and sharing through a win-win social responsibility platform, and promote the value creation of the company's social responsibility work.
We form a closed-loop management of social responsibility work and constantly promote the sustainable development of social responsibility work, set up a model of social responsibility practice, continuously promote the construction of the Dongfeng Motor responsibility community, and build a model of central enterprises.
RESPONSIBILITY SYSTEM
To guarantee the smooth performance of the social responsibility work, Dongfeng Motor establishes and constantly improves the social responsibility system, modifies Social Responsibility Management Measures, Fund Management Measures of Dongfeng Motor Public Welfare Foundation, formulates Guiding Opinions of Dongfeng Motor Social Responsibility System Construction and other rules and enhances Dongfeng Motor's social responsibility system construction and management. In 2022, Dongfeng Motor has optimized and improved four social responsibility work-related systems and processes and guaranteed project compliance.
150.Choice Int’l Forwarding Co., Ltd
Choice Int’l Forwarding Co., Ltd was established in 1997, focusing on Middle East and African countries and providing complete one-stop logistics service for customers in countries including Middle East and Africa. Through many years of operation, it has developed into a professional international comprehensive logistics enterprise with transnational operations at present, engaging in Middle East and Africa import and export cargo transportation and extended logistics services. The company is headquartered in Guangzhou City, Guangdong Province, with branches in Shenzhen Yiwu and Hong Kong. In overseas market, it also set up wholly-owned branch

companies in Dubai, Saudi Arabia, Kenya, Cameroon, Nigeria, D.R.Congo, Uganda, R.E.P Congo, Tanzania, Côte d'Ivoire and Senegal.

The company utilized advanced IT system to conduct effective quality management system, financial control system and personnel training system and gradually formed multimodal transport service system centering on countries including Middle East and Africa and core business plates based on port warehousing, container leasing, ship leasing, shipping company warehouse chartering, air warehouse chartering, contracting container plate, chartered airplane, express line, and Middle Eastand Africa project logistics. As a professional logistics company in Middle East and Africa, we are committed to constantly perfecting the building and improvement of our various service platforms, setting up complete extended service system for different customers' personalized demands in Middle East and African countries, and providing better, high-efficiency and safe logistics service for customers.

The company always adheres to the corporate culture of "Responsibility, Integrity, Innovation and Harmony", with service network covering China main port cities and overseas agencies in over 50 African countries and regions to provide perfect one-stop logistics services for African customers.

CHOICE's Strength

Team

We build up a team with average industry

Experience of 5 years, consisted with African And Chinese staffs.

Service

We've serviced over 1 million customers.

We provide logistics services include pick up service,Customs clearance, payment agent and delivery.

Resource

Cooperate with over 50 shipping countries, 13 global branches support the service, to cargo

Provide you premium services.

Respond

Provide 7*12hours instant respond by online customer service and 24 hours real-time cargo

Tracking.

TECNO Cooperation Case

Customer Introduction:

Established in 2006, TRANSSION Holdings is a diversified, global group, specializing in mobile communication and related industries. The company is best-known for its leading technology brands, including TECNO, itel and Infinix. After 10 years of expansion, TRANSSION is now a leading player in the mobile industry, with a current ranking as the No.7 global exporter and a market share of over 40% in Africa's six major countries. Its cumulative sales volume has reached a landmark of nearly 280 million Dual-SIM handsets.

Pain Point： Batteries of mobile phone are difficult to deliver, and they need to control the logistics cost.

CHOICE’ s Solution: Make custom logistics plan for TECNO to solve the problems of delivery and tariff.

Result: CHOICE provides high efficient and safe logistics service to TECNO. Currently, TECNO is the best mobile seller in Africa and top 50 in China.
Company culture
Our mission
To provide customers with safe, fast, professional logistics solutions;
To provide employees with a platform to learn growth and seek sustainable development.
Our vision
To become the strongest logistics network company in Africa and the Middle East.
Core Values
Integrity
Integrity-based win the trust of the customer
Respect
Mutual respect and achieve win-win situation
Responsibility
Full-time commitment to focus on
Innovation
Based on traditional innovation pilot
Perseverance
Perseverance and resolute down to earth
Struggle
The ultimate fighting spirit

151.Wepon Medical Technology Co., Ltd.

Wepon Medical Technology Co., Ltd. (hereinafter referred to as Wepon) is a novel internationalizing enterprise specializing in high-end medical instrument development, medical equipment manufacturing and hospital engineering services, subordinate to Wepon Group.
In the development thought of “Gather technologies by capitals, gather talents by platforms”, Wepon actively takes part in global industry integration, laying a solid foundation for the development of the group in the local medical instrument industry. Our company has established Anglorand Medical Pty Ltd. in South Africa, which acquired Elite Surgical Supplies Pty Ltd. and Tecmed Africa Pty Ltd., and completed the merger and reorganization with two oversea enterprises, to provide our subsequent development with international advanced technologies and the support and guarantee of subsidiary resources through intellectual property rights and technical platforms of two enterprises, realize industry optimization and transformation of our company and technical upgrading of our products and bring technical breakthrough to the medical instrument manufacturing industry and hospital engineering service industry of China, reaching the international leading level.
In the objective of creating the largest orthopedic instrument production base in Asia, Wepon

strives to realize seamless joint of international advanced technologies and domestic medical instrument industry.
Abroad, technology joint and transfer are propelled deeply at the completion of our acquisition of Elite Surgical and Tecmed Africa;
At home, our company also actively carries out merger and acquisition negotiation with other medical instrument manufacturers;
Recently, there will be four medical instrument production and operation enterprises held and purchased by our company, which marks that Wepon has taken the first change in the layout of medical instrument industry and made adequate preparations for entering international and domestic R&D market of high-end medical instruments, thus comprehensively promoting massive health industry strategies of Wepon Group to develop.

Elite Surgical Supplies (Pty) Limited is based in Pretoria, South Africa, Elite Surgical Supplies provides world-leading medical supplies in the spinal and orthopedic related field. From research, to development, to manufacture, and to distribution, in order to maintain its quality of production, Elite Surgical is able to complete all of these procedures in-house. Elite's products have received wide recognition globally and is used by surgeons worldwide. With seasoned veterans in the field, Elite is able to provide cutting-edge medical technology, and also cater to specific requirements given by clients. It is also relentless in keeping its edge in terms of industry advantage by continuously venturing and succeeding in newer related medical fields such as sports medicine and toolkit solutions.

Tecmed Africa (Pty) Limited Founded in 1992, Tecmed Africa is one of the leading medical equipment suppliers in Africa. Based in South Africa, its operations reaches Kenya, Botswana, Zimbabwe, Mozambique and many other African countries. Apart from its impressive portfolio of products, Tecmed also develop and manufacture its own range of accessories and products. With its state-of-the-art medical technology tailored specifically towards African environment and clients, Tecmed is often called upon to equip entire hospitals from scratch and is one of the only two companies in all of Africa that offers full turn-key hospital solutions.

Wepon

Culture

Exploitation & Innovation, Care for Life

Vision

High-end medical devices

Medical device manufacturing

Hospital engineering services

Innovative and international enterprise group

Core values

Scientific and technological innovation, immediate action

Fine operation, value orientation

Enterprise spirit
Pragmatic, Quality, Innovative, Efficient

Employee life value honor and disgrace
1. Be proud of loving the company and be ashamed of damaging the company;
2. Be proud of pioneering and innovative, and be ashamed of being unwilling to make progress;
3. Be proud of loving one's post and work, and be ashamed of shirking responsibility;
4. Be proud of being active and practical, and be ashamed of being passive and slack;
5. Be proud of unity and mutual assistance, and be ashamed of harming others and benefiting yourself;
6. Be proud of being honest and trustworthy, and be ashamed of losing sight of profit;
7. Be proud of obeying the rules and regulations, and be ashamed of breaking the rules;
8. Be proud of cherishing resources and be ashamed of squandering.
Product & Solution
Department of orthopedics implant instrument series

Implant Department of orthopedics is a general term for a large class of human bone implant replacement, repair, and filling, for human bone maintenance, support and repair, is currently in clinical use of common Department of orthopedics medical materials. The use of implant materials, such as medical implants, enables the recovery of tens of thousands of patients and improves the quality of life of patients with bone fracture.
Wepon
Department of orthopedics implant instrument series

Implant Department of orthopedics is a general term for a large class of human bone implant replacement, repair, and filling, for human bone maintenance, support and repair, is currently in clinical use of common Department of orthopedics medical materials. The use of implant materials, such as medical implants, enables the recovery of tens of thousands of patients and improves the quality of life of patients with bone fracture.

- Orthopedic tools
- Spine
- Joints
- Trauma
- Neurosurgery

Medical polymer series

Medical polymer products can be broadly divided into two categories: machine use and the use of the body. Outside the body is mainly used for the preparation of medical materials, such as infusion bags, infusion lines, syringes, etc..
Surgical instruments series

Surgical instruments are all kinds of medical devices that need to be used in operation. In

principle, it can be divided into two categories: basic surgical instruments and surgical instruments.

Wepon

Department of orthopedics implant instrument series

Implant Department of orthopedics is a general term for a large class of human bone implant replacement, repair, and filling, for human bone maintenance, support and repair, is currently in clinical use of common Department of orthopedics medical materials. The use of implant materials, such as medical implants, enables the recovery of tens of thousands of patients and improves the quality of life of patients with bone fracture.

- Orthopedic tools
- Spine
- Joints
- Trauma
- Neurosurgery

Medical polymer series

Medical polymer products can be broadly divided into two categories: machine use and the use of the body. Outside the body is mainly used for the preparation of medical materials, such as infusion bags, infusion lines, syringes, etc..

- Syringe
- Infusion set
- Needle
- Respiratory care
- Bags
- Other

Wepon

Wepon

Surgical instruments series

Surgical instruments are all kinds of medical devices that need to be used in operation. In principle, it can be divided into two categories: basic surgical instruments and surgical instruments.

- Basic surgical instruments
- Specialty Surgical Instruments

Medical equipment series

The medical equipment used alone or in combination on the body's instruments, equipment, equipment, materials or other items, including the required software for the human body; its role

is not achieved by means of pharmacology, immunology or metabolism, but may have the means to participate and play a supporting role; the use of which is to achieve the expected objective: prevention, diagnosis, treatment and remission of the disease, monitoring.
152.DIDI
More Than a Journey
The World ‘s Leading Transportation Platform
DiDi Global Inc. is a leading mobility technology platform. It offers a wide range of app-based services across Asia Pacific, Latin America, and other global markets, including ride hailing, taxi hailing, designated driving, hitch and other forms of shared mobility as well as certain energy and vehicle services, food delivery, and intra-city freight services.

DiDi provides car owners, drivers, and delivery partners with flexible work and income opportunities. It is committed to collaborating with policymakers, the taxi industry, the automobile industry, and the communities to solve the world’s transportation, environmental, and employment challenges through the use of AI technology and localized smart transportation innovations. DiDi strives to create better life experiences and greater social value, by building a safe, inclusive, and sustainable transportation and local services ecosystem for cities of the future.
Mission
To build a better journey

Vision
To become:
The world’s largest one-stop transportation platform
The world’s largest operator of vehicle networks
A global leader in smart transportation technologies
A global leader in the revolution in transportation and automotive technology
DIDI VALUES
To Become a Global Leader in the Revolution in Transportation and Automotive Technology
Creating Customer Value
1.DiDi exists for the purpose of creating value for customers.

2.Continue to innovate or face elimination.

3. Safety first, experience second and efficiency third.
Data-driven Thinking
1.Being data-driven is part of the way we think and the way we work.

2.We tremendously value data accumulation, and use data to make rational decisions.

3.We objectively analyze and use data.
Win-win Collaboration
1.We treat our partners and colleagues with altruistic spirit for achieve a win-win situation.

2.We have an open mind, show empathy, and trust one another.

3.We thoroughly communicate before making any decision and commit to excellent execution.

Integrity
1.Integrity is the bedrock of our foundation.

2.We communicate with honesty and openness.

2.We have courage to do the right thing and speak our mind.
Growth
1.Growth comes with accepting challenges, breaking out of your own mold, and exceeding expectations.
2.Expand our vision, think independently, self-reflect, and learn from mistakes.
3.Develop ourselves and help others grow.
Diversity
1.Diversity represents equity.

2.Diversity brings forth energy and creativity.

3.Respect and appreciate each other’s differences and embrace diversity.
CORPORATE SOCIAL RESPONSIBILITY
DiDi’s Mission Is to Make Traveling Better.
It Is Our Inexorable Commitment with
Unyielding Social Responsibilities.
Vision
DiDi believes the significance of technological transformation is to benefit broader communities, and to create higher value for society by enabling collaboration with stakeholders.
We Care
Sustainable Transport
DiDi is committed to offering convenient sustainable transport to the society. There are currently 600,000 EVs on DiDi platform, suppose a vehicle travels 150,000 kilometers a year, these EVs have the potential to reduce about 350,000 tonnes of CO_2 each year.

We Care
Accessible Transportation
DiDi is committed to offering iterative transportation solutions for populations with special needs, such as a senior citizen hotline, allowing users to request ride and pay on behalf of senior citizens, special vehicles for users with disabilities, and for mothers with babies.
We Care
Employment
In 2016, DiDi created more than 21.078 million flexible jobs and income opportunities. Around 5.719 million of our drivers are veterans or workers from industries cutting overcapacities

Driver Initiatives for Social Good
DiDi Driver Foundation: as of early 2018, DiDi has provided financial aids to 164 troubled families of drivers with illnesses.

DiDi Care Foundation: DiDi published the Health Survey on Internet Car-hailing Service Drivers jointly with China National Health Development Research Center.

DiDi Bravery Initiative: 34 "DiDi Bravery" teams in 22 cities have been established, and 473 drivers have been rewarded for their positive influence since 2016. These drivers have participated in 150 activities for social causes such as poverty alleviation, disaster relief, and quick aid.

SERVICES
Mobility Services
International Services
Financial Services
Orange Energy
TECHNOLOGY
Smart Transportation
DiDi Safety
AI Labs
Autonomous Driving
DiDi Cloud
153.HOLLEYINTL
Holley international, its predecessor was the international trade department of Holley Group, founded in 1993. Zhejiang Holley import and export co., LTD was established in 1998. In 2003, the diversified company changed its name to "Zhejiang Holley international Development co., LTD." , which is specializing in supply chain services of bulk commodities and chemical plastics raw materials, comprehensive export business of small and medium-sized enterprises, export of medical devices, export of power transmission and photovoltaic products as well as overseas electric power project contracting. Over the past two decades, Holley international has been constantly pioneering and innovating. Relying on the national "One Belt And One Road" layout, with a global strategic thinking and an open international vision, it has optimized and integrated resources as well as implemented the "going global" strategy. On the basis of mutual benefit and win-win cooperation, we will strive to become a diversified and professional high-quality supplier and overseas engineering service provider.

Main business includes:

Supply Chain Services of Bulk Commodities and Chemical Plastics Raw Materials:
Mainly engaged in DOP\DOTP\DINP\DPHP plasticizer plate series products; PVC series products; stabilizer series products; coal, calcium carbide, methanol, BDO, PBT, GBL, THF and other upstream and downstream polyurethane products; PTA, ethylene glycol, styrene and other

chemical products, the company owns the professional qualification of "Hazardous Chemicals Business License". Relying on the strong strength of Holley Group, the company actively exerts the advantages of resource integration, deeply integrates into the fields of polyester fibers, plastic soft products and petrochemical industry, provides professional supply chain services for upstream and downstream industrial customers, and actively implements the concept of green environmental protection, continuously explores the sustainable development model.

Comprehensive Export Business of Small and Medium-sized Enterprises:
Focusing on the export business of hardware tools, home textiles, outdoor light box film, outdoor tent and other decorative materials, lighting lamps, auto accessories, network cameras, safety seats, wires and cables, artificial leather and other products, as well as the import business agent issuing service. Adhering to the business philosophy of mutual benefit and win-win, we provide professional services and platforms for small and medium-sized enterprises, domestic offices of foreign companies and SOHO foreign trade talents through the global international station platform, and we are determined to be your sincere friends.

Export of Medical Devices:
In March 2020, the company obtained the medical device business record, it has the qualification to operate the first and second class medical devices. The main business includes the export of medical masks (disposable medical masks, surgical masks and protective masks) and non-medical masks (anti-particulate masks, such as N95), protective clothing and other services.

Export of Power Transmission and Photovoltaic Products: Focusing on power transmission and transformation products, photovoltaic systems and their components, instruments and accessories of Holley brand. Through more than 20 years of market cultivation, the company has established a long-term and stable cooperative relationship with foreign customers and domestic partners. Implementing technical cooperation and leading strategies in the technical field, the company actively achieve the goal of global leadership in the field of power and electrical products and systems through independent and joint research and development.

Overseas Electric Power Project Contracting:In December 2005, the company acquired the qualification of Overseas Project Contracting. Through establishing strategic cooperative relations with well-known domestic power and photovoltaic products manufacturers and power system design units, relying on the resources platform, reliable product quality, excellent marketing team and good customer relations, we have actively promoted the implementation of international strategy to open up overseas markets, and we have undertaken and completed several overseas projects, covering Africa, South America, Southeast Asia and the Middle East. We have gradually formed the core competitiveness of overseas projects (EPC).
COMPANY CULTURE
Purpose of Enterprise
Customer-centered
Spirit of Enterprise

Diligence and dedication
Team Paramountcy
Self-surmounting
Core values of Enterprise
Consensus, Joint Creation, Harmony, Share
PRODUCTS
Supply Chain Services of Bulk Commodities and Chemical Plastics Raw Materials
Agrochemicals
Comprehensive Export Business of Small and Medium-sized Enterprises
Export of power transmission and photovoltaic products
Overseas Electric Power Project Undertaking
TALENT CONCEPT
Intellectual and moral integrity — Important position
Talent without Virtue — Use with CareVirtue without talent — Seldom used
No virtue, no talent — Disuse
Character
Loyal
Coordination
154.Greenroad International Logistics
One of China's leading international engineering logistics management companies
Greenroad International Logistics was founded in 1998 and is one of the top 100 Chinese Freight Forwarding Enterprises and the top 50 Chinese Private Freight Forwarding Enterprises. Greenroad has also obtained a AAA corporate credit rating and was verified by the ISO90001 quality management system.
Selected by the Ministry of Commerce as one of the first batch of 288 national key contact enterprises in commerce and logistics in 2022, and set up overseas warehouses around the world. Today, Greenroad' s logistics specialists and branches are located around the world to provide professional solutions for project logistics.At present, Greenroad has 16 branch offices located in the port cities in China, which cover China' s major sea, land and air ports. Meanwhile, Greenroad sets up more than50 self-operated branch offices in Asia, Africa, America, Europe and other regions.During the past 24 years, its global logistics service network construction and capability has been steadily improved, products and services have been continuously innovated. Greenroad is committed to providing professional and efficient end-to-end supply chain management service platform to its clients.
A word from our CEO
Nowadays, science and technology are developing rapidly and the environment around us is changing fast .Our Society is in a period of economic structural adjustment and upgrading of traditional industries The network information age that integrates "Internet plus" in all walks of life has arrived. New industries new commercial activities and new business models are constantly emerging and the market is full of challenges and opportunities!
Facing the challenge, GREENROADINTERNATIONAL LOGISTICS adheres to the concept of "market oriented, customer-centric, and innovation for development", and continuously improve the company's resource development and management capabilities with the help of science and new technologies, and is committed to build a cooperative and open service platform, integrating

resources domestic and overseas inside and outside the industry, leading the future of the industry with quality logistics service, extending upstream and downstream industries, optimizing the industrial chain and value chain, being a resource integration and industry combination development herald, being a development and operation service provider and a market dynamic contemporary enterprise.

lt doesn't matter what you dream, but what you work hard for.

Rewards&Recgonition

License

Letters

Evaluate

IOS

Enterprise Culture

Mission Statement

Enterprise idea

We're dedicated to pave a green passage to connect corridors domestically and internationally, and provide value for our clients to eventually achieve common development

Our Mission

Establish a logistics greenway connecting various areas at home and abroad to create value for customers and achieve common development.

Customers

Adhering to rigorous and efficient artisan spirit, Greenroad strives for excellence and gains trust and support from our clients ranging from small-sized business to top global fortune 500 companies. Continuous improvement and innovation is our eternal pursuit.

Service

GREENROAD INTERNATIONAL LOGISTICS has been established for 22 years, which specilized in engineering project logistics and contract logistics, We have a professional team with proficient industry knowledge and dedication&responsibility, and experienced in the rules &characteristics of overseas and domestic market.

Meanwhile, we have created long term partnerships with many well-known container shipping lines, breakbulk cargo shipping lines, heavylift transporter and warehouse, custom clearance, inspection companies, etc of overseas&domestic in the operation of international project logistics, which can provide customers with flexible, high efficiently and cost-effectively total logistics solution!

155.CHINASTAR

GuangDong CHINESTAR Steel Structure Co. , Ltd. (Hereinafter referred to as:CHINASTAR)) was founded in 2002.The business is covering construction steel structure,curtain wall develop and apply,design consulting,processing production,construction installation and after-sales service,it's a five-in-one international construction service provider. CHINA STAR in multi.high-rise,large span and complex space steel structure construction and other fields have a leading advantage,with national professional steel structure top grade qualification,steel structure special design qualification,curtain wall design and construction qualification.The company is headquartered in Shunde district, Foshan city, Guangdong province.The domestic production base"Guangdong Sampu Garage Co. , Ltd. " is located in the national high-tech zone, Zhaoqing City, Guangdong

Province,covers an area of more than60, 000 square meters,steel structure annual production capacity of more than 40, 000tons. For overseas companies, China Star Construction (Africa) Co. , Ltd. ,is the largest steel structure enterprises in East Africa. China Star Construction (Rwanda) Co. , Ltd.has East Africa A-level construction qualification.Keep improving to make extraordinary. CHINA STAR is committed to providing brilliant construction services,excellently completed variety of assembly steel structure high-rise residential,ultra-high-rise/shaped structure,public buildings,large industrial plants,mesh frames,automobile showrooms and other types of steel structure engineering design and installation,at the same time on construction curtain wall and building general contracting construction area has made continuous progress and long-termdevelopment. We provided high-quality and professional services for many nation and aboard customers: Midea, Country garden, Mona Lisa, Dao dao quan, Mr. Lin wood industry, Yadea, Tongwei, China Evergrande, Lesso, China Resources, Yill, MengniuDairy, China Railway Construction, China Communications Construction, China Construction, Wanhe Group, Korea SeAH FS, Pohang Iron and Steel, Japan Takenaka Corporation and so on. CHINA STAR is widespread recognized by society,numerous project won the Guangdong steel structure gold award"Guangdong Steel Award"; Won the reputations for national high-tech enterprises, Guangdong Province"Abide contract&value credit' enterprises, Guangdong Province integrity demonstration enterprises.Guangdong Province famous trademarks, Guangdong steel structure business 25 years outstanding enterprise award.Building the world with peace, Rely on quality to go a long way. CHINA STAR actively respond to the national"the Belt and Road"initiative and implement the strategy of going abroad. As China's private enterprises in East Africa,we build the first international advanced technology equipped steel production and processing base:contracted to build the Rwanda International Airport expansion projects, Rwanda's ruling party headquarters and conference center, Rwanda President House expansion, Uganda International Airport expansion, Bank of Kigali multiple curtain wall projects, Mumbai International Airport.Maldives Sports Center and a number of other quality projects with international infuence Bravely climb the peak,the builders has no borders. After many years of development.CHINA STARs overseas business covers engineering construction,processing and manufacturing,real estate development,textile,neiea echnology industry,mining anecome alading overseas Chinese ente other fn Africa and Rwanda's to reinvestment demonstration enterprises Chinese President Xi Jinping met with the General Manager of China Star Construction（Rwanda) Co. , Ltd.and took a group photo on his first visit to Rwanda in 2018.in Suld a better world with our technology and services l"CHINA STAR people are taking this as their mission,forge ahead in a new era to brilliant.

Enterprise mission: To provide excellent construction services,to build a better world!

Enterprise Vision: Committed to becoming a world-class integrated service provider in the construction sector

Core values:service,commitment,innovation,win-win

Service-the attitude of doing things as a person
Commitment-Honest and trustworthy behavior
Innovation-the ability to innovate continuously
Win-win-value of win-win cooperation

Enterprise spirit:

As the most important force in the industry,following integrity as
Business concept:
professional,honest,high-quality,efficient
Service concept:
faithfully and heart-to-heart service
Quality concept:
excellence and the pursuit of brilliant
Executive concept:
constant effort to obtain fruits
Company Honor
Guangdong Steel Structure Gold Award "Guangdong Steel Award"

Company Silhouette
Chinese companies donate money and materials to support Rwanda's fight against COVID-19
April 18-19, 2019---The 14th Outdoor Development Activity of Zhongchen Steel Structure
Company Qualification
156. ZNSHINE SOLAR
Engineering design qualification certificate
Steel structure and curtain wall design qualification certificate
Leading PV Module
Manufacturer
Founded in 1988
Founded in 1988, a BNEF Tier 1 module manufacturer with over 35 years of manufacturing excellence, ZNSHINE SOLAR (NEEQ Stock Code: 838463) is a high-tech photovoltaic enterprise specializing in the R&D, manufacturing, sales and EPC services and providing whole line solutions for product applications.

ZNSHINE aims to become one of the largest utility-scale solar project developers, having its global footprints in Japan, India, Germany, Italy, Switzerland, UK, the USA, Canada, Chile, Australia and Africa.
Module Manufacturer   EPC team of 200 worldwide Investor & Developer
Mission
To make solar power become the most economical clean energy in the world.
Vision
Where there is SUNSHINE, there is ZNSHINE.
Value
Fairness and Trust

Tier 1 PV company worldwiden

China Well-Known Trademark

+17 growth in the solar business (2006-2023)

10GW projects in pipeline worldwide
History

ZNSHINE aims to become one of the largest utility scale solar Project developers, having its global footprints in Japan, India, Germany, Italy, Switzerland, UK, USA, Canada, Chile, Australia and Africa.

The Light Will Not Stop,
Neither Will The Dreams
More than a PV manufacturer, ZNSHINE SOLAR is a green energy provider, dedicated to various public welfare activities and anti-poverty programs. The company continuously provides sustainable clean energy to people. The light will not stop, neither will the dreams. ZNSHINE will continue to be engaged in providing innovative PV solutions, powering the green life.
Products
Solar Modules
Energy Storage
INTEGRATED ROOFTOP SYSTEM
EPC Services
Projects
Utility
Commercial &Industrial
Residential

Innovative Technology
ZNShine Solar R&D team in cooperation with China University of Science and Technology developed the Graphene Coating Solar Modules Series.

An innovative, self-cleaning technology capable to reduce Operation and Maintenance costs and increase power generation capacity.

Graphene is the world ’ s thinnest, lightest, most flexible, strongest, and most conductive nanomaterial. Tests have shown that graphene has hydrophilic, self-cleaning and photocatalyst properties: great benefits for solar modules.

More Power Generation
innovative, self-cleaning technology
Self-Cleaning Technology
innovative, self-cleaning technology

Low Micro-Crack Risk
innovative, self-cleaning technology
R&D Spirit
With Advanced Technology, We Provide Clients Bigger Benefits
Znshine Solar puts high importance on technology advancement and it is deep down in our spirit.

This can be seen from our products and verified by our clients and partners.

Znshine Solar co-founded the brand new R&D center with Shanghai Jiao Tong University, aiming to improve the conversion rate of solar cells and develop the photoelectric conversion technology. By cooperating with R&D labs in the US, we have improved the module efficiency, reaching an international leading level. We maintain the long term cooperation with the world-famous PV enterprises and operators, making endless efforts to improve the silicon cell architecture and module technology.

Over 100 Patents

innovative, self-cleaning technology

Over 100 Research And Development Staff

innovative, self-cleaning technology

MES/MCS/ERP Industrial Software

innovative, self-cleaning technology

Intelligent Manufacturing

Smart Manufacturing

Leveraging cutting-edge technology, Znshine Solar takes the lead in the manufacture of broad spectrum of top-notch solar panels with varying specifications and efficiencies which includes Double-Glass Mono-crystalline, Double-Glass Polycrystalline, 5BB Monocrystalline, 5BB Half-Cell Monocrystalline, 9BB Half-Cell Mono-crystalline, 5BB Half-Cell Polycrystalline, Graphene coating module products and a lot more.

As an innovative company with a laser-like focus, Znshine Solar has boasted its solar module production capacity to 5GW. Over the years, we demonstrate high level of unwavering dedication towards providing superb-quality, most-productive and high ROI solar products.

High Reliability

innovative, self-cleaning technology

30 Years Performance Warranty

innovative, self-cleaning technology

Frame Design, Easy Transportation And Installation

innovative, self-cleaning technology

Quality Control

HIGH QUALITY PRODUCTS COME FROM

STRINGENT QUALITY CONTROL SYSTEMS

Quality is the core competitiveness of ZNSHINE， To meet this expectation of high quality, we continue to invest in state-of-the art equipment and professional training of our employees . We are proud of our product quality and reliable performance even in the most extreme conditions.

ZNSHINE has been certified by the quality Management system (ISO 9001:2015), environmental Management system (ISO 14001:2015) and Occupational Health and Safety Management System (ISO45001:2018). Photovoltaic modules have simultaneously obtained TUV, CSA, BIS, KS, MCS, CQC, CEC, INMETRO, DEWA, RETIE and CE product certification, providing a guarantee for the export of products.

The company has a product performance testing center, which will play an important role in ensuring the quality of our products. The center operates in accordance with the ISO / IEC17025 system and is approved by CNAS. At the same time, it has the TUV SUD eyewitness laboratory qualification. The test covers many photoelectric performance tests in IEC, UL and relevant national standards. For the company to continuously improve the product quality, to provide scientific and accurate data.

Quality Control Points

01. Quality System zxQA+
02. High Quality Starts from the Very Beginning
03. Process Quality Control
04. The l-V testing used for performance testing
05.Information and Datamation system construction
06.Periodical Tests for Long-term Guarantee
07. Service Quality Promotion
08. Team Ability lmprovement

157. Yiwugo

Yiwugo is the world's leading e-commerce platform for wholesale procurement and retail of small commodities. It focuses on providing global buyers with services such as direct purchase of quality goods from the source and manufacturers. It is the official platform of Yiwu Small Commodity Wholesale Market. Yiwugo has moved the large-scale physical market (Yiwu International Trade Mart) to the Internet, with a unique 360° panorama display of shops. With the strength of 50,000 shops, 210,000 suppliers, and 5 million kinds of commodities, it serves as a platform for purchasers and operators. It provides controllable, credible and traceable transaction guarantees for customers and personal shoppers, allowing global buyers to view new models and select good products without leaving home, reducing purchase costs and store opening pressure.

Yiwugo, a choice of 10 million buyers. Find what you need,when you need it!

History and Milestones

2014

September 21thYiwugou.com was awarded the title of “2014 The most valuable brand of Chinese E-Commerce website” and “2014 The most promising Industry Portals Website”

2013

September 25thNews conference of Honest trading guarantee system

2012

December 25thlaunching of 360° Panoramic view technique

October 21thYiwugou.com came online.
2017

June 30thAPP new function “Grocery Online Shopping” came online

May 6thOfficially released New Project “National Pavilion”-Global Partnership Plan

January 17thYiwugou launched new propaganda slogan “Small Commodities, Big World”

2016

November 10thYiwugou won the title sponsorship of 2016 Yiwu International Marathon

October 21thYiwugou Express service came online

July 21thPresident Xi inspected Yiwugou operating center in Ningxia

June 1thcrowdfunding project came online

2015

June 4thYiwugou.com was awarded “ China ’ s E-Commerce model enterprise of the year 2015-2016”

March 18thYiwugou.com firstly released online business license in domestic market
2018

January 18thYiwugou officially change its English title to "Yiwugo"
Unique Business Mode
Yiwugo.com has combined its offline traditional markets with online Internet sales. All stores in Yiwu International Trade Mart have their online shops inYiwugo.com. By typing into the store's name/ID/Booth No, you can easily find the corresponding shop and select relevant products.
Featured products and service
YIWUGO Honest trading guarantee system&Online business license

Credit Rating
All real-name authentication shops have been marked with credit ratings and credit scores
Yiwugo Secured Transactions

Complaint Handling

Compensation Scheme For Fraud

YIWUGO APP

Available for Android & IOS devices
YIWUGO Overseas Service Station Partnership Plan

Our advantage:
Integration of professional third-party Imp&Exp facilitators, we provide one-stop cross- border transaction solutions to solve the payments, logistics, customs clearance and other issues.
158. SPH
Shanghai Pharmaceuticals Holding Co., Ltd. (“ SPH ”) is a vertically-integrated and diversified pharmaceutical group. The company has dual listings on the stock exchanges in Shanghai (601607) and Hong Kong (02067) respectively. The company provides leading healthcare services in: Research & Development, Manufacturing, Distribution and Retail.

In 2022, SPH reported revenues of US$34.5bn, and it ranked 438th on the 2023 Global Fortune 500 list, making the company one of the country’ s major forces in the industry. SPH is one of the few listed pharmaceutical companies with a leading position in both manufacturing and distribution in China. It is included in the constituent stocks of the SSE 180, CSI 300 index and MSCI.
Pharmaceuticals
Shanghai Pharma is committed to pharmaceutical innovation and the Company has been consistently increasing its investment in R&D. In 2022, SPH invested USD 416mm (CNY 2.8bn) in pharmaceutical R&D, which was 10.47% of Manufacturing Sales, and a Y .o .Y growth of 11.87%.

Through in-house innovations and partnerships, SPH is developing novel drugs. In 2022, the Company improved its innovative drug pipeline. SPH has 50 innovative products in its pipeline, 6 of which are in pivotal studies or Clinical Phase III.

- I001 (“SPH3127 Tablets”): Phase II clinical trials for efficacy and safety were completed. The tablets are used for the treatment of essential hypertension. Phase III clinical trials was underway.

- I001-B (“SPH3127 Tablets”): a major breakthrough was achieved for this innovative drug: the FDA approved the application of Phase II clinical trials for ulcerative colitis, expanding the indication for this drug.

- Prolgolimab Injection: a fully human anti-PD-1 monoclonal antibody in collaboration with Russia’ s BIOCAD, received a Notice of Clinical Trial and started Phase III clinical trials.

SPH is working with global partners on early incubation of novel drugs, construction of clinical research platforms, development of novel drugs and new indications of TCM. The alliance between SPH and universities, hospitals and research institutes facilitates new drug discovery, new processes, new therapy development and commercialization.

SPH ’ s manufacturing facilities provide products ranging from chemicals and biochemicals to

modern TCM (Traditional Chinese Medicine), healthcare products and medical devices. SPH’s core therapeutic areas are Oncology, Cerebrocardiovascular, CNS, General Infection, Immunology, Digestive and Metabolism, Respiratory.

The Company has manufacturing bases in 12 provinces in China, including quality API (Active Pharmaceutical Ingredient), modern TCM (Traditional Chinese Medicine), fine chemical reagent factories and nutrition & supplements production. The products are manufactured in over 20 dosage forms. 48 products have sales revenues of over USD 14.87mm (CNY100mm).

SPH has implemented a Lean Management System, optimized production and conducted pilot programs to integrate multi-IT systems in the manufacturing process to increase output. A number of its APIs and preparations have passed quality authentications from the WHO, FDA, EU and other ICH member countries.

Pharmaceutical Services

SPH is the second largest Pharmaceutical distributor and the largest drug importer in China. It owns over 2,000 pharmacies and its distribution network covers 32,000 hospitals in 25 provinces, municipalities and autonomous regions.

SPH keeps innovating its business model, and is committed to becoming a service and technology-driven healthcare provider, offering integrated and creative solutions to our partners and people across China.

SPH Health Commerce, a leading Internet + Med-tech in China, is building its own Yiyao ecosystem:

- Separation between Dispensing and Prescription (SDP)

- The largest specialty drug network in China (22 Provinces covered)

- The largest public electronic prescription platform (10mm issued In 2019)

- A leading service provider in clinical trials, medical aid, physicians and patients’ education, e-prescription management, remote and cloud hospitals.

Meditrust Health, another SPH subsidiary in the healthcare insurance business, launched 20+ new projects in 2020, and issued 14mm insurance policies (a total of 20mm policies between 2018 Q3 To 2020 Q4). Its Yaokangfu drug welfare platform has become the largest patient welfare platform in China. The special drugs commercial insurance Yiyaobao has 90% of the Chinese market share. By cooperating with local governments, it has launched customized urban supplementary medical insurance in Suzhou, Hangzhou and 32 other cities, accounting for 50% of its market share.

R&D

SPH believes in centralized management, and its R&D Center is overseeing the group's innovation activities：

SPH Centre Research Institute is the Innovative Core
Monoclonal Antibody,ADC,Chemical(API & Formulations), Pilot Test & Manufacturing, Cell Therapy
San Diego, Hong Kong R&D Center
R&D GOALS
Pioneering safe and effective drugs for chronic and serious diseases
R&D STRATEGY
lnnovative Drugs
Innovate both in-house and through partnerships
Generic Drugs
Develop high quality and efficient generic drugs
Life Cycle Management
Prolong product life cycle to meet patients' needs
R&D WORKFORCE
1500+ staff involved iu R&D
More than 45% have postgraduate degrees
2014，2015 FIRST PRIZE OF THE NATIONAL AWARDFOR SCIENCE AND TECHNOLOGY PROGRESS
Qingchunbao cooperated with Tianjin University of TCM:Mechanism, production progress and QA program of TCM lifecycle management
Shanghai Traditional Chinese Medicine Co.cooperated with Institute of Materia Medica: The development and industrialization of synthetic musk
SEVEN THERAPEUTIC AREAS
Digestive & Metabolic
Immune System
Anti-infection
Oncology
Cerebral &Cardiovascular
Central Nervous System
Respiratory
Manufacturing
SPH's Manufacturing Management Center isimplementing the Made in SPH 2025' strategyThis is building a state-of-the-art manufacturingsystem.SPH provides top-rated drugs withemphasis on safety, quality and efficacy
ADVANCED MANUFACTURING SYSTEM
Advanced Management
Advanced Technology
Advanced Equipment
Advanced Efficiency
Streamlined and Efficient
Rapid Response
High Quality
Low Cost
Intelligent Manufacturing
Environment-Friendly

Quality Principle: SPH's reputation is dependent on its high quality products
Quality Objectives: “Full marks” for in-house QC and regulatory checks
“Zero-defect” in quality certification and routine defects regulatory checks
Manufacturing Management:
Manufacturing optimization through resource centralization and management standardization
Tech Support:
Cost-management, cross-plant tech exchange, new product introduction
Project Management:
Purchase and recycle equipment
Oversee the design of manufacturing plants
Corporate Culture
MISSION
Committed to lmproving Health and Quality of Life
VISION
An Internationally Trusted and Competitive Biopharmaceutical and Healthcare Provider
CORE VALUES
Innovation,Integrity,Cooperation,Inclusiveness and Responsibility
Enviro-Protection & Sustainable Development
STRATEGIC GOAL
Build an energy-efficient and environmentally friendly enterprise based on proper and effective management and monitoring systems
ENVIRONMENTAL PROTECTION
ldentify the scope for environmental protection; use the right tools to implement and monitor protection policies;audit and assess the performance of environmental protection through the enterprise
CLEAN MANUFACTURING
Design and implement a manufacturing auditing plan for clean manufacturing:
- Increase the use of recyclable goods, the rate of water reuse
- Decrease manufacturing waste
SUSTAINABLE GROWTH
Ensure sustainable growth of the enterprise by implementing effective environmental control measures with an emphasis on prevention. ldentify the root cause of any issues and control the whole manufacturing process.
ENERGY CONSERVATION
Lower the year-on-year energy consumption per RMB10,000 of industrial output by:Implementing and promoting sound energy conservation measures
- Optimizing our manufacturing systems
- Training SPH staff in energy conservation
Focus on Public Welfare
SPH is deeply committed to charitable causes as a means of promoting social equality, achieving common prosperity and strengthening the5ocial security system.Guided by its mission to "persistently improve people's health and quality of life, SPH takes its corporate social responsibility seriously and has a long history of active involvement in public welfare and charity, giving back to society, people, and the nation through proactive, sustained, and standardized

charitable actions.

Standardizing the Management of Charitable Programs

SPH is dedicated to exploring sustainable ways to manage its charitable programs. During the Reporting Period, in strict adherence to the Charity Law of the People's Republic of China and the Law of the People's Republic of China on Public Welfare Donations, SPH introduced thenterim Measures of SPH for the Management of Charitable Programs. Through effective means such as categorization, budgeting, hierarchical approval, and record-keeping, SPH established a mechanism for managing charitable donations, thus enhancing proactivity, sustainability, and standardization of these programs.Currently, SPH's charitable programs encompass categories such as health, science, education, culture and sports, social welfare, disaster relief, and rural revitalization.

159.Humanwell

Founded in 1993 by a group of passionate college graduates, Humanwell Healthcare has grown into a fully integrated healthcare solutions provider. With headquarters in the center of China, Humanwell operates and serves its customers in over 50 countries and districts. The company’s vision is to be the leader of each market segment it choses to participate, by providing its customers the highest quality products, the most comprehensive healthcare solutions, at the affordable prices.

Humanwell is the market leader in anesthetics/analgesics, fertility regulation drugs, and Uyghur medicine in Asia. It is also a major player with expanding product portfolio and market shares in treating central nerve system (CNS), respiratory and dermatological diseases in China.

Humanwell’s core value centers on integrity, equality and humanity. Its mission is to benefit humankind (as it is reflected in the company’s name) by improving human healthcare around the world through our commitments to patients, customers, shareholders and employees. By leveraging our global resources and partnerships, the company strives to challenge the unmet medical needs of every patient and customer around the world.

Over the last two decades, Humanwell has achieved phenomenal growth and expansion.From 2011 to 2017, our operating revenue has grown at an annual rate of 23.64%, twice of the industry average. In 2017, the operating revenue reached $2.3 billion and the netprofit was $345.97 million.

MANAGEMENT TEAM

Our management team drives Humanwell’s continuing success with their combined passion and expertise.

OUR VALUES

Vision

To be a lasting global brand renowned for top quality healthcare solutions.

Mission

To be a lasting global brand renowned for top quality healthcare solutions.

Humanwell envisions to be a lasting global brand renowned for top quality healthcare solutions. It’s Humanwell’s mission to provide better healthcare solutions by leveraging global resources and live up to the commitments to our patients, customers, shareholders and employees.

Core Values

Integrity • Equality • Humanity

Humanwell is committed to our core values of integrity, equality, and humanity. These values embody how Humanwell operates and is the fundamental of our business strategy. They are the unceasing support to keep us challenging the unmet medical needs of every patient around the world.

FOOTPRINTS

Humanwell strives to be the leader in targeted market segments.Humanwell is determined to provide global customers with top quality and affordable products by leveraging our superior manufacturing base in central China.

Humanwell, together with CITIC Capital, acquired the Sexual Wellness Business of Ansell, now renamed as LifeStyles Healthcare. LifeStyles Healthcare is the world's No. 2 condom and sexual wellness product marketer, with products sold to more than 55 countries. It’s products include a range of branded premium and middle-market condoms, lubricants and devices. These products are sold at retail outlets through a combination of distributors, direct to key retail partners or e-commerce platforms.

Humanwell stays true to its core values of equality and believes in access to equitable healthcare services.

Humanwell expanded its presence to Africa to provide equitable healthcare solutions to a region with tremendous unmet medical needs but historically under-served by the global pharmaceutical industry. The company established Humanwell Pharma Mali S.A. in 2009 and Humanwell Pharma Burkina S.A. in 2010 to specifically serve the Economic Community of West Africa States (ECOWAS) countries.

Humanwell (Africa) Pharmaceutical S.A. started to build a manufacturing facility under the current GMP standard to meet the tremendous needs of essential drugs in Africa. The Mali pharmaceutical manufacturing facility, the first of its kind in West Africa, was later adopted as the industry standard by the Mali government. In 2015, Humanwell Pharmaceutical Ethiopia PLC started to build a manufacturing facility in Ethiopia, which is expected to be completed in 2017, to serve patients in East African countries.

PRODUCT

Anesthetics/Analgesics

Women's Health

CNS DRUGS

Herbal Medicines

Anti-infection Drugs

OTC Medicines

Other Products

R&D

Humanwell has set up five research centers in China, the United States and Europe. In the United States, it has more than 70 approved ANDAs, more than 100 OTC SKUs, and more than 20 drug candidates in various stages of development. The company spends approximately 5% of its revenue in research and development, which ranks it in the top R&D investment group among its domestic pharmaceutical peers. Currently, Humanwell has 218 drugs in development, including 28 new drugs and 9 new chemical entities (NCEs) in various stages of clinical development.

R&D TEAM

Humanwell's remarkable R&D team is the driving force behind Humanwell's innovation and powers the quest for better solutions to the vast healthcare challenges in the world. Currently, Humanwell has over 1200 scientists, 66 of which have Ph.D. degrees.

160.GCIGC/CGICO

As the overseas operation platform for Gansu Construction Investment (Holdings) Group Co., Ltd. (hereinafter referred to as "GCIGC"), China Gansu International Economic and Technical Cooperation Co., Ltd. (hereinafter referred to as "CGICO") is a multinational diversified company with the business scope as follows:

- International Project Contracting,
- Import and Export Trade,
- Economic and Technological Cooperation,
- Labor Service Collaboration,
- Pharmaceutical Manufacturing,
- Hotel Management,
- Overseas Real Estate Development,
- General Contracting of Domestic Housing Construction. The certificates issued by various institutions:

- Grade A General Contractors of Housing Construction,
- Grade A Qualification for General Contracting of Mechanical and Electrical Works Construction,
- Qualification for Implementing General Contracting for the Overseas Construction by the Ministry of Commerce,
- Qualification for General Contracting Enterprise for China-Aid Project Construction by the Ministry of Commerce,
- Qualification for Implementing China-Aid Technical Assistance Project.
- Certification of Quality,
- Certification of Occupational Health and Safety,

• Certification of Environmental Management.

CGICO is the only enterprise in Gansu province which has been listed among the Top 250 International Contractors by ENR every year since 2014, and was honorably entitled to the national Certificate of AAA Credit. In addition, CGICO was identified as one of the top 100 enterprises in China in terms of foreign contracted project business (newly signed contract amount) by the Ministry of Commerce. Due to the positive efforts made in promoting the construction of "the Belt and Road" in recent years, CGICO was praised by office of the leading group for promoting the construction of "the Belt and Road" of the National Development and Reform Commission and won the title of Outstanding Company of " the Belt and Road ".

The company's first overseas project, the construction of China-aid project in Togo, "La Mansion du Rassemblement du Peuple Togolais", was undertook in 1978. At present, CGICO has established an overseas business management system with business department system as the core. Its main business covers more than 40 countries and regions such as Africa, Europe, Asia, Oceania and Latin America etc. With its branches in more than 20 countries, CGICO has formed the following markets:

• The East African market centered in Ethiopia and Kenya,

• The West African market centered in Ghana, Radiating to Côte d'Ivoire and Sierra Leone,

• The South African market centered in Angola radiating to surrounding countries such as Zimbabwe, Malawi, Mozambique and Namibia,

• The Middle East market centered in Saudi Arabia and covering the United Arab Emirates and Jordan,

• The East European market centered in Belarus, covering Russia and five Russian speaking countries,

• The South American market centered in Brazil and covering Suriname, Argentina, and

• The South and Southeast Asian market centered in Pakistan and Indonesia, covering India, Cambodia, Vietnam and other countries.

CGICO takes international project contracting and international trade as its main business, implements the management mode of "one country, one policy", carries out diversified

operation and investment, and expands overseas markets in an all-round way. The company adheres to the overseas development strategy of "four kinds of localization" (management localization, operation localization, personnel localization and capital localization), takes the integration of investment, construction and operation as the development mode, persists in business strategy, "capacity globalization, market localization, industry revitalization, profits repatriation", and gradually transforms into an international first-class service provider for the full cycle of project construction.

CGICO has inherited overseas spirit from GCIGC for more than 40 years, namely: hard working, the courage to challenge, the courage to innovate and pioneering. All the staff will continue to strive for excellence, keep promises and cast quality. A large number of overseas projects with great social influence and high quality have been completed and many of them have become the landmark buildings of the host country. The company has constantly built new monuments of friendly exchanges between China and foreign countries. The projects awards are as follows:

- "Golden Mercury International"----"La Mansion du Rassemblement du Peuple Togolais" project in Togo.

Zimbabwe National Stadium was honored as "the monument to the friendship of people between China and Zimbabwe from generation to generation".

National Theater of Côte d'Ivoire was honored as "the symbol of friendship between China and Côte d'Ivoire, the fruit of South-South Cooperation".

The TV Production Center and Administration Complex in Republic of Angola was known as "the most modern landmark architecture" in Angola.

The overseas constructors of CGICO cherish their families and countries and have the courage to make contributions. They have constantly created "Chinese speed" and "Chinese quality" with their actions. Multiple projects have won the praise of the heads of government of the host country and investors, and recorded brilliant achievements.

In the future, CGICO will keep leading high-quality development with new development concept, speeding up the construction of a new development pattern of mutual promotion of domestic and foreign dual circulation. It will cultivate new advantages for the company to participate in international cooperation and competition under the new situation through seizing development opportunities of "the Belt and Road" and fully integrating into the national strategy and opening-up development plan of Gansu Province. The company is going to constantly improve comprehensive strength, competitiveness and risk resistance, striving to make the company stronger, better and greater, and making efforts to forge a first class enterprise with international influence and competitiveness.

Certificates

Special Class Qualification for General Contracting of Building Works Construction

First Class Qualification for General Contracting of Mechanical and Electrical Works Construction

First Class Qualification for General Contracting of Power Works Construction

First Class Qualification for General Contracting of Municipal Public Works Construction

First Class Qualification for General Contracting of Petrochemical Works Construction

First Class Qualification for General Contracting of Building Works Construction

First Class Qualification for Special Contracting of Nuclear Works

First Class Qualification for Special Contracting of Environmental Protection Works

First Class Qualification for Special Contracting of Steel Structure Works

First Class Qualification for Special Contracting of Ground Foundation Works

First Class Qualification for Special Contracting of Building Curtain Wall Works

First Class Qualification for Special Contracting of Fire Protection Facilities Works

First Class Qualification for Special Contracting of Waterproof, Corrosion-Proof and Insulation Works

First Class Qualification for Special Contracting of Building Mechanical and Electrical Installation Works

First Class Qualification for Special Contracting of Lifting Equipment Installation Works

First Class Qualification for Special Contracting of Electronic and Intelligent Works

First Class Qualification for Special Contracting of Building Decoration Works

Class A of Building Industry (Building Works) Design

Class A of Building Industry (Air Defense Works) Design

Class A of Building Decoration Works Design

Class A of Building Works Inspection

Class A of Geotechnical Works Inspection

Class A of Municipal Works Inspection

Class A of Building Construction Works Supervision

Class A of Municipal Public Works Supervision

Class A of Highway (Bridge) Works Inspection

Class A of Building Works Indoor Environment Inspection

Class A of Ground Foundation and Main Structure Inspection

Class A of Works Investigation Major (Geotechnical Works) Investigation

First Class Qualification for General Building Construction Issued by Ministry of Construction in Ghana

First Class Qualification for Water and Electricity Installation Construction Issued by Ministry of Construction in Ghana

First Class Road Qualification Issued by Ministry of Roads and Highways in Ghana

First Class Qualification Certificate for Road Works

First Class Qualification Certificate for Electrical Works

First Class Qualification Certificate for Building Construction Works

First Class Qualification Certificate for Mechanical Works

Qualification for Civil Construction

Qualification for Installation Works

Qualification for Road and Bridge Works

Class A Qualification of Building Industry Association
Enterprise Value

Enterprise style: unity, hard work, cooperation, pragmatic, integrity

Enterprise philosophy: excellence, rigorous and efficient, beyond the self, innovation

Business philosophy: internationalization, integration, diversification, specialization, localization, informatization

Core values: develop the enterprise, carefully guide personal growth; Mutual benefit and win-win, and strive to promote customer success; Forge ahead and take up social responsibilities

Corporate vision: to build a respected multinational enterprise and create an international brand image

Enterprise mission: develop the enterprise, enrich the staff and benefit the society

Enterprise positioning: to build a first-class international construction operator
BUSINESS
Construction & Installation
Road & Municipal
Industrial Construction
International Trade
Labor Export
Diversified Industries
161.CJ Smart Cargo Group
CJ Smart Cargo Group (Qingdao CJ Smart Cargo International Logistics Co., Ltd./Shanghai SMART CARGO SUPPLY CHAIN MANAGEMENT Co., Ltd.) provides you with leading integrated smart logistics solutions worldwide. Through core business such as marine engineering logistics, EPC engineering logistics, oil and gas chemical logistics, and providing innovative, personalized, integrated and intelligent expert logistics services to world-class enterprises in African supply chains, overseas warehouses, factory relocation and other innovative business areas. CJ Smart Cargo Logistics is an EPC logistics general contractor under China's “Belt and Road” national strategy. It is also trusted by the government and scientific research institutions. It cooperates to build the industry's first engineering logistics supply chain management big data platform to serve as an EPC along the “Belt and Road” Enterprises provide data decision-making services.

The CJ Smart Cargo logistics network covers 9 industrial cities and ports in China and has 15 self-operated branches overseas. The business scope covers 50 major ports and inland cities in

five countries of Southeast Asia, Middle East, Africa, South America, Europe and Central Asia, and has become a strategic partner with more than 200 overseas agents. CJ Smart Cargo Logistics is a national high-tech enterprise, 2017 Ernst & Young Fudan's most promising enterprise technology giant company, national top 100 logistics enterprise, and WTO China-Africa Cooperation Forum recommended enterprise. It independently owns 28 national software intellectual property rights to provide you with global smart EPC High-end experience of supply chain operations.

Company Culture

value

We can only be HAPPY when Our Clients are SATISFIED and HAPPY to our work.

The BENEFIT of the Company should be Protected to the greatest extent.

Treat each other with an OPEN MIND, and the Team that WORKS TOGETHER is the most EFFICIENT.

The one who takes RESPONSIBILITY of everything and everyone Now, will be SUCCESSFUL in the Future.

The YOUTH who DARE to INNOVATE and STRUGGLE are the most EXCELLENT and PRIDE.

Mission

The Lighthouse of “the Belt and Road” Initiative

Vision

Presence in 50 Countries around the world.

Support and Assist 1 million Chinese Companies' Global Development.

Offer 10 thousand Job Opportunities to local people in 50 Countries.

CJ SMART CARGO in Africa

CJ SMARTCARGO has branches in Tanzania, Kenya, Mozambique, South Africa, Nigeria and Ethiopia. And plans to open branches in Togo, Algeria and Ghana in the future; CJ SMARTCARGO network will cover East, West, South and North Africa. In order to vigorously develop the supply chain business in Africa, CJ SMARTCARGO possesses its own customs clearance company, fleet and warehouse, which can provide various customized services such as customs clearance, transportation, storage, trade finance and trade risk control. The company focuses on creating exclusive services for every customer stationed in Africa and becoming the "African consultant" beside you.

In 2016, Ms. Lyu Cuifeng, President of CJ SMARTCARGO, was officially appointed Director of the Shanghai Representative Office of the WTO China-Africa Cooperation Forum, dedicated to the progress and development of Africa. CJ SMARTCARGO will continue to use its own advantages to serve Africa, and is willing to work with the international community and enterprises to continue to help African countries and regions to the best of its ability, to achieve

common prosperity and development.

Business area

Home Business area CJ SMART CARGO in Africa CJ SMART CARGO in Africa

CJ SMART CARGO in Africa

CJ SMART CARGO in Southeast As

CJ SMART CARGO in the Middle E

CJ SMART CARGO in South Americ

CJ SMART CARGO in Africa

CJ SMARTCARGO has branches in Tanzania, Kenya, Mozambique, South Africa, Nigeria and Ethiopia. And plans to open branches in Togo, Algeria and Ghana in the future; CJ SMARTCARGO network will cover East, West, South and North Africa. In order to vigorously develop the supply chain business in Africa, CJ SMARTCARGO possesses its own customs clearance company, fleet and warehouse, which can provide various customized services such as customs clearance, transportation, storage, trade finance and trade risk control. The company focuses on creating exclusive services for every customer stationed in Africa and becoming the "African consultant" beside you.

In 2016, Ms. Lyu Cuifeng, President of CJ SMARTCARGO, was officially appointed Director of the Shanghai Representative Office of the WTO China-Africa Cooperation Forum, dedicated to the progress and development of Africa. CJ SMARTCARGO will continue to use its own advantages to serve Africa, and is willing to work with the international community and enterprises to continue to help African countries and regions to the best of its ability, to achieve common prosperity and development.

Tanzania Heavy Vehicles

Tanzania Liuhe Heavy Cargo International Transportation Company, owned by CJ SMARTCARGO, is the only Chinese company in East Africa that specializes in inland transportation of major parts and equipment. Boasting many professional transportation vehicles and equipment such as low flat plates and hydraulic axle plates and equipped with experienced technology and Chinese operators, Liuhe can customize professional logistics plans for heavy cargo for customers to ensure the safe, successful and timely arrival of heavy cargo at their destinations. Relying on the network of brother companies all over East Africa, we can realize inland transportation throughout Southeast Africa, including large cargo, bulk cargo, containers, cross-border transportation, etc.

Tanzania Customs Clearance

CJ SMARTCARGO has a wholly-owned customs declaration in Tanzania with a history of

10 years, which can carry out customs clearance for local import, export and transit businesses. It has an excellent understanding of customs policies, with especially rich experience in handling duty exemption form, temporary import and export of goods under construction, etc.

Nigeria Team

SINOMA CARGO is a subsidiary of CJ SMARTCARGO, established in October 2015 and located in Lagos, Nigeria. It is the first branch set up in West Africa in line with CJ SMARTCARGO's globalization strategy; it will also be a base of CJ SMARTCARGO in West Africa.

Equipped with 16 ordinary flatbed trucks, 10 low-beds and 2 cranes; Special fleet site, maintenance workshop and spare parts warehouse; Self-provided refueling equipment, oil tanks, Chinese employees: 8 local staff: 30 maintenance workers: 5.
Solution
EPC Project Logistics

Committed to the logistics operation of various domestic and overseas engineering projects, CJ SMARTCARGO provides personalized and all-round logistics services. We will comprehensively analyze customers' logistics needs, provide sound overall logistics plans, and formulate elaborate emergency measures, to ensure that customers' logistics needs at different stages are met in the safest and most economical way. We believe customers will definitely benefit from our professional services.
With rich experience in the logistics operation of cement plants, steel plants, power plants, oil and gas refineries and other construction projects, CJ SMARTCARGO can comprehensively utilize various modes of transportation, namely highway, railway, inland-water transportation, ocean shipping, harbour handling, import and export customs clearance, in-position large-scale equipment workshops, etc., to insure the smooth progress of projects.

Marine project logistics

As a leader in the marine engineering module transportation, CJ SMARTCARGO is specialized in ro-ro, translation and maritime transport services for over-limit structures and major modules.

We boast rich experience in project design and operation and hammer at providing creative solutions to customers. We dabble in multiple fields such as ocean, energy and electricity.

We can transport your overweight cargo anywhere in the world through our own SPB and SPMT.

Oil and gas chemical logistics
CJ SMARTCARGO has long been committed to providing professional logistics and transportation solutions for major oil, gas and chemical engineering customers. With abundant professional knowledge and operational experience, we can offer customers all-round and integrated

land-air-sea logistics and transportation services.The business areas involved include oil exploration and production equipment such as drilling machines in upstream, transportation of pipelines and ships in midstream and global transportation of refining and chemical plant equipment in downstream. The type of transport ships used include multi-purpose cargo ship, self-propelled barge, tugboat, semi-submerged ship, etc.

Ro-Ro Logistics Services

CJ SMARTCARGO has transported a large number of vehicles from China to all parts of the world. The transportation vehicles include ro-ro ships and general cargo ships. It has accumulated rich operating experience and established good cooperative relations with many ro-ro and general cargo ship owners. Able to provide customers with more professional and economical vehicle transportation services.

CJ SMARTCARGO delivers many different vehicles from China to worldwide every year, using RO/RO vessel and break bulk vessel, and has built very close relationship with many ship owners. With rich experiences, CJ SMARTCARGO can provide professional and economical transportation service to our clients.

Africa Supply Chain Services

CJ SMARTCARGO has branches in Tanzania, Kenya, Mozambique, South Africa, Nigeria and Ethiopia. And plans to open branches in Togo, Algeria and Ghana in the future; CJ SMARTCARGO network will cover East, West, South and North Africa. In order to vigorously develop the supply chain business in Africa, CJ SMARTCARGO possesses its own customs clearance company, fleet and warehouse, which can provide various customized services such as customs clearance, transportation, storage, trade finance and trade risk control. The company focuses on creating exclusive services for every customer stationed in Africa and becoming the "African consultant" beside you.

In 2016, Ms. Lyu Cuifeng, President of CJ SMARTCARGO, was officially appointed Director of the Shanghai Representative Office of the WTO China-Africa Cooperation Forum, dedicated to the progress and development of Africa. CJ SMARTCARGO will continue to use its own advantages to serve Africa, and is willing to work with the international community and enterprises to continue to help African countries and regions to the best of its ability, to achieve common prosperity and development.

Supply chain finance business

1. Automobile trade supply chain finance (P2P + supply chain management + insurance)
2. Cross-border e-commerce supply chain finance (bank + supply chain management + insurance)
3. Sino-Africa supply chain finance (self-owned funds + trade financing + insurance)

162.CBVAC Technology Co., Ltd

Zhongke Nine Micro Technology Co., Ltd. is a high-tech company focusing on the vacuum field. Its products include vacuum pumps, vacuum valves, vacuum chambers, vacuum measurement and vacuum components, which are widely used in semiconductor, photovoltaic solar energy, biomedical research institutes and other fields. Its main customers are located in more than 60 countries and regions around the world. Zhongke Jiuwei spared no effort to develop innovative technologies, excellent solutions and products, enable high-end manufacturing with vacuum

technology, continuously create value for customers, and inject power into the development of the industry.

At the same time, China Science and Technology Ninth Microenterprise has vigorously developed the innovation ecology, joined hands with the government, enterprises, colleges and research institutions to achieve collaborative innovation. The multi win company has been recognized as a national high-tech enterprise, engineering research center, and has set up innovation research institutes. It has completed more than 10 national product research tasks and undertaken many major projects such as the National Development and Reform Commission, the Ministry of Science and Technology, and the Ministry of Industry and Information Technology. At present, more than 350 excellent R&D and engineering technicians are gathered under Sinopec Jiuwei, and many R&D centers, such as high vacuum, low vacuum and vacuum valves, have won more than 400 effective patents and patent applications.

In Shunqing, Nanchong, Sichuan and Xichong, Zhongke Jiuwei has built a 450 mu semiconductor vacuum production base and a 108 mu industrial vacuum production base, built an industrial 4.0 intelligent digital workshop, and has advanced processing equipment and strong manufacturing capacity. It is a highly representative advanced manufacturing center in China's vacuum field.

Based in China and looking at the world, Sinopec Jiuwei takes honesty, efficiency, responsibility and sharing as its values, customer as its center, and the mission of making China's high-end manufacturing go global. It is committed to becoming a world-famous vacuum technology enterprise, enabling scientific and technological progress with vacuum technology.

Vision

To become a world-famous vacuum technology enterprise, enabling scientific and technological progress with vacuum technology

Goals

Create a leading vacuum technology enterprise and achieve a century old foundation.

Mission

Focusing on customers, let China's high-end manufacturing go global.

Sense of worth

Honesty, efficiency and responsibility sharing.

The original intention of Seiko innovation

Zhongke Jiuwei, with the original intention of helping the development of the semiconductor industry and taking enabling high-end manufacturing as its own responsibility, is a semiconductor core equipment and core component manufacturer specializing in integrating independent scientific research and development, intelligent manufacturing, sales and service. The company's R&D headquarters is located in Beijing Yizhuang Development Zone. It is planned and designed in Nanchong using the advanced standards of the foreign semiconductor industry, and has built a 170000 square meter intelligent manufacturing plant

Endless exploration

Zhongke Jiuwei has the courage to explore breakthrough technologies. Its main core products include dry vacuum pump, magnetic suspension molecular pump grease lubricating split pump, semiconductor vacuum chamber, etc. Its products are widely used in more than 60% of semiconductor manufacturing processes such as wafer growth, film deposition, ion implantation, etching, etc

A delicate attitude

Zhongke Jiuwei, introducing a large number of professional talents from domestic and foreign industries, focuses on strengthening the R&D, intelligent manufacturing, quality and other aspects, focuses on improving the overall solutions of customers in the semiconductor manufacturing market, and strengthens the service quality with a more delicate and exquisite attitude, making unremitting efforts to meet the differentiated needs of customers

Continuous and far-reaching impact

Zhongke Jiuwei firmly believes that science and technology should help mankind overcome major problems and challenges together. With advanced semiconductor core equipment and core components R&D, intelligent manufacturing, and technical service teams, we continue to serve enterprises and society, help people pursue a better life in a new way, and make the world more harmonious

Honor

Engineering Research Center

163.China Hway Group

China Hyway Group Limited (the Group), registered in Hong Kong with registered capital of HKD 1.5 billion, is an international diversified enterprise. Its main business scope covers the general contracting of international projects, exploitation of resources and trade, international trade, and management &consulting on investment etc. It is the first and only private enterprise in China that has the overall contract performance of overseas large-scale railway survey, design and construction. The group has nearly 20 subsidiaries (branches) and offices in more than 10 countries and regions in Asia (including the Middle East), Africa, Europe and the United States, and has more than 5,000 (sets) of various construction equipment, including: railway track-laying machines, locomotives, asphalt mixing plants, asphalt pavers, excavator, road rollers, girder-erecting machines, tower cranes, etc., with an annual production capacity of USD 2 billion. Adhering to the development principle of "Talents are the first productive force", the group adopts an innovative talent training mechanism of bringing in and going out. It has more than 500 senior technical and management personnel, and has also engaged many experts in various fields as professional consultants. It also has established long-term and stable partnership with numerous domestic & international companies, banks and insurance companies. The group has industry-leading planning and design capabilities, engineering construction capabilities, project management and control capabilities, and core competitive advantages in railway, highway, municipal and water conservancy and hydropower engineering, construction and steel structure construction, chemical and petroleum equipment pipeline installation engineering construction, international logistics and international trade, mineral resources exploration and exploitation, ecological agriculture and other fields.

Principal business performance of Group in recent years:

1. General Contracting of International Projects:

In Angola, the Group has contracted Mocamedes Railway Project by way of survey, design and general contract. This railway starts from Namibe Port in the west to Menongue in the east with a total length of 1003. 1km and it connects 56 stations. It is one of the three trunk railway lines, as

well as EPC+F generally contracted luanda Railway extension and its five integrated hub station projects. In Ethiopia, we have successively undertaken the construction of four loan projects in use of World Bank Standard Bidding and Management, with a total length of 244.6km, with excellent project performance capability of high standard; In Serbia, the Group undertook the construction work of E763 Highway; In Saudi Arabia, the Group executed the construction of Al-jouf University Hospital Project with a total floor area of 120,000m^2; In Malaysia, the Group completed Commercial Office Building Project in Sabah; In Mongolia, the Group completed the construction of municipal road in Ulanbatar.

2. Trade and logistics:

In Asia, Africa and other countries and regions, it has built an international logistics network that integrates highways, railways and other transportation capacities, and has its own multiple logistics bases in the countries where it operates businesses. Signed a land contract in Angola, established the Matara Agricultural Development Demonstration Zone, and realized a new and modern comprehensive agricultural project of mechanized planting and harvesting.

3. Real Estate Development:

The Group invested and constructed a 5A Smart Building in Suzhou of China with a project construction area of 95138m^2; and invested the development and construction of XinMinZhou Industrial Park project in ZhenJiang of China with an area of 10km^2; And Houyunwan Real Estate Development project in Yangzhong, Jiangsu Province with an area of about 137,000m^2.

China Hyway Group Limited, adhering to the philosophy of "Creating value for the owners and partners", actively builds a cohesive and inclusive corporate culture, and sincerely cooperates with owners to make the common progress and development and create brilliance together.

4. Qualification Honor

China Hyway Group Co., Ltd. is one of the top 100 China’s foreign contracting companies in 2017 by the Ministry of Commerce, and has attracted the attention of CCTV, Phoenix Satellite TV, Tianjin Satellite TV and many other media.

Meanwhile, it is entitled of:

Vice President Unit of China-Africa Business Council

Standing Director Unit of China Chamber of International Commerce

Member unit of China International Contractors Association

Member Unit of China Chamber of Commerce for Import and Export of Machinery and Electronic Products

Member Unit of Tianjin Foreign Economic Cooperation Association

5. Social Responsibility

The ability determines altitude, and height symbolizes responsibility. While working hard to develop the market, China Hyway Group has always kept its mission and social responsibilities in its mind. In the construction of long-term international projects, it has also been actively participating in the construction of local livelihood infrastructure of the project, and making a contribution to the local society of the project.

In 2014, with the support of the leaders of the Chinese Embassy in Angola and the Economic and Commercial Office, China Hyway Group, together with the Angolan government, the Ministry of Transport, and the Ministry of Higher Education, jointly launched a talent training program. A total of 32 outstanding Angolan youths were fully funded by Hyway to go to China for their education. It opened a precedent for jointly running school between China's "Going out" private enterprises and domestic universities, which contributed to China-Africa economic and trade cooperation and cultural exchanges, and received strong support and care from the Angolan government, the Chinese Ministry of Commerce and the Ministry of Foreign Affairs and other relevant leaders.

In March 2015, Hyway Group donated 200 boxes of mineral water, 20 boxes of big tents, 20 boxes of compressed biscuits, 200 pairs of rubber shoes and a batch of urgently needed materials to the disaster area, located in Lobito City, Benguela Province, Angola, which was flooded by heavy rains, and sent the personnel to the scene for comforting the local victims.

In June 2017, the donations for the Khalida Central Primary School in Huíla Province included 6 container classrooms, and some sports and school supplies.

In January 2017, Hyway Group (Malaysia) Sdn, Bhd., as the first batch of volunteers, actively cooperated with the Chinese Consulate in Sabah to comfort and escort to the compatriots rescued from the "1.28 Shipwreck" incident, which was given a special report by Phoenix TV, Xinhua News Agency and other media.

In March 2019, Hyway Group made donations for disabled children in Malaysia.

In April 2019, all Malaysian employees have taken part in participated in the 4th "Heros RUN" event organized by the Seri Mengasih charity in Malaysia.

In July 2019 and 2020, the party branch of the group company participated in the east-west poverty alleviation cooperation and support cooperation, with a donation of a total of 10,000 Yuan.

On December 23, 2019, the Angola branch of the group company entered the Muxima

Orphanage on Santa Road by the sea in Luanda, and sent rice, fuba, mineral water, drinks, pasta, and biscuits, cooking oil, clothes and shoes and other daily supplies to the children, and put on Christmas hats for the children to welcome Christmas together.

In early 2020, when the novel coronavirus broke out in China, the group donated a total of 2 million Yuan and nearly 100,000 masks.

Enterprise Culture

Definition of “Hyway”: Faithful Commitments Forge Great Achievements, Diligent Innovation Contributes to Happy Life.

Management thought: Achieve the goal of long term development with ambition; manage the enterprise according to law and focus on solid work, create global brand and build up a century enterprise.

Hyway spirit: Exploration, innovation, pragmatic and forward-looking view, work actively with high efficiency and embrace responsibility with passion.

Core value: Revitalize the enterprise and be patriotic, benefit society and employees, focus on customer service.

Management philosophy: Scientific and elaborated, diligent and thrifty, enforce with high efficiency and pursue excellence.

Employee ethics: Dedication, devotion, integrity, gratitude; tolerance, appreciation, harmony, friendship and sincerity.

Qualifications

Angola Model Enterprise Certificate

Certificate of Member of China International Contractors Association

Chairmans Words

Cooperation for a better future

——Chairman of China Hyway Group Limited, Liu Daiwen

Take integrity as a foundation and cooperate for a better future. China Hyway Group Limited takes integrity as a foundation, and considers cooperation as a road to harmony and development. With several years of international operation, China Hyway Group Limited has developed a series of unique and high-quality projects for owners at home and abroad.

Currently, China Hyway Group Limited is a comprehensive cross-border group with tremendous strength and multiple industries, engaged in businesses of international engineering, mining, trading and logistics, investment and financing, real estate, tourist, ecological agriculture, etc. The vision of “being a world-famous enterprise” is a new driving force to make us forge ahead with determination.

Now, by developing selected projects, we are willing to sincerely cooperate with friends from home and abroad and persons with lofty ideals from all walks of life for creating a more glorious career through sincere cooperation, far-sighted strategy and steady pace.

164.KPC Pharmaceuticals

INTEGRITY FIRST

KPC Pharmaceuticals, Inc (hereinafter be abbreviated as ‘KPC’, 600422 SH) was established in

March 1951 and floated on the Shanghai Stock Exchange in December 2000. With its considerable professional experience in pharmaceutical industry, the company is listed as one of the new key national high-tech companies and one of the Top 100 companies in the Chinese medical industry. The company’s brands such as Luotai®, Tianxuanqing®, Artemedine®, Artem® and Arco® currently enjoy a good reputation both at home and abroad.

KPC is a pharmaceutical company which focused on innovative natural botanical drugs and integrates R&D, production, marketing, commercial wholesales and international marketing. Its business scope covers traditional Chinese medicine, chemical medicine and medicinal circulation. Companies owned by KPC include the Kunming Traditional Chinese Medicine Co. Ltd., Kingtide Notoginseng Industry Co. Ltd., Xishuangbanna Pharmaceutical Co. Ltd., Kunming Pharmaceutical Commercial Co. Ltd., Kunming Baker Norton Pharmaceutical Co. Ltd., Nukus Botanic Technology Co. Ltd., Kunming Yinnuo Medical Technology Co. Ltd., and Beijing Holley-Cotec Pharmaceuticals Co. Ltd. KPC also boasts a large-scale manufacturing center, Xuesaitong drug research institute and postdoctoral workstation. Situated in the National Biological Industry Base of the Kunming High-tech Zone, the "KPC Bio-Pharmaceutical Science and Technology Park" will be a large-scale, professional and international biomedical park, taking the lead both at home and abroad.

Based on Yunnan’s abundant plant resources, KPC has developed more than 40 new natural botanical drugs, including the artemether series, panax notoginseng series, gastrodine series, special Chinese medicines and special ethnic drugs. With multiple national patents, KPC has filled several gaps in the pharmaceutical industry both at home and abroad. Being highly renowned and popular in terms of treatments for cardio-cerebral-vascular diseases, nervous system diseases and malaria, KPC has made positive and significant contributions to the health of human.

KPC's unique core competitive advantages include its outstanding R&D ability, abundant product resources, strong integrated manufacturing system, internationally compatible quality management system and its international marketing team. In the future, following the principle "From nature; For Human Health", KPC will develop into an international pharmaceutical supplier focusing on cardio-cerebral-vascular disease and chronic disease treatment. At the same time, it will continue striving to be an outstanding and globally famous brand in terms of cardio-cerebral-vascular drugs.

R&D History

In its early days, KPC established Xuesaitong Pharmaceutical Research Institute aimed at utilizing the natural botanical resources of Yunnan to solve the problem of medical shortages in Yunnan. Up to the present day, the institute has over 60 years of drug R&D history and 68 effective patents of invention.

1950-1980

A wide variety of formulations were developed, including urgently needed basic drugs and medicinal materials such as sapolin (an important intermediate of hormone drugs), colchicine and reserpine which reached the international advanced level. This laid the foundation for KPC as a comprehensive pharmaceutical company.

1980-2000

Successful innovated and developed artemether, panax notoginseng and gastrodin products, which reached an internationally advanced level. These three series of products were first put into industrial production by KPC. And later, it began to research and develop ethnic drugs and high-tech preparations including freeze-dried powder for injection, sustained release preparation and controlled release preparation.

The 21st Century

A new stage of development has been entered, with innovative new natural drugs such as Pailike and innovative antimalarial drug ARCO, which is a groundbreaking compound based on natural substances and is the first single dose administration antimalarial drug in the world. In addition, this century has seen the formulation of a development plan targeting the treatment of cardiovascular and cerebrovascular disease and gradual expansion into the field of chronic disease.

R&D Capability

KPC Xuesaitong Pharmaceutical Research Institute aims at taking the road of combing independent research with joint development based on its own strengths and fully take advantage of the abundant natural drug resources of Yunnan. It occupies a leading place in terms of phytoextraction, quality research and preparation research; and it leading the way domestically in terms of research, development and pilot production of semi-synthetic drugs.

◆ National Enterprise Technical Center Awarded by the National Development and Reform Commission, Ministry of Science and Technology, Ministry of Finance, General Administration of Customs and State Administration of Taxation.

◆ National Innovative (Pilot) Enterprise Granted by the Ministry of Science and Technology, State-owned Assets Supervision and Administration Commission, and All-China Federation of Trade Unions.

◆National Model Enterprise for Technology Innovation Affirmed by the Ministry of Industry and Information Technology and the Ministry of Finance.

◆ Laboratory for National Standard Sample Definite Value of Natural Products Granted by specialist team of SAC/TC118 on standard samples of natural product.

◆Postdoctoral Scientific Research Workstation Approved by the National Ministry of Personnel and in cooperation with Yunnan University and the Kunming Institute of Botany of the Chinese Academy of Sciences.

R&D Team

KPC continues to follow a development path based on industry-university-research partnerships and has a professional technical R&D team majoring in medicine and biological sciences, who have made a huge contribution to new product R&D for the company.

Technological Innovation Team of Biological Research for the medicinal product for treating of cerebral apoplexy: leading by Yang Zhaoxiang, who is one of the Innovative Talents of Yunnan and the director of Xuesaitong Pharmaceutical Research Institute, with the team members including top-level talents Doctor Zhang Jianwen, national new drug evaluation experts and senior engineer Shang Jianhua to carry out innovative biological research to natural drugs primarily used for treating of cerebral apoplexy.

Diabegone Innovation Project Team: leading by Chief Scientist Professor Wang Qinghua and Doctor of Biology, Liu Riting, the team is developing a long-acting anti-diabetic GLP-1 fusion protein. As a Chinese Canadian, the team leader Professor Wang Qinghua is a special term professor of Fudan University (endocrinology department of Huashan Hospital), an expert from Thousand Talents Program of the Central Organization Department, Chief Scientist of the JDRF Project, CIHR and CDA, chief PI researcher of the 12th Five-Year Plan major special project on innovative drugs by the Ministry of Science and Technology and a medical and science advisor to Sweden DiamydMedical AB. 。

Biology Research and Evaluation Innovation Team:includes senior researchers Doctor Zhang Jianwen, an American Chinese who is a top-level overseas talent, and Shang Jianhua, who is a national biological evaluation expert for new drugs; their main work includes pharmaceutical biology evaluation and related research work.

Drug Structure Modification Innovation Team: leading by top level talent Doctor Li Jianfeng and doctors from Kunming Institute of Botany of the Chinese Academy of Sciences. Their main work is to do the researches related to structure modification and synthesis of drugs.

R&D innovation team for new preparations:composed of five core scientific and technical personnel from the preparation specialized committee of Chinese Pharmaceutical Association. They mainly engaged in researching of new preparations of drugs and undertaking tasks from other units.

Yunnan R&D Innovation Team for the Artemether series of products: altogether 13 people, of whom six are core members, one with a special allowance granted by the provincial government, four are being groomed as provincial technological innovation talents, two off-station staff, eight senior engineers and four postgraduates.

Technical Innovations (partial list of honors)

In 1996, the research project of oral schistosomiasis prevention drug was recognized as the National 8th Five-Year Plan Major Scientific Achievement for Tackling Scientific Problem.

In 2000, Luotai Project (Panax notoginseng for injection) won The Best Project in National 15-Year Patents Achievements Exhibition;

In 2001, the application and basic research project on preventing schistosomiasis japonica, schistosomiasis mansoni and schistosomiasis haematobia by Artemther was awarded the Second Prize in the 2001 National Scientific and Technological Progress.

In 2001, Luotai Project won the prize of the National 9th Five-Year plan Outstanding New Product of Technological Innovation.

In 2007, compound Artemther R&D, internationalization and industrialization project won the Second Prize in the 2007 National Award for Scientific and Technological Progress.

In 2009, the antimalarial medicine Coartem won the European Inventor Award.

In 2009, the development and international market expansion of the new antimalarial Compound

Naphthoquine Phosphate was awarded the Second Prize of People's Liberation Army Scientific and Technological Progress.

In 2014, several national projects were undertaken, including the “Major New Drug Development” and the major new drug innovation projection “Evaluation of the Safety and Post-Marketing Re-Evaluation of Panax Notoginseng for injection (lyophilization)” of the Ministry of Science and Technology and the State Administration of Traditional Chinese Medicine.

Quality Control

Over the course of its 60-year development, KPC has invested a large amount of money into GMP technical modification for oral dosage forms plant, injection plants and API plants. All production equipment and facilities are strictly designed and installed in accordance with the GMP standards and have passed national and international GMP inspections, this indicates that KPC has advanced technical level as well as a complete production and quality management system. Since 1986, China Food and Drug Administration has implemented spot-checks on the quality of medicines and KPC’s market spot-check qualified rate has continuously remained at 100%. In order to ensure product quality and safety of medicines, quality management uses relevant standards and records to establish a comprehensive quality assurance system for monitoring and management of the entire process.

◆Freeze-dried injection plant, small-volume injection plant and oral dosage plant have passed CFDA GMP inspection;

◆Freeze-dried powder for injection, small-volume injection, API I (Artemether), (API) gastrodin, Acegastrodine and Calcium Levulinate have passed CFDA GMP inspection;

◆No.4 phytochemistry plant has passed GMP inspection of WHO, TGA, and FDA;

Honors

KPC Pharmaceuticals, Inc (hereinafter be abbreviated as ‘KPC’, 600422 SH) was established in March 1951 and floated on the Shanghai Stock Exchange in December 2000. With its considerable professional experience in pharmaceutical industry, the company is listed as one of the new key national high-tech companies and one of the Top 100 companies in the Chinese medical industry. The company’s brands such as Luotai®, Tianxuanqing®, Artemedine®, Artem® and Arco® currently enjoy a good reputation both at home and abroad.

Culture

KPC's unique core competitive advantages include its outstanding R&D ability, abundant product resources, strong integrated manufacturing system, internationally compatible quality management system and its international marketing team. In the future, following the principle "From nature; For Human Health", KPC will develop into an international pharmaceutical supplier

focusing on cardio-cerebral-vascular disease and chronic disease treatment. At the same time, it will continue striving to be an outstanding and globally famous brand in terms of cardio-cerebral-vascular drugs.

Corporate Strategy

Taking the treatment of cardiovascular and cerebrovascular disease as its core field, KPC is gradually developing into an international medicine supplier in the field of chronic disease.

Corporate Vision— From Nature For Human Health

In the future, KPC will be an internationally leading pharmaceutical company with flat organization, resource pooling, goal-oriented business, low-profit operations, innovative employees, streamlined processes and flexible mechanisms. KPC will seek mutual development and create achievements with employees, shareholders, customers as well as society.

INTERNATIONAL

Since obtaining the rights to conduct import and export operations in 1993, KPC has been actively expanding its overseas market. In January 1997, KPC established its first foreign business office in Yangon, Myanmar. Over nearly 20 years' development, KPC has obtained a great deal of experience and a mature team of overseas IP registration for drugs, overseas sales agents, new product listing and promotion personnel.

In China

◆ KPC has an elite sales team of approximate1000 people and 27 marketing offices and branches;

◆ KPC has a marketing network that covers 30 provinces, over 25,000 medical institutions of different levels and over 10,000 drugstores nationwide.

Overseas

◆ KPC has an elite overseas sales team of nearly 100 people;

◆ KPC has a marketing network that covers 47 countries worldwide located in Asia, Europe, Africa, Oceania and South America.

Clinical Application

With its professional R&D system, KPC has always maintained close links with government institutions, medical experts and academic organizations both at home and abroad. With a pioneering attitude and a broad view of the future, it continues to provide the most advanced medicine for people worldwide.

From 2005 to 2014, KPC has conducted clinical trials on new antimalarials and medicines to treat cardio-cerebrovascular diseases in multiple countries (including Nigeria, Sudan, Cote d'Ivoire, Uganda, Benin, Congo-Kinshasa, Tanzania, Papua New Guinea, Indonesia, Myanmar, Cameroon and Vietnam). After seven years of international multi-center clinical research, compound naphthoquine phosphate tablets have now been demonstrated to be capable of curing drug-resistant non-complex malaria with administration of a single dose, which built a milestone in the history of the fight against malaria.

165.Reanda

Reanda is the brand built up by Reanda Certified Public Accountants ("Reanda CPA"), a licensed accounting firm established in Beijing in 1993. Today, Reanda CPA is the top tier firm in China with over 20 offices and a staff force of 2,000 people.

Following the initial formation announcement of the Reanda International Network by Reanda CPA in 2009, Reanda International Investment (Beijing) Company Limited ("RIIB") and its wholly owned subsidiary, Reanda International Network Limited, were established in Beijing and Hong Kong respectively in 2010. The two companies together are the administration headquarters of the Reanda International Network.

To cope with the recommendations from the CICPA to grow in lock step with ASEAN, the Reanda International Network has begun to establish networking activities throughout Southeast Asia. The Reanda International Network was formally launched on 15 September 2010 with the initial 7 member firms from China, Hong Kong, Macau, Japan, Singapore, Malaysia and Cambodia. Reanda International, as the first China branded international accounting network, strives to assist the domestic clients of the member firms doing business internationally, at the same time, provides a springboard to those foreign enterprises who are planning to explore their business ventures in China.

Today's internationally-focused companies rely on the expertise of accountants and advisors who are not only knowledgeable about the regulations and tax systems of their local jurisdiction, but who are also experienced in working nationally and internationally in other jurisdictions. To succeed in a complex global business environment, senior company executives often need to make key decisions quickly. They need to call on professional advisors in diverse locations who can provide a broad range of high quality services. And it is all the more convenient if the full range of audit, tax and consultancy services can be coordinated through the firms in the same network. Reanda International aims to build a network that matches clients with carefully screened, independent, professional firms that are able to meet these exact requirements and other stringent membership criteria. Reanda International commissions its member firms to help prominent and growing companies in every industry to succeed in today's fast-changing legal and financial landscape.

How we do business

Reanda International collaborates independent member firms from overseas countries and regions with the accounting professions in China. Each member firm of Reanda International is a separate and independent legal entity and renders professional services in the territory based on

deep industry knowledge and understanding of local markets.

Reanda International is aimed at co-operation of member firms within the network. Although operating locally in countries and regions around the world, member firms share the common brand name, a common system of quality control and a significant part of professional resources. As a result, we will get you quicker access of professional advisors in diverse locations offering wide range of high quality services.

In today’s hyper-competitive and ever-changing global economy, Reanda International not only avails its member firms of enhancing local branding and international perspective but also synergy of sharing best practices and knowledge in providing excellent client service to companies of all sizes. This keeps us up-to-date with global industry trends as well as identifying new market opportunities.

Service

Audit & Assurance

Tax

Consulting

China Desk

166.SIMI MOBILE

SIMI is an excellent mobile product & service provider. We focus on mobile product design,develop,production and sales.We provide high quality,competitive,perfect experience product and service for global telecom operators and telecommunication companies.

We have deep cooperation relationship with main chipset companies. And we have the ability to provide total solution including software and hardware. Our engineers have more than 10 years design experience, and they lead or take part in some projects for famous international companies for example LG,SHARP,Sonim,ASUS etc..We have excellent project management system and production quality system. We do believe that innovation and high quality make us competitive.

In the future, SIMI will keep innovating continuously. We have the confidence to become a famous telecommunication company in the world.

SIMI Electronics Co., Ltd. is professionally engaged in wireless communication and the field of electronic products research, development, production and marketing, and we also offer our customers total solution -- “One-step shopping”, from Concept planning, ID appearance, Mechanical design, Hardware, Software development, Quality control & management to final mass production.

SIMI Electronics Co.,Ltd. aims on domestic and international market and serves operator customers. We already built an experienced team, included independent R&D and management team. With ISO9001 quality system, we specialize in customized product design, especially in providing extraordinary experience to fulfill the operators’ requirements.

In the future,SIMI will continue adhering to the "Quality,Innovation, Tean, Service" business philosophy, will gradually become the global leader in mobile communication products and service providers.

Simi overall solution

We have excellent communications product development and production capacity

Dedicated to the design, development, production and sale of wireless terminal products for the global telecom operators.Mobile phone brand provide high-quality, competitive products and services perfect experience.

Simi strong research and development

We have Spreadtrum, MTK, professional and technical research and development capabilities of Qualcomm platform

Owned enterprises world-class design and production experience.

Simi Excellent design quality

Our innovative product design and advanced production technology,

We have excellent quality control system and after-sales response mechanism.

Strength

SIMI has an excellent R&D team， team members have more than 10 years R&D experience. They are familiar to Spreatrum,MTK,Qualcomm chipset platforms. Base on these platforms we have feature phone, android smart phone, tough phone, tablet, wireless card, power bank etc. We have the abilities to design and produce following customer’s requirement.

Innovation

Our team design high effective process to increase efficiency and make cost down,and design beautiful ID to raise product value.

Global quality

We have passed ISO 9001 quality system approval. During the cooperation with oversea operators, we strictly follow their request to do field test to find problems under local network and environment. We have strict quality control system and after sales response system to find problems and resolve them at first time which make our product’s quality good and stable.

Culture

Vision

- ☉ Keep innovation
- ☉ design valuable product
- ☉ full of energy
- ☉ be an famous company in the world

Philosophy

- ⊙ Quality
- ⊙ innovation
- ⊙ value

Products
Feature Phone
Simple and durable Long standby time
Smart Phone
Android system
Mainstream configuration
Accessories
Aluminum Matte highlights
Imported chips
PC/PAD
HD screen
Preferred Entertainment
Other
Course

June 2013 Simi electron established.

February 2014 Simi cooperated with African operators in mass production of mobile phones and successfully entered the African operator market.

2015 Simi started to visit the African market and set up a factory in Ethiopia.

2016 The smesei plant began operation, and the strategic cooperation with Ethiopia telecom.

2017 Simi esei achieved a strong second market share by the end of the year, the same year smi Uganda began operations.

2018 Simi began operations in Cameroon and joined the Cameroon higher education network in the same year.

2019 Smyugandan plant is up and running.

167.China Huanqiu Contracting & Engineering Co., Ltd.

China Huanqiu Contracting & Engineering Co., Ltd. (HQC), founded in 1953, and now a wholly-owned subsidiary of China Petroleum Engineering Co., Ltd., is an international engineering company that integrates the entire EPC process, full-value chain and “one-stop” comprehensive services including R&D, consulting, technology licensing, FEED, design & engineering, procurement, construction, equipment manufacturing, commissioning, startup, operation & maintenance, financing, etc. We engage mainly in engineering construction projects in the petroleum refining, petrochemical, natural gas liquefaction/receiving/treatment, new coal chemical, organic chemical, inorganic chemical and pharmaceutical chemical industries.

HQC has the state-issued Engineering Design Comprehensive Class A Qualification Certificate, Project Engineering Consultant Class A Certificate, Environmental Impact Assessment Class A Certificate, Engineering Project Cost Estimate Class A Certificate, Certificate for the Design,

Manufacturing and Maintenance of Special Equipment, Top Class Certificate for General Contracting of Chemical & Petroleum Engineering Construction Projects, Foreign Engineering Projects Contractor Certificate, and other management system conformity certificates such as ISO 9001, ISO 14001, ISO45001 and CNPC HSE certificates as well.

HISTORY

First Stage (1953~1983): Developed into a famous chemical design institute in China in 30 years' time . Founded in March 1953, the first national chemical design institute in New China. Over the 30 years, the design systems, procedures and methods had been reformed to get in alignment with the international ones. A domestically renowned chemical design institute gradually came into being, possessing professionals with all types of expertise, and strong technical capabilities, for chemical industry.

Second Stage (1984~1994): Grown up within 10 years from a design institute into an engineering company famous in China. Year 1984 was when the transformation from a design institute to an engineering company began. In 1992, approved to be the first "institution-to-enterprise" design unit in China, greatly promoting the optimized allocation of such resources as human, technology and assets and the continual reforms of management systems, pushing forward the company's process of internationalization and marketization.

Third Stage (1995~2005): Stepped up to an internationally renowned engineering company from a domestically known. In 1995, won the first overseas EPC project. Over the ten years, had consecutively secured and executed a lot of EPC and PMC projects in the overseas market. International business revenue increased, year by year, to about 70% of HQC's total revenue.

Fourth Stage (2005~2015): On fast track of development, paced up to becoming a world's first-class engineering company. In June 2005, merged into China National Petroleum Corporation (CNPC). Capability to execute large-scale complex EPC/PMC projects substantially improved, together with market competitiveness and technical innovation ability, leading to the year-byyear doubled economic indices. Thus marching on a high-end development road toward the world's fi rstclass engineering company target.

Fifth Stage (2016~~present): Accelerated construction of a world's first-class comprehensive engineering service provider for refining and petrochemical sectors.

China Huanqiu Contracting & Engineering Co. Ltd. was established, following CNPC’s directive to reform and reorganization, based on the former China Huanqiu Contracting & Engineering Corp. and the former CNPC Northeast Refining & Chemical Engineering Co., Ltd, integrating the refining and chemical engineering and construction business to form a new “7+2” enterprise made up of a force of seven engineering companies and two construction companies.

HQC is moving toward the new orientation and new goal of building a world’s first-class, comprehensive engineering service provider for the petroleum refining and petrochemical projects.

Business

In the traditional petrochemical industry, we have strong technological and brand advantages, equipped with technology packages of our own. Technologies we have mastered enable us to have completed the EPC (Domestic) of 14 categories of large-scale petrochemical facilities, e.g. ethylene complex, polymers plant, ethylene glycol unit, fertilizer complex, acetic acid plant, methanol plant, storage facility (including offshore part), LNG terminal, natural gas processing and transmission facility, chemical mining development, large-scale styrene / polystyrene / butadiene / PVC installations, pharmaceutical plant and so on.

We completed the world's largest acrylic unit - Formosa Plastics Group Ningbo NAE project, the S/SE Asia's largest oil storage facility - Sri Lanka's Ceylon Petroleum offshore oil unloading and onshore storage tank facilities, the CSPC 800,000 T/A ethylene project (in collaboration with JGC), the CSPC 200,000 T/A LDPE, 250,000 T/A HDPE and 240,000 T/A PP projects (with TCM), and completed the PetroChina Lanzhou Ethylene Plant - the first time HQC independently completed the basic design and detailed design of a large-scale ethylene plant with purchased PDP.

Also independently completed was the Zhejiang PC 1.4 Million T/A Ethylene Plant which is the largest single train ethylene unit in China. Other large-scale ethylene facilities we accomplished include Dushanzi 1 Million T/A Ethylene Project, Sichuan 800,000 T/A Ethylene Project, Fushun 800,000 T/A Ethylene Project, Daqing 1.2 Million T/A Ethylene Project and etc. Further, HQC undertook the Shenhua Ningxia Coal Group 500,000 T/A PP Project & 1 Million T/A olefins project and Sichuan PC 450,000 T/A PP Project. The Singapore Double Train Ethylene Project made HQC a recognized top mechanical installation contractor in Singapore. Execution of the Saudi Arabia 400,000 T/A High-Density Polyethylene (HDPE) Project opens the way for HQC to operate in Saudi Arabia and the Middle East.

Business Scope

Petrochemical Industry

Oil Refining

Mega Coal

Chemical/Fertilizer Projects

New Energy

Featured Projects
Representative projects include Yunnan PC 13,000,000 T/A Refinery Project (China's biggest single-train refinery so far), PetroChina Sichuan 10,000,000 T/A Refinery Project, PetroChina Guangxi 10,000,000 T/A Refinery Project, PetroChina Dushanzi 10,000,000 T/A Refinery Project, etc.
Quality, Safety, Health, Environment
Project Evaluation

Following the principle of "Independence, Impartiality, Science and Reliability", HQC accepts the entrustment of government and enterprises to evaluate and demonstrate all kinds of preliminary consulting accomplishments such as Project Planning, Project Proposal, Feasibility Study Report, Project Application Report and Fund Application Report. HQC provides customers with scientific evaluation conclusions, advice and suggestions in implementation of the new development philosophy, promoting energy conservation and emissions reduction, optimizing industrial layout, guiding resource allocation, accelerating the transformation of economic growth model and other aspects.
Environmental Impact Assessment
HQC has more than 20 years of experience in environmental impact assessment (referred to as EIA), has obvious technical advantages in professional fields, and has a wealth of environmental impact assessment performance, including super-large coal chemical projects, refining and chemical integration project, as well as petrochemical project approved by national, provincial, and municipal ecological environment authorities. The business scope involves large-scale coal chemical, refining, petrochemical, chemical, regional development, thermal power, municipal administration, long-distance pipelines, petroleum storage and many other fields. The business covers Xinjiang, Inner Mongolia, Ningxia, Shaanxi, Guangdong, Guangxi, Fujian, Shandong, Yunnan, Hainan, Zhejiang and other regions. The company has successively won a number of national and industry consulting awards. It has strong management capabilities for large and complex environmental impact assessment projects, and has rich experience in coordination and management of sea areas, groundwater, monitoring, atmosphere and other professional units with multiple environmental elements.
Post environmental impact assessment means that when the project construction and operation process does not conform to the environmental impact assessment documents that have been approved, the construction unit shall organize the post-environmental impact assessment, take improvement measures, and submit to the previous approval of the environmental impact assessment documents Department and construction project approval department for record.

As the main unit, the company participated in the compilation of "Technical Guidelines for Post of Environmental Impacts assessment of Construction Projects-Petrochemical Industry" and formed a draft for soliciting opinions.
168.Sinosteel MECC

Founded in 1972 and incorporated in Sinosteel Group in 1999, Sinosteel Equipment & Engineering Co., Ltd. (abbr. Sinosteel MECC) is the sole operational asset wholly owned by Sinosteel Engineering & Technology Co., Ltd. (stock code: 000928), focusing on industrial

engineering & service, municipal engineering and investment, energy saving and environment protection, as well as high-tech businesses.

Sinosteel MECC and its affiliated companies are granted with class-A qualifications for metallurgy and construction engineering design, steel and construction engineering consulting, environmental engineering design, ecology construction and environmental engineering consulting, construction and municipal public engineering general contracting, class-B qualification for highway engineering general contracting and qualifications for special equipment design, equipment integration, equipment supervision, overseas contracting, foreign trade, environmental pollution treatment facility operation, certified by quality, occupational health and safety and environmental systems and running National Engineering Research Centers for Environmental Protection & Industrial Fume Control and for Industrial Fume Dedusting.

LEADERSHIP

As a well-known engineering company in China, Sinosteel MECC has made outstanding contributions to the development of Chinese metallurgical industry by accomplishing over 500 national key projects for giant steel producers. Being one of the first “go global” companies in China, Sinosteel MECC enjoys high reputation in overseas metallurgical engineering market and has set up a more complete business network in more than 50 countries. Among those records of “ largest projects exported by Chinese companies" made by Sinosteel MECC, the TOSYALI 950mm Hot Strip Mill, ISDEMIR No.4 Blast Furnace, JSPL 1.2mtpa Pelletizing Plant and ICDAS 2x600MW Coal-fired Power Plant Projects were awarded respectively National Quality Project Award of the year.

Sinosteel MECC has ranked on top of “China’s Top 100 General Contractors by Turnover” since it was first listed in 2004. Other than being one of the first AAA credit enterprises for overseas contracting, the company was also rated AAA for machinery and electrical products import & export (large complete equipments), AAA for foreign trade and AAAAA for international operation. For several consecutive years, Sinosteel MECC has ranked in the ENR Top 250 International Contractors and Top 250 Global Contractors.

BUSINESSES

INDUSTRIAL ENGINEERING & SERVICE

ENERGY SAVING & INVIRONMENT PROTECTION

SAFETY & PROTECTION

HIGH-TECH

Proficient at steel complex general contracting, Sinosteel MECC has also achieved breakthroughs in mining, power, coal chemical, energy saving and environmental protection fields under the guidance of “internationalization and diversification” development strategy. Meanwhile, a greater focus on emerging industries has been given. By establishing Tianyu Intelligent Manufacturing Co., Ltd. as a joint venture, Sinosteel MECC and Huazhong University of Science and Technology have engaged in cooperation in metal additive manufacturing field, to develop

high-end parts and tool and mould manufacturing business in aerospace, high speed rail and nuclear power equipment areas with application of the most advanced 3D technology. To accelerate market exploring in energy saving and environment protection, Sinosteel MECC actively promoted the marketization and industrialization of advanced technology of its holding company - Sinosteel Tiancheng Environmental Protection Science & Technology Co., Ltd., and anticipated market opportunities of coal mine methane and carbon asset management. Given the opportunities of investment and financing system reform and increasing customer demands in infrastruction , Sinsoteel MECC has developed municipal works and investment business aiming at PPP and urban rail transit in affiliation with financial institutions.

INTERNATIONALIZATION & DEVERSIFICATION

Focusing on five core capacities building of international contractors, Sinosteel MECC will constantly improve its sustainable development ability and quality in four main business sectors with the guideline of " internationalization and diversification " and gradually turn into diversified business mode of investment, construction and operation, with the purpose of providing better service and creating more value to our customers.

AWARDS

National Project Construction Quality Award SilverPrize for Tosyali 950mm Hot Strip Rolling Mill,Turkey

Most Growing Value Award in Listed Company Reputation Ranking

Metallurgical Quality Project Award for KhazakstanSintering Machine Flue Gas Dedusting Project

INDUSTRIAL ENGINEERING AND SERVICE

As an industry-leading engineering company, we started our exploration of overseas market since 1990s and have established a solid business network in more than 50 countries so far. In new century, we developed diversified business based on traditional advantage, so as to provide our customers in mining, metallurgical, power and coal chemistry industries with overall contracting and support service.

HEALTH, SAFETY & PROTECTION

Sinosteel Wuhan Safety & Environmental Protection Research Institute Co., Ltd (Sinosteel SEPRI) provides professional HSE consulting services & solutions and helps partners reach the goal of an accident-free workplace. With rich experience in risk management & control, the company expands its business into the area of green and decarbonization development. Excels in basic research and best-in-class know-how, Sinosteel SEPRI can tailor-make roadmap and strategies for customers to cope with CO2emission controlling targets and climate change.

- Business covers health & safety, environmental protection and circular economy, excelling in basic research and technology and accumulating a mount of industrial applications;

- Come to the fore in terms of risk management & control, as well as controlled blasting technology;

- Provide solutions for Hazard & Risk Identification, Assessment and Prevention; Advanced

facilities and techniques for Personal Protective Equipment (PPE) Testing, Occupational Health Testing & Screening, Safety Evaluation & Training and Risk Analysis of Dust Explosion; World-class technology of controlled blasting;

● Have great R & D achievements in recycling of steel slag and Tunable Diode Laser Absorption Spectroscopy (TDLAS) technologies.

ENVIRONMENTAL PROTECTION

Sinosteel Tiancheng Environmental Protection & Technology Co., Ltd (Sinosteel Tiancheng), a subsidiary of Sinosteel E& T, is a front runner in developing green solutions for iron & steel makers. Boasting of two national technology centers and one workstation for academicians of the Chinese Academy, Sinosteel Tiancheng has built a great number of model projects by applying its pillar tech on ultra-low emissions. With its dedication to technology innovations, Sinosteel Tiancheng will help customers realize the synergy between pollution and carbon emissions control.

● Ranked as a front runner in developing green solutions covering dedusting, dust source control, as well as desulfurization and denitration for iron & steel makers:

1. Pre-charged high-efficiency PM2.5 control technology; high-efficiency and low- resistance bag filter; Wet Electro Static Precipitators (WESP);

2. Solar film sealing technology; intelligent mobile ventilation slot dust collection technology; water-sealed tank plus ground de-dusting station;

3. Synergistic ultra-low emission treatment for SO2 and NOx from sintering, pelletizing and coking process;

● Independently developed Fluid Catalytic Cracking(FCC) flue gas filtration technology, a breakthrough in the field;

● Two national technology centers and one workstation for academicians of the Chinese Academy of Engineering, endeavoring to lower emissions, minimize energy consumption and maximize environmental responsibility;

● Completed eight national research programs and being granted over eighty patents by

collaborating with renowned institutions & organizations;
HIGH-TECH
INTELLIGENT MANUFACTURING

Originally initiated and invested by Sinosteel MECC, Tianyu Intelligent Manufacturing is a high-tech company mainly involved in sectors of 3D printing, metal parts restore and remanufacturing and industrial intelligent system, integrating manufacturing, equipment research and development, as well as technical service. The R&D team consists of leaders from international prestigious university, experts of concerned field and high-educated youth talents, aiming aggressively to be the industry pioneer in high-end intelligent manufacturing equipment and technical service.

l Customized high-tech service

3D printing technology

Cladding and re-fabrication

Intelligent manufacturing equipments

Arc/laser micro casting-forging-milling composite addictive manufacturing equipment

Laser cladding and remanufacturing equipment

Intelligent arc/laser welding robot

Automated stereoscopic warehouse logistics system

Technical advantages

High performance

High efficiency

High competitiveness

Innovative and revolutionary

Applications

169.Hangzhou Water Treatment Technology Development Center

Affiliated with China National BlueStar (Group) Co Ltd, Hangzhou Water Treatment Technology Development Center is the supporting unit of National Engineering and Technological Research Center for Liquid Separation Membrane and a base for the national membrane sector. Established in 1984, the center was previously a scientific institute under the State Oceanic Administration. In 2006, it joined China National BlueStar (Group) Co Ltd and specialized in membrane-based water treatment technology, product development, engineering design, production and system integration. Covering an area of 2.67 hectares with registered capital of 95 million yuan ($15 million), it has nearly 330 employees, among whom 70 percent are technical experts. The center boasts of one academician, 10 experts that enjoy special governmental allowances, 48 senior technical staffs and 55 intermediate technical staff members. It is one of the earliest domestic membrane R&D institutions and a leader of the liquid separation membrane sector.

It insists on safeguarding its leading position through innovations. Since the 6th Five-Year Plan, the center has undertaken nearly 100 projects, such as the nation's "863" and "973" projects, as well as major technical projects in Zhejiang, which has won awards such as first and third prizes of National Prize for Progress in Science and Technology, three prizes for tackling problems in science and technology, nearly 40 prizes at ministerial and provincial levels and obtained over 80 national patents. Overall technology is among the most advanced, reaching global advanced levels. The center is the backing unit of Chinese Seawater Desalination and Water Reuse Society and Zhejiang Province Membrane Society.

For decades, as a base for the national membrane sector adheres to the philosophy of "Full Solution" and has made achievements in sea water and brackish water desalinization, waste water and reclaimed water reuse, industrial water and municipal water treatment, pure water and ultrapure water preparation, chemical separation, concentration and purification sector. With its scale ranking foremost in the industry, the center has undertaken the design and construction work for over 400medium and large-sized water-treatment projects, offered more than 500 units of equipment to the market, and exported products to over 10 countries and regions in Southeast Asia and Middle East.

It is endowed with engineering design certificates, Grade A and Grade B consulting certificates and general contract certificates, as well as nearly 40 national registered engineers.

The Center sets, under jurisdiction of it, Hangzhou (Torch) Membrane Industries Co., Ltd. and Hangzhou Beidouxing Membrane Products Co., Ltd.

Hangzhou (Torch) Membrane Industries Co., Ltd. was founded in 1996, and as a corporation wholly owned by the Center, it integrates sci-tech development, production, business together, and mainly operates development, manufacture, sale and services of membrane separation techniques, separation membrane, water treatment engineering and technical products.

Hangzhou Beidouxing Membrane Products Co., Ltd. was founded in 2002. It is a hi-tech enterprise which was incorporated by the Center and is engaged specially in development, production and popularization of spiral wound and hollow fiber membrane elements.

Qualifications and Honors

General Contract Certificate for Environment Pollution Disposal in Zhejiang Province

Class A Enterprise for Utilization of Seawater

Environmental Protection Product Certification for Power Industry Reverse Osmosis Water Treatment Fa

Top Ten Units of China Association of Oceanic Engineering

Excellent Team for Outstanding Execution of the National S&T Plan during the 11th Five-year Plan

Industrial Status

As the supporting institute of the National Liquid Membrane Separation Engineering & Technology Research Center, Hangzhou Water Treatment Technology Development Center is affiliated with the China Society of Sea Water Desalination and Water Reutilization, National Water Purification Technology and Equipment Center and Zhejiang Membrane Society and distributor of the magazine, Water Treatment Technology.

The center is the base for research and development, achievements and production of separation membrane as well as for domestic and overseas academic exchanges.

It boasts of the best professionals, including academicians of the China Academy of Engineering and six experts who enjoy special government subsidies. The center can confer master degrees.

Party and national leaders such as Li Ruihuan, Huang Ju, Zou Jiahua, Chi Haotian, Jiang Zhenghua and Song Jian visited the center respectively. Former President Jiang Zemin observed its project.

Zou Jiahua and Chi Haotian wrote, “develop water technology to benefit whole society”, which demonstrated the mission and responsibility of the center.

In the past two decades, the center has grown from a research office with less than 3 million yuan ($474,846.14) assets to a leader in the membrane separation sector.

President Hu Jintao presented the reverse osmosis seawater desalination equipment as a gift to the Republic of Kiribati.

The center is the leader in R&D of membrane technology and enjoys rich experiences and great strength in product development and applications.

Since the Seventh Five-Year Plan (1986-90), it has completed nearly 100 key projects in the

Seventh, Eighth, Ninth, Tenth and Eleventh Five-Year Plan, the 973 Plan, 863 Plan, National Key Technologies R&D Program and the scientific and technological plans of National Natural Science Foundation of China, State Oceanic Administration and Zhejiang provincial government.

It has won first and third prize of the National Prize for Progress in S&T, three awards of the National Prize for Significant Achievements in Science and Technology R&D and over 40 provincial and ministerial prizes for progress in S&T.

It has developed new products and received more than 50 patents at the national level. It has undertaken the project for Key Technologies and Equipment Research of 10,000-ton Seawater Desalination through Membrane Technologies sponsored by the Ministry of Science & Technology.

The achievements end the long-term reliance on importing membrane technologies and products.

Membrane technology has become a typical clean technology and backbone of industries, that include concentration and extraction of special materials, desalinating seawater, purifying industrial water, boiler feed water and waste water recycling.

The center drafted and wrote more than 30 industry and national standards as well as standards and education guidelines for the National Membrane Technology Project.

It held over 20 international academic conferences and 30 training classes, which trained more than 3,000 people.

It has participated in drafting national membrane technology and industry development plans and provided consulting services in key projects.

Meanwhile, as a representative of the China membrane separation enterprise, the center participated in international membrane technology conferences.

The center has become one of the biggest companies in the membrane sector.

It has completed projects for chemical pure water, seawater desalination, boiler feed water, process water, medicinal water, ultra-pure water for electrical industry, concentration and separation of metal compounds and other industrial fields.

The 500 t/d output reverse osmosis seawater desalination project won gold prize in survey and design awarded by the State Oceanic Administration.

It participated in the construction of 1,000 ton and 10,000 ton reverse osmosis seawater desalination model projects, the 100,000 t/d project in Zhoushan, and 50,000 t/d project in Caofeidian.

The Caofeidian project was built by Chinese companies as the general contractor and model project of the National Development and Reform Committee.
Corporate Culture
Our Vision: To build an enterprise with cutting-edge technology and competitiveness in the international market as an industry leader.

Our Mission: To develop water technology and bring benefits to society.

Our Aim: To satisfy clients with creation, seek developments with innovations and realize life values with business.

Our Values: Work hard to develop the company and industry to contribute to industrial prosperity and motherland’s developments to achieve mutual developments and self-fulfillment.

Our Spirit: United, pragmatic, dedicated to work and innovative

Our Management Concepts:

Strategic Management Concepts: Be mindful of potential dangers

Organizational Management Concept: Focus on efficiency

Human Resources Management Concept: respect every employee

Marketing Management Concept: Try to seize every possible opportunity

Financial Management Concept: Be meticulous in planning and strict in budget

Business Management Concept: Fully devote oneself to work

Service Management Concept: Be committed to integrity

Safety Management Concept: Take responsibility at every moment

Code of Conduct for Employees:

To the Center: Be faithful; share honor or disgrace with it;

To Work: Be devoted to duties and work conscientiously

To Clients: Be enthusiastic and honest

To Colleagues: Respect each other, be united and work together

To Superiors: Respect leaders and comply with management

To Subordinates: Respect and care for them and maintain effective communication

To Oneself: Be confident and self-disciplined and strive to become stronger

Our Trademark:
H-Hangzhou

WT-Water Treatment

T—Technology

Blue stands for the color of water. And the center is engaged in water treatment, which is desalinating seawater and transforming sewage into resources that are closely linked to water.

Green represents energy savings and environmental protections. The center has striven to save energy while desalinate seawater, and its technology of transforming sewage into resources have contributed to environmental protections.

The horizontal stroke that stretches across H like a wave symbolizes a steady flow of water for human beings and time-honored history of the center’s water treatment business.

Cares

In 1985, former premier Li Peng inspected scientific research achievements of the center.

In November 1998, Zou Jiahua, former vice-chairman of the Standing Committee of the National People’s Congress, visited the center.

On July 13, 1997, Jiang Zemin, former Party general secretary of the CPC Central Committee, inspected water supply equipment for Zhongnanhai.

In November 1998, former member of the Political Bureau of the CPC and the Party Secretary of Shanghai City, Huang Ju, and former mayor of Shanghai City, Xu Kuangdi, visited the Water Treatment Center.

In April 1999, Jiang Zhenghua, former vice-chairman of the Standing Committee of the National People’s Congress, visited the center.

Innovation

Scientific Research Team

The center has 300 employees and 70 percent of them are professional technicians and nearly 100 are senior technicians. It has a leading technology team, which is composed of an academic from the Chinese Academy of Engineering, nine experts who enjoy special government allowances, experts in engineering and technology leader, formed an international leading level of technology innovation team, undertaken and completed more than 50 national and provincial major scientific and technological innovation project. The team was listed in the first batch of key construction technology innovation team in Zhejiang province, and was awarded the "11th five-year national science and technology plan implementation outstanding team" by the

Ministry of Science and Technology.
Research & Development
The center has the most comprehensive membrane manufacturing technologies in China and can produce a full range of membrane products including ultra-filtration membrane, spiral composite reverse osmosis membrane, spiral composite nano-filtration membrane.
Achievements

170.ENERGY CHINA
China Energy Engineering Group Co., Ltd.(ENERGY CHINA), is a Fortune Global 500 company, ranking 3rd in ENR Top 150 Global Engineering Design Firms and 13th in ENR Top 250 Global Contractors.

As a comprehensive conglomerate that provides holistic solutions and full-chain services in areas such as power and infrastructure in China and the world at large, Energy China’s main business scope covers energy and power, water conservancy and water affairs, railways and highways, ports and navigation channels, municipal engineering, urban rail, eco-environment protection and housing construction, with a complete industrial chain integrating planning and consulting, evaluation and review, survey and design, construction and contracting and management, operating maintenance and investment operation, technical services, equipment manufacturing and building materials.

In 2021, under the guidance of “1466” Strategy, Energy China strives to build a "Main Body, Two Wings" overseas priority development system, and an integral “Main Body” platform embodied by an entity name of China Energy International Group Co., Ltd that leads international business development, continuously providing high-quality business services for global customers， contributing Chinese wisdom and Chinese solutions to the building of a community with a shared future for mankind.
Message From Chairman
Rooted in the soil of China's electric power industry, Energy China has thrived thanks to the care and support from all walks of life, and hereby I would like to extend my sincerest gratitude.

Energy China adheres to the principle of green, circular and low-carbon development, the concept of “Bringing Energy to the World” and the vision of becoming a “world-class industrial leader” with international competitiveness. We have made strenuous efforts to strengthen our core competence in power engineering industry, to become a bellwether in China’s energy engineering field, and to bring world-class projects to both China and the world.

Energy China is willing to sincerely cooperate with domestic and foreign friends to pursue common development, share achievements and jointly create a bright future for all!

—— Song Hailiang, Chairman of CEEC
Business
First-class Energy Integration Solutions Provider
First-class General Contractor

First-class Infrastructure Investor
First-class Integrated Ecosystem Restoration Solution Provider
First-class Integrated Urban Development Operator
First-class Provider of Building Materials, Industrial Products and Equipment
Corporate Culture
Energy China vigorously develops its unique corporate culture for fundamental purpose of achievement growth of the enterprise and its employees in an all-round way.
Mission
Bringing Energy to the World
Vision
World-class Industrial Top leader
Core Values
Ever-improving professional competence and operational capacity
Corporate Spirit
To achieve harmonious collaboration and solid development
Goals
To establish a legacy of refinement and quality innovation
Business Culture
To create an inclusive workplace of viability and efficiency
Management Ideology
To execute integrity operation and quality productivity
With people-oriented management as the core concept, corporate culture development as the means to form synergy, and brand building as a spirit for inspiration, the company is transforming itself from scientific management to cultural management.

171.CNCEC
China National Chemical Engineering Group Corporation Ltd. (abbreviated as CNCEC) is a large-scale engineering corporation directly supervised by State-owned Assets Supervision and Administration Commission of the State Council of China. CNCEC is an international engineering enterprise with the most comprehensive qualifications, complete functions and integrated business chains. As the technology intensive and national demonstrative enterprise for intellectual property, CNCEC is not only the founder of China's petroleum and chemical industrial system, but also the provider of advanced industrial comprehensive solutions, the pioneer of China's construction engineering system and mechanism reform. Furthermore, CNCEC is the vanguard of the "Belt and Road" Initiative, the leader in the field of clean energy engineering, and the construction practitioner of Beautiful China Initiative.

CNCEC's root could extend back to 1953, the establishment of Design & Construction Companies under former Ministry of Heavy Industry. Since then, more than 90% of China's chemical engineering projects and over 50% of China's oil refining and petrochemical projects were executed by CNCEC. In the 1970s, by the completion of 4 sets of fiber units and 13 sets of fertilizer units in which foreign investment was adopted, CNCEC made outstanding contribution to solving the "eating and dressing" problems of Chinese people at that time. In 1984, CNCEC registered in the Bureau of Industry & Commerce Administration under the name of China

National Chemical Engineering General Company and was renamed as China National Chemical Engineering Group Corporation in 2005. In September 2008, China National Chemical Engineering Co. Ltd. was established by China National Chemical Engineering Group Corporation, together with Shenhua Group and Sinochem Corporation. On January 7th, 2010, CNCEC was successfully listed on Shanghai Stock Exchange. In December 2017, CNCEC completed the reform of modern corporate structure system and changed its name to China National Chemical Engineering Group Corporation Ltd.

China National Chemical Engineering International Corporation Ltd. (abbreviated as CNCEC International) is the core subsidiary company and overseas business platform of CNCEC. CNCEC international is authorized to conduct international business in the name of CNCEC. Its business scope includes chemical, petrochemical, coal chemical, nature gas chemical, fine chemical, environment protection, power generation, municipal engineering and infrastructure works. It has comprehensive competence to provide services ranging from planning, consultation, design, procurement, construction, commissioning and maintenance to project financing. Meanwhile, it also engages in project investment and merger & acquisition.

Development Strategies

As a pioneer participant in fulfilling the Belt & Road Initiative, CNCEC always bears the mission to promote development of the overseas business and expand the brand influence. CNCEC is committed to becoming a provider of integrated solutions in industrial sector and a supplier of a high-end chemicals and advanced material and realizing the goal of being a world-class engineering company with distinctive features, leading technologies and strong core competitiveness.

Business

Integrated Engineering Services

【Planning & Consultation】Provide overall solutions and management & consulting services for industry development, and project decision-making, including but not limited to: planning preparation and consultation for overall, special item, regional, industry and park Consulting Services for FEL/FEED/BEP, Plant Site Selection and Evaluation, Project Application Report, Project Feasibility Study Report, project financing, etc.

【Survey & Engineering】CNCEC owns 6 subsidiaries with Comprehensive First-Class Qualification in Engineering Design and 3 subsidiaries with Comprehensive First-Class Qualification in Engineering Survey. CNCEC has accomplished more than 200,000 projects in different sectors and fields.

【EPC】CNCEC is the first engineering company that adopts the EPC model to execute large and medium scale industrial plants in China. It has 40 years of general contracting experience and can provide “turnkey” services of engineering, procurement, construction and commissioning. CNCEC possesses 4 Extra-Grade Qualifications in Construction General Contract, over 40 First-Class Qualification in Construction General Contract and over 60 First-Class Qualifications in Construction Contract. With more than 30,000 technical experts working for CNCEC, CNCEC has completed over 90,000 high quality projects both in domestic and overseas markets. CNCEC’s strong management capacity, technical level and advanced machinery equipment make CNCEC staying in a dominated position in domestic market.

【Operation & Maintenance】In addition to a large number of domestic and overseas EPC projects, CNCEC has also undertaken many overseas BOT and BOOT projects and invested several industrial projects, accumulated rich experience in plants operation and maintenance.
【Project Management】CNCEC is capable of skillfully applying PMC, EPC, EPCM, BOT, CM and adopting prevailing international project management standards and procedures to provide optimized project management services to the owners with excellent management and control of safety, environmental protection, quality, progress and cost.
【Financing Services】CNCEC is one of the first batch companies in China to implement investment and financing of projects on basis of BLT, BOT, BOOT etc. The development of company has been supported by the growth of global financing business with professional financial and low financial cost. It effectively plays the important role of finance and capital and realizes effective coordination between industry and finance.
Business Map

【Asia】: Bangladesh, Cambodia, India, Indonesia, Iraq, Japan, Kazakhstan, Korea, Kuwait, Laos, Malaysia, Mongolia, Myanmar, Oman, Pakistan, Qatar, Saudi Arabia, Singapore, Sri Lanka, Thailand, Turkmenistan, Uzbekistan, UAE, Vietnam, etc.

【Europe】: Albania, Belgium, Britain, France, Germany, Italy, Russia, Turkey, etc.

【Africa】: Angola, Egypt, Ethiopia, Mozambique, Nigeria, Sudan, Tanzania, Tunisia, etc.

【North America】: Canada, USA, etc.

【South America】: Argentina, Brazil, Chile, Cuba, etc.

【Oceania】: Australia, New Zealand
Project
Refinery
Petrochemical
Natural Gas Chemical
Coal Chemical
lnorganic Chemical
Terminal
Power Plant
Renewable Energy
Environmental Protection
Infrastructure
Others
Investment Operation
172.Power China Sichuan Electronic Power Engineering Co., LTD
Power China Sichuan Electronic Power Engineering Co., LTD is subordinate to the Power Construction Corporation of China (Power China), a member of Fortune 500, and owns

operations in over 116 countries and regions around the globe.

With more than 30 years of industrial accumulation, we have developed into an engineering company integrating planning, construction, operation and maintenance and financing services of power engineering. We own a number of class-A qualifications for power industry prospecting, design, consulting and general contracting as issued by the Chinese government, have the import and export qualification for contracting overseas projects, and have accredited the quality, environment, occupational health and safety certifications.

We have more than 600 engineering and project management experts. In addition to the Chinese market, we have entered more than 20 foreign countries covering regions of Eastern Europe, South Asia and Africa, providing quality services to dozens of internationally renowned energy companies.

We have rich experience in engineering design, mature project management skills, reliable professional and technical strength, strong business development capabilities, and efficient resource integration capabilities. We provide global customers with integrated, full-industrial-chain and comprehensive construction services in the field of power engineering and infrastructure.

Management System

Aiming at constantly improving services quality, fulfilling our commitment towards social and staff care, we have maintained GB/T 19001 idt ISO 9001 certification since 1996, GB/T 24001 idt ISO 14001 and GB/T 28001 idt ISO 18001 certifications since 2006.

Business Fields

Engineering survey and design; EPC; project management; project consultation; project supervision; environmental impact assessment; preparation of soil and water conservation plans for development and construction projects; contracting overseas electric power projects and domestic international tendering projects, the survey, consultation, design and supervision works in such overseas projects and the export of equipment and materials required for such overseas projects, and dispatching laborers required for implementing such overseas projects; project tendering agency; and wholesale and retail.

Services

Investment and financing services:

we join hands with famous financial institutions to build up strong resources network, so as to provide financing and credit supports for potential projects, offer complete business and financial solutions, and research and realize investment opportunities for our customers.

Engineering construction-related overall-process services:

we are able to provide design.procurement, construction and operation services as well as

derivative related project management services throughout the project process, and offer differentiated engineering construction solutions based on customers’ individual demands, in order to truly realize “customization and satisfy all customers’ demands.

Planning and consultation services:

we are always willing to conduct deconstruction from the perspective of customers, and provide various earlier services such as power energy planning, studies on project feasibility and project investment opportunity, and project investment & financing preparation and appraisal.

PROJECT PERFORMANCE

We deliver life-cycle management services for power projects, including development, design, construction, operation, and maintenance. Total contract value been undertaken has reached 26.3 billion Yuan, among which power generation projects worth15.2 billion Yuan; power grid projects worth 7.3 billion yuan; new energy projects worth 3.8 billion Yuan. Our company has been listed among China’s top 100 EPC contractors in survey and design industry for 14 consecutive times.

Planning and Consulting

In the past ten years, we have led or participated in more than 40 domestic and foreign power grid planning and wind power base planning. We have also participated in a number of UHV grid feasibility studies, completing more than 500 power plant access system study and grid feasibility studies, and completed more than 670 cases of communication projects.

Survey and Design

We have advanced survey and design technology and unique advantages in the field of circulating fluidized bed, gas-steam combined cycle power generation, distributed energy, etc. We provide grid survey and design at all voltage levels from 10 kV to 1100 kV. We have industrial edging capability in UHV grid design. Our design of transmission line at high altitude, in heavy icing area, and steep mountain is among the best in the industry. We also have leading technology in China for long span transmission design, smart grid design and aerial survey.

International Businesses

We are deeply expanding our international business. We have completed 31 surveys and design consultations in India, Côte d’Ivoire, Kenya, and other countries and have undertaken contract value of 11 billion Yuan, demonstrating our excellent contractor brand.

Other Businesses

We keep making new attempts in structural adjustments and development method. We have achieved steady development by pioneering awareness, sufficient talents, and technical advantages.

EXPERTISE

Competitive Edge

We have rich experience in general project contracting and project management. We are technically leading in UHV power transmission, smart power grid, survey and design in geologically and geomorphologically complex area high-altitude area, heavy-ice area and steep mountain area. We have unique experience in flue gas desulfurization and denitration, circulating fluidized bed power station and design of PV power stations in topographically complex mountainous areas. We rank first industrially and domestically in relevant fields. Now we have 15 invention patents, 100 utility model patents, and 16 proprietary technologies in power engineering design.

Scientific and Technological Strength
We are national high-tech enterprise and enterprise technology center in Sichuan Province. We build our competitive edge in the market with technological innovation. We are able to provide a one-stop solution for systematic and customized energy projects.

Patent Proprietary Technology (Part)
Industry leader (Part)
Scientific and Technological Progress Award (Part)
We wish to work with our partners for mutual benefit and win-win results to create a better future.
SOCIAL RESPONSIBILITY
We shoulder the science and technology, experience and responsibility to help more and more countries, cities and regions to meet development challenges. We build solid foundation for happiness for people's livelihood, deliver light to remote areas, and provide guarantee for the prosperity in cities.
173.China ENFI
The China ENFI Engineering Co., Ltd. and China ENFI Engineering Corporation ("China ENFI"), formerly known as the China Nonferrous Engineering and Research Institute, i.e. the former Beijing Central Engineering Institute for Nonferrous Metallurgical Industries, was established in 1953. China ENFI is now a subsidiary of one of the world's top 500 enterprises - China Metallurgical Group Corporation.

Through more than sixty years of trials and hardships, China ENFI has developed into an international engineering company and formed three major businesses: project integration, new energy industry and resource development.

—— Project integration

EPC services, engineering consulting and design, scientific research, supervision, equipment development & supply, information and automatic control systems integration, as well as upstream and downstream businesses derived from projects, etc. in the fields of mining engineering, nonferrous metallurgical engineering, electrical engineering and automation, municipal engineering and architecture, chemical and environmental engineering, energy and environmental engineering, etc.

—— New energy industry

The photovoltaic materials industry and photovoltaic power generation industry based on polysilicon production;

—— Resources development

Waste incineration for power generation; water resource development; mineral resources development; real estate business.

China ENFI is considered as:

A Well-known Brand in the International Nonferrous Engineering Circle;

A Leader in Technological Progress in the Nonferrous Metals Industry in China;

A Pioneer in National Polysilicon Industry;

An Outstanding Enterprise in the New Energy Industry;

And an Explorer in Resources Development.
Address of the Chairman
Welcome to the ENFI site! Thank you for your attention!

ENFI, short for Beijing Central Engineering Institute for Nonferrous Metallurgical Industries, is well known in the international nonferrous engineering circle.

ENFI grew out of China Nonferrous Engineering and Research Institute and China ENFI Engineering Co., Ltd. The two were the epitome of ENFI keeping abreast of the times, and reflected its features at different times.

ENFI became a subsidiary of the China Metallurgical Group Corporation in 2005.In 2006, China ENFI Engineering Corporation was founded, marking ENFI’s strategic transformation into an international engineering company, and from then on, ENFI embarked on a new stage of development.

ENFI, with a glorious past, becomes stronger with the development of China's nonferrous metals industry. We have spent more than sixty years on a journey replete with stories. This is what we are proud of.

ENFI boasts profound culture that has bred the spirit of seeking truth from facts, an open and inclusive mind, a noble sense of devotion, and an innovative concept of advancing with the times. This is who we are.

ENFI envisions a more splendid future, for we uphold the scientific outlook on development, focus on people, and respect employees, with a view to developing the company in a scientific manner, building harmonious relations between departments, and rendering our employees happiness. This is what we believe.

“Laying a solid foundation, focusing on innovation and entering global market” is the blueprint of ENFI. ENFI is advancing towards a brilliant future on the journey of striving to be the most trustworthy international company specialized in engineering services and resource & energy development!

We hope that you can learn more about ENFI through our website www.enfi.com.cn, a bridge for our communication and cooperation. We are willing to join hands with you and develop together.

Lu Zhifang, Chairman of China ENFI Engineering Corporation
Corporate Culture
The logo consists of letters “ENFI” and the corporation name in Chinese. “ENFI”, short for the Central Engineering Institute for Non-ferrous Metallurgical Industries, represents historical inheritance of the corporation. “E” stands for Engineering, “NF” for Non-ferrous and “I” for “Institute”.

The logo is composed of straight but flexible lines and the ratio between curves and straight lines demonstrates uniqueness of the brand. Letters are positioned like a rational and harmonious formula, which uniquely synchronizes characteristics of nonferrous industry into stability and inventiveness. The streamline design shows the image of ENFI’s responsive, cooperative, progressive, active and energetic style. Meanwhile, the cool color of blue conveys the message of firmness, pragmatism and eternity, and with the combination of frameless design, it symbolizes knowledgeableness, openness and inclusiveness. The whole design, simple but meaningful, distinguishes ENFI’s corporate identity with being technology-oriented and effective.

Cultural Philosophy
Vision

To be the most trustworthy international company committed to engineering services and resource & energy development

Mission

Turn a stone of resource into a gem of undertaking.

Core Value

People-oriented, Excellent, Moral, Shareable, Open & Inclusive, and Effective & Profitable

Corporate Style

Responsive, Committed, Realistic & Pragmatic, and Cooperative & Progressive

Corporate Spirit

Dedicated, Loyal, United and Enterprising

Management Philosophy

Uphold Corporate Integrity, and Take Pride in Customer Satisfaction.

Culture of the Parent Company – Culture of China Metallurgical Group Corporation (MCC)

MCC Cultural Philosophy
Overall Strategy

Make Innovation and Improvement, Become Stronger and Bigger, Scientific Development, and Lasting Property and Peace

Overall Objective

To build a competitive world-leading enterprise group

Management Philosophy

Uphold Corporate Integrity, and Take Pride in Customer Satisfaction.

Corporate Spirit

Dedicated, Loyal, United and Enterprising

Overall Strategy

Make Innovation and Improvement, Become Stronger and Bigger, Scientific Development, and Lasting Property and Peace

Overall Objective

To build a competitive world-leading enterprise group

Management Philosophy

Uphold Corporate Integrity, and Take Pride in Customer Satisfaction.

Corporate Spirit

Dedicated, Loyal, United and Enterprising
Vision

To be the most trustworthy international company committed to engineering services and resource & energy development

Mission

Turn a stone of resource into a gem of undertaking.

Core Value

People-oriented, Excellent, Moral, Shareable, Open & Inclusive, and Effective & Profitable

Corporate Style

Responsive, Committed, Realistic & Pragmatic, and Cooperative & Progressive

Corporate Spirit

Dedicated, Loyal, United and Enterprising

Management Philosophy

Uphold Corporate Integrity, and Take Pride in Customer Satisfaction.

Culture of the Parent Company － Culture of China Metallurgical Group Corporation (MCC)
The logo consists of letters “ENFI” and the corporation name in Chinese. “ENFI”, short for the Central Engineering Institute for Non-ferrous Metallurgical Industries, represents historical inheritance of the corporation. “E” stands for Engineering, “NF” for Non-ferrous and “I” for “Institute”.

The logo is composed of straight but flexible lines and the ratio between curves and straight lines demonstrates uniqueness of the brand. Letters are positioned like a rational and harmonious formula, which uniquely synchronizes characteristics of nonferrous industry into stability and inventiveness. The streamline design shows the image of ENFI’s responsive, cooperative, progressive, active and energetic style. Meanwhile, the cool color of blue conveys the message of firmness, pragmatism and eternity, and with the combination of frameless design, it symbolizes knowledgeableness, openness and inclusiveness. The whole design, simple but meaningful, distinguishes ENFI’s corporate identity with being technology-oriented and effective.
SUSTAINABILITY
Safe Production
HSE Management System Policy:

Conformance, Commitment and Quality Enhancement

Environmental Protection, Health & Safety and Continual Improvement

HSE Commitment:

Establish and continually improve the quality, environment and occupational health and safety management system according to GB/T19001-2008 Quality Management System Requirements, GB/T24001-2004 Environmental Management System Requirements and User ’ s Guide and GB/T28001-2011 Occupational Health and Safety Management System Standard, so as to ensure the company's products and services (including the results and services of each stage) meet customer requirements and conform to national laws, regulations and industry standards; and to ensure preferential selection of intrinsically safe technology, equipment and materials with less impact on the environment, as well as strengthen environmental safety management of the headquarters and project site, avoid, reduce or control the business processes ’ impact on environment, eliminate and reduce the potential risk of bringing harm to health and safety of employees, improve the health level of employees, and continually improve environmental management, occupational health and safety management performance.

Environmental Protection

Our aim is to create a better tomorrow, and our responsibility is to protect environment for the society.

We have focused on sustainable development of the nonferrous metals industry when performing R&D work, and put our priorities on key technology R&D and popularization for energy efficiency and environmental protection in this industry:

Ø The process of oxygen bottom-blown smelting － reduction smelting of lead in blast furnace makes the comprehensive energy consumption being the highest level of the world.

Ø The new technology of oxygen bottom-blown smelting - side-blown direct reduction of liquid lead slag based on the process of oxygen bottom-blown smelting - reduction smelting of lead in blast furnace makes the comprehensive energy consumption being the highest level of the world.

Ø For the technology of copper smelting with bottom-blown oxygen, no additional fuels are required during the smelting process.

Ø The bottom-blown continuous copper matte converting technology has effectively addressed the current worldwide problem, i.e. fugitive SO2 at low altitudes during converting in PS converter;

Ø The introduction of the rotary kiln - electric furnace technology into China has changed the backward ferro-nickel smelting method, small equipment capacity, high energy consumption, low-grade ferro-nickel, high impurity content, poor environment and severe pollution in China;

Ø Take the lead in establishing China's first largest industrial demonstration line with independent intellectual property rights and closed cycle, and achieving low energy consumption and harmless production of polysilicon.

Ø Conduct studies of the dust collection system, improve its design, and make the technology

characterized by a short process, low energy consumption, etc.

In the project design, construction and operation process, we always adhere to standard designs, stable operation, standardized emissions and information disclosure to achieve harmonious development of the living environment, social responsibility and economic benefits.

In production and business activities, we establish environmental management systems based on internationally recognized standards, identify potential environmental factors, make plans, implement controls, meet regulatory requirements and achieve sustainable development.

Care on Staff

We care about the needs of our employees, improve their quality of life, and enrich their cultural and spiritual life by conducting a variety of activities with distinctive themes.

Each year, we organize cultural and sports events on a regular basis, including the annual basketball match, football match, badminton match, table tennis match, English performance match, chess and cards. These events have been conducted on a regular basis due to the wide audience and high degree of participation, and corresponding associations have been established.

Social Responsibility

Actively participate in public welfare activities, and assume social responsibility

As the capital’ s spiritual civilization unit, we organized our staff to donate money and goods for victims in disaster-stricken areas, residents and children in remote areas, and needy employees. Meanwhile, the Young Volunteers Association, a team committed to the dedication of love and making contributions to the society, was established. Since the establishment, the team has conducted multiple voluntary guiding activities for passers-by around the Military Museum metro station, performed guard duties at multiple road segments and public security monitoring sites during two sessions (NPC & CPPCC) to provide security for convening the conferences, called for and organized staff to carry out activities "Warm Winter with Clothing Donations" and "Clothing Donations for the Poor" within the company, and disinfected and classified clothes donated in a professional manner, delivering warm to the poor in remote areas in the winter.

INNOVATION

Core Technical Know-hows

Over years, ENFI has carried out extensive research work and practice in the field of scientific and technological breakthrough of nonferrous metal mining, mineral processing and smelting. ENFI is the first to apply several new technologies and equipment in the projects, and has obtained several important scientific and technological achievements with independent intellectual property and created several core expertise and proprietary technologies.

At present, ENFI owns nineteen core expertise technologies which cover the specialties of mining, mineral processing, heavy nonferrous metallurgy, rare metal metallurgy, new material, ESP, wastewater treatment, gas cleaning, electrics and equipment and listed below:

1. Oxygen bottom-blowing smelting process

2. Copper-nickel flash smelting process

3.Oxygen-enriched air top-blowing smelting process

4. Extraction technology by pressure leaching

5.Fluid bed roasting of sulfide ore

6. Polysilicon production process

7. Mechanical and electrical integration technology for metallurgical furnace

8. Waste heat recovery from smelting off-gas

9. Tailings dam building

10. Medical and dangerous waste disposal

11. Key equipment and technology for sulfur removal in air control

12. Integrated technology of deep mining

13. Block caving

14. Paste filling

15. Urban sewage treatment

16. SAG/ ball milling process

17. Optimized control for mineral processing

18. Special communication system for mining

19. Electrical control system for shaft hoist

Innovation Platform

We undertake the responsibility for guiding the nonferrous metallurgy development towards a higher level by laying emphasis on core technology and IPR (intellectual property protection), focusing on the high end of international level and attitude of industry development, and making good use of core technology, consistent innovation capability and irreplaceable advantages in full-chain integration. We have formed a platform of sci-tech innovation consisting of one laboratory, one institute, three stations and nine centers through resource integration.

174.China XinXing Construction & Development Co., Ltd.

China XinXing Construction & Development Co., Ltd. (Originally Engineering Team of General Logistics Department of the PLA), established in 1953, is a large construction enterprise which is governed by State-owned Assets Supervision and Administration Commission of the State Council. Enterprise has the Special Qualification of EPC for Building Engineering, Grade-A Qualification of Architectural Design, three first-level qualifications of EPC of Engineering and five first-level qualifications of Specialized Contracting. Possess the Certificate of Security and Confidentiality Conditions for Military Confidential Business Consulting Service and National High and New Tech Enterprises.

Over the past 60 years since its establishment, Xinxing Construction has undertaken a large number of strategic, key and confidential projects of the Party and the State including the renovation of Chairman Mao Memorial Hall, the Central Government Departments and Ministries Project like the Organization Department and the Propaganda Department, Ministry of Foreign Affairs and Public Security, the United Front Work Department of CPC Central Committee, the Great Hall of the People and People's Daily, Series projects of General Office of the CPC Central Committee, key support projects for Celebrating the 70th anniversary of the founding of the people's Republic of China, Series projects of Beijing Daxing International Airport, Municipal road project of Beijing World Horticultural Exposition, Series projects of 2022 Beijing Olympic Winter Games and 2018 Beijing Olympic Games, etc.

Qualification

Business License

Qualification Certificate

ISO9001 Quality Management System Certification issued by Quality Assurance Center

ISO14001 Environment Management System Certification issued by Quality Assurance Center

Honors

The Company has successively won multiple national honors including "National Civilized Unit", “Meritorious Enterprise of Celebrating the 70th anniversary of the founding of the people's Republic of China”,"National May 1st Labor Award", "National Outstanding Construction Enterprise (Jinma Award)", "National Outstanding Construction Enterprise", "National Customer Satisfaction Enterprise", "National Enterprise of Good Creditworthiness", "National AAA Credit Enterprise in Construction Industry", "Top 100 Competitive Enterprises in China’s Construction Industry", "National Outstanding Enterprise in Engineering Construction Quality Management", “China Best Practice Enterprise of Management Systems” "National Advanced Collective for Earthquake Relief in Construction System", "Excellent Unit of Corporate Culture Construction in the 12th Five Year Plan of China" and “National Culture Building Model Enterprise in Construction Industry”.It has been rated as an enterprise with "AAA" credit status by China Construction Bank in 22 consecutive years and as a "Key Client" by the head office of China Construction Bank in 20 consecutive years. It has been awarded as an "Enterprise with A-Class Tax Credit" and an "Advanced Unit for Quality Management in Engineering Construction" by Beijing Municipality. It has been accredited with ISO9001 International Quality Guarantee System, ISO14001 International Environmental Management System and OHSAS18001 Occupational Safety and Health Management System.

Capabilities
Ministry and Commission Government
Building Construction
Infrastructure Construction
Investment Construction
Architectural Design
Professional Field
Overseas Project
Social Responsibility
Xinxing Construction undertook the reconstruction of Beijing YouAn Hospital during SARS
Post-disaster reconstruction in Shifang
Medical staff going to fight SARS
The team from Xinxing Construction went to Jiangyou of Sichuan Province immediately after Wenchuan Earthquake and built 5,027 temporary dwellings in two months
Going to the front line to visit and please the employees
Donating RMB 10 million to build facilities for the 21st World University Games in 2001
Donating for the construction of Xinxing Zuoquan Road for Zuoquan County, Shanxi Province
Participation in many major activities and tasks such as the construction of the Olympic Games facilities and the National Day military parade
Enterprise Philosophy
Corporate Values： Strive for development with superior quality

Corporate Vision： Create excellent project with honesty and integrity； Century-old business for human well-being

Corporate Spirit ： Constant self-improvement, striving for the first

Corporate Style： Resolute, uncompromising, highly disciplined

Quality Concept： Competitive products, famous brand

Safety Concept： Safety should be put before everything else and top priority should be given to responsibility

Service Concept： Exceed customer expectations and win trust with sincerity
175.Pinggao Group

Pinggao Group Co., Ltd (hereinafter referred to as PG)is an pillar enterprise of China's electrical industry directly under China Electrical Equipment Group Co., Ltd (CEE) .PG’s business covers R&D, design, manufacturing, sales, testing of power transmission and distribution equipment, related equipment integration, service and engineering contracting . The main business is R&D，manufacturing, sales and maintenance services MV,HV,UHV AC and DC switchgears.
Pinggao Group , headquartered in Henan, has subsidiaries in Beijing, Tianjin, Shanghai, Zhengzhou, Changsha, Weihai, Langfang, Changchun, and other Chinese cities. Of which PG

Electric Co., Ltd is a public company, Pinggao-Toshiba, Pinggao-Yaskawa, Langfan-Toshiba, Toshiba (Henan) Parts are Sino-Japan joint ventures.PG has successively set up PG India Subsidiary, PG Poland Branch Company, PG Laos Subsidiary, India manufacturing base of GIS, and some offices in Nepal, Pakistan, Ecuador, South Africa, etc. There are national quality inspection centers and three research and development

Pinggao Group adheres to independent innovation, mastering R&D and manufacturing technologies of AC/DC, whole series, and voltage of switch gears. Nearly 80 new products passed the national appraisal, of which more than 20 reached the international leading level. With more than 1,000 patents, PG won more than 60 provincial and ministerial-level science and technology awards, participated in setting more than 50 national and industry standards.

Pinggao Group continues to introduce advanced processing equipment and scientifically improve its manufacturing capabilities. It has 20 specialized assembly workshops, with an annual production capability of 5,000 bays of GIS, 13,000 sets of circuit breakers, 7,000 sets of disconnectors and 110,000 sets of switchgear cabinets. PG also has the ability to manufacture core parts such as vacuum interrupter, insulator manufacturing, conductor silver plating, enclosure manufacturing and coating, precision machining, rubber sealing products and composite insulators.

Pinggao Group insists on building high-quality equipment and serving the construction of power grid. The products are widely used in China's key power projects. PG has provided power transmission & distribution equipments for China's first 550kV high voltage AC transmission project, the first 750kV UHV AC transmission project, the first intelligent demonstration substation of the highest voltage level-750kV Yanan Substation, the first EHV AC demonstration project "southeastern Shanxi - Nanyang - Jingmen", "Anhui-to-east Power Transfer Project", "Jin Su DC Transmission Line" and other national key projects. Qinghai-Tibet Network project and Shandong Mouping substation project PG involved in were respectively awarded “National Gold Award for Quality Engineering” and “China Luban Award for Construction Engineering”.

Pinggao Group's products cover more than 60 countries and regions such as Eastern Europe, Southeast Asia, Middle East, Africa, South America and Oceania.Based on the advantages of technology, manufacturing and brand, Pinggao Group is actively expanding export of equipment supply ， while endeavouring to expand the business of general contracting projects.At present， PG has also independently constructed several EPC projects in Poland, India, Venezuela, Pakistan, Nepal, Laos, Zambia, Syria Kenya and other countries, and achieved great success.

Pinggao Group will continue to carry forward the enterprise spirit of “In search of Excellence and in Pursuit of Out-Performance ” , adhere to the core values of “ customer-centric, professional focus, continuous improvement” and strive to build a world-class electrical group.
Qualification and Honors
Quality Management System and ISO Certificates

Strictly carry out ISO9001 quality management procedures

Introduce the Crosby “Zero Defect “management philosophy

Use Six-Sigma management tool

Advance Quality Management Innovation
Enterprise Culture
Basic Concepts of the Company

Orientation: Leader of the global energy revolution and forerunner of serving national economy and the people's livelihood

Mission: promote re-electrification and build energy Internet to meet power demand in a clean and green way

Objective: people's electric power is for people

Development concepts of the grid: safe, quality, economical, green and efficient

Core values: customer-centered, professional and dedicated, continuously improved

Spirit: In search of Excellence and in Pursuit of Out-Performance
Key Projects
Domestic Projects
Overseas Projects
Research and Technology
Scientific Research System
Research Results
Test Capability
Patents
Technology of developing high-end and intelligent switchgear

Technical research capability: It has accumulated rich experience in basic technical research of switchgear and mastered key technologies such as analysis and testing of arc extinguish mechanism, electric field, airflow field and circuit breaker characteristic parameters. Especially in terms of the operating mechanism of extra-high voltage and ultra-high voltage switchgear, the hydraulic operating mechanism with high power and reliability has been successfully developed;

the serialization and modularization of the hydraulic mechanism have been fully realized; the comprehensive technical capability in the research of hydraulic systems has been improved. In the research of large insulation parts, the insulation pouring technology of ultra-high voltage, extra-high voltage and high voltage levels has been fully mastered.
Equipment manufacturing capacity: It has the first-class domestic CNC machining center, shell welding production line, large electroplating production line of aluminum and copper parts, surface coating production line, imported epoxy pouring insulation parts production line and GIS / GCB assembly production line, and has become a leading switch manufacturing base in China, laying a solid foundation for product development.

Full series, full voltage grade switchgear: It has formed the national first-class and international advanced R&D technology of full series, AC / DC, full voltage grade series switches with fully independent intellectual property rights. It also took the lead in becoming the first switch manufacturer to develop 800 kV and 1100 kV GIS, and the 1100 kV GIS provided for UHV demonstration projects won the "2010 China Power Quality Engineering Award" and "2009 First Prize for Excellent Engineering Design in Power Industry" awarded by State Grid Corporation of China. LW6-550 Circuit Breaker won the National Gold Medal; the "Circuit Breaker with 220 kV and above" has been the famous brand in China; "PG" brand won the "China Famous Brand".
Pinggao Group Co., Ltd. is a professional manufacturer of HV electrical apparatus. It has accumulated rich experience in production and manufacturing, and ensures product quality by adopting mature and advanced manufacturing technology, selecting advanced high-performance materials and advanced processing technology. It has the capability of machining, metal shell manufacturing, epoxy resin insulation pouring, pressing of rubber parts, injection of plastic parts, manufacturing of various coils, electrochemical surface treatment, surface coating, forging, welding, heat treatment, casting, tool and mold manufacturing and assembly, and is capable of detection means, such as chemical analysis, metallographic analysis, mechanical property test, electrical property test, environmental test, particle analysis, chromatographic analysis, physical property test, ultrasonic flaw detection, radiographic flaw detection, fluorescent flaw detection and sealing test. All types of HV test equipment meet the requirements of the partial type test and ex-factory test of products.

So far, 349 national patents have been applied, including 39 patents for invention, and 228 patents have been authorized, including 9 patents for invention. A lot of achievements have been made, including 51 scientific and technological achievements at provincial and ministerial levels and above, 2 items of "China Torch Program", 2 items of "National Science & Technology Pillar Program" and 2 items of "Key S&T Special Projects in Henan". A lot of prizes have been acquired, including 1 special prize and 1 second prize of "State Grid Corporation Science and Technology Progress Award" and 1 special prize, 2 second prizes and 3 third prizes of "Science and Technology Award of Mechanical Industry in China".

176.SINOSOAR
Sino Soar Hybrid (Beijing) Technology Co., Ltd. (Abbr. SINOSOAR) is an international high-tech company specialized in solar hybrid and off grid fields. SINOSOAR’s main business scope covers R&D, system integration, project development, engineering, procurement, construction,

maintenance as well as project financing and investment. Relaying on strong R&D capabilities, advanced supply chain and rich experiences in resources integration, SINOSOAR provides customized turnkey solutions for solar hybrid and off-grid projects all over the world.
The development strategy of SINOSOAR is to expand our business chains upstream and downstream (including financing & investment) during the implementation of solar hybrid and off-grid projects. Our aim is to supply clean and sustainable power to industrial and individual users at isolated islands and remote undeveloped areas from pole to pole.
So far, the total contract value of SINOSOAR projects has exceeded one billion Chinese Yuan, and the projects spread across more than 10 countries and regions in Asia, Africa, Oceania, and Latin America. SINOSOAR has built more than 30 hybrid projects and installed over 400,000 sets off-grid solar power system, benefiting over 3.2million people.
Our Culture
Sinosoar Mission
Power the world with hybrid energy
Sinosoar Vision
Century mega enterprise, world top 3 enterprise in solar hybrid field
Sinosoar Value
Honest & Reliable, Striving & Progressive, Professional & Effective, Teamwork, Teaching & Learning, Open-minded & Generous
Sinosoar Operation Principle
Client benefit upmost, Employee benefit next, Shareholder benefit last
Sinosoar Operation Concept
Let all staff obtain material and spiritual happiness productively
Qualifications & Certifications
Invention Patents
High-tech Certificates
copyright of computer software
ISO System Certification
HSE certification
Our Team
SINOSOAR is proud of its sophisticated R&D team, the self-developed EMS (Energy Management System), SCADA (Supervisory Control and Data Acquisition) and PCS (Power Conversion System) have been launched and successfully applied to the solar hybrid projects in Maldives, Myanmar, Uganda, Suriname etc.
SINOSOAR has obtained the Certificate of National High-tech Enterprise and the Certificate of Zhongguancun High-tech Enterprise. SINOSOAR has been authorized with 9 national invention patents, 25 certificates for computer software copy right, and 35 utility model invention patents. In addition, SINOSOAR has been issued with the ISO Certificates and HSE Certificate.
The technical team of SINOSOAR has more than 15 years of experience in design and implementation of solar hybrid and off grid projects. With the service tenet of “Customer First”, SINOSOAR starts from customer demand and ends with customer satisfaction, provides complete solar hybrid and off grid solutions based on the actual requirements of customers.
SOLUTIONS
System Intro

Solar mini-grid is a local and independent energy system that can generate, distribute, store and regulate current. It is basically a smaller version of the main grid, and can run with the large grid, or in "island mode". In case of necessity and actual operation, it can also switch between that two mode. Solar mini-grid can extract energy from conventional energy and renewable energy or a combination of them. The more optimized Solar Hybrid grid is for using renewable energy sources (such as solar energy), the lower its environmental impact and long-term operation cost.

Sinosoar can provide one-stop turnkey service for solar hybrid power system power supply system, including design, product integration, system installation and operation and maintenance services. As a whole set of power generation system, the key technology of system integration lies in the response and guarantee of solar hybrid power system to customer's electricity demand, as well as the matching and coordinated operation of main components. The technical team of Sino Soar Hybrid (Beijing) Technology Co., Ltd. has more than 15 years of experience in solar mini-grid product design and integration. Sino Soar always takes the customer at first priority, starting from customer needs, and finalizing in customer satisfaction. Sino Soar provides perfect mini-grid system solutions based on the actual application needs of customers.

System advantages

The mini-grid quickly builds power grid in local areas, saving construction costs.

On a small island far away from the mainland, it is necessary to lay submarine cables and erect iron towers to supply electricity through a large power grid. This is costly and time-consuming. The entire mini-grid system is cheaper and more economical than building a large power grid.

Mini-grid is very flexible and can realize the local consumption of solar energy.

Remote areas are rich in solar energy resources. The mini-grid uses solar energy to generate electricity and realizes local consumption for local residents. The extra electricity can be stored for later use.

The mini-grid has high power supply stability.

In some remote areas, although the power grid is already connected, the electricity supply is unstable and there are frequent power outages. After the use of the mini-grid as a supporting tool, the reliability of power supply can be guaranteed. After the main power is cut off, the mini -grid can operate in isolated mode, which greatly improves the productivity and living standards of local residents.

Applications

Solar hybrid power system for islands

According to the statistics of island number from the maps published by different countries, there are about 100,000 islands in the world. Island power supply is an important field for the application of renewable energy mini-grid. The construction of renewable energy resources, such as photovoltaic power generation, wind power generation, and energy storage systems, could provide uninterrupted supply of pollution-free energy to those isolated islands. For islands with existing diesel power generation, the renewable energy could be integrated with diesel generator to reduce the fuel consumption, to improve the economic efficiency, as well as to reduce the environmental pollution and carbon emissions.

Solar hybrid power system for Mines and oilfields

Mines and oilfields are mostly located in remote areas without electricity access while with larger electricity consumption. The power supply cost is relatively high and the power stability is low no

matter connecting with new state grid or consuming with heavy oil and diesel for power generation. The Solar Hybrid System could effectively reduce the cost of electricity consumption as well as improve greatly the reliability of power supply.
Solar hybrid power system for remote villages and towns
In remote areas, the large power grid can not be covered or the laying cost is high, so the independent hybrid power system can be used for power supply. To ensure the stability of power, in a certain period of time from the grid to continue to operate. when the power grid has the conditions to cover the area, the hybrid power system can operate independently or be directly incorporated into the power grid. The switching between the two modes can be easily realized by EMS control logic of Sinosoar.
177.China Railway Engineering Corporation

A backbone enterprise of China Railway Engineering Corporation, which is one of the World Top 500, China Railway No.10 Engineering Group Co., Ltd. is a super-large state-held cross-industry, transnational construction corporation, mainly specializing in contracting architectural engineering. It possesses a series of government-registered or issued qualification certificates which include: Super Class General Contractor of Railway Engineering Construction; Class A General Contractor of Highway Projects, Municipal Works, Building Works; Class A Special Contractor of many categories of engineering works, ranging from bridge, tunnel, railway track-laying and girder-erection project, environmental protection project, steel-structure works, railway electrification, communication, signaling and electric power projects, to road bed and surface, earth & stone works, architectural decoration project, intelligent building, electromechanical equipment installation engineering; Class B General Contractor of blasting demolition, water conservancy, hydropower project and telecommunication; （Ⅱ）class qualification of railway design, the second class of estate development, specialized Contractor of urban mass transit project. Additionally, it has also class A qualification certificate for contracting engineering project abroad and undertaking comprehensive supporting project of foreign aid.

With total assets of 20.13 billion Yuan, including registered capital of 1.334 billion Yuan, as well as the 24 subsidiaries and branches, China Railway No.10 Engineering Group Co., Ltd. retains an annual construction capacity of over 30 billion Yuan. Moreover, among the nearly 14,237 staff members it has over 8,323 technicians, of whom 2,737 ones have senior or intermediate-level professional titles, and nearly 300 ones are awarded Class-one qualification certificates of registered constructor, ensuring its strong competitive power.

For years, China Railway No.10 Engineering Group Co., Ltd. has made remarkable achievements in such construction fields as railway, urban mass transit, highway, municipal-service works and property development. Up to now, it has taken part in the construction of a great number of national key railway projects such as: about 100 large-scale railway lines including Beijing-Kowloon, Qinghai-Tibet, Beijing-Shanghai, Shanghai-Hangzhou, Hangzhou-Nanjing, Shanghai-Nanjing, Yichang-Wanzhou, Jiaozhou-Xinyi, Wenzhou-Fuzhou, Ningbo-Wenzhou, Hefei-Wuhan, Taiyuan-Yinchuan, Xiangtang-Putian, Lanzhou-Chongqing, Datong-Xi’an, Kunming-Nanning and south-centre railways，some of whom being high-speed lines or passenger transit special railway lines, a number of railway terminals and stations including those in the

cities of Ji’ nan, Qingdao, Xuzhou, Yantai, Taizhou and West Ji’ nan, involving newly building, renovating and extending over 6,0oo kilometers of trunk or branch lines. It has successively taken part in the construction of over 100 expressways, including Ji’ nan-Qingdao, Beijing-Shanghai, Lianyungang- Khorgos expressways , totaling 1,500 kilometers, and undertaken the construction of over 1,000 large or extra-large bridges. In the cities of Guangzhou, Chengdu, Dalian, Wuhan, Fuzhou, Changchun, Xi’ an, and Guiyang, it has undertaken a number of urban mass transit projects, and in many other cities of the country, fulfilled the construction of more than 1,000 engineering projects of other categories, including high-rise buildings, large workshops, urban interchanges, electrification works, automobile testing dromes, golf courses, environmental protection works and water works, showing its considerable comprehensive strength. Moreover, it has in recent years set foot in the construction of overseas engineering projects, invested to develop mineral resources in the countries of Belorussia, Venezuela, South Sudan, Uganda, Kenya, Sri Lanka and so on.

Based on its consistent and strict quality control policy, a great number of engineering projects of the corporation won various high-quality awards, of whom 17 ones are ‘China Luban Prize for Achievements in Construction Project’, ‘China Zhantianyou Prize for Civil Engineering’ and ‘the National Client-satisfied Project Prize’, all being state-level high-quality awards, and 67 ones are provincial/ ministerial level quality awards, including the Taishan Cup Prize. Due to its unremitting efforts in scientific & technological innovation, 13 construction techniques developed by the corporation were adopted as state-level construction standards, and 42 items as provincial level ones. And it has also gained 102 patents. By relying on strict and scientific management, the corporation has in succession gained the certificates for quality control system, environmental management system and professional health & safety management system, and was awarded a number of titles, which include those of National Excellent Construction Enterprise, National Excellent Creditable Enterprise, State Excellent Enterprise for Ideological & Cultural Work, State Advanced Enterprise in Highway Construction Industry, State Advanced Quality and Efficiency Oriented Construction Enterprise, State Contract-Observing and Promise-Keeping Enterprise, State Advanced Construction Enterprise in Scientific & Technological Innovation and Progress, Shandong Provincial Top 10 Units for Enterprise Culture, Shandong Provincial Advanced Enterprise in Building Harmonious Labour Relations, as well as the Certificate of Accomplishment for Richening Citizens and Revitalizing Shandong and Shandong Provincial Top Model Creditable Enterprise.

Corporation Culture

Company logo

The logo is formed by the Chinese short name "China Railway" and English "CREC", and use blue as the standard colour indicating the science and technology. Interlacing earth background shows the company's strategic and global vision. The regular, vigorous character "Gong" is like a steel rail carrying the glorious history, and at the same time, like a giant building, indicating the thriving future.

China Railway No.10 Group Engineering Co., Ltd core values:

Cherish the favour and try to repay it

It mainly contains two levels of meanings: In the view of Corporation, it should thank the society to provide a good development platform, appreciate Employers for providing the foundation for the live and grow up, and also be grateful for the trust and support from the Employees. We should think based on the fact, with the harmonious and healthy development of corporation, feedback to the society, the Employers and our staff. For individuals, it is need to be grateful for the company which provides the job and a platform to display their talent, thanks the superior organization, and also thank the training and caring from Leaders, Employees should learn to love colleagues, love the collective, love the company, and love the society, do their job well in a down-to-earth way, do their contribution during daily works, and use hardworking and good performance repay to the society, the origination and the company.

Leadership Speech

Sincerely welcome you to visit the website of China Railway No.10 Engineering Group Co., Ltd! Thank you for your kindness and concern.

China Railway No.10 Engineering Group Co., Ltd is formed by the former Ji'nan Railway Engineering (Group) Co. Ltd., the Third Engineering Limited Company of China Railway No.3 Engineering Group, the Third Engineering Limited Company of China Railway No.4 Engineering Group. It is the subsidiary of China Railway Engineering Corporation, and is a large cross industry, cross-border business group with 50 years glorious history in construction field.

The benefit of public is our goal, contributing to society is our value embodiment. We are going to catch the fine developing opportunities both in domestic and international market, guided by Scientific Outlook on Development, implement enterprise development strategy, continue to deepen the reform, accelerate structural adjustment. Standing on the core position of construction business, also we are going to expand the Engineering upstream areas, and form our business into three segments; the traditional Engineering, the upstream industry, and overseas businesses. Enhancing the company's profitability and the ability of sustainable development, promote a sound and rapid development of enterprises; continue to achieve mutual benefit with clients. China Railway No.10 Engineering Group will rely on the leading brand of China Railway, continue to forge ahead, and continue to write a new chapter in development.

I hope this website will be the windows for you to know China Railway Engineering No.10 Group and the bridge of mutual communication. Let us work together to make a better future.

Business And Products

HIGH-SPEED RAILWAY

RAILWAY

URBAN MASS TRANSIT

MUNICIPAL

HIGHWAY

BRIDGE

TUNNEL

INDUSTRIAL & CIVIL

HYDRAULIC

ELECTRIFICATION
REAL ESTATE
OVERSEA PROJECT
SPORT FACILITIE
178.AVIC INTL

AVIC International Holding Corporation (AVIC INTL) is a global shareholding enterprise affiliated with Aviation Industry Corporation of China (AVIC). The main business involves four segments: aviation business, high-end manufacturing, overseas public service, and service & trade. The corporation owns six domestic and overseas public companies and has established branches in sixty countries and regions.

With aviation as its core business, AVIC INTL focuses on the development of integrated aviation supply chain service systems, aviation industry networking and industrial data platforms, international aviation technology cooperation, integrated services for aviation standard parts and products, special display and human – computer interaction, aviation operations and support, tendering services, and more.

In everything we do, we uphold the mission of “Go beyond Commerce for a Better World.” We also take advantage of global networks and platforms and actively participate in the Belt and Road Initiative. Focusing on priority sectors, AVIC INTL has been engaged in project planning, project finance management, export of electromechanical products, general contracting, and the operation and maintenance of overseas engineering projects. These efforts have established a positive brand impression in overseas markets and effectively promoted local and regional economic prosperity.

Over forty years of reform and development, AVIC INTL has incorporated innovation, entrepreneurship, market orientation, and global orientation into the corporation. Noted brands under AVIC INTL include TIANMA, SCC, RAINBOW, and FIYTA. Over time, these brands have developed and established competitive advantages in their relevant fields. The corporation serves as a leader in building national brands and is committed to enhancing the presence of “Made in China” and Chinese brands worldwide. This is accomplished through constantly promoting transformation within our business model and technology upgrades.
Corporate Culture
Mission
Go beyond commerce for a better world
Vision
To foster a first-class enterprise group and to become a world-leading enterprise loved by its

employees, praised by its customers and respected by the society.

Values

People-oriented

Responsibility

Partnership

Innovation

The unity of knowing and doing

Chairman’s Message

All enterprises follow the same presumption at the outset: businesses are sustainable forever. Yet, up to this day, none of us is sure whether there will be an enterprise that can really make it happen, for mankind currently cannot determine the end of time. Emerging from the Republic’s aviation industry decades ago, AVIC INTERNATIONAL has undergone restructuring and has been renamed and relocated several times. However, our original intention to “serve the country through the development of the aviation industry” has remained unchanged.

As with all enterprises, we cannot accurately predict the ultimate prospects of AVIC INTERNATIONAL. In the foreseeable future, however, we will always be dedicated to serving the aviation industry, constantly exploring the international market, seeking technological cooperation and sharing, and turning AVIC INTERNATIONAL into an excellent aviation manufacturer and service provider. Meanwhile, by continuing to promote technological innovation, business model innovation and management innovation in the advantageous fields of electronics, international businesses and the modern service industry, we will strive to maintain our leading position in the industry, create value for shareholders, provide the stage for our employees and pursue sustainable growth.

AVIC INTERNATIONAL’s mission is to “go beyond commerce for a better world”. While paying close attention to its own development, a great company will also be concerned about the fate and well-being of the country, the nation and the entire human race. As an international company, AVIC INTERNATIONAL is willing to join hands with all friends for a better world!

History

Founded in the initial period of China’s reform and opening-up drive,over the past three decades, AVIC INTERNATIONAL has exported more than a thousand of aircrafts and imported several hundred aircrafts.From introducing foreign technology to taking part in independent R&D and to having products exported around the world, we have witnessed a brilliant history of the aviation industry in China.

As an important member of China’s aviation industry,AVIC INTERNATIONAL is a pioneer in the development and reform of the industry and serves as a comprehensive platform for the aviation industry to develop overseas markets, set up related businesses and expand international investment.

Service and Trade

Seizing the opportunity of consumption upgrading and IT innovation, AVIC INTL puts brand building at the core, establishes new business models, creates values for clients, and take the

lead of high-quality life. A group of famous brands have been cultivated such as RAINBOW, FIYTA, Harmony World Watch Center and Grand Skylight.

Commercial Retail

Hotel Management

Integrated Management of the Steel Industry Chain

Steel Raw Materials and Steel Products

Asphalt and Mechanical& Electrical Products

CSR

Social Responsibility System

While seeking for self-development of the company, we are playing an active role in serving and paying back the society, and creating common values for all parties involved, in order to realize the harmonious development of the company, employees, and the society.

Our Concept of Social Responsibilities

Philanthropic Responsibilities

Philanthropy programs /corporate citizenship

Ethical Responsibilities

Business ethics/ environmental protection/ customer orientation/care for people

Legal Responsibilities

Abidance by law

Economic Responsibilities

Provision of valuable products and service to The society

Fulfilling social responsibilities is not simply about giving money out; instead it should combine with promoting the reform and development of the company, and should be regarded as the key part of the strategic transformation and improvement of international competitiveness of AVIC INTERNATIONAL to realize sustainable development; it should adapt to the reality of the company, rely on the aviation strength, highlight the priorities, advance step by step to achieve substantial outcomes. The fulfilling of social responsibilities should be incorporated with the building of a harmonious company, honor the laws and integrity, guarantee production safety, and safeguard the legitimate rights and interests of employees to promote their all-round development. While realizing self development, the company should actively serve and pay back the society, and create common values for all parties involved, in order to realize the harmonious development of the company, employees, and the society.

The Happiness Station

The Happiness Station of AVIC INTERNATIONAL covers services of staff care hotline, professional training, care for expatriates and crisis intervention. As a bridge between the company and its staff, the project aims to help staff elevate their physical and mental health, achieve work-life balance and improve their psychological capital and self-worth, in an effort to enhance the performance of the company and its staff and create more happy experience for the staff!

Rural Teacher Training

Themed as “give a man a fish and you feed him for a day; show him how to catch fish, and you feed him for a lifetime”, “Blue Chalk” Rural Teachers Training Project is designed to recruit excellent teachers from Beijing, Shenzhen and other areas with an thriving education to train teachers from rural primary and middle schools in impoverished areas with poor education and exchange ideas on education theories and pedagogics with them, so as to support children from

rural areas to realize their dreams. Until now, it has trained and benefitted more than 20,000 teachers from over 8,000 rural schools.

Africa Tech Challenge(ATC)

ATC is an overseas public welfare event launched by AVIC INTERNATIONAL in Africa that attracts outstanding young people from Kenya, Ghana, Uganda and Zambia to participate in the training and competition. To date, it has trained more than 290 technical teachers and students, casting a glow of light for African youth.

Careers

People-oriented

AVIC INTL Business School

AVIC INTERNATIONAL is a distinctive new-style state-owned enterprise that has taken "go beyond commerce for a better world" as its mission. The employees/potential employees of AVIC INTERNATIONAL are chosen on the basis of its values, which are "people-oriented, responsibility, partnership, innovation and the unity of knowing and doing."

This is a unique, highly mission-driven ("go beyond commerce for a better world"), and new-style state-owned enterprise.

In its three decades of glorious history, AVIC INTERNATIONAL has made great contribution to the modernization of the aviation industry and the growth of the national economy.

In its three decades of inspiring journey, in the pursuit of its mission and business success, AVIC International has nurtured an elite team who goes beyond commerce and are committed to taking the lead.

The major task for the Human Resources Department is to help the company accomplish its strategic goals and the employees fulfill their own roles through staff selection, employment, training and stimulation.

AVIC International, a company that has enjoyed a stable and rapid development, has been committed to exploring and creating a new working mechanism for its employees. It offers a competitive "incentive pay system" for its employees. It ensures that they can live a life of dignity. It provides a diversified development platform without glass ceiling. In a few words, it has created a very rewarding working environment and cultural atmosphere for them.

Building a platform for its employees to pursue career development and self-realization, AVIC INTERNATIONAL has enabled its employees to grow with the enterprise and therefore set a good example for other companies.

AVIC International has always been concerned about the development of professional competence of each employee. With AVIC International Business School as the main channel for trainings, and through multiple forms such as job rotation, stationed overseas positions and secondment, we develop internationally competitive talents.

The employees/potential employees of AVIC International are chosen on the basis of its values,

which are “people-orientation, responsibility, partnership, innovation and the unity of knowing and doing.”

AVIC International, a company that pursues excellence, welcomes all of you who have a good character and professional competence and identify with the values of our company to join us! Let’s move forward together and help AVIC INTERNATIONAL fulfill its mission of “fostering a first-class enterprise group to become a multinational outstanding enterprise that is loved by employees, praised by customers, and respected by society”.

179.HNAC Technology Co., Ltd.

HNAC Technology Co., Ltd. (Stock Code: 300490) is a large listed group company that provides overall solutions for water conservancy, electric power, environmental protection &water treatment, and industrial control etc. HNAC have 6 bases in Changsha, Beijing, Wuhan and Shenzhen city, China, which have overseas branches and offices in Central African Republic, Chile, Pakistan, Indonesia, Uzbekistan and Zambia.

HNAC has a global market share of automation control equipment for power stations and pumping stations, which is the United Nations Industrial Development Organization International Small Hydropower Center Control Equipment Manufacturing Base. HNAC is the member of International Hydropower Association and authorized as the major drafter for more than 10 Chinese national industry standards in water conservancy, hydropower and new energy.

HANC has nearly 30 years of project implementation experience, with comprehensive service capabilities such as survey and design, equipment manufacturing, engineering implementation, intelligent operation and maintenance, and investment and financing.

180.Beijing TRT Group

Long history

Tong Ren Tang Founded in 1669. It served eight Qing emperors for 188 years since it was designated to provide traditional Chinese medicine to the imperial family in 1723。

The founding of the People’s Republic of China in 1949 gave a new life to weather-beaten TRT.In 1992, Beijing TRT Group was established,and became a wholly state-owned company in 2001. It has gradually established a modern corporate system.

In 1997, the subsidiary Beijing Tong Ren Tang Co., Ltd. was listed in Shanghai Stock Exchange,while Beijing Tong Ren TangTechnology Development Co., Ltd. were listed in Hong Kong Stock Exchange in 2000.Beijing Tong Ren Tang Chinese Medicine Co., Ltd.were listed in Hong Kong Stock Exchangein2013, These listings strengthened the overall competitiveness of Beijing TRT Group.

In 2019, answering the Party’s call to develop TCM, the group invited the third-party company (focus on "two centenary goals")to provide strategic consultancy services and map out the development strategy in the new era, taking the group onto a journey of high-quality growth.

Glorious achievements

Specializing in traditional Chinese medicine and therapy,Beijing TRT Group focuses on TCM production as its core pillar, supplemented by four other pillars of health and wellness, senior medical care, commerce and retail, and international business-shaping a health industry chain that covers herb growing, TCM production, sales, medical services, healthcare, and R&D.

With the establishment of its Hong Kong manufacturing base as a milestone,Tong Ren Tang started from Beijing, expands in China and becomes international. It now has business or services in 28 countries and regions in 5 continents, helping internationalize TCM.

The group has seven sub-groups, two institutes, many subsidiaries, and more than 2400 retail points and medical organizations.It has 36 production bases and over 100 modern production lines that can produce more than 2600 medicines and healthcare food in six categories and 20 types. Many of its TCM are household names, such as Cow-bezoar Bolus for Resurrection, Tongren Cow-Bezoar Sedative Bolus and Tongren White Phoenix Bolus of Black-Bone Chicken.

In terms of R&D, the group has established a complete system that includes a national engineering center and a post-doctoral research station. Since 2016, the group has developed 265 new products,among which, Morinda Officinalis Oligosaccharide Capsule, as the first TCM for depression treatment, won the first prize of Beijing Science and Technology Award. The group is dedicated to innovating techniques to help address challenges facing the industry and improve the automation of TCM production.

In2018, the group registered a revenue of 19 billion yuan,a profit of 2.7 billion yuan and an asset of more than 29 billion yuan.Since the group’s founding in 1992, it has paid 36.5 billion yuan in tax, increasing the value of state capital.The group also won many awards and honorary titles such as China Trademark Gold Prize - Trademark Application,Madrid System for International Trademark Registration Special Award, the first Quality Management Award by the Beijing Government, and the Pillar Enterprise Award of the Medical Industry for the 70th Anniversary of the PRC.

Rich culture

As one of the first China Time-honored Brands, Beijing TRT Group goes along a growth path that coordinates both business growth and cultural development. As a state-owned company, the group fulfills all its political, economic, social and cultural responsibilities.It plays a unique role in advancing the TCM industry and carrying on the TCM culture.

TRT started its business with the belief to “promotepeople’s health with noble morality and kindness”. “Serving the people’s health soul and heart” is TRT’s motto.It follows the codes of conduct from the ancient times, such as “although patients do not see the process of making TCM, we should always do it earnestly”, “no manpower shall be spared, no matter how complicated the procedures of production are”, “no material shall be reduced, no matter how much the cost is”, “it is our hope that people will never get ill, even if that means we won’t

have any business.” TRT is therefore known for its unique prescription, high-quality raw materials, great techniques and outstanding therapeutic effects” - contributing to its fame of quality and credibility.

TRT carries on craftsmanship from generation to generation.The group has three national intangible heritage programs - TRT TCM culture, traditional technique of TCM preparation,and preparation technique of Cow-bezoar Bolus for Resurrection. The group also has a number of other intangible heritage programs at municipal and district levels,37 heirs of intangible heritage at national, municipal and district levels, 14 TRT master TCM therapists, 8 TCM masters and 20 heirs of special techniques.

The group assigns a priority to the education of socialist core values,innovation of cultural inheritance, and evaluation on both Party building and business performance. Through these efforts, the group has developed a culture of valuing the brand, staying united with a strong team spirit and striving for excellence,which helps fuel the growth of the group.

Grand blueprint

Based on the new era and new mission,Beijing TRT Group will be guided by Xi Jinping's socialist ideology with Chinese characteristics in the new era and the spirit of the 19th National Congress of the Communist Party of China and the National Congress of traditional Chinese Medicine to keep abreast with the times and people’s need in the new era.Its priority should transit from “medical services” to “promoting people’s health”.It will stick to steady growth to improve,strengthen and refine its “three-phase” growth strategy for a high-quality development.With Party building, quality and credibility as the three cornerstones, it aims to build a world-class TCM health group under the vision of serving people’s health around the world.

Rosy future

In 1956, Chairman Mao Zedong said that TRT must be kept forever, which reflected his high expectation on TRT.General Secretary Xi Jinping has called to “promote the high-quality growth of the TCM industry”.

The group will work hard to live up to the expectations of the Party and the people.Leveraging all the opportunities facing the TCM industry,always retaining the original intention, the bravery undertaking the mission,the determination to keep moving forward, getting the high quality development achievement,TRT is committed to its mission of “serving people’s health and revitalizing the TCM” to achieve high-quality growth in the new era.
Brand Culture

“Cultivate both noble morality and benevolence to do good to society and to keep people healthy” is a new summary of Tongrentang’s original desire, mission and spirit as a Traditional Chinese Medicine enterprise, showing the ideal and pursuit that Tongrentang people are committed to serving human and preserving their health, and also regard this as their own duties.

The saying “the strenuous efforts in making exquisite medicines can rarely be seen, but heaven knows our sincerity” is not only a traditional rule comprehensively followed by Traditional Chinese Medicine sector in China, but also a self-discipline rule passed on generations among Tongrentang people.

Though the processing of medicines was complicated, we must not save labors; though the medicinal materials were expensive, we must not reduce any of them.The “two mustn’t” highly aligns with the “integrity” required in the core values of Chinese socialism, which needs to be promoted with great efforts. Up to now, the “two mustn’t” is well-known to every household. Not only Tongrentang people regard it as “family heirloom” to carry forward, but many enterprises also incorporate it into their own business philosophy.

We wish there were no illnesses in the world, even though the drug shelves were covered with dust.

181.Huayou Cobalt

Huayou Cobalt is a new high-tech enterprise founded in 2002 and specializing in research and development, manufacturing business of new energy Li-ion battery materials and new cobalt materials.

After ten years of development, we’ve completed the global layout of headquarters in Tongxiang, with overseas mineral resources security and manufacturing base in China while market in the world; We’ve also formed three business units including Resources Development, New Materials Manufacturing and New Energy Manufacturing, and created a new energy lithium battery industrial ecology from cobalt and nickel mineral development, refining, and further processing of Li-ion battery cathode materials, with reusing and recycling.

With the mission of creating value for customer and leading industrial development, Huayou Cobalt is firmly to develop the business with the transformation path of more upstream resources security, global market promotion and manufacturing capacity-building, and adhere to the development strategy of “Two new areas and Three trends”, and is committed to becoming the global leader in new energy Li-ion battery materials industry.

Strategy as Outline, Talent Oriented, Mechanism Driven, Culture Escorted

Honor

Leader of Benefit Per Mu in Zhejiang Province(Manufacturing Enterprise)

Honorary Credential of Donation(Employees Donation for Fighting COVID-19)

The Top 100 Enterprises with the fastest Growth in Zhejiang Province

Corporate Social Responsibility

Since its establishment in 2002, the company has adhered to the development philosophy of "innovation, coordination, openness, green, and sharing", attached great importance to corporate internal social responsibility governance and sustainable development, and is a leader in the construction of social responsibility in the industry. Huayou has been practicing the

responsibility concept of "production and ecological harmony, economic benefits and social benefits unity", adhering to the green development, vigorously promote energy saving and consumption reduction, clean production, circular economy, green manufacturing, to create a resource saving, environmentally friendly, leading industry benchmark; adhere to Shared development, care for employees, comprehensively protect the rights and interests of employees, realize the mutual promotion between the healthy development of enterprises and the improvement of employee value, and pay attention to people's livelihood; actively engaged in social welfare undertakings, in the performance of social responsibility to promote the common progress of enterprises and society.
Labor Practices and Human Rights
Labor：

With the continuous deepening of the "Two New Areas ＆ and Three Trends" strategy of Huayou Cobalt, the internationalization degree of the company is constantly improving, and the team is becoming more and more diversified. The company insists on the principle of "equality, fairness and impartiality" and provides equal employment opportunities for people of different nationalities, races, genders, ages and educational backgrounds.

The company respects and upholds international human rights law, strictly abides by national and local laws and regulations, establishes a standardized employment system, which strictly prohibits child labor, the use of debt (including debt slaves), slavery, prison or bound labor, and resolutely combats human trafficking Continuously improve, optimize the human resources management system, protect the legitimate rights and interests of employees, protect the health and safety of employees, against forced labor, and against occupational discrimination. There was no corporate discrimination case in the company since the establishment of the company.

The company is committed to providing an equal, fair, diversified working environment, everyone is treated fairly at work, regardless of race, age, sex, religion, beliefs and other factors, and fair opportunities for employment will be given based on the ability of employees.

The company insists on people-oriented, pays attention to personnel's ability promotion and the career development, provides the vocational training and the development channel for the staff, to realize common growth and development of both the company and staff's.

Compensation and benefits is a very important factor to attract and retain talents, and also an important guarantee to improve the lives of employees. Huayou Cobalt insists that the salary level is competitive in the same industry and the region, allowing employees to work decently and live in dignity. According to the requirements of laws and regulations, the company implements the comprehensive working hour calculation system for grass-roots employees, the standard working hour system for management and technical personnel, and strictly controls the overtime hours to protect the rest rights and interests of employees; If employees work overtime on weekdays and holidays, we will give overtime pay according to law and take time off for work overtime on Sunday. The company provides employees with "social security packages and housing provident fund" social welfare guarantee, and sets up reasonable salary structure,

including post salary, performance encourages employees to continuously improve their professional ability, reflects the culsalary, skill allowance, management allowance, overtime pay, post allowance, night shift allowance, monthly performance bonus, annual performance salary and other subsidies. It not only embodies the reasonable return to employees' labor, but also tural concept of "high performance, high income; enterprise efficiency, income increase", and guides employees to strive for common development and growth with the enterprise. In addition to the benefits provided by statute, the company offers a variety of special benefits, such as: providing employees with event condolences, talent apartment, rental subsidy, talent allowance, commercial medical insurance and other welfare systems.

Occupational health and safety management:

Over the years, the company has persisted in earnestly implementing and implementing the national occupational health and safety production guidelines, policies and the spirit of documents, actively learning advanced occupational health and safety management models at home and abroad, combining with its own reality, comprehensively constructing occupational health and safety management system, production safety standardization system, and constantly improving occupational health and safety management level.

Environment

The company is committed to becoming a global leader in new energy materials for lithium battery, and realize the coordination and promotion of environmental protection and enterprise benefits. It strictly implements the requirements of environmental protection laws and regulations such as the Environmental Protection Law, the Environmental Impact Assessment Law, various special environmental protection laws, and the environmental protection management regulations of construction projects in the course of project construction, production and operation, and complies with the provisions of the competent ecological and environmental authorities.

The company exercises unified leadership and centralized management over the environmental protection, and the main responsible persons of the company shall be fully responsible for the environmental protection management. The company establishes an environmental protection management system composed of the leaders of various departments, environmental protection administrators, technicians and environmental protection management personnel, and sets up an EHS committee to be responsible for leading, organizing, planning and coordinating the environmental protection management work of various departments of the company.

A full-time organization and staff were also set up environmental protection management, to be responsible for the company's internal environmental protection monitoring, inspection and other management tasks. The company regularly collects and organizes environmental protection laws and regulations, publicity and education work. The company establishes responsibility system for environmental protection, including environmental protection and pollution reduction into the plan, participates in the formulation of environmental protection development plans for factories, draws up environmental protection work plans, participates in the preparation and implementation of environmental protection management plans or measures for factories,

participates in the preparation, revision and exercise of control plans for preventing sudden pollution accidents in factories and environmental protection plans for factories, and cooperates with monitoring departments in monitoring pollutants.

The company builds environmental protection facilities for waste water, waste gas, solid waste and noise treatment, discharges all pollutants up to standard after treatment, and disposes of solid waste in compliance with regulations.

Business Ethics

Since its inception, Huayou Cobalt has always upheld the highest standards of business ethics, and takes "integrity" as one of the core values of the enterprise. The company, in the course of operation, abides by the law, and publicly pledges to oppose any form of corruption, embezzlement of public funds or extortion; avoids all forms of conflicts of interest; respects intellectual property rights and protect customer information; abides by the law of the market, and resists false advertisement and unfair competition; and discloses information in accordance with the requirements of information disclosure of listed companies; the company establishes effective channels of communication to facilitate complaints and prosecutions and provide identity protection for complainants and prosecutors, and prohibits any form of retaliation; and respects the privacy of all people who do business with the company. The company has also formulated the Code of Business Ethics and Conduct, and ensures that all employees can scrupulously abide by the highest requirements of business ethics, abide by applicable laws and regulations at home and abroad, realizing self-restraint and public service, thus contributing to the centennial Huayou's everlasting inheritance.

Anti-corruption

A. Institutional culture construction

The company attaches great importance to anti-corruption and anti-commercial bribery, and, formulates Code of Business Ethics and Conduct, Administrative Measures for Business Reception and Gift Reimbursement, Gift Management Methods, Management Method For Employee Receiving Gift Money and Gifts, Reporting Management System and Regulations on Prohibition of Business Cooperation with Units with Relatives, Friends or Business Interests Related to Company Cadres in compliance with all applicable laws and regulations of the country or region in which the operation is conducted and relevant international conventions and in accordance with characteristics of its own business. The company also guide the performance of employees by opening their performance through the OA platform, requires employees to establish the awareness of honesty and discipline, implements it in all links of work, and strengthens the construction of compliance culture and business ethics.

B. Establishment of a supervision department

Huayou sets up Audit and Supervision Department and makes it as the first-level department of the group headquarters. The department directly reports to Group President, and is responsible for the overall management of the internal audit of the Group, performs audit functions of Group

subsidiaries, thus doing well in project construction, financial activities and economical responsibilities through process supervision and project recall. The Department also seeks truth from facts, and builds independent and objective image in the global subsidiaries to implement anti-corruption, anti-commercial bribery practices. It severely punishes performance violations of discipline, which not only purifies the ranks of business managers, but also timely finds management loopholes, effectively maintaining the survival and development of enterprises.

C. Preventing conflicts of interest

At present, the international community generally believes that conflict of interest is an important source of corruption, and management and prevention of conflict of interest are the key strategy to effectively prevent corruption. Therefore, Huayou requests the Audit and Supervision Department to conduct commercial bribery investigations on business partners in accordance with the Regulations on Prohibition of Business Cooperation with Units with Relatives, Friends or Business Interests Related to Company Cadres in March and September of each year. As per the requirement, all suppliers shall sign a Letter of Undertaking for No Relatives, Friends or Business Interests in order to make clear relevant requirements such as anti-commercial bribery. During the two audits in 2018, no direct or indirect participation of the company's cadres in relevant suppliers was found, and no unqualified suppliers were found; Of the 15 suppliers selected in September, 3 of them didn't sign the Letter of Undertaking for No Relatives, Friends or Business Interests and had been requested for replacement.

D. Provide access to information

Huayou has established and improved a reporting system, widening complaint channels, encouraging informed reporting of irregularities, mobilizing and giving full play to the positive role of employees and stakeholders in reporting corruption and commercial bribery, and formally promulgating the Reporting Management System to all employees through the OA platform on November 16, 2018, and providing reporting phones and e-mail addresses to all stakeholders through the official website. Audit and Supervision Department is responsible for receiving, investigating, approving and directing reports by the Group President. Reporting Management System clearly stipulates that all whistle-blowing information is strictly confidential; anonymous reporting is allowed. Information leaks, investigation obstructing and retaliation are strictly prohibited. For reporting information, the whistle-blower will be rewarded according to the value of the infringement, and the information of the whistle-blower will be kept secret. In 2018, a total of six incidents were reported, one of which was a corruption matter, and one of the person involved in the case was eventually dissuaded.

Ways of reporting:

① Reporting letter, mailing address: Audit and Supervision Department (Principal), Zhejiang Huayou Cobalt Co., Ltd., No.18 Wuzhendong Road, Tongxiang City, Zhejiang Province; ②Reporting Tel.: 0573-88589103;

③E-mail:report@huayou.com;

④ Reporting box: Unpacking once a week (except holidays), the unpacking time is 9:30-10:30 every Monday morning.

Reporting management process:

The undertaker of the Audit and Supervision Department shall record the reporting items according to the reporting information, register the Report Registration Form of Petition Letter, submit it to the person in charge of the Audit and Supervision Department for re-examination and signature, and appoint a special person to be responsible for the investigation after reporting to the president or chairman of the board of directors for approval;

The undertaker or investigation team must conduct the investigation confidentially and hear the arguments of the person to be reported, and may request the relevant department to assist in obtaining sufficient investigation evidence to form a trial report (including handling opinions);

Opinions on the handling of the reported matters shall be given within 60 days and shall be reported to the President for approval; opinions on the handling of the reported matters entrusted by the company for investigation shall be given within 30 days and shall be reported to the President for approval;

The Audit and Supervision Department shall handle matters according to relevant provisions according to the examination and approval results;

The undertaker or the investigation team shall notify the whistle-blower in writing of the result of the handling within 5 working days after the handling of the reporting is completed;

The undertaker shall file all the report materials after the processing is completed.

The Audit and Supervision Department shall maintain the same working hours as other administrative departments from 8:00 to 11:15, 13: 30-17:00, Monday to Friday, 6.75 hours a day; 8:00-11:15, 13:30-16:30 on Saturdays, 6.25 hours, guaranteed 40 hours a week.

Patent maintenance

In 2018, the company applied for 28 patents and newly authorized 15 patents (4 of which were invention patents and 11 utility model patents), bringing the total number of valid patents to 78 (46 of which were invention patents and 32 utility model patents).

In 2018, Huayou New Energy Technology (Quzhou) Co., Ltd. passed the certification of intellectual property management system.

Community Engagement

The company's investment philosophy is "no matter where you invest, you must contribute to the local economy and society". The growth and expansion of the company are closely linked with the strong support and understanding of the local community, therefore, enterprises should also be grateful and return to the local community, perform public welfare undertakings in its power for local community, and let the community grow with the enterprise.

Participating in Accurate Poverty Alleviation and Accomplishing Counterpart Support

In 2018, Huayou Cobalt undertook two precise poverty alleviation projects in Heishui County,

Sichuan Province.

Heishui County is a glorious revolutionary history of the region, but it is also a national poverty-stricken county due to geographical reasons. In the activity "ten thousand enterprises help ten thousand villages and striving for a well-off life hand in hand", Huayou Cobalt, as the only private enterprise in Tongxiang City, independently supports Luoerba Village in Zhawo Township, which is the poorest in the locality, and contributes 100,000 yuan to Luoerba Village Ecological Fruit and Vegetable Professional Cooperative, which is specially used for the project of poverty alleviation workshop.

Another activity of the company is to set up a company in Heishui to carry out project investment, in order to achieve the industrial assistance of Heishui. Company's subsidiary Huayou Recycling makes investment to Heishui, and sets Heishui Huayou Recycling Technology Co., Ltd., laying solid foundation for the follow-up development of recycling industry in Sichuan. Only 11 days are used from the establishment of the company to the delivery of RMB 70 million registered capital, making local aid cadres praised.

2018 was a year of historic significance for Huayou Cobalt to achieve a "great-leap-forward RMB 10 billion" and the company smoothly joint the Top 500 Private Enterprise Manufacturing Industries. While the enterprise grows stronger and bigger, the company concerns the society and the embodiment of social responsibility. In 2018, the headquarters of the company launched a series of charity activities, which greatly enhanced the influence of the enterprise and the cohesion of the workforce.

The main activities are as follows:

Good neighborly and friendly relations of village communities

On February 6, 2018, on the eve of the Spring Festival, under the leadership of trade union chairman Zhang Jianhong, the trade union visited and condoled with more than 10 healers of the Municipal Dermatology Hospital and 46 elderly people of the Longxiang Street Nursing Home, delivering New Year presents, wishing them a happy New Year and life. On the same day, the condolence group went to Anle Village to condolence the poor family of Zhang Jinfu in the village, and sent the New Year's consolation money and necessities of life such as rice and oil.

huayou
(Photo: Zhang Jianhong, chairman of the trade union at the group headquarters, sends condolences and money to the municipal leprosy hospital and the Longxiang nursing home for the elderly)

Heart to heart with special needy families, providing necessary assistance

Two family members of Dong Xinfeng in Xiang Hou Village of Wuzhen suffer serious illness, and write to the company leaders for medical expenses funding. The company leaders attach great

importance to this, instruct trade unions and special personnel to seriously verify, and carry out rescue if necessary. After checking, RMB50,000 directional donation was provided by the company to help the family pass through the crisis.

huayou
(Photo: Chen Xiaoming, head of the administrative department of the group headquarters, delivering RMB 50,000 Yuan to Dong Xinfeng)

Participation in the "Six Campaigns"

From March to October, in order to welcome the arrival of the work of building an advanced clean city, the company responded positively to the calling of party committees and government at higher levels on the "six campaigns". Under the unified and coordinated deployment of the co-built areas, the Party organization of the company organizes Party members and employees to carry out continuous patrol inspection and maintenance in the responsible area of the company's long-road section, and organizes voluntary garbage clean activities for 3 times. They performed nearly 100 times of road patrols, involving more than 200 people; and also carried out four civilized persuasions, involving 12 people. They also participated in the centralized road improvement in the jointly constructed community for two times from May to June.

huayou
(Photo: Party members of the Headquarters continue to carry out the rectification and maintenance activities of building an advanced clean city in the responsible sections of roads and co-built communities)

Good neighborly and friendly relations, poverty alleviation and relief

On February 10, 2018, on the eve of the Spring Festival, Quzhou Huayou Company launched a two-day visit and consolation work around the city. Lu Ximing, Assistant General Manager of the company, visited eight surrounding village committees on behalf of Quzhou Industrial Base and sent festive greetings, condole 16 families in rural areas and delivered Spring Festival greetings.

huayou
(Photo: Lu Ximing, Assistant General Manager of Huayou Quzhou Company, delivering condolences and money to poor households.)

Donations for KASAPA community

CDM donated school supplies to the nearby LA PERENITE Comprehensive School and supplied living materials to the nearby poor residents of KASAPA community. On March 6, 2018, Chinese employees of CDM General Management Department donated 1,000 notebooks, 1,000 pens and 5 soccer balls to LA PERENITE Comprehensive School; On March 26, 2018, CDM donated corn flour, which is the staple food of the local population, and cooking oil to residents of the KASAPA community.

huayou

Maintenance road，bring benefits to villagers

There is an old saying in China, "If you want to be rich, you must first build roads." Owing to economic underdevelopment condition, most roads in Congo Kinshasa are unsurfaced roads, except for the main roads. During the rainy season, the roads into the CDM and within the community are muddy and difficult to cross. To this end, the CDM repairs the JOLI SITE road and the road extending to the MOïSE market. This move was appreciated and praised by the Minister of Ministry of infrastructure, who encouraged CDM to continue its efforts to benefit the community villagers.

huayou

Dissemination of agricultural knowledge

Congo Gold is a large and rare country. Due to the scarcity of water resources, it is difficult to grow vegetables, and most crops are imported except cassava and maize, making life extremely difficult for the local population. The SHAD Farm organized training for university students on rice planting in the countryside, taught students on how to grow vegetables and rice in the current season, bringing hope to the Congolese people thirst for knowledge and food. In 2018, the head of Huayou Cobalt Agricultural Project Subsidiary Company, as the sole enterprise representative, introduced the project cooperation mode of the combination of industry, university and research institute as well as the demonstration effect of the park at the High-level Forum on South-South Cooperation in Global Agriculture, which was recognized by FAO, the Ministry of Agriculture of the DRC and other parties.

huayou

huayou

Drill the manual well, place the solar well

There are only a few wells in the local community, which are inadequate for nearby residents, and because of the long way to fetch water and the difficult living conditions of the residents, CDM drilled a manual well in the village of KAWAMA and placed a solar well in the village of KAMATETE to provide daily water for the villagers. During the acceptance of the KAMATETE well, a large number of residents gathered with water tanks, and their joy was overwhelming. In an interview with the media, the district governor and the villagers' representatives expressed their gratitude one after another.

huayou

In 2018, in order to let the leaders and all employees of the company understand and support the public welfare construction of the company, the company invited teachers from external professional institutions to conduct a series of public welfare classes at the company's headquarters. In 2018, a total of three courses were conducted, namely, Corporate Public Welfare Communication, Corporate Participation in Precision Poverty Alleviation and Operation of Corporate Public Welfare Foundation.

Mr. Zhang Jinhu, Deputy Secretary-General of Hongyi Poverty Alleviation Foundation, said: "Contact with Huayou gives me the opportunity to observe closely how a multinationally listed private enterprise practices and explores the path of social responsibility. Building responsibility competitiveness and encouraging second-time entrepreneurship have become the new goals and directions of Huayou. In the field of social responsibility, Huayou attaches great importance to the protection of employees' occupational safety risks, actively conducts research on the sustainable development of Congolese gold and cobalt mining communities, and carries out community infrastructure construction. Huayou are moving forward step by step on corporate public welfare, covering activities like subsidizing impoverished college students, performing community volunteer service of respecting the elderly, making enterprise public welfare staff propaganda, and implementing poverty alleviation in poverty-stricken areas. In the constant exploration, employees in Huayou participate in the public welfare awareness actively, and the planning and execution of enterprise public welfare projects are gradually move from fragmentation, randomization to systematization and maturity.”

Cultural Ideas

[Spirit of Huayou]　Constantly Strive to Become Stronger in Pursuit of Excellence

Constantly striving to become stronger in pursuit of excellence is an inexhaustible spirit to achieve Huayou undertaking.

As heaven maintains vigor through movements, a gentle man should constantly strive for self-perfection. It is this inexhaustible and passionate driving that inspires entrepreneurs to constantly reinvent themselves, thus achieving the status of global leader in cobalt industry.

[Corporate Vision]　Becoming a Global Leader of New Energy Li-ion Battery Materials

Huayou vows to become a global leader of new energy li-ion battery materials relying on continuous struggle of all staff and positioned in “new energy and new material industry”.

[Mission Statement]   Create customer value, lead industrial development

The career of Huayou is the persistent pursuit of Huayou people, and the common ideal of Huayou new energy li-ion battery material industry ecosystem. We solemnly declare that the mission of Huayou is: creating customer value, leading industrial development.

[Huayou Value]   Integrity Innovation Responsibility Learning Passion

Huayou people should take the spirit of constantly striving to become stronger in pursuit of excellence as the core, and the integrity, innovation, responsibility and learning as the value criterion, determined to become the striver of Huayou undertaking.

Scientific Research Platform

In recent years, Huayou was committed to build the international first-class enterprise R&D platform, to platform construction based on research work, gradually improve science and technology innovation platform, set up a headquarters institute, cutting-edge materials, new energy research institute, institute of non-ferrous, postdoctoral workstation, research office, and a complete set of testing center; In the mean time, Huayou also built Zhejiang Huayou Cobalt New Energy Battery Materials Research Key Enterprises Institute, Zhejiang Enterprise Technology Center, Zhejiang Huayou Cobalt Green Smelting Technology and New Materials Development Key Institute, Jiaxing Li-ion Battery Materials Key Laboratory and more than 10 provincial R&D innovation platforms and Huayou - CSU Joint Research Base.

At present, Huayou has completed the construction of 8000 square meters technology R&D site, 50,000 square meters of the main structure of a science and technology building, introduced field emission electron probe EPMA-8050G, field emission scanning electron microscope SIGMA HD, Rigaku X-ray diffraction instrument, Japanese IM4000 Plus ion grinding instrument, GC-MS instrument Agilent-8860+5977B and other advanced R&D/detection/analysis equipment with the total value of more than 200 million yuan, completed more than 100 small, medium and large test lines. A consolidated foundation is laid for the science and technology R&D, product development project implementation.

182.China Derry Group

China Derry International Group Co., Ltd. (hereinafter referred to as China Derry group),

registered in Hong Kong, mainly deals in real estate development, investment and development of mineral resources, international trade and logistics, financial investment, international engineering contracting, etc. Apart from mainland China, Africa is the focus of investment and
first-class service, first-class technology and first-class products to build the company's core competitiveness achievements of the best industry brand, to provide the most valuable industry services

Real Estate and Construction Engineering
International Trade and Logistics
Mineral Resources
Finance and Investment

183.Angel Yeast

Angel Yeast was founded in 1986, it originated from the China National Yeast Research Center. Angel Yeast Co., Ltd was listed on the Shanghai Stock Exchange in 2000. As one of the leading companies in the worldwide yeast industry, we are committed to the mission of developing yeast biotechnology and innovate for healthy life.

Our business activities range from baking to food taste, nutrition & health, and biotechnologies. Our products are available in more than 155 countries and regions.
We make full use of biotechnology, focusing on the research and application of yeast technology, which developed the world's leading yeast and deep-processed products.

Our products are widely used in baking, food taste, nutrition & health and biotechnologies.

We make full use of biotechnology, focusing on the research and application of yeast technology, which developed the world's leading yeast and deep-processed products.

Our products are widely used in baking, food taste, nutrition & health and biotechnologies.
The Angel Logo, Full of Power and Activity
The inspiration comes from the growth and fission of biological cells. It is shaped like a rotating cell, constantly giving birth to new life, symbolizing that Angel will keep pressing forward and growing stronger.

MISSION
Developing yeast biotechnology, innovate for healthy life.
VISION
To be an international specialized biotechnology group.
Core Values

Based on staff
Respect for talents, knowledge and creativity, and the interaction between company development and staff promotion.
Customer oriented
Focused on the market and customer concerns, and committed to creating customer value.
Technology & quality as driving force
Technological progress as the primary driving force and quality management as the guarantee.
Strict Quality Management
The quality and safety of our products is our top priority. Our commitment is to never compromise on the safety and quality, and this requires everybody to be engaged.
Angel has been very strictin terms of consumer safety and product quality.

Angel has obtained certifications including the ISO9001, ISO22000,HACCP, GMP, Kosher (OU), MUI Muslim HALAL certification, BRC certification, and SEDEX moral audit certification. Angel's testing center has been certificated by CNAS, and is the only testing center with a national laboratory accreditation in China
Our Research Institute
Angel Group adheres to the innovative research in the field of yeast and derivatives, focuses on cultivating and developing emerging biotechnology industries, concentrates advantageous resources, deeply studies and develops yeast functions, and strives to build a yeast research base with high independent R & D and innovation ability, so as to promote the high-quality development of China's yeast industry.

In 2021, the R & D complex was officially put into use. Angel re integrated the technology R & D system and set up nine technology centers, firmly taking a new step towards the global biotechnology industry.
Scientific research team

At present, the company has 640 scientific and technological personnel, including 2. State Council allowance experts, 7 provincial management experts, 12.Doctors and 163 masters.
R & D platform

It has a national enterprise technology center and postdoctoral.Scientific research workstation, national CNAs accredited laboratory.Hubei yeast engineering technology research center yeast.Function: Hubei Key Laboratory and otherhigh-level researchSend the platform.
Technological innovation

The company undertakes and completes national, provincial and ministerial scientific research projects.There are 32 projects and 46 awards for scientific and technological progress,Among them, there are 2 National Science and technology awards and provincial and ministerial science and technology awards.Excitation 29 items.
Sustainability
The Society
Angel has provided training for more than 500,000 people, who have gone on to become bakers,

confectioners and some have even started their own businesses.

As a listed company, Angel continues to improve its level of information disclosure, attaches great importance to investor relations, and fully guarantees the legitimate rights and interests of shareholders and creditors.

The Environment

We believe that good environmental protection is part of our competitiveness as a company. Therefore, we pursue sustainable development through our strict systems for environmentally friendly plants.

More of our budget is allocated every year towards waste water treatment, energy saving, and emissions reduction.

Through continuous improvement, we strive to always meet government standards and satisfy our communities.

The Economy

Actively implement the Chinese government's targeted poverty alleviation project to help disadvantaged groups.

Founded the Biotechnology Public Service Center in Yichang, which supported the development of small and medium-sized biotechnology enterprises in the region.

For years, we had hosted “The Home of Bakers” and “The Home of Winemakers” training events. Angel is the initiator and organizer of the “Angel Yeast Cup” Chinese Fermentation Food Competition, and an active promoter of Chinese traditional dim sum and its culture.

Enjoy Working at Angel

Every Employee Matters

Welcome to learn about Angel to spot the specific entries and training paths for you. Every individual’s ideas are respected, and every individual’s needs are fully valued here. Look forward to your joining. Let’s enjoy working at Angel.

Innovator in Yeast Fermentation

Founded in 1986, Angel, as one of the leading companies in the worldwide yeast industry, has focused on yeast biotechnology development for more than 30 years. We, the world’s second largest yeast company, have completed 78 national and provincial scientific research projects, and have obtained 44 scientific and technological progress awards and more than 240 authorized invention patents. We possess factories in 10 cities at home and overseas investment projects in Egypt and Russia. Our products are available in more than 155 countries and regions.

Equal development space

Angel provides equal development space for every employee. We support professional and technical personnel of high-end to rapidly grow into field experts, and provide double channels for employees' career development at the same time. Here, both workshop staff and senior management are provided specified and equal development opportunities.

Based on staff

We insist on mutual respect and trust with our employees. And the employees are the host, because everyone is equal under the policy at Angel. To grow together with employees is our goal, because employees are not only our cooperators with mutual-trust, but also our partners moving forward side by side.

AHH-Angel Human Health

AHH(Angel Human Health) is a yeast functional ingredients division of Angel. Specialized in yeast and fermentation, AHH is committed to developing innovative, differentiated, science-based functional ingredients and customized solutions, to help our customers get enduring success, as well as contribute to a healthier and sustainable world together.

Targeting a wide range of applications including immune support, gut health, weight management, sports nutrition, healthy ageing, vegetarian support and overall wellbeing, with featured products of Yeast Beta-glucan, Nutritional Yeast Flakes, Yeast Protein, Probiotics of Saccharomyces Boulardii, Selenium Yeast, etc., AHH keeps adding values to end products of many famous brands all around the world.

Health Benefit Solutions

Gut Health

Sport Nutrition

Antioxidant

Vegetarian Diets

Immunity Health

Minerals/Vitamins Fortified

184.New Hope Group Co., Ltd.

New Hope Group Co., Ltd. is a private enterprise group mainly engaged in modern agriculture and food industry. It was founded by the famous private entrepreneur Mr. Liu Yonghao in 1982. Since struggling with the tide of reform, new hope group has the second feed production capacity in the world, the first in China and the first poultry processing capacity in China. And one of the largest integrated suppliers of meat, eggs and milk in China.

In 40 years of development， new hope group is based on the modern agriculture and food industry and continues to extend to upstream and downstream industries to dairy FMCG, smart urban and rural areas, financial investment and other related industries. It has more than 600 subsidiaries around the world, more than 135000 employees, the group's asset scale exceeds RMB 300 billion, and its sales revenue exceeds RMB 240 billion in 2021, ranked 390th on t the Fortune Global 500 in 2021. New Hope is given an AAA credit rating by China Chengxin

International Credit Rating Co. Ltd

From 1994，When Mr. Liu Yonghao initiative launch "glorious cause" industrial poverty alleviation, to 2021, joined hands with the "Yonghao Charity Foundation" to launch the "Five-Five Plan" to help the National rural revitalization Strategy，new hope group has always rooted in the fields of agriculture, rural areas and farmers, focused on people's livelihood industries, and strived to be the "vanguard of Rural Revitalization". In the future, new hope group will continue to implement the mission of "Hope, Make Life Better", lead development with the concept of "five new", and strive to build an excellent enterprise with world competitiveness.

Chairman's Message

Since our foundation in 1982, New Hope Group has gone through a journey of more than three decades, following the pace of China’s economic reform and opening-up. We are known as an “evergreen tree” among Chinese private enterprises. But compared with other time-honored enterprises around the globe, we have just completed the first leg of an arduous journey.

Our journey started from agriculture, an ancient but always promising industry. The land provides us with an inexhaustible source of support, as the company developed from feed manufacturing to farming technology, food processing, channel development, facility construction and financial services. Today, New Hope Group has a broad base of operations that mainly engage in agriculture, animal husbandry and food processing, but we also operate and invest in emerging innovative industries with growth potential.

Currently, New Hope Group has a presence in more than 30 countries and regions worldwide. Meanwhile, we are accelerating the establishment of overseas regional headquarters. We are dedicated to becoming a truly international company that brings together the best global resources and delivers the best products and services to customers across the world.

The 30-year-old New Hope strives to “grow young”. Entrepreneurial passion and innovative mindset are the key to our success today and in the future. The New Hope Group fulfills its mission to promote industry and social progress based on its own growth. We actively adapt to the trend of the time, meet the demand of customers and foster continuous development always “half a step ahead”.

Growth is the ultimate beauty on the earth; hope is the utmost freshness in nature. New Hope Group shares beauty with the world!

刘永好

Liu Yonghao

Since our foundation in 1982, New Hope Group has gone through a journey of more than three decades, following the pace of China’s economic reform and opening-up. We are known as an “evergreen tree” among Chinese private enterprises. But compared with other time-honored enterprises around the globe, we have just completed the first leg of an arduous journey.

Our journey started from agriculture, an ancient but always promising industry. The land provides us with an inexhaustible source of support, as the company developed from feed manufacturing to farming technology, food processing, channel development, facility construction and financial services. Today, New Hope Group has a broad base of operations that mainly engage in agriculture, animal husbandry and food processing, but we also operate and invest in emerging innovative industries with growth potential.

Currently, New Hope Group has a presence in more than 30 countries and regions worldwide. Meanwhile, we are accelerating the establishment of overseas regional headquarters. We are dedicated to becoming a truly international company that brings together the best global resources and delivers the best products and services to customers across the world.

The 30-year-old New Hope strives to “grow young”. Entrepreneurial passion and innovative mindset are the key to our success today and in the future. The New Hope Group fulfills its mission to promote industry and social progress based on its own growth. We actively adapt to the trend of the time, meet the demand of customers and foster continuous development always “half a step ahead”.

Growth is the ultimate beauty on the earth; hope is the utmost freshness in nature. New Hope Group shares beauty with the world!

Liu Yonghao
History & Awards
Over the decades, New Hope Group has thrived through its continuous pursuit of higher goals.
Corporate Value: Illuminative, Positive, Normative, Innovative
For a long time, the Group has been committed to the beautiful vision of evergreen business.
We follow the core values “Illuminative, Positive, Normative, Innovative” to build an eco-friendly,
sustainable and world-class enterprise of food and modern agriculture.

Illuminative
An illuminative character contributes to business operation
Be simple, sincere, happy, confident and illuminative
Willing to give and contribute to collective interests
Be friendly, energetic and communicative

Positive
Promote positive thinking and integrity

"Positive mind and action bring positive results."

Normative
Be professional, dedicated, diligent and conscientious
Be honest, fair and trustworthy
Serve the customers and society

Innovative
Dedicated to product and service innovation to build core competency
Improve management ideas and practices for better performance
Innovate business models for continual growth
Industries
New Hope, New Life
Since it was founded in 1982, New Hope Group has always regarded industries as the cornerstone of its growth. Over the past three decades, New Hope Group has constantly widened the range of the industries it is involved in, which now include food and modern agriculture, dairy, real estate, and the chemical industry.
Food and Modern Agriculture
Real Estate and Infrastructure
Dairy and FMCG
Chemical Industry and Resources
Investment
One of the first Chinese private companies to invest in the financial sector
New Hope Group began to develop its financial investment platform in 1995, and was among the first private Chinese enterprises to enter the sector.
Corporate Responsibility
Fulfilling corporate social responsibility
Since the foundation in 1982, we have pursued a unique journey from zero to one, and further to a group company with annual revenues of 75 billion Yuan and 70,000 employees. "Greater size means greater responsibility." The development of New Hope Group means a lot to its shareholders, customers, employees as well as other members of society.
CSR Philosophy
"Greater size means greater responsibility." The development of New Hope means a lot to its shareholders, customers, employees as well as other members of society
New Hope Group is committed to delivering sustainable development with continual innovation to all of the communities in which it operates. A cornerstone of our values is our relationship with our environment.
For Society
All of New Hope Group’s people hold to a common set of shared values: Illuminative, Positive, Normative, Innovative, and contribute to the society through honest operation.
For Customers
New Hope Group continually focuses on the needs of its customers. From wanting to benefit farmers, to growing our partners’ businesses, and to providing consumers with safe and healthy products. New Hope Group commits to the accountability of its

For Employees

Our people are our priority. New Hope Group has more than 70,000 people spanning all facets of our operations. We want to be the employer of choice, and to provide our people with a safe, enjoyable and progressive career.

For Shareholders

We create value for shareholders. We adopt world’s best practice governance standards and manage our risks and reporting accordingly. Our people are empowered, accountable and available across the Group.

Public Welfare

Throughout the years, New Hope Group has always been a dedicated practitioner of public welfare. Social responsibility underlies our continual growth

Public welfare has always been our greatest concern since the founding of New Hope Group. From initiating the Guangcai Program,lifting poverty in rural areas, establishing the House of Philanthropy, to donating for the Wenchuan earthquake, New Hope Group has been and will always be an active contributor to public welfare.

Let’s Hope

Join us, for the beautiful, for the hopeful

A unanimous understanding of talents is the foundation of our mutual recognition.

A young and diverse team will enable you and the company to grow together.

A variety of culture-building activities will bring you to a full play.

Join us and strive for a bright future together.

Perspective of Talents

Based on a comprehensive analysis of high-end talents, we also value the following qualities, besides sound professional abilities.

Positiveness

Be positive towards your jobs

Be result-oriented

Always self-examine and keep improving yourself

Be flexible psychologically

Be positive and open-minded

Be result-oriented

Be self-motivated and willing to drive for continuous improvement

Altruism

Be willing to help others succeed for the benefits of the organization or the team

Be willing to contribute or take jobs beyond your duty

Confidence

Believe in yourself and your team

Be willing to take challenges

Be able to lead the others

Ability of Thinking

Have the capacity to learn quickly so that you can become an expert in your field within a short period of time, and learn continuously;

Ability of Learning

Be able to learn fast
Be able to become an expert in a particular field quickly
Keep on learning and trying
Ability of Communication
Be able to communicate efficiently with your superior or subordinate
Be able to express your opinions appropriately
Be able to deliver right messages
Be able to influence others by effective communication
Culture-building Activities
A dynamic and efficient team results from open and tolerant culture. In NHG, you are treated kindly no matter where you come from. We hold diversified culture-building activities, which are great catalyst for team building and triggering vitality.
A dynamic and efficient team results from open and tolerant culture. We hold diversified culture-building activities, which are great catalyst for team integration and triggering vitality.
Training
“Newbies, Leaders, Elites and Falcons” program, “Management Trainee” program, “young-and-middle-aged training” programs, “Soaring dragon” programs will meet career development needs at different levels.
Spring/Autumn Outing
Seasonal outing activities are organized for our staff to close to the nature.
Team Building
Here we have not only rigorous military exercise but also light and joyful games. They are both intended to help you mix into the NHG family faster and better.
Birthday Party
Colleagues will be gathered to celebrate the birthday person in each month. Blessings and candle lights bring you the family warmth.
Local Festivals
At any corner of the world, NHG employees can always merge into the local culture: you may celebrate Holi Festival with Indian friends, greeting the spring, and hope.
New Year Gala
When entering the world, we are entering the local life. In the meantime, we are also bringing our culture, like Hongbao, to the world.
Our Team
Wherever and whatever cultural background you come from, you are able to share the world as long as you have a young heart.Young and Positive
To realize the group’s strategy and adapt to the changes of the society nowadays, we have high expectations and demands on the young people. That’s why the group is never sparing any effort in training and using young talents. It’s not only about trust and support. It’s more about transforming and nurturing. The company is willing to put more challenges and responsibilities on the shoulder of the next generation of NHG leaders so that they can inject new vitality and energy into the 34 year old ever-flourishing company.Diverse and Comprehensive
In NHG, we have here graduates from a variety of reputable universities around the globe. We are joined by many professionals who have working experiences in Fortune 500 companies. We have also expat employees working with us in and outside China. A diverse background always

makes it possible to generate innovative concepts. In an atmosphere where individual character is respected and differences are tolerated, they are working as one and rapidly promoting their own values while creating values for the company.
Education Background
Domestic: Tsinhua Univ., Beijing Univ., Fudan Univ. etc.
Overseas: Harvard, Cornell etc.
Professional Experiences
Elites from foreign and domestic Fortune 500 enterprises and governments
Diversified Culture
Teams made up of people from various countries and regions and with diverse cultural backgrounds
185.China Gold International Resources Corp. Ltd.
China Gold International Resources Corp. Ltd. (TSX:CGG – HKEx:2099) is a Canadian based, profitable and growing gold and copper producer. The company operates two producing mines in China: the CSH Gold Mine in Inner Mongolia Region, and the Jiama Copper-Polymetallic Mine in Tibet Region. CGG is the flagship and the only overseas listing vehicle of China National Gold Group Co., Ltd. (formerly known as China National Gold Group Corporation)? which holds a 40.01% interest in the company.
BUSINESS CODE & ETHICS
We have a Code of Business Conduct and Ethics applicable to all employees, officers, directors and contract employees regardless of their position in our organization, at all times and everywhere we do business. The Code provides that our employees, officers, directors and contract employees will uphold our commitment to a culture of honesty, integrity and accountability and that we require the highest standards of professional and ethical conduct from our employees, officers, directors and contract employees.

To view a PDF of China Gold International Resources Corp. Ltd. Code of Business Conduct and Ethics, click here.
CEO'S MESSAGE
March, 2019
We bid farewell to 2018 with full of challenges and steady development.

In 2018, we overcame significant foreign exchange fluctuations, saw a decrease in product prices and achieved profitability in mine operations for the 11th consecutive year. The Company also almost reached a pre-tax net profit, which was adversely effected by the depreciation of RMB exchange rates causing a realized foreign exchange loss of US$15.82 million. The Jiama Mine’s Phase II expansion achieved full commercial production, which resulted in copper production reaching a record high and production capacity to continually increase. The CSH Mine completed the optimization of pit wall slope parameters, achieving pit wall stabilization and sustainable development. The CSH Mine is currently working on a study of inferred resources development.

We enter 2019 with hope and progress.

In 2019, we will continue to put more effort on production and operation at our Jiama Mine and

CSH Mine. We will continue to improve the efficiency of our equipment and increase our recovery rate, maintaining the year-on-year growth of performance through increased production capacity records of copper. We will strengthen our efforts on resource exploration and environmental protection at the two mines and fully promote the sustainable development of the company. We are hopeful and optimistic about potential M&A opportunities. We will carefully evaluate potential projects and strive to inject new assets as soon as possible. Thanks to the support of shareholders and directors!

Thanks to all the members of the team for their contribution! With your strong support, and with the hard work and dedication of all employees, we will continue to move forward by successfully completing the goals of 2019 and creating better all around performance.

Liangyou Jiang

Chief Executive Officer

MISSION STATEMENT

As the overseas flagship company of China National Gold Group Corporation, the vision of China Gold International Resources Corp. Ltd. is to become a leading gold and by-product non-ferrous mining company by producing quality reserves from existing mines, updating and discovering new resources through exploration rights and selectively acquiring additional high quality assets. China Gold International Resources Corp. Ltd. is committed to achieving maximum shareholder value through growth, stability, low cost and responsible operations.

CORPORATE CITIZENSHIP

The Company believes that maintaining high standards in environmental protection and cultural sensitivity is critical to its long-term success. The Company has adopted advanced technologies such as the recycling and reuse of water in the flotation process as well as dry heaping of tailings at the Jiama Mine to minimize the impact of its operations on the environment. The Company has also made substantial efforts to integrate with the local population in the areas where its mines are located and assisted them in advancing social and economic development. Such efforts are recognized by the local government and population in Inner Mongolia and Tibet, both of which are autonomous regions with a large minority ethnic population. The Company intends to continue to undertake best international environmental and cultural practices.

186.ICBC

Industrial and Commercial Bank of China was established on 1 January 1984. On 28 October 2005, the Bank was wholly restructured to a joint-stock limited company. On 27 October 2006, the Bank was successfully listed on both Shanghai Stock Exchange and The Stock Exchange of Hong Kong Limited.

Corporate Culture

Our Mission

Excellence for You.

——Excellent services to clients

——Maximum returns to shareholders

——Real success for our people

——Great contribution to society

Our Vision

Build a world-class and modern financial enterprise with global competitiveness by adhering to the principles of "delivering excellence, sticking to our founding mission, customers' favourite, leading in innovation, security and prudence, and people-oriented"

Our Values

Integrity Leads to Prosperity.

——Integrity, Humanity, Prudence, Innovation, and Excellence.

Corporate Strategy

Industrial and Commercial Bank of China (“ICBC” or the “Bank”) places a high value on the formulation and implementation of its development strategy and has adhered to the strategy in its business development. Business transformation is the core strategy of the Bank. Since the joint-stock reform, the Bank has developed and implemented four three-year development plans centering around this core strategy and made remarkable achievements, proving that the transformation strategy complied with the economic and financial development trend and customer demands, conformed to the Bank's actual operation and development, and thereby was correct and effective. 2017 is the last year of the Bank's fourth three-year development strategic plan (2015-2017) and the initial year of its fifth three-year plan (2018-2020). The Bank will keep implementing the guidelines for transformation, focus on the in-depth transformation of business mode, improve the core competitiveness and push forward the sound and sustainable development of all businesses.

I. ICBC's main achievements in business transformation

Seizing the historic opportunities arising from the transformation of domestic economic development mode after the joint-stock reform, the Bank strived to overcome the adverse impact of international financial crisis, firmly promoted structural adjustment and development mode transformation, and preliminarily built an intensive and sustainable development path featuring balanced assets and capital, quality and benefits, and cost and efficiency. The Bank did a good job in structural adjustment and development mode transformation, and built a sustainable operation framework. It promoted the transformation of system, mechanism and process in depth and built a sound management framework and foundation for business transformation and development. It also made new breakthroughs in internationalized and integrated operations and kept improving its cross-border and cross-market service capability. In addition, the Bank improved significantly its competitiveness, demonstrating comprehensive competitive advantages among domestic banks, and ranked atop among international advanced banks in terms of main competitiveness indexes, showcasing constantly growing market influence.

II. The Strategic Framework of ICBC's New 3-year Plan

As the economic and financial environment at home and abroad will still be full of uncertainties and complexities for some time, the Bank will, based on the strategy for the new period, rely on

the real economy, focus on stable quality, structural adjustment, pursuit of innovation and transformation promotion, and guarantee the constant quality improvement and efficient development of the Bank by sizing up the situation and taking active efforts. First, the Bank will implement the three major projects (reconstruction of credit management foundation, comprehensive disposition of non-performing loans and improvement of enterprise risk management) and improve the quality of transformation; second, the Bank will implement four major structural adjustments in terms of assets, liabilities, earnings and channels, and build a new operation framework matching with the new market and new type of business; third, the Bank will implement innovative transformation in five fields, namely, IT-based banking, retail banking, corporate banking, mega asset management & integration, and internationalization, and build the strategic foundation for stable profit growth and core competition advantage expanding against the background of new normal and interest rate liberalization; fourth, the Bank will deepen the institutional mechanism reform and lay a solid management foundation for transformation and development.

The Bank believes that the new three-year plan will serve as the strategic guidance and driver for its future operational development, and, following the plan, the Bank will promote its transformation and development to a new level.
Awards & Rating
Outstanding Organizer of "Mach 15" Campaigns
2022 Big Data Industry Development Pilot Demonstration Project
Best CNY/RUB Market Maker
Products & Services
Personal Banking
Corporate Banking
E-Banking
Bank Card
Corporate Social Responsibility
The report consists of eight chapters: Governance and Sustainable Risk Management, Sustainable Finance of Domestic Institutions, Green Finance, Domestic Inclusive Finance, Privacy and Data Security of Domestic Institutions, Protection of Lawful Rights and Interests of Domestic Financial Consumers, Human Resources Development, and ESG Performance
Green Finance

ICBC 2023 One-Belt-One-Road Green Bond Pre-Issuance Stage Green&Sustainable Finance Certificate（HKQAA)

ICBC 2023 One-Belt-One-Road Green Bond Third Party Verification Statement（Beijing Zhongcai Green Financing Consultant Ltd.)

External Reviews & Opinion on ICBC 2023 One-Belt-One-Road Green Bond（Beijing Zhongcai Green Financing Consultant Ltd.)

Industrial and Commercial Bank of China Limited 2022 Green Bond Report

Third-party Assessment for Industrial and Commercial Bank of China Limited 2022 Green Bond Report

2022 Special Report on Green Finance (TCFD)

ICBC 2023 Carbon Neutrality Green Bond Pre-Issuance Stage Green and Sustainable Finance Certificate（HKQAA）

Industrial and Commercial Bank of China Limited 2021 Green Bond Report

Industrial and Commercial Bank of China Limited 2021 Green Bond Report Third-party Assessment

Special Report on Green Finance (TCFD) （2021）

ICBC 2022 Carbon Neutrality Green Bond Pre-Issuance Stage Green and Sustainable Finance Certificate（HKQAA）

Training Development

Success can be achieved only by many a talented people. Industrial and Commercial Bank of China has always attached great importance to talent training. It built ICBC College, a corporate college for the new era, by integrating the Group's learning channels, methods and contents to serve as a big platform for learning, knowledge sharing and ideological innovation of more than 400,000 employees in 17,000 ICBC institutions worldwide, with a view to continuously output innovative thoughts, interdisciplinary talents and ICBC wisdom to boost the sustainable development of the bank.

As a corporate college of the financial institution under the central government, ICBC college has always given top priority to fulfilling its mission and responsibilities as a large-sized state-owned bank. Adhering to the principles of "opening-up, diversification, integration and win-win", the bank established internally a learning-oriented organization to cultivate talents and invigorate ICBC's undertaking, and built externally an intelligent platform to boost the national economy and the people's livelihood and fulfill its social responsibilities.

To follow the fundamental principle of serving strategies and cultivating employees, ICBC College has facilitated the Group's strategies and employee career growth, making overall arrangements for position adaptability training for all employees and the training for key personnel in the key areas, promoting the training for local employees, cross-border talents and customers in a coordinated manner. It also established a three-dimensional training system, rooted at development and covering all positions at all levels and the whole career cycle, to precisely boost business development and cultivate high-quality talents.

With a tangible, boundless, efficient and empowering personnel cultivation pattern, ICBC College, with more than 40 physical campuses across China, has also built a convenient and efficient

online learning platform, as well as a qualification certification mechanism to encourage various types of employees at all levels to keep growing. A training support system (such as practical program, text book, case, course and trainer) and an innovative learning mechanism featuring "smart, personalized and social networking community", are set up to create a sound ecosystem for all employees to enjoy learning, and a community for knowledge sharing.

Bearing the responsibilities of serving the society and improving people's livelihood, ICBC College, actively catering to national policies and people's demand, carried out a series of "training +" programs for senior executives of private enterprises, micro and small enterprises and private banking customers through smart learning, such as accompanying learning and public-welfare live video streaming. "Macau Young Financial Talent Cultivation Academy" has been established to promote the development of corporate-education integration enterprises and provide ICBC schemes to serve the real economy, national economy and people's livelihood.

Focused on the future development of integration and co-building, ICBC College, by centering on the state's regional development strategy, will build regional academies, such as Beijing-Tianjin-Hebei Future Academy and Yangtze River Delta Science and Technology Innovation Academy. Focusing on the key areas of financial development, it will specially build colleges on inclusive finance and risk management. To serve the society, distinctive academies including Xingchen Academy and Xinghuo School, will be built for school teachers and students. Leveraging on the advantages arising from global presence, it will extensively cooperate with well-known universities at home and abroad on scientific research on training, social service and cultural exchange, to gather and incubate core forces leading future development.

187.SINOVAC

Sinovac Biotech Ltd. (SINOVAC) is a China-based biopharmaceutical company that focuses on the R&D, manufacturing, and commercialization of vaccines that protect against human infectious diseases.

SINOVAC's product portfolio includes vaccines against COVID-19, enterovirus 71 (EV71) infected Hand-Foot-Mouth disease (HFMD), hepatitis A, varicella, influenza, poliomyelitis, pneumococcal disease, and mumps.

The COVID-19 vaccine, CoronaVac®, has been approved for use in more than 60 countries and regions worldwide. The hepatitis A vaccine, Healive®, passed WHO prequalification requirements in 2017. The EV71 vaccine, Inlive®, is an innovative vaccine under "Category 1 Preventative Biological Products" and was commercialized in China in 2016. In 2022, SINOVAC's Sabin-strain inactivated polio vaccine (sIPV) and varicella vaccine were prequalified by the WHO.

SINOVAC was the first company to be granted approval for its H1N1 influenza vaccine Panflu.1®, which has supplied the Chinese government's vaccination campaign and stockpiling program. The Company is also the only supplier of the H5N1 pandemic influenza vaccine, Panflu®, to the Chinese government stockpiling program.

SINOVAC continually dedicates itself to new vaccine R&D, with more combination vaccine products in its pipeline, and constantly explores global market opportunities. SINOVAC plans to conduct more extensive and in-depth trade and cooperation with additional countries, and business and industry organizations.

Mission

Supply vaccines to eliminate human diseases.

Innovation

Platform

SINOVAC is committed to developing and innovating cutting-edge vaccines to meet the challenge of preventing human diseases worldwide. SINOVAC has mastered multiple core vaccine technologies and boasts an R&D system made up of seven diverse technology platforms that fuel product development for viral and bacterial vaccines, attenuated live vaccines, recombinant protein vaccines, antibodies, and mRNA vaccines. These platforms are supported by world-class facilities that carry out advanced molecular and cellular biology, immunology, and biochemical analysis, as well as large-scale, animal-based research. Our strong interdisciplinary research team includes experts in pharmacy, biology, medicine, and chemistry, who support a robust capacity for research, development, and innovation.

Pipeline

SINOVAC upholds a diversified product pipeline and a continuous process of innovation to support its aims to develop safer and more effective vaccines that provide protection from disease for all people, everywhere. SINOVAC's vaccine products and R&D pipeline cover a range of infectious and major diseases, including but not limited to vaccines for hand, foot and mouth disease, meningitis, pertussis, pneumococcus, rabies, and rotavirus; as well as multiple vaccines under development with major clinical trials.

Scientific Achievements

SINOVAC has undertaken nearly 60 national and regional science and technology R&D projects; received two State Scientific and Technology Progress Awards and three national-level institutional technology awards; and published more than 140 SCI papers, many of which were published in top academic journals including New England Journal of Medicine, The Lancet, Science and Nature, etc. SINOVAC has obtained more than 80 invention patents for its core technologies in China.

Products

COVID-19 Vaccine (Vero Cell),lnactivated - CoronaVac

Influenza Vaccine Product Family

Enterovirus Type 71 Vaccine (Vero cell),Inactivated - Inlive

International Business

With the mission to "supply vaccines to eliminate human diseases", SINOVAC has developed a comprehensive product portfolio vaccinating against traditional endemics such as hepatitis A, seasonal influenza, pneumococcal disease, poliomyelitis, varicella, and mumps; as well as emerging pandemics, such as COVID-19, enterovirus71 (EV71) infected Hand-Foot-Mouth disease (HFMD), H5N1 pandemic influenza (avian flu), H1N1 influenza (swine flu) and SARS.

Based on its comprehensive quality management system, SINOVAC has four vaccines approved by the World Health Organization ("WHO"). Among which, its hepatitis A vaccine Healive®, Sabin-strain inactivated polio vaccine (sIPV) and varicella vaccine were granted with WHO prequalification; and its COVID-19 vaccine CoronaVac® was validated by the WHO under the Emergency Use Listing ("EUL") Procedure.

Up to now, with more than 3 billion doses shipped globally, SINOVAC's vaccines have been used in more than 80 countries and regions, providing protection for a larger number of populations.

Quality Management System

The Company is developing several new products including combination vaccines and multivalent vaccines. SINOVAC primarily sells its vaccines in China, while also exploring growth opportunities in international markets.

The company has established a quality management system covering the entire product life cycle from drug research and development, technology transfer, commercial production to product termination in accordance with China GMP, WHO GMP, and ICH Q10 guidelines. Risk management is fully implemented at each stage to effectively ensure the effective operation and continuous improvement of the quality system.

We have a GMP production workshop designed and constructed in accordance with international standards and GMP requirements, utilizing the advanced facilities and equipment at home and abroad, strictly in accordance with China's current "Pharmaceutical Production Quality Management Regulations" requirements to organize production and verification, and continue to adopt WHO GMP, EU GMP, American cGMP advanced management concept to ensure that the entire production process meets domestic and international GMP requirements, thereby ensuring the quality of human vaccines.

Talent Development

Talent Development

SINOVAC continuously motivates and develops outstanding talents through the personnel training and development support system, encouraging employees to pursue diversified careers, persistently improving employee achievements and adding value to social contribution.

Talent Strategy

Gather talents with the development vision of an international group company and excellent corporate culture

Provide innovative and challenging job opportunities for employees to select talents

Attract and retain talents with industry-leading salary and benefits

Talent Concept

Attract talents and make them success with career ； Make company and company development success with excellent talents.

SINOVAC Academy

Sinovac Academy has established a "dual channel" career development system for staff management and professional development. Through various courses and training, it strives to become a growth consultant for employees and a development partner for business teams.

Sinovac Academy teaches culture, strategy, leadership, professional skills and general education courses for employees through platforms and projects such as school of management, School of Technical, high-end talent integration program and Ivy cultivation project.

Social Responsibilities

We have been actively fulfilling our social responsibility and helping to promote scientific research in the field of biomedicine.

Corporate Social Responsibility (CSR) is one of the driving forces for sustainable development of our company. We always attach great importance to social responsibility work, actively undertake corporate social responsibility, deliver healthy ideas and products to thousands of households, support the global unmet needs of disease prevention and control, promote the development of public health with practical actions, and work together with all sectors for a healthier world.

While repaying the country and society with high-quality vaccines and guarding people's health, Kexing has always been actively fulfilling its social responsibility, helping to promote scientific research and health science propaganda in the field of biomedicine, and carrying out various donations, including vaccines, to support the development of public health with practical actions and join hands with all circles to work together for a healthier world. Since its establishment, Kexing has donated more than 8 billion yuan to public health and society, including vaccine donations, material donations and construction donations.

188.GOODDOCTOR GROUP

Large enterprise groups with comprehensive development of the "big health" industry centered on biopharmaceuticals

After more than 30 years of development, Good Doctor Group has become a large-scale enterprise group that integrates science, industry and trade, and has comprehensively developed the "big health" industry with biomedicine as its core. The group's business is distributed in the seven major sectors of pharmaceutical industry, pharmaceutical business, pharmaceutical research and development, medical treatment, traditional Chinese medicinal material breeding base, modern agricultural product processing, and general health. It has 37 wholly-owned subsidiaries, 1 Internet medical platform company, and 3 medical trade companies. They are distributed in Sichuan, Yunnan, Shanxi, Liaoning, Guizhou and other provinces, with more than 20,000 employees.

Adhering to the corporate philosophy of "Being a good person and making good medicine", Good Doctor Group takes innovation as its development strategy, and has successively established a national enterprise technology center, a provincial enterprise technology center, a Sichuan provincial key laboratory for medicinal Periplaneta americana, and a Sichuan provincial medicinal animal engineering technology research center; the group has 4 high-tech enterprises and 1 national key leading enterprise for agricultural industrialization; it has established the first provincial-level academician (expert) workstation and a national-level post-doctoral research workstation in the Sanzhou area; it has cooperated with Shanghai Jiaotong University, Zhejiang University, Sichuan University, Macau University of Science and Technology, and Chengdu University of Traditional Chinese Medicine. , Wenzhou Medical University, Southwest University for Nationalities, Dali University, Kunming University of Science and Technology and more than 20 domestic university research institutes have established cooperation in production, learning and research, and have won more than 70 national, provincial and ministerial-level R&D and innovation projects.

Good Doctor Group has been listed among the "Top 100 Enterprises in China's Pharmaceutical Industry" by the Ministry of Industry and Information Technology for 11 consecutive years. The good doctor brand is "China Famous Trademark", "China Famous Brand", "Chinese Pharmaceutical Brand", "Sichuan Famous Brand", "Sichuan Innovative Brand" and "Sichuan Name Card".

"Be a good person, make good medicine, and care for the public's health." Good Doctor Group is well aware of its mission. We don't say those rhetoric, just try to abide by and practice the most unpretentious rules, be a good person with a conscience, make good medicine with high efficacy, and bring real health to the public. It was in the past, it is now, and it will be even more in the future!

CORPORATE CULTURE

For ten years, enterprises rely on management, and for a hundred years, enterprises rely on culture. Corporate culture is the gene of enterprise development, determining the "personality" and development direction of the enterprise.

Geng Funeng, the chairman of Good Doctor Group, believes that corporate culture condenses the spirit of the enterprise, starting from the top leader of the enterprise to every member of the enterprise. The style, habits, and impression of doing things ultimately form a brand reputation. If an enterprise does not have a good brand, it will not have a good reputation, nor a good spirit,

and will not go far. Therefore, it must shape the core culture and values of the enterprise.
ENTERPRISE IDEA
Be a good man, make good drugs
As a pharmaceutical enterprise, we deeply understand our mission and the importance of what we are undertaking.
We shall not make any heroic utterance and shall only try our best to comply with and execute the plainest rules and be a conscientious good man, make good drugs and bring health to people in a down-to-earth manner.
Be a good man, make good drugs.
We were, we are, and we'll better be......
Good Doctor Pharmaceutical Group Co., Ltd.
Gooddoctor Pharmaceutical Group Co., Ltd. - a modern medicine and health care product production base integrating chemical medicine preparations, traditional Chinese patent medicines and simple preparations preparations and Chinese herbal decoction pieces.

In 1998, the Group acquired the Anzhou District Branch of the former Mianyang Pharmaceutical Factory, and renamed it "Sichuan Gooddoctor Pharmaceutical Group Co., Ltd.". In September 2018, "Sichuan Gooddoctor Pharmaceutical Group Co., Ltd." was renamed "Gooddoctor Pharmaceutical Group Co., Ltd.". After expansion, the company has three factory areas covering an area of over 200 acres, with 7 dosage forms and 14 production lines, making it a large-scale comprehensive pharmaceutical base in China. There are mainly 10 brand products, including "Zunlao" Sangjiang Ganmao Tablets, "Zuzongbao" Xiaoer Jiebiao Granules, "Good Doctor" Amoxicillin Capsules, and "Good Doctor Qingke" Compound Aminophenol Alkane Glue. As a national level enterprise technology center and national high-tech enterprise, the base has undertaken more than 30 national and provincial level major special projects, won 4 provincial and ministerial level science and technology awards, and participated in the formulation of 1 international standard.

Gooddoctor Pharmaceutical Group Co., Ltd. has been awarded "AAA Credit Rating Enterprise", "Sichuan Technological Innovation Demonstration Enterprise", "Sichuan Innovative Enterprise", "Sichuan Integrity Demonstration Enterprise", "National Customer Satisfaction Enterprise", "National Intellectual Property Demonstration Enterprise", "The First Quality Government Award of Anzhou District", "Sichuan Excellent Private Enterprise", "National May Day Labor Award" and other awards.

Product Center
Brand Product
Health Product
Exclusive Variety
Marketing Network
Good Doctor Group has established 2600 county-level brand service centers and over 2000 county-level brand promotion stations nationwide, covering over 80% of counties in China.
Good Doctor Kangfuxin Solution, Children Kanggan Granules, Sangjiang Ganmao Capsules/Tablets, Compound Chenxiang Stomach Tablets, Children Jiebiao Granules, Good Doctor Compound Clam

Green Injection, Good Doctor Yumai Kouyan Mixture, Yiqi Phenoxymethylpenicillin Tablets, Yiqi Qiangqiang Combination (five dimensional glucose), Qinggong Longdan (Haima Duobian Pill), Qinggong Xiongdan Jiuxin Pills, Minqi Azlastine Hydrochloride, Good Doctor Gutongling Tincture and other high-quality drugs are sold throughout the country, Europe, America Foreign markets such as Russia and Southeast Asia.
37 subsidiaries
Wholly owned subsidiary
14 factories
GMP production plant
2 bases
GAP Medicinal Base
2600
Brand Service Centers
20000 + people
Active employees
CIRCULATE
Good Doctor Group has established a modern pharmaceutical logistics system with Canon Da Medical Trade as the main body, radiating to regions such as Yunnan, Guizhou, Sichuan, Chongqing, and Xinjiang. In recent years, the group has integrated intelligent technology, accelerated the construction and industrial upgrading of modern pharmaceutical commercial circulation systems guided by information digitization and warehousing intensification, continuously improved standardization and specialization capabilities, created efficient operation, and provided high-quality services for the upstream and downstream of the industrial chain. Good doctors have fully upgraded the modern intelligent logistics centers of Yunnan Canonda Pharmaceutical and Liangshan Canonda Pharmaceutical. In 2022, the Good Doctor Automated Intelligent Warehousing and Logistics Center located in the West High tech Zone of Chengdu will be fully operational, providing product circulation services for hundreds of thousands of medical institutions, chain pharmacies, and grassroots medical units nationwide.
189.China Kingho
Devoted to coal mining, washing and dressing, coking, by-products deep processing, coal for natural gas production, and coal- methanol-hydrocarbon production, China Kingho Energy Group Co., Ltd. (hereinafter referred to as China Kingho) is an international modern resources, energy and chemical company integrating metal ores mining and dressing, metallurgical chemical, refined coal chemical, building materials, logistics, hoteling and international trade. Headquartered in Beijing, China Kingho has 50-plus wholly-owned and controlled subsidiaries including Inner Mongolia Kingho Group Co., Ltd., Qinghai Kingho Metallurgy & Coal Chemical Group Co., Ltd., Ningxia Kingho Coal Chemical Group Co., Ltd., Xinjiang Kingho Energy Group Co., Ltd., Kingho International Trade Co., Ltd., Mozambique Kingho Investment Co., Ltd., Sierra Leone Kingho Investment Co., Ltd., Mongolia Kingho Energy Co., Ltd., Hong Kong Kingho Investment Co., Ltd., to name but a few. As of the end of 2011, the number of its on-the-job employees reached over 20,000; its total assets were valued at nearly 40 billion Yuan; its annual output value hit over 15 billion Yuan.

China Kingho focuses on the orderly development of resources and clean energy utilization,

resolutely abides by the national energy conservancy and emission reduction laws and regulations, industrial planning and policies, actively develops low-carbon and circular economy, and persistently performs the social responsibility of balancing economic, social and environmental benefits. It has ranked among the top 100 coal companies of China and the top 500 private companies of China for many years in a row.

To take root in the west, go global and build a world-class ecological energy enterprise is a lofty mission of China Kingho. In the northwest China, regardless of extremely harsh natural conditions, Kingho’s staff, in the spirit of endurance, aggressiveness, unity and sacrifice, realized the group’s incredible leapfrog development. In the tide of the shift of China’s economic growth mode, Kingho’s staff seized the opportunities and took “proper use of resources, green development” as their duties. They successively established the circular economy industrial parks in Inner Mogolia, Qinghai, Ningxia and Xinjiang, thereby producing a development characterized by “Circular business, resources sustainable, environment friendly”. On the basis of these achievements, China Kingho carried out the diversified development and “Going Global” strategies, developing new business steadily and tapping overseas markets actively. Thus far, it has become a multi-industry and multinational company and has made presence in domestic and overseas markets.

To make contributions to society, advance the harmonious development and promote regional economic prosperity and social stability is the principle that can make China Kingho get a foothold in its industrial sector. The Group has been always attached importance tothe harmonious relationship between its profits and the development of local economy and took initiative to incorporate the establishment of the circular economy industrial parks into the overall plan for the development of the regional economy, making great contributions to the long-run development of regional economy. Furthermore, China Kingho is passionate to public welfare programs, such as infrastructure construction, education, medical care, poverty alleviation, disaster relief, etc. By the end of 2011, its directly invested and donated funds had exceeded 500 million Yuan.

Practicing innovative management, Acceleraing transformation and implementing the “Giant Kingho” strategy is a fresh start of China Kingho. The group is further accelerating the transformation from a resource-based company into a deep processing and high value-added company, from the industrial operation to combination of industrial operation and capital operation, from regional management to group management, from localization to internationalization, constantly optimizing the industrial structure, pushing forward transformation and upgrading of companies, and sparing no effort to building a large multinational resources and energy company.
In the future, adhering to the idea of social commitment, honesty and trustworthiness, innovation, harmonious development and relying on the resources advantage, China Kingho will strive to develop the coal-based cogeneration circular economy, extend the conventional industry chain and accelerate the exploration and development of unconventional oil and gas resources, keep forging ahead and make a great contribution to the rapid and sound development of the Chinese economy to achieve the goal of building a green, harmonious, creditable and

internationalized Kingho.

Chairman's Statement

As the old saying goes, a journey of a thousand miles begins with a single step. Nowadays, in the time of "leapfrog development" for China, bathing with the spring breeze of a new wave of reform, China Kingho Group further emancipates its mind and seeks innovation. With the support of technological progress and talent development, China Kingho Group regards improvement of self-development as a core, fears no hardship and strives to move up, focuses on the important period of strategic opportunities, and steadily promotes a healthy and sustainable development for special advantage industries.

China Kingho Group always persists in the scientific outlook on development and guidance development of market economy, follows the overall national economic development plan, adheres to the phycology of green energy, clean production and circular economy, chooses the projects that are contributive to the economic development of countries and regions in a long term, and continue to optimize our industrial structure across China and even the globe, and strives to build Kingho into a leading energy company that is influential not only in China but also in the world.

Looking forward to the future, China Kingho Group will continue carrying out the industrial policy of the state and adhering to the concept of social commitment, honesty and trustworthiness, innovation, harmonious development to make due contributions to national and global economic development.

Industries

Recycling Industry

Clean Energy

Businesses

Product Display

Research

Culture

Group Philosophy

Corporate mission:

Committed to Green Energy, Dedicated to Social Development.

Corporate vision:

Becoming a world first-class clean energy enterprise.

Corporate values:

Pursuing enterprise values, promoting social values and realizing personal value of employees.

Corporate motto:

Virtue, honesty and faithfulness, innovation and harmonious development.

Enterprising spirit:

Endurance, Aggressiveness, Tolerance, Solidarity and Dedication.
Development philosophy:

Robust, leap-over， innovative, harmonious.
Management philosophy:

People-oriented, strict and accurate, scientific and efficient.
Operation philosophy:

Honesty and creditworthiness, mutual benefit and win-win result
Integrity Philosophy:

Indifferent to fame and wealth, cautious in power and dedicated to career.
Talent development philosophy:

Recruiting talents from around the world and fostering Kingho people.

Quality policy and connotation:

Pursuing green development and creating outstanding brand

Goals of corporate value:

Modern, professional, friendly and leading.

Modern - Modern in ideas, operation and management.

Professional - Professional in technology, teamwork and operation.

Friendly - Friendly working environment, partners and environment.

Leading -Leading in the industry, at home and abroad.

Environment protection philosophy:

Respecting nature through rational development, conforming to nature through circular utilization and protecting nature through energy conservation and emission reduction.

Safety philosophy:

Safety is the root of life, origin of happiness and basis for benefit.

Responsibility

Commitments

During economic development, China Kingho will always adhere to and implement the national energy saving and related industrial policies, and will always be oriented to the overall planning on national energy from the "12th FYP", based on the core philosophy of "ideal resource utility and green development" to firstly focus on corporate social responsibilities, emphasize the harmony and unity of economic, social, environmental benefits, actively practice the national advocacy of a low-carbon economy, continue to promote regional economic prosperity and social stability, and contribute to the construction of a harmonious society.

Charities

"Concern about the public interests, return to the community" is a traditional virtue of the Chinese nation, and also a social responsibility China Kingho has consistently adhered to. Over the years, while striving for economic development, the Group has been vigorously supporting the public welfare, taking the initiative to strengthen communication and exchanges with the public welfare institutions, actively involving in China's infrastructure construction, education, health care, poverty relief, and other public welfare undertakings.

In accordance with the principle of "social care, love dedication, help the poor and be charitable ", China Kingho has taken effective measures to solidly carry out poverty alleviation and public donation further, constantly sent loves and hopes to poor students, the disabled, the needy people and disaster areas. Over the years, China Kingho has been directly subsidizing communities and individuals via various forms. The total subsidy has exceeded 500 million yuan.

Low-carbon

Since its inception, China Kingho has been in strict compliance with the relevant policies and regulations of the national environmental protection, focusing on the enhancement of environmental protection in the operation area and its surrounding areas during production and management processes, and strengthening the environmental impact assessment of major construction projects to seek to minimize the environmental impacts. Meanwhile it has been actively promoting the research and application of environmental technologies, adhering to the absorption and application of domestic and foreign advanced energy, new ideas and new methods of environmental management, and exporting the existing management models and technologies to gain high recognition from the communities.

Making "high-carbon economy, low-carbon operation" as a breakthrough and "safe, green and environment-friendly development" the corporate mission, China Kingho focuses on the coordinated development of the economy and the environment in order to create a "green Kingho" and make environmental protection as a top priority in the Group's development. By the

end of 2010, the income of China Kingho that has been directly used for environmental protection is more than 2 billion yuan, mainly for the treatment of coal yard dust, fugitive emission of coke oven gas, production and domestic wastewater treatment and green plant projects, etc.

Worldwide

Partnership Idea

Having struggled for more than ten years, China Kingho Group sizes up the situation, takes a long-term view and move forward to create a large inter-regional, multi-industrial and international energy enterprise. It formulates and firmly pushes forward internationalization strategy by using its experienced resource development and mature industrial chain construction of recycling economy. The company speeds up overseas strategic layout, carries forward investment and development of global mineral resources and construction and operation of coal chemical industry economy park.

Looking to the future, China Kingho Group responds positively to the national strategy of "going out" and takes the idea of "Integrated Culture, Mutual Reciprocity and Mutual Benefit, Honesty and Credit, Harmonious Development" in the principle of "High Starting Point Planning, High Standard Construction, High Level Management, High Quality Development". By using the mature operation pattern of "Resource Development and Infrastructure Construction" under the precondition of comprehensive development and utilization of resources, the company roots in invested areas, creates job chance for the local, invests in infrastructure construction, improves living environment, stimulates regional economic growth. The company always adheres to sustainable development and industrialization to realize the overseas development target of forging "Green Kingho, Harmonious Kingho, Honest Kingho and International Kingho ".

Cooperation scope

As a leading business in domestic resource development and recycling economy, Kingho Group has set up several large-scale recycling economy parks at home and abroad based on resource development. The group advocates and implements the idea of recycling economy to change the original exploitation in extensive form. It uncovers untapped value of resources through the industrial chain circulation, improves utilization efficiency, and realizes sustainable and environmental goal.

Mongolia: In reply to the call of the country, China Kingho Group took the lead to going out and successfully developed of Naryn Sukhait coal mine in 2003, which was really a leading card among private companies in terms of large scale resource development in foreign countries, as well as a good example in implementation of "going out" and "north open" strategy for Inner Mongolia autonomous region and the whole country. Mongolia cooperation projects have not only fueled the local economy, but also greatly improved the local infrastructure and public utilities, thus the company has won recognition and praise from Mongolia government and the locals.

Mozambique: The ministry of commerce of China issued “Certificate of Overseas Investment” to Kingho Group, which also announced the foundation of “Mozambique Kingho Investment co., Ltd.” . In the principle of "Promoting Infrastructure Construction and Social and Economic

Development", Kingho Group negotiates with Mozambique government departments, and the both party entered a framework agreement. The company made investigations on Mozambique agriculture, railway, port, channel, water conservancy and other areas and proposed preliminary construction planning.

Sierra Leone: The ministry of commerce of China issued "Certificate of Overseas Investment" to Kingho Group, which also announced the foundation of "Sierra Leone Kingho Investment co., Ltd.". Kingho Group and Sierra Leone government departments explored cooperation. Finally, the company won the exploration right in several areas within Sierra Leone through hard work.

Projects

The successful operation of resource and energy cooperation projects between China Kingho Group and Mongolia companies marks the start of Overseas Strategy of China Kingho Group and becomes a model of international resources energy cooperation for the two countries.

Below are the cooperative projects:

1. Developing Naryn Sukhait coal mine and Taopu Tolgol Ovoot TolgoiOpen coal mine together with Mongolia companies;

2. Investing in construction of an 80km coal road from Ceke port to Naryn Sukhait mining area;

3. Setting up 35kv high voltage transmission and transformation lines from Ceke port to mining areas;

4. Investing in construction of the youth center in Gurban Tesi County;

5. Setting up power transmission lines for Aobaotu troops and Gurban Tesi County;

6. Working with Eurasian Natural Resources Corp. PLC to plan and construct coal chemical industry recycling economy park in Ulan Bator;

7. Providing assistance or aid in construction of infrastructure such as schools, hospitals and stations;

Drawing some experiences from development and construction of Mongolia, China Kingko Group follows the trend of economic globalization, national resource and energy policies and "going out" strategy and actively expand the overseas market in the context of global arrangement and strives to form the Group's strategic supporting point and new profit center by fully utilizing the "two markets and two kinds of rsources" so as to gradually realize sustainable development of the group. In the next five years, the group will stretch out development and construction of related projects in Africa, Oceania, central Asia and ASEAN areas in a scientific and reasonable manner and in stages based on its own management and industry trend. At present, here are the projects that are running their preliminary work in a planed and orderly way:

Africa:

Sierra Leone: Plan to explore and develop local iron ore, bauxite and rutile resource etc.

Mozambique: Finance 100 Mozambique students to study in China and formal entry to school will come in September 2011. Starting from that, China Kingho group actively negotiates with the local governments in investment and development of coal, oil and natural gas, and proposes to build coking recycling economy park according to rationed reserves.

Oceania:

We are engaging in exploitation of mineral resources such as iron ore in Australia, New Zealand, Fiji, etc.

The Central Asia:

With Xinjiang Khorgos port as a start point, we plan to exploit resources such as coal, iron ore, phosphate rock and natural gas in the Five Central Asian countries.

Careers

Concept of talent

China Kingho Group adopts the talent principle of career cohesion, performance incentive, cultural inspiration, environment attraction and affection influence, based on domestic and international competition environment, to create a first-class talent team and provide strong human resource supports for steady development and the second rising of the Group.

China Kingho upholds the HR concept of "performance for position" and opens up the sequence channel of the professional growth of talent to effectively promote the career development of all kinds of talents, fully mobilize their enthusiasm, and encourage technical talents study their trades utilizing skills. The leadership growth channel has been created based on the position sequence in order to ensure the fair, impartial, open selection of leading cadres of all levels, vigorously train cadre reserve forces and effectively inspire all kinds of talents' enthusiasm for the work.

Personnel structure

By the end of 2012, the Group already had tens of thousands of employees worldwide. For gender structure the male employees is 75% and female 25% approximately. For age structure, employees under the age of 35 accounted for 70%, the ones from 36 to 45 years old accounted for 22% of the total number of employees. Employees in management positions with undergraduate degree or above accounted for 80%.

Personnel training

Training is an important form of personnel cultivation and an important part of talent team construction. In accordance with the principle of "overall planning, graded responsibility, focused effort and scientific management" and under the training and management mode with unified leadership of the Group, centralized management of HR department, graded responsibility of

various functional departments and classified implementation of subordinate companies, the classified, large-scale, full-covered of training pattern with various companies involved and at different levels has been created so as to establish the "China Kingho workforce quality and ability training system ", a training resource management database shared by the Group's internal training departments at all levels and systematic, targeted training mechanism in order to make China Kingho have a "competent, high-quality workforce to adapt to the needs of the future development of the company", a " diversified talent team that has a sense of modern management and professional standards" and a "modern leadership that loves the Group ’ s business and agree with the its culture and core values".

According to the construction and management of the key personnel of the Group, the HR department has developed the corporate training program, training program for middle & senior management and for managers of functional departments and knowledge and skill training programs for key personnel, etc.; according to professional division, the functional departments is responsible for the development of professional training programs related to the functions of each department, organization and implementation of the department staff expertise heritage training and guidance for staff professional training of subordinate units; the subordinate units based on their own needs combining the Group ’ s strategic goals carry out their own targeted training.

190.Midea Group

Humanizing Technology

Midea Group is a Fortune 500 company, with robust business growth across multiple sectors.

We believe in humanizing-technology, which means we provide customized solutions based on our deep understanding of human nature, enabled by the joint forces of 52 years manufacturing excellence and global leading robotic and automation technology.

We go above and beyond to embrace the future, constantly exploring and inventing to meet the ever-changing demand of our customers and consumers.

Our Vision, Mission & Values

Our Vision is to bring great innovations to life.

Our Mission is to integrate with the world and inspire the future.

We embrace what is next, which is why our core Values are:

- Customer Centric

- Aspiration

- Dedication

- Collaboration

- Innovation

Our Strategy

Our core strategic objectives are:

Technological leadership
Scale up R&D strength through strategic planning and investment in core and cutting-edge technologies.

Digital & intelligent transformation
Comprehensive digital and intelligent transformation to improve internal efficiency and attract more users.

Global impact
Seek breakthroughs in markets, channels, and business models for key areas to serve global users.

Direct-to-clients (DTC)
Adapt existing value chains and business models to directly engage users.
INNOVATION
Innovation is one of our core values, which is why Midea Group invests 3.5% of its profits in research. Our portfolio features global R&D resources across the world, with 31 worldwide innovation centers. We work with over 10,000 R&D personnel and more than 300 leading academics and senior experts from all around the world. We have secured more than 35,000 patents and filed 80,000 granted patents to date.
Award Winning Products
From the first disrotary AC in the world to the first micro-crystalline refrigerator, our R&D team is dedicated to bringing cutting-edge technology into your homes and workplaces. See our award-winning products and recent innovations.
Global Innovations
We structure innovation into four levels so we can focus our research on the latest technology. This system allows us to build our global innovation capacity to support research development over the next 5 to 8 years.
RESEARCH CENTERS
Corporate Research Center (CRC)

Established in 2014

The Corporate Research Center (CRC) is Midea’s top research institute, and focuses on transferring long-term research into common technology, core technology, prospective technological studies, and disruptive product platform innovation.

Serving as Midea Group’s research headquarters, CRC brings the latest trends in global research together with development resources, while promoting global R&D distribution in the USA, Germany, and Italy, along with its R&D Centers in Shanghai and Shenzhen in China.

The focus of CRC’ s research includes technological development in :

- Basic technologies like hydromechanics, solid mechanics, and thermodynamics ;
- Intelligent technologies, such as artificial intelligence and sensors ; and
- Future technologies including IPM , Wireless Power Transmission , and Robotic Technologies.

When CRC founded, specialist teams focusing on user research and product innovation were also established.

Established in 2016

Midea Silicon Valley Emerging Technology Center (ETC) is located in San Jose, California, and focuses primarily on the field of artificial intelligence technology research and application, including computer vision, speech, data science , and industrial AI solutions. Located in Silicon Valley, the world’ s top talent and resource portal, ETC will help Midea Group to establish global partnerships with top industry leaders and to support the applications of cutting-edge technologies in Midea home appliances.
The Midea Germany Research and Development Center
Established in 2017

Midea’s Research and Development Center in Germany is based in Stuttgart, one of the country’s most important locations for innovation and technology. This Center has teams of top specialists from Europe’ s household electrical appliances industry working to support the R&D team and our Headquarters R&D team on local consumer-guided product innovation.

This Center approaches leading businesses from the relevant sectors, as well as the directors of top institutions, with a view to forging close technological partnerships. Our researchers work to bring us the latest cutting-edge research outcomes and innovation know-how, to boost the Midea Group’ s ground-breaking product technology developments and new product R&D.
MANUFACTURING
Midea Group is now one of the biggest manufacturers in the world in several product categories. We command world-leading production capacity and experience and make a diverse range of products in our extensive network of manufacturing bases across the globe.
Midea Business System
As the backbone of Midea Smart Manufacturing, Midea Business System has strategy, talent building, and daily operation at its core. The lean improvement tools and management methods that it provides enable us to boost the efficiency of the entire value chain.

Midea Business System is a unique business method, exclusive to Midea Group, which helps us cut costs and satisfy our customers all over the world.
T+3 Business Model
By carefully studying the real-world demands of our end users, we have geared our business model to tailoring household appliances in line with their true needs. T+3 business model promotes an efficient business process across the entire value chain. From the first stage when the customer places an order (T0), to preparing the materials (T1), making the products (T2), and delivering them (T4), we aim to complete the order within 12 days.
OUR BUSINESSES
SMART HOME
ELECTRO-MECHANICAL
BUILDING TECHNOLOGIES
ROBOTICS & AUTOMATION
DIGITAL INNOVATION
CAREERS
Work at Midea
We offer an environment that’ s open, just, and fair. Our core company values are honesty, trustworthiness, and respect for each other. We believe in rewarding talent and hard work, and we help our employees succeed within Midea Group family, we also think it’ s important to win trust with morality and ability. You will get lifelong training, the opportunity to grow professionally and personally, and you will work together with a dynamic and multinational group of people
Culture
We are inclusive and diverse, and we don’ t just see each other as colleagues, but friends, and sometimes even family. We cherish talent with enthusiasm, courage, and creativity. We believe everyone has unlimited potential.
Career Path
Whether you’ re an intern starting out or an experienced professional, you can find the right position for you here at Midea Group. We offer training at any stage of your career, which facilitates mobility within the company.
191.Chery Automobile Co., Ltd.

Chery Automobile Co., Ltd. was founded in 1997. It is a globalized automobile brand located in China.
INDEPENDENT INNOVATION
Over the past 20 years, Chery has always insisted on independent innovation, established R&D centers in China, Germany, United States and Brazil. It also has established a global automotive R&D team of more than 5,500 people， thus gradually establishing the holistic technology and product R&D system. In this way, Chery has successfully created product brands such as Arrizo, Tiggo with cumulative global sales of more than 10 million units.
GLOBAL PARTNERSHIPS
Chery has implemented a globalization strategy since its establishment and has become the first passenger car company in China to export complete vehicles, CKD parts, engines, manufacturing

technology and equipment to the global market.

In 2012, Chery and Jaguar Land Rover Motors jointly invested in the establishment of Chery Jaguar Land Rover Motors Co., Ltd., which is China's first Sino-British joint venture high-end automobile company.

Chery is not only rich in traditional automotive core technologies, but also accelerating its embrace of the era of intelligent vehicles. Up to now, Chery is cooperating with leading companies on intelligent technologies, such as Horizon Robotics, Huawei, iFly Tek, Alibaba, etc.

FORESIGHT TECHNOLOGY

In the future, the automobile market will enter the era of Electrification, Connection, Intelligence and Sharing. Chery Lion System emerges as the times require. The latest Chery Lion 2.0 system has been applied on Tiggo 8 models, supporting the functions such as voice control, facial recognition, AR navigation, internet service and smart home. Chery has successfully developed the first and second generation of unmanned driving products and has planned to achieve L4 highly autonomous driving in 2025.

Based on information technology, Chery will integrate smart transport, intelligent manufacturing, IOT and block chain, dedicated to providing an intelligent interconnected lifestyle for global consumers in the new era.

HONORS

Chery signed a contract with Grupo-Q, the general agent of five countries in Central America

Chery donated a TIGGO 8 PRO to the association for the disabled ERZI of Kazakhstan

TIGGO 8 PHEV won the title of "the Best Engine under 2.0L" selected by the magazine Auto Esporte in 2023

CAOA Chery won the best after-sales service award of the year selected by UOL, with more than 130000 consumers voting and 26% supporting CAOA Chery.

DESIGN & TECHNOLOGY

DESIGN

Chery's design concept is to combine the spirit and dynamic water flow body shape, reflecting the natural dynamics and appeal of the streamlined new generation 2.0 HDS (Hydro Dynamic Surfacing). HDS styling defines the concept of "vivid" and caters to young users, as well as those forward-thinking users.

AUTOPILOT

Chery places a high value on our customer’s safety and security. Autopilot features monitor, assist or even take control of certain steering, braking and even parking functions on your vehicle, applying advanced technology to further ensure your security in daily driving.

NEW ENERGY

Persists in leading the green revolution of auto industry

ECO-FRIENDLY SMART MODULAR PLATFORM

ECO

Shortened manufacturing process

Evolution of the traditional auto-making process, with number of stations reduced by 50%.

Whole-process green manufacturing, with energy consumption reduced by 80%.

SMART
Smart electric drive
Smart platform-based and integrated pure electric drive.
Multi-power drive system, multi-drive combination mode.
Motor efficiency up to 97%.
Energy recovery rate up to 100%.
Smart battery
Battery intelligent management system.
Liquid-cooled high-efficiency heat transfer structure.
Intelligent heat management.
Thermal runaway suppression technology.
@PILOT
Stronger computing power
64-bit processor, with computing speed up to 4 trillion times per second, which is the double of the competitors of same class.
Improved performance
Extremely rich perceptual information, with more than100 types of objectives detectable.
FROM CEO
Since it was founded, Chery has always taken adhering to technology-driven strategy, creating a vehicle brand with international competitiveness and influence as its corporate vision.

Relying on the constant pursuit of technological innovation, Chery has become the first passenger vehicle enterprise in China to master the core technology of engine, gearbox, chassis, platform and new energy, and also the first one in China to export vehicle, CKD parts, engine and vehicle manufacturing technology and equipment to overseas market.

Chery has always focused on developing domestic and international markets. Chery has continuously deepened its globalization through the implementation of product strategy, localization strategy and talent strategy. After more than 20 years of development, Chery’s sales and services network covers more than 80 countries and regions and has won the trust of 9.5 million consumers worldwide.

The global vehicle market is ushering in a new era of Electrification, Connection, Intelligence and Sharing. which will lead to major changes once in a century. In 2018, Chery officially released the intelligent brand CHERY LION, marking an overall transformation to intellectualization and it committed to providing an intelligent and interconnected lifestyle of new era for global consumers.

Meanwhile, Chery has carried out a variety of social contribution activities concerning green development, environmental protection, social welfare, talent cultivation and other fields in the global market. We hope that our efforts will contribute to the society and make a good impact.

Chery will always persist in technology-driven strategy, take the consumer experience as the core, and provide better products and services to consumers around the world through continuous

innovation and resources integration.

192.BBA

Founded in May 2003, BMW Brilliance Automotive Ltd. (BBA) is a joint venture between BMW Group and Brilliance Group. Its business operations include R&D, procurement, production, sales and after-sales services of BMW automobiles in China. BBA is focused on high-quality development, and aspires to be the leading sustainable provider of first-class individual mobility across the entire value chain of China’s automotive industry. BBA has a state-of-the-art production base in Shenyang, Liaoning Province, branch companies in Beijing and Shanghai, and a subsidiary named Lingyue Digital Information Technology Ltd., with offices in Beijing and Nanjing. The joint venture has around 23,000 employees, more than 430 local content suppliers, and has been the top taxpayer in Shenyang for 16 consecutive years. BBA produced more than 700,000 BMW cars in 2021, making it – for the first time – the largest producer of premium cars in China. Its national sales and service network of 683 BMW outlets has a leading reputation for customer satisfaction among premium automotive brands in China.

Founded in May 2003, BMW Brilliance Automotive Ltd. (BBA) is a joint venture between BMW Group and Brilliance Group. Its business operations include R&D, procurement, production, sales and after-sales services of BMW automobiles in China. BBA is focused on high-quality development, and aspires to be the leading sustainable provider of first-class individual mobility across the entire value chain of China’s automotive industry. BBA has a state-of-the-art production base in Shenyang, Liaoning Province, branch companies in Beijing and Shanghai, and a subsidiary named Lingyue Digital Information Technology Ltd., with offices in Beijing and Nanjing. The joint venture has around 23,000 employees, more than 430 local content suppliers, and has been the top taxpayer in Shenyang for 16 consecutive years. BBA produced more than 700,000 BMW cars in 2021, making it – for the first time – the largest producer of premium cars in China. Its national sales and service network of 683 BMW outlets has a leading reputation for customer satisfaction among premium automotive brands in China.

BBA’s Shenyang production base is the largest in the BMW Group global production network. Guided by the BMW iFACTORY production masterplan, its plants produce technology-leading BMW products using the latest advanced manufacturing processes and digital innovations. It currently manufactures the BMW 1 Series Sedan, BMW 3 Series (long and standard wheelbase), All New BMW i3, BMW 5 Series long wheelbase (and plug-in hybrid variant), BMW X1 long wheelbase (and plug-in hybrid variant), BMW X2, BMW X3 and BMW iX3, and All New BMW X5. In 2021, BBA produced more than 700,000 BMW vehicles across its two vehicle plants – Plant Dadong and Plant Tiexi. The Tiexi location is also home to an R&D Center and Powertrain Plant, which produces BMW Group’s latest generation of 3 and 4 cylinder engines and Gen 5 high-voltage batteries. BBA continues to expand its production footprint in Shenyang, with a complete new plant and a major plant structure extension opening in 2022.

Sustainability is central to BBA’s corporate strategy. As well as lowering its fleet emissions by launching more electrified and low-emission vehicles, BBA has significantly reduced its CO2 emissions, energy consumption, and water consumption per vehicle produced over the years. Its production base is powered by 100% renewable electricity and features circular systems for steel and aluminum. It shares its best practices with its partners to establish CO2 disclosure

mechanisms and energy management capabilities, to cascade sustainability into its value chain. Through these actions, BBA is contributing to the BMW Group target of reducing CO2 emissions across the entire life cycle of a vehicle by at least 40% by 2030 from 2019 levels. BBA ranked No.1 in China’s Automotive Enterprise Green Development Index in 2021, and has received National Green Plant accreditation for 5 years in a row.

Corporate Social Responsibility (CSR) is an integral part of BBA’s sustainable development strategy. BMW CSR leverages BMW’s core strengths and stakeholders to solve real social problems, create shared value and contribute to Chinese society. BMW CSR activities include caring for left-behind children with BMW JOY Home, safeguarding China’s intangible heritage with BMW China Culture Journey and improving road safety with BMW Children’s Traffic Safety Education. In 2021, BMW extended its CSR strategy to environmental protection with the launch of the BMW Beautiful Homeland Initiative.

As a responsible corporate citizen of China, BBA is fully committed to developing local talent, creating high-quality jobs, actively supporting its partners, and contributing to the economic development of Shenyang, Liaoning Province and Northeast China. It will continue to integrate responsible and sustainable practices throughout its entire value chain, contribute positively to Chinese society and provide its customers with the high-quality products and services that the BMW brand stands for.

Corporate Social Responsibility

BMW believes that a truly responsible and sustainable business must have a higher purpose than simply achieving profit and offer employment - it must work to contribute to improve the society. At home in China, BMW China continues to create values for the Chinese customer, the local society and industry for mutual growth.

Good CSR understands the urgent and long term needs of government and society, and contributes to the needs. As a contributor addressing social needs, a connector of local communities, and a differentiator in fulfilling corporate responsibility with innovation in China, the BMW leverages core strengths and widely engages stakeholders to create shared value.

Strategy

BMW advocates "Creating Shared Value" in fulfilling Corporate Social Responsibility. In implementing innovative CSR projects that respond to real social issues, BMW creates shared value that benefits both society and the enterprise. We continuously seek innovative opportunities in our CSR projects, and join hands with stakeholders to address social challenges.

Guided by strategic CSR, BMW holds true to its three principles: long-term development, efficacy comes first, and all-round stakeholder engagement.

BMW Children’s Traffic Safety Education

BMW Children’s Traffic Safety Education (CTSE) focuses on the issue of children’s traffic safety. The program integrates the traditional Chinese value of “propriety” into traffic safety education; develops relevant experiences, toolkits and an online platform; engages stakeholders in CTSE Ambassador Volunteer Training and volunteer service; and enables dealers to implement CTSE-related activities in local communities. This ongoing program launched 18 years ago has benefited more than 100 million people so far.

BMW Sustainability & CSR Forum

BMW has consistently dedicated itself to being a good corporate citizen, actively performing the obligations of its localization strategy, and contributing to the sustainable development of China. With an international perspective, BMW has run the BMW Sustainability & CSR Forum in cooperation with various partners since 2013, providing a platform for key stakeholders to demonstrate, share and exchange their experiences in sustainability, while involving extensive social forces to encourage creative ideas and practices on corporate social responsibility.

CSR Awards

For years, BMW has been adhering to the concept of sustainable development, pursuing the harmonious development between corporate and society, passing honesty and warm heart to the public, and contributing to the society with innovative CSR concept. Therefore, BMW has been widely recognized and received many CSR awards.

BMW believes that a truly responsible and sustainable business must have a higher purpose than simply achieving profit and offer employment, it must work to contribute to improve the society. The implement of BMW's corporate citizenship in China exemplifies BMW ’ s long-term commitment to the Chinese society.

Create Shared Value

BMW advocates "Creating Shared Value" in fulfilling Corporate Social Responsibility. In implementing innovative CSR projects that respond to real social issues, BMW creates shared value that benefits both society and the enterprise. We continuously seek innovative opportunities in our CSR projects, and join hands with stakeholders to address social challenges.

Guided by strategic CSR, BMW holds true to its three principles: long-term development, efficacy comes first, and all-round stakeholder engagement.

Our Philosophy

At home in China, BMW Group continues to create value for Chinese customers, local society and industry for mutual growth.

Our Mission

To Continually Contribute to a Better Chinese Society

Our Goals

• Promote upward social mobility through tailor-made programs targeting the needs of disadvantaged groups in the Chinese society.

• Leverage BMW’s core competencies to contribute to the safe and sustainable mobility in local community.

Our Focus Areas

Cultural Protection, Social Development, and Environmental Protection.

193.China Longyuan

China Longyuan was established in 1993. At that time, it was affiliated to the China National Department of Energy. Later, it was turned to be affiliated to the Ministry of Electric Power and State Power Corporation, then it was put under the former China Guodian Corporation in the 2002 power system reform. In 2017, China Guodian Corporation and Shenhua Group merged and reorganized, and China Longyuan was put under China Energy.

China Longyuan has long been representing the country in the research and development of new

energy technologies. It is the earliest specialized company to develop wind power in China. It was successfully listed in Hong Kong in 2009, setting a number of firsts including the largest financing in overseas initial public offerings by Chinese power companies and the highest price-earnings ratio. It is known as the "First Chinese Stock of New Energy". It became the world's largest wind power operator in 2015. By the end of 2018, China Longyuan had an installed capacity of 21,044 MW, of which wind power had an installed capacity of 18,919 MW, and continued to maintain its position as the world's largest wind power operator. After 26 years of development, the company has become a large comprehensive power group focusing on the development and operation of new energy. Its business is distributed in 32 provinces and cities in China and Canada, South Africa and other countries.

Based on good business performance, the company has won many awards such as Best Management, 13th Five-Year Most Investment Value, Most Brand Value and Best Listed Company in the Golden Bauhinia Awards. It has been rated as Global Top 500 New Energy Enterprises for 6 consecutive years, and won the National May 1st Labor Award granted by the All-China Federation of Trade Unions for the highest honor of enterprises and institutions, and was awarded the honorary title of National Civilized Unit by the Spiritual Civilization Development Steering Commission.

Message form the chairman

As global energy shortage, oil price rises, environmental pollution and climate warming issues become increasingly prominent, governments worldwide have taken the development of new energy as a major strategic choice for seeking sustainable development approach and cultivating new economic growth points. The 18th CPC National Congress put ecological civilization construction in a prominent position and put forward the construction of beautiful China. Striving to develop new energies and promoting energy production and consumption revolution is an important guarantee for constructing ecological civilization and beautiful China.

China Longyuan Power is the pioneer of new energy industry in China, and the company has always been dedicated to developing and utilizing clean, efficient and sustainable new energy since the establishment. Through productive exploration and practice over the years, its wind power has maintained an industry-leading level in scale and efficiency. The company has also established complete new energy power generation systems including solar energy, biomass energy, tidal energy and geothermal energy, thus forming core competition advantages with leading strategies, management and technologies. As a listed new energy company in international capital market, we will always adhere to the operation principle “good faith, profession, standardization, efficiency”, and repay investors and the society with outstanding achievements.

The development and utilization of green energy resources will simply never be an economic topic. What it conveys is the expectation for good life and the identification with the value system of harmonious development of environment, society and human. China Longyuan Power is just marching forward on the path of developing new energy based on the fundamental starting point of human well-being and sustainable social development. We will continue to follow the national strategic energy deployment and seize the world’s new energy development direction, and will

untiringly struggle for building a world class listed new energy company and make more contribution to the development of global low carbon economy.
Corporate Culture
Corporate Objective

Corporate mission----Build up a resource-saving and environment-friendly society; to develop green energies and cultivate a wind power brand.

Business concept----Develop wind power, save resources, protect the environment, and bring benefits to the next generation.

Corporate Values

Guidelines ----Pragmatic and innovative for bigger and more competitive

Corporate spirit-----Promote the industry with electricity, and serve the country worthily by reinforcing the enterprise

Professional ethics----Loyalty to the cause, loyalty to the Group, devotion to posts and work, and devotion to be an useful staff

Core values----Stringency Efficiency Justice Harmony future
Accolades and Honors

HONOURS AND AWARDS

2018

“The Best Listed Company” at the Eighth Session of China Securities Golden Bauhinia Award

2018

“Model Company in Corporate Culture Building in the Power Industry in China ”

2018

“Outstanding Issuer” and “Innovative Business” at the “Commendation Conference of 2017 Excellent CCDC Members”
Business
Wind Power Generation
Solar Power Generation
Sci-tech Businesses

Thermal Power Generation
Geothermal Power Generation
Tidal Power Generation
Biomass Power Generation
194.Jiangsu Geology & Engineering Co.Ltd,
Jiangsu Geology & Engineering Co.Ltd, founded in 1997, was registered by capital of 101.88 million yuan, which was approved by the Ministry of Commerce, the Ministry of housing and the Ministry of land and resources, which has the qualification of foreign project contracting, Grade-A of geotechnical engineering investigation, Grade-A of geological hazard control engineering construction, Second level of housing construction general contracting (level two), professional contractors of Decoration Engineering (level two), foundation and foundation engineering contracting (level two), electrical and mechanical equipment installation engineering contractor (level two), Grade-B of engineering survey, Grade- B of hydro geological survey, engineering survey and qualification services laboratory accreditation certificate and etc, and also pass through state-owned shares and by three in one comprehensive enterprise quality system certification project. In 2013, Sugec was included in the eighteen units of backbone enterprises for complete sets of foreign aid project survey of the Ministry of Commerce.

Under perfect corporate governance, the company has set overseas branches in Mozambique, Tanzania, Angola, Bolivia, Australia, Kazakhstan, Nigeria and other countries, and also domestic branches in Suzhou, Wuxi and Huaian.

The company attaches great importance to the cultivation of compound talents who know technology, language and management. The company has all kinds of professional and technical personnel, totally 161 members, accounting for 95% of the total employees, of which more than 13 personnel of master's degree or above, 128 personnel of the bachelor's degree, 15 personnel with senior engineer titles or above, 27 personnel with intermediate engineer titles or above, 5 registered civil engineers (Geotechnical) , 7 registered constructors of Level one, 10 registered constructors of Level two, 1 registered safety engineer, 1 certified accountant, 46 security staff.

The company has always attached great importance to the overseas

construction equipment investment construction and overseas base construction. According to the needs of overseas projects, new equipment investment has been 80.11 million yuan during 12th Five-Year, totally more than 900 sets, such as rotary drilling rig SR250, loaders, rollers, bulldozers, dump trucks, asphalt paver, mixing machine, crane and a large number of huge equipment for highway construction, basic construction and mineral exploration.

The domestic and overseas projects are involved in road and bridge construction, infrastructure construction, well drilling and water supply, engineering investigation, the disaster management, engineering survey, foundation pit design and testing, mineral resources exploration & development, housing construction and mechanical & electrical installation, geothermal energy development, international trade, etc. For the last decade, SUGEC had undertaken hundreds of investigations, designs, construction projects and completed several large international bidding projects in Chinese mainland, Africa, Asia, Middle East, South America, and South Pacific area which had drilled two thousand wells, constructed pumping stations, water towers, laid water pipelines for one thousand miles. All the achievements in overseas project investigations, large equipment installations were particularly well received by owners and other enterprises in the same trade.

Enterprise culture

Strengthening the building of enterprise culture can motivate our employees a sense of mission and raise their sense of belonging and responsibility, it can also entrust employees with senses of honor and achievements.

SUGEC is always thinking highly of enterprise culture and has strengthened the culture, enhanced cohesive force, centripetal force of enterprise by employee training, internal& external technology, experience exchange, competitions, outdoor tour which all staff took part in time by time.

Development

The projects which are managed by SUGEC in the field of domestic and overseas are involved in road and bridge construction, infrastructure construction, well drilling and water supply, engineering investigation, the disaster management, engineering survey, foundation pit design and testing, mineral resources exploration & development, housing construction and mechanical & electrical installation, geothermal energy development, international trade, etc. SUGEC had undertaken hundreds of investigations, designs, construction projects and completed several large international bidding projects in Chinese mainland, Africa, Asia, Middle East, South America, and South Pacific area. All the achievements in overseas project investigations, large equipment installations were particularly well received by owners and peers.

Qualification certificate

Engineering Survey Qualification Certificate(Class A Geological Engineering)

Geological Survey Qualification Certificate(Class C Liquid Mineral Survey)
Service center
Infrastructure Pipeline
In the recent years, as a new growth point in the field of overseas engineering business, the foundation construction and pipeline excavation have made great development. The company has implemented more than 30 projects of different types and scales in Mozambique, Tanzania and Angola by increasing input of equipment and personnel. Especially in the process of the implementation of Tanzania Natural Gas Pipeline Project, our staff played a good tradition of hard-working, allocated reasonably and managed scientifically, which got highly praised by owners and cooperative unit and rated the title of "Excellent Cooperative Enterprise" by China Petroleum Pipeline Bureau.
Road Bridge
WellWater Supply
Engineering Exploration
Lab Intro
Survey Monitoring
Geological Disaster Control
Building Construction
Geothermal Energy
International Trade
Idea of talents
The core idea: let the active employees have opportunity to work, let the man of ability have platform to perform, Let achieved employees have fair position and remuneration. The ones really need. Valuing talents, developing talents!

The first principle is to pay attention to both ability and political integrity. The selection and use of personnel should be in accordance with a comprehensive measure and basic requirements of both ability and political integrity.

Second principle is "effect theory". In the use of talent, we pay more attention to ability rather than degree. No matter what position, what degree, what age it is, if done well with outstanding performance, boldly entrusted with important task. At present, the average age of the company's technical staff and middle-level staff is around 35 years old; the average age of the workshop director is around age 30. They are not aged, some education level is not very high, however, their ability, energy, performance was been recognized. By Implementation of "effect theory" employing mechanism, a lot of employees with both ability and political integrity became the pillars of company development.

Third principle is "practice first". It is said that it may take 3 days to test the fake of jade, but must wait 7 years to test the ability of talent/person. Over the years, all kinds of professionals who are working in our company were arranged to the frontline in practicing to improve their abilities. Then recruit the personnel through examination.

Fourth principle is "survival of the fittest". In the selection of talent, we realized the transition

from "selection" to "competition". The one, who do the best, will be considered as a talent to be reused; otherwise, can only be eliminated.

Fifth principle is "everyone is talent". In use of the talents, we do eliminate formula and mysterious form; apply principle of "suitable for human". As long as in the appropriate position to perform the greatest degree of intelligence, it can call talent to some extent. We follow this principle according to each employee's level of expertise, experience, personality, etc., arrange to the relative position; make the best use to do their best to promote the sustained, rapid and efficient development of enterprises.

195.PowerChina Guizhou Engineering Co.,Ltd.

PowerChina Guizhou Engineering Co.,Ltd. was established in Guiyang on November 27, 2015, which is affiliated with Power Construction Corporation of China. It was reorganized from the former Guizhou Power Construction 1st and 2nd Engineering Company under China Power Construction Group. According to the overall deployment and requirements of the Group, PowerChina Guizhou Engineering Co.,Ltd. decided to change its name and obtained the approval of State Administration for Industry and Commerce of the People's Republic of China for name change on September 29, 2017. On December 22 of the same year, it completed the relevant procedures required by the administrative department for industry and commerce, obtained the business license and renamed as PowerChina Guizhou Engineering Co.,Ltd.(hereinafter referred to as GEPCC)

As a comprehensive engineering management company integrating investment and financing of power engineering and infrastructure, planning and design of new energy projects, construction and operation management, GEPCC is committed to creating wealth for the society and value for customers around the five energy markets of new energy business, overseas business, infrastructure business, power traditional business and traditional derivative business. With our professional technical ability and excellent project management, we have gradually formed the advantages of the whole industrial chain integrated system solution platform. We are an important player, contributor and leader in the field of green development of global energy. In order to realize the development objectives of renewable energy set in the China's 13th Five-Year Plan, we have always followed and implemented the national development concept of Innovation, Coordination, Green, Openness and Sharing in developing new energy business. As a state-owned enterprise, we undertake the mission and responsibility, and spare no effort to make contributions to the mitigation and adaptability of climate change for China and even the world， establishment of a green and low-carbon circular development economic system for social progress and development, promotion of international energy structure and adjustment of strategic project.

Based on the management experience in EPC project of new energy in recent ten years, PowerChina Guizhou Engineering Co., Ltd. has significantly enhanced its resource allocation ability, market competitiveness and value creativity through mode innovation and brand re-engineering, and has become a brand leader company in the field of new energy and a industry-leading company in the domestic integration services of development, construction and operation for clean energy.

PowerChina Guizhou Engineering Co., Ltd. actively responds to the national "One Belt and One Road" initiative and is committed to becoming the world's leading EPC contractor of renewable energy projects. Focusing on the development trend of renewable energy, it has actively entered overseas markets from an international perspective. As the executive vice president of International New Energy Solution Platform, executive chairman of Photovoltaic Green-Ecosystem Organization (PGO) and member of National Photovoltaic Industry Alliance for Modern Agriculture., the company has successively established strategic alliances with China Merchants New Energy Group, Huawei Technologies Co., Ltd., China Photovoltaic Industry Association and various large financial institutions, etc. and has established a good cooperative and long-term strategic partnership with owners, supervisors, investment and financing companies, jointly aiming to the promotion, application and research of high-tech equipment and technologies of new energy power station. On September 9, 2017, the company's subsidiary was restructured and renamed as PowerChina New Energy Power Engineering Co., Ltd., which increased the company's confidence and ability to provide integrated customized services for new energy projects for all sectors of society.

In the future, under the correct leadership of the Group, all employees will continuously stick to deepen reform and transformation, lean management to improve quality and efficiency of service, and advance towards the goals of making the company a world-renowned and domestic advanced renewable energy leader, intelligent energy creator , comprehensive solution provider and value creator of industrial chain with diligence, determination, unity and cooperation.

Message from Chairman

With the corporate vision of "create value for customers, create benefits for the company, create happiness and benefits for employees, and create wealth for the society" and by virtue of word-class quality, reputation and construction, we are committed to providing world-class engineering services for the industry with high-end brand, flexible mechanism, leading technology, advanced culture and high-quality resources, and carrying out extensive economic and technological cooperation.

We look forward to working with all relevant parties on the basis of equality and mutual benefit to build a path of high-quality development that is in line with China 's national condition, which is inclusive and mutually beneficial to all. We infiltrate the performance of social responsibility into all aspects of operation and management, committed to sincere cooperation with customers, employees, partners, industry and social stakeholders, and attach equal importance to both business operation and social responsibility..

Up to now, PowerChina Guizhou Engineering Co., Ltd. has successfully realized the optimal allocation and complementation of resources, the continuous expansion of industrial structure and business areas, and further promotion of the transformation and upgrading of the company. Facing the new development platform, PowerChina Guizhou Engineering Co., Ltd. is embarking

on a new journey, and we will work with all sectors of society with full passion to create and realize harmonious development among enterprises, society and nature.!

Honor and strength

PowerChina Guizhou Engineering Co., Ltd. has won the Luban Prize for Construction Project, Tien-yow Jeme Civil Engineering Prize, National Excellent Construction Enterprise, National May 1st Labor Award and other awards. In recent years, thanks to its good reputation in new energy business and its strong performance strength, Guizhou Engineering Co., Ltd. has won the 2016 "China Photovoltaic" Top Ten Photovoltaic EPC Service Providers and 2016 Best Photovoltaic Power Plant Operation & Maintenance Service Providers, as well as 2017 Asia Photovoltaic Innovation Enterprise and other honors. As a national high-tech enterprise, Guizhou Engineering Co., Ltd. has established an Innovation Institute, with a number of experimental, testing and talent training institutions including Guizhou Jinchengte Nondestructive Testing Co., Ltd., Guizhou Electric Research Special Equipment Inspection Center Co., Ltd., Skill Appraisal Station of Guizhou Engineering Co., Ltd., Welding Training Center of Guizhou Engineering Co., Ltd., State-level Liu Yunchun Skills Master Studio , etc. The Company also actively carried out the IUR (Industry-University-Research), and signed scientific and technological cooperation framework agreements with Guizhou Mechanical and Electrical Research and Design Institute, Institute for Advanced Technology of Guizhou University and other scientific institutions, in order to jointly carry out research on scientific and technological projects.

With the vision of “creating value for customers, creating benefits for enterprises, creating wealth for society, and creating well-being for employees”, the Company is committed to providing customers with the full EPC project industrial chain "integrated" customized services in engineering construction. The Company also has perfect technology, quality management system and strong scientific and technological innovation ability.

Ⅰ. Integrated investment and financing operations: The Company inherited the traditional contracting business and has continuously improved the structure optimization and industrial adjustment in an orderly manner, created a business model in which investment, financing, and general contracting are organically combined, and built an integrated upstream-downstream business model. The Company is transforming into a diversified, collectivized, knowledge-type, technology type and management type company, with the capacity of resource integration, one-stop professional services and investment and financing.

Ⅱ. Talents and machinery: Guizhou Engineering Co., Ltd. has more than 2,200 employees, including more than 1,200 persons with middle-level and senior-level titles in technical and economic management, and nearly 200 persons with national registered practicing qualifications. The Company has 3,200 sets of various advanced machinery. The total power of machinery and equipment is nearly 40,000 kilowatts. Its original value of fixed assets is 500 million yuan, and it has the capability for large-scale modern engineering construction.

Ⅲ. System Certification: The Company has passed the "Four Standards" Certification of ISO9001 Quality Management System, GB / T28001 Occupational Health and Safety Management System,

ISO14001 Environment Management System and ISO10012 Measurement Management System.

Ⅳ. Research & Development Strength: The Company has passed the accreditation of provincial enterprise technology center and is committed to the promotion and application of "five new" technologies (including new equipment, new workmanship, new materials, new technology and new process) and the technology research & development. The Company has 16 masters and 65 engineering technology application researchers. The Company has 81 employees with vice-senior title and 244 employees with intermediate title. The Company employs 1 expert with provincial government special allowance and 2 other experts. Guizhou Engineering Co., Ltd. has a number of experimental, testing and talent training institutions including Guizhou Jinchengte Nondestructive Testing Co., Ltd., Guizhou Electric Research Special Equipment Inspection Center Co., Ltd., Skill Appraisal Station of Guizhou Engineering Co., Ltd., Welding Training Center of Guizhou Engineering CO., Ltd., State-level Liu Yunchun Skills Master Studio, etc. The Company has obtained more than 30 scientific research achievements, including more than 20 patents, software copyrights, third prize for Provincial and Ministerial Level Scientific and Technological Progress Award, and construction methods. The Company has participated in the compilation of more than 10 local and industrial standards and developed a series of core technologies with independent intellectual property rights.

Ⅴ. Informatization Capability: The Company has developed and put into operation more than 20 information systems based on WAN, including OA, financial capital system, human resource system, management and control platform, integrated information system platform, multimedia presentation, PRP project management, production command system and others. The information system has realized the full life cycle information management for the project construction.

Sustainable Development

Adhering to the strategic policy of "developing harmoniously and fulfilling social responsibility", sticking to the sustainable development strategy of "improving people's livelihood and promoting social development", holding on the mission of "promoting clean energy", and persisting in the cooperation concept of "win-win cooperation and development". While promoting overseas economic growth, benefiting the local people and building friendship, Guizhou Engineering Co., Ltd. will create an ecological friendly environment and establish a positive brand image.

Service: customer oriented, continuous innovation, to provide customers with integrated full industry chain services.

Leadership: in terms of global new energy, the Company is committed to building a leading brand and becoming the "hidden-champion enterprise".

Responsibilities: the Company is ready to perform the responsibilities entrusted by the government, industry and society at any time.

Vision: creating value for customers, creating benefits for enterprises, creating wealth for society, and creating well-being for employees.

Overseas business

Positively responding to the national "the Belt and Road" Initiative, PowerChina Guizhou Engineering Co., Ltd strives to push forward overseas strategy and 2018 has witnessed a growth of overseas market since breakthrough was achieved in this aspect in 2017, with signature of many contracts including the Helio Wind Power Project in Argentina, the 97 MW Photovoltaic Project in Cafayate of Argentina and the 130 MW photovoltaic power generation project in Cherikov, Belarus.

It established an international marketing mode, with the overseas business division to lead the global marketing team. Besides setting up many marketing development teams, it also built overseas cooperation teams including European team, African team and American team, nurturing a team of talents to perform projects overseas, gradually developing the overseas marketing system.

Services&products

Thermal power

New energy

Infrastructure

Traditional extension

196.Sentuo Group

Supported by advanced technology, Sentuo group has been rooted in Ghana since 2008. We are fully involved into local market and with consistently expanding, Sentuo group currently comprises six business sections including Sentuo Steel Limited, Fujian Sentuo Ceramic Tile Company Limited, Habilass Resources Company Limited, Sentuo Resources Recycling Limited, Sentuo Oil Refinery Limited and Sentuo Building Materials Complex and now hires more than 4500 local emplyees.

BUSINESS

Fujian Sentuo CeramicTile Company Limited

Fujian Sentuo Ceramic Tile Company Limited is specialized in the production of ceramic tiles. It covers an area of 50 acres and has a total production capacity of 45 million cubic meters. Sentuo Ceramic Tile company is the largest ceramic manufacturer and whole-seller in Ghana, employing more than 2,000 local employees.

The company uses advanced ceramic tile production equipment with an environmentally friendly recycling system and has established strict internal quality control standards.it also has a complete logistics and storage center to provide quality products and efficient service.

The products consist of indoor floor tiles, indoor wall tiles, outdoor floor tiles, outdoor wall tiles and related accessories, with thousands of designs and colors.

The company focuses on innovative technology, and strives to create the highest quality products to Ghana and West Africa markets. "Inheritance, innovation and People oriented" is the business philosophy that Sentuo Ceramic tile company has adhered to. We will continuously enhance the core competitiveness of the enterprise, create a healthy and comfortable living space for consumers, strive to become a well-known brand in Africa, and strive towards the goal of a century-old ceramic enterprise.

Sentuo Steel Limited

Sentuo Steel Ltd. is the first project of Sentuo Group in Ghana. We came to Ghana in 2008 for business investigation and spent two years to conduct in-depth research on Ghana's political, culture, investment environment and Ghana's industry and market conditions. The phaseI of the project was completed in March 2012, mainly producing iron rods and coil; the phaseII of the project started in early 2014 and completed in 2015, with a total production capacity of over 800,000 metric tons per year. There are more than 1,200 local employees. The high quality of our products has earned us recognition and trust of our customers, and established a first-class brand image in the Ghanaian steel market, which has laid a solid foundation for the development of follwing projects of Sentuo Group in Ghana.

Sentuo Steel is a cooperative project between Chinese private investor and the Ghanaian government. It is not only a fuel for promoting local economic development and employment, but also a carrier for sparking international cooperation ideas. In today's international society, competition and cooperation interact, opportunities and challenges coexist. We not only participate in competition, but also pursue cooperation, and work together with partners to seek common development. Sentuo Steel will adhere to the business philosophy of "advance with the times, adhere to development, mutual benefit and win-win" to make the enterprise better and stronger.

Habilass Resources Company Limited

Habilass Resources Company was established in 2014 and is located in KIBI City, Eastern Ghana. The company currently has about100 local employees. It owns the mining rights of a gold mine covering an area of 90 square kilometers, with abundant mining resources. It is estimated that 30-50 production lines will be installed in the full-scale mining stage. Currently in the early stage of exploration and production, the initial investment is 40 million US dollars. Since its establishment, Habilass has always been committed to efficient and high-quality mining with environment protection responsibility in the mining field.

Sentuo Resources Recycling Limited

Sentuo Resources Recycling Limited, a subsidiary of Sentuo Group, was registered in 2019 and

located in Tema industrial area with 20,000 square meters land. It is close to the national highway and main port which is easily accessible and convenient for import and export of raw materials and accessories.

Recycled waste circuit boards from electrical household appliances and electronic products such as monitors, smart phones, feature phones,batteries , as well as coppers are dealt through dismantling, pyrolysis processing and the electrolytic processing to achieve systematic classification, safe disassembly, efficient recycling and delivery.

Sentuo Renewable Resources currently has employees who are experienced in the E-waste recycling and related industries to assure professional production management and quality control and we now have Apple system workshop, intelligent IC workshop, functional IC workshop, waste battery, finished battery workshop and copper workshop and we have established raw material procurement offices and long-term supplier partners in the United States, Europe and Africa.

Sentuo Oil Refinery

Sentuo Oil Refinery Limited was conceived from the Chinese government ' s Belt and Road Development Strategy for the oil and gas industry in China & Africa. Following extensive research and consultations, the Sentuo Group executes the oil refinery project. The "Sentuo Oil Refinery Limited (SORL)" phase 1 will be completed in October 2022 and will have an initial production capacity of 2 million tons per year (tpy). Phase 2 of the Sentuo Oil Refinery will be completed in January 2024 and will operate at a capacity of 5 million tons per year (tpy).

The Refinery is located at the Tema Industrial Zone in Ghana in West Africa. In accordance with the government of Ghana's "One District One Factory" development policy, as well as the general planning of the Tema Industrial Park area, the project essentially factors in key features such as modern scientific technology and innovation, energy conservation and environmental protection, recycling economics, phase & rolling development and the scalability of production capacity and quality; which are all key requirements of any new production and/or manufacturing project.

By 2024, when the two phases of the project are fully completed and commissioned, 5 million tons of all types of crude assays can be refined per annum, with an output of 3.2 million tons of refined petroleum products such as, gasoline, kerosene, and diesel of high quality above the Euro IV standard. In addition, the refinery will produce 350,000 tons of a series of pitch products, 200,000 tons of lubricating base oil and solvent naphtha and 400,000 tons of by-products such as polypropylene, liquefied gas, ammonium sulphate, sulfuric acid and sulphur. Sentuo Oil Refinery Limited (SORL) will become the model refinery project of the medium size category in the West African market. It will provide thousands of employment opportunities and drive an interconnected development for the industries of chemical engineering, automobile, machinery

and logistics in Ghana as well as the West Africa region.

We aim to become the hallmark in refinery projects in the Ghanaian market and to drive interconnected development for the industries of chemical engineering, automobile, machining and logistics in Ghana, and the West African region. And we are dedicated to develop a benchmark for petrochemical production in the West African market based on superior technology, and to provide a safe home for West Africa's crude oils, to unlock energy value.

Sentuo Building Materials commercial Complex

Sentuo Building Materials Complex is Located in Ashiaman, Tema and covers an area of 80 acres, it is only 7 kilometers away from Tema port, which is very convenient for logistics and dispatching.

The company was planned with a construction area of 390,000 square meters and invested by Sentuo group to develop. And it is already under the civil works and expected to be put into use in 2024, with a total investment of 320 USD million on the project.

Depending on the industry of building materials and technologies in China, the company set up a brand-new industry model, constructing five platforms and one center involving three parties including manufactures, retailers and consumers. Five platforms are for trading, rating, logistics, manufacturer’s storage, management & after-sale; One center is for vehicle operations. By then, the company will become an international commercial complex of multi-functions, including building materials mall, brand shops, warehouse logistics, HQ building, residential supporting facilities, providing a comprehensive after-sale guarantee service for countries and regions including Ghana, Nigeria, Mali, Ivory Coast and Togo.

With an integration of the industry chain of building materials, commerce, and related services, it will become the largest and most modern environment-friendly building materials market as well as the industry leader in the West Africa.

197.Ningxia Tianyuan Manganese Industry Group

Ningxia Tianyuan Manganese Industry Group in Progress

Ningxia Tianyuan Manganese Industry Group Co., Ltd. is a professional company, intensification, internationalized large enterprise groups, is the world's largest electrolytic manganese production enterprise, the market share is 40%. These years, the Group has adhered to manganese as the main industry, to build a century-old enterprise as the development goal, and vigorously develop circular economy. After many reforms and upgrades, the industrial structure is more rational, the total economic volume is increasing, the comprehensive strength and the

market competitiveness are increasing. A development pattern based on real economy, driven by international trade and led by strategic emerging industries has been established, and manganese industry, energy and chemical industry, international trade, logistics transportation, financial services, overseas mines, emerging industries and real estate services eight major business sectors have been formed. It was awarded the "Excellent Environmental Protection Enterprise Award" by the United Nations Environment Planning Foundation and the Environmental Protection Association of the four places across the Taiwan Straits, and recognized by the Ministry of Industry and Information Technology as the "Cleaner Production Demonstration Enterprise" in key industries and the first batch of "Green Factory" demonstration enterprises; it was awarded the " China's Most Influential Enterprises in 2017 " by China Enterprise News Group and the Top Ten News Selection Committees of Chinese Enterprises; ranked as "Top 500 Chinese Enterprises" for five consecutive years. Tianyuan Manganese trademark has been rated as a well-known trademark in China.

Enterprise advantage

Diversified advantages - form a diversified development pattern based on entity, driven by trade, promoted by finance and led by strategic emerging industries. Take trade and finance as the escort of the real economy, which effectively guarantees the long-term and sustainable development of the company.

Scale advantage - as the company's leading product, electrolytic manganese has an annual capacity of 800,000 tons, accounting for about 40% of the industry's total production capacity. With a series of supporting products such as high carbon ferrochrome, high-silicon si-mn, it has become the absolute leading enterprise in the industry.

Technical Advantage - The company is the only electrolytic manufacturer that uses all imported high-grade manganese ore in China. The exclusive secondary leaching technology of high-grade manganese ore increases the recovery of manganese from 60% to 98%, improves the utilization rate of ore and saves the cost of ore.

The technology of manganese dioxide closed roasting and tail gas recovery to produce sulphuric acid developed by the company is the first in China, which solves the environmental protection problem of manganese dioxide roasting and diversifies the raw materials of electrolytic manganese metal production.

Advantage of industrial chain - A "one-stop" production mode has been formed, which produces and supplies all raw materials, auxiliary materials and electricity by itself and eventually outputs products, thus avoiding the impact of market fluctuation of raw and auxiliary materials on production.

Advantage of comprehensive utilization of resources - water, steam and slag produced in production are all recycled, which reduces the production cost and eliminates the pressure of environmental protection.

The advantages of green environmental protection - the company is a national green

environmental protection demonstration enterprise, which has laid a solid foundation for the long-term development of the company and the support of the national policy.

Resources Advantage - China is short of manganese resources, basic reserves are only about 200 million tons, accounting for about 2% of the global reserves, and the grade is low (10% - 15%) and impurities are large. Our company has high-grade manganese ore resources in Australia and Ghana (more than 30%) and has absolute advantages in ore resources.

Energy Advantage - At present, Ningxia's per capita electricity generation is the first in the country, with sufficient electricity, which guarantees the electricity demand of electrolytic manganese metal production, and the low cost of electricity makes the company have absolute advantage in power resources.

Policy Advantage - Manganese resources belong to the national strategic resources. The national policy supports the introduction of manganese ore.
Enjoy 15% Preferential Tax Rate of "Western Development"
Enjoy the preferential tax policy of "three exemptions, two halves" for attracting investment
Enjoy the preferential policies of "leading enterprises and key supporting enterprises in the autonomous region" in land, electricity prices and transportation

Climate Advantage - The company is located in the plateau, the climate is dry, reducing the degree of surface oxidation of electrolytic manganese metal sheets, ensuring the quality of electrolytic manganese metal, purity can be up to 99.8%.
Business Sector
Manganese industry
Energy and chemical
Real Estate Services
Logistics Transportation
Financial Service
International Trade
The international trade sector is positioned in the import and export management of the group's ore and non-ferrous metal trade, and the management mode adopts the two-line parallel and professional operation mode. The international trade sector will make full use of Shanghai's advantages as an international trade center and financial center, further improve the operational efficiency and management level of the international trade sector by optimizing the management system and process, and realize the optimal allocation of financial and trade resources by enlarging and strengthening the trade scale.
Overseas Mining Industry
Emerging Industry
PRODUCTINT RDUCTION
Electrolytic Manganese Metal (HS code: 81110010 unforged manganese, manganese scrap, powder). Manganese and manganese alloys are indispensable important raw materials for iron and steel industry, aluminum alloy industry, magnetic material industry, chemical industry and so on. Manganese is an indispensable additive in smelting industry. With the continuous

development of science and technology and the continuous improvement of productivity level, electrolytic manganese metal has been successfully and widely used in iron and steel smelting, non-ferrous metallurgy, electronic technology, chemical industry, environmental protection, food hygiene, welding rod industry, aerospace industry and other fields. As the leading product of Tianyuan Manganese Industry Group, the annual production capacity of electrolytic manganese metal has reached 800,000 tons and the market share has reached about 40%.
198.MSCcargo
LEADER IN SHIPPING & LOGISTICS
Our Solutions

As well as being a global leader in container shipping , our worldwide teams of industry specific experts mean we can offer our customers round-the-clock personalised service. This ensures we deliver fast and reliable transit times, and that we provide the best solutions for your needs.
MSC is one of the world’s most reliable carriers, with outstanding global schedule reliability.
SHIPPING SOLUTIONS
Ocean Transport
Whatever you are importing or exporting, whether it is food or clothing, chemicals or pharma, our ocean shipping solutions get products to their destination. From selecting the right container for your goods, to loading safely and efficiently at the portside, to monitoring the condition of your container throughout its time at sea, we will support you at every step of your cargo’s journey.

Working with MSC will plug you into a network of experts across more than 675 offices, planning 300 global routes to 520 ports in over 155 countries. We’re committed to offering competitive rates and fast transit times along with personalised support.

Full Portfolio of Shipping Solutions
Dry Cargo
Project Cargo
Reefer Cargo
Liquid Cargo Solutions
Our Trade Services
All Types of Commodities
From what we eat to the cars we drive and everyday items, MSC helps a huge range of industries move goods overseas. We have shipped almost every kind of cargo over more than 50 years. We have grown experience in how to load, store and deliver different kinds of items by ocean transport, along with a comprehensive knowledge of port operations.
All Types of Containers
Our fleet of vessels can hold different types of containers for different kinds of cargo. Our dry cargo containers move goods that can be shipped in a container without the need for any specialised features. We transports refrigerated and frozen goods all over the world, with one of the largest and most advanced reefer container fleets in the world. You can choose from a wide

range of container types, such as 20’, 40’, high cube, open top and collapsible for your breakbulk cargo depending on your needs.

Sea Freight Specialists for Over 50 Years

With decades of experience in our core business – moving containers of goods across the world’s oceans, making sure they are syncronised with inland connections and intermodal transport, when you book with us, you can be sure of a high quality sea freight service.

Industries

Agriculture

Automotive

Chemicals 8 Petrochemicals

Food 8 Beverages

Fruits 8 Vegetables

Mining 8 Minerals

Pharmaceuticals

Plastics 8 Rubber products Pulp, Paper 8 Forestry Products

Retail

eBUSINESS

24/7 Real-time Access to your Freight

MSC is continually enhancing myMSC, our secure eBusiness platform which provides you with 24/7 real-time access to your freight information.

Sustainability

Enabling Logistics Decarbonisation

Decarbonising Logistics

MSC has a key role to play in decarbonising logistics, collaborating with others across the value chain to accelerate solutions at scale, while ensuring a just energy transition. As a key part of our customers’ supply chains, we see ourselves as partners in decarbonisation and enablers of their increasingly ambitious climate targets.

Journey to Net Zero

MSC envisages a net zero future that will see shipping draw on a combination of fuels, and our focus is on ensuring the readiness of our fleet to adopt zero-carbon fuels as they become available. While recognising the uncertainty around their availability we expect zero-carbon fuels will become increasingly available from 2030 onwards following which our industry will see a sharp acceleration in deep emissions reductions.

We believe that synthetic and bio-methanol, green ammonia and bio and synthetic Liquefied Natural Gas (LNG), may become fuel choices for larger ocean-going vessels, while green hydrogen, batteries and fuel cells may be used for smaller vessels and short-sea shipping.

The transition to a low-carbon economy requires broad collective action and productive partnerships among actors across and beyond shipping. By cooperating and collaborating with others, we capitalise and build on the interdependencies between ocean-going and inland logistics as well as other sectors providing fuels, distribution systems and infrastructure. Read more about our partnerships for decarbonisation here.

As part of our decarbonisation pathway, MSC is pursuing transition solutions with the potential to

accelerate the energy transition, including the adoption of low-carbon marine fuels. Newbuildings as well as the retrofitting of existing vessels with flexible fuel burning capability are also key to the transition to sustainable energy sources.

Through our carbon insetting programme we are joining forces with our customers to reduce supply chain emissions using transition fuels such as biofuel. Fossil-based LNG offers opportunities to transition to bio- and synthetic LNG as these become available at scale.

Improving Our Energy Efficiency
As we work towards our net-zero target, we have set goals to progressively reduce our carbon intensity by implementing an array of energy efficiency measures. Our fleet renewal plan includes the delivery of energy efficient newbuildings as well as ongoing implementation of our retrofit programme. Vessel performance and route optimisation are key elements of our energy efficiency programme, ensuring our compliance with IMO regulations including the Carbon Intensity Indicator (CII) introduced in 2023.

Major network overhaul, including newbuildings and a far-reaching retrofit programme.
Fleet digitalisation and a high-frequency data collection system, using machine learning and AI.
Underwater hull inspection and cleaning scheme.
Monitoring and progressive optimisation of all voyages.
Installation of onboard energy-saving devices.
Crew awareness and training on new fuels, technologies and vessel infrastructure.
Deployment of low- and zero-carbon fuels as they become available at scale.
Fostering Inclusive Trade

Addressing Social Challenges
Sustainability report
2022 Sustainability Report
GRI Content Index
Partnerships
Certifications
Our Management Systems are Certified Against the Most Important ISO Standards
Our commitment to compliance with ethical practices and international standards – as well as to continuously improving existing environmental and social processes and practices – is demonstrated by the receipt of various awards and certifications and also by recognised classification societies:
Code of Conduct
Our Management Systems are Certified Against the Most Important ISO Standards
Our commitment to compliance with ethical practices and international standards – as well as to continuously improving existing environmental and social processes and practices – is demonstrated by the receipt of various awards and certifications and also by recognised classification societies:
MSC Code of Business Conduct
MSC Supplier Code of Conduct

MSC Modern Slavery
Transparency Statements
MSC Ship Recycling Policy
At MSC, we have a strong commitment to ensure the safe, sustainable and responsible recycling of our ships at the end of their operational life.
We recognise our industry’s end-of-life challenges and are committed to promoting the respect for labour and human rights and supporting the welfare of our supply chain partners, including those working at the yards where MSC ships are recycled. We take recycling into consideration throughout all stages of a ship’s lifecycle – from newbuildings to operations to dismantling – and incorporate circular economy principles wherever possible.

When an MSC-owned ship reaches the end of its operational life, we seek to make a positive contribution by supporting the local ship recycling industry. In addition to enabling the livelihoods of people working at shipyards, ship recycling-related activities generate local economic growth, with surrounding communities benefiting from local business activity related to the sale of recycled materials. We are also engaging with other stakeholders to improve conditions at shipyards to promote a safe and responsible ship recycling industry, including participation in European Union delegation visits to Alang, India.

We monitor our ships throughout their operational life and conduct annual audits and vetting of shipyards to inform decision-making as our ships approach end-of-life. During the dismantling process, MSC receives regular reports from the shipyard to ensure safe working conditions and practices, as well as proper waste management and handling of all hazardous materials.

When we recycle, the process is conducted in accordance with the MSC Ship Recycling Policy, which stipulates the selection of shipyards that are certified compliant with the technical provisions of the Hong Kong Convention for the Safe and Environmentally Sound Recycling of Ships. In cases where an MSC vessel is sold for further trading, we require that the buyer undertakes safe and environmentally sound recycling practices according to the standards of the Hong Kong Convention.
199.CPE

China Energy Engineering Group Planning and Engineering Co., Ltd. (hereinafter referred to as “CPE”) was established in May 2018 by merging 6 regional design institutes originally subordinate to China Power Engineering Consulting Group Co., Ltd. (CPECC) and 14 provincial design institutes originally subordinate to China Energy Engineering Corporation Limited (CEEC). After the merging, the new company CPE has a registered capital of RMB 10 billion RMB yuan and is wholly-owned by CEEC, while CPECC continues to exist and is administered by CPE.

CPE is positioned as a service provider active in domestic as well as international market offering government departments, financial institutions, investors, developers and project legal entities with integrated engineering and construction solutions, mainly for power planning research, consulting, evaluation, survey, engineering, supporting, EPC general contracting, power project investment and operation, and development of related proprietary technological

products, etc.

With formidable technological strength, complete disciplinary combination, rich experience in engineering practice as well as solid comprehensive management capabilities on top of a massive employment size of nearly 20,000 people, including 12 State Masters in survey and design,

CPE is considered the "Pacemaker" and "National Team" in the power engineering service line and holds a guiding and leading position in electric power survey and design in China. In particular, CPE has outstanding advantages in the fields of ultra-supercritical coal-fired units of 1000MW, conventional islands of nuclear power, clean coal power generation, air-cooling units, UHV AC-DC power transmission and transformation, and offshore wind power.

As a survey and design consulting company with the largest scale in the world, CPE has undertaken the research tasks in one after another major power development and planning program such as national electricity market analysis, power consumption, power grid planning, West-to-East power transmission, nationwide grid coverage, power industry structure optimization and upgrading, etc. It has completed the survey and design works for a large number of domestic power generation projects and power transmission and transformation projects as well as hundreds of power projects in dozens of other countries, and has won dozens of national quality engineering gold awards in the process.

As a constant advocate for company transformation and upgrading, CPE innovatively created the innovative Design-headed EPC Model, with which it successfully completed a series of unprecedented EPC projects such as the first 600MW class and 1000MW class power generation units in China, and has successively won the "General Contracting Project Gold Key Award" for several dozens of times.

Giving full play to the leading role of its academician expert workstation, postdoctoral workstation, and 10 technology research and development centers in technological innovation, CPE has been the unequivocal leader in the research and standardization course of China's electric power survey and design sector. It has undertaken most of the research and standardization tasks in the sector in China, actively fulfilling the objectives for independent development of new power technologies as well as innovation based on introduction and assimilation of advanced foreign technologies. In the process, it has won Grand prize, the First Prize and the Second Prize of National Science and Technology Progress Award for multiple times.

CPE actively participates in the development and implementation of urban rail transit, smart city, sponge city (including water treatment), underground integrated pipe corridor, roads and other infrastructure projects, and has broken through milestones after milestones in the project construction scorebook.

Facing the future, CPE will stick to the guidance of Xi Jinping's new era of socialism with Chinese characteristics ideology and continue to deepen reform, promote the company’s transformation

and upgrading, raise the quality and efficiency levels, and strive to become a world-first-class SMIDIP (scientific, management, innovation, diversification, internationalization, and platform) type transnational engineering company.

Qualification

Main qualifications:

Each member unit has the most complete survey and design qualifications in the power industry, including more than 10 national highest-level qualification licences such as Engineering Design Comprehensive Class-A, Engineering Survey Comprehensive Class-A, and Engineering Consulting Class-A.

Standard

‘One plus Three’ standard system, ‘one’ means the enterprise standard system, and ‘three’ refers to the technical standard system, managerial standard system, and work standard system. By implementing ‘One plus Three’ standard system, CPE aims at creating a standard system that meets the requirements of ‘leading the industry and world first class’.

There are three major objectives of building the company standard system:

(1) Standard system building objectives: to achieve full coverage of all businesses for technical standards, full coverage of the whole procedures for managerial standards, and full coverage of all posts for position standards;

(2) Quality target: to meet the overall requirements of the national and power industry standardization construction, and in line with the technical, managerial and position requirements of multi-integrated operation;

(3) Backbone human resource building objectives: to cultivate standardized backbone teams.

Reputation

Global Top 150 Design Company

World Top 225 Design Company

The World's Largest 250 Contractors

The World's Largest 250 International Contractors

China's Top 80 Contractors

Top 60 Chinese Engineering Design Companies

National High Performance Model Implementing Advanced Enterprise

National Special Award for High Performance Model Implementing Advanced Enterprise

Excellent Enterprise of National Quality Management Team Activities

National Quality Trustworthy Team Building Excellent Enterprise

National Award for Power Industry Enterprise Management Innovation Achievement

CPE(at that time named CPECC) Ranked 4th in the 2017 ENR Top 60 Chinese Engineering Design Companies list and was listed 46th in the Top 80 Contractors list. In 2017, it ranked first in the Top 100 Contract Value Completed by Engineering Contractors among the members of China Survey and Design Association.

Till May 2018, CPE and its affiliates totally won 152 Awards of 2017 Four-Excellence Awards for the Power Industry (including 47 first prizes, 43 second prizes, and 62 third prizes), and won 27 Awards of 2018 China Power High Quality Engineering Award, and 51 Awards of Outstanding QC Activities in the National Electric Power Survey and Design Industry (including 10 first prizes, 18 second prizes, and 23 third prizes).

In the 2016-2017 National Excellent Quality Engineering Awards, there are eight projects designed by CPE winning the National Excellent Quality Engineering Golden Prize, they are the Chongqing Shenhua Wanzhou Power Plant 2×1050MW Project, Zhebei-Fuzhou UHV AC Transmission and Transformation Project, Tibet Changdu Power Grid-Sichuan Power Grid Transmission, Transformation and Connection Project, Huaneng Anyuan ‘replacing small units with large ones’ Project, Jiangsu Guodian Taizhou Secondary Reheat Expansion Demonstration Project, Zhejiang Taizhou Second Power Plant 2×1000MW Project, Guodian Hami Da Nanhu Coal-Electricity Integration 2×660MW Project and Turkey Atlas 2×600MW Iskenderun Thermal Power Plant. In addition, CPE has won more than 30 Awards of National Excellent Quality Engineering.

CPE has received the Special Award for National Science and Technology Progress as the reward of the achievement of participating in the Ultra High Voltage ±800kV DC Transmission research in 2017 and the UHV AC Transmission Key Technology, Complete Equipment and Engineering Application research in 2011.

CPE has also won the National Science and Technology Progress First prize Award as the reward of the achievement of participating in several researches and projects. They are the 600MW Supercritical Circulating Fluidized Bed Boiler Technology Development and Engineering Demonstration Project in 2017, the HVDC Power Transmission Project with Complete Design, Autonomous Technology Development and Engineering Practice study in 2011, China’s First 750kv Power Transmission And Transformation Demonstration Project and its Key Technology Research and the Study on and Application of Ultra-Supercritical Coal-Fired Power Generation Technology in 2007, and the Qinshan 600MWe Nuclear Power Plant Design And Construction project in 2004.

CSR

Independent Innovation and Contribution to Society

Leading technology and building green energy. CPE will make full use of the advantages in power planning research and technology, independent and innovatively, providing customers with quality services, and promoting scientific and technological progress in the energy sector, in order to make an effort in building a stable, economical, clean, safe and sustainable energy supply system.

Scientific Development and Building Harmony

CPE will adhere to the principle of people-oriented and realize the common growth of company with the employees and customer. CPE will also pursue high efficiency, energy saving and high quality projects, in order to ensure the innovative development, harmonious development and sustainable development of the energy industry and the company group. Furthermore, CPE will enhance the sense of social responsibility and will be couragous to undertake social responsibility, and make efforts to social harmony progress.

Core Business

Planning & Study

Survey&Design

EPC

Investment&Operation

Infrastructure

Technology

System

China Energy Engineering Group Planning & Engineering Co., Ltd. (hereinafter referred to as Planning & Engineering Group) always adheres to Technology-led, Innovation-driven development strategy, gradually formed a technological innovation and development system composed by academician expert workstation, engineering technology center, ten professional technology centers (nuclear power, IGCC, air cooling, power station’s cooling tower, high voltage direct current, smart grid, lignite, solar thermal power generation, CFB, flue gas treatment, etc.), production departments and R&D departments in each subordinate units. Planning & Engineering Group has built a high-end research and development platform, improved the level of team’s technological innovation.

Planning & Engineering Group joined National Strategic Alliance for Solar Thermal Industry Technical Innovation initiated by Ministry of Science and Technology and Strategic Alliance for Technological Innovation of China's Thermal Power Industry jointly established by 18 power groups, universities and research institutes. Cooperation with Strategic Alliances provides good opportunities for team’s talents in external cooperation and exchanges. Meanwhile, Planning & Engineering Group applied for the establishment of Station for Post-Doctoral Research, successively established the China Green Development Collaborative Innovation Center in cooperation with Beijing Normal University and other units. The Group cooperated with Hohai University to form a Renewable Research Center, cooperated with Huazhong University of Science and Technology to form a Grid Technology Engineering Research Center, and cooperated with China University of Geosciences to form a Research Center for Electric Geotechnical Engineering. Planning & Engineering Group achieved complementary resources, gradually optimized talents team through its cooperation with universities and research institutes.

China Energy Engineering Group Planning & Engineering Co., Ltd. has one academician expert workstation, five postdoctoral research workstations, two postgraduate workstations and 15 provincial research institutes. They have formed a scientific and technological innovation research and development system with academician expert workstation, post-doctoral research workstation, engineering technology center, Technology Center (including nuclear power technology center, IGCC technology center, air cooling technology center, high voltage DC technology center, smart grid technology center, power plant cooling tower technology center, lignite technology center, solar thermal power technology center, CFB technology center, flue gas treatment technology center) as the main body, subsidiary enterprises’ production and Research and Development institutions as the supplement, innovation and cooperation alliances (including solar thermal power engineering design research center, national solar thermal industry technology innovation industry strategic alliance, China thermal power industry technology innovation strategic alliance, Hehai University New Energy Research Center, China Green Development Cooperative Innovation Center, etc.) as the expedition

With a good scientific and technological innovation system and high-level innovation, China Energy Engineering Group Planning & Engineering Co., Ltd. has successively undertaken the major national scientific and technological research projects, such as sub-topics of the National Science and Technology Support Plan - ‘the optimization and integration of the thermal system of secondary reheater units’, the national energy application technology research and engineering demonstration project - ‘the development and application demonstration of key

equipment for ultra-supercritical coal-fired power generation at 700 ℃ ‘, and ‘the Joint Technical Research and Engineering Demonstration of wind power generation and seawater desalination ’, international cooperation technology project - ’ New Generation of Coal Conversion and Power Generation Technology Oriented to Advanced Coal Technical Cooperation between China and the United States’ and ‘Joint Research on Carbon Dioxide Capture and Storage Technology between China and the United States ’. They have presided over the compilation of major national and industrial standard, such as ‘Design Specifications for Large and Medium-sized Thermal Power Plants ’, ‘ Design Specifications for 1000kV Overhead Transmission Lines’, and the compilation of the Electric Power Engineering Design Manual has been officially launched at the end of 2015 and is about to complete.

With excellent technology, system integration and integration capabilities, China Energy Engineering Group Planning & Engineering Co., Ltd. is exploring and researching in various forward-looking areas, leading the technological development of the industry. Through sustained scientific and technological investment, the achievements of scientific and technological innovation in many fields of power engineering have reached the international advanced and domestic leading level. Firstly, in the research of AC/DC transmission and transformation engineering design technology, they have reached the international leading level; Secondly, in the research and application of ultra-supercritical unit power plant design technology, they have reached the international advanced level; Thirdly, they have a comprehensive grasp of the conventional island design technology for nuclear power plants; Fourthly, they have made major breakthroughs in the field of lignite power generation. Fifthly, their research on air cooling localization technology of large thermal power units has broken the monopoly of foreign companies, and has made breakthroughs and innovations. Sixthly, the design technology of large CFB boiler system has been introduced, digested and absorbed to realize the localization of large CFB boiler. Seventhly, the research on design technology of super-large cooling tower fills the gap in China; Eighthly, the research achievements of energy-saving, water-saving and environmental protection technology of power plants have reached the advanced level in China; Ninthly, the research on new energy design technology such as large-scale wind power has been widely applied; And tenthly, the informatization of engineering design has reached the leading level in China.

200. Sinoma (Suzhou)

Sinoma (Suzhou) Construction Co., Ltd. [hereinafter referred to as 'Sinoma (Suzhou)' for short] belongs to Sinoma International Engineering Co., Ltd. subordinated to China National Building Material Group Corporation, stock code: 600970. Sinoma (Suzhou) is a large engineering company with multiple national first-class construction qualification such as smelting engineering construction general contracting etc., certificates for overseas project contracting and which is identified as new high-tech enterprise. It’s certified by “three standard embraced in one system” integrated management system of ISO9001 quality management system, ISO14001 environmental management system and GB/T28001 occupational health and safety system.

Sinoma (Suzhou) mainly engaged in the general contract of large and medium scale NSP (New

Type Dry Process) cement production line at home and abroad, cement equipment manufacturing, operation & maintenance of cement plant, overhaul and medium repair of cement plant, import & export trade, etc.

Culture

Enterprise culture is the soul of an enterprise. The 60 years' accumulation and precipitation creates the unique Sinoma (Suzhou) enterprise culture

Upon the principle of people-first, with the enterprise features of high staff turnover rate, long working time and high labor intensity, Sinoma (Suzhou) keeps the working way of strong and tough, fighting spirit and willing to sacrifice on the basis of the spirit of "wolf culture". Meanwhile Sinoma (Suzhou) highlights the "family culture" to strengthen the team idea and harmonious working relationship on account of the isolation from the family, loneliness and hard living condition.

Enterprise tenet: thoughtful and timely, high-quality and high-efficient, win-win cooperation, beginning to end

Management principle: faith-keeping, service first

Enterprise spirit: making it better, striving for excellence

Guideline of quality, environment and occupational health and safety: people-first and faith-keeping & law-abiding for the continual supply of satisfied products, pollution prevention and safety & healthy for the improving management performance.

Qualification

Bank Credit Grade AAA

Environmental System Certification

Safety Production License

OHS System Certification

Honor

Top 500 of China Building Material Enterprise

Luban Award(Vietnam Phuc Son)

MAIN BUSINESS

Cement Turnkey Project

Sinoma (Suzhou) is equipped with complete cement engineering industry chain, and has the ability to integrate the whole process system from cement project consultation, engineering

design, technical equipment research and development, equipment manufacturing, equipment complete sets and supply, civil construction, equipment installation, production and debugging to production operation and maintenance.

The company has always implemented the advanced, reliable, energy-saving, environmentally protection, as well minimizing the investment cost. By actively adopting new technologies and new equipment as well as providing individualized designs based on the different environments of the project and the individual requirements of the employer, the company can use the optimized design to maximize the employer’s investment benefits while meeting the functional requirements.

By making full use of the experiences in building material industry over the years, the company can provide specialized and systematic centralized procurement services. The large-scale procurement of multiple general contracting projects can greatly reduce the procurement cost of equipment. With the powerful on-site construction resources, the company can realize large-scale equipment delivery and production, thus saving the costs for manufacturing and transportation. By monitoring the entire process of equipment manufacturing through a professional supervision team for equipment manufacturing, we can ensure the equipment quality and meet the schedule. At present, the company’s procurement covers almost all the professional equipment of the new dry-process cement production line, including large vertical mills, roller presses, grate coolers, crushers, rotary kilns, ball mills, kiln tail pre-decomposition systems, dust collection equipment, powder selection machine, stacker and reclaimer, etc.

Besides a large number of experienced and professional talents in electrical, mechanical, civil engineering and other fields, Sinoma (Suzhou) also boasts a broad range of skilled personnel in terms of contract management, risk control, planning and statistics, logistics and transportation, material management, financial management and other areas. In the meantime, it is home to many skilled crane workers, furnace builders, welders and other professional operators, while enjoying varied resources and business qualifications with regard to smelting, mechanical and electrical equipment installation, steel structures, furnaces, lifting equipment, etc.

The company has achieved the goal of green and intelligent construction by the emphasis on digital transformation and upgrading, continuous introduction of BIM, the Internet, the Internet of Things, etc. and the digital management of the whole construction process. With scientific and systematic methods of management, the company is capable of gradually shortening construction cycle, reducing the investment and improving the quality of EPC project. Hence, the

business idea of win-win relationship with the employer is fully implemented.

Operation & Maintenance

Based on the main business, with the idea of building sustainable projects, the company accelerates to upgrade the "integration of construction and operation". The company now has a number of professional production debugging and operation management teams, but also has a wide range of human resources network, can undertake the personnel organization, cement clinker production, equipment operation and maintenance, equipment overhaul and spare parts supply, etc. of the production line. The company has worked on 14 cement production lines in 9 countries, especially in Iraq, and has undertaken the operation and maintenance business of all the executed EPC projects. The rapid development of operation and maintenance business not only provides customers with more comprehensive, systematic, high-quality and efficient services, but also lays a foundation for the company's sustainable development in the future.

Related Diversification

After more than 20 years of overseas development，Sinoma （Suzhou）has not only explored and built new models for cement engineering internationalization construction, but also been deeply committed into non-cement engineering construction business relying on its global brand influence, rich overseas project management experience and its technology and talent advantages accumulated over such a long time. It has successfully undertaken and performed the contracts for oil refinery, power plant, roads and bridges construction, factories construction and industrial parks construction projects.

At the same time, the company explores to expand the trade logistics business, focusing on the trade of clinker, cement, coal, gypsum, color tile, coke, aggregate and other bulk building materials, to provide customers with supporting logistics, customs clearance, warehousing and other comprehensive value-added services.

New Industries

The company has established the global development strategy with two developing directions of localization and diversification, four developing areas of Africa, the Middle East, Southeast Asia and Central Asia, and six developing bases including but not limited to Nigeria, Tanzania, Ethiopia, Zambia, Iraq, Indonesia, and Vietnam.

The company adheres to the "localization operation" and "participation in the CNBM investment of international industries made" simultaneously. It has given full play to its first-mover advantage of “going global” as a building material engineering company. On the basis of industrial parks, lightweight building material and warehouse logistics, Sinoma (Suzhou) focuses on global strategy of new industries investment with CNBM advanced enterprises and achieves the new growth point constantly.

Talent Philosophy

Core ideology: opportunity and stage for people who want to do something, social status and

better rewards for people who is successful.

Principle of who is moral and intelligent: talent selection and employment should be as the comprehensive assessment and the requirement of combining ability with character.

Principle of effect theory: staff employment is depending on the ability and competence no matter education background, age, identity, etc. Now, the average age of technical and management staff is about 35 and workshop directors 30. The personnel selected as the principle of effect theory is the main force of enterprise development.

Principle of practice first: various professional personnel will be arranged to the project site for practice and development at the beginning of their career. Promotion will be equal to everyone by exams.

Principle of survival of fitness: the one who is the best will be entrusted with an important post.

Principle of opportunity for everyone: consider and choose the most reasonable and proper post for everyone in consideration of his/her character, like the capabilities, specialties, experiences, personalities, etc to keep the sustainable, rapid and effective development of an enterprise.
