# Supplementary material for: Exploring the narrative landscape: The discursive construction of identity for Chinese enterprises in Africa
Source: PLoS One. 2025 Feb 28;20(2):e0314285. doi: 10.1371/journal.pone.0314285 (PMC11870363; doi:10.1371/journal.pone.0314285)
Supplement: Attachment — Submitted filename: Discursive construction review.docx [file pone.0314285.s004.docx]

The study entitled “Exploring the narrative landscape: the discursive construction of identity for Chinese enterprises in Africa” examines the strategic employment of language in order to construct corporate identities and communication practices of Chinese companies in Africa. This study is a significant contribution as it provides an extensive comprehension of discursive identity construction of companies in cross-cultural context. Overall, this research article flows logically and the research undertaken is contextualized clearly. The introductory section offers relevant background of the study, highlighting the rationale and significance of this research. The further subsections of introduction also discuss the selected frameworks. The methodology used is described in detail and aligns with the research questions. Further, the results and discussion sections are up to the mark and reflect authenticity of this research. However, I would recommend some minor improvements before the publication. The following issues need attention:

- The authors are advised to cite some studies in section 3 “Three-dimensional model of critical discourse” and section 6.2.3 “State-owned vs Private Enterprises” in order to strengthen the arguments.

Afzaal, M. (2023). A corpus-based analysis of discourses on the belt and road 209 initiative: Corpora and the belt and road initiative (Vol. 10). Springer Nature.

Afzaal, M., Hu, K., Chishti, M. I., & Khan, Z. (2019). Examining Pakistan news media discourses about China-Pakistan economic corridor: A corpus-based critical discourse analysis. Cogent Social Sciences, 5(1), 1-18. <https://doi.org/10.1080/23311886.2019.1683940>

Wei, F. E. N. G., & WU, D. D. (2016). State-owned or Otherwise: Dialogic Construction of Corporate Identities by Chinese Banks on Sina Weibo. *Intercultural Communication Studies*, *25*(2).

Fu, H., & Zhu, H. (2022). Discursive construction of corporate identity through websites: An intercultural perspective on the commercial banks of the United States and China. *Frontiers in Psychology*, *13*, 947012.

- Mention years of the selected frameworks in abstract.
- Avoid the frequent use of contraction (‘s) as in Line 44, 98, 104, 133, 140, 155, 157.
- It is suggested to initiate the paragraphs with articles or cohesive devices as in line 365, 408, 420, 456.
- Use article (the) in lines 451 (the example 25), 486 (the terms), 499, 500,515, 538, 550, 626, 661.
- Use plural form (the examples) in line 485
- Re-structure lines 566-567, 716-717 and 583-586.
