# Supplementary material for: Exploring the narrative landscape: The discursive construction of identity for Chinese enterprises in Africa
Source: PLoS One. 2025 Feb 28;20(2):e0314285. doi: 10.1371/journal.pone.0314285 (PMC11870363; doi:10.1371/journal.pone.0314285)
Supplement: Attachment — Submitted filename: Response to Reviewers.docx [file pone.0314285.s006.docx]

**Dear Reviewers,**

On behalf of all contributing authors, I would like to express our sincere gratitude for your thoughtful and constructive feedback on our article, *“Exploring the Narrative Landscape: The Discursive Construction of Identity for Chinese Enterprises in Africa.”* We have carefully reviewed each of your suggestions and incorporated the necessary revisions into the manuscript. Your insightful comments have been instrumental in enhancing the clarity, coherence, and overall quality of our work, and we hope the revised version aligns with your expectations.

Below, we provide a detailed, point-by-point response to your comments, with the reviewer’s remarks italicized for clarity. All revisions have been marked using tracked changes in the resubmitted manuscript to facilitate your review.Yellow highlighting indicates the original text, and red highlighting indicates the revised text.

Response to Reviewer 1:

1. ***The authors have provided a relatively clear definition and theoretical background for “Corporate Identity” at the beginning of the literature review. However, the discussion on how scholars approach corporate identity from various perspectives could benefit from further elaboration.***

Response for the first comment:

Thank you for your insightful feedback regarding the need for further elaboration from multiple perspectives. In response, we have expanded the discussion to reflect the multidisciplinary nature of corporate identity research. Specifically, we have incorporated insights from economics, international relations, and journalism studies to demonstrate the diverse approaches scholars take in exploring corporate identity.

The revised section now discusses how corporate identity influences brand equity and financial performance from an economic perspective, how multinational corporations leverage corporate identity to shape geopolitical influence within the framework of international relations, and how corporate identity is represented and communicated in the media through the lens of journalism studies.

By integrating these perspectives, the expanded discussion offers a more holistic understanding of corporate identity, aligning with your recommendation for further elaboration. Your feedback has been invaluable in enhancing the depth and clarity of this section.

Please refer to the revised draft: lines 106-119.

2. ***The statement, “Despite the extensive research on corporate identity across diverse fields such as economics, international relations, and journalism, there is a noticeable shortfall in applying discourse analysis methods to corporate identity studies,” is too brief to capture the breadth of existing research. Offering more detail on how different researchers examine this concept across disciplines would help readers better understand the research context.***

Response for the second comment:

Thank you for your insightful suggestion. In response, we have expanded the discussion to provide a more detailed account of the application of discourse analysis in corporate identity research. While discourse analysis has offered valuable insights into this field, much of the existing research tends to focus on specific industries and individual corporate strategies, leaving broader, cross-industry evaluations underexplored (Vaara, 2008; Spanos & Lioukas, 2001). Furthermore, studies examining corporate profiles on official websites remain limited, despite these platforms playing a crucial role in constructing and projecting corporate identity (Melewar, 2003; Tourky et al., 2021). The revised section aims to address these gaps and emphasizes the importance of expanding the scope of corporate identity research beyond industry-specific approaches.

Please refer to the revised draft: lines 120-132.

3. ***Similarly, the discussion of Kim and Rader’s corporate communication strategy typology requires further elaboration. I recommend incorporating more detailed information about the development background, theoretical underpinnings, and diverse applications of this typology in existing literature. This will provide readers with a clearer understanding of the theory’s strengths and its relevance to your study.***

Response for the third comment:

Thank you for your suggestion. We have expanded the discussion of Kim and Rader’s corporate communication strategy typology to provide further detail on its theoretical foundation and practical applications. Specifically, we included examples of how this typology has been applied to assess integrated marketing communications, compare corporate advocacy with CSR strategies, and evaluate communication approaches across industries (Cornelissen, 2020; Yuan & Sheng, 2018; Kim, Park, & Kim, 2019; Chen & Tao, 2020). These examples illustrate how the typology supports the analysis of messaging consistency, aids in managing public perception, and addresses key communication challenges. This additional context clarifies the typology’s relevance and utility, in line with your recommendation.

Please refer to the revised draft: lines 182-201.

4. ***In Section 5.1 Data Collection, it would be beneficial to include the specific methods used for data collection, such as whether the data was gathered through web crawling or manual copying and pasting. Additionally, presenting the data in a tabular format would enhance clarity. For example, you could include the number of texts and tokens collected from different types of corporates.***

Response for the fourth comment:

Thank you for your helpful suggestion. In response, we have clarified the specific method employed for data collection, specifying that the data was manually gathered from the official websites of Chinese companies operating in Africa to ensure both accuracy and relevance. Furthermore, we have reorganized the data into a tabular format, as recommended, with Table 2 offering a detailed breakdown of the number of texts and tokens collected from various types of corporations.

These revisions aim to improve the clarity of the data collection process and enhance the overall presentation of the information.

Please refer to the revised draft: lines 204-206,213-217.

5. ***The sentence “delineates a primary identity unique to professional service and product providers, setting them apart from other sectors” is unclear. I recommend rephrasing it for clarity.***

Response for the fifth comment:

Thank you for pointing out the need for clarification. In response, we have rephrased the sentence to enhance clarity. The revised sentence now specifies that the first category defines the core identity of professional service and product providers, distinguishing them from other sectors by emphasizing their fundamental actions and general business practices. These changes aim to convey the intended meaning more clearly and ensure the sentence is accessible to readers.

Please refer to the revised draft: lines 279-284.

6. ***There are two concerns regarding the data presented in Table 4. First, although Category 1 includes the semantic domains A1.1.1 and I2.1, Table 4 only displays high-frequency words from the A1.1.1 domain. Please clarify whether the omission of data related to I2.1 was intentional or if it was an oversight in the “semtag” column. Second, please specify whether the terms “projects,” “operation,” and “production” are the highest frequency words within this semantic domain and provide their respective frequencies. If these terms are not the highest frequency words, please explain why they were selected for analysis.(Similarly, frequencies for high-frequency words in the other categories should also be included.***

Response for the sixth comment:

Thank you for your valuable feedback. In response to your concerns, we have made several key clarifications. First, we expanded the analysis to encompass all semantic domains within each category. Specifically, we incorporated an analysis of I2.1 (lines 338–371) in addition to A1.1.1. Moreover, we included an analysis of M7 [places] (lines 542–588) within Category 4 to ensure comprehensive coverage. Second, we clarified that the terms “projects,” “operation,” and “production” are high-frequency, representative terms, and we have now provided their respective frequencies in the revised manuscript. Additionally, we included the frequencies of other high-frequency words across the remaining categories to enhance transparency and consistency.

Please refer to the revised draft: lines 339-371,545-591.

7. ***It would be helpful to provide a brief explanation of the calculation method used for the relative frequency presented in Table 2.***

Response for the seventh comment:

Thank you for your insightful suggestion. In response, we have included a brief explanation of the calculation method used to determine the relative frequency presented in Table 2. Specifically, we identified 377 unique semantic domain types, which collectively appear 74,086 times throughout the dataset. Our analysis focuses on the top ten domains, as detailed in Table 3.

The relative frequency is calculated using the following formula:

Relative Frequency= ( $\frac{Frequency of a specific semantic domain}{Total frequency of all semantic domains}$ )×100

This calculation highlights the prominence of each domain within the dataset and provides a clearer understanding of its significance.

Please refer to the revised draft: lines 251-258.

8. ***The analysis of high-frequency words in Section 6.1.2 could be more in-depth. A more thorough examination of specific verbs would be advantageous. For instance, analyzing the object of the verb “support” and the particular areas in which Chinese firms have provided assistance would enhance the depth of your analysis.***

Response for the eighth comment:

Thank you for your insightful feedback. In response to your suggestion for a more in-depth analysis of high-frequency words in Section 6.1.2, we have expanded the examination of the term “support,” which functions both as a noun and a verb. This analysis illustrates how companies strategically employ the term to differentiate between the sources and targets of assistance, effectively framing both their needs and offerings. These additions enhance the depth of the analysis and provide a clearer understanding of the communicative strategies at play.

Please refer to the revised draft: lines 386-434.

9. ***Table 5 is titled "Top 20 Keywords of Tagset Z2 [Geographical Names]." Generally, the term "keyword" denotes words that occur more frequently in the focus corpus compared to a general language or reference corpus. However, it appears that the use of "keywords" in your title may not align with this conventional definition. Could you please clarify how "keywords" is defined and applied in this context?***

Response for the ninth comment:

Thank you for your observation regarding the terminology used in Table 5. To clarify, the term “high-frequency words” in this context refers specifically to words that occur most frequently within the semantic domain Z2 [Geographical Names] in the CCIA corpus. This usage focuses solely on frequency within the targeted corpus and does not involve a comparison to a general or reference corpus, which is typically associated with the conventional definition of “keywords.”

Please refer to the revised draft: lines 449-451.

10. ***It appears that the methodology for assigning CSR and CAB scores to each company in Section 6.2.1 is not fully clear. I recommend offering a more detailed explanation and a specific example to clarify this process. For instance, how is a single CSR or CAB score determined for multiple texts collected from each company’s website? Additionally, reporting on the reliability of the scores would be valuable. If the scoring involved multiple evaluators, including information on inter-rater reliability，such as Cohen’s kappa coefficient， would strengthen the robustness of your analysis.***

Response for the tenth comment:

Thank you for your valuable feedback. In response to your comments regarding the methodology for assigning CSR and CAB scores in Section 6.2.1, I have clarified the process.

As outlined in Table 1, we employed specific CSR and CAB indicators for the analysis. Two trained coders independently evaluated each company’s website and assigned scores to each indicator. Any discrepancies between the coders were resolved through consultation with a third evaluator to ensure consistency. The inter-rater reliability, assessed using Cohen’s kappa coefficient, was high (0.85), indicating a strong level of agreement between the coders. For example, the China Road and Bridge Corporation (CRBC) received a CSR score of six and a CAB score of three, suggesting a strategy primarily focused on corporate social responsibility. Additional details regarding the annotation process can be found in Tables 12 and 13.

Please refer to the revised draft: lines 777-798.

Response to Reviewer 2:

Dear Reviewer:

On behalf of all contributing authors, I would like to express our sincere gratitude for your insightful comments on our article, *“Exploring the Narrative Landscape: The Discursive Construction of Identity for Chinese Enterprises in Africa”*. Your feedback has been invaluable in enhancing the quality of our work, and we believe that the revisions now effectively incorporate the improvements you recommended.

1. ***Mention years of the selected frameworks in abstract.***

Response for the first comment:

Thank you for your suggestion. In response, we have included the publication year of the selected frameworks in the abstract to provide clearer context and enhance the relevance of the discussion.

Please refer to the revised draft: lines 44-46,77, and 146.

2. ***The authors are advised to cite some studies in section 3 “Three-dimensional model of critical discourse” and section 6.2.3 “State-owned vs Private Enterprises” in order to strengthen the arguments.***

Response for the second comment:

Thank you for your valuable suggestion. In response, we have incorporated relevant studies to strengthen the arguments in Sections 3 and 6.2.3.

(1) In Section 3, we referenced Afzaal (2023), who emphasizes that Critical Discourse Analysis (CDA) serves as a comprehensive tool for unpacking complex geopolitical narratives. For example, Afzaal et al. (2019) applied Fairclough’s model to examine media constructions of the China-Pakistan Economic Corridor (CPEC), demonstrating how discursive practices shape both public perceptions and geopolitical narratives.
(2) In Section 6.2.3, we incorporated insights from Wei and Wu (2016), who observe that state-owned enterprises (SOEs) frequently align their identities with national development goals, portraying themselves as champions of the state’s socio-economic vision. Furthermore, Fu and Zhu (2022) highlight that Chinese SOEs often employ hierarchical messaging to maintain a consistent and credible corporate identity.

These additions enhance the discussion by providing a stronger theoretical foundation and more in-depth empirical insights to support the arguments presented in these sections.

Please refer to the revised draft: lines 137-138,152-155,889-891 and 892-894.

3. ***Avoid the frequent use of contraction (’s) as in Line 44, 98, 104, 133, 140, 155, 157.***

Response for the third comment:

We understand your concern regarding the use of contractions in academic writing, as they can sometimes detract from the formal tone expected in scholarly articles. Upon reviewing the specified lines, we have taken the following steps:

(1) In Line 44 and 133, the contraction “Norman Fairclough’s Three-dimensional Model” has been replaced with “the Three-dimensional Model of Fairclough” and “the Three-dimensional Model proposed by Fairclough” to maintain a formal tone.
(2) In Line 98 and in Line 104, the contraction “organization’s” has been revised to “... of organizations” for consistency and to align with academic conventions.
(3) In Line 140, “Fairclough’s model” has been changed to “the model of Fairclough” (Line 153 in the revised manuscript) to ensure formality.
(4) In Line 155, the contraction “company’s” has been removed to avoid redundancy.

Please refer to the revised draft: lines 44, 146 and 157.

4. ***It is suggested to initiate the paragraphs with articles or cohesive devices as in line 365, 408, 420, 456.***

Response for the fourth comment:

We sincerely appreciate your valuable feedback regarding our application of framing analysis. In response, we have made the following revisions to ensure accuracy and consistency:

(1) In Line 365, the phrase “Examples 15 and 16” has been revised to “The examples 21 and 22.”
(2) In Line 408, the phrase “Example 19 and 20” has been replaced with “The examples 29 and 30.”
(3) In Line 420, the phrase “The examples 21 and 22” has been updated to “The examples 31 and 32.”
(4) In Line 456, the phrase “Examples 36 and 37” has been corrected to “The examples 36 and 37.”

Please refer to the revised draft: lines 509,601,613 and 650.

5. ***Use article (the) in lines 451 (the example 25), 486 (the terms), 499, 500,515, 538, 550, 626, 661.***

Response for the fifth comment:

We appreciate the reviewer’s insightful observation regarding the use of articles. In response, we have made the following corrections:

(1) In Line 451, “Example 25” has been revised to “The example 35.”
(2) In Lines 486, 500, and 515, “Terms” has been updated to “The terms.”
(3) In Line 499, “Examples 31, 32, and 33” has been revised to “The examples 41, 42, and 43.”
(4) In Line 538, “Examples 36 and 37” has been modified to “The examples 46 and 47.”
(5) In Line 550, “Example 38 and 39” has been corrected to “The examples 48 and 49.”
(6) In Line 626, “Companies” has been changed to “The companies.”
(7) In Line 661, “Variations” has been updated to “The variations.”

We appreciate your attention to detail, which has contributed to improving the clarity and precision of our manuscript.

Please refer to the revised draft: lines 645,682,696,711,695,734, 746,835,870 .

6. ***Use plural form (the examples) in line 485***

Response for the sixth comment:

Thank you for your valuable feedback regarding the use of the plural form. In response, we have revised Line 485, changing “Example 28, 29, and 30” to “The examples 38, 39, and 40.”

We appreciate your attention to detail, which has helped enhance the clarity of our manuscript.

Please refer to the revised draft: line 681.

7. ***Re-structure lines 566-567, 716-717 and 583-586.***

Response for the seventh comment:

Thank you for your valuable suggestion. In response, we have revised several sentences to enhance clarity and precision:

(1) Original sentence: The analysis of semantic domains related to the activities and perceptions of Chinese enterprises in Africa reveals a diverse range of identities they embody.

Revised sentence: This study reveals the diverse identities that Chinese enterprises construct in Africa by analyzing the semantic domains related to their activities.

(2) Original sentence: This analysis further illuminates the interconnectedness between the indicators of Corporate Social Responsibility (CSR) strategies and Corporate Ability (CAb) strategies, along with the identities they embody.

Revised sentence: This analysis also illuminates how Corporate Social Responsibility (CSR) and Corporate Ability (CAb) strategies intertwine to shape corporate identity.

(3) Original sentence: Rooted in China’s collectivist ethos, these enterprises prioritize ties with their homeland, emphasizing their Chinese origin to reinforce their identity.

Revised sentence: Grounded in China’s collectivist culture, these enterprises strategically leverage their Chinese origins to reinforce their identity.

Please refer to the revised draft: lines 761-762,799-81,929-931.

Thank you again for your valuable comments and suggestions. We look forward to your feedback on the revised manuscript.

Sincerely,

Liu Wenyu

On behalf of all contributing authors
